# Chiroptical Switching by Stereoselective Photochemical Sigmatropic Rearrangements: Experimental Realization of Calderoselectivity

**DOI:** 10.1002/anie.9477381

**Published:** 2026-09-30

**Authors:** Anne Krenkel, Frederike Beyer, Malte Feßner, Trung Tran Ngoc, Tobias Rüffer, Dominik Hornig, Martin Breugst, Christian Merten, Johannes F. Teichert

**Affiliations:** ^1^ Institut Für Chemie Fakultät für Naturwissenschaften Technische Universität Chemnitz Chemnitz Germany; ^2^ Fakultät Für Chemie und Biochemie Organische Chemie II Ruhr‐Universität Bochum Bochum Germany

**Keywords:** chiroptical switches, pericyclic reactions, photochemistry, sigmatropic rearrangements, stereoselectivity

## Abstract

The stereochemical outcomes of pericyclic reactions provide direct insight into the geometry and directionality of concerted bond reorganization. Although torquoselectivity has emerged as a defining concept for electrocyclizations, an analogous principle for sigmatropic rearrangements on a single cyclic π system has remained unknown. We report that a homoannulene‐based photoswitch undergoes an auxiliary‐controlled, stereoselective photochemical [1,11]‐sigmatropic rearrangement, thereby establishing selectivity over directional migration on a single cyclic π framework. We coin the term calderoselectivity for such an unreported stereoselective sigmatropic rearrangement. The switching process is reversible, proceeds to photostationary mixtures of homoannulene and *iso*‐homoannulene isomers, and furnishes diastereomeric products in a 56:44 ratio; combined chiral HPLC, NMR, ECD, and DFT studies assign the major directionality of acyl migration in pro‐*R* directionality. This study provides the first experimental realization of calderoselectivity and reveals a direct analogue in sigmatropic rearrangements to torquoselectivity with implications for both mechanistic physical organic chemistry and chiroptical switch design.

## Graphical abstract

Calderoselectivity is established as a stereochemical principle for sigmatropic rearrangements. A homoannulene‐based chiroptical photoswitch undergoes a photochemical [1,11] sigmatropic rearrangement with control of the acyl movement on top of the cyclic π system. This reveals the stereochemical counterpart to torquoselectivity in electrocyclization reactions.

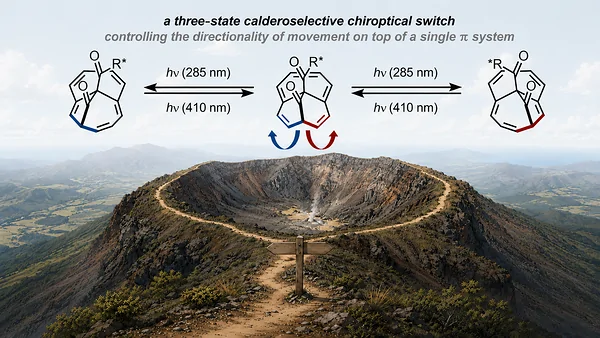

## 1 Introduction

Pericyclic reactions continue to serve as mechanistic proving grounds as their stereochemical outcomes are intimately coupled to the underlying orbital symmetry and thus the topology of concerted bond formation and cleavage [1, 2, 3]. In this vein, stereoselective Diels‐Alder reactions (as prototypical cycloadditions) have been studied with regards to differentiation of the two hemispheres of the π systems involved, resulting in diastereoselectivity and enantioselectivity (Scheme 1a) [4, 5, 6, 7]. In electrocyclizations, as yet another class of pericyclic reactions, torquoselectivity stands as an illustration of how directional motion in a π system can be translated into predictable stereochemical control [8, 9, 10]. Remarkably, no corresponding concept has been realized for sigmatropic rearrangements on top of single π systems, even though these processes are governed by similarly stringent orbital and geometric constraints. In a suitable cyclic π system, the control of the directionality of a sigmatropic rearrangement could reveal the direct counterpart of torquoselectivity for this class of pericyclic reactions. The respective (non‐stereoselective) rearrangements are known in cyclopentadiene motifs [11, 12, 13, 14]. However, to the best of our knowledge no study addressing the stereocontrol of the directionality of rearrangement on top of a single π system, which we coin “calderoselectivity” [15], has appeared [16, 17, 18, 19, 20, 21, 22, 23, 24, 25, 26].

**SCHEME 1:**
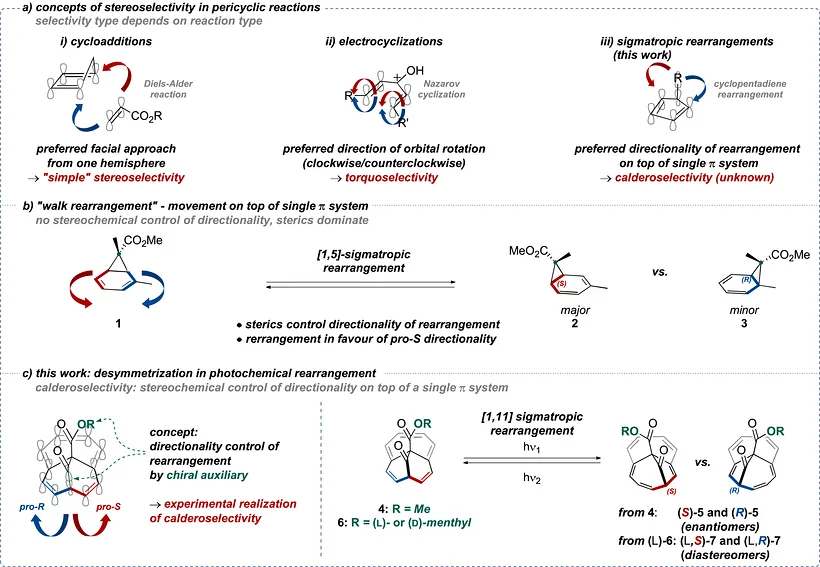
Conceptualization of calderoselectivity in sigmatropic rearrangements. (a) Types of stereoselectivities in pericyclic reactions. (b) Directionality of “walk rearrangement” determined by sterics, as example for rearrangement on top of a single π system. (c) This work: Constructing a calderoselective chiroptical switch by control of directionality of photochemical [1,11]‐sigmatropic rearrangement.

The so‐called “walk rearrangement”, describing the thermal [1,5]‐sigmatropic rearrangement of norcaradiene **1** to **2,** and **3** is a rare case of a migrating group on top of a single π system (Scheme 1b) [27, 28, 29, 30]. Dominated by steric factors rather than stereochemical discrimination, the walk rearrangement preferably takes place away from the methyl group as steric bias (i.e., toward **2** rather than **3**) [27].

If realized in a reversible photochemical setting, calderoselectivity would provide a design principle for chiroptical switching [31, 32, 33, 34, 35, 36, 37]. Therefore, the envisaged approach bears importance for single molecule information storage entities in molecular nanotechnology [38, 39, 40, 41].

We have recently reported on a structurally and mechanistically unprecedented molecular switch (**4**⇆**5**, Scheme 1c) based on a neutral homoaromatic hydrocarbon framework [42]. Upon irradiation, homoannulene **4** undergoes an unusual reversible photochemical [1,11]‐sigmatropic rearrangement to *iso*‐homoannulene **5**, which could also be described as acyl migration on top of a single π system in a suprafacial manner [42]. In agreement with the Woodward‐Hoffmann rules [1, 2], no thermal back‐isomerization (**5**→**4**, “back switching”) is allowed, giving rise to a p‐type photoswitch, in which both switching processes are exclusively initiated in a photochemical manner [43, 44, 45]. The absence of any thermal back‐isomerization makes this photoswitch relevant for long‐term information storage on the molecular level [38, 39, 40, 41].

From a stereochemical perspective, the switching process of the achiral methyl ester **4** leads to a racemate (*R*)‐**5** and (*S*)‐**5**, and thus—if stereoselectivity was controlled—would manifest a desymmetrization process [46]. To construct a model system for calderoselectivity (and thus a chiroptical switch), one would need to control the directionality of the acyl movement on top of the π system.

Incorporation of a chiral alcohol, such as readily available menthol [47, 48, 49] at the ester moiety as a UV‐silent chiral auxiliary [42, 50] allows for a symmetry‐breaking process during the photochemical rearrangement (Scheme 1c). In contrast to the walk rearrangement (Scheme 1b), such an approach is devoid of any symmetry‐breaking substituents directly on the π system involved in the rearrangement [51]. Additionally, this approach bears the advantage that the calderoselective switching process of **6** would be facile to read out, as the products (*S*)**‐7** and (*R*)**‐7** are diastereomers (for example, (L)‐**6** would lead to the diastereomers (L,*S*)‐**7** and (L,*R*)‐**7**) [52, 53, 54]. We herein describe the realization of the envisioned model system for a calderoselective chiroptical switch building upon a stereoselective photochemical [1,11]‐sigmatropic rearrangement of homoannulenes. The stereochemical outcome is corroborated by chromatographic and spectroscopic evidence. In particular, ECD spectroscopy combined with TD‐DFT calculations is used to address the directionality of the calderoselective photochemical rearrangement.

## 2 Results and Discussion

The synthesis of the menthyl‐bearing precursor **6** emanates from naphthalene (**8**) and follows a previously published route to *cis*‐acid **9** (Scheme 2) [42, 50]. Several of the intermediate steps have now been improved in terms of yields and selectivity; please see the Supporting Information for optimized synthetic protocols. Through activation of *cis*‐**9** by carbonyl diimidazole, both commercially available enantiomers of the menthol auxiliary could be introduced via Claisen‐like‐condensation using the corresponding acetates to give both enantiomers of *β*‐ketoester **10** in good yields (72% and 65%, respectively). Subsequent diazo transfer delivered diazo acetoacetates **11** in very good yields of 98%. Gratifyingly, the Buchner dearomatization catalyzed by copper(II) hexafluoroacetoacetate led to the corresponding asteranes **12** in good yields, followed by an oxidation with DDQ to give the desired chiral homoannulenes (L)‐**6** and (D)‐**6**. X‐ray crystal structure analysis of (D)‐**12** and (L)‐**6** fully supported the proposed connectivity [55, 56]. From these structures, it becomes evident that the chiral menthol auxiliary resides fairly distant from the homoannulene π system, at which the photochemical rearrangement will take place.

**SCHEME 2:**
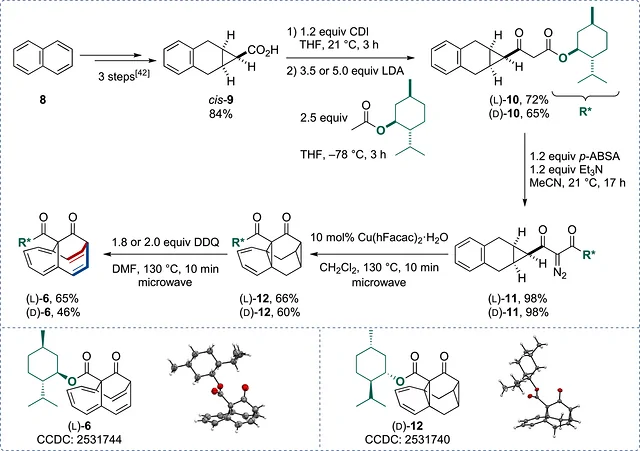
Synthetic route to enantiopure menthyl homoannulenes (L)‐**6** and (D)‐**6**. Abbreviations: CDI = 1,1′‐carbonyldiimidazole, DDQ = 2,3‐dichloro‐5,6‐dicyano‐1,4‐benzoquinone, DMF = dimethylformamide, EDA = ethyl diazoacetate, hFacac = hexafluoroacetylacetonate, LDA = lithium di*iso*propylamide, *p*‐ABSA = *para*‐acetamido‐benzenesulfonyl azide, OTf  = triflate, trifluoromethansulfonate. The descriptors (L) and (D) refer to the stereochemistry of the menthol enantiomer employed in its synthesis and are used throughout the manuscript to ensure unambiguous naming of the respective stereoisomers. Crystal structures are depicted by ORTEP thermal ellipsoids on the 50% probability level. Four molecules of (L)‐**6** in the unit cell. For clarity, only one molecule is shown.

With both enantiomers of the menthyl homoannulene **6** in hand, we conducted experiments probing its ability to act as a chiroptical switch (Scheme 3). If the menthol auxiliary exhibits a stereodiscriminating influence onto the photochemical [1,11]‐sigmatropic rearrangement, (L)‐**6** would lead to preferred movement toward one direction preferring the formation of either diastereomer (L*,S*)**‐7** or (L*,R*)‐**7** over the other (Scheme 1c). In contrast, the enantiomeric homoannulene (D)‐**6** would lead to the preferential formation of either (D,*R*)‐**7** or (D*,S*)‐**7** over the other, respectively. Combined, this leads to a proposed scenario that upon the presence of calderoselectivity, the chiroptical switching of either enantiomer of enantiopure **6** should lead to a set of diastereomers, such as (L*,S*)‐**7** and (L*,R*)‐**7** emanating from (L)‐**6**. Based on this hypothesis, we conducted several analyses of the photoswitching process with both enantiomers of the menthyl homoannulene **6**.

**SCHEME 3:**
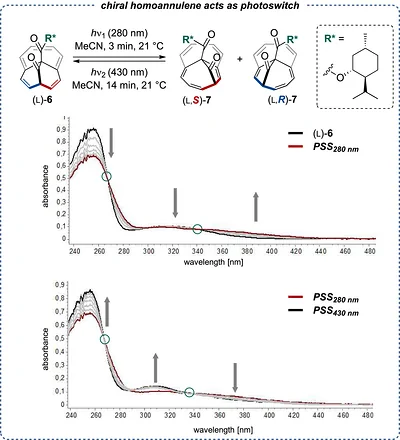
Photoswitching experiments with chiral homoannulene (L)‐**6**. UV/Vis spectra were measured during irradiation of (L)‐**6** (c = 4.36 ∙ 10^−5^ mol L^−1^, black curve) at 280 nm in MeCN. Spectra were recorded every 30 s for 3 min (grey curves) until PSS_280 nm_ (red curve) was reached. Bottom: Series of UV/Vis spectra recorded every 2 min during the photochemical back‐conversion from PSS_280 nm_ (c = 4.36 ∙ 10^−5^ mol L^−1^, red curve) by irradiation at 430 nm for 14 min in MeCN until PSS_430 nm_ was reached (black curve). For experimental details, see the Supporting Information.

Photoswitching experiments were carried out in MeCN as solvent: Upon irradiation of (L)‐**6** with an LED at 280 nm for 3 min, we observed the diminishing of the UV bands at 255 and 310 nm, accompanied by an increase of a broad absorption band at 380 nm (Scheme 3). Alternatively, the photoswitching experiments could be carried out with similar results in a larger scale with irradiation with a Hg lamp and a cutoff filter of 285 nm (see Supporting Information for details). From the latter, analysis of the resulting mixture gave a photostationary state of (L)‐**6**/(L)‐**7** = 41:59 after 60 min of irradiation, as confirmed by HPLC analysis. Back‐switching was achieved by irradiation with 430 nm (LED) as confirmed by the reappearance of the UV bands at 255 and 310 nm. Reformation of homoannulene **6** was completed after 14 min of irradiation, resembling the photoswitching behavior of the parent **4** [42]. From these experiments, we could establish that **6** indeed acts as a reversible photoswitch. The switching process was demonstrated to be repeatable up to four times with similar photostationary states, albeit every photoswitching step leads to some fatigue [57]. Furthermore, the thermal stability of the photoswitched states has been investigated. Neither state showed a significant change upon prolonged heating, supporting the notion of a photochemically allowed pericyclic reaction as key photoswitching mechanism (see the Supporting Information for details).

In order to investigate the stereochemistry of the resulting *iso*‐homoannulenes **7**, detailed chromatographic and NMR‐spectroscopic characterizations were carried out next. Chiral HPLC analysis of the photostationary states of the irradiation experiments of either enantiomer of enantiopure **6** revealed the presence of two photoproducts. (Scheme 4a). For each enantiomer of the chiral homoannulene **6**, the two products were formed in a 56:44 ratio. Importantly, the differing retention times of the photoproducts formed by irradiation of either (L)‐**6** or (D)‐**6** on the chiral stationary phase indicate that the newly formed proposed stereoisomers are non‐identical (Scheme 4 and the Supporting Information for details). These results support the above working hypothesis that the chiral menthyl auxiliary directs the [1,11]‐sigmatropic rearrangement into one preferred direction (pro‐*R* versus pro‐*S*, compare arrows in Scheme 1c). The photoproducts could be identified as the two diastereomers of (L)‐**7** ((L*,S*)‐**7** and (L*,R*)‐**7**, see further experiments below) by NMR spectroscopy (Scheme 4b). The hydrogen atom located at C‐4 of the *iso*‐homoannulene π system could be used as probe for the stereochemical analysis. At the photostationary state, two signals were found for this hydrogen atom with an integrated ratio of 56:44, regardless of which enantiomer of the photoswitch **6** was employed, with the chemical shift of the major species being centered at 4.45 ppm in both experiments. From a stereochemical perspective, this indicates that either enantiomer of the menthyl homoannulene **6** preferentially leads to the same diastereomer **7** in the switching process (for example, preferred formation of (L*,S*)‐**7** from (L)‐**6** versus preferred formation of (D*,R*)‐**7** from (D)‐**6**). This stereochemical outcome is further supported by VT‐NMR experiments (see the Supporting Information) and by ECD spectroscopy of the photoswitching experiments of enantiopure **6** before and after irradiation: In this vein, mirroring ECD spectra are found for the menthyl homoannulenes (L)‐**6** and (D)‐**6** (Scheme 4c). Both enantiomers of **6** showed a strong Cotton effect at ca. 220 nm (g_abs_,_223 nm_ = 2.6·10^−4^ for (L)‐**6** and g_abs_,_223 nm_ = –2.4·10^−4^ for (D)‐**6**). Importantly, also after photoswitching, the measured ECD spectra remained mirror images of each other, with opposite bisignate Cotton‐effect patterns, with the mixture derived from (L)‐**6** showing a negative first Cotton effect at ca. 275 nm and a positive second Cotton effect at ca. 220 nm, whereas the mixture derived from (D)‐**6** displayed the inverse pattern (g_abs_,_217 nm_ = 2.1·10^−4^ from (L)‐**6** and g_abs_,_217 nm_ = –2.0·10^−4^ from (D)‐**6**). These experiments complete the stereochemical picture of the photoswitching process: The combination of NMR and ECD spectroscopy underlines that the major isomers formed in the photoswitching are the same diastereomer (as indicated by the same major peak in the ^1^H NMR) and opposite enantiomers as shown by the ECD spectra. Overall, the experimental results shown in Schemes 3 and 4 combined corroborate the hypothesis that chiral homoannulene **6** acts as structurally novel chiroptical switch.

**SCHEME 4:**
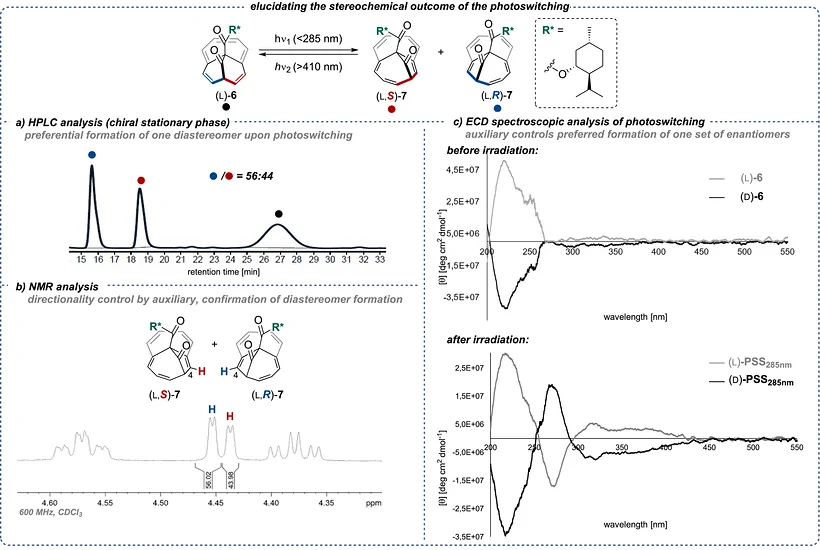
Stereochemical analysis of the photoswitching experiments of **6** and establishment of **6** as chiroptical switch. For (a and b), analysis of only one enantiomer, (L)‐**6**, is depicted here. For the analogous analysis of the opposite enantiomer, (D)‐**6**, see the Supporting Information. (a) HPLC chromatogram after irradiation of (L)‐**6** (c_(L)‐_ **_6_** = 0.0412 mol L^−1^) after irradiation with 285 nm for 1 h at 0 °C, PSS_285 nm, (L)‐_ **_6_**_/(L)‐_ **_7_** =  41:59; Chiralpak AD‐H, n‐heptane/iPrOH = 90:10, flow rate: 0.4 mL min^−1^, (b) ^1^H NMR spectrum of the irradiated mixture of (L)‐**6**, (L,S)**‐7,** and (L,R)‐**7** after irradiation at 285 nm for 1 h at 0° C. (c) Top: Detected CD spectra of (L)‐**6** and (D)‐**6** in MeCN. g_abs_,_223 nm,(L)‐_ **_6_** = 2.6·10^−4^, g_abs_,_223 nm,(D)‐_ **_6_** = –2.4·10^−4^. Bottom: CD spectra of photoswitched mixture originating from (L)‐**6** or (D)‐**6**, respectively in MeCN. Samples were irradiated at 285 nm for 1 h at 0 °C. g_abs_,_217 nm,(L)‐_ **_6_**_, irr._ = 2.1·10^−4^, g_abs_,_217 nm,(D)‐_ **_6_**_, irr._ = –2.0·10^−4^. For experimental details, see the Supporting Information.

These experiments on the photostationary state indicate that the stereochemistry of the menthyl auxiliary dictates the preferential directionality of the movement on top of the homoannulene π system in the photochemical [1,11]‐sigmatropic rearrangement. To facilitate the unambiguous determination of their absolute configurations, the diastereomeric photoproducts were separated by means of chiral HPLC. Before injection to the HPLC system, the sample solution (2.5 mg mL^−1^ of homoannulene (L)‐**6** in the mobile phase) was irradiated with 254 nm light for up to 140 min to reach a photostationary equilibrium. The best conditions for optical resolution of the diastereomers were found for a Chiralpak IG column (*n*Hep/*i*PrOH 95:5) [58]. Yet, due to the structural similarity, the difference in retention times of the photoproducts (L*,S*)‐**7** and (L*,R*)‐**7** was quite small (23.7 and 25.3 min, Scheme 5b, left), so that rather slow and dilute conditions had to be chosen for separation even on a semi‐preparative column (3.5 mL min^−1^ flow; 10 µL injections; 150 repetitions). Fractions (L*,S*)‐**7** and (L*,R*)‐**7** were collected directly into brown‐glass flasks to avoid exposure to light by any means, and the solvent was removed without heating the flasks. Finally, about 0.8 mg of each isomer could be isolated.

**SCHEME 5:**
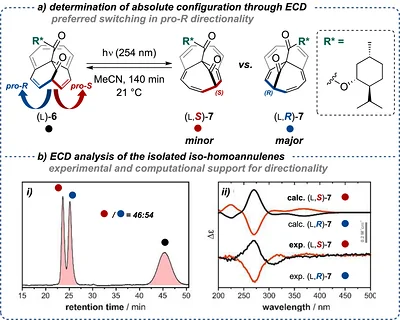
Determination of absolute configuration of *iso*‐homoannulenes **7**. (a) Chromatogram for the optical resolution of (L)‐**6** and its diastereomeric photoproducts (L,*S*)‐**7** and (L,*R*)‐**7** (injections of 10 µL of a 2.5 mg/mL solution of (L)‐**6** irradiated for 140 min with 254 nm; semi‐preparative CHIRALPAK IG, nHep/iPrOH 95:5, 3.5 mL/min flow). (b) Experimental and computed ECD spectra of (L,*S*)‐**7** and (L,*R*)‐**7** (for experimental conditions, see the Supporting Information.).

ECD measurements were carried out for the dried fractions of (L*,S*)‐**7** and (L*,R*)‐**7** in MeCN. In accordance with the enantiomeric nature of the main homoannulene chromophore, the two diastereomers show virtually mirror image spectra (Scheme 5b, right). The comparison of the experimental spectra with simulated spectra of the two isomers, which were computed at CAM‐B3LYP/6‐311++G(2d,p)/IEFPCM(ACN) level of theory, confirm that the formation of (L*,R*)‐**7** is preferred of (L*,S*)‐**7**.

Therefore, the major product in this calderoselective photochemical [1,11]‐sigmatropic rearrangement can be assigned to the one in which the acyl group moves in the pro‐*R* directionality.

The exact mechanism of stereoinduction for the photochemical calderoselective [1,11]‐sigmatropic rearrangement is unknown at present, also taking into account that the chiral auxiliary resides spatially distant from the π system involved in photoswitching. In order to elucidate qualitatively a potential distance relationship of stereoinduction with respect to the menthyl auxiliary, we probed other stereoselective reactions at various positions of the homoannulene core (Scheme 6). By virtue of these model reactions, regions of the homoannulene framework in which the chiral auxiliary exerts stronger or weaker stereoinduction could be identified. These results thus allow for a “scaling” of the stereoinduction of reactions occurring either on top of or at the π system of **6**: i) Electrophilic bromination of the 10π system of (L)‐**6** using pyridinium tribromide afforded the two possible monobrominated diastereomers (L,*S*)‐**13** and (L,*R*)‐**13** in an equimolar ratio of d.r. = 50:50. This result indicates that a functionalization distant from the chiral auxiliary leads to essentially no stereochemical bias, indicating the absence of a long‐range stereoinduction (Scheme 6, top). ii) In contrast, short‐range stereoinduction is significantly higher, as represented by the stereoselective ketone reduction of (L)‐**6** yielding the two diastereomeric alcohols (L,*S*)‐**14** and (L,*R*)‐**14** in a ratio of d.r. = 72:28. This underpins that higher stereoinduction is found for reactions in close proximity to the menthol auxiliary (Scheme 6, bottom). Consequently, the d.r. values of the medium‐range selectivity in the abovementioned calderoselective photochemical [1,11]‐sigmatropic rearrangement fit into this picture as being in between the short‐range and the long‐range stereoinduction processes. Further studies aimed at the elucidation of the exact mechanism of stereoinduction of the calderoselective chiroptical switching of homoannulenes are necessary.

**SCHEME 6:**
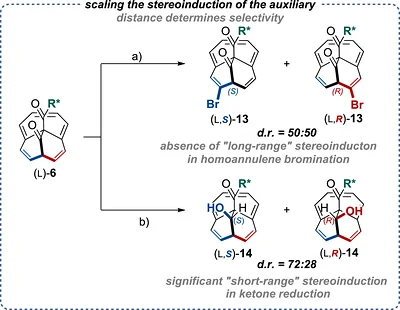
Distance‐dependence on stereoselective reactions on chiral homoannulene **6**. Reaction conditions: (a) 1.0 equiv Py∙HBr_3,_ CH_2_Cl_2_, 45 h, 62%, (b) 1.5 equiv NaBH_4_, MeOH, 21 °C, 2 h, then 6 M HCl, 21 °C, 30 min, 57%.

## 3 Conclusion

In conclusion, we herein report the first experimental realization of stereochemical control over directional migration in a sigmatropic rearrangement on top of a single cyclic π system and a proof of principle of a calderoselective process. We prepared a chiral auxiliary‐appended chiral homoannulene that can undergo reversible photochemical [1,11]‐sigmatropic rearrangement leading to a preferred movement in pro‐*R* directionality. Full stereochemical analysis of the chiroptical homoannulene switch has established the absolute configuration of the *iso*‐homoannulene switching products. This delivers key evidence for the presence of calderoselectivity. We believe that the present work bears potential for fundamental understanding of stereoselective processes of pericyclic reactions in general. At the same time, the presented homoannulene‐based chiroptical switch represents a structurally and mechanistically untapped framework for potential molecular data storage.

## Author Contributions

**Anne Krenkel**: investigation, visualization, writing – review and editing, writing – original draft, methodology, validation, and software. **Frederike Beyer**: investigation, methodology, and software. **Malte Feßner**: investigation, methodology, and software. **Trung Tran Ngoc**: investigation, methodology, and writing – review and editing. **Tobias Rüffer**: methodology, data curation, and writing – review and editing. **Dominik Hornig**: investigation, visualization, and software. **Martin Breugst**: methodology, software, data curation, resources, visualization, and supervision. **Christian Merten**: investigation, conceptualization, software, data curation, formal analysis, supervision, resources, writing – review and editing, and methodology. **Johannes F. Teichert**: conceptualization, funding acquisition, writing – original draft, writing – review and editing, visualization, formal analysis, project administration, resources, and supervision.

## Conflicts of Interest

The authors declare no conflicts of interest.

## Supporting information



The authors have cited additional references within the Supporting Information [59, 60, 61, 62, 63, 64, 65, 66, 67, 68, 69, 70, 71, 72, 73, 74, 75, 76, 77, 78, 79, 80, 81, 82, 83, 84, 85, 86].
**Supporting File**: anie75099‐sup‐0001‐SuppMat.pdf.

## Data Availability

The data that supports the findings of this study are available in the supplementary material of this article
