# Supplementary material for: Chiroptical Switching by Stereoselective Photochemical Sigmatropic Rearrangements: Experimental Realization of Calderoselectivity
Source: Angew Chem Int Ed Engl. 2026 Sep 30;65(46):e9477381. doi: 10.1002/anie.9477381 (PMC13749347; doi:10.1002/anie.9477381)
Supplement: Supplementary file 1 [file ANIE-65-e9477381-s001.pdf]

Supporting Information

Chiroptical Switching by Stereoselective Photochemical Sigmatropic Rearrangements – Experimental Realization of Calderoselectivity

Anne Krenkel,[a] Frederike Beyer,[b] Malte Feßner,[b] Trung Tran Ngoc,[a] Tobias Rüffer,[a] Dominik Hornig,[a] Martin Breugst,[a] Christian Merten,[b] Johannes F. Teichert[a]*

(a) Institut für Chemie, Technische Universität Chemnitz, 09111 Chemnitz, Germany; (b) Ruhr Universität Bochum, Fakultät für Chemie und Biochemie, Organische Chemie II, Universitätsstraße 150, 44801 Bochum, Germany.

johannes.teichert@chemie.tu-chemnitz.de

TABLE OF CONTENTS

1. EXPERIMENTAL SECTION 4

1.1 General Information 4

1.2 Solvents 6

1.3 Chemicals 6

1.4 UV/vis spectroscopy and photophysical experiments 6

1.5 Computational section 7

1.6 Experimental details 8

1.6.1 Optimized synthesis of *cis*-**9** 8

1.6.2 Synthesis of homoannulenes **6** 12

1.6.3 Experimental details........ 15

1.7 Photoswitching Experiments 31

1.7.1 Overview on photoswitching data........ 32

1.7.2 HPLC analysis of photoswitching of homoannulene methyl ester **4**........ 35

1.7.3 UV/vis absorption experiments of homoannulene menthyl ester **6** 37

1.7.4 Determination of PSS and d.r.........39

1.7.5 Thermal stability experiment 49

1.7.6 Photoswitch-cycling monitoring and photodegradation........ 51

1.7.7 ECD spectra of homoannulenes **6** and *iso*-homoannulenes **7**........ 61

1.7.8 Determination of absolute configuration of *iso*-homoannulenes **7** 65

1.8 Investigation on rotational barriers in homoannulenes 4, 6 and *iso*-homoannulenes 7 67

1.8.1 Rotational barriers in homoannulene methyl ester **4** 67

1.8.2 Rotational barriers in homoannulene menthyl ester **6** 71

1.8.3 Rotational barriers in *iso*-homoannulene menthyl esters (L)-**7**........ 80

1.9 Crystallographic data 92

2. REFERENCES........ 94

1. Experimental Section

1.1 General Information

The numbering of the respective molecules in the main manuscript and supporting information do not necessarily follow IUPAC recommendations and were done at our own discretion to guide the reader. All reactions were carried out in flame dried glassware under a nitrogen atmosphere using standard Schlenk techniques. Glassware and stir bars contaminated with transition metals were treated with aqua regia (conc. HCl/conc. HNO_3 = 3:1) prior to cleaning. For cleaning, glassware and stir bars were kept in an *i*PrOH/KOH bath overnight, rinsed with H_2O, kept in a HCl/H_2O bath overnight, rinsed again with deionized H_2O and dried at 120 °C. Solutions and reagents were added with nitrogen-flushed disposable syringes/needles. Solvents were added using glass or disposable syringes and stainless-steel needles (stored at 120 °C).

Analytical thin layer chromatography (TLC) was performed on silica gel 60 G/UV254 polyester sheets (*Macherey-Nagel*).

- Irradiation of the TLC plate with UV light (λ = 254 and 366 nm) with UV absorption by the analytes.
- Dipping the TLC plate in a solution of $12MoO_3 \cdot H_3PO_4 \cdot nH_2O$ (25 g), $Ce(SO_4)_2$ (10 g) and conc. H_2SO_4 (60 mL) in H_2O (940 mL) followed by heating with a heat gun.[1]
- Dipping the TLC plate in a solution of $KMnO_4$ (4.0 g), K_2CO_3 (20 g), and KOH (0.30 g) in H_2O (200 mL) followed by heating with a heat gun.

Flash column chromatography was performed on silica gel Davisil LC60A (40–63 µm, pore size 60 Å, *Grace*) using the indicated solvents. The required amounts of silica gel, column diameter, and fraction size were based on elaborated parameters known in the literature[2] and were adjusted if necessary.

NMR spectra were recorded on Avance III 600 (Bruker) at the Institute of Chemistry at *Technische Universität Chemnitz*. Chemical shifts (δ) are reported in parts per million (ppm) and are referenced to the residual solvent resonance as the internal standard according to the standard literature.[3,4] Data are reported as follows: chemical shift, multiplicity (br s = broad singlet, s = singlet, d = doublet, t = triplet, q = quartet, m = multiplet, m_c = centrosymmetric multiplet), coupling constants (Hz), integration and – if possible – atom assignment. The assignment refers to the atom number shown in the corresponding molecule Figure S and was achieved *via* analysis of 2D NMR spectra (COSY, HMQC, HSQC, HMBC, NOESY). If a distinct assignment was not possible, atoms were marked with "*" and are interchangeable.

Diastereotopic hydrogen atoms were given the subscripts "a" and "b", where "a" represents the high field shifted hydrogen atom and "b" represents the hydrogen atom shifted to the low field.

Melting points (Mp.) were determined using the Melting Point System MP70 (*Mettler Toledo*).

Infrared (IR) spectra were recorded on a Cary 630 FT-IR spectrometer equipped with an ATR unit (*Agilent Technologies).*

Optical rotation measurements were performed at the Institute of Chemistry at the *Technische Universität Chemnitz* by *Peter Petzold* using the automatic digital polarimeter P300RS (*Krüss*) or by using the digital polarimeter UniPol (*SCHMIDT+HAENSCH*) which was kindly provided by Univ-Prof. Dr. Robert Kretschmer (professorship of Inorganic Chemistry, *Technische Universität Chemnitz*).

High resolution mass spectra were obtained from the Analytical Faculty at the Institute for Chemistry at *Technische Universität Chemnitz* ESI and APCI mass spectrometric studies were performed on a timsTOF MS system (*Bruker Daltonik*).

Analytical gas chromatography (GC) of reaction mixtures was performed using a gas chromatograph *Agilent* 8890 GC System. The instrument was equipped with an *Agilent* J&W HP-5 GC column (length: 30 m, inner diameter: 0.32 mm, film thickness of the stationary phase: 0.25 µm). The following temperature program was used for the analysis: carrier gas N_2, detector temperature 320 ˚C, flow rate 6.0 mL min^{-1}, temperature program: 40 °C start temperature, 20 °C/min heating rate to 250° C and remain for 10 min at 250 °C. The data was recorded with the program OpenLab CDS (*Agilent softwares*).

Analytical gas chromatography coupled with mass spectrometry (GC-MS) of reaction mixtures was performed using a 5975C system (*Agilent Technologies*), which was equipped with an EI ionization source. The following temperature program was used for analysis: carrier gas He; flow rate 0.8 mL/min; temperature program: 40 °C starting temperature, 20 °C/min heating rate to final temperature 250 °C for 10 min. Data were recorded using the MSD ChemStation E.02.02.1432 program (*Agilent softwares*).

Analytical high performance liquid chromatography (HPLC) of reaction mixtures was performed at the 1260 Infinity II system (*Agilent Technologies*) using the chiral OJ-H column (*Chiralcel* from *Daicel Corporation,* 4.6 mm x 250 mm) or AD-H column *(Chiralcel* from *Daicel Corporation,* 4.6 mm x 250 mm) at 40 °C. The method used is mentioned in the respective experiment. The data were recorded using the OpenLab CDS software (*Agilent softwares*).

Reactions under microwave irradiation were carried out in microwave reaction vessels (*Biotage*) and were performed in an Initiator+ (*Biotage*). The reaction parameters that were used are written down in the respective substrates.

1.2 Solvents

THF and Et_2O were dried over sodium/benzophenone and distilled under N_2 atmosphere prior to use. Et_3N, CH_2Cl_2 and MeOH were dried over CaH_2 and distilled under N_2 atmosphere prior to use. Solvents (technical grade) for extraction/chromatography (*n*-pentane, cyclohexane, CH_2Cl_2, Et_2O, EtOAc) were distilled under reduced pressure prior to use.

1.3 Chemicals

All reagents were purchased from established commercial suppliers (*Sigma-Aldrich, AlfaAesar, TCI, Acros, Strem, Merck, ABCR, Fluka, Fisher Scientific, BLD Pharm*) and used without further purification unless otherwise stated. 2,3-Dichloro-5,6-dicyanobenzoquinone (DDQ) which was purchased by *Thermo Fisher Scientific Inc.* or *TCI Deutschland GmbH* was crystallized from $CHCl_3$ prior use. Pyridinium tribromide, purchased by *TCI Deutschland GmbH*, was washed with $CHCl_3$ prior use.

1.4 UV/vis spectroscopy and photophysical experiments

Ultraviolet–visible absorbance spectroscopy were performed on AvaSpec-ULS2048CL-EVO-RS StarLine spectrometer (*Avantes BV*) with the AvaLight-DHS halogen and deuterium light source (*Avantes BV*) in 10 × 10 mm quartz cuvettes with 4 mL volume. Slit size for the spectrometer was 25 nm, optic fibers diameter was 400 nm.

Data was analyzed using Spectragryph v1.2.16.1. (*Dr. Friedrich Menges Software-Entwicklung*).

Large scale irradiation experiments were performed using a high-pressure mercury lamp (TQ 150, *Heraeus*) conducting the light through water-cooled quartz lenses combined with a 285 nm cutoff filter (*Schott*) and a 410 nm LED panel (*Lumintronix*) equipped with a 12 V fan cooler (*Cooler Master*). For irradiation samples in small scale, samples were irradiated using mounted LEDs as light source of various wavelengths (*Thorlabs*) together with the T-Cube™ LED driver LEDD1B power supply (*Thorlabs*). The LEDs were used with 0.2 A if not stated otherwise.

1.5 Computational section

For the calculation of the rotational barriers of homoannulene menthyl ester (L)-**6**, methyl ester **4** and *iso*-homoannulene menthyl esters *(*L*,S)*-**7** and *(*L*,R)*-**7**, a conformational analysis using the Conformer Rotamer Ensemble Sampling Tool (CREST) was carried out.[5] The default parameter with the exception of an enlarged energy window of 83.7 kJ mol^{-1} (20 kcal mol^{-1}) were used in combination with the GFN-FF method[6] and a generalized Born solvation model for $CHCl_3$.

A clustering approach was used to limit the number of conformers to 20. All structures were subsequently optimized with the range-separated hybrid density functional ω-B97X-D[7] and the 6-31+G(d,p)[8] basis set. Solvation by $CHCl_3$ was accounted for by the IEFPCM continuum model.[9] A superfine grid was used for the numerical integration of the density. Vibrational analysis verified that each structure was a minimum. Potential energy surface scans were conducted on the lowest-energy structures using the GGA functional TPSS,[10] Grimme's D3 dispersion correction with Becke-Johnson damping,[11] the def2-SVP basis set,[12] and the IEFPCM model for $CHCl_3$. The dihedral angle was varied from –180° to +180° in 10° steps. All DFT calculations were performed with Gaussian 16.[13]

1.6 Experimental details

1.6.1 Optimized synthesis of *cis*-9

In order to synthesize homoannulenes **6,** *cis*-acid *cis*-**9** is employed as key intermediate. Therefore, the synthesis of *cis*-acid *cis*-**9** (Scheme S1) is discussed in the following sections. Please note that this is an optimized procedure of the previously reported synthetic route.[14,15]

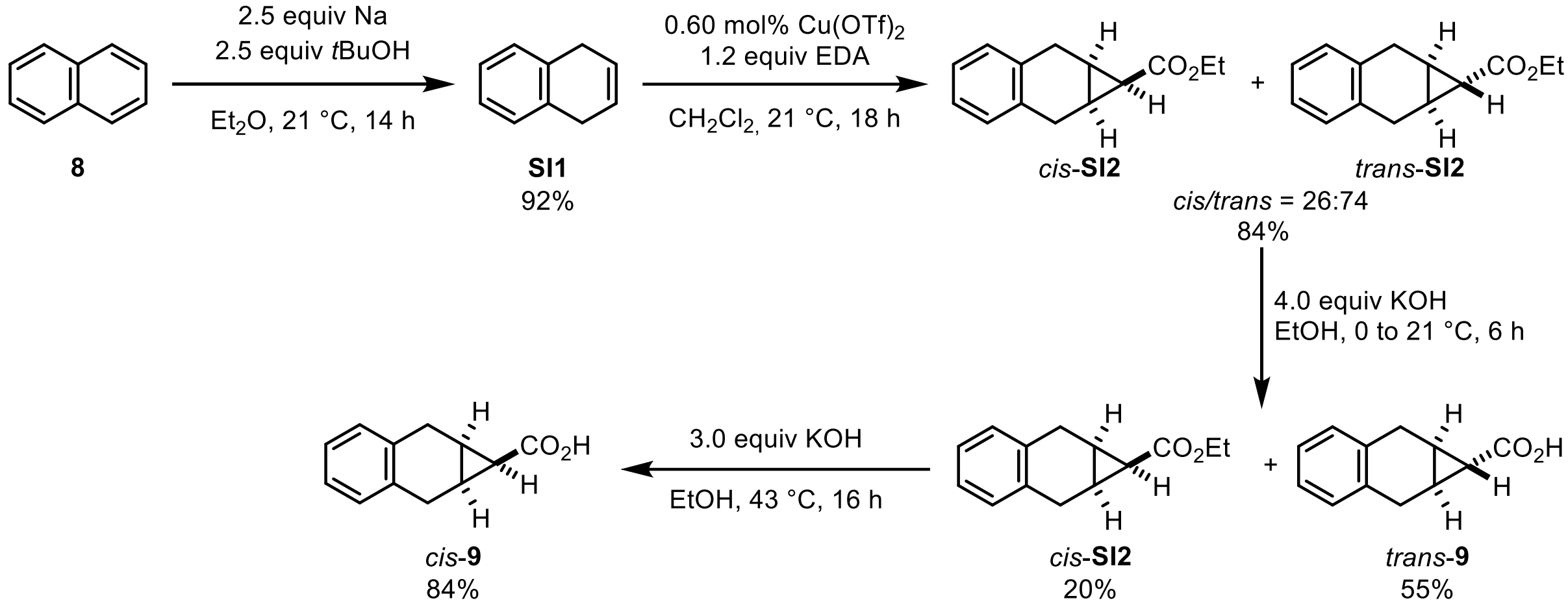

Scheme S1: Synthesis of *cis*-**9** from naphthalene (**8**).

1.6.1.1 1,4-Dihydronapthalene (SI1)

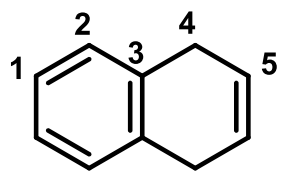

SI1
$C_{10}H_{10}$
M = 130.08 g mol^{-1}

According to a literature procedure,[15,16] a Schlenk flask was charged with naphthalene (**8**, 25.6 g, 200 mmol, 1.00 equiv) in dry Et_2O (400 mL). Sodium (11.5 g, 500 mmol, 2.50 equiv) was added portionwise to the reaction mixture. The mixture was stirred at room temperature for 15 min, then t*ert*-butanol (47.5 mL, 500 mmol, 2.50 equiv) was added dropwise and the reaction mixture was stirred at room temperature for 17 h. After complete conversion of **8** was detected (monitored by ^{1}H NMR), the mixture was filtered through a glass frit (pore 4) and the filter cake was washed with Et_2O (300 mL). The combined org. layers were washed with H_2O (3 × 100 mL) and brine (200 mL), then dried over $MgSO_4$. Filtration and removal of all volatiles under reduced pressure afforded 1,4-dihydronaphthalene (**SI1**, 24.2 g, 183 mmol, 92%) as a colorless oil and was used without further purification.

^{1}H NMR (600 MHz, $CDCl_3$): δ = 3.41 (s, 4H, H-4), 5.93 (s, 2H, H-5), 7.14 (m_c, 4H, H-1, H-2) ppm. The sample contains impurities of naphthalene and 1,2-dihydronaphthalene.[17,18]

^{13}C NMR: (151 MHz, $CDCl_3$): δ = 29.9 (C-4), 124.9 (C-5), 126.0 (C-1), 128.6 (C-2), 134.3 (C-3) ppm. The sample contains impurities of naphthalene and 1,2-dihydronaphthalene.

GC-MS for $C_{10}H_{10}$: calculated 130.1; found: 130.1.[17,18]

The data is in accordance with literature.[15,16,19]

1.6.1.2 Ethyl (1a*R*,7a*S*)-1a,2,7,7a-tetrahydro-1*H*-cyclopropa[b]naphthalene-1-carboxylate (SI2)

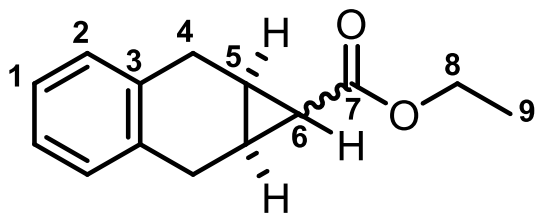

SI2
trans/cis = 74:26
$C_{14}H_{16}O_2$
M = 216.12 g mol^{-1}

A Schlenk flask was charged with $Cu(OTf)_2$ (246 mg, 1.15 mmol, 0.60 mol%) and 1,4-dihydronaphthalene (**SI1**) (25.0 g, 192 mmol, 1.00 equiv). Ethyl 2-diazoacetate (0.88 M in $CHCl_3$, 262 mL, 230 mmol, 1.20 equiv) was slowly added to the reaction mixture at room temperature for 18 h. After complete conversion of dihydronaphthalene **SI1** was detected (monitored by TLC), the reaction mixture was filtered over a plug of SiO_2 (3 × 5 cm, eluent: CH_2Cl_2, 400 mL). All volatiles were removed under reduced pressure and the crude product was purified by vacuum distillation by removing impurities ($3·10^{-3}$ mbar, 100 °C head temperature). The product **SI2** (35.0 g, 197 mmol, 84%) was obtained as a red colored oil.

The ratio of *trans*/*cis* = 74:26 was determined by ^{1}H NMR spectroscopy as determined by the two isolated resonances of H-8 at 4.09 ppm and 3.76 ppm. Wherever possible, the distinct signals of each rotamer were assigned and marked with $^{\&}$ (*cis*-**SI2**) and § (*trans*-**SI2**), respectively.

^{1}H NMR (600 MHz, $CDCl_3$): δ = 1.07 (t, $^3J_{9,8}$ = 7.1 Hz, 3H, H-9$^{\&}$), 1.22 (t, $^3J_{9,8}$ = 7.1 Hz, 3H, H-9§), 1.47 (t, $^3J_{6,5}$ = 3.9 Hz, 1H, H-6§), 1.69–1.73 (m, 3H, H-5$^{\&}$, H-6$^{\&}$), 1.96 (m_c, 2H, H-5§), 3.02–3.16 (m, 4H, H-4), 3.75 (q, $^3J_{8,9}$ = 7.1 Hz, 2H, H-8$^{\&}$), 4.09 (q, $^3J_{8,9}$ = 7.1 Hz, 2H, H-8§), 7.00–7.14 (m, 4H, H-1, H-2).

^{13}C NMR (151 MHz, $CDCl_3$): δ = 14.2 (C-9$^{\&}$), 14.4 (C-9§), 17.0 (C-5$^{\&}$), 18.9 (C-6§), 21.1 (C-6$^{\&}$), 22.3 (C-5§), 25.1 (C-4$^{\&}$), 28.4 (C-4§), 60.1 (C-8$^{\&}$), 60.5 (C-8§), 125.9 (C-1$^{\&}$), 126.6 (C-1§), 128.6 (C-2$^{\&}$), 129.1 (C-2§), 133.7 (C-3§), 135.8 (C-3$^{\&}$), 171.1 (C-7$^{\&}$), 174.5 (C-7§).

HRMS (ESI) for $C_{14}H_{17}O_2^+$ [$(M+H)^+$]: calculated: 217.1223, found: 217.1216.

The data is in accordance with literature.[14]

1.6.1.3 (*1*r,1a*R*,7a*S*)-1a,2,7,7a-Tetrahydro-1*H*-cyclopropa[b]naphthalene-1-carboxylic acid (*trans*-9), & Ethyl (1s,1a*R*,7a*S*)-1a,2,7,7a-tetrahydro-1*H*-cyclopropa[b]naphthalene-1-carboxylate (*cis*-SI2)

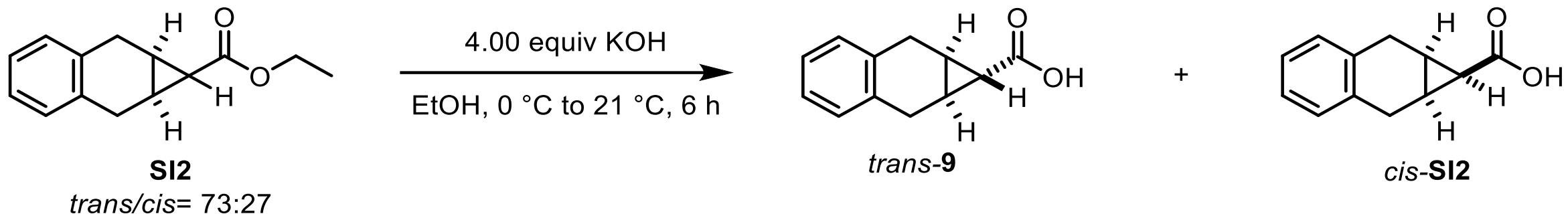

According to a literature procedure,[14] a round-bottom flask was charged with ester **SI2** (*trans/cis* = 74:26, 41.6 g, 190 mmol, 1.00 equiv) in EtOH (305 mL). KOH (42.7 g, 761 mmol, 4.00 equiv) was added in two portions at 0 °C and the reaction mixture was stirred for 1 h at 0 °C, then allowed to warm to room temperature and stirred for additional 5 h. After complete conversion of *trans*-ester *trans*-**SI2** was detected (monitored by ^{1}H NMR spectroscopy), H_2O (500 mL) was added, and the layers were separated.

1.6.1.4 *trans*-Carboxylic acid, *trans*-9

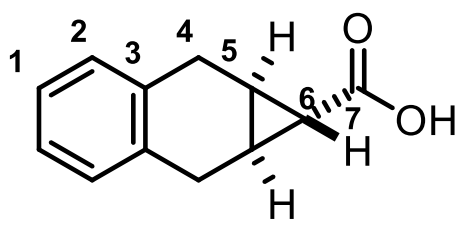

trans-**9**
$C_{12}H_{12}O_2$
M = 188.08 g mol^{-1}

According to a literature procedure,[14] the aq. layer was washed with Et_2O (3 × 300 mL). Conc. aq. HCl solution (32%) was added until pH ≤ 1 was set and the aq. layer was extracted with Et_2O (3 × 300 mL). The combined org. layers were washed with brine (200 mL) and dried over $MgSO_4$. After filtration and removal of all volatiles under reduced pressure, the afforded crude product was purified by recrystallization from cyclohexane and EtOAc to afford *trans*-carboxylic acid *trans*-**9** (22.7 g, 110 mmol, 55%) as a colorless solid.

^{1}H NMR (600 MHz, $CDCl_3$): δ = 1.48 (t, $^3J_{6,5}$ = 3.8 Hz, 1H, H-6), 2.02–2.04 (m, 2H, H-5), 3.04–3.16 (m, 4H, H-4), 7.00 (m_c, 2H, H-2), 7.13 (m_c, 2H, H-1), 11.40 (br s, 1H, O*H*) ppm.

^{13}C NMR (151 MHz, $CDCl_3$): δ = 18.8 (C-6), 23.4 (C-5), 28.3 (C-4), 126.7 (C-1), 129.1 (C-2), 133.4 (C-3), 180.3 (C-7) ppm.

HRMS (ESI) for $C_{12}H_{12}O_2Na^+$ [$(M+Na)^+$]: calculated: 211.0730; found: 211.0735.

The data is in accordance with literature.[14]

1.6.1.5 *cis*-Carboxylic ester *cis*-SI2

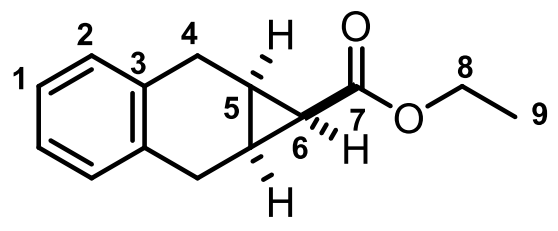

cis-**SI2**

$C_{14}H_{16}O_2$

M = 216.12 g mol^{-1}

According to a literature procedure,[14] the aq. layer was extracted with Et_2O (3 × 200 mL). The combined org. layers were washed with brine (200 mL) and dried over $MgSO_4$. After filtration and removal of all volatiles under reduced pressure, the afforded product *cis*-**SI2** (8.1 g, 42.6 mmol, 20%) was used without further purification.

^{1}H NMR (600 MHz, $CDCl_3$): δ = 1.07 (t, $^3J_{9,8}$ = 7.1 Hz, 3H, H-9), 1.70–1.73 (m, 3H, H-5, H-6), 3.03–3.13 (m, 4H, H-4), 3.75 (q, $^3J_{8,9}$ = 7.1 Hz, 2H, H-8), 7.03–7.08 (m, 4H, H-1, H-2) ppm.

^{13}C NMR (151 MHz, $CDCl_3$): δ =14.2 (C-9), 17.0 (C-5), 21.1 (C-6), 25.1 (C-4), 60.1 (C-8), 125.9 (C-1), 128.6 (C-2), 135.8 (C-3), 171.1 (C-7) ppm.

HRMS (ESI) for $C_{14}H_{17}O_2^+$ [(M+H)$^+$]: calculated: 217.1223; found: 217.1222.

The data is in accordance with literature.[14]

1.6.1.6 (1s,1a*R*,7a*S*)-1a,2,7,7a-Tetrahydro-1*H*-cyclopropa[b]naphthalene-1-carboxylic acid (*cis*-9)

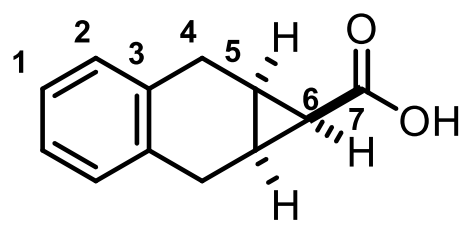

cis-**9**

$C_{12}H_{12}O_2$

M = 188.08 g mol^{-1}

According to a literature procedure,[14] a round-bottom flask was charged with *cis*-**SI2** (2.05 g, 9.39 mmol, 1.00 equiv) in EtOH (9 mL). After the addition of KOH (1.58 g, 28.2 mmol, 3.00 equiv), the reaction mixture was stirred at 43 °C for 16 h. After complete conversion of ester *cis*-**SI2** was detected (monitored by ^{1}H NMR spectroscopy), H_2O (50 mL) was added, and the aq. layer was washed with Et_2O (3 × 60 mL). Conc. aq. HCl solution (32%) was added until pH ≤1 was reached. The aq. layer was extracted with Et_2O (3 × 60 mL) and the combined org. layers were washed with brine (75 mL) and dried over $MgSO_4$. After filtration and removal of all volatiles under reduced pressure the crude product was purified by recrystallization from cyclohexane and EtOAc to afford *cis*-carboxylic acid *cis*-**9** (1.50 g, 7.89 mmol, 84%) as a colorless solid.

^{1}H NMR (600 MHz, $CDCl_3$): δ = 1.75–1.79 (m, 1H, H-6), 1.80–1.84 (m, 2H, H-5), 2.92–3.07 (m, 4H, H-4), 7.09 (s, 4H, H-1, H-2) ppm.

^{13}C NMR (151 MHz, $CDCl_3$): δ = 19.2 (C-5), 20.9 (C-6), 24.2 (C-4), 125.9 (C-2), 128.4 (C-1), 136.5 (C-3), 176.5 (C-7) ppm.

HRMS (ESI) for $C_{12}H_{12}O_2Na^+$ [(M+Na)$^+$]: calculated: 211.0730; found: 211.0733.

The data is in accordance with the literature.[14]

1.6.2 Synthesis of homoannulenes 6

The synthesis of homoannulenes **6** was commenced by following the synthetic route as displayed in Scheme S2. This synthesis is based on a literature-known procedure[14,15] and adapted to synthesizing L- and D-menthyl homoannulenes (L)-**6** and (D)-**6**. In the following section the synthetic details will be discussed.

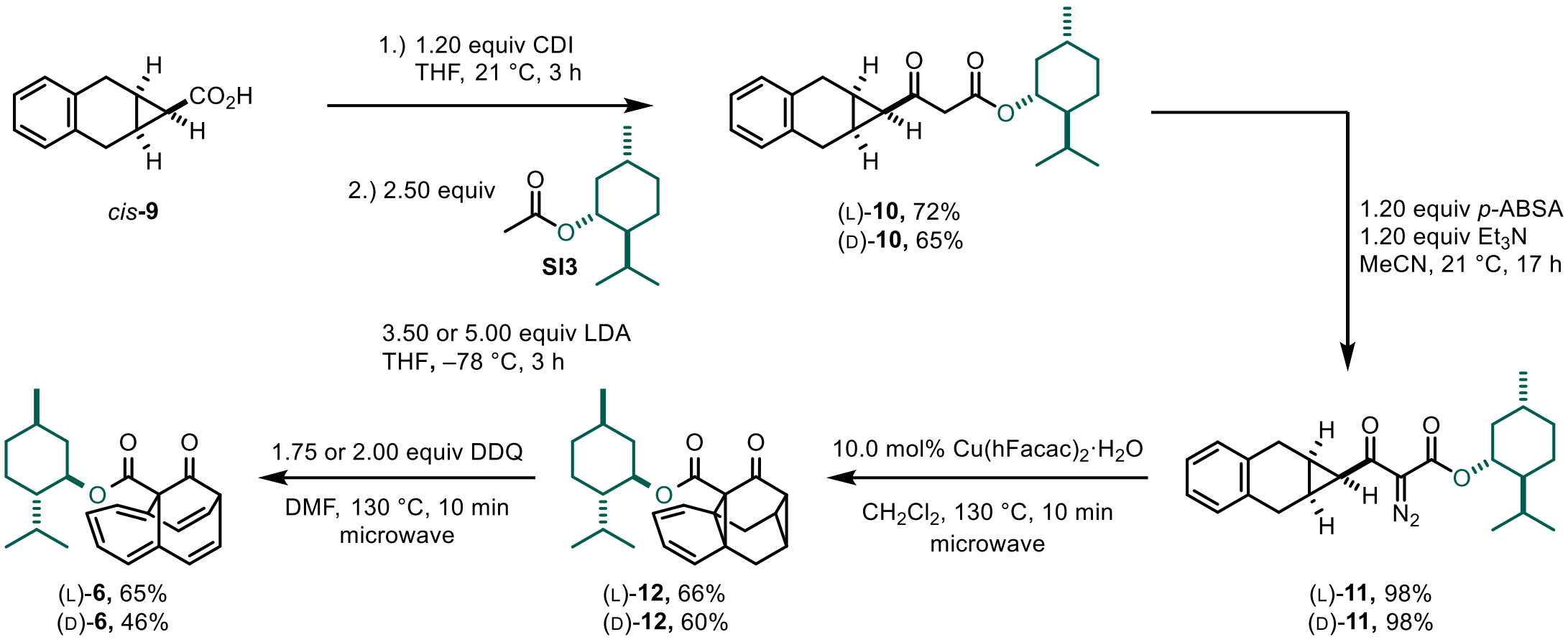

Scheme S2 Synthesis of homoannulenes **6** from *cis*-acid *cis*-**9.**

1.6.2.1 General procedures

1.6.2.1.1 Synthesis of acetoacetates 10 (GP1)

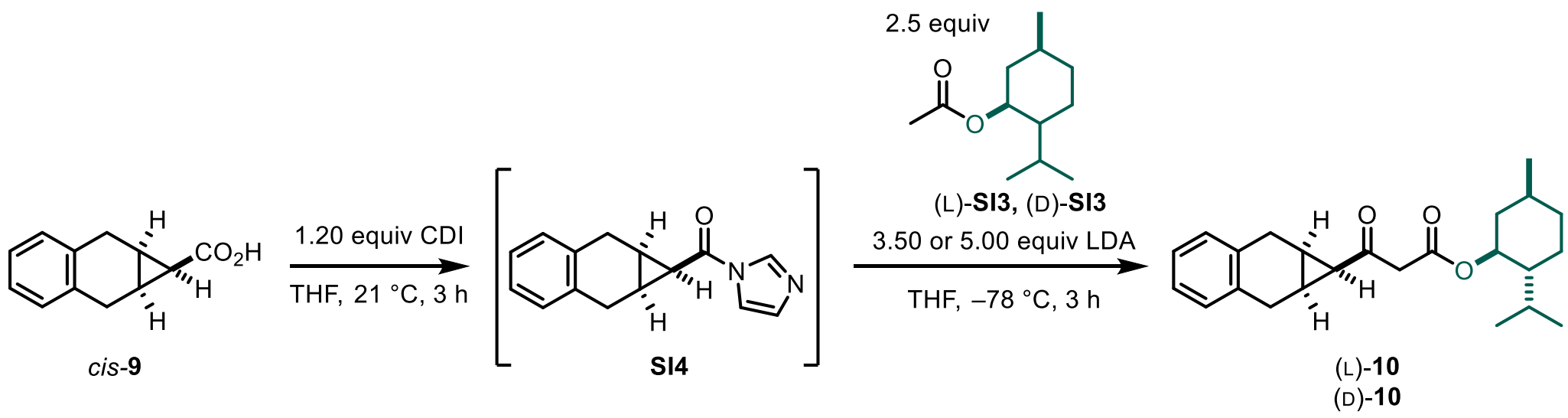

According to a modified literature procedure,[15] a Schlenk flask is charged with *cis*-**9** (1.00 equiv) in THF (4.0 mL · mmol^{-1}). After the addition of *N,N'*-carbonyldiimidazole (1.20 equiv), the mixture is stirred for 3 h at room temperature. A separate Schlenk flask is charged with di*iso*propylamine (3.50 equiv for (D)-**SI3** or 5.00 equiv for (L)-**SI3**) in THF (1.5 mL · mmol^{-1}). The solution is cooled to –78 °C and *n*BuLi (3.50 equiv for (D)-**SI3** or 5.00 equiv for (L)-**SI3**) is added dropwise. The reaction mixture is stirred at –78 °C for 10 min, warmed to 0 °C and stirred for 30 min, and again cooled to –78 °C. A solution of the respective acetate **SI3** (2.50 equiv) in THF (1.5 mL · mmol^{-1}) is added dropwise to the LDA solution. The reaction mixture is stirred at –78 °C for 1 h. The solution of the activated carboxylic acid is transferred dropwise to the enolate solution at –78 °C and the mixture is stirred for 3 h at –78 °C. After complete conversion of *cis*-**9** was detected (monitored by TLC and ^{1}H NMR

spectroscopy) the reaction is quenched by addition of 3 M HCl. The layers are separated, and the aq. layer is extracted with Et_2O (3 × 100 mL · mmol^{-1}). The combined org. layers are washed with sat. aq. $NaHCO_3$ (2 × 50 mL) and brine (1 × 75 mL) and are dried over $MgSO_4$. After filtration and removal of all volatiles under reduced pressure the crude product **10** is purified by flash column chromatography on silica gel.

1.6.2.1.2 Diazo transfer reaction to diazoacetoactetae 11 (GP2)

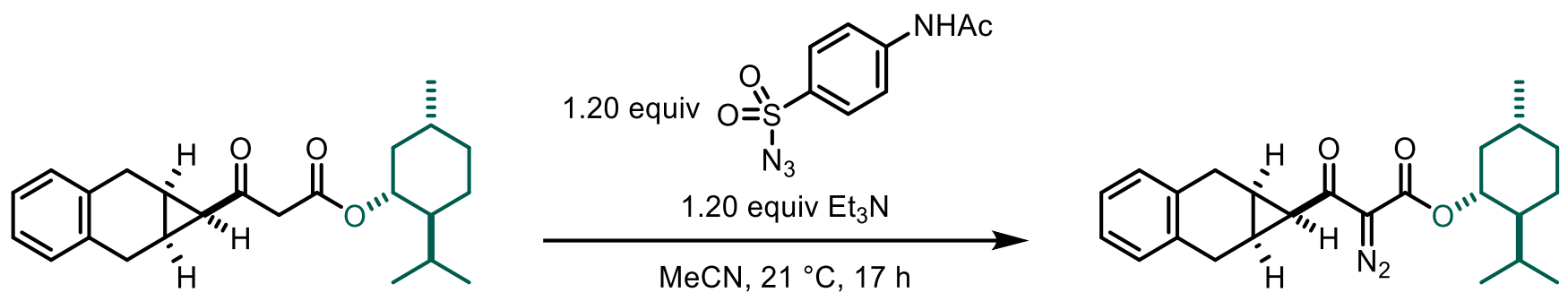

(L)-**10**
(D)-**10**

(L)-**11**
(D)-**11**

In deference to a literature,[15] in a Schlenk flask 4-acetamidobenzenesulfonyl azide (1.20 equiv) is dissolved in MeCN (10 mL · mmol^{-1}). Et_3N (1.20 equiv) is added to the solution, followed by the addition of the acetoacetate **10** in MeCN (3.3 mL · mmol^{-1}). The mixture is stirred at room temperature for 17 h. After complete conversion of **10** was detected (monitored by TLC) the reaction mixture is filtered through a glass frit (pore 4) and washed with MeCN (3.3 mL · mmol^{-1}). All volatiles of the filtrate are removed under reduced pressure. The crude product **11** is purified by flash column chromatography on silica gel.

1.6.2.1.3 Intramolecular *Buchner* dearomatization to triasteranone 12 (GP3)

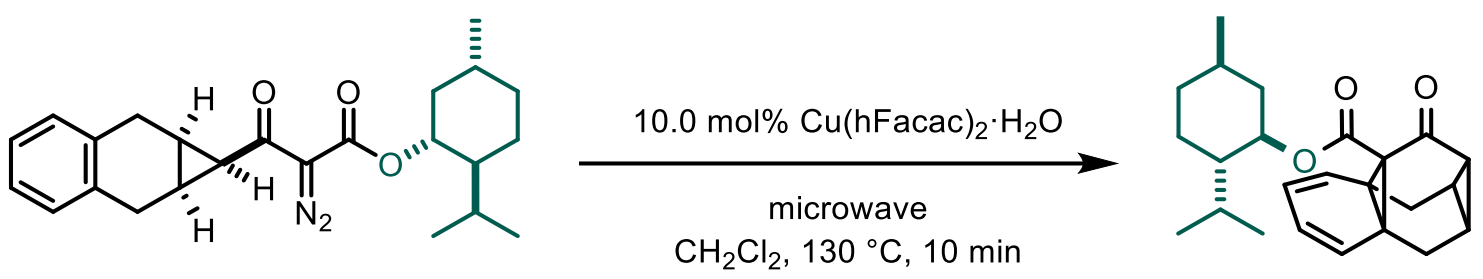

(L)-**11**
(D)-**11**

(L)-**12**
(D)-**12**

In deference to a literature,[15] a microwave vessel is charged with copper(II) hexafluoroacetylactetonate hydrate (10.0 mol%) in CH_2Cl_2 (90 mL · mmol^{-1}). A solution of diazoacetoacetate **11** (1.00 equiv) in CH_2Cl_2 (10 mL · mmol^{-1}) is added to the catalyst solution. The vessel is closed and transferred to the microwave reactor. The reaction is carried out with the following settings: 10 min, 130 °C, 10 s pre-stirring, normal absorption. If the volume of the reaction mixture exceeds the volume of the microwave vessel, a stock solution of the catalyst and diazoacetoacetate **11** is prepared, and the reaction is carried out in portions. After completion of the set reaction time, the vessel is punctured with a canula to

release all gaseous compounds. All volatiles are removed under reduced pressure. The crude product **12** is purified by flash column chromatography on silica gel.

1.6.2.1.4 Dehydrogenation with DDQ (GP4)

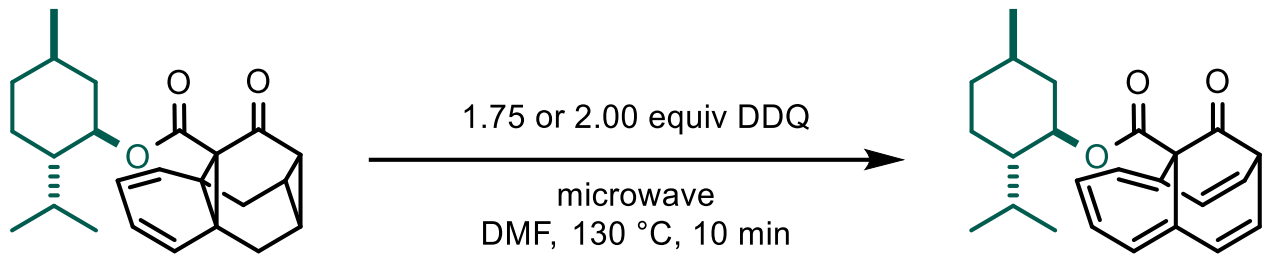

(L)-**12**
(D)-**12**

(L)-**6**
(D)-**6**

According to a modified literature procedure,[15] a microwave vessel is charged with triasteranone **12** (1.00 equiv), DDQ (2.00 equiv for (L)-**6** and 1.75 equiv for (D)-**6**) and dissolved in DMF (7.00 mL · mmol^{-1}). The vessel is closed and transferred to the microwave reactor. The reaction is carried out with the following settings: 10 min, 130 °C, 10 s pre-stirring, high absorption). After complete conversion of **12** (monitored by TLC) the reaction mixture is taken up in Et_2O (75 mL · mmol^{-1}) and the org. layer is washed with brine (5 × 50 mL · mol^{-1}), then dried over $MgSO_4$, and filtered. All volatiles are removed under reduced pressure. The crude product **6** is purified by flash column chromatography on silica gel.

1.6.3 Experimental details

1.6.3.1 (1*R*, 2*S*, 5*R*)-(5-Methyl-2-propan-2-ylcyclohexyl) acetate ((L)-SI3)

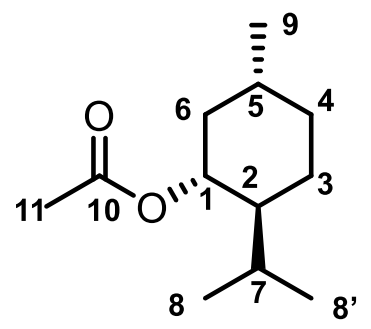

(L)-**SI3**
$C_{12}H_{22}O_2$
M = 198.16 g mol^{-1}

Based on a modified literature procedure,[20] a round-bottom flask was charged with (L)-menthol (5.30 g, 33.9 mmol, 1.00 equiv), acetic anhydride (3.85 mL, 40.7 mmol, 1.20 equiv) and four drops of conc. H_2SO_4 (98%). The mixture was heated to 100 °C for 6 h. After complete conversion (monitored by TLC and ^{1}H NMR spectroscopy), the mixture was poured into ice-cold H_2O (100 mL) and the org. layer was separated. The aq. layer was extracted with *n*-pentane (2 × 100 mL). The combined org. layers were washed with aq. $NaHCO_3$ solution (30 mL), H_2O (30 mL) and were dried over $MgSO_4$. After filtration and removal of all volatiles under reduced pressure, the crude product was purified by flash column chromatography on silica gel (cyclohexane (#1–15) → cyclohexane/EtOAc = 9:1 (#16–55), 3 × 24 cm, 20 mL, #31–43) to afford acetate (L)-**SI3** (6.03 g, 30.4 mmol, 90%) as a yellow oil.

$\mathbf{R_f}$ = 0.58 (SiO_2, cyclohexane/EtOAc = 9:1).

^{1}H NMR (600 MHz, $CDCl_3$): δ = 0.76 (d, $^3J_{9,5}$ = 7.0 Hz, 3H, H-9), 0.85 (m, 1H, H-4a) 0.89 (pseudo-q, $^2J_{8/8',7}$ = 3.5 Hz, 6H, H-8, H-8'), 0.96 (q, $^2J_{6a,6b}$ = 11.7 Hz, 1H, H-6a), 1.05 (qd, $^2J_{4a,3b}$ = 12.8 Hz, $^3J_{3a,4a}$ = 3.3 Hz, 1H, H-3a), 1.35 (m, 1H, H-2), 1.48 (m, 1H, H-5), 1.67 (m, 2H, H-3b, H-4b), 1.86 (m, 1H, H-7), 1.98 (m, 1H, H-6b), 2.03 (s, 3H, H-11), 4.67 (dt, $^2J_{1,2}$ = 10.9 Hz, $^3J_{1,6a}$ = 4.4 Hz, 1H, H-1) ppm.

^{13}C NMR (151 MHz, $CDCl_3$): δ = 16.5 (C-8'), 20.9 (C-8), 21.5 (C-11), 22.2 (C-9), 23.6 (C-3), 26.5 (C-7), 31.5 (C-5), 34.4 (C-4), 41.1 (C-6), 47.2 (C-2), 74.3 (C-1), 170.9 (C-10) ppm.

HRMS (ESI) for $C_{12}H_{22}O_2Na^+$ [(M+Na)$^+$]: calculated: 221.1512; found: 221.1514.

Optical rotation: $[\alpha]_D^{20}$ = –0.975 (c = 0.06 mol · L^{-1} in CH_2Cl_2).

The data is in accordance with the literature.[21]

1.6.3.2 (1*S*, 2*R*, 5*S*)-(5-Methyl-2-propan-2-ylcyclohexyl) acetate ((D)-SI3)

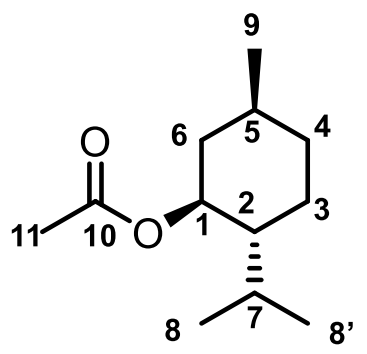

(D)-**SI3**
$C_{12}H_{22}O_2$
M = 198.16 g mol^{-1}

Based on a modified literature procedure,[20] a round-bottom flask was charged with (D)-menthol (10.7 g, 68.2 mmol, 1.00 equiv.), acetic anhydride (7.74 mL, 81.8 mmol, 1.20 equiv) and two drops of conc. H_2SO_4 (98%). The mixture was heated to 100 °C for 6 h. After complete conversion (monitored by TLC), the mixture was poured into ice-cold H_2O (100 mL) and the org. layer was separated. The aq. layer was extracted with *n*-pentane (2 × 10 mL). The combined org. layers were washed with aq. $NaHCO_3$ solution (30 mL), H_2O (30 mL) and were dried over $MgSO_4$. After filtration and removal of all volatiles under reduced pressure. The product (D)-**SI3** (12.0 g, 60.6 mmol, 89%) was obtained as a colorless oil and used without further purification.

$\mathbf{R}_f$ = 0.72 (SiO_2, cyclohexane/EtOAc = 8:2).

^{1}H NMR (600 MHz, $CDCl_3$): δ = 0.76 (d, $^3J_{9,5}$ = 7.0 Hz, 3H, H-9), 0.82–0.88 (m, 1H, H-4a), 0.89 (pseudo-q, $^2J_{8/8',7}$= 3.7 Hz, 6H, H-8, H-8‘), 0,96 (q, $^2J_{6a,6b}$= 11.7 Hz, 1H, H-6a), 1.05 (m_c, 1H, H-3a), 1.35 (m_c, 1H, H-2), 1.45 (m_c, 1H, H-7), 1.64–1.70 (m, 2H, H-3b, H-4b), 1.86 (m_c, 1H, H-5), 1.98 (m_c, 1H, H-6b), 2.03 (s, 3H, H-11), 4.70 (dt, $^2J_{1,2}$ = 10.9, $^3J_{1,6a}$ = 4.4 Hz, 1H, H-1) ppm.

^{13}C NMR (151 MHz, $CDCl_3$): δ = 16.5 (C-9), 20.9 (C-8), 21.5 (C-11), 22.2 (C-8’), 23.6 (C-3), 26.5 (C-5), 31.5 (C-7), 34.4 (C-4), 41.1 (C-6), 47.2 (C-2), 74.3 (C-1), 170.9 (C-10) ppm.

IR (ATR): ṽ = 2956 (m), 2870 (w), 1737 (s), 1454 (w), 1372 (m), 1238 (s), 1182 (w), 1096 (w), 1021 (m), 984 (w), 906 (w), 842 (w) cm^{-1}.

GC-MS for $C_{10}H_{18}$ (M-CO_2H) calculated 138.1; found: 138.2.

Optical rotation: $[\alpha]_D^{20}$= +0.059 (c = 0.050 mol · L^{-1} in CH_2Cl_2).

The data is in accordance with the literature.[21]

1.6.3.3 (1*R*,2*S*,5*R*)-2-Isopropyl-5-methylcyclohexyl 3-oxo-3-((1*s*,1*aR*,7*aS*)-1a,2,7,7a-tetrahydro-1*H*-cyclopropa[b]naphthalen-1-yl)propanoate ((L)-10)

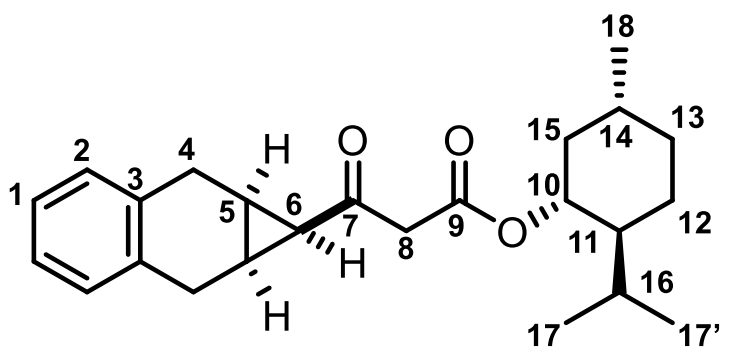

(L)-**10**
$C_{24}H_{32}O_3$
M = 368.24 g mol^{-1}

Prepared according to **GP1**, from *cis*-carboxylic acid *cis*-**9** (1.50 g, 7.89 mmol, 1.00 equiv), *N,N'*-carbonyldiimidazole (1.53 g, 9.46 mmol, 1.20 equiv), di*iso*propylamine (5.56 mL, 39.4 mmol, 5.00 equiv), *n*BuLi (2.5 M in *n*-hexane, 15.8 mL, 39.4 mmol, 5.00 equiv) and acetate (L)-**SI3** (4.25 mL, 19.7 mmol, 2.5 equiv) in THF (120 mL). The crude product was purified by flash column chromatography on silica gel (cyclohexane/EtOAc = 10:1, 3 × 25 cm, 20 mL, #20–32) to afford acetoacetate (L)-**10** (2.09 g, 5.64 mmol, 72%) as a yellow solid.

Mp.: 68–75 °C.

R*f* = 0.51 (SiO_2, cyclohexane/EtOAc = 9:1).

For the NMR analysis, please note: The sample contains traces (<5%) of a side product, derived from menthyl acetate (L)-**SI3** which could not be separated at this stage. The side product is observed in ^{1}H NMR at δ = 2.27, 3.42–3.44 ppm and in ^{13}C NMR at δ = 20.9, 22.1, 23.4, 26.3, 27.04, 34.3, 40.8, 47.0, 50.7, 75.7, 166.8, ppm.

^{1}H NMR (600 MHz, $CDCl_3$): δ = 0.75–0.78 (m, 3H, H-18), 0.87–0.92 (m, 7H, H-17, H-17', H-13a), 0.95–1.09 (m, 2H, H-12a, H-15a), 1.38 (m_c, 1H, H-11), 1.49 (m_c, 1H, H-16), 1.68 (m_c, 2H, H-12b, H-13b), 1.88 (m_c, 1H, H-14), 1.93–2.00 (m, 2H, H-5), 2.02 (m_c, 1H, H-15b), 2.12 (t, $^3J_{6,5}$= 8.2 Hz, 1H, H-6), 2.84–3.00 (m, 4H, H-4), 3.46–3.48 (m, 2H, H-8a, H-8b), 4.70–4.76 (m, 1H, H-10), 7.09 (s, 4H, H-1, H-2) ppm.

^{13}C NMR (151 MHz, $CDCl_3$): δ = 16.3 (C-18), 20.9 (C-17)*, 22.1 (C-17')*, 22.8 (2 signals, C-5), 23.4 (C-12), 23.8 (2 signals, C-4), 26.2 (C-14), 28.2 (C-6), 31.5 (C-16), 34.3 (C-13)**, 40.9 (C-15), 47.0 (C-11), 52.8 (C-8), 75.4 (C-10), 125.8 (C-1), 128.4 (C-2), 136.8 (C-3), 167.1 (C-9), 200.8 (C-7) ppm.

IR (ATR): ῦ = 2956 (m), 2930 (m), 2870 (w), 1733 (s), 1692 (s), 1454 (w), 1405 (w), 1305 (m), 1245 (s), 1178 (m), 1148 (s), 1088 (m), 1036 (m), 842 (w), 745 (m) cm^{-1}.

HRMS (ESI) for $C_{24}H_{33}O_3^+$ [$(M+H)^+$]: calculated: 369.2424; found: 369.2426.

Optical rotation: $[\alpha]_D^{20}$ = –0.340 (c = 0.020 mol · L^{-1} in CH_2Cl_2).

1.6.3.4 (1*S*,2*R*,5*S*)-2-Isopropyl-5-methylcyclohexyl 3-oxo-3-((1*r*,1a*S*,7a*R*)-1a,2,7,7a-tetrahydro-1*H*-cyclopropa[b]naphthalen-1-yl)propanoate ((D)-10)

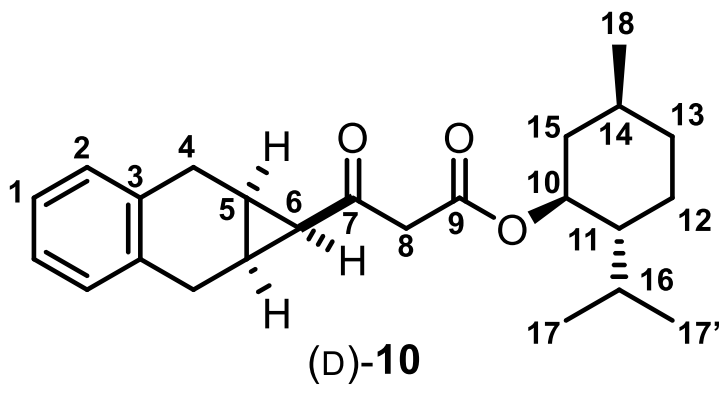

(D)-**10**
$C_{24}H_{32}O_3$
M = 368.24 g mol^{-1}

Prepared according to **GP1**, from *cis*-carboxylic acid *cis*-**9** (1.80 g, 9.46 mmol, 1.00 equiv), *N,N'*-carbonyldiimidazole (1.67 g, 10.30 mmol, 1.25 equiv), di*iso*propylamine (3.35 mL, 33.1 mmol, 3.50 equiv), *n*BuLi (2.5 M in *n*-hexane, 13.2 mL, 33.1 mmol, 3.50 equiv) and acetate (D)-**SI3** (5.10 mL, 23.7 mmol, 2.5 equiv) in THF (120 mL). The crude product was purified by flash column chromatography on silica gel (cyclohexane/EtOAc = 10:1, 3 × 20 cm, 20 mL, #14–24) to afford acetoacetate (D)-**10** (2.26 g, 6.07 mmol, 65%) as a yellow oil.

R*f* = 0.62 (SiO_2, cyclohexane/EtOAc = 8:2).

For the NMR analysis, please note: The sample contains traces (<5%) of a side product, derived from menthyl acetate (D)-**SI3** which could not be separated at this stage. The side product is observed in ^{1}H NMR at δ = 2.27, 3.42–3.44 ppm and in ^{13}C NMR at δ = 20.9, 22.1, 23.4, 26.3, 34.4, 40.9, 47.0, 50.7, 75.7, 166.9, ppm. The sample contains traces of EtOAc.

^{1}H NMR (600 MHz, $CDCl_3$): δ = 0.75–0.78 (m, 3H, H-18), 0.88–0.91 (m, 7H, H-17, H-17', H-13a), 0.97–1.07 (m, 2H, H-12a, H-15a), 1.38 (m_c, 1H, H-11), 1.49 (m_c, 1H, H-16), 1.68 (m_c, 2H, H-12b, H-13b), 1.88 (m_c, 1H, H-14), 1.95–1.99 (m, 2H, H-5), 2.02 (m_c, 1H, H-15b), 2.12 (t, $^3J_{6,5}$= 8.3 Hz, 1H, H-6), 2.84–3.00 (m, 4H, H-4), 3.46–3.48 (d, $^2J_{8,8'}$ = 2.5 Hz, 2H, H-8a, H-8b), 4.70–4.74 (m, 1H, H-10), 7.09 (s, 4H, H-1, H-2) ppm.

^{13}C NMR (151 MHz, $CDCl_3$): δ = 16.3 (C-18), 20.9$^+$ (C-17)*, 22.1$^+$ (C-17')*, 22.8$^+$ (C-5), 23.4$^+$ (C-12), 23.8$^+$ (C-4), 26.2$^+$ (C-14), 28.3 (C-6), 31.5 (C-16), 34.3$^+$ (C-13)**, 40.9$^+$ (C-15), 47.0$^+$ (C-11), 52.8 (C-8), 75.5$^+$ (C-10), 125.9 (C-1), 128.4 (C-2), 136.8 (C-3), 167.2$^+$ (C-9), 200.8 (C-7) ppm.

IR (ATR): $\tilde{\upsilon}$ = 3023 (w), 2956 (s), 2870 (m), 1737 (s), 1692 (s), 1648 (w), 1495 (w), 1454 (m), 1409 (m), 1372 (w), 1305 (m), 1249 (s), 1182 (m), 1148 (s), 1092 (m), 1036 (m), 984 (m), 842 (w), 746 (m) cm^{-1}.

HRMS (ESI) for $C_{24}H_{33}O_3^+$ [$(M+H)^+$]: calculated: 369.2424, found: 369.2423.

Optical rotation: $[\alpha]_D^{20}$ = +1.254 (c = 0.128 mol · L^{-1} in CH_2Cl_2).

1.6.3.5 (1*R*,2*S*,5*R*)-2-Isopropyl-5-methylcyclohexyl 2-diazo-3-oxo-3-((1*s*,1*aR*, 7*aS*)-1a,2,7,7a-tetrahydro-1*H*-cyclo-propa[b]naphthalen-1-yl)propanoate ((L)-11)

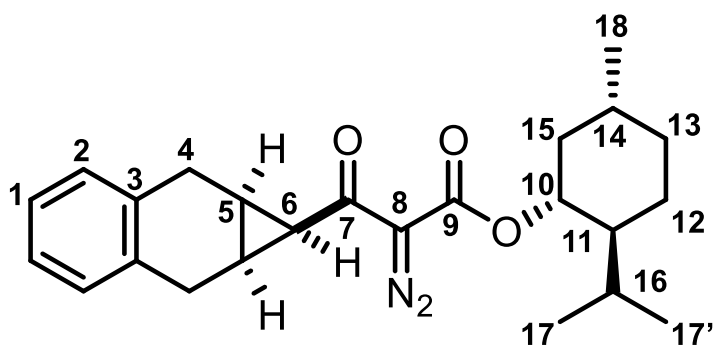

(L)-**11**
$C_{24}H_{30}N_2O_3$
M = 394.23 g mol^{-1}

Prepared according to **GP2** from acetoacetate (L)-**10** (128 mg, 0.346 mmol, 1.00 equiv), 4-acetamido-benzenesulfonylazide (123 mg, 0.512 mmol, 1.20 equiv) and Et_3N (71.3 µL, 0.512 mmol, 1.20 equiv) in MeCN (3 mL). The crude product was purified by flash column chromatography on silica gel (cyclohexane/EtOAc = 10:1, 3 × 25 cm, 20 mL, #6–10) to afford diazoacetoacetate (L)-**11** (134 mg, 0.338 mmol, 98%) as a yellow oil.

R*f* = 0.36 (SiO_2, cyclohexane/EtOAc = 10:1).

^{1}H NMR (600 MHz, $CDCl_3$): δ = 0.79–0.84 (m, 3H, H-18), 0.89–0.96 (m, 7H, H-17, H-17', H-13a), 1.08 (m_c, 2H, H-12a, H-15a), 1.39–1.46 (m, 1H, H-11), 1.54 (s, 1H, H-16), 1.72 (m_c, 2H, H-12b, H-13b), 1.84–1.90 (m, 1H, H-14), 1.99 (m_c, 2H, H-5), 2.09 (m_c, 1H, H-15b), 2.83 (t, $^3J_{6,5}$ = 8.4 Hz, 1H, H-6), 2.96–3.08 (m, 4H, H-4), 4.80–4.86 (m, 1H, H-10), 7.07 (s, 4H, H-1, H-2) ppm.

^{13}C NMR (151 MHz, $CDCl_3$): δ = 16.8 (C-18), 20.8 (C-17)*, 21.7$^+$ (C-5), 22.1$^+$ (C-17')*, 23.8 (C-12)**, 24.9$^+$ (C-4), 26.4 (C-6), 26.8$^+$ (C-14), 31.6$^+$ (C-16), 34.3$^+$ (C-13)**, 41.3 (C-15), 47.2 (C-11), 75.6 (C-10), 125.8 (2 signals, C-1), 128.5 (2 signals, C-2), 136.4 (2 signals, C-3), 161.6 (C-9), 189.8 (C-7) ppm. Carbon atom C-8 was not detected.

IR (ATR): ṽ = 2956 (m), 2930 (m), 2870 (w), 2132 (m), 1707 (s), 1640 (m), 1454 (w), 1413 (w), 1368 (w), 1297 (m), 1211 (m), 1129 (m), 1073 (m), 999 (m), 872 (w), 809 (w), 746 (m) cm^{-1}.

HRMS (ESI) for $C_{24}H_{31}N_2O_3^+$ [(M+H)$^+$]: calculated: 395.2329, found: 395.2336.

Optical rotation: $[\alpha]_D^{20}$= –0.432 (c = 0.020 mol · L^{-1} in CH_2Cl_2).

1.6.3.6 (1*S*,2*R*,5*S*)-2-Isopropyl-5-methylcyclohexyl 2-diazo-3-oxo-3-((1*r*,1*aS*, 7*aR*)-1a,2,7,7a-tetrahydro-1*H*-cyclo-propa[b]naphthalen-1-yl)propanoate ((D)-11)

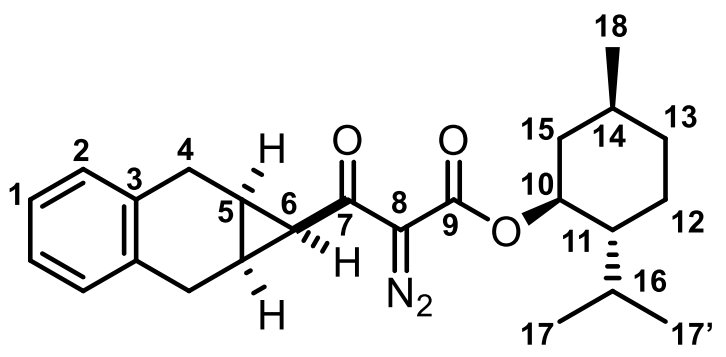

(D)-**11**
$C_{24}H_{30}N_2O_3$
M = 394.23 g mol^{-1}

Prepared according to **GP2** from acetoacetate (D)-**10** (760 mg, 2.05 mmol, 1.00 equiv), 4-acetamidobenzenesulfonylazide (591 mg, 2.46 mmol, 1.20 equiv), Et_3N (0.343 mL, 2.46 mmol, 1.20 equiv) in MeCN (7 mL). The crude product was purified by flash column chromatography on silica gel (cyclohexane/EtOAc = 10:1, 3 × 30 cm, 20 mL, #11–23) to afford diazoacetoacetate (D)-**11** (0.80 g, 2.02 mmol, 98%) as a yellow oil.

R*f* = 0.69 (cyclohexane/EtOAc = 8:2).

For the NMR analysis, please note: The sample contains traces (<5%) of a side product, derived from menthyl acetate (D)-**SI3** which could not be separated at this stage. The side product is observed in ^{1}H NMR at δ = 0.76, 0.80, 2.49 and 4.62, ppm and in ^{13}C NMR at δ = 20.9, 21.5, 22.2, 23.6, 26.5, 31.5, 34.4, 41.1, 47.1, 74.3, ppm. The sample contains traces of CH_2Cl_2.

^{1}H NMR (600 MHz, $CDCl_3$): δ = 0.79–0.84 (m, 3H, H-18), 0.87–0.89 (m, 1H, H-13a), 0.91–0.96 (m, 6H, H-17, H-17'), 1.04–1.44 (m_c, 2H, H-12a, H-15a), 1.45 (m_c, 1H, H-11), 1.50 (m_c, 1H, H-16*), 1.72 (m_c, 2H, H-12b, H-13b), 1.84–1.90 (m, 1H, H-14*), 1.99 (m_c, 2H, H-5), 2.09 (m_c, 1H, H-15b), 2.83 (t, $^{3}J_{6,5}$ = 8.4 Hz, 1H, H-6), 2.96–3.08 (m, 4H, H-4), 4.80–4.86 (td, $^{3}J_{10,15a}$ = 10.9, 4.40 Hz, 1H, H-10), 7.07 (s, 4H, H-1, H-2) ppm.

^{13}C NMR (151 MHz, $CDCl_3$): (151 MHz, $CDCl_3$): δ = 16.8 (C-18), 20.8 (C-17)*, 21.7 (2 signals, C-5), 22.1 (C-17')*, 23.8 (C-12)**, 24.9 (2 signals, C-4), 26.4 (C-6), 26.7 (C-14)***, 31.6 (C-16)***, 34.3 (C-13)**, 41.3 (C-15), 47.2 (C-11), 75.6 (C-10), 125.8 (2 signals, C-1), 128.5 (2 signals, C-2), 136.4 (2 signals, C-3), 161.6 (C-9), 189.8 (C-7) ppm. Carbon atom C-8 was not detected.

IR (ATR): ṽ = 2960 (m), 2870 (w), 2132 (s), 1707 (s), 1640 (m), 1457 (w), 1413 (w), 1297 (s), 1211 (m), 1133 (m), 1077 (m), 999 (m), 746 (m) cm^{-1}.

HRMS (ESI) for $C_{24}H_{31}N_2O_3^+$ [(M+H)$^+$]: calculated: 395.2329, found: 395.2331.

Optical rotation: $[\alpha]_D^{20}$ = +2.380 (c = 0.100 mol · L^{-1} in CH_2Cl_2).

1.6.3.7 (L)-Menthyl ester triasteranone ((L)-12)

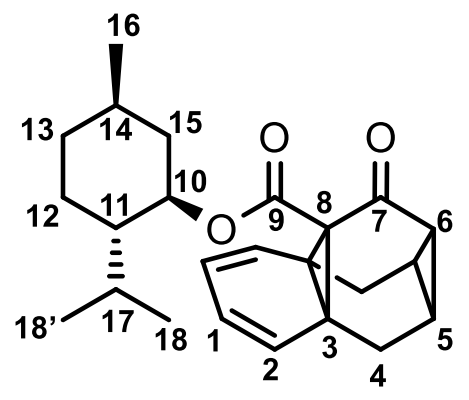

(L)-**12**
$C_{24}H_{30}O_3$
M = 366.22 g mol^{-1}

Prepared according to **GP3** from diazoacetoacetate (L)-**11** (400 mg, 1.01 mmol, 1.00 equiv) and copper(II) hexafluoro acetylacetonate hydrate (48.2 mg, 101 µmol, 0.100 equiv) in CH_2Cl_2 (200 mL). The crude product was purified by flash column chromatography on silica gel (cyclohexane/EtOAc = 9:1, 1.5 × 25 cm, 10 mL, #12–20) to afford triasteranone (L)-**12** (244 mg, 0.666 mmol, 66%) as a yellow solid.

Mp.: 163–168 °C.

$\mathbf{R}_f$ = 0.38 (SiO_2, cyclohexane/EtOAc = 7:3).

^{1}H NMR (600 MHz, $CDCl_3$): δ = 0.70 (d, $^3J_{16,14}$ = 6.7 Hz, 3H, H-16), 0.83–0.93 (m, 8H, H-18, H-18’, H-15a, H-13a), 0.98 (m_c, 1H, H-12a), 1.30 (t, $^3J_{11,10}$ = 11.5 Hz, 1H, H-11)**, 1.39 (br s, 1H, H-17)**, 1.63 (m_c, 2H, H-12b, H-13b), 1.85 (d, $^3J_{5,6}$ = 6.1 Hz, 2H, H-5), 1.92 (d, $^2J_{15b,15a}$ = 11.3 Hz, 1H, H-15b), 2.02–2.09 (m, 2H, H-14, H-6)**, 2.33 (d, $^2J_{4a,4b}$ = 14.2 Hz, 2H, H-4a)*, 2.52–2.58 (m, 2H, H-4b)*, 4.54 (m_c, 1H, H-10), 5.90–5.97 (m, 4H, H-1, H-2) ppm.

^{13}C NMR (151 MHz, $CDCl_3$): δ = 15.7 (C-16), 21.0 (C-18)*, 22.2 (C-18’)*, 22.8 (C-5), 22.9 (C-12), 23.2 (C-4), 25.2 (C-14)**, 26.6 (C-6), 31.5 (C-17)**, 34.4 (C-13), 40.6 (C-15), 40.9 (C-8), 45.8 (C-3), 47.0 (C-11)**, 75.4 (C-10), 123.0 (C-2), 128.8 (C-1), 164.8 (C-9), 203.3 (C-7) ppm.

IR (ATR): ῦ = 3038 (w), 2960 (m), 2930 (m), 2863 (w), 1726 (s), 1647 (s), 1465 (w), 1353 (m), 1282 (w), 1237 (s), 1137 (m), 1081 (m), 1032 (m), 962 (m), 924 (m), 827 (m), 731 (s) cm^{-1}.

HRMS (ESI) for $C_{24}H_{31}O_3^+$ [$(M+H)^+$]: calculated: 367.2268; found: 367.2266.

Optical rotation: $[\alpha]_D^{20}$= –0.166 (c = 0.02 mol · L^{-1} in CH_2Cl_2).

1.6.3.8 (D)-Menthyl ester triasteranone ((D)-12)

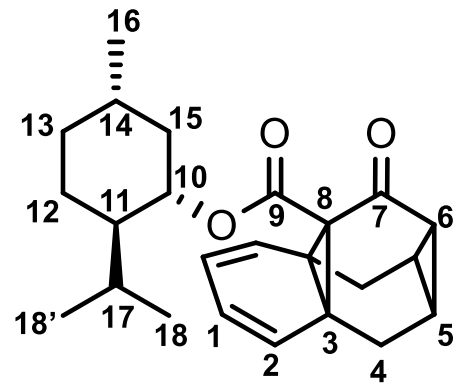

(D)-**12**
$C_{24}H_{30}O_3$
M = 366.22 g mol^{-1}

Prepared according to **GP3**, from diazoacetoacetate (D)-**11** (400 mg, 1.01 mmol, 1.00 equiv) and copper(II) hexafluoro acetylacetonate hydrate (48.2 mg, 0.101 mmol, 0.10 equiv) in CH_2Cl_2 (100 mL). The crude product was purified by flash column chromatography on silica gel (cyclohexane/EtOAc = 8:2, 1.5 × 20 cm, 10 mL, #35–50) to afford triasteranone (D)-**12** (220 mg, 0.600 mmol, 60%) as a colorless oil.

R_f = 0.19 (SiO_2, cyclohexane/EtOAc = 8:2).

^{1}H NMR (600 MHz, $CDCl_3$): δ = 0.71 (d, $^3J_{16,14}$ = 6.9 Hz, 3H, H-16), 0.79–0.82 (m, 1H, H_a-13*), 0.85–0.89 (m, 7H, H-15a, H-18, H-18‘), 0.96–1.01 (m, 1H, H-12a*), 1.30 (m_c, 1H, H-11), 1.40 (m_c, 1H, H-17), 1.61–1.63 (m, 2H, H-12b, H-13b), 1.85 (d, $^3J_{5,6}$ = 8.3 Hz, 2H, H-5), 1.91 (m_c, 1H, H-15b), 2.02–2.07 (m, 2H, H-6, H-14), 2.33 (d, $^2J_{4a,4b}$ = 14.3 Hz, 2H, H-4a), 2.55 (dd, $^2J_{4b,4a}$ = 13.8, $^3J_{4b,5}$ = 7.3 Hz, 2H, H-4b), 4.51–4.55 (m, 1H, H-10), 5.89–5.96 (m, 4H, H-1, H-2) ppm.

^{13}C NMR (151 MHz, $CDCl_3$): δ = 15.7 (C-16), 21.0 (C-18)*, 22.2 (C-18')*, 22.7 (2 signals, C-5, C-5'), 23.0 (C-12)***, 23.2 (C-4), 25.2 (C-14)**, 26.6 (C-6), 31.5 (C-17)**, 34.4 (C-13)***, 40.6 (C-15), 40.8 (C-8), 45.8 (C-3), 47.0 (C-11), 75.4 (C-10), 123.0 (2 signals, C-2), 128.8 (2 signals, C-1), 164.8 (C-9), 203.1 (C-7) ppm.

IR (ATR): ṽ = 2956 (m), 2870 (m), 1733 (s), 1670 (s), 1531 (m), 1457 (w), 1356 (m), 1282 (m), 1252 (s), 1197 (m), 1141 (s), 1081 (m), 1036 (m), 916 (w), 929 (w), 794 (w), 731 (m), 664 (w) cm^{-1}.

HRMS (ESI) for $C_{24}H_{30}O_3Na^+$ [(M+Na$^+$)] calculated: 389.2087, found: 389.2085.

Optical rotation: $[\alpha]_D^{20}$= 0.508 (c = 0.075 mol · L^{-1} in CH_2Cl_2).

1.6.3.9 (1*R*,2*S*,5*R*)-2-Isopropyl-5-methylcyclohexyl 1-oxo-1,2-dihydro-9a*H*-2,9-ethenobenzo[7]annulen-9a-carboxylate ((L)-6)

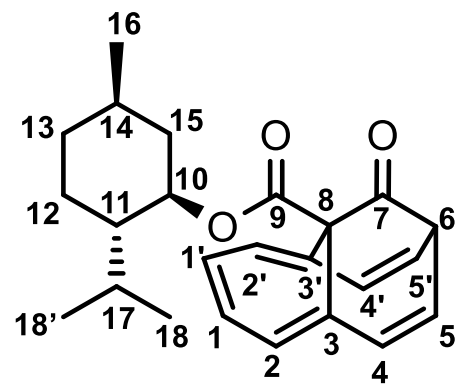

(L)-**6**
$C_{24}H_{28}O_3$
M = 364.20 g mol^{-1}

Prepared according to **GP4** from triasteranone (L)-**12** (274 mg, 0.748 mmol, 1.00 equiv) and DDQ (339 mg, 1.50 mmol, 2.00 mmol) in DMF (10 mL) using the microwave reactor. The crude product was purified by flash column chromatography on silica gel (SiO_2, cyclohexane/EtOAc = 9:1, 4 × 30 cm, 20 mL, #25–60) to afford homoannulene (L)-**6** (176 mg, 0.483 mmol, 65%) as a colorless solid.

Mp.: 94–98 °C.

R*f* = 0.34 (SiO_2, cyclohexane/EtOAc = 9:1).

For the ^1H NMR analysis, the product was isolated as a mixture of two rotamers in a ratio of 50:50, as determined by the two isolated resonances of H-10 at δ = 4.20 ppm and 4.38 ppm. The presence of rotamers was indicated by VT-NMR experiments (see Section 1.8.2). Wherever possible, the distinct signals of each rotamer were assigned and marked with [&] and [§], respectively.

^{1}H NMR (600 MHz, $CDCl_3$): δ = 0.59 (d, $^3J_{16,14}$ = 6.9 Hz, 1.5H, H-16[&]), 0.64–0.70 (m, 0.5H, H-15a[§]), 0.70 (d, $^3J_{16,14}$ = 6.9 Hz, 1.5H, H-16[§]), 0.74–0.96 (m, 8.5H, H-15a[&], H-18, H-18’, H-13a, H-12a), 1.16–1.24 (m, 1H, H-11)****, 1.33 (m_c, 1H, H-17), 1.52–1.62 (m, 2.5H, H-12b, H-13b, H-15b[§]), 1.77 (m_c, 0.5H, H-14[&])****, 1.83 (m_c, 0.5H, H-14[§]) ****, 2.06 (m_c, 0.5H, H-15b[&]), 3.79 (q, $^3J_{6,5/5'}$ = 6.9 Hz, 1H, H-6), 4.20 (td, $^3J_{10,11}$ = 11.0, $^3J_{10,15}$ = 4.1 Hz, 0.5H, H-10[&]), 4.38 (td, $^3J_{10,11}$ = 10.9, $^3J_{10,15}$ = 4.2 Hz, 0.5H, H-10[§]), 5.28 (t, $^3J_{5/5',6}$ = 7.8 Hz, 1H, H-5, H-5’)***, 5.33 (t, $^3J_{5/5',6}$ = 7.8 Hz, 1H, H-5, H-5’)***, 6.67–6.71 (m, 2H, H-4, H-4’), 6.74 (m_c, 1H, H-2)**, 6.81 (d, $^3J_{2/2',1/1'}$ = 6.8 Hz, 0.5H, H-2’)**, 6.85 (d, $^3J_{2/2',1/1'}$ = 6.8 Hz, 0.5H, H-2’)**, 6.88–6.92 (m, 0.5H, H-1), 6.92–6.97 (m, 0.5H, H-1)*, 7.02–7.07 (m, 1H, H-1’)* ppm.

^{13}C NMR (151 MHz, $CDCl_3$): δ= 15.6 (C-16[&]), 15.8 (C-16[§]), 20.9 (C-18)******, 21.1(C-18)******, 22.1 (C-18’)******, 22.2 (C-18’)******, 22.7 (C-13), 23.1 (C-13), 25.4 (2 signals, C-14) *******, 31.4 (C-17), 31.5 (C-17), 34.4 (C-12), 39.9 (C-15[&]), 40.5 (C-15[§]), 45.4 (C-6), 45.5 (C-6), 46.6 (C-11)*******, 46.8 (C-11) *******, 61.3 (C-8), 61.4 (C-8), 75.4 (C-10[§]), 75.9 (C-10[&]), 110.5 (C-5, C-5’)******, 110.6 (C-5, C-5’)******, 110.8 (C-5, C-5’)******, 111.0 (C-5, C-5’)******, 121.3 (C-3, C-3´)*****, 121.5 (C-3, C-3´)*****, 121.6 (C-3, C-3´)*****, 121.9 (C-3, C-3´)*****, 123.6 (C-2)****, 123.7 (C-2)****, 123.9 (C-2’)****, 124.2 (C-2*)****, 127.5 (C-1)***, 127.6 (C-1)***, 127.7 (C-4, C-4´)**, 127.9 (C-4, C-4´)**, 128.0 (2 signals, C-4, C-4´)**, 129.1 (C-1’)*, 129.3

(C-1')*, 165.3 (C-9), 165.4 (C-9), 202.8 (C-7), 203.2 (C-7) ppm. The sample contains an unidentified impurity at δ = 29.9 ppm.

IR (ATR): $\tilde{\upsilon}$ = 2956 (m), 2870 (w), 2363 (w), 1752 (w), 1700 (s), 1640 (s), 1457 (m), 1390 (m), 1275 (m), 1185 (w), 1155 (w), 1118 (s), 1066 (w), 1040 (w), 917 (m), 749 (w) cm^{-1}.

HRMS (ESI) for $C_{24}H_{29}O_3^+$ [$(M+H)^+$]: calculated: 365.2111; found: 365.2095.

Optical rotation: $[\alpha]_D^{21}$= –0.086 (c = 0.017 mol · L^{-1} in CH_2Cl_2).

1.6.3.10 (1*S*,2*R*,5*S*)-2-Isopropyl-5-methylcyclohexyl 1-oxo-1,2-dihydro-9a*H*-2,9-ethenobenzo[7]annulen-9a-carboxylate ((D)-6)

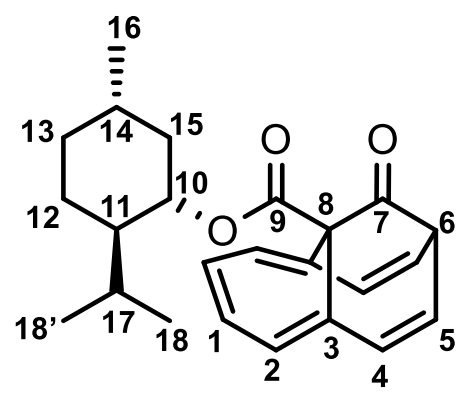

(D)-**6**
$C_{24}H_{28}O_3$
M = 364.20 g mol^{-1}

Prepared according to **GP4** from triasteranone (D)-**12** (65 mg, 0.177 mmol, 1.00 equiv) and DDQ (70.5 mg, 0.310 mmol, 1.75 equiv) in DMF (2 mL) using the microwave reactor. The crude product was purified by flash column chromatography on silica gel (cyclohexane/EtOAc = 9:1, 1.5 × 20 cm, 10 mL, #8–11) to afford homoannulene (D)-**6** (30.0 mg, 0.0823 mmol, 46%) as an orange solid.

R_f = 0.55 (SiO_2, cyclohexane/EtOAc = 8:2).

For the ^{1}H NMR analysis, the product was isolated as a mixture of two rotamers in a ratio of 50:50, as determined by the two isolated resonances of H-10 at δ = 4.20 ppm and 4.38 ppm. Wherever possible, the distinct signals of each rotamer were assigned and marked with $^{\&}$ and §, respectively. For the analysis of rotamers and rotational barriers, see compound (L)-**6**.

^{1}H NMR (600 MHz, $CDCl_3$): δ = 0.59 (d, $^3J_{16,14}$ = 6.9 Hz, 1.5H, H-16$^{\&}$), 0.64–0.70 (m, 0.5H, H-15a§), 0.70 (d, $^3J_{16,14}$ = 6.9 Hz, 1.5H, H-16§), 0.74–0.96 (m, 8.5H, H-15a$^{\&}$, H-18, H-18', H-13a, H-12a), 1.16–1.24 (m, 1H, H-11)****, 1.33 (m_c, 1H, H-17), 1.52–1.62 (m, 2.5H, H-12b, H-13b, H-15b§), 1.77 (m_c, 0.5H, H-14$^{\&}$)****, 1.83 (m_c, 0.5H, H-14§)****, 2.06 (m_c, 0.5H, H-15b$^{\&}$), 3.79 (q, $^3J_{6,5/5'}$ = 6.9 Hz, 1H, H-6), 4.20 (td, $^3J_{10,11}$ = 11.0, $^3J_{10,15}$ = 4.1 Hz, 0.5H, H-10$^{\&}$), 4.38 (td, $^3J_{10,11}$ = 10.9, $^3J_{10,15}$ = 4.2 Hz, 0.5H, H-10§), 5.28 (t, $^3J_{5/5',6}$ = 7.8 Hz, 1H, H-5, H-5')***, 5.33 (t, $^3J_{5/5',6}$ = 7.8 Hz, 1H, H-5, H-5')***, 6.67–6.71 (m, 2H, H-4, H-4'), 6.74 (m_c, 1H, H-2)**, 6.81 (d, $^3J_{2/2',1/1'}$ = 6.8 Hz, 0.5H, H-2')**, 6.85 (d, $^3J_{2/2',1/1'}$ = 6.8 Hz, 0.5H, H-2')**, 6.88–6.92 (m, 0.5H, H-1), 6.92–6.97 (m, 0.5H, H-1)*, 7.02–7.07 (m, 1H, H-1')* ppm.

^{13}C NMR (151 MHz, $CDCl_3$): δ = 15.6 (C-16$^{\&}$), 15.8 (C-16§), 20.9 (C-18)******, 21.1(C-18)******, 22.1 (C-18')******, 22.2 (C-18')******, 22.7 (C-13), 23.1 (C-13), 25.4 (2 signals, C-14) *******,

31.4 (C-17), 31.5 (C-17), 34.4 (C-12), 39.9 (C-15&), 40.5 (C-15§), 45.4 (C-6), 45.5 (C-6), 46.6 (C-11)*******, 46.8 (C-11) *******, 61.3 (C-8), 61.4 (C-8), 75.4 (C-10§), 75.9 (C-10&), 110.5 (C-5, C-4')******, 110.6 (C-4, C-4')******, 110.8 (C-5, C-5')******, 111.0 (C-5, C-5')******, 121.3 (C-3, C-3´)*****, 121.5 (C-3, C-3´)*****, 121.6 (C-3, C-3´)*****, 121.9 (C-3, C-3´)*****, 123.6 (C-2)****, 123.7 (C-2)****, 123.9 (C-2')****, 124.2 (C-2*)****, 127.5 (C-1)***, 127.6 (C-1)***, 127.7 (C-4, C-4´)**, 127.9 (C-4, C-4´)**, 128.0 (2 signals, C-5, C-5´)**, 129.1 (C-1')*, 129.3 (C-1')*, 165.3 (C-9), 165.4 (C-9), 202.8 (C-7), 203.2 (C-7) ppm.

IR (ATR): ῦ = 2952 (m), 2870 (w), 1729 (s), 1454 (w), 1282 (m), 1238 (s), 1185 (m), 1118 (w), 1066 (m), 1014 (w), 980 (m), 954 (w), 913 (m), 842 (w), 790 (w), 738 (w) cm^{-1}.

HRMS (ESI) for $C_{24}H_{28}O_3Na^+$ [(M+Na$^+$)] calculated: 387.1931, found: 387.1929.

Optical rotation: $[\alpha]_D^{20}$= +0.075 (c = 0.020 mol · L^{-1} in CH_2Cl_2).

1.6.3.11 (1*R*,2*R*,5*S*)-2-Isopropyl-5-methylcyclohexyl 10-oxo-9,10-dihydro-10a*H*-1,9-(metheno)heptalene-10a-carboxylate ((L,*S*)-7, (L,*R*)-7)

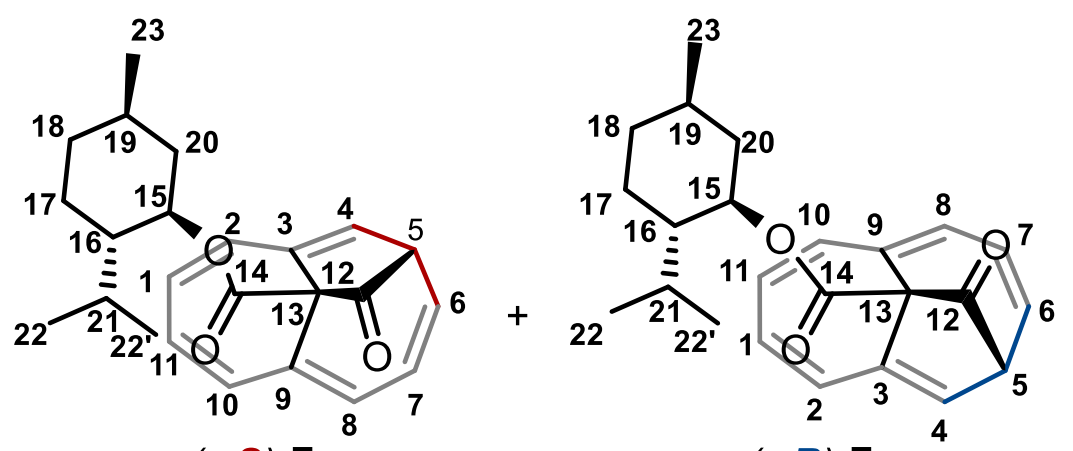

(L,*S*)-**7** (L,*R*)-**7**

(60:40)
$C_{24}H_{28}O_3$
M = 364.20 g mol^{-1}

According to a literature,[14] homoannulene (L)-**6** (50 mg, 137 μmol, 1.00 equiv) in $CDCl_3$ (3 mL) was irradiated with a Hg lamp equipped with a λ = 285 nm cut-off filter for 1 h at 0 °C. After reaching the photostationary state (monitored by ^{1}H NMR spectroscopy), all volatiles were removed under reduced pressure. The crude product was purified by flash column chromatography (cyclohexane/EtOAc = 9:1, 2 × 25 cm, 6 mL, #12–15) to obtain product (L)-**7** (6.00 mg, 16.5 μmol, 12%) as a yellow oil. The product was obtained as a diastereomeric mixture with the ratio of d.r. = 60:40, which was determined by NMR spectroscopy and HPLC (see Figure S1 S1 and Section 1.7.4).

R*f* = 0.53 (SiO_2, cyclohexane/EtOAc = 8:2).

For the ^{1}H NMR analysis, the product was isolated as a mixture of two diastereomers in a ratio of 60:40, as determined by the two isolated resonances of H-4 at δ = 4.43 ppm and 4.45 ppm. Wherever possible, the distinct signals of each diastereomer were assigned and marked with & and §, respectively.

^{1}H NMR (600 MHz, $CDCl_3$): δ = 0.64–0.69 (m, 3H, H-23), 0.76–0.99 (m, 9H, H-22, H-22', H-17a, H-18a, H-20a), 1.32 (m_c, 1H, H-16), 1.43 (m_c, 1H, H-21), 1.63 (m_c, 2H, H-17b, H-18b), 1.89 (m, 1H, H-20b), 2.03 (m, 1H, H-19), 3.69–3.72 (m, 1H, H-5), 4.44 (d, $^3J_{4,5}$ = 2.4 Hz, 0.4H, H-4$^{\&}$), 4.45 (d, $^3J_{4,5}$ = 2.4 Hz, 0.6H, H-4§), 4.54–4.60 (m, 1H, H-15), 5.48–5.53 (m, 1H, H-6), 6.04 (m_c, 1H, H-11), 6.10 (m_c, 1H, H-8), 6.15–6.20 (m, 2H, H-1, H-7), 6.74 (m_c, 1H, H-2), 6.97–7.01 (m, 1H, H-10) ppm. The sample contains traces of homoannulene (L)-**6**

^{13}C NMR (151 MHz, $CDCl_3$): δ = 15.7 (C-23), 15.8 (C-23), 20.9 (C-22)*, 21.0 (C-22)*, 22.1 (C-22)*, 22.2 (C-22)*, 23.0 (C-18), 23.1 (C-18), 25.3 (C-19), 25.7 (C-19), 31.5 (2 signals, C-21), 34.3 (C-17), 34.4 (C-17), 40.1 (C-20), 40.4 (C-20), 46.8 (C-16), 46.9 (C-16), 51.8 (2 signals, C-5), 67.4 (C-13), 67.7 (C-13), 75.9 (C-15), 76.1 (C-15), 92.7 (C-4$^{\&}$), 93.2 (C-4§), 119.6 (C-6), 119.8 (C-6), 120.9 (C-8), 121.1 (C-8), 122.1 (C-11), 122.3 (C-11), 123.7 (C-7), 123.9 (C-7), 127.1 (2 signals, C-1), 128.9 (C-2), 129.1 (C-2), 133.5 (C-3) 133.6 (C-3), 133.7 (C-9), 134.5 (C-9) 135.0 (C-10), 135.2 (C-10), 166.9 (2 signals, C-14), 205.5 (2 signals, C-12) ppm.

HRMS (ESI) for $C_{24}H_{29}O_3^+$ [$(M+H)^+$]: calculated: 365.2111; found: 365.2113.

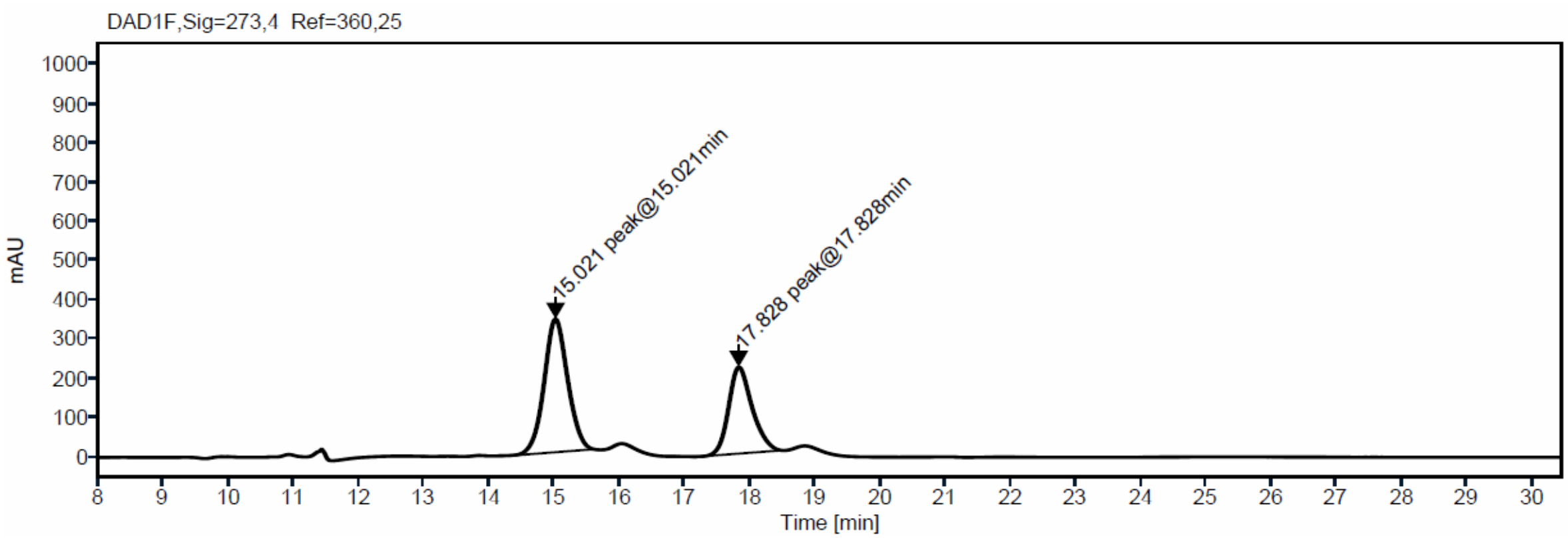

Signal: DAD1F,Sig=273,4 Ref=360,25

Name	RT [min]	Area	Peak Area Percent
peak@15.021min	15.02	8508.910	60.63
peak@17.828min	17.83	5524.500	39.37

Figure S1. HPLC chromatogram of isolated *(*L*,S)*-**7** and *(*L*,R)*-**7**. CHIRALPAK AD-H, 0.4 mL · min^{-1}, *n*-heptane/*i*PrOH = 90:10.

1.6.3.12 (1*R*,2*R*,5*S*)-2-Isopropyl-5-methylcyclohexyl 3-bromo-1-oxo-1,2-dihydro-9aH-2,9-ethenobenzo[7]annulene-9a-carboxylate (L,*S*)-13, (L,*R*-13)

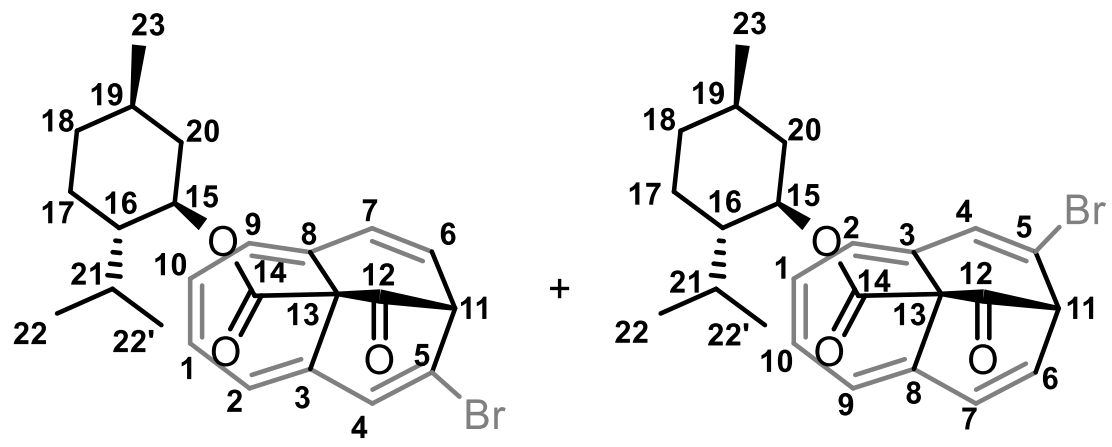

$C_{24}H_{27}O_3Br$
M = 442.11 g mol^{-1}

In deference to a literature,[15] a round-bottom flask was charged with homoannulene (L)-**6** (40.0 mg, 0.110 mmol, 1.00 equiv.) in CH_2Cl_2 (3.1 mL). Pyridinium tribromide (35.1 mg, 0.110 mmol, 1.00 equiv) was added. The reaction was stirred at room temperature for 45 h. After complete conversion was detected (monitored by TLC), the reaction mixture was filtered through a plug silica gel (0.5 × 2 cm, eluent: CH_2Cl_2, 50 mL). The crude product was purified by flash column chromatography on silica gel (cyclohexane/EtOAc = 10:1, 1 × 10 cm, 3 mL, #3–13) to afford the two diastereomers of bromide (L,*S*)-**13** and (L,*R*)-**13** (30.0 mg, 0.0677 mmol, 62%) as a brown oil.

Their ratio of (L,*S*)-**13** and (L,*R*)-**13** was determined to be 50:50 as can be determined from HPLC (Figure S3). In analogy with the analysis in homoannulene L-**6**, in addition the presence of rotamers is observed as can be seen for example from the ^{13}C NMR resonances at C-3 in a ratio of 1:1:1:1 (Figure S2). Therefore, in total four signals for each C-atom can be observed, wherever peak separation allows. Wherever assignment was possible, the rotamers are marked with § and $^{\&}$, the diastereomers $^{\alpha}$ and $^{\beta}$, respectively.

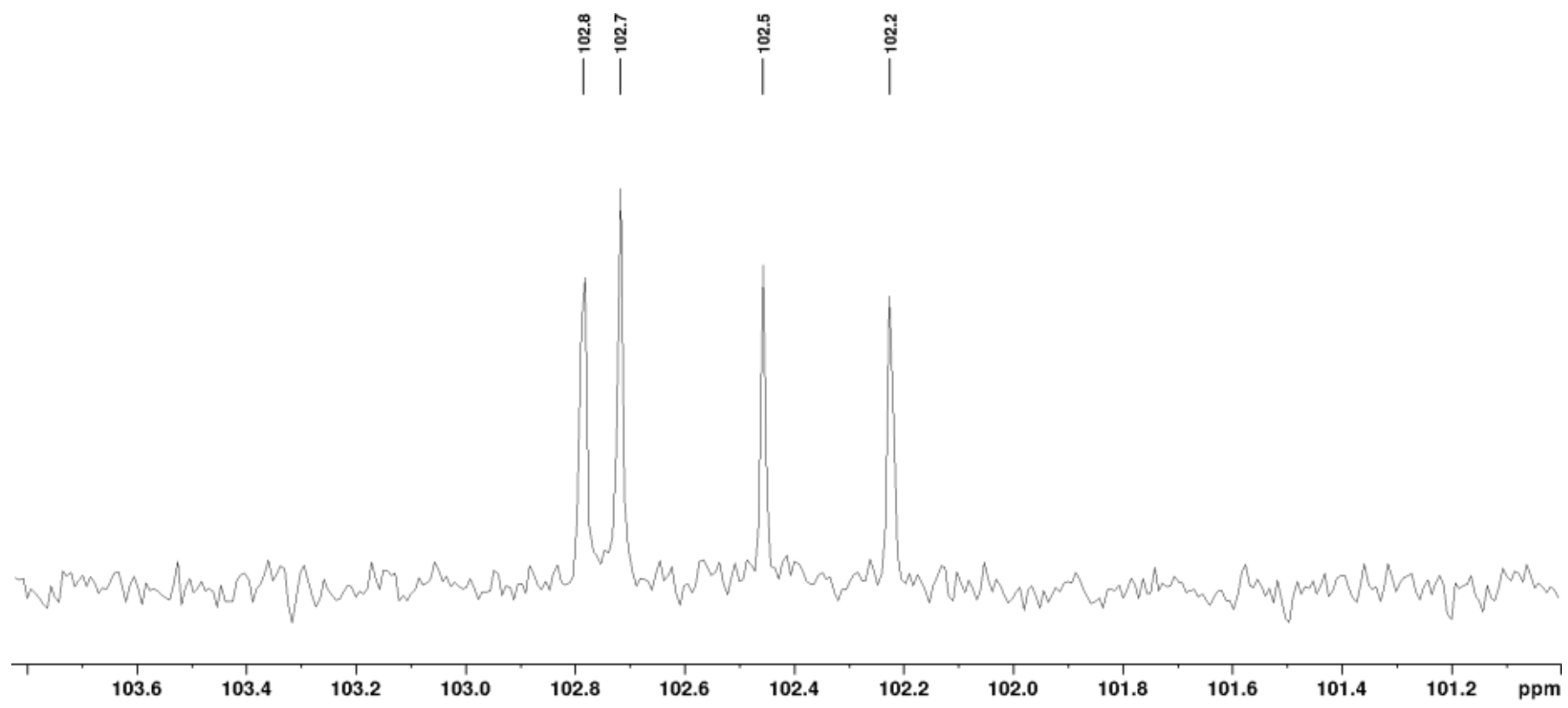

Figure S2. Section of the ^{13}C NMR spectrum of (L,*S*)-**13** and (L,*R*)-**13**.

$\mathbf{R_f}$ = 0.39 (SiO_2, cyclohexane/EtOAc = 10:1).

^{1}H NMR (600 MHz, $CDCl_3$): δ = 0.58 (d, $^3J_{23,19}$ = 6.9 Hz, 1.5H, H-23), 0.62–0.69 (m, 0.5H, H-20a§), 0.70 (d, $^3J_{23,19}$ = 6.9 Hz, 1.5H, H-23), 0.75–0.95 (m, 8.5H, H-20a$^{\&}$, H-22, H-22', H-17a, H-18a), 1.16–1.27 (m, 1H, H-16)*, 1.30–1.37 (m, 1H, H-21), 1.50–1.62 (m, 2.5H, H-20b§, H-17b, H-18b), 1.72 (m$_c$, 0.5H, H-19)*, 1.82 (m$_c$, 0.5H, H-19)*, 2.08 (m$_c$,

0.5H, H-20b$^{\&}$), 4.06 (pseudo-q, $^3J_{5,6}$ = 6.5 Hz, 1H, H-11), 4.23 (m$_c$, 0.5H, H-15$^{\&}$), 4.44 (m$_c$, 0.5H, H-15§), 5.32–5.40 (m, 1H, H-6), 6.67 (m$_c$, 1H, H-2)**, 6.72–6.80 (m, 2H, H-7, H-9)**, 6.83–7.07 (m, 3H, H-1, H-4, H-10)** ppm.

^{13}C NMR (151 MHz, $CDCl_3$): δ = 15.6 (C-23), 15.7 (C-23), 15.8 (C-23), 20.8 (C-22, C-22'), 20.9 (C-22, C-22'), 21.0 (C-22, C-22'), 22.1 (C-22, C-22'), 22.2 (C-22, C-22'), 22.7 (C-22, C-22'), 23.0 (C-18), 23.1 (C-18), 25.4 (4 signals, C-19)*, 31.4 (C-21), 31.5 (C-21), 34.3 (C-17), 39.4 (C-20§), 40.5 (2 signals$^{\alpha,\beta}$, C-20$^{\&}$), 46.5 (C-16)*, 46.8 (C-16)*, 54.6 (3 signals, C-11), 60.0 (4 signals, C-5, C-13), 75.8 (2 signals$^{\alpha,\beta}$, C-15$^{\&}$), 76.3 (2 signals$^{\alpha,\beta}$, C-15§), 102.2 (C-3), 102.5 (C-3), 102.7 (C-3), 102.8 (C-3), 110.9 (C-6). 111.0 (C-6), 111.2 (C-6), 111.4 (C-6), 120.7 (C-8), 120.9 (C-8), 121.1 (2 signals, C-8), 121.5 (C-8), 123.3 (C-2), 123.6 (C-2), 123.9 (C-2), 124.1 (2 signals, C-2), 124.5 (C-2), 124.7 (C-2), 127.8 (2 signals, C-4)**, 128.2 (C-4)**, 128.3 (C-4)**, 129.0 (C-9)**, 129.1 (C-9)**, 129.2 (C-9)**, 129.3 (3 signals, C-10)**, 129.5 (C-10)**, 129.6 (2 signals, C-1)**, 129.7 (2 signals, C-1)**, 164.8 (C-9$^{\&}$), 164.9 (C-9$^{\&}$), 165.0 (C-9§), 165.1 (C-9§), 199.9 (C-7), 200.0 (C-7), 200.2 (C-7), 200.4 (C-7) ppm.

IR (ATR): $\tilde{\nu}$ = 2956 (m), 2870 (w), 2363 (w), 1752 (s), 1580 (w), 1454 (w), 1372 (m), 1305 (m), 1249 (s), 1185 (m), 1125 (w), 1096 (w), 1062 (m), 1014 (w), 954 (m), 895 (w), 861 (w), 801 (w), 768 (m), 742 (m), 667 (w) cm^{-1}.

HRMS (APCI) for $C_{24}H_{28}O_3{}^{79}Br^+$ [$(M+H)^+$]: calculated: 443.1216; found: 443.1229.

Optical rotation: $[\alpha]_D^{20}$= –0.004 (c = 0.011 mol · L^{-1} in CH_2Cl_2).

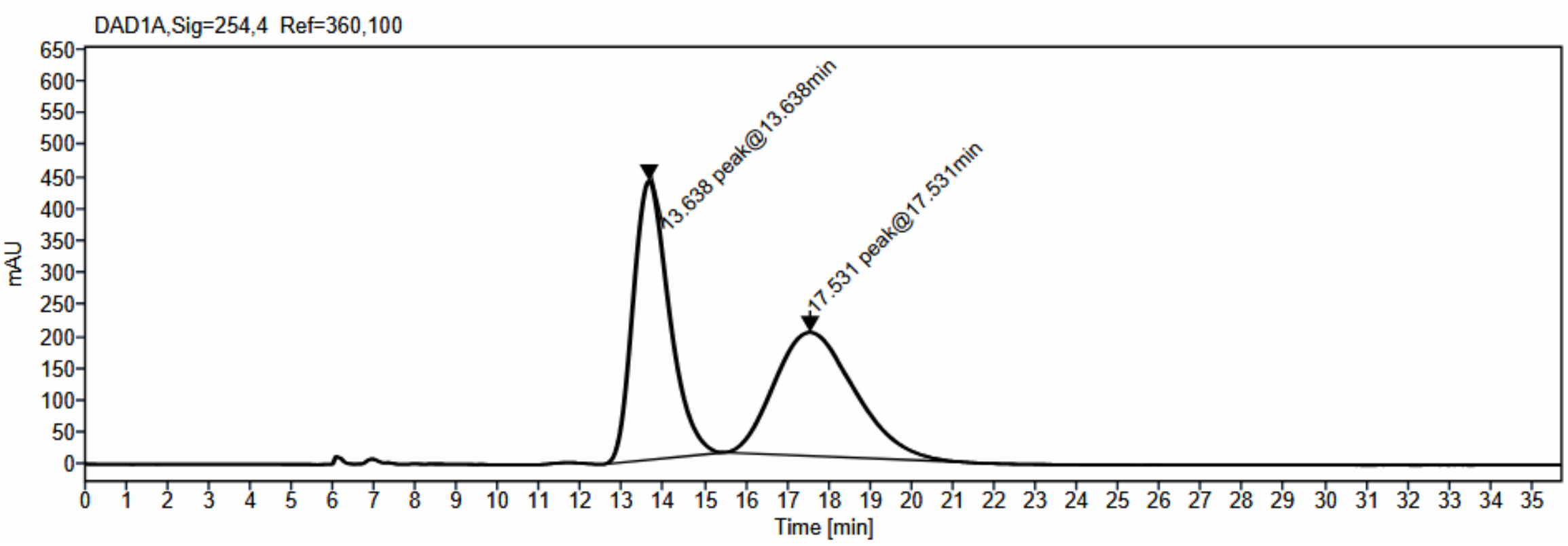

Signal:	DAD1A,Sig=254,4 Ref=360,100			
**Name**	**RT [min]**	**RF**	**Area**	**Peak Area Percent**
peak@13.638min	13.64		26529.048	50.42
peak@17.531min	17.53		26091.704	49.58

Figure S3. HPLC chromatogram of isolated (L,*S*)-**13** and (L,*R*)-**13**, CHIRALCEL OJ-H, 0.5 mL · min^{-1}, *n*-heptane/*i*PrOH = 90:10.

1.6.3.13 (1*S*,2*R*,5*S*)-2-isopropyl-5-methylcyclohexyl-1-hydroxy-1,2-dihydro-9a*H*-2,9-ethenobenzo[7]annulene-9a-carboxylate ((L,*S*)-14, (L,*R*)-14)

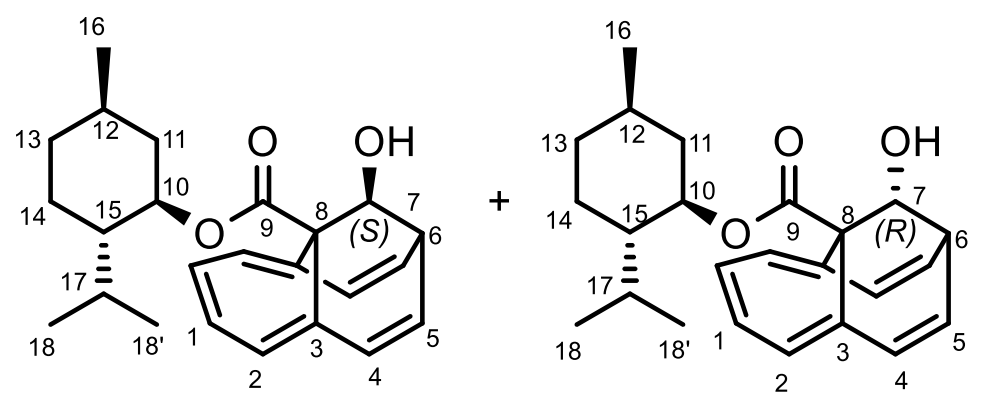

(L,*S*)-**14** (L,*R*)-**14**

A round-bottom flask is equipped with a stirring bar and homoannulene ester (L)-**6** (40 mg, 0.120 mmol, 1.00 equiv) and is dissolved in MeOH (4 mL). $NaBH_4$ (6.00 mg, 0.159 mmol, 1.45 equiv) is added and the reaction is stirred for 2 h at room temperature. Then 6 mL of 6 N HCl are added and the reaction mixture is stirred for additional 30 min at room temperature. All volatiles were removed under reduced pressure. The crude product is purified by flash column chromatography (1 × 19 cm, cyclohexane/EtOAc = 9:1, 3 mL, #9–19). The products (L)-**14** (23 mg, 0.0628 mmol, 57%) was obtained as a colorless oil.

R_f = 0.20 (SiO_2, cyclohexane/EtOAc = 8:2).

In the observed NMR spectra two distinct compounds can be found: The two alcohols (L,*S*)-**14** and (L,*R*)-**14** (see above) each have a distinct signal set. Wherever possible, the distinct signals of each diastereomer were assigned and marked with [&] and [§], respectively. Their ratio is 72:28 as can be determined from HPLC (Figure S4) and NMR with the resonance at δ = 4.19 and 4.27 ppm. The sample contains traces (<5%) of a side product, which could not be separated at this stage. A side product is observed in ^{1}H NMR at δ = 2.23, 2.28, 3.48, 3.63 ppm and in ^{13}C NMR at δ = 14.0, 14.3, 19.34, 27.1, 63.8, 68.6 and 71.3 ppm.

^{1}H NMR (600 MHz, $CDCl_3$): δ = 0.60 (d, J = 6.9 Hz, 3H, H-16), 0.67–0.73 (m, 1H, H-11a[$,&]), 0.76 ($m_c$, 1H, H-14a), 0.80–0.88 (m, 6H, H-18, H-18‘), 0.90–0.95 (m, 1H, H-13a), 1.19–1.27 (m, 1H, H-15), 1.32 (m_c, 1H, H-17), 1.56–1.61 (m, 2H, H-14b, H-13b), 1.62–1.65 (0.5H, m, H-11b[&]), 1.71 (m_c, 1H, H-11b[§] , 0.2H, H-12[&]), 1.82 (m_c, 0.8H, H-12[§]), 3.54 (m_c, 1H, H-6), 3.72 (dd, J = 9.1, 2.6 Hz, 1H, H-7), 4.19 (td, J = 10.8, 4.1 Hz, 0.72H, H-10[§]), 4.27 (td, J = 10.9, 4.2 Hz, 0.28H, H-10[&]), 5.14 (m_c, 1H, H-5), 5.28 (m_c, 1H, H-5’), 6.59 (pseudo-d, J = 9.3 Hz, 1H, H-4’), 6.66–6.70 (m, 1H, H-2’), 6.78–6.82 (m, 2H, H-2, H-4), 6.95–7.02 (m, 1H, H-1‘), 7.04–7.10 (m, 1H, H-1) ppm.

The O-*H* atom was not detected in the ^{1}H NMR spectrum. The sample contains traces of EtOAc.

^{13}C NMR (151 MHz, $CDCl_3$): δ = 15.6, (C-16$^{\&}$), 15.7 (C-16§), 21.0 (2 signals, C-18*), 22.1 (2 signals, C-18'), 22.8 (C-13§), 22.9 (C-13$^{\&}$), 25.5 (C-12§), 25.7 (C-12$^{\&}$), 31.4 (2 signals, C-17), 34.3 (2 signals, C-14), 37.1 (C-6$^{\&}$), 37.2 (C-6§), 40.1 (C-11§), 40.5 (C-11$^{\&}$), 46.7 (C-15§), 46.8 (C-15$^{\&}$), 53.3 (2 signals, C-8), 67.2 (C-7§), 67.3 (C-7$^{\&}$), 75.0 (C-10$^{\&}$), 75.1 (C-10§), 107.2 (C-5§), 107.3 (C-5$^{\&}$), 111.0 (C-5'$^{\&}$), 111.1 (C-5'§), 115.6 (C-3**§), 115.7 (C-3**$^{\&}$), 121.5 (C-3**§), 121.6 (C-3'**$^{\&}$), 122.8 (C-2'§), 122.9 (C-2'$^{\&}$), 123.8 (C-2$^{\&}$), 124.0 (C-2§), 126.9 (C-4'), 127.2 (C-4$^{\&}$), 127.4 (C-4§), 127.6 (C-1'§), 127.8 (C-1'$^{\&}$), 128.9 (C-1$^{\&}$), 129.0 (C-1§), 168.8 (C-9§), 169.2 (C-9$^{\&}$) ppm.

IR (ATR): $\tilde{\upsilon}$ = 3504 (br), 3037 (w), 2956 (s), 2870 (m), 1722 (s), 1454 (m), 1387 (m), 1245 (s), 1185 (m) 1081 (m), 1029 (m), 984 (m), 954 (m), 898 (w), 835 (w), 798 (w), 734 (s) cm^{-1}.

HRMS (ESI) for $C_{24}H_{3}O_{3}Na^{+}$ [$(M+Na)^{+}$]: calculated: 389.2087; found: 389.2079.

Optical rotation: $[\alpha]_{D}^{20}$= –0.075 (c = 0.005 mol · L^{-1} in CH_2Cl_2).

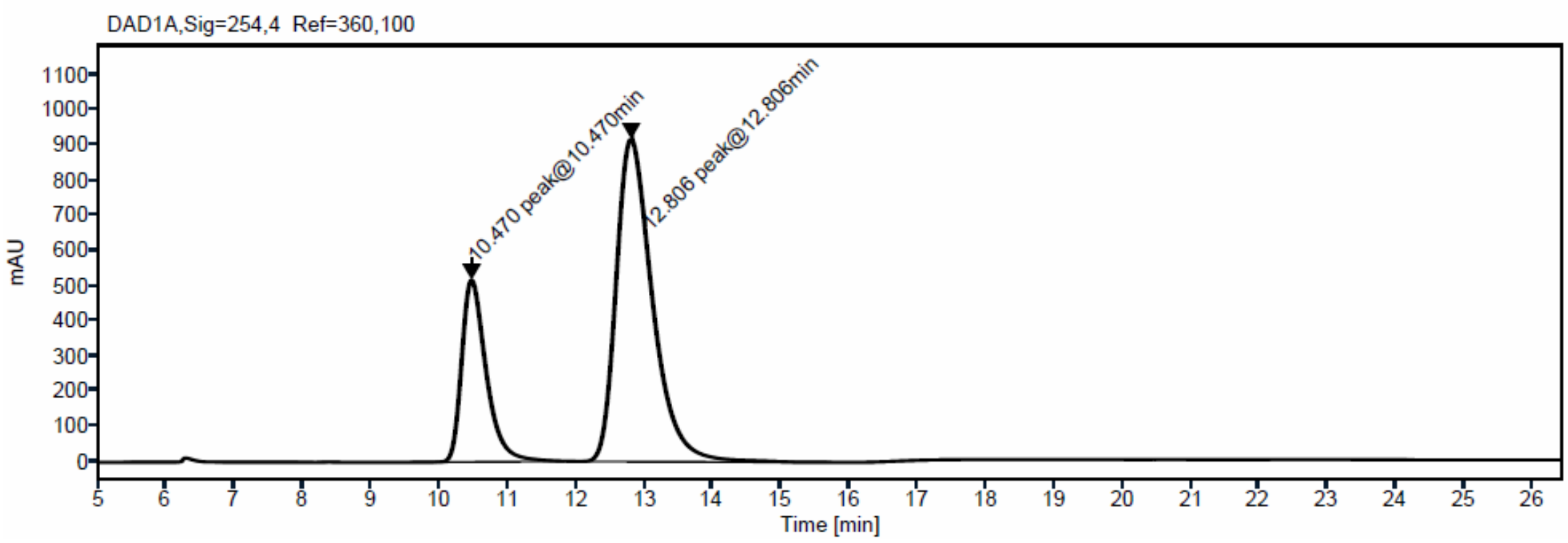

**Signal:**	DAD1A,Sig=254,4 Ref=360,100		
**Name**	**RT [min]**	**Area**	**Peak Area Percent**
peak@10.470min	10.47	12938.280	27.64
peak@12.806min	12.81	33876.589	72.36

Figure S4. HPLC chromatogram of isolated (L,*S*)-**14** and (L,*R*)-**14**, CHIRALCEL OJ-H, 0.5 mL · min^{-1}, *n*-heptane/*i*PrOH = 95:5.

1.7 Photoswitching Experiments

Homoannulene **6** was investigated regarding its photoswitching properties. By irradiation of homoannulene menthyl ester **6** the corresponding *iso*-homoannulenes *(L,S)*-**7** and *(L,R)* *(L,S)*-**7** are formed. The products **7** of this photochemical reaction are diastereomers which can be separated by HPLC and display distinct signals in ^{1}H and ^{13}C NMR experiments.

For concentrations of about $c = 2 \cdot 10^{-5}$ mol · L^{-1} (UV/vis absorption experiments) to $c = 9 \cdot 10^{-3}$ mol · L^{-1} (in the following referred to as "*photoswitching in small scale*", see Figure S5) the photoswitching of homoannulenes **6** was initiated by an LED of λ = 280 nm (forward switching process) and λ = 430 nm (backward switching progress) at 21 °C.

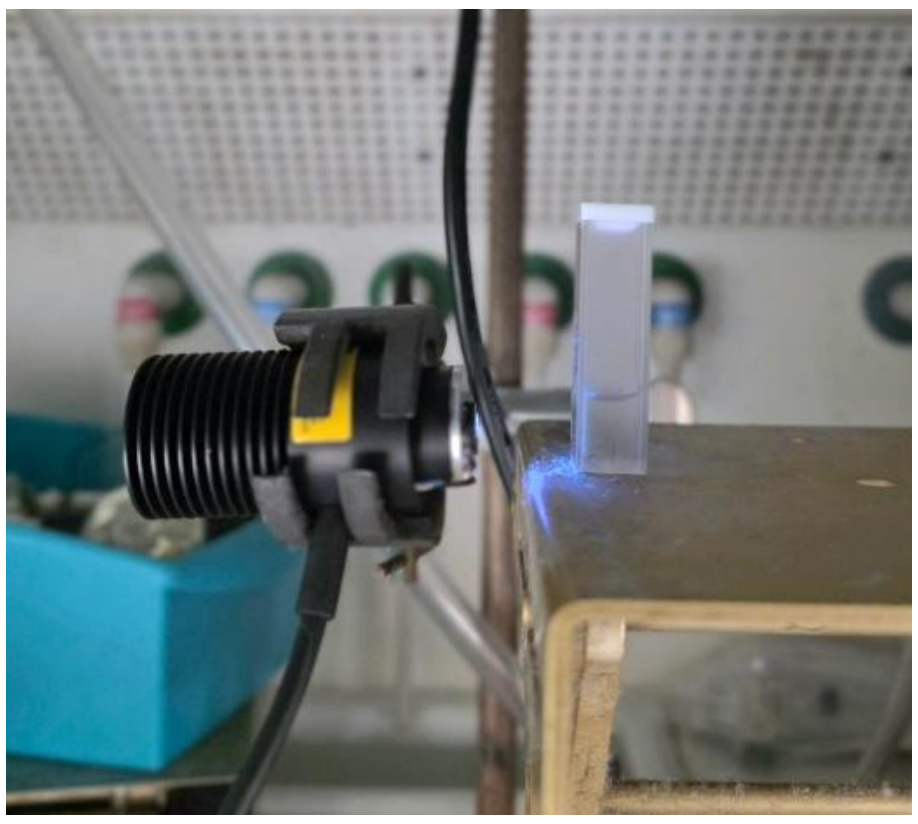

Figure S5. Set-up for photoswitching of homoannulenes **6** in a quartz class cuvette using mounted LEDs.

Furthermore, for concentrations of about $c = 0.05$ mol · L^{-1} (in the following referred to as "*photoswitching in large scale*") an Hg lamp equipped with a 285 nm cutoff filter (see Figure S6, left, forward switching process at 0 °C) and λ = 410 nm LED panel equipped with a fan cooler (see Figure S6, right, backward switching process at 21 °C) was used.

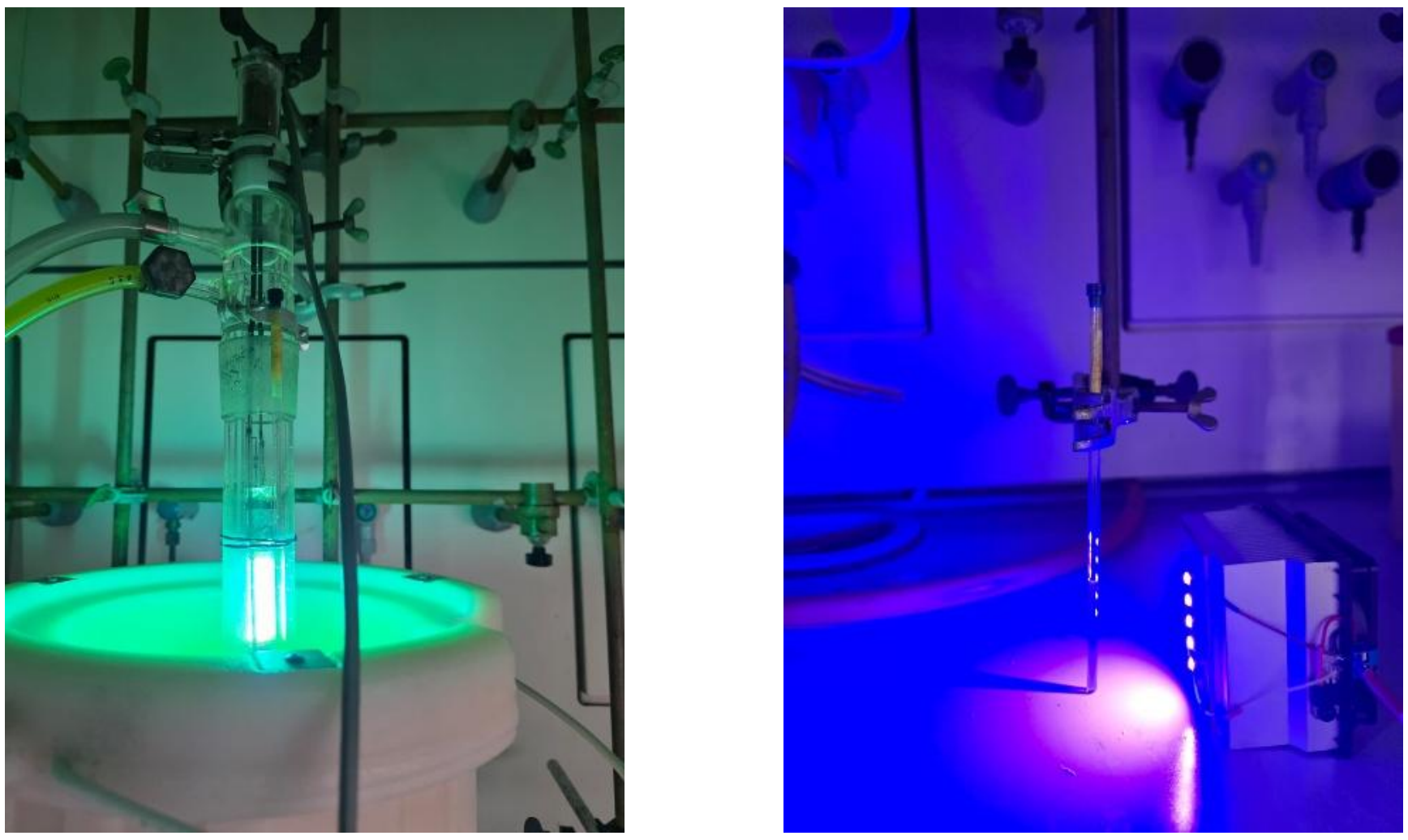

Figure S6. Photoswitching set-up using an Hg lamp equipped with a 285 nm cutoff filter (left) and LED panel equipped with a fan cooler (right).

The set-up used for the described experiments is mentioned in each experiment specifically.

1.7.1 Overview on photoswitching data

With both enantiomers of the menthyl homoannulene **6** in hand, we conducted experiments probing the ability of **6** to act as a chiroptical switch (Scheme S3). If the menthol auxiliary would exert stereodiscrimination onto the photochemical [1,11]-sigmatropic rearrangement, one enantiomer of the menthyl homoannulene (L)-**6** would lead to preferred movement towards one direction (top reaction, Scheme S3), such as *(*L*,S)*-**7**, whereas the enantiomeric homoannulene (D)-**6** would lead to the preferential formation of *(*L*,R)*-**7** (Scheme S3, bottom).[22] Combined, this leads to a scenario that upon the presence of calderoselectivity, the chiroptical switching should lead to one set of preferred enantiomers (*(*L*,S)*-**7** and *(*D*,R)*-**7** or *(*L*,R)*-**7** and *(*D*,S)*-**7**, respectively). Based on this hypothesis, we conducted several analyses of the photoswitching process with both enantiomers of the menthyl homonannulene **6**.

Photoswitching experiments of both enantiomers of **6** were carried out in MeCN as solvent. Upon irradiation of (D)-**6** or (L)-**6** with an LED at 280 nm for 3 min, we observed the diminishing of the signals at 255 and 310 nm, accompanied by an increase of a broad absorption at 380 nm (Scheme S3b, i). Alternatively, the photoswitching experiments could be carried out with similar results in larger scale with irradiation with a high pressure mercury lamp and a cutoff filter of 285 nm. From the latter, analysis of the resulting mixture gave a photostationary state ((L)-**6**/(L)-**7**= 41:59, as confirmed by HPLC analysis, see Section 1.7.4) after 60 minutes irradiation. Back-switching by irradiation with 430 nm (LED), confirmed by the reappearance of the signals at 255 and 310 nm, with full reformation of homoannulene (L)-**6** after 14 min,

resembling the photoswitching of the parent **4**.[23] The switching process could successfully cycled up to six times.[24]

In order to investigate the stereochemistry of the resulting *iso*-homoannulenes **7** (i.e. the stereochemical relationship of the four possible switching products of **7**), we carried out HPLC analysis on chiral stationary state of the individual irradiation experiments next (Scheme S3b ii). We observed that for each enantiomer of the chiral homoannulene **6**, two stereomers were formed, in a 56:44 ratio (for *(*L,*R)*-**7**/*(*L,*S)*-**7**) or *vice versa* for *(*D,*S)*-**7b**/*(*D,*R)*-**7**. Importantly, this analysis indicates that the preferred stereoisomers of each switching experiment individually (A or B, Scheme S3, top) are non-identical. These results support the above working hypothesis that each menthyl auxiliary directs the [1,11]-sigmatropic rearrangement into to one preferred direction (pro-*S* or pro-*R*). In order to fully support this assumed stereochemical pathway and a potential calderoselectivity, NMR and ECD analysis of the photoswitching experiments were carried out with the goal to identify the stereochemical relationship of the stereoisomers found in experiments A and B.

We analyzed the outcome of experiments A and B, carried out individually by ^{1}H NMR (Scheme S3b iii). We employed the hydrogen atom located at C-4 of the *iso*-homoannulene π system as probe for the ensuing stereochemical analysis. For this hydrogen atom, two signals were found, in a 56:44 for A and 44:56 for B. Of importance for the stereochemical analysis is the fact that for both A and B, the same signal is the major one (chemical shift of the major resonance of 4.45 ppm). This indicates that both enantiomers of the menthyl homoannulene **6** preferentially lead to the same diastereomer in the switching process.

As can be seen from the ^{1}H NMR spectra from photoswitching experiments A and B in Scheme S3b iii, we confirm the ratio of stereoisomers of 56:44 for A and 44:56 for B as indicated by the integrals of the two resonances found. The stereochemical analysis was complemented by ECD spectroscopy of the same mixtures of reactions A and B (Scheme S3b iv). As expected, before irradiation mirroring ECD spectra are found for the menthyl homoannulenes (L)-**6**/(D)-**6** (for a details, see Section 1.7.7). Importantly, also after photoswitching, the measured ECD spectra remained mirror images of each other. These experiments complete the stereochemical picture of the photoswitching process: the combination of NMR and ECD spectroscopy underlines that the formed major isomers formed in the photoswitching are the same diastereomer (as indicated by the same major peak in the ^{1}H NMR) and opposite enantiomers as shown by the ECD spectra.

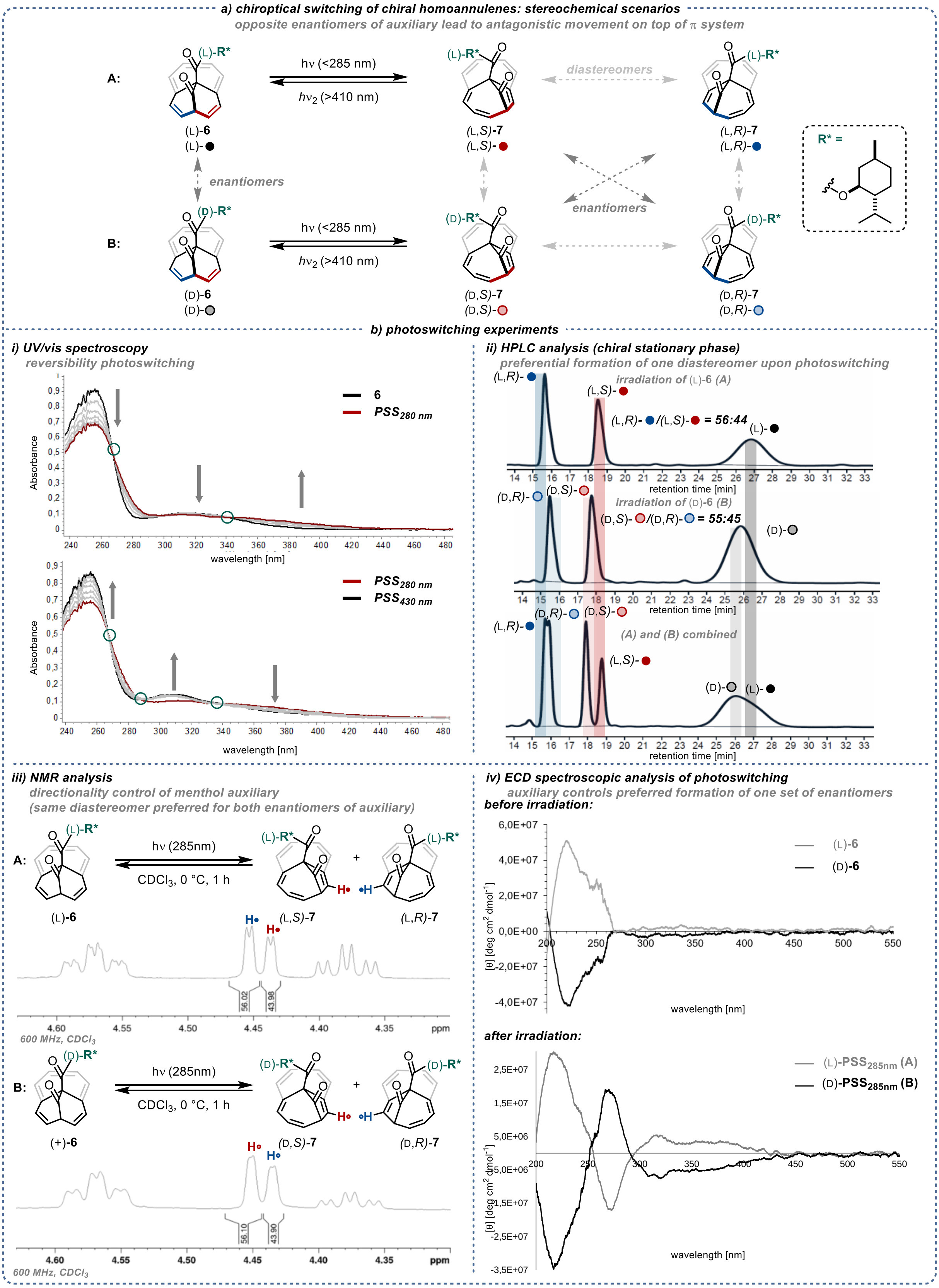

Scheme S3. i) Detected UV/vis spectra of irradiation of (L)-**6** (c = 4.36 · 10^{-5} mol · L^{-1}, black curve) at 280 nm in MeCN. Spectra were detected every 30 s for 3 min (grey curves) until PSS$_{280\ nm}$ (red curve) was reached. Bottom: Detected UV/vis spectra of mixture at PSS$_{430\ nm}$ (c = 4.36 · 10^{-5} mol · L^{-1}, red curve) which was irradiated

at 430 nm for 14 min in MeCN. Spectra were detected every 2 min (grey curves) until $PSS_{430\,nm}$ was reached (black curve). ii) HPLC chromatograms: Top: HPLC chromatogram after irradiation of (L)-**6** ($c_{(L)\text{-}\mathbf{6}}$ = 0.0412 mol · L^{-1}) after irradiation with 285 nm for 1 h at 0 °C, $PSS_{285\,nm}$, (L)-**6**/(L)-**7** = 41:59; middle: HPLC chromatogram after irradiation of (D)-**6** ($c_{(D)\text{-}\mathbf{6}}$ = 0.0569 mol · L^{-1}) with 285 nm for 1 h at 0 °C, $PSS_{285\,nm}$, (D)-**6**/(D)-**7** = 56:44; bottom: HPLC chromatogram of (D)-**6** and (L)-**6** after irradiation with 285 nm for 1 h at 0 °C; CHIRALPAK AD-H, *n*-heptane/*i*PrOH = 90:10, flow rate: 0.4 mL min^{-1}, iii) ^{1}H NMR spectra of photoswitched mixture of (L)/(D)-**6**, (L)-**7** and (D)-**7** after irradiation at 285 nm for 1 h at 0 °C. iv Top: Detected CD spectra of (L)-**6** and (D)-**6** in MeCN. Bottom: CD spectra of photoswitched mixture of (L)/(D)-**6**, (L)-**7** and (D)-**7** in MeCN. Samples were irradiated at 285 nm for 1 h at 0 °C.

1.7.2 HPLC analysis of photoswitching of homoannulene methyl ester 4

Homoannulene methyl ester **4** was dissolved in MeCN (c = 0.0549 mol · L^{-1}) and analyzed by HPLC. The chiral CHIRALPAK AD-H column was used. As eluent *n*-heptane/*i*PrOH = 90:10 was used and a flow rate of 0.4 mL · min^{-1}. Homoannulene **4** was detected at a retention time of t = 28.3 min (Figure S7, top). The sample was irradiated with an Hg high pressure mercury lamp equipped with a cutoff filter of λ = 285 nm for 1 h at 0 °C until $PSS_{285\,nm}$ was reached. The reaction mixture was subsequently analyzed by HPLC on a chiral stationary phase (Figure S7, bottom). Two additional signals at t = 19.9 and 20.8 min were detected which can be assigned to the formed *iso*-homoannulenes **5**. The ratio between the two *iso*-homoannulenes **5** was detected to be $e.r._{(S)\text{-}\mathbf{5}/(R)\text{-}\mathbf{5}}$ = 48:52 at $PSS_{285\,nm}$ a ratio of homoannulene to *iso*-homoannulene was determined to be **4**/**5** = 35:65. It is shown that the two possible *iso*-homoannulenes **5** are formed as a racemic mixture, when no chiral substituent is appended to the homoannulene ester.

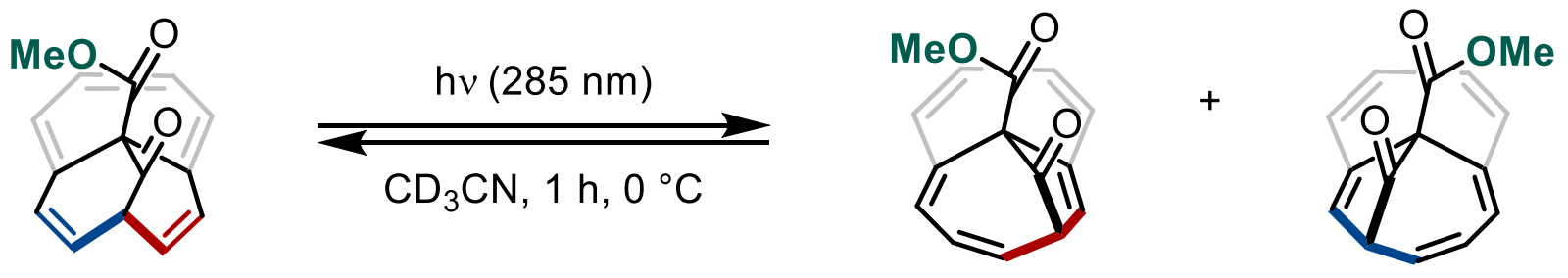

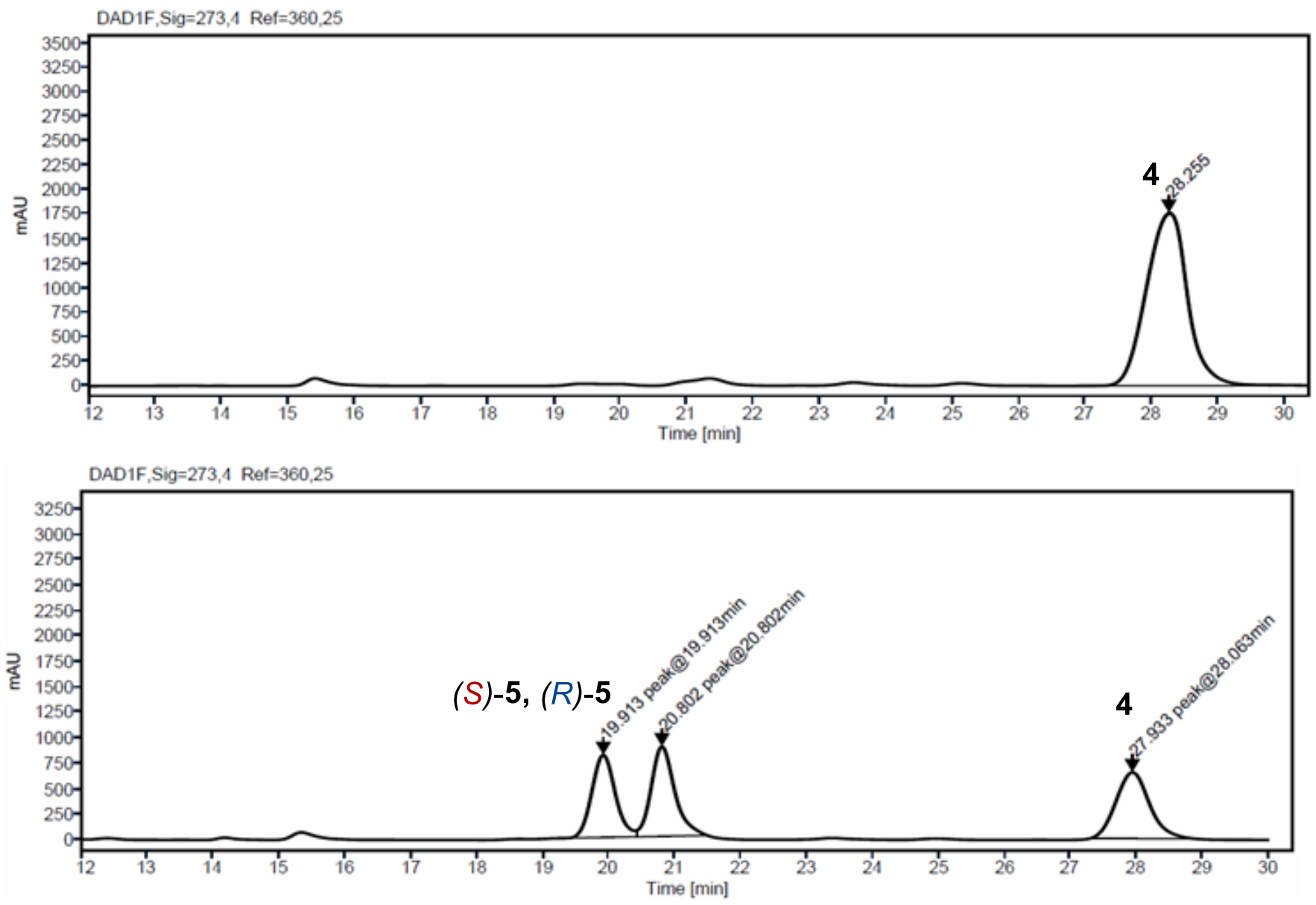

Signal: DAD1F,Sig=273,4 Ref=360,25

Name	RT [min]	Area	Peak Area Percent
peak@19.913min	19.91	20060.164	30.90
peak@20.802min	20.80	21933.765	33.79
peak@28.063min	27.93	22922.733	35.31

Figure S7. HPLC analysis of photoswitching of homoannulene methyl ester **4**. Top: HPLC chromatogram of homoannulene **4**. Bottom: HPLC chromatogram of mixture at PSS_{285nm}. CHIRALPAK AD-H, *n*-heptane/*i*PrOH = 90:10, flow rate: 0.4 mL · min^{-1}.

1.7.3 UV/vis absorption experiments of homoannulene menthyl ester 6

Photoswitching experiments were conducted and the reaction progress was monitored by UV/vis absorption spectroscopy (set-up: "*photoswitching in small scale*", Figure S8 and Figure S9). Upon irradiation of homoannulene (L)-**6** ($c = 4.36 \cdot 10^{-5}$ mol · L^{-1}) with an LED at λ = 280 nm absorption bands at λ = 255 nm and λ = 310 nm decrease while a broad absorption band emerged at 380 nm (Figure S8). Isosbestic points were detected at λ = 270 and 340 nm (marked with green circles in Figure S8).

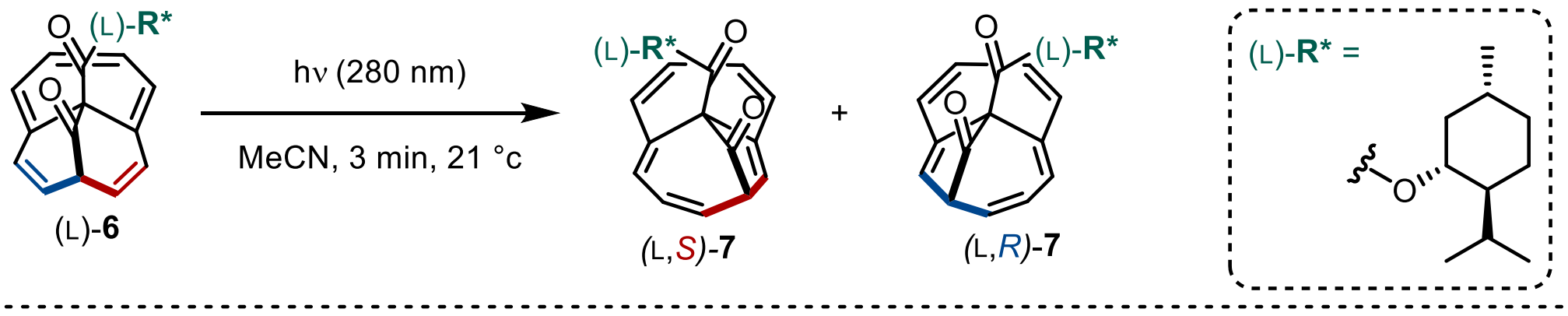

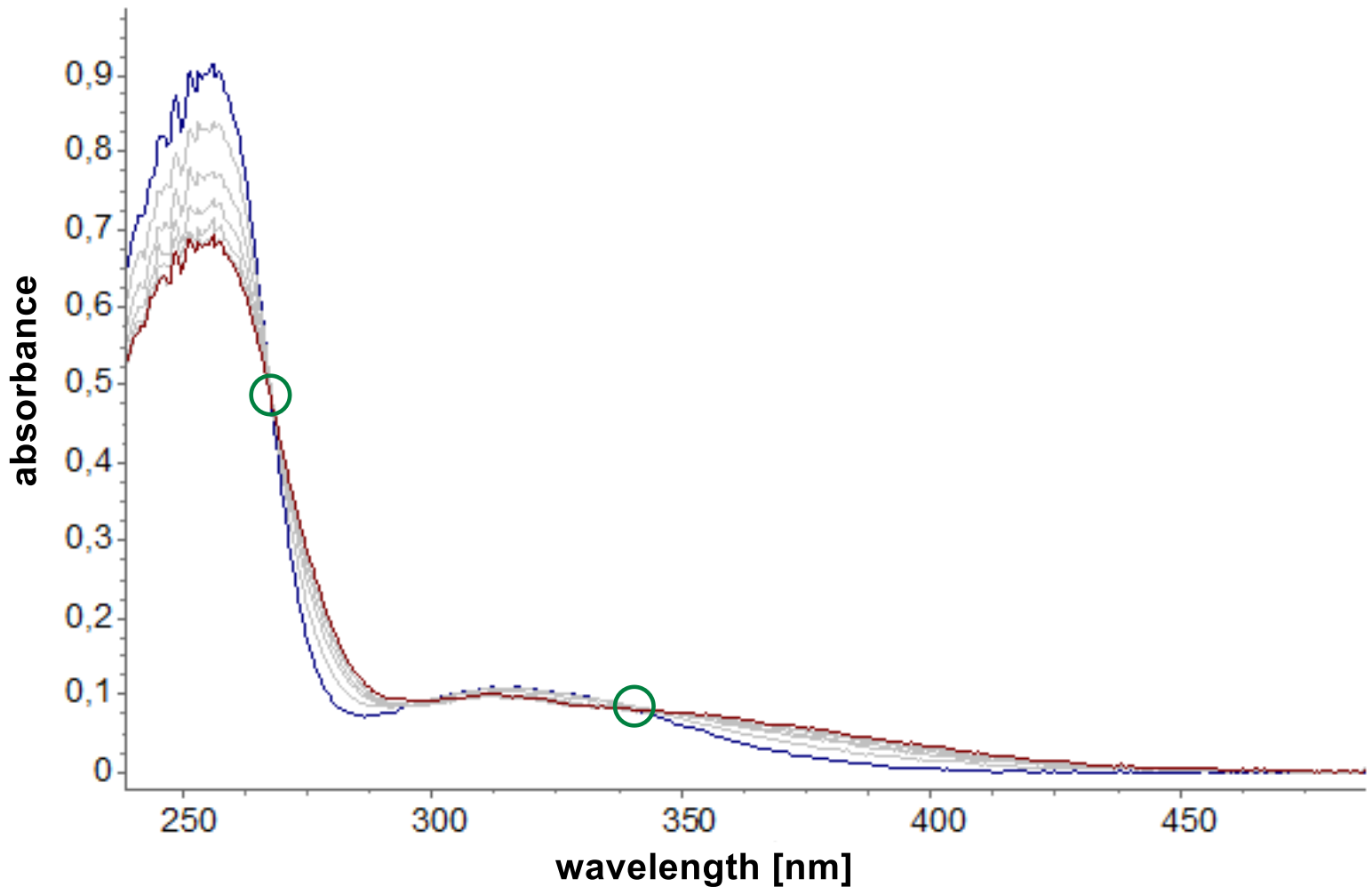

Figure S8. UV/vis absorption spectra of homoannulene (L)-**6** in MeCN ($c = 4.36 \cdot 10^{-5}$ mol · L^{-1}) before irradiation (blue graph), during irradiation (grey graphs) and at the $PSS_{280\ nm}$ (red graph). **6** was irradiated using an LED of λ = 280 nm for an irradiation time of 3 min. Absorption spectra were measured every 30 s.

Irradiation of the mixture obtained at $PSS_{280\ nm}$ of homoannulene (L)-**6** and *iso*-homoannulenes (L)-**7** (Figure S9, red graph) with an LED at λ = 430 nm for 14 min, regenerated the absorption spectrum of homoannulene (L)-**6** (Figure S9, blue graph). Under these conditions, the absorption bands at λ = 255 nm and λ = 310 nm increased in intensity, while the absorption band at λ = 380 nm decreased in its intensity. The photoswitching process toward homoannulene (L)-**7** exhibits isosbestic points at λ = 270 nm and λ = 340 nm (marked with green circles in Figure S9).

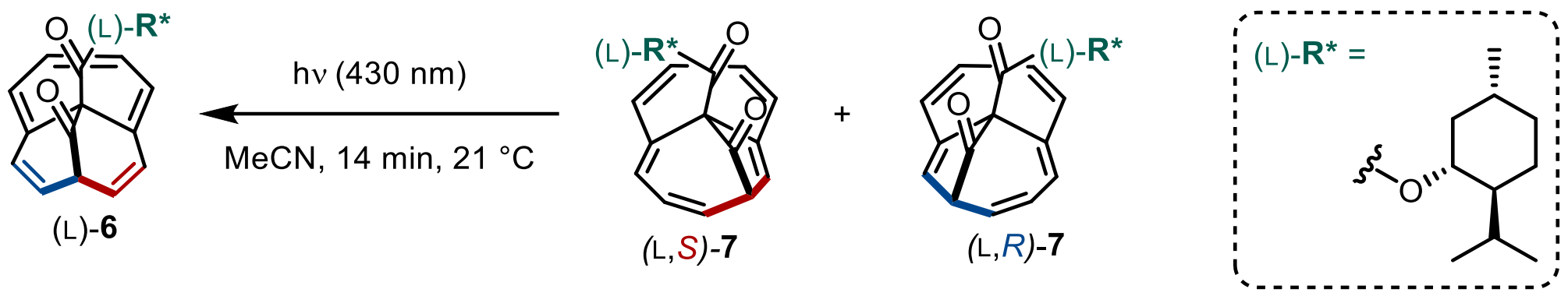

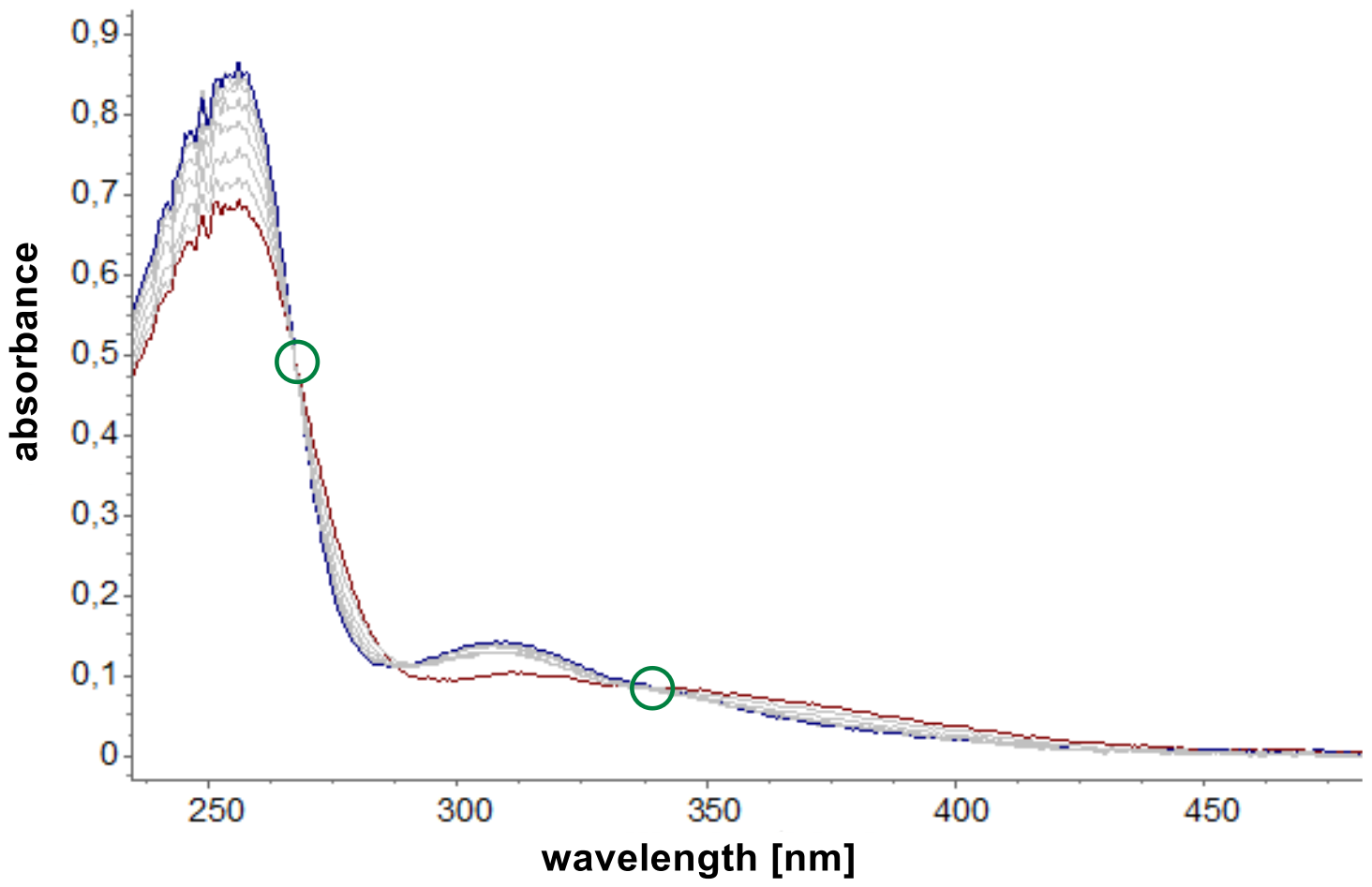

Figure S9. UV/vis absorption spectra of the mixture of (L)-**6** and (L)-**7** obtained at $PSS_{280\ nm}$ ($c = 4.36 \cdot 10^{-5}$ mol · L^{-1}, red graph), during irradiation with a 430 nm LED (grey graphs) and at $PSS_{430\ nm}$ (blue graph). The sample was irradiated for 14 min and spectra were measured every 2 min.

Homoannulene menthyl ester (L)-**6** was irradiated with an LED of λ = 280 nm for 3 min and after reaching the photostationary state with an LED at λ = 430 nm for 14 min to complete one cycle of photoswitching. (Details of cycles of photoswitching will be given in section 1.7.6.3) The spectra of the starting material (L)-**6** (Figure S10, blue graph) and up to six cycles of photoswitching were recorded (Figure S10). The spectra display a comparable absorption band with its maxima at λ = 255 nm. The absorption band detected in homoannulene ester (L)-**6** with a maximum at λ = 310 nm, shifts slightly after one cycle of photoswitching (3 min irradiation at λ = 280 nm and 14 min of irradiation at λ = 430 nm) to an absorption maximum at λ = 305 nm, which is consistent up to six cycles of photoswitching. The decreased absorption could be accounted for by a photodegradation process, which will be detailed in section 1.7.6).

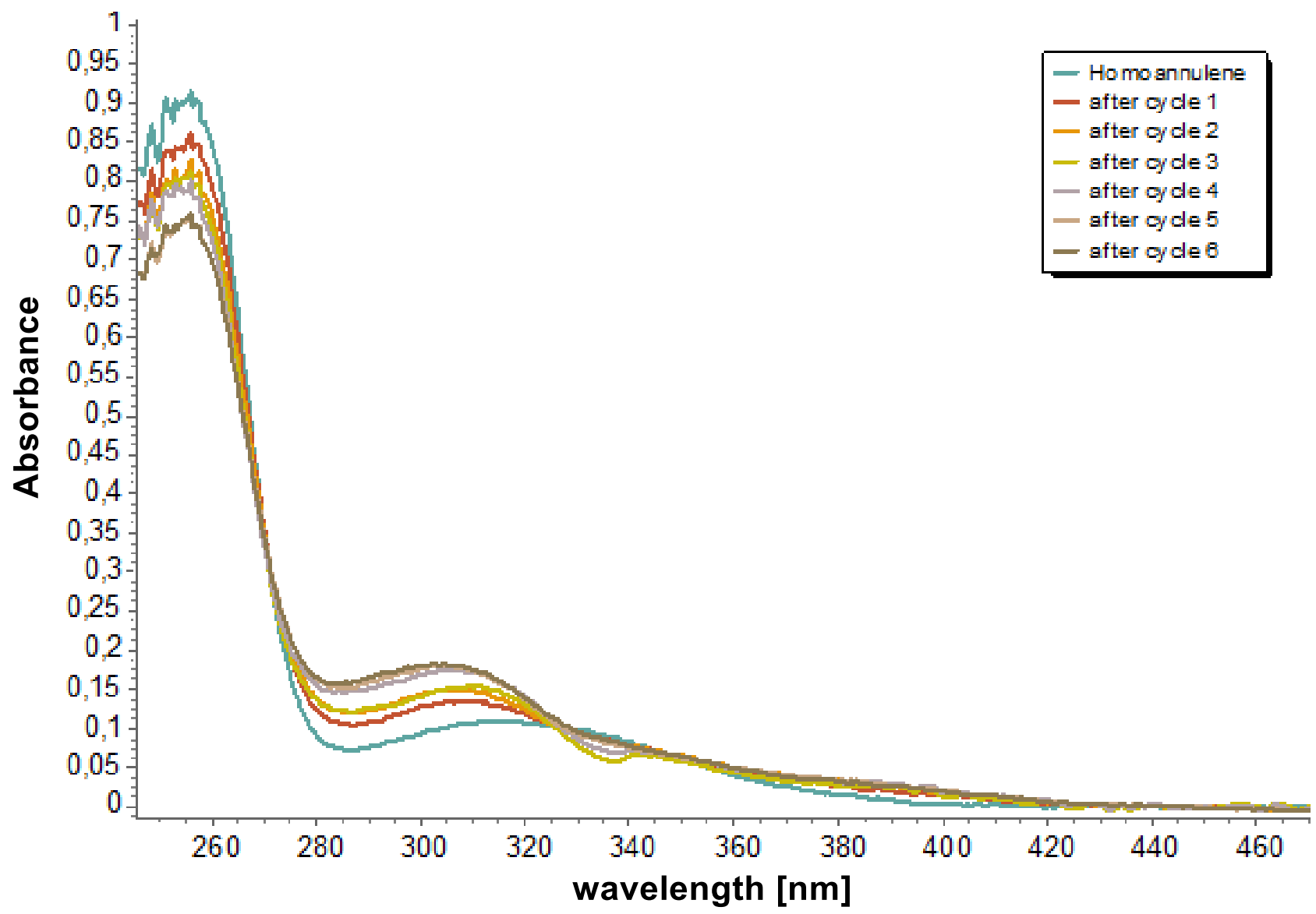

Figure S10. UV/vis absorption spectra of (L)-**6** and after up to six cycles of photoswitching. The sample of (L)-**6** was irradiated for 3 min with λ = 280 nm and for 14 min with λ = 430 nm in MeCN (c = 2.2 10^{-5} mol · L^{-1}).

1.7.4 Determination of PSS and d.r.

1.7.4.1 Analysis by HPLC

The diastereomeric ratio of products *(*L,S*)*-**7**/(D,*S*)-**7** and *(*L*,R)*-**7**/(D,*R*)-**7** of the photochemical reaction of homoannulenes (D)-/(L)-**6** as well as the ratio at PSS_{285nm} of homoannulene **6** to *iso*-homoannulene **7** was determined by HPLC analysis. For both, homoannulene ester (L)-**6** and (D)-**6** and their photoswitched products *(*L,S*)*-**7**/(D,*S*)-**7** and *(*L*,R)*-**7**/(D,*R*)-**7** the CHIRALPAK AD-H column was used. As eluent *n*-heptane/*i*PrOH = 90:10 was used with a flow rate of 0.4 mL · min^{-1}.

Homoannulene (L)-**6** was dissolved in MeCN-d_3 (c = 0.0412 mol · L^{-1}) and analyzed by HPLC. One signal was detected with a retention time of t = 26.5 min (Figure S11, top). After irradiation with a Hg high pressure mercury lamp equipped with a cutoff filter of λ = 285 nm for 1 h at 0 °C (set-up: "*photoswitching in large scale*") the reaction mixture of homoannulene ester (L)-**6** and *iso*-homoannulene esters *(*L*,S)*-**7** and *(*L*,R)*-**7** was subjected to HPLC. Two additional signals with a retention time of t = 15.5 and 17.7 min were detected as well as the signal corresponding to (L)-**6** (Figure S11, bottom). A ratio of homoannulene (L)-**6** to *iso*-homoannulene (L)-**7** of (L)-**6**/(L)-**7** = 41:59 as well as a d.r. of *iso*-homoannulenes (L)-**7** of $d.r._{(L,S)\text{-}7/(L,R)\text{-}7}$ = 44:56 was determined. With the combination of NMR and ECD spectroscopy it was shown that the formed major isomers formed in the photoswitching are the same diastereomer (as indicated by the same major signal in the ^{1}H NMR spectroscopy) and

opposite enantiomers as shown by the ECD spectra (see Section 1.7.7). With that it can be rationalized that (L,*R*)-**7** is formed preferentially in the [1,11]-sigmatropic rearrangement of homoannulene (L)-**6**.

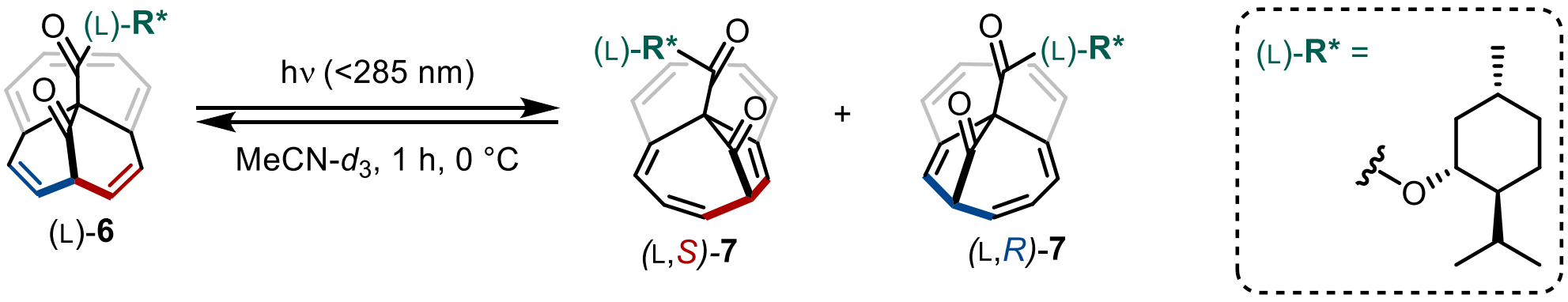

before irradiation:

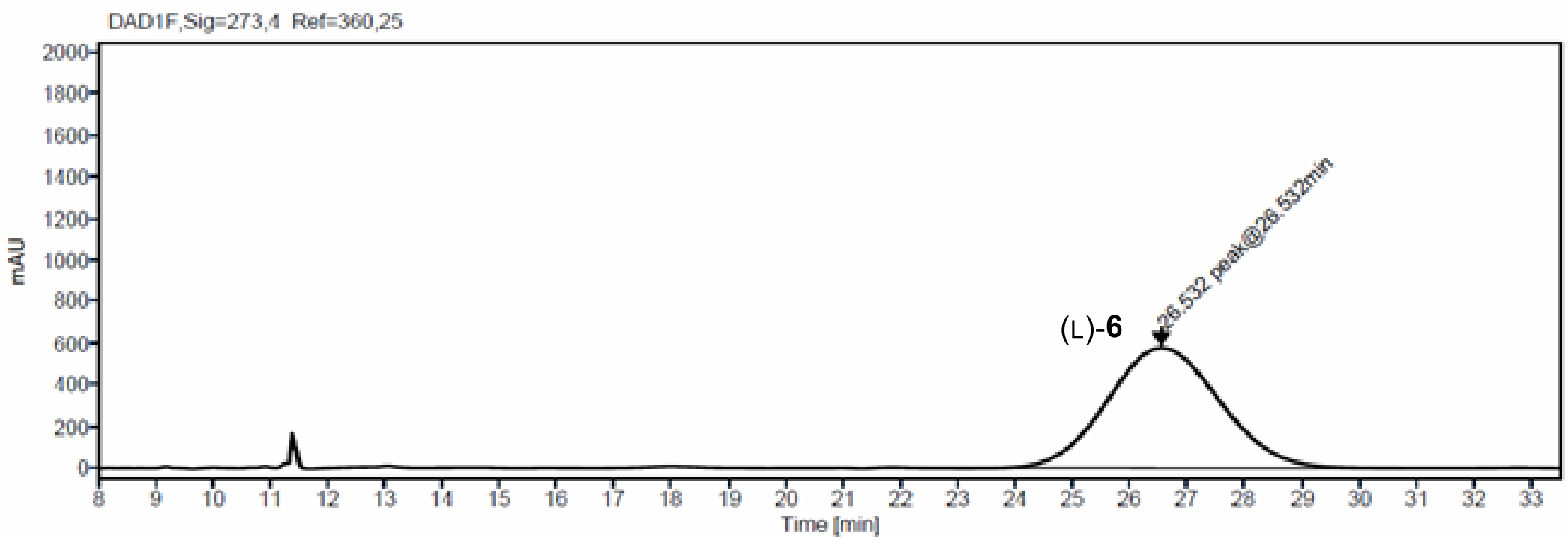

$PSS_{285\ nm}$

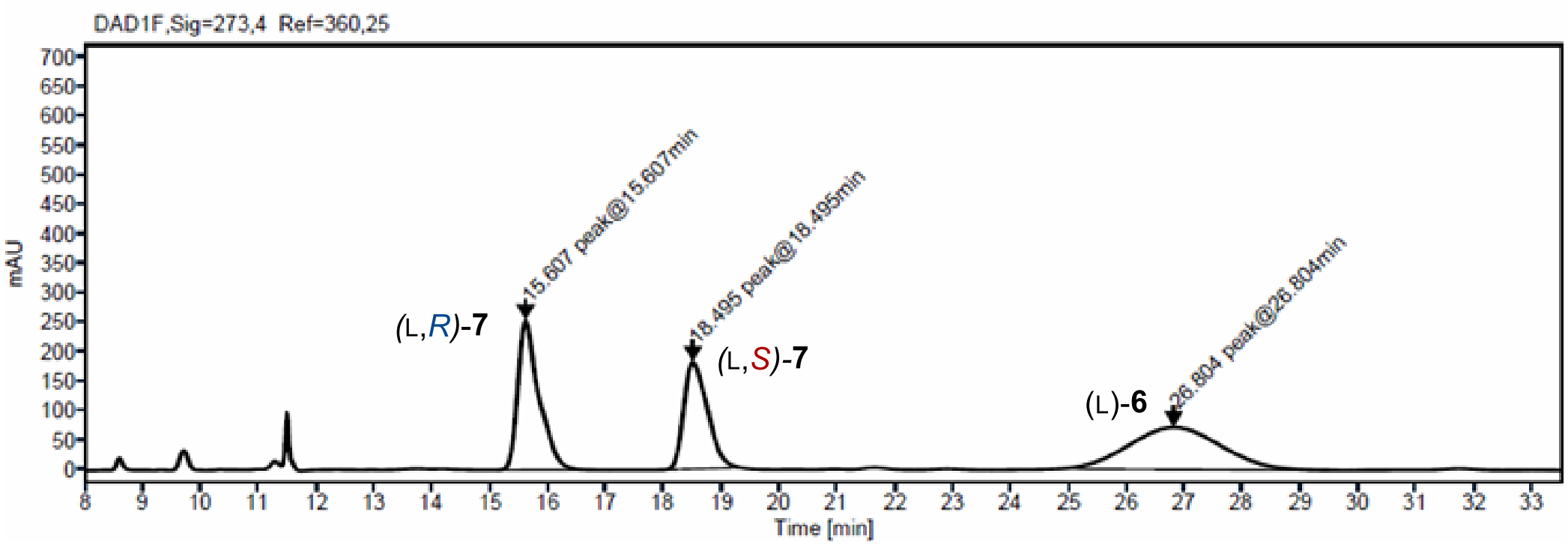

Signal: DAD1F,Sig=273,4 Ref=360,25

Name	RT [min]	Area	Peak Area Percent
peak@15.607min	15.61	6445.107	33.01
peak@18.495min	18.50	5138.439	26.32
peak@26.804min	26.80	7941.110	40.67

Figure S11. Top: HPLC chromatogram of homoannulene (L)-**6**. Bottom: HPLC chromatogram at PSS_{285nm} of the mixture of (L)-**6** and (L)-**7**. CHIRALPAK AD-H, *n*-heptane/*i*PrOH = 90:10, flow rate: 0.4 mL · min^{-1}.

Furthermore, homoannulene ester (D)-**6** was analyzed by HPLC. For that (D)-**6** was dissolved in MeCN-d_3 (c = 0.0569 mol · L^{-1}), subjected to HPLC and detected at a retention time of t = 25.7 min (Figure S11, top). After irradiation of homoannulene ester (D)-**6** for 1 h at 0 °C with an Hg lamp and a 285 nm cutoff filter (set-up: "*photoswitching in large scale*") the sample was analyzed by HPLC. Two new signals at t = 15.8 and 18.0 min as well as the signal of homoannulene (D)-**6** were detected (Figure S11, bottom). From that a ratio of homoannulene

(D)-**6** to *iso*-homoannulenes (D)-**7** of (D)-**6**/(D)-**7** = 56:44 was determined. Additionally, a diastereomeric ratio for *(*D,*S)*-**7**/*(*D,*R)*-**7** can be calculated to be d.r.$_{(D,S)\text{-}7/(D,R)\text{-}7}$ = 55:45.

With the combination of NMR and ECD spectroscopy it was shown that the formed major isomers formed in the photoswitching are the same diastereomer (as indicated by the same major signal in the ^{1}H NMR spectroscopy) and opposite enantiomers as shown by the ECD spectra (see Section 1.7.7). With that it can be rationalized that *(*D,*S)*-**7** is formed preferentially in the [1,11]-sigmatropic rearrangement of homoannulene (D)-**6**.

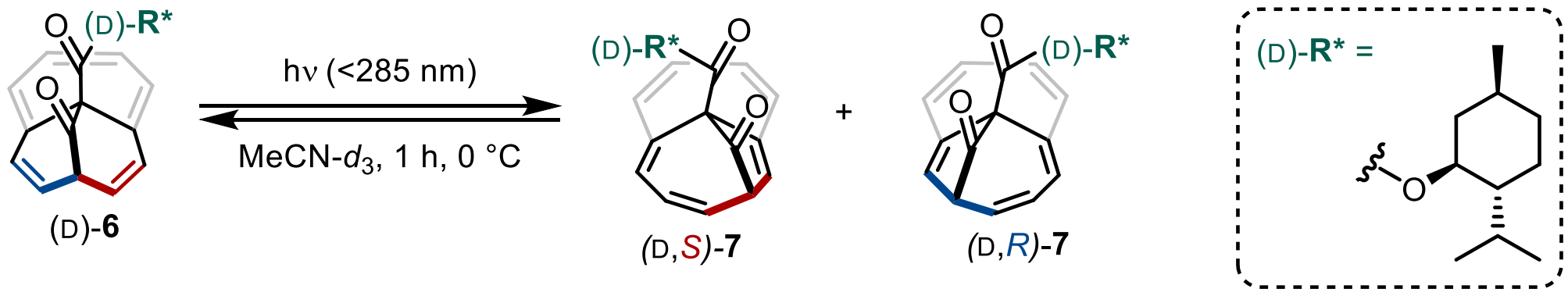

before irradiation:

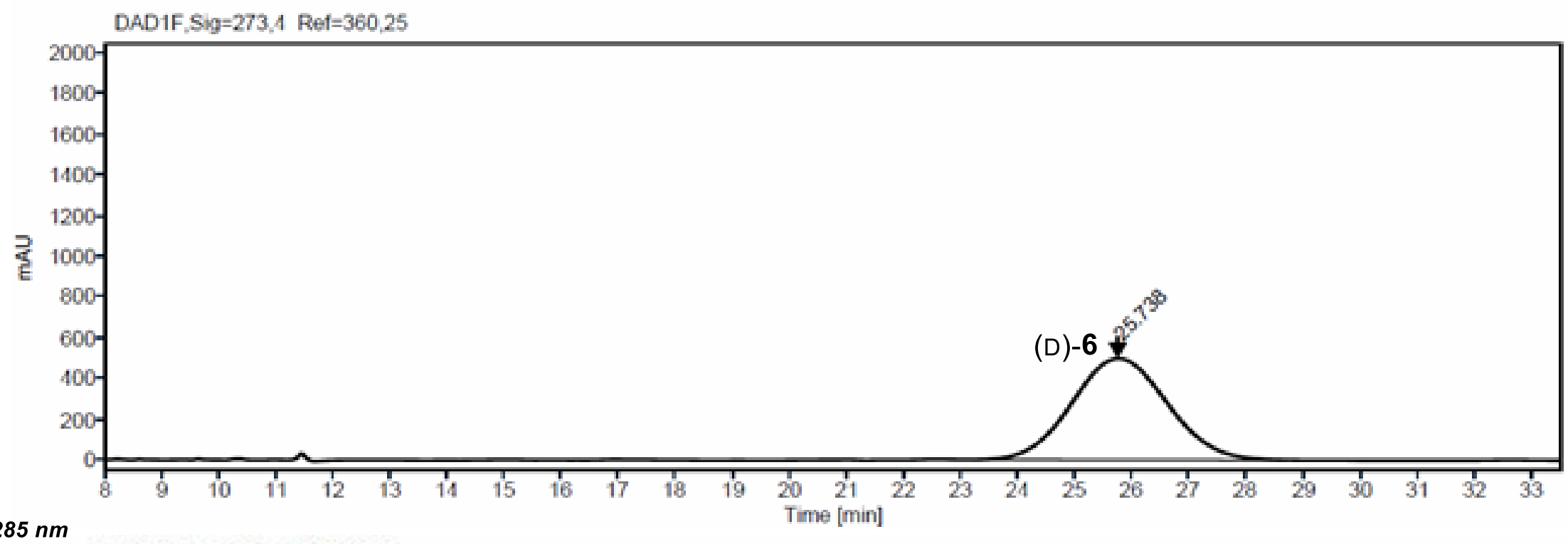

PSS$_{285\ nm}$

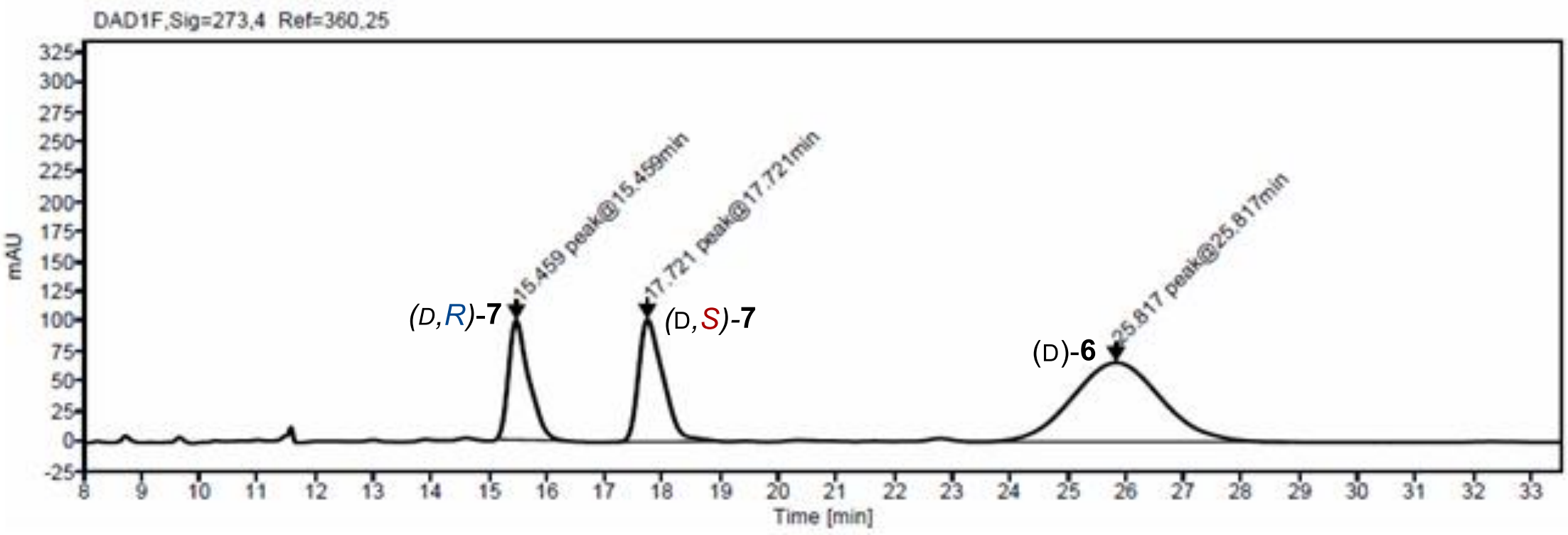

Signal: DAD1F,Sig=273,4 Ref=360,25

Name	RT [min]	Area	Peak Area Percent
peak@15.459min	15.46	2447.153	19.58
peak@17.721min	17.72	2993.428	23.96
peak@25.817min	25.82	7055.032	56.46

Figure S11. Top: HPLC chromatogram of homoannulene ester (D)-**6**. Bottom: HPLC chromatogram at PSS$_{285nm}$ of the mixture of (D)-**6** and (D)-**7,** flow rate: 0.4 mL · min^{-1}, CHIRALPAK AD-H, *n*-heptane/*i*PrOH = 90:10.

Homoannulene esters (L)- and (D)-**6** could be separated in HPLC on a chiral stationary phase (CHIRALPAK OJ-H) when being mixed (Figure S12). Homoannulene ester (L)-**6** was detected at t = 29.9 min whereas (D)-**6** was detected at a retention time of t = 35.03 min.

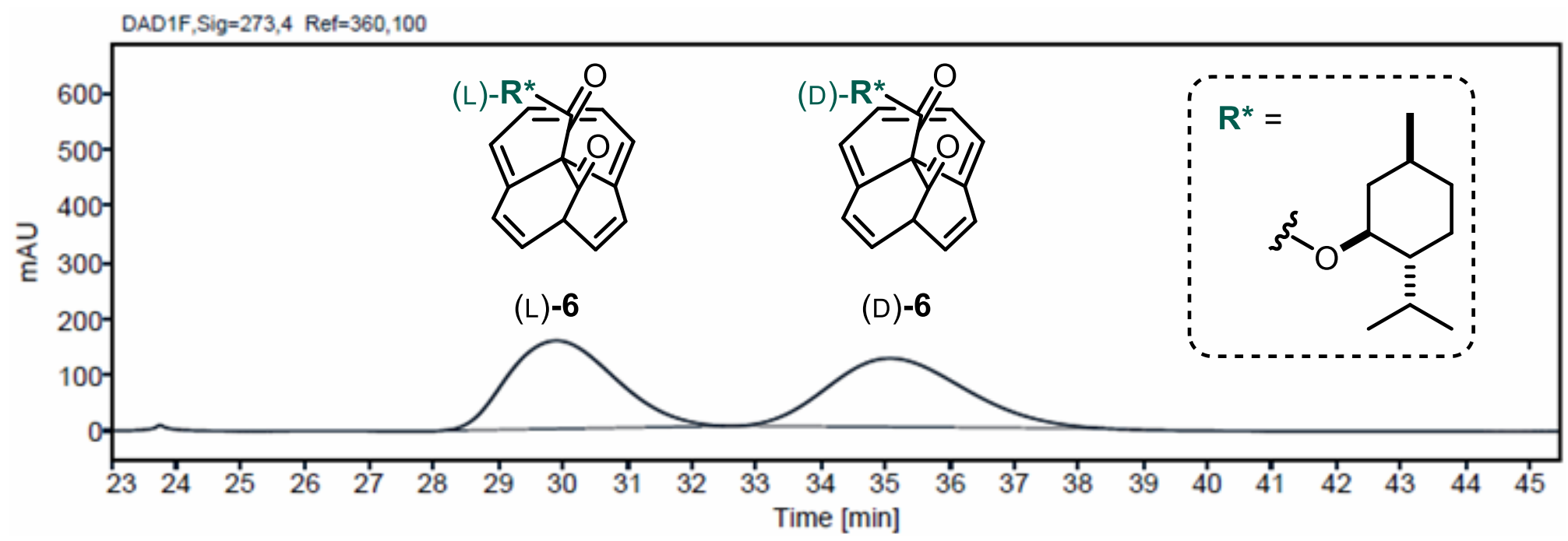

Signal:	DAD1F,Sig=273,4 Ref=360,100			
**Name**	**RT [min]**	**RF**	**Area**	**Peak Area Percent**
peak@29.889min	29.89		18153.606	50.18
peak@35.065min	35.06		18025.849	49.82

Figure S12. HPLC chromatogram of mixed samples of homoannulene (L)- and (D)-**6**. CHIRALPAK OJ-H, (*n*-heptane/*iso*-propanol = 90:10, flow rate: 0.3 mL · min^{-1},

When mixing the irradiated samples of (L)- and (D)-**6** after irradiation four signals for *iso*-homoannulenes **7** and one broad signal for homoannulene **6** was detected in HPLC (Figure S13 and Scheme S4 for the stereochemical analysis). As displayed in Scheme S3, the detected signals can be assigned to the (L)- and respective (D)-menthyl substituted (*iso*-)homoannulene compounds (L)-**7**/(D)-**7**. The detected signal at a retention time of t = 15.7 and 18.7 min was assigned to (L)-menthyl substituted *iso*-homoannulenes *(*L*,S)*-**7** and (L,R)-**7**. The detected signals at t = 15.9 and 17.9 min were assigned to (D)-menthyl substituted *iso*-homoannulenes *(*D*,S)*-**7** and *(*D*,R)*-**7**.

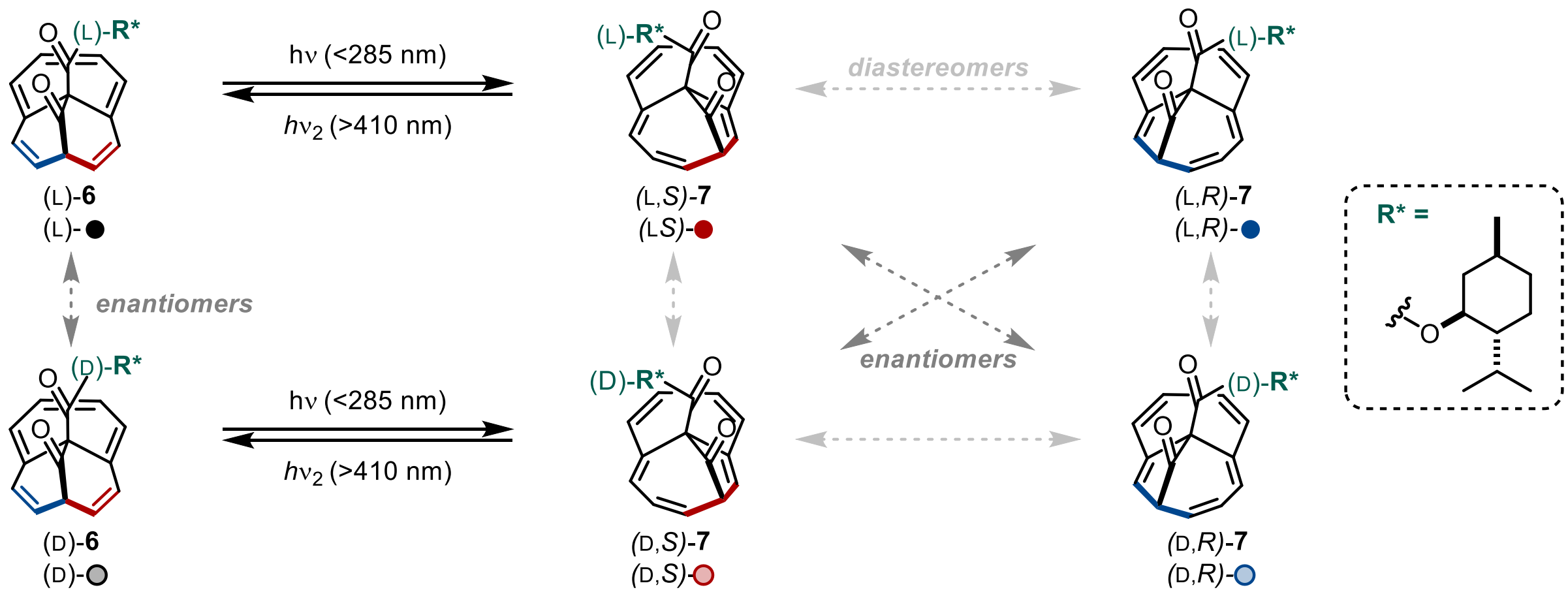

Scheme S4. Stereochemical analysis of chiral homoannuelene **7**.

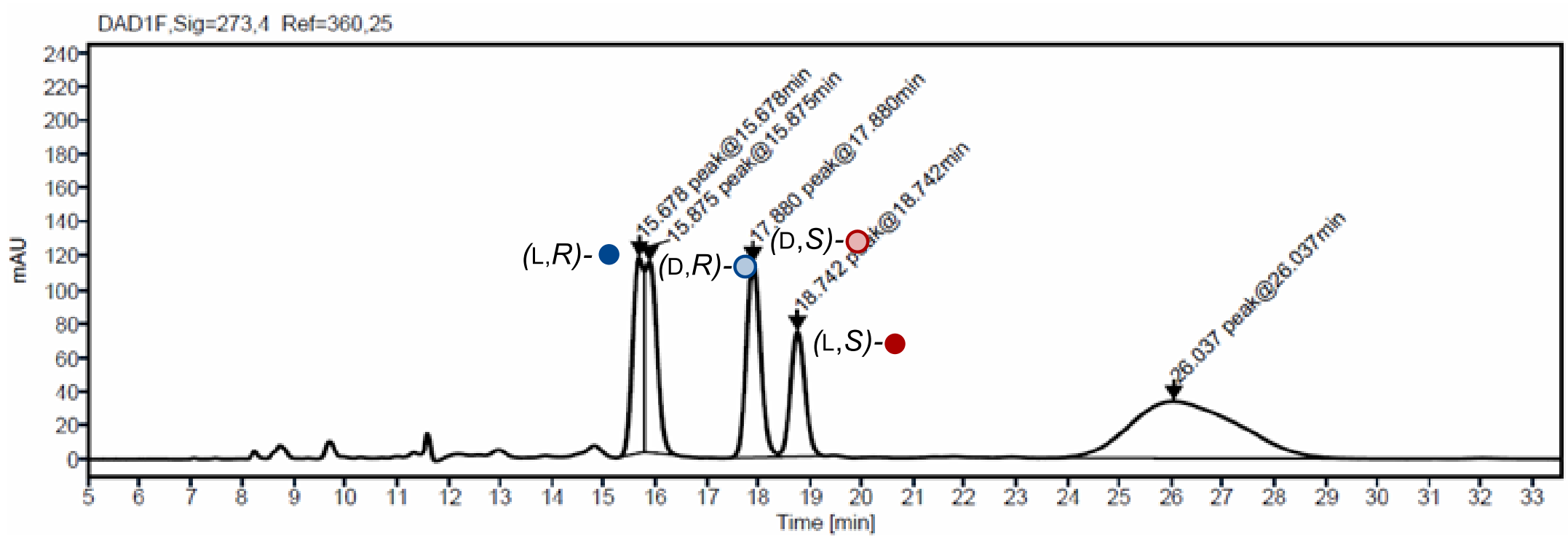

Signal: DAD1F,Sig=273,4 Ref=360,25

Name	RT [min]	Area	Peak Area Percent
peak@15.678min	15.68	1706.679	14.19
peak@15.875min	15.88	1779.287	14.79
peak@17.880min	17.88	2204.267	18.32
peak@18.742min	18.74	1439.536	11.96
peak@26.037min	26.04	4901.606	40.74

Figure S13 HPLC chromatogram of mixed sample of irradiated solution of (L)- and (D)-**6**. CHIRALPAK AD-H, *n*-heptane/*i*PrOH = 90:10, flow rate: 0.4 mL · min^{-1}.

1.7.4.2 Analysis of d.r. by NMR experiments

The isolated *iso*-homoannulenes (L)-**7** (which were obtained from "*photoswitching in large scale*" of homoannulene (L)-**6**) were dissolved in THF-d_8 and measured in ^{1}H NMR spectroscopy (Figure S14). Signals at δ = 4.41 and 4.44 ppm were assigned to hydrogen atoms H-4 of the diastereomeric *iso*-homoannulenes (L)-**7**. A ratio of the *iso*-homoannulenes (L,*R*)-**7** to (L,*S*)-**7** of d.r. = 56:44 was determined. As discussed in detail in Section 1.7.8 the signal with larger area was assigned to H-4 of *(*L*,R)*-**7**. This result is in agreement within the limits of measurement accuracy with the aforementioned ratio of *iso*-homoannulenes (L)-**7** detected by HPLC analysis on a chiral stationary phase (see Figure S11).

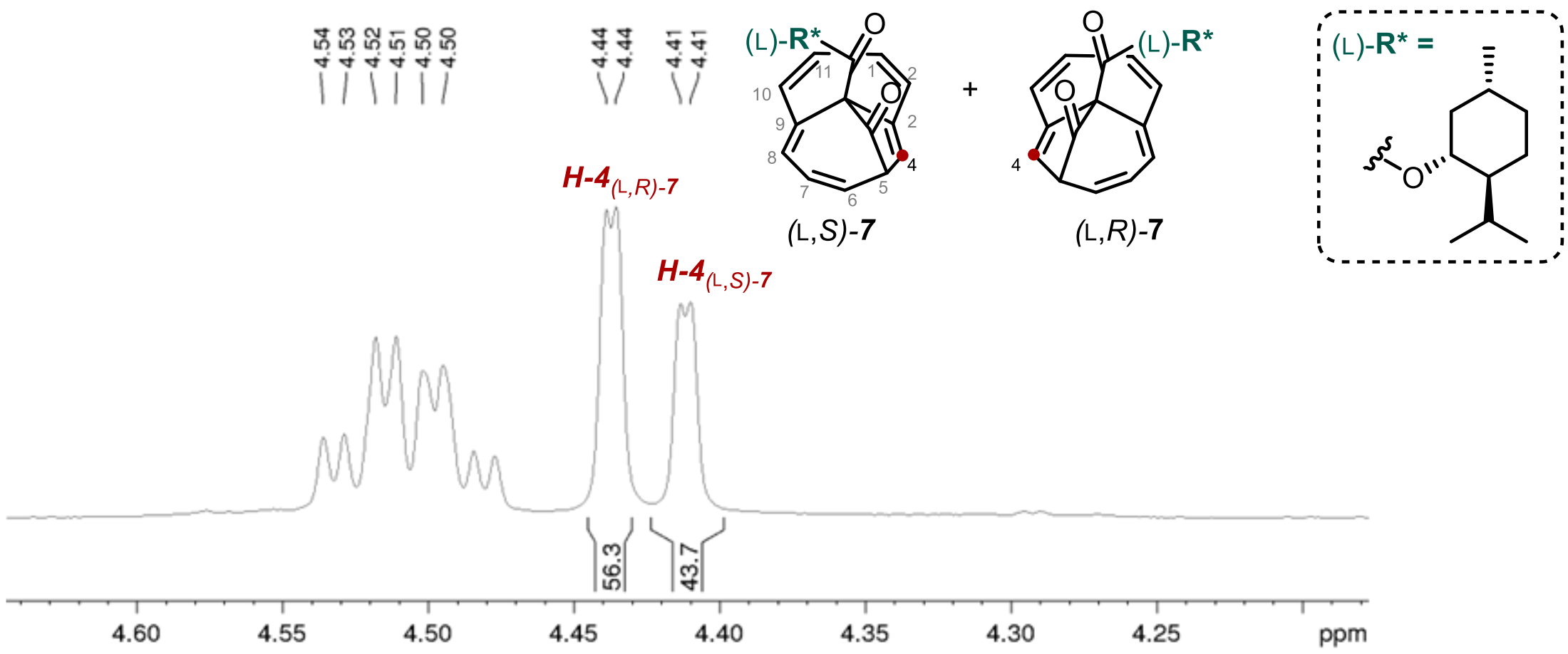

Figure S14 Section of ^{1}H NMR spectrum of isolated (L)-**7**. The spectrum was measured in THF-d_8 at 600 MHz.

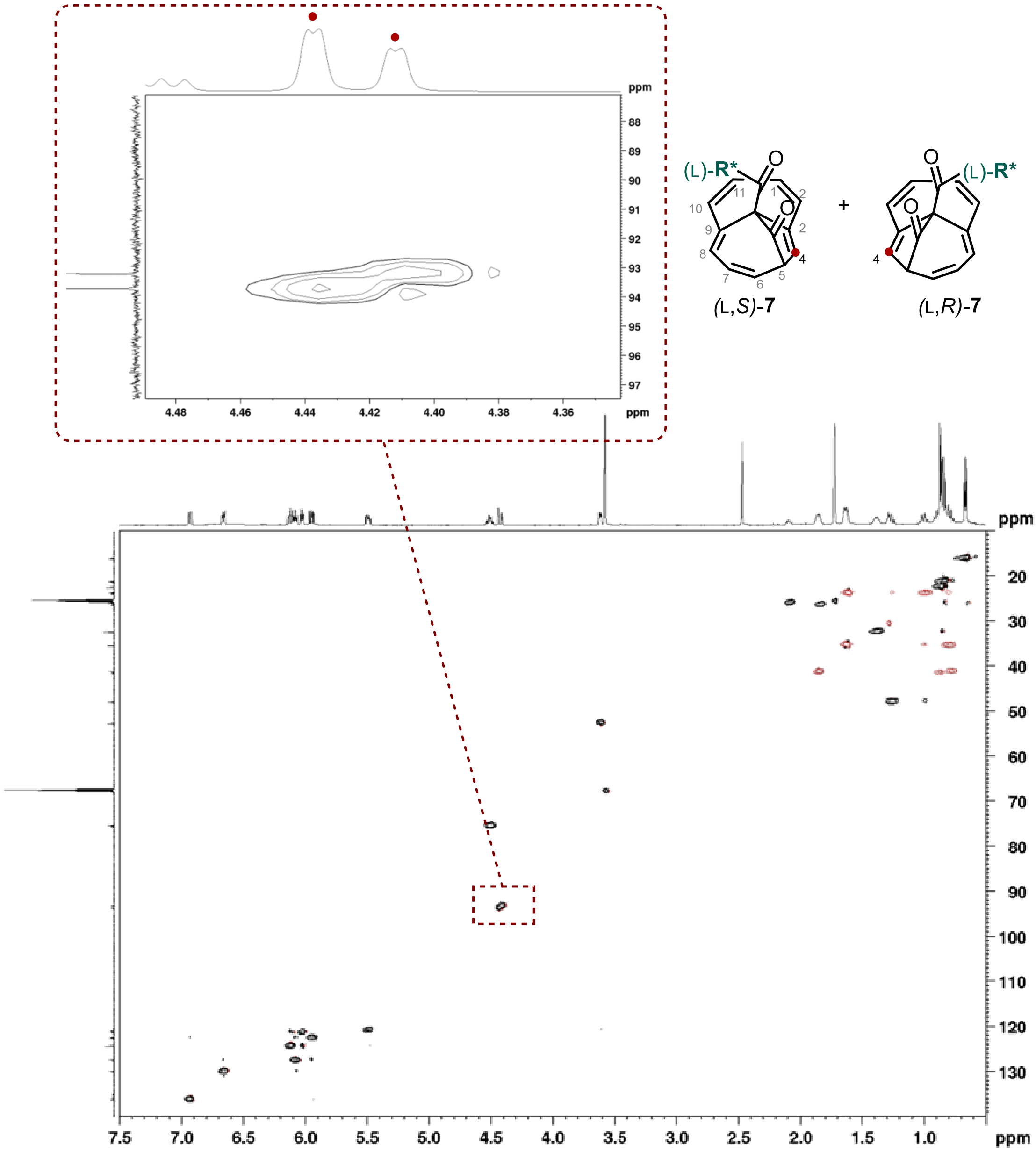

Figure S15. Section of ^{1}H, ^{13}C HSQC spectrum of isolated *(*L*,S)*-**7** and *(*L*,R)*-**7**. Measured in THF-d_8. Negative phase is colored in red.

^{1}H NMR spectra detected of irradiated samples of homoannulene esters (L)- and (D)-**6** after irradiation with 285 nm for 1 h at 0 °C (Scheme S5) display similar signal pattern (middle and bottom spectra, Scheme S5). In both irradiated samples the starting material **6** was detected, as expected by previously discussed HPLC chromatograms also displayed that the photochemical [1,11]-sigmatropic rearrangement does not proceed until full conversion of homoannulene **6** (compare Figure S11). It is shown that the same diastereomer is formed upon irradiation of the enantiomeric homoannulene esters **6**. Furthermore, the ratio of *iso*-homoannulenes **7** is comparable for (L)-and (D)-substituted compounds (d.r. = 56:44).

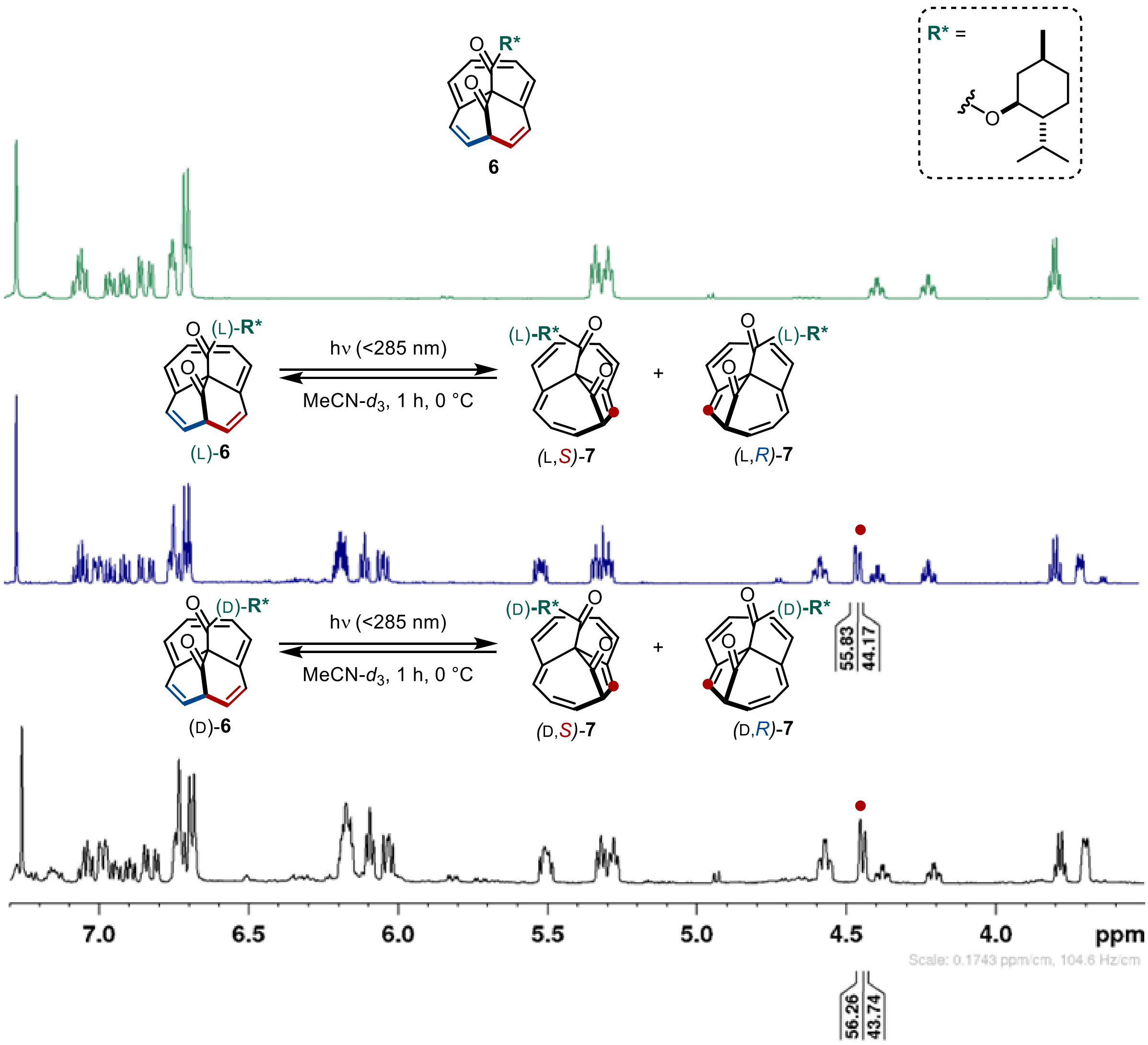

Scheme S5. Top: ^{1}H NMR spectrum of homoannulene (L)-**6**. Middle: ^{1}H NMR spectrum of (L)-**6**, (L)-**7** at $PSS_{285\ nm}$, Bottom: ^{1}H NMR spectrum of (D)-**6**, (D)-**7** at $PSS_{285\ nm}$. NMR spectra were measured in $CDCl_3$, 600 MHz.

1.7.4.3 PSS and d.r. by using LEDs

To investigate whether photoswitching of homoannulene (L)-**6** using mounted LEDs (set-up: "*photoswitching in small scale*") affords results comparable to those in the "*photoswitching in large scale*" (see previous sections), the ratio of homoannulene (L)-**6** to *iso*-homoannulenes (L)-**7** at the respective PSS and the diastereomeric ratio of the *iso*-homoannulenes (L,*R*)-/((L,S)-**7** were determined. The same wavelength as in the UV/vis absorption experiments were used, namely λ = 280 nm for the forward switching and λ = 430 nm for the backward switching process.

For the experiment, homoannulene (L)-**6** was dissolved in MeCN ($c = 9.84 \cdot 10^{-3}$ mol · L^{-1}), irradiated and analyzed by HPLC. One representative HPLC chromatogram is shown in Figure

S17; additional HPLC chromatograms are provided in the "Spectral and Chromatographic Appendix". First, homoannulene (L)-**6** was irradiated at λ = 280 nm and the ratio of homoannulene (L)-**6** to *iso*-homoannulene (L)-**7** was determined as a function of irradiation time (Figure S16, orange). After 3 h of irradiation, the $PSS_{280\ nm}$ was reached and a ratio of (L)-**6**/(L)-**7** = 58:42 was determined. This value is faintly decreased compared to the ratio detected when using the Hg high pressure mercury lamp equipped with a cutoff filter of λ = 285 nm ($d.r._{(L)-6/(L)-7}$ = 41:59 at $PSS_{285\ nm}$, compare section 1.7.4.1). In addition, the diastereomeric ratio of *iso*-homoannulenes (L,R)-**7** to (L,S)-**7** was monitored as a function of irradiation time until $PSS_{280\ nm}$ was reached (Figure S16, blue). The ratio remained essentially constant throughout the irradiation, ranging between $d.r._{(L,R)-7/(L,S)-7}$ = 56:44 to 55:45.

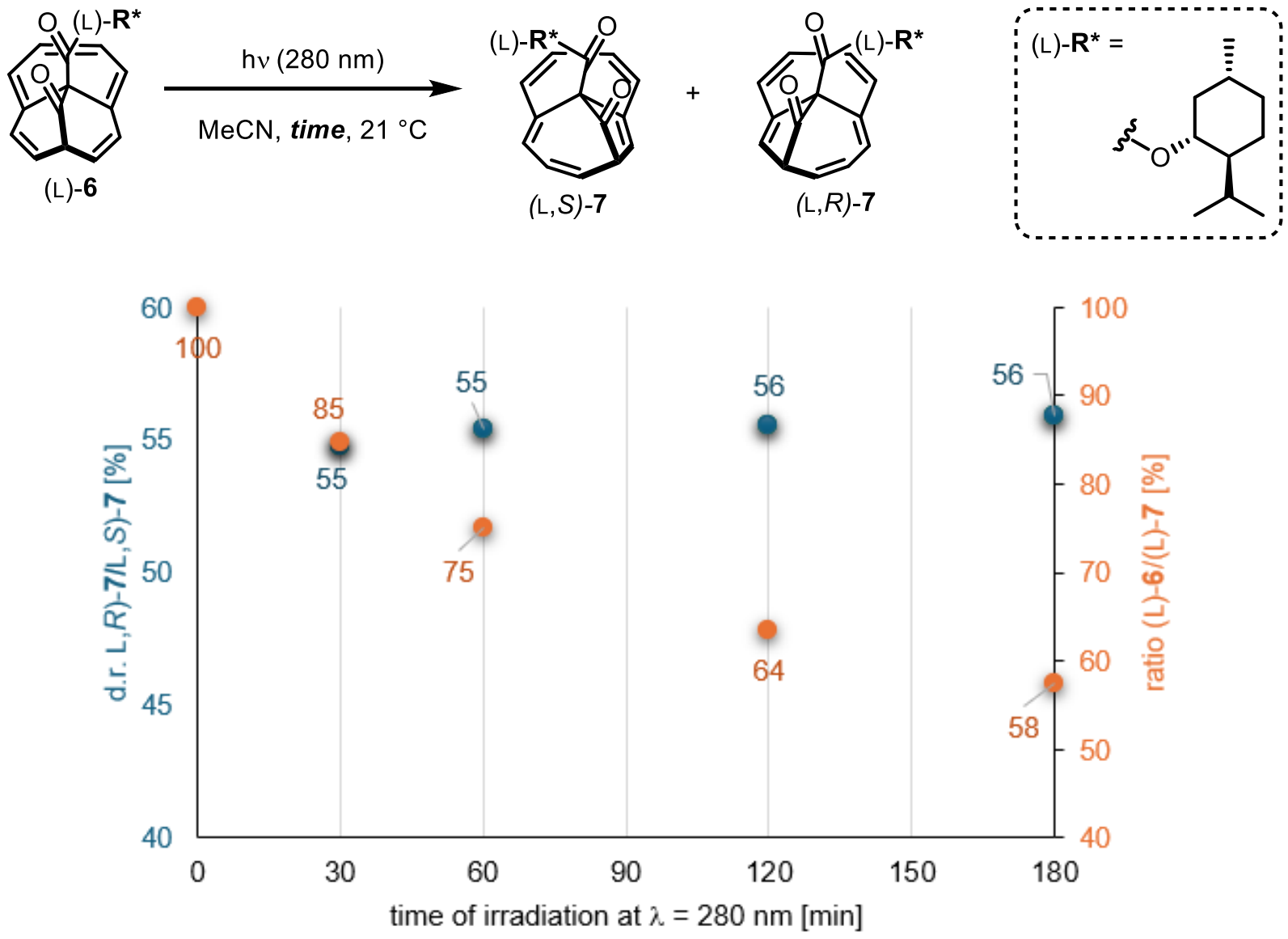

Figure S16. d.r. of *iso*homoannulenes **7** (blue) and ratio of homoannulene **6** to *iso*-homoannulenes **7** (orange) over time when using an LED at λ = 280 nm.

One representative HPLC chromatogram homoannulene (L)-**6** after 180 min of irradiation is shown in Figure S17 (for further HPLC data, see the "Spectral and Chromatographic Appendix"). Homoannulene (L)-**6** was detected at a retention time of t = 26.9 min, the formed *iso*-homoannulenes (L)-**7** at t = 15.9 and 18.7 min, respectively (see Figure S17). Additionally, the reference compound naphthalene was detected at a retention time t = 8.4 min and was used to quantify the photodegradation of compounds **6** and **7** (for details see section 1.7.6.1).

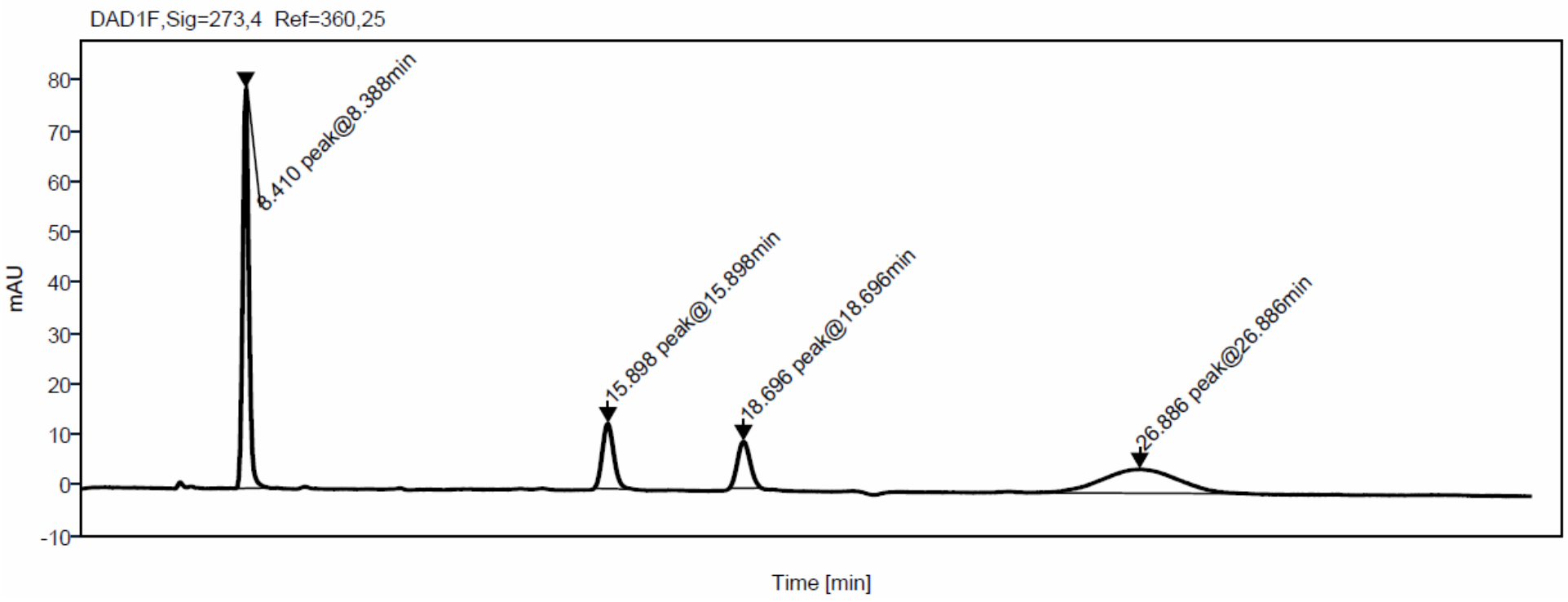

Signal: DAD1F,Sig=273,4 Ref=360,25

Name	RT [min]	Area	Peak Area Percent
peak@8.388min	8.41	684.237	43.43
peak@15.898min	15.90	211.351	13.41
peak@18.696min	18.70	166.953	10.60
peak@26.886min	26.89	512.991	32.56

Figure S17. HPLC chromatogram of irradiated homoannulene (L)-**6** with λ = 280 nm for 180 min. CHIRALPAK AD-H, *n*-heptane/*i*PrOH = 90:10, flow rate: 0.4 mL · min^{-1}.

Additionally, the backward switching process was monitored by HPLC analysis. A sample of homoannulene (L)-**6** and *iso*-homoannulenes (L)-**7** obtained at $PSS_{280\ nm}$ was irradiated with an LED at λ = 430 nm. The ratio of (L)-**6** to (L)-**7** was determined as a function of irradiation time (Figure S18, orange, HPLC chromatograms are provided in the "Spectral and Chromatographic Appendix"). After 1 h of irradiation at λ = 430 nm the *iso*-homoannulenes (L)-**7** were fully converted to homoannulene (L)-**6**. Furthermore, the d.r. of *iso*-homoannulenes (L)-**7** was monitored as a function of irradiation time (Figure S18, blue). Within the experimental error of the HPLC analysis, the d.r. of the *iso*-homoannulenes (L)-**7** remained constant throughout the irradiation.

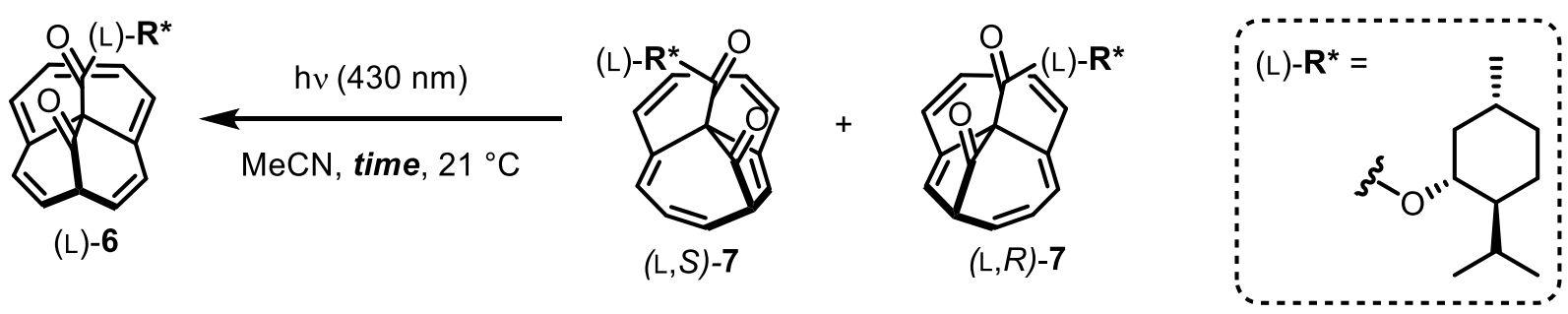

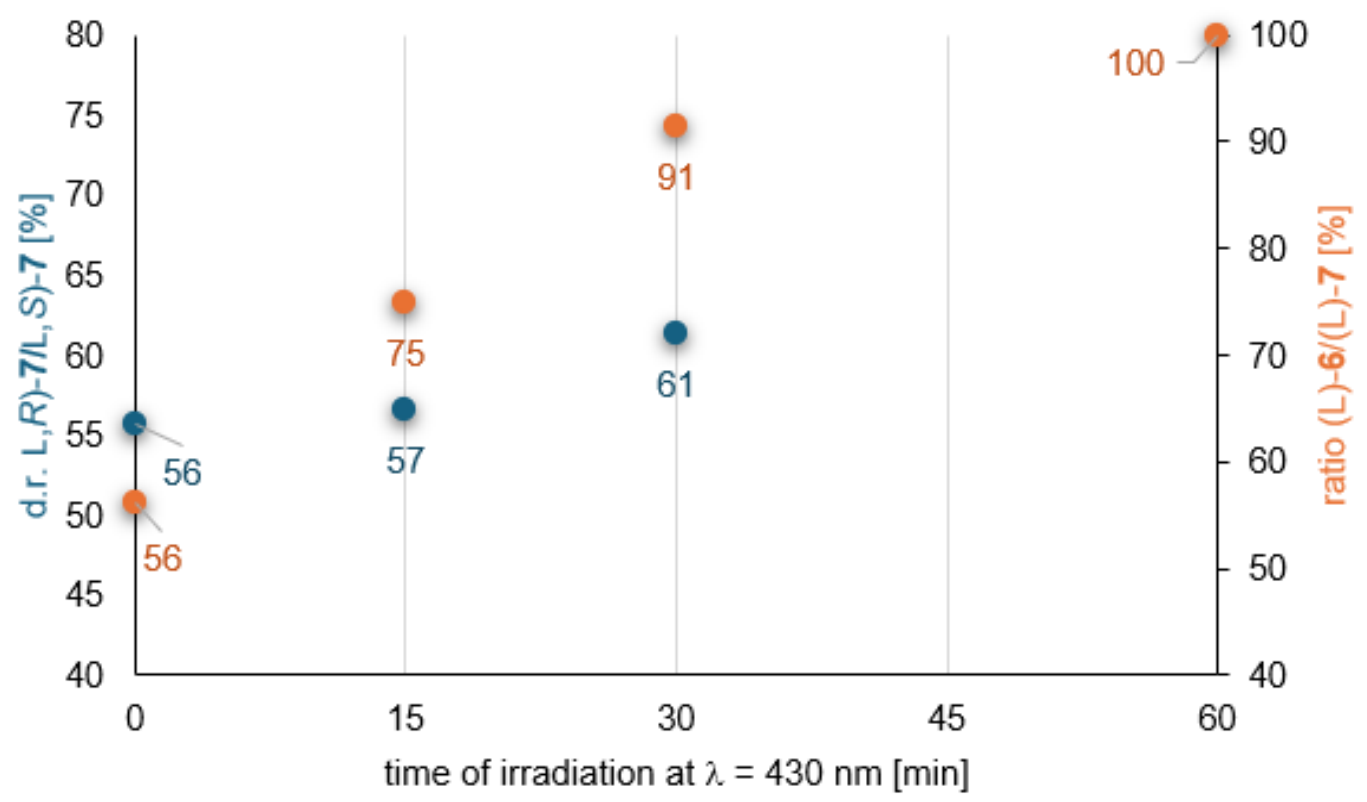

Figure S18. d.r. of *iso*homoannulenes **7** (blue) and ratio of homoannulene **6** to *iso*-homoannulene **7** (orange) over time when using an LED at λ = 430 nm.

1.7.5 Thermal stability experiment

It was investigated whether the photoswitching of the *iso*-homoannulenes **7** can be initiated thermally. For that homoannulene ester (L)-**6** was dissolved in MeCN ($c = 0.0588\ mol \cdot L^{-1}$) and irradiated for 1 h at 0 °C with a Hg lamp equipped with an 285 nm cutoff filter until $PSS_{285\ nm}$ was reached (set-up: "*photoswitching in large scale*"). An HPLC chromatogram of the mixture of homoannulene ester (L)-**6** and *iso*-homoannulene esters (L)-**7** was measured (Figure S19, top) on a CHIRALPAK OJ-H analytical column (*n*-heptane/*i*PrOH = 90:10, flow rate: $0.5\ mL \cdot min^{-1}$). At $PSS_{285\ nm}$ a ratio of homoannulene (L)-**6** to *iso*-homoannulenes (L)-**7** of (L)-**6**/(L)-**7** = 35:65 was detected. The diastereomeric ratio of *(*L,*S)*-**7** and *(*L,*R)*-**7** was determined to be d.r. = 56:44. The irradiated mixture of homoannulene ester (L)-**6** and *iso*-homoannulene esters (L)-**7** was heated to 60 °C and hold at that temperature for 16 h. The sample was analyzed by HPLC (Figure S19, bottom) and a ratio of (L)-**6**/(L)-**7** = 35:65 was detected. The ratios of homoannulene (L)-**6** to *iso*-homoannulenes (L)-**7** was not altered upon the period of heating. This indicates that a switch to homoannulene ester (L)-**6** cannot be initiated thermally. Furthermore, the diastereomeric ratio of *iso*-homoannulenes (L)-**7** remained at d.r. = 56:44 after the heating, indicating that no isomerization takes place under the reaction conditions used.

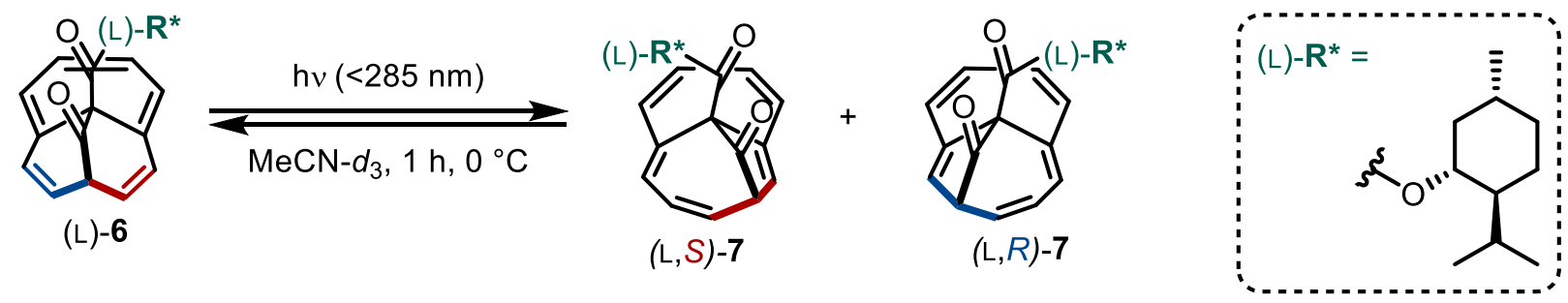

PSS$_{285nm}$

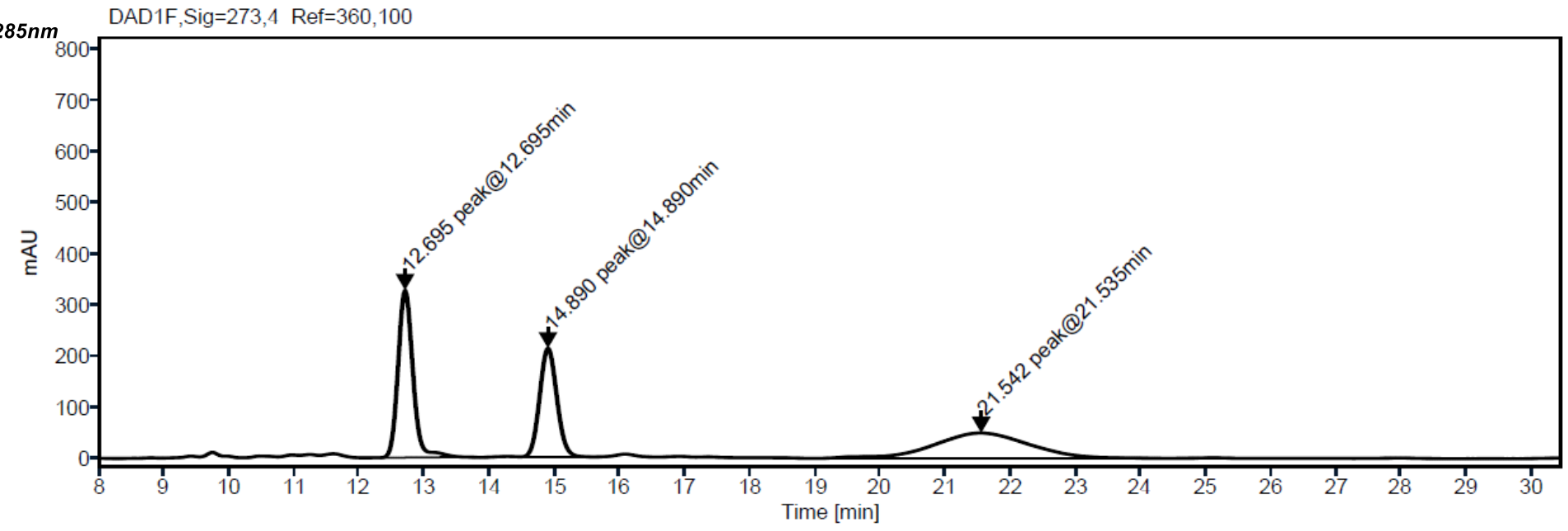

**Signal:**	DAD1F,Sig=273,4 Ref=360,100		
**Name**	**RT [min]**	**Area**	**Peak Area Percent**
peak@12.695min	12.70	5195.928	36.89
peak@14.890min	14.89	3902.342	27.71
peak@21.535min	21.54	4984.867	35.40

After heating to 60 °C for 16 h:

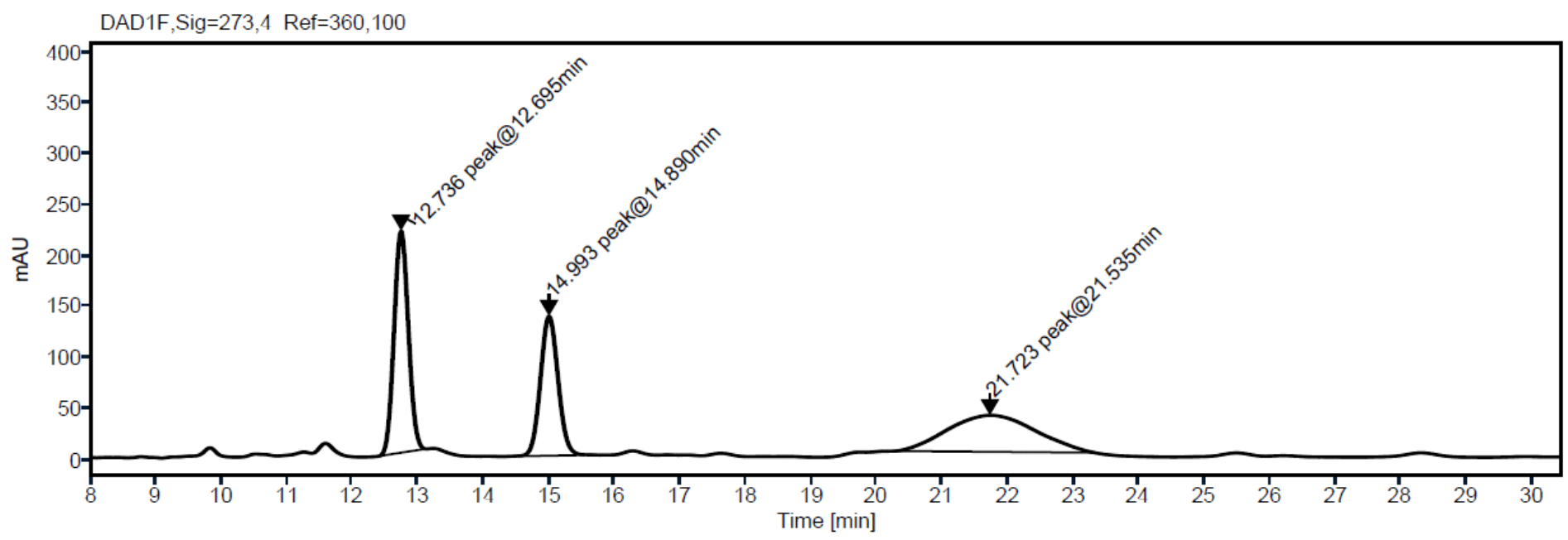

**Signal:**	DAD1F,Sig=273,4 Ref=360,100		
**Name**	**RT [min]**	**Area**	**Peak Area Percent**
peak@12.695min	12.74	3299.607	35.80
peak@14.890min	14.99	2558.758	27.76
peak@21.535min	21.72	3359.178	36.44

Figure S19. Top: HPLC chromatogram after irradiation of homoannulene ester (L)-**6** with light of 285 nm for 1 h. Bottom: HPLC chromatogram after heating the mixture of homoannulene ester (L)-**6** and *iso*-homoannulene esters (L)-**7** to 60 °C for 16 h. CHIRALPAK OJ-H, *n*-heptane/*i*PrOH = 90:10, flow rate: 0.5 mL · min^{-1}.

1.7.6 Photoswitch-cycling monitoring and photodegradation

To display multiple rounds of photoswitching of homoannulene (L)-**6** the compound was diluted in MeCN and irradiated using an Hg lamp equipped with a λ = 285 nm cutoff filter and λ = 410 nm LED panel equipped with a fan cooler (set-up: "*large scale photoswitching*") or by using mounted LEDs of λ = 280 and 430 nm to complete one cycle of photoswitching (set-up: "*small scale photoswitching*") to investigate and compare the photodegradation by NMR and HPLC analysis. HPLC analysis on a chiral stationary phase (CHIRALPAK AD-H, *n*-heptane/*i*PrOH = 90:10, flow rate: 0.4 mL · min^{-1}) was employed to investigate multiple cycles of photoswitching in regards of the diastereomeric ratio of the *iso*-homoannulenes (L,*S*-**7** to (L,*R*)-**7** as well as the ratio of homoannulene (L)-**6** to *iso*-homoannulenes (L)-**7**.

1.7.6.1 Photoswitch-cycling using LEDs ("*small scale photoswitching*")

Homoannulene (L)-**6** was dissolved in MeCN (c = $9.84 \cdot 10^{-3}$ mol · L^{-1}) and an aliquot of the resulting solution was taken for HPLC analysis (HPLC chromatograms are provided in the "Spectral and Chromatographic Appendix"). Prior to HPLC injection, a defined amount of a naphthalene standard solution (c = 0.0285 mol · L^{-1}) was added to the sample as an internal standard.

A calibration curve for naphthalene was established to verify the linearity and reproducibility of its HPLC response over the concentration range used (Figure S20, HPLC chromatograms are provided in the "Spectral and Chromatographic Appendix"). Naphthalene was subsequently used as an internal standard for the normalization of the HPLC signal areas of homoannulene **6** and *iso*-homoannulenes **7**, enabling the relative changes in photoswitch concentration upon irradiation to be quantified and the photofatigue to be assessed.

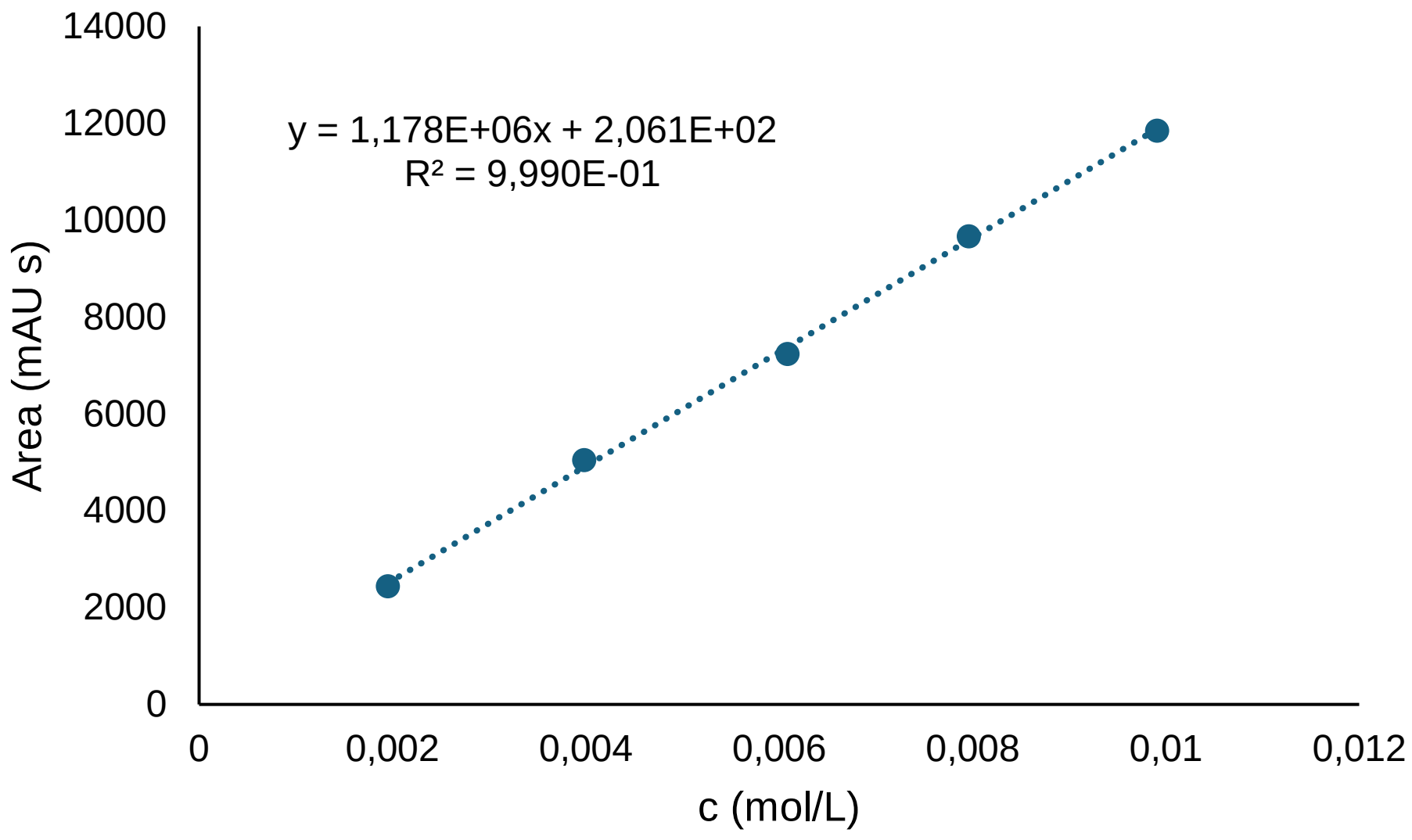

Figure S20. Calibration curve for the internal standard naphthalene used in HPLC analysis.

One photoswitching cycle consisted of 3 h of irradiation at λ = 280 nm followed by 1 h of irradiation at λ = 430 nm (set-up: "*photoswitching in small scale*"). At $PSS_{280\ nm}$ the ratio of homoannulene (L)-**6** to *iso*-homoannulenes (L)-**7** remained constant within the error of HPLC analysis at (L)-**6**/(L)-**7** ≈ 60:40 over the investigated cycles (see Figure S21). Likewise, the ratio of the two *iso*-homoannulenes (L,*R*)-**7** and (L,*S*)-**7** remained unchanged within the experimental error with (L,*R*)-**7**/(L,*S*)-**7** ≈ 56:44. Due to the significant photodegradation observed (see Figure S22) the uncertainty of the HPLC analysis increased for the fourth photoswitching cycle. This is attributed to the low concentrations of homoannulene **6** and *iso*-homoannulenes **7** detected by HPLC (see chromatograms in the "Spectral and Chromatographic Appendix").

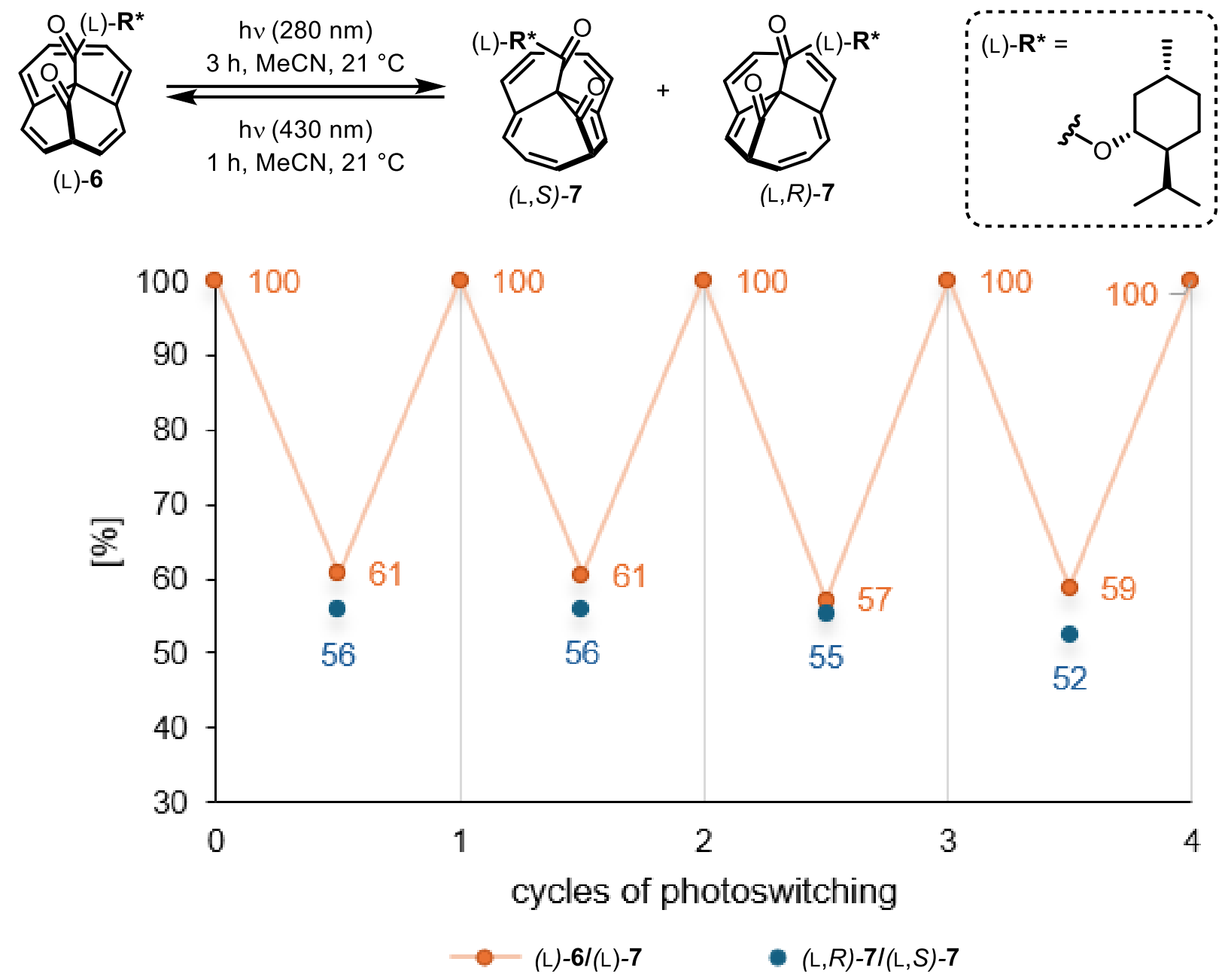

Figure S21. d.r. of (L,*R*)-**7** and (L,*S*)-**7** (orange) and ratio of (L)-**6** and *iso*-homoannulenes (L)-**7** (blue) at the respective PSS. One cycle of photoswitching consists of 3 h irradiation at λ = 280 nm and 1 h irradiation at λ = 430 nm.

Photodegradation over four cycles of photoswitching was monitored by HPLC (Figure S22, HPLC chromatograms are provided in the "Spectral and Chromatographic Appendix"). After four cycles, 93% photodegradation was observed. Notably, the backward switching process at λ = 430 nm resulted in substantially less photodegradation than the forward switching process at λ = 280 nm.

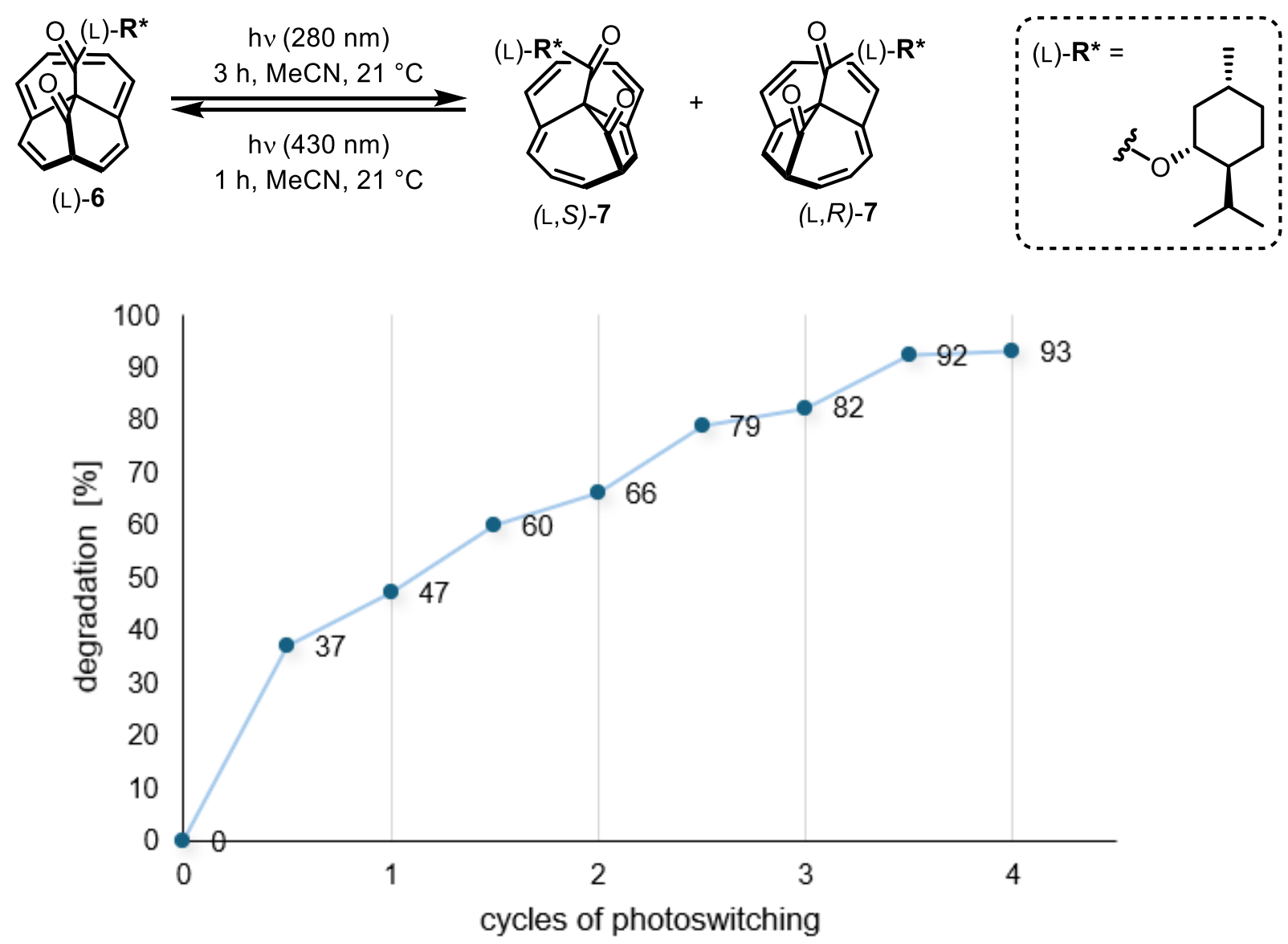

Figure S22. Degradation of homoannulene (L)-**6** and *iso*-homoannulenes (L)-**7** over four photoswitching cycles. One cycle of photoswitching consists of 3 h irradiation at λ = 280 nm and 1 h irradiation at λ = 430 nm.

As shown in the preceding Figure S22, photoswitching toward the *iso*-homoannulenes (L)-**7** results in considerably greater photofatigue than the backward switching process. For the first switching step, samples were taken at regular intervals until $PSS_{280\ nm}$ was reached to monitor the amount of photodegradation during this individual switching process over time (Figure S23, HPLC chromatograms are provided in the "Spectral and Chromatographic Appendix").

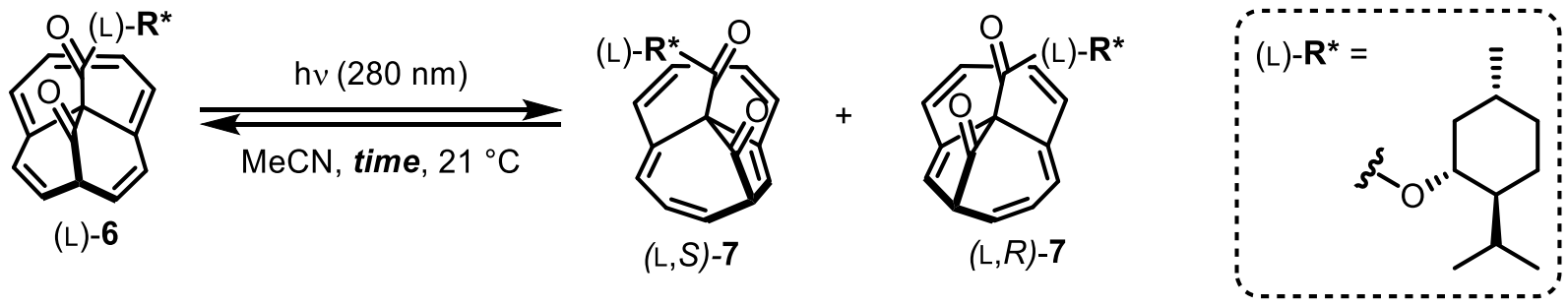

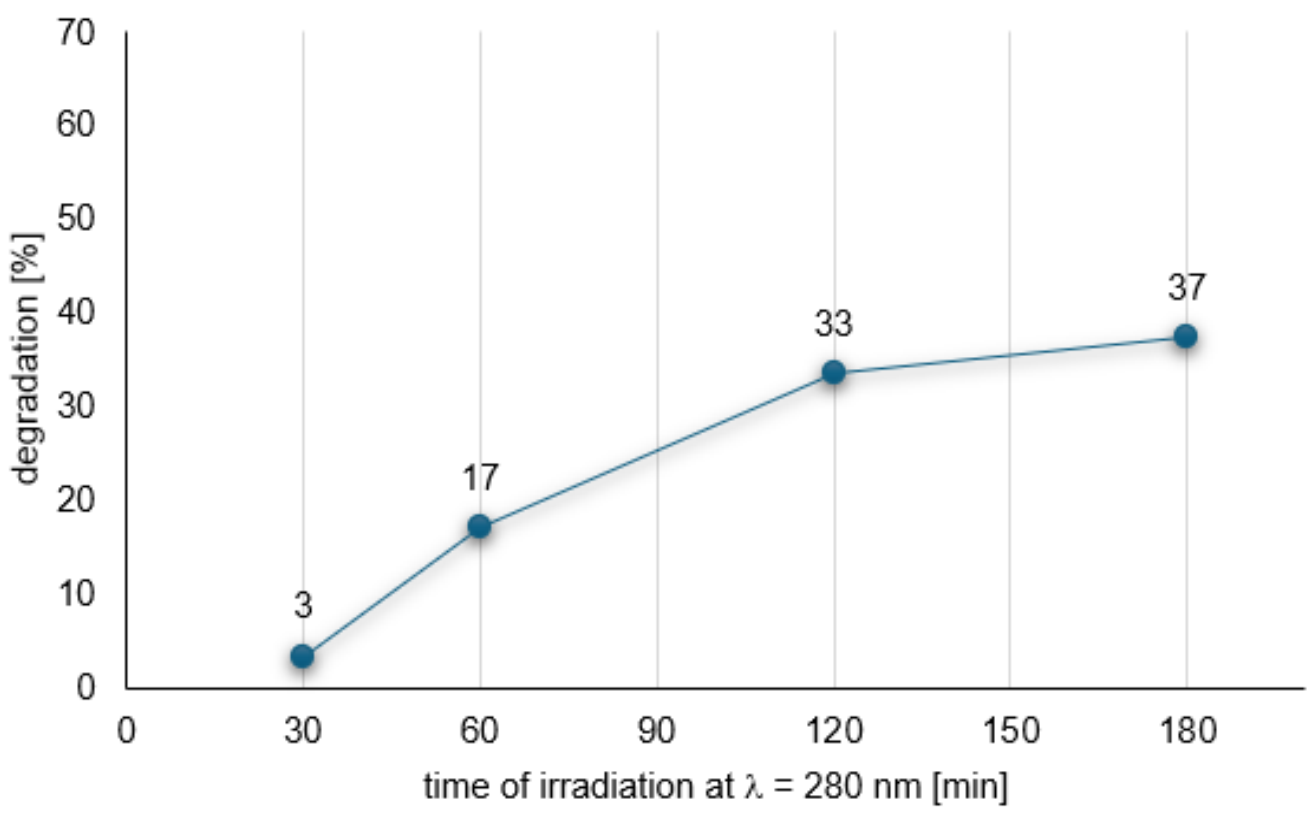

Figure S23. Degradation of homoannulene **6** and *iso*-homoannulene **7** as function of irradiation time using an LED at λ = 280 nm until $PSS_{280\ nm}$ was reached.

1.7.6.2 Photoswitch-cycling using Hg high-pressure lamp with cut-off filter and LED panel – "*large scale photoswitching*"

Photoswitch-cycling

Homoannulene menthyl ester (L)-**6** was dissolved in MeCN and subjected to HPLC analysis (Chromatogram 1, Figure S24). Homoannulene (L)-**6** was detected at a retention time of t = 26.3 min. After irradiation with λ = 285 nm for 1 h at 0 °C until PSS was reached, the mixture was subjected to HPLC (Chromatogram 2, Figure S24). *Iso*-homoannulenes (L)-**7** were detected at a retention time of t = 15.9 and 18.8 min. At $PSS_{285\ nm}$ the ratio of homoannulene (L)-**6** to *iso*-homoannulenes (L)-**7** was calculated to be (L)-**6**/(L)-**7** = 39:61. After irradiation with light of λ = 410 nm for 90 min at room temperature, the PSS was reached. At $PSS_{410\ nm}$ the ratio of homoannulene (L)-**6** to *iso*-homoannulenes (L)-**7** was calculated to be (L)-**6**/(L)-**7**= 81:19 (Chromatogram 3, Figure S24). Subsequent irradiation of the sample with λ = 285 nm for 1 h at 0 °C and analysis by HPLC resulted in a ratio at $PSS_{285\ nm}$ of homoannulene (L)-**6** to *iso*-homoannulenes (L)-**7** of (L)-**6**/(L)-**7**= 46:54 (Chromatogram 4,

Figure S24). Subsequently, the sample was irradiated with λ = 410 nm until $PSS_{410\ nm}$ was reached and analyzed by HPLC (Chromatogram 5, Figure S24). A ratio of homoannulene (L)-**6** to *iso*-homoannulene (L)-**7** of (L)-**6**/(L)-**7** = 82:18 was detected. The ratio of *iso*-homoannulenes (L)-**7** were calculated to be $PSS_{285\ nm,cycle1}$ = 55:45, $PSS_{410\ nm,\ cycle1}$ = 59:41, $PSS_{285\ nm,\ cycle\ 2}$ = 56:44 and $PSS_{410\ nm,\ cycle2}$ = 60:40.

Signals which were not assigned to homoannulene or *iso*-homoannulene compounds were detected in the HPLC after one round of photoswitching at a retention time of t = 8.6, 9.7 and 11.5 min (Figure S24) which increase in its intensity after every photoswitching step. These compounds could be assigned to photodegradation products, which could not get structurally elucidated yet. (Photodegradation processes were also indicated by the absence of isosbestic point in the UV/vis spectra recorded for the forward switching of homoannulene (L)-**6**, see section 1.7.3). The forward and backward switching process can be displayed as signals of *iso*-homoannulenes were detected after irradiation at λ = 285 nm and get diminished when irradiating with λ = 410 nm (compare chromatogram 2, 4 with 3, 5). After one cycle of photoswitching the ratio of *iso*-homoannulenes (L)-**7** was detected to be decreased compared to the first cycle of photoswitching due to the formation of photodegradation products, which could also absorb light of the wavelength used. The ratio of *iso*-homoannulenes *(*L,*S)*-**7** and *(*L,*R)*-**7** remain consistent upon irradiation with λ = 285 nm and 410 nm.

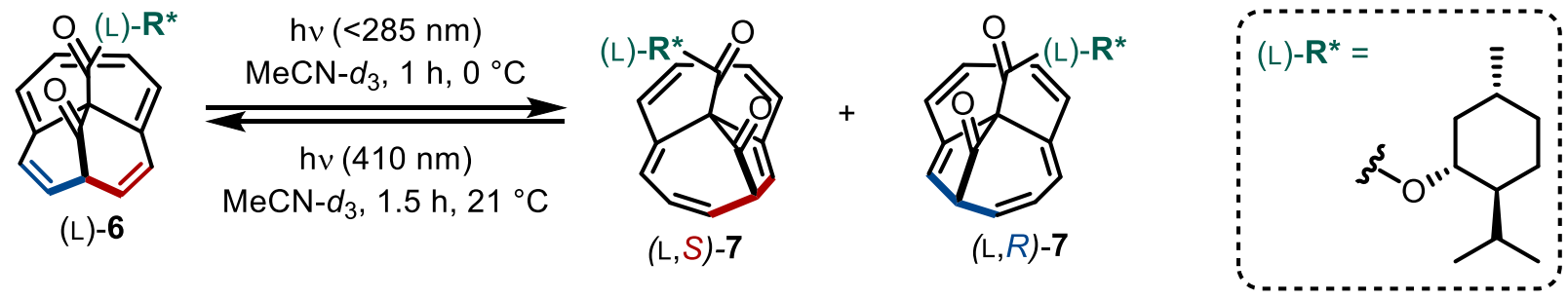

(L)-6

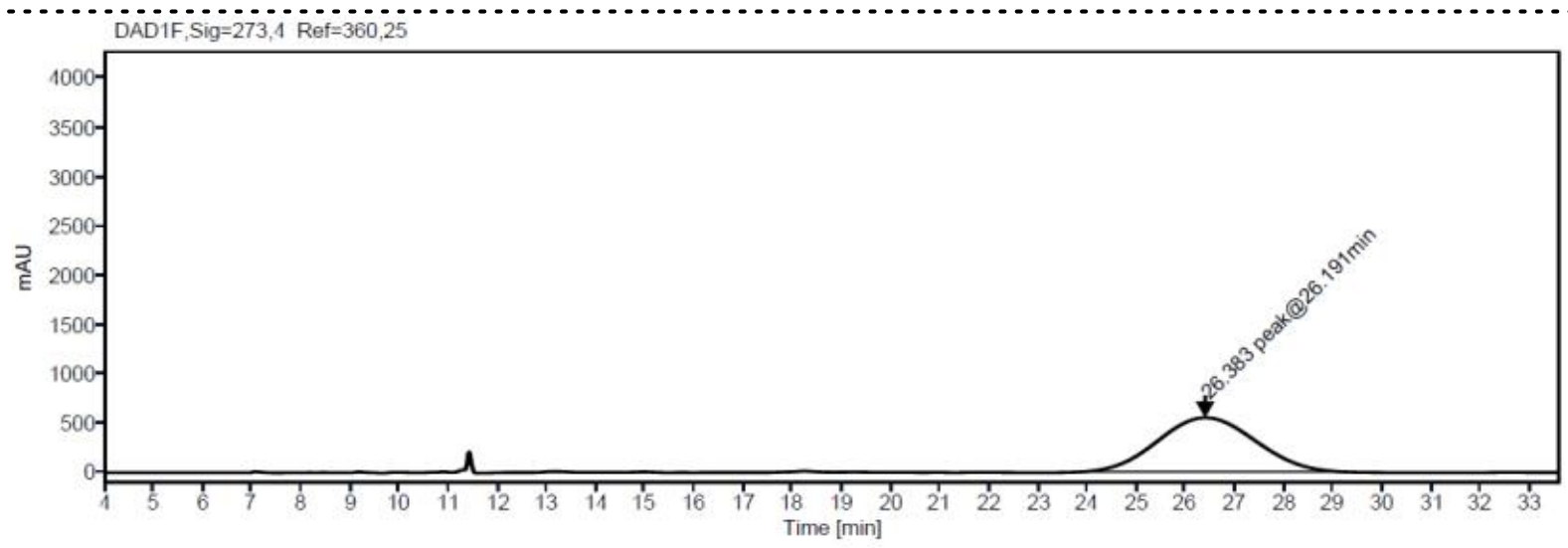

Signal:	DAD1F,Sig=273,4 Ref=360,25		
**Name**	**RT [min]**	**Area**	**Peak Area Percent**
peak@26.191min	26.38	75691.258	100.00

***PSS$_{285\ nm}$*, cycle 1**

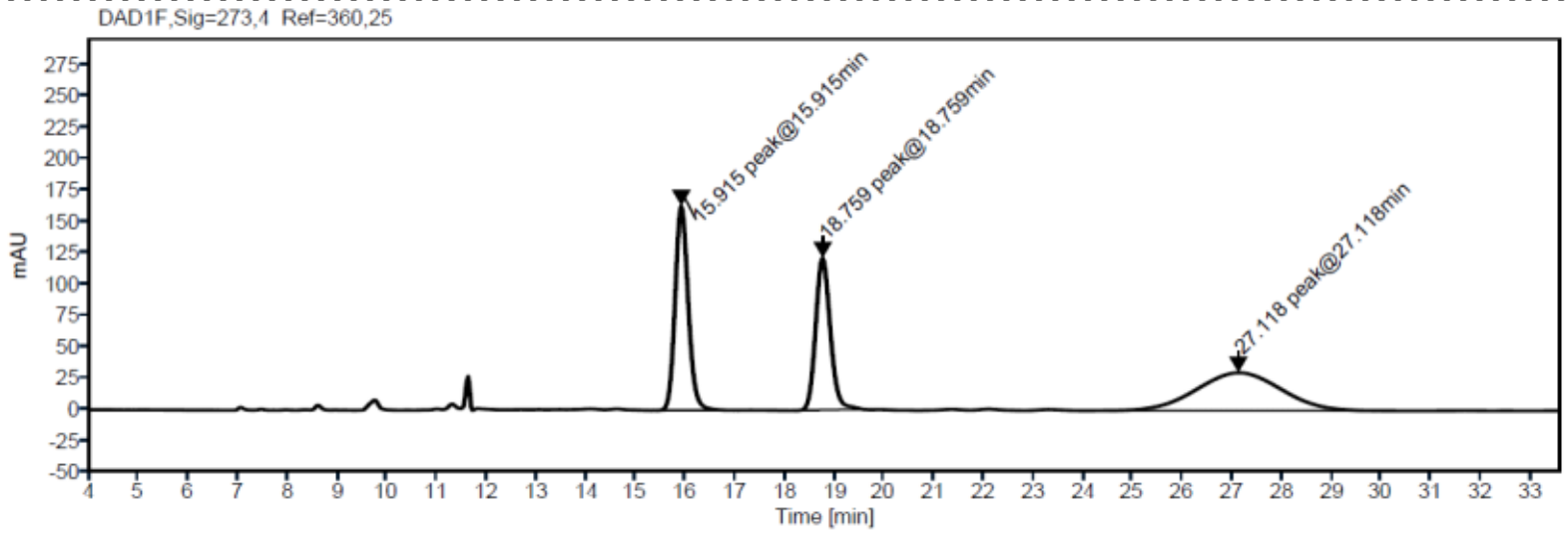

Signal:	DAD1F,Sig=273,4 Ref=360,25		
**Name**	**RT [min]**	**Area**	**Peak Area Percent**
peak@15.915min	15.92	2991.042	33.07
peak@18.759min	18.76	2495.295	27.59
peak@27.118min	27.12	3559.420	39.35

***PSS$_{410\ nm}$*, cycle 1**

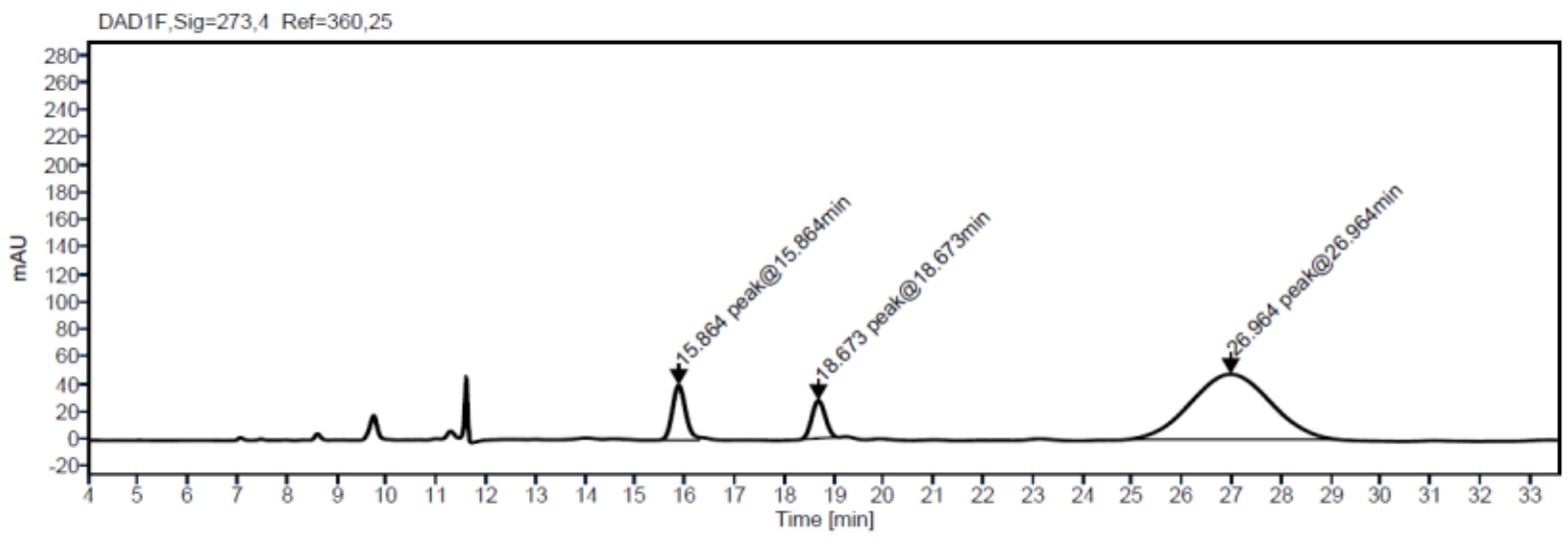

Signal:	DAD1F,Sig=273,4 Ref=360,25		
**Name**	**RT [min]**	**Area**	**Peak Area Percent**
peak@15.864min	15.86	735.396	11.22
peak@18.673min	18.67	515.223	7.86
peak@26.964min	26.96	5301.666	80.91

***PSS$_{285\ nm}$*, cycle 2**

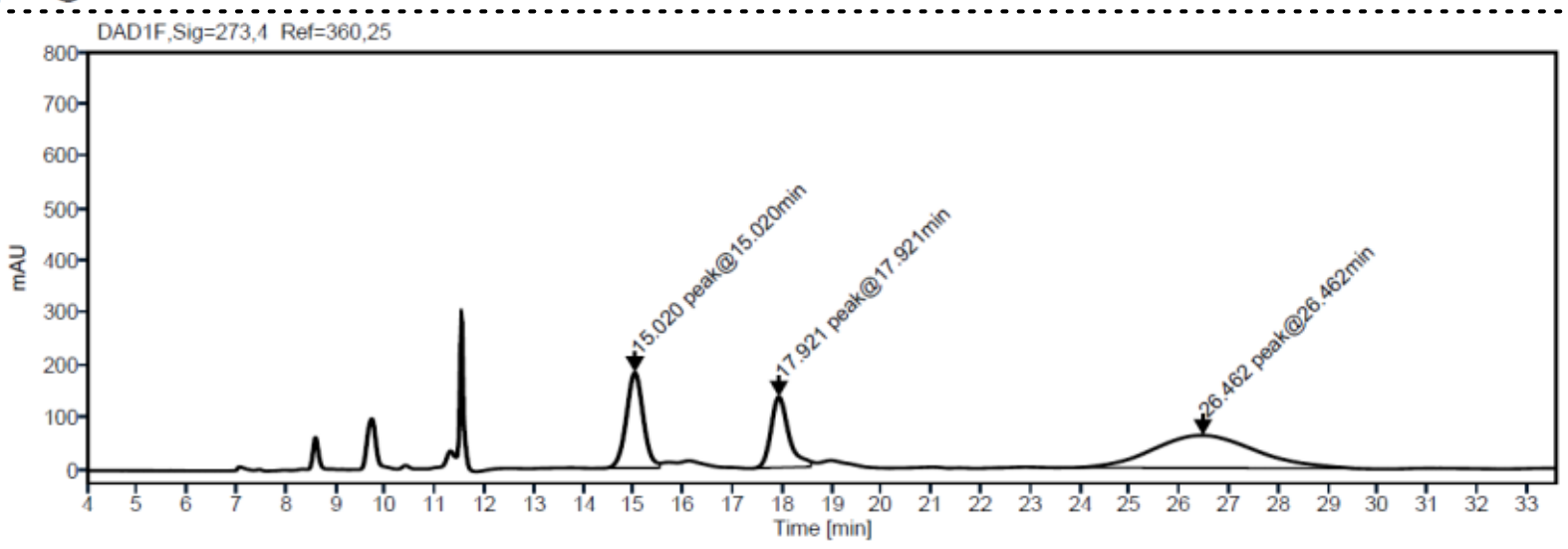

Signal:	DAD1F,Sig=273,4 Ref=360,25		
**Name**	**RT [min]**	**Area**	**Peak Area Percent**
peak@15.020min	15.02	4325.896	25.88
peak@17.921min	17.92	3383.707	20.25
peak@26.462min	26.46	9003.767	53.87

PSS$_{410\ nm}$, cycle 2

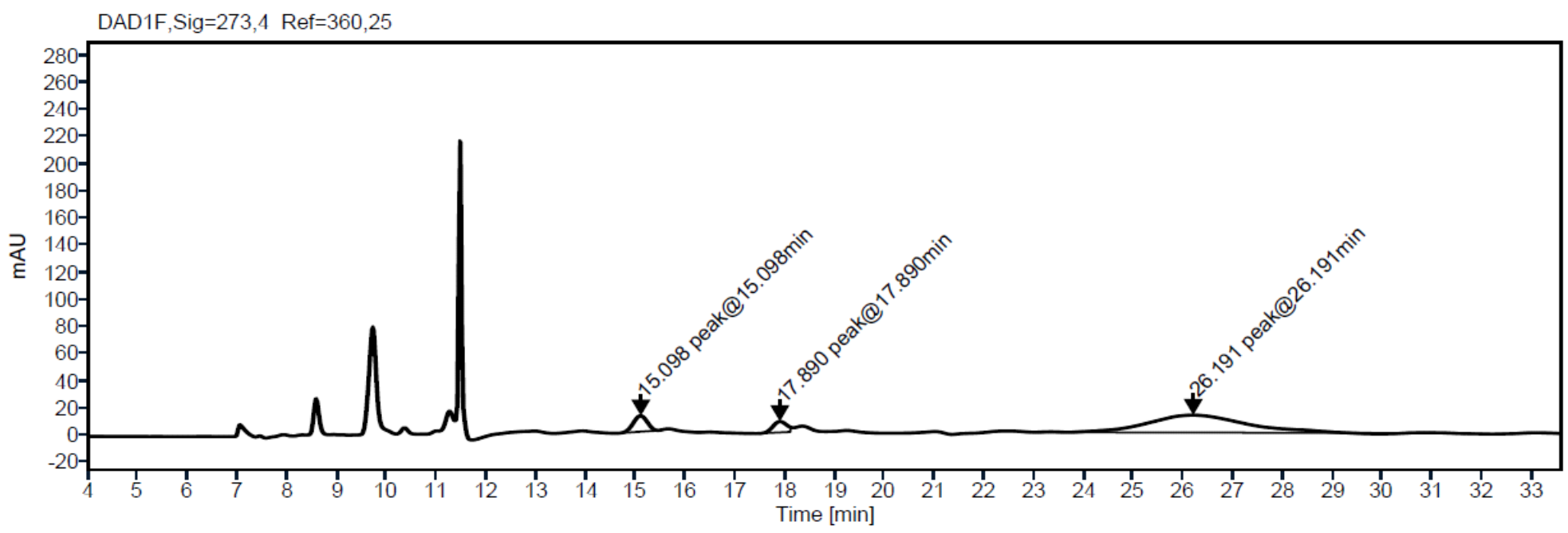

**Signal:**	DAD1F,Sig=273,4 Ref=360,25		
**Name**	**RT [min]**	**Area**	**Peak Area Percent**
peak@15.098min	15.10	228.668	10.78
peak@17.890min	17.89	154.187	7.27
peak@26.191min	26.19	1738.867	81.96

Figure S24. HPLC chromatograms at multiple cycles of photoswitching of homoannulene (L)-**6**. CHIRALPAK AD-H, *n*-heptane/*i*PrOH = 90:10, flow rate: 0.4 mL · min^{-1}.

*Photodegradation of homoannulene ester **6***

As discussed in the previous sections, during the photoswitching process of (*iso*)-homoannulene menthyl ester **6**/**7** photodegradation was observed.

To investigate the amount of photodegradation homoannulene menthyl ester (L)-**6** was dissolved in MeCN irradiated at λ = 285 nm (set-up: "*photoswitching in large scale*") and subsequently irradiated at λ = 410 nm for 90 min. A defined amount of the reference compound was added to each sample before subjecting the sample to HPLC analysis (Figure S25). After irradiation with 285 nm a d.r. of the two formed *iso*-homoannulenes (L,R)-/(L,S)-**7** of (L,R)-**7**/(L,S)-7 = 56:44 and a ratio of homoannulene (L)-**6** to *iso*homoannulenes (L)-**7** of **6**/**7** = 50:50 was detected. 21% of photodegradation was determined for the first photoswitching process. After irradiation with λ = 410 nm for 90 min a d.r. of the remaining *iso*-homoannulenes (L,R)-/(L,S)-**7** of (L,R)-**7**/(L,S)-7 = 56:44 and a ratio of homoannulene (L)-**6** to *iso*-homoannulenes (L)-**7** of **6**/**7** = 83:17 was detected. After one cycle of photoswitching 41% of photodegradation were determined.

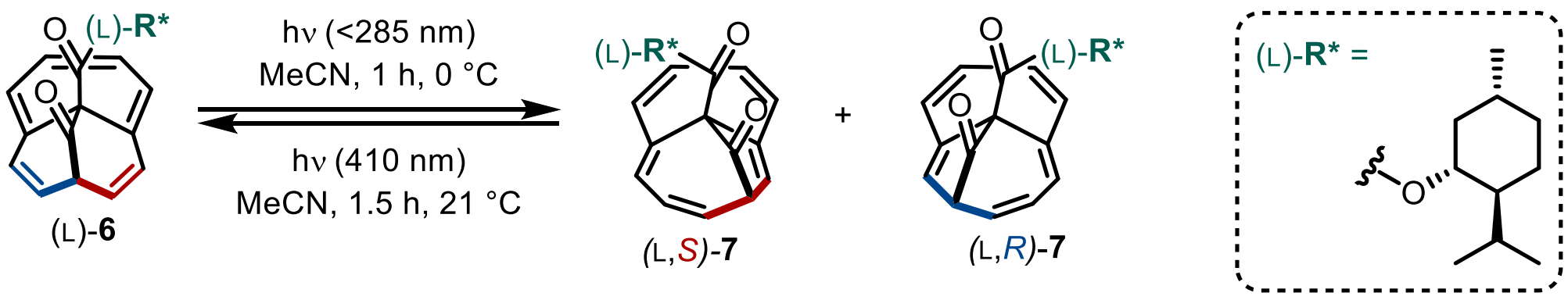

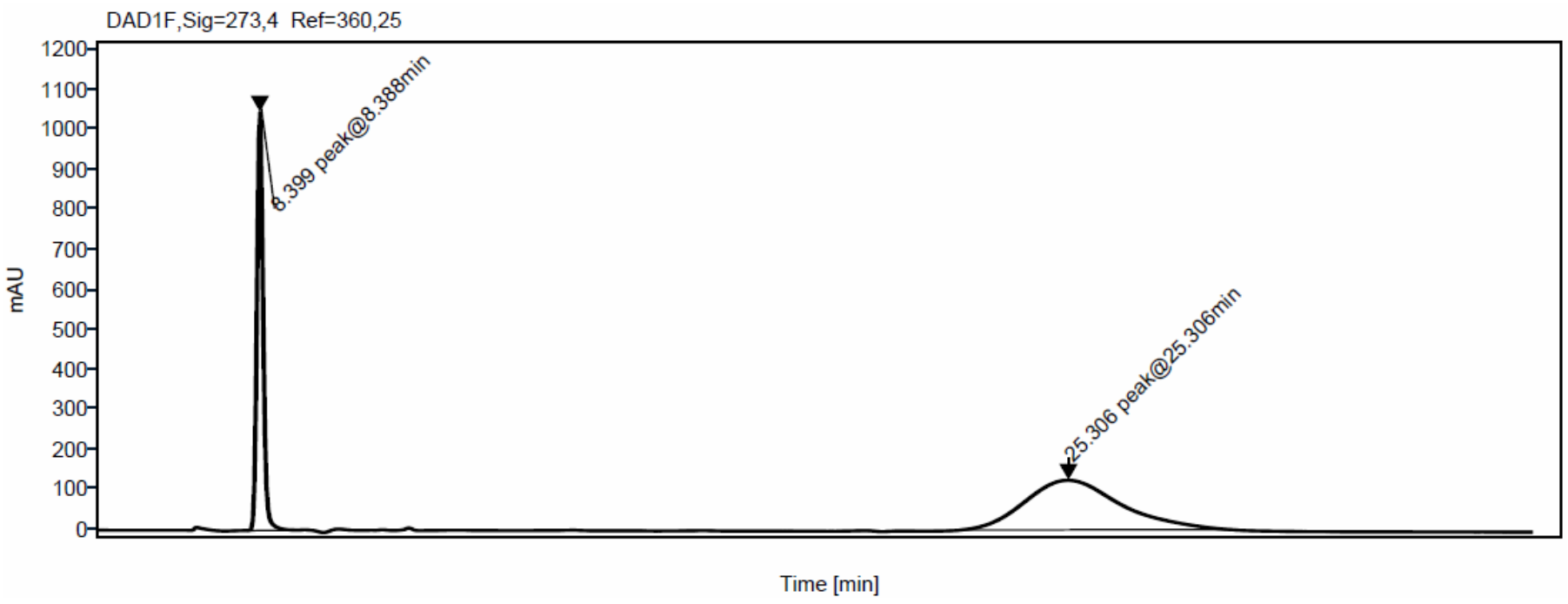

Signal: DAD1F,Sig=273,4 Ref=360,25

Name	RT [min]	Area	Peak Area Percent
peak@8.388min	8.40	9463.533	35.43
peak@25.306min	25.31	17245.153	64.57

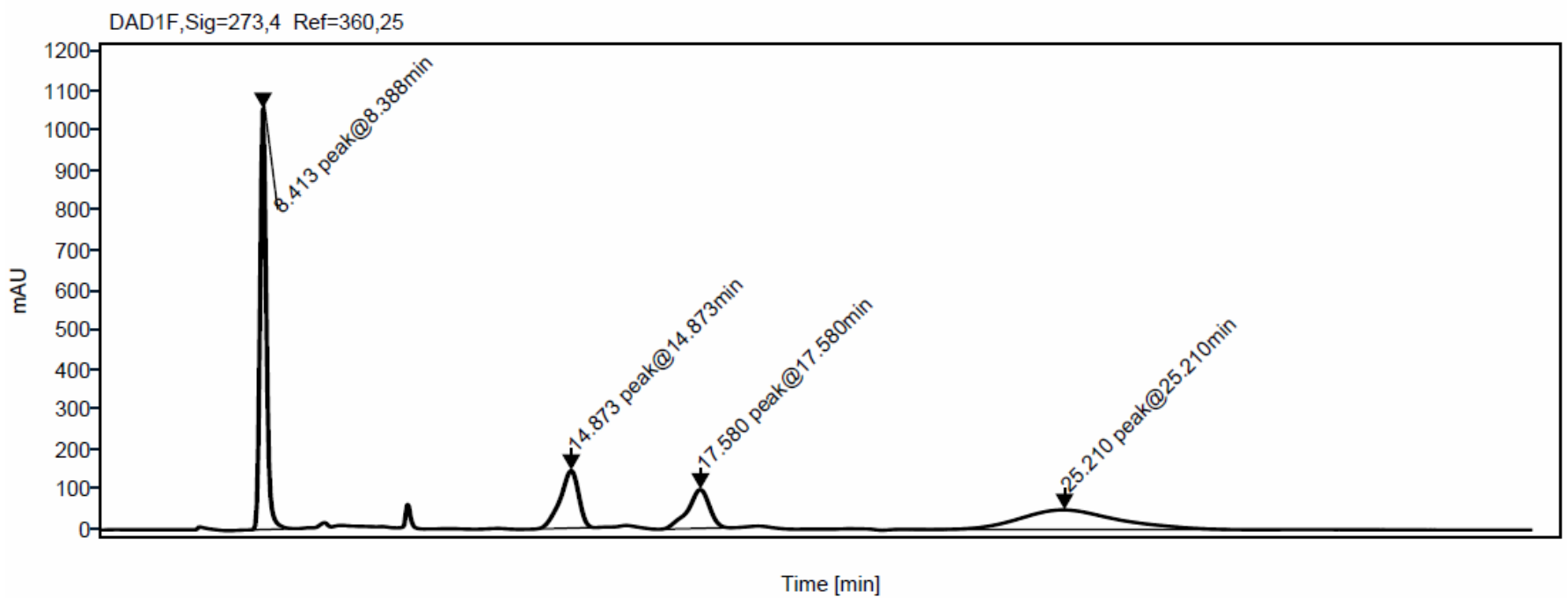

Signal: DAD1F,Sig=273,4 Ref=360,25

Name	RT [min]	Area	Peak Area Percent
peak@8.388min	8.41	9706.751	41.09
peak@14.873min	14.87	3911.062	16.56
peak@17.580min	17.58	3090.677	13.08
peak@25.210min	25.21	6915.260	29.27

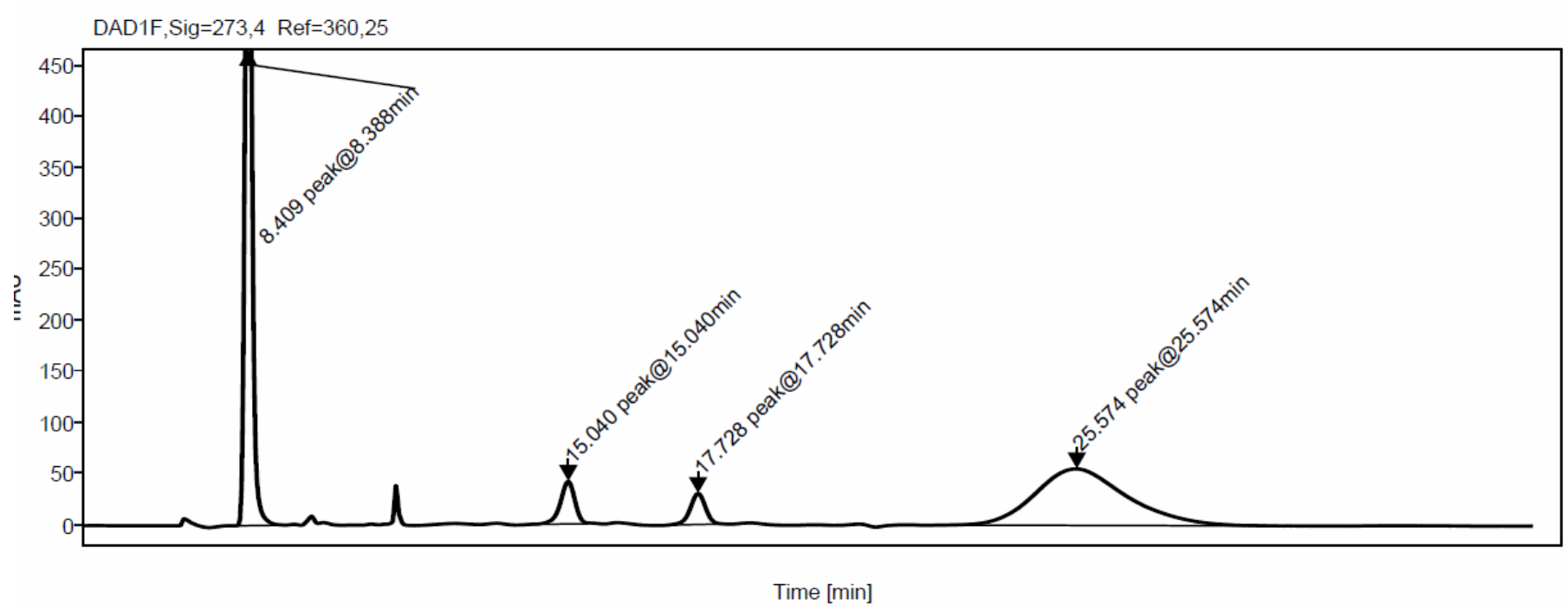

Signal: DAD1F,Sig=273,4 Ref=360,25

Name	RT [min]	Area	Peak Area Percent
peak@8.388min	8.41	8149.162	48.00
peak@15.040min	15.04	862.079	5.08
peak@17.728min	17.73	666.870	3.93
peak@25.574min	25.57	7298.200	42.99

Figure S25. HPLC chromatograms of homoannulene (L)-**6** (top), after irradiation at λ = 285 nm (middle) and after irradiation at λ = 410 nm (bottom). Naphthalene (t = 8.4 min) was added as a reference compound. CHIRALPAK AD-H, *n*-heptane/*i*PrOH = 90:10, flow rate: 0.4 mL · min^{-1}.

In order to investigate, which photodegradation products are formed upon irradiation, a sample of homoannulene menthyl ester (L)-**6** dissolved in CD_3CN was irradiated at λ = 285 nm for 1 h and subsequently analyzed by means of HPLC analysis and ^{1}H NMR spectroscopy (Figure S26 and Figure S27). After 4 h of irradiation at λ = 285 nm homoannulene ester and *iso*-homoannulene esters (L)-**7** were detected in HPLC analysis (retention time t = 15.0, 17.9 and 26.3 min, Figure S26). After additional 3 h of irradiation no signal of homoannulene (L)-**6** or *iso*-homoannulene (L)-**7** was detected. After 4 h as well as 7 h of irradiation additional signals at t = 8.6, 9.7 and 11.4 min were detected. Presumably, these signals correspond to degradation products.

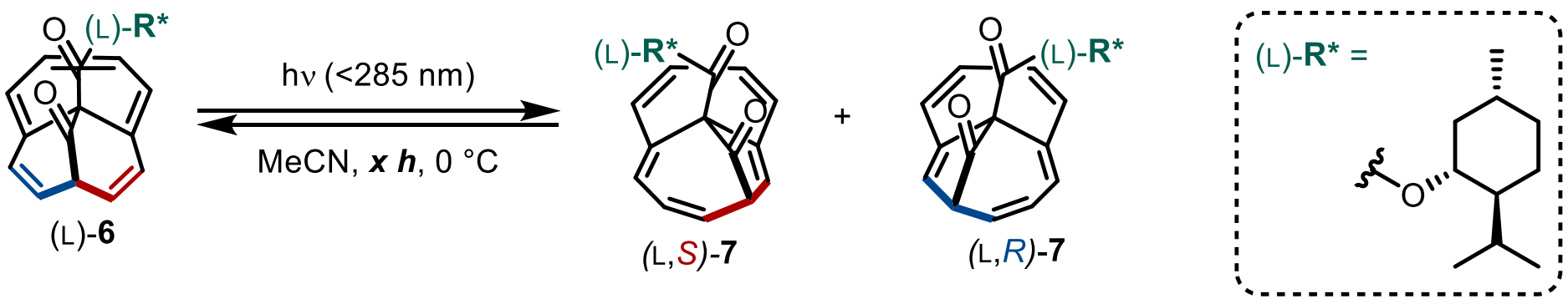

4 h of irradiation:

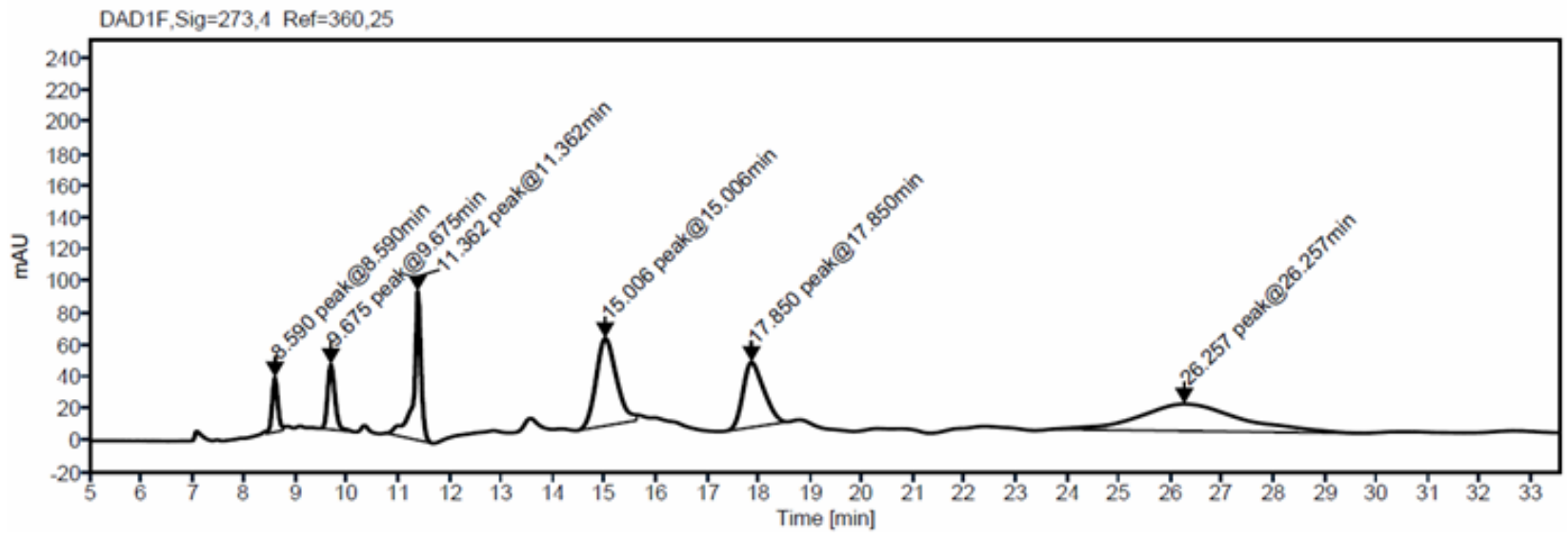

Signal: DAD1F,Sig=273,4 Ref=360,25

Name	RT [min]	Area	Peak Area Percent
peak@8.590min	8.59	258.767	3.94
peak@9.675min	9.67	404.782	6.16
peak@11.362min	11.36	934.516	14.22
peak@15.006min	15.01	1512.432	23.01
peak@17.850min	17.85	1148.156	17.47
peak@26.257min	26.26	2313.988	35.21

7 h of irradiation:

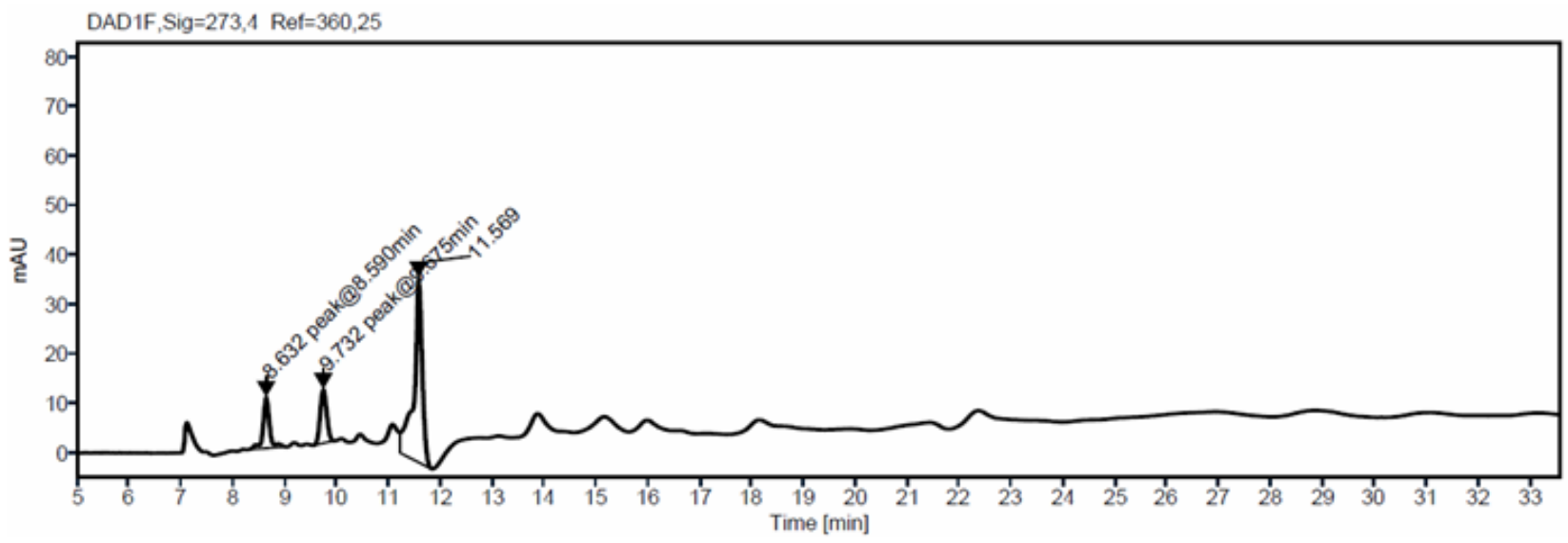

Signal: DAD1F,Sig=273,4 Ref=360,25

Name	RT [min]	Area	Peak Area Percent
peak@8.590min	8.63	88.826	14.76
peak@9.675min	9.73	93.101	15.47

Figure S26. HPLC chromatograms of irradiated samples of (L)-**6** after 4 h (top) and 7 h (bottom).

Furthermore, a ^{1}H NMR spectrum of the sample which was irradiated for 7 h was measured (Figure S27). Besides residues of the solvents used for the irradiation (MeCN-d_3) broad signals with low intensity at δ = 4.5 ppm were detected as well as broad signals from δ = 0–2 ppm, the region in which alkanes are usually detected. In conclusion, the products of photodegradation might be alkanes and alkenes derived from the homoannulene framework.

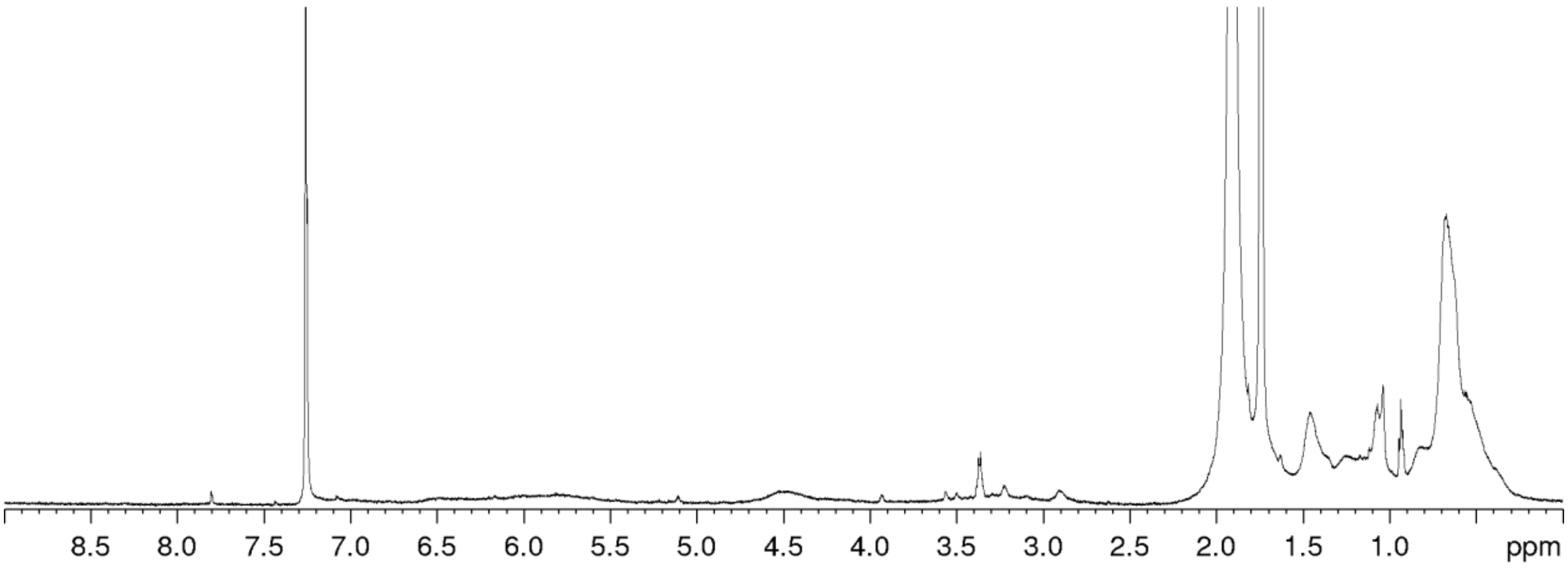

Figure S27. ^{1}H NMR spectrum of the irradiated sample of (L)-**6** in $CDCl_3$ at 600 MHz.

1.7.6.3 Analysis by UV/vis spectroscopy

UV/vis spectroscopy was employed to monitor photoswitching of (L)-**6** over multiple switching cycles. As described previously (see section 1.7.3) homoannulene **6** exhibits an absorption maximum at λ = 255 nm, whereas *iso*-homoannulene (L)-**7** shows a absorption maximum at λ = 380 nm (Figure S28, top). These characteristic absorption bands were therefore used to follow the forward and backward photoswitching process. Homoannulene menthyl ester (L)-**6** was dissolved in MeCN (c = 2.2 10^{-6} mol · L^{-1}). Forward switching was initiated by irradiation with an LED at λ = 280 nm at room temperature for 3 min. Subsequently, backward switching toward homoannulene ester (L)-**6** was induced by irradiation with an LED at λ = 430 nm for 14 min at room temperature. After each forward and backward irradiation step a UV/vis spectrum was recorded and the absorbance at λ = 255 nm and 380 nm was evaluated (Figure S28, bottom). A total of six forward-backward switching cycles were performed. A slight decrease in maximum absorbance of both characteristic bands was observed over successive cycles, which can be attributed to minor photodegradation during repeated irradiation. This is consistent with the absence of isosbestic points in switching of *iso*-homoannulene (L)-**7** to homoannulene (L)-**6**, as discussed in Section 1.7.3.

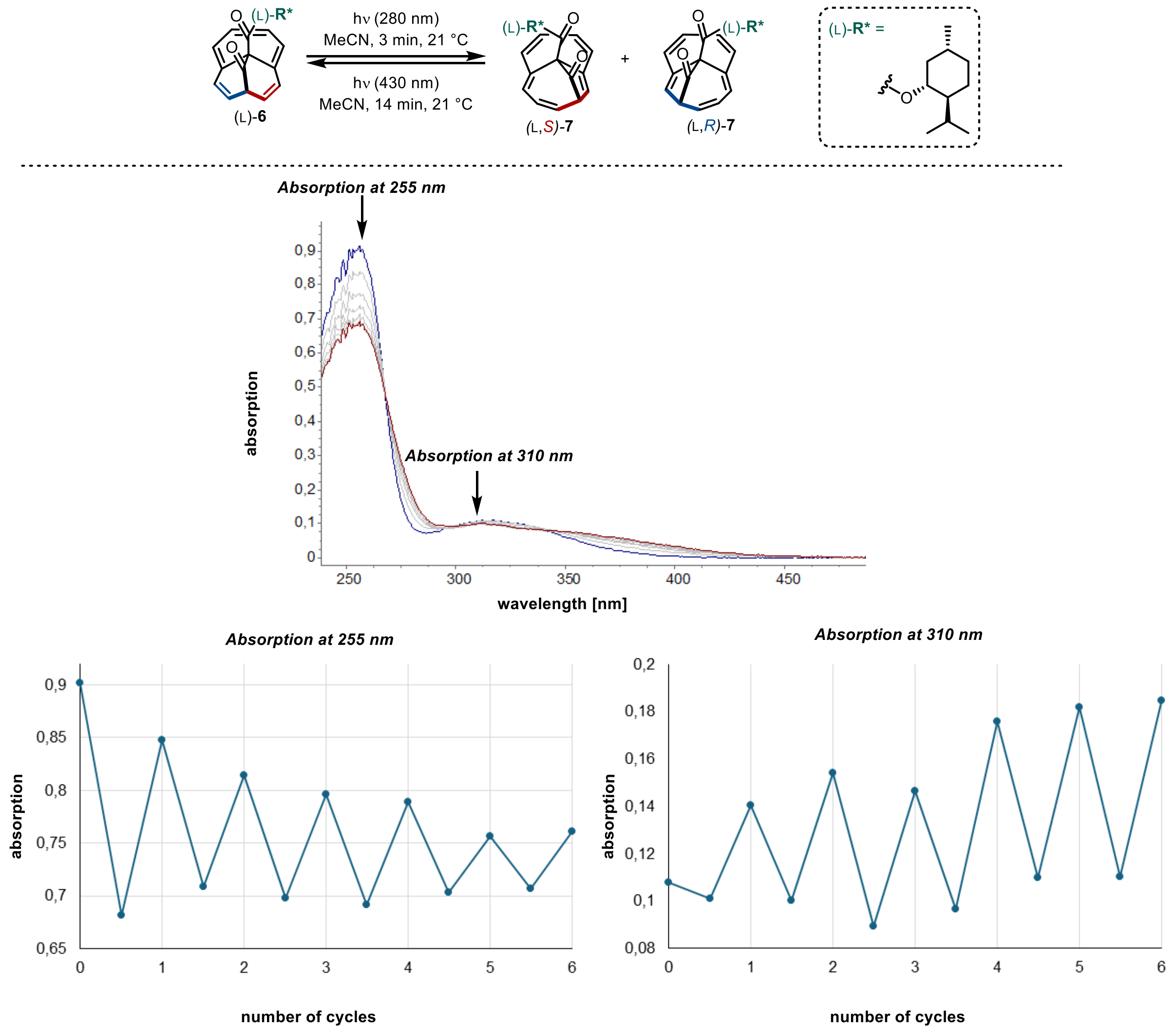

Figure S28. Multiple cycles of photoswitching of homoannulene (L)-**6** monitored by UV/vis spectroscopy. One cycle consists of 3 min irradiation at λ = 280 nm and 14 min of irradiation at λ = 430 nm at room temperature in MeCN.

1.7.7 ECD spectra of homoannulenes 6 and *iso*-homoannulenes 7

Circular Dichroism (CD) spectra were recorded using a JASCO J-1500 Circular Dichroism Spectrophotometer *(JASCO Deutschland GmbH*, Pfungstadt, Germany) equipped with a standard air-cooled 150 W xenon lamp as a light source. Measurements were carried out in quartz cuvettes with a path length of 1 mm over a wavelength range of 200–700 nm. The experimental parameters were as follows: scanning speed of 200 nm · min^{-1}, bandwidth of 1.00 nm, data pitch of 0.1 nm, data integration time (D.I.T.) of 2 s and averaging over three scans. The compounds were dissolved in acetonitrile and from this solution, the analyte was diluted to c = 0.00168 mol · L^{-1} ((D)-**6**), 0.0018 mol · L^{-1} ((L)-**6**), 0.00241 mol · L^{-1} (PSS$_{285\ nm,(L)-6/(L)-7}$) and 0.00303 mol · L^{-1} (PSS$_{285\ nm,(D)-6/(D)-7}$). The acetonitrile baseline was recorded separately and automatically subtracted from all spectra.

ECD spectra of homoannulene esters (L)-**6** and (D)-**6** were measured (Figure S29). For homoannulene menthyl ester (L)-**6** a positive Cotton effect is detected at a wavelength λ = 202–270 nm as well as λ = 270–400 nm (grey curve in Figure S29). Homoannulene ester (D)-**6** displays at the same wavelength as (L)-**6** opposite Cotton effects (black curve in Figure S29). In both compounds the cotton effect at λ = 202–270 nm is of large magnitude which can be attributed to a π→π* transition of the conjugated homoannulene framework as well as the C=O-groups within the homoannulene. The Cotton effect at λ = 270–400 nm is of small magnitude which can be assigned to a formally forbidden n→π* transition of the C=O-functionalities.[25–28] These results show that (L)-**6** and (D)-**6** have mirror-like CD spectra, which discloses the enantiomeric relation of the homoannulene esters.

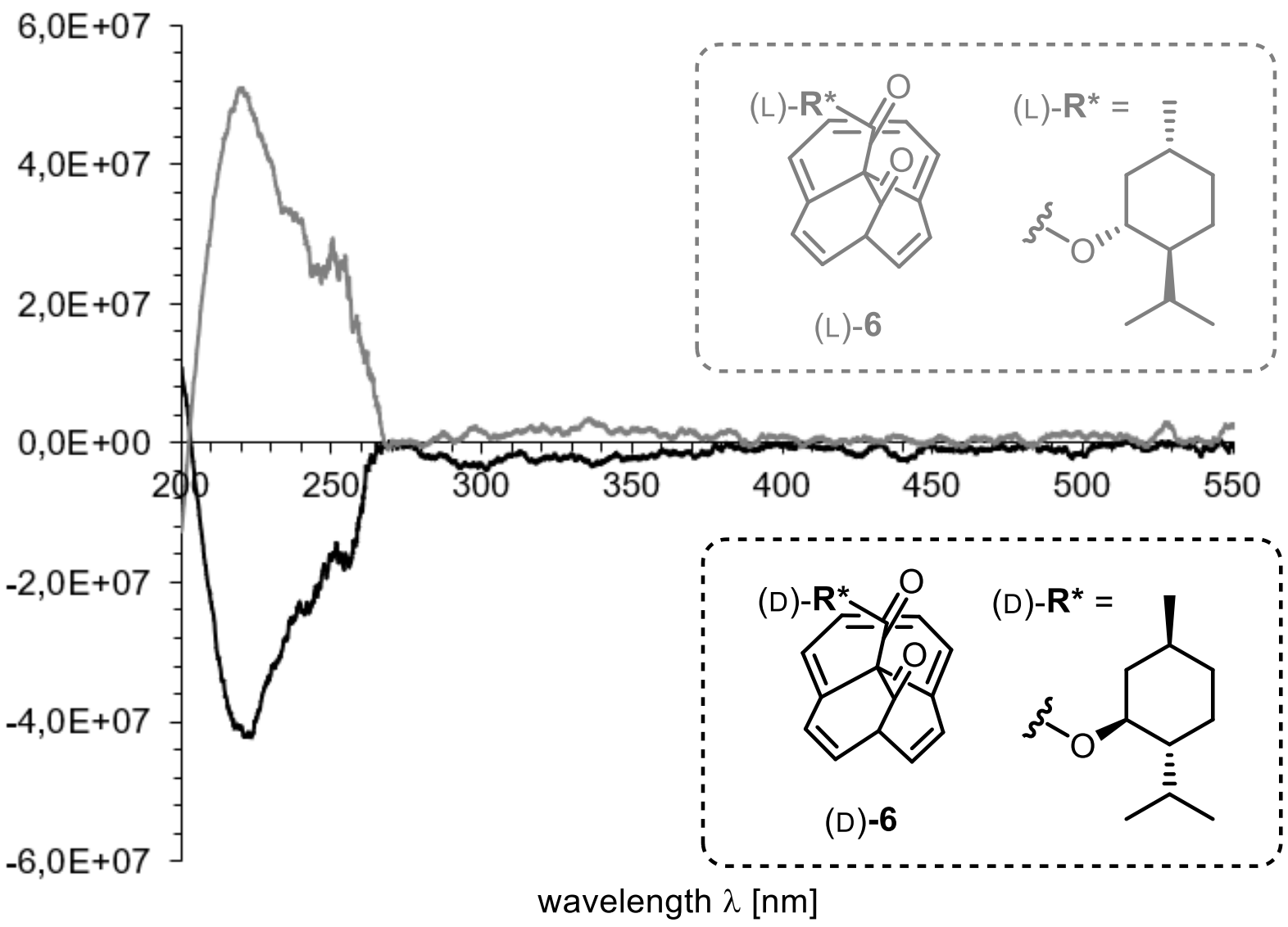

Figure S29. ECD spectra of homoannulene esters (L)-**6** (grey curve) and (D)-**6** (black curve).

Each homoannulene was irradiated with an Hg lamp equipped with a 285 nm cut-off filter for 1 h at 0 °C. ECD spectra of the irradiated reaction mixtures were recorded. The mixture of homoannulene (L)-**6** and the corresponding *iso*-homoannulene esters (L)-**7** display a positive Cotton effect at λ = 200–255 nm, a negative Cotton effect until λ = 295 nm, followed by a positive Cotton effect until λ = 450 nm (grey curve in Figure S30). The mixture of homoannulene (D)-**6** and its corresponding *iso*-homoannulenes (D)-**7** display a negative Cotton effect from λ = 200–251 nm, followed by a positive Cotton effect until λ = 290 nm and another negative Cotton effect until λ = 450 nm (black curve in Figure S30) and is a mirror-like image to the photoswitched mixture of (L)-**6**. The strong effect at λ = 200–255 nm can be assigned to a π→π* transition of the conjugated homoannulene core. The sign inversion in the CD spectra of the photoswitched mixtures could be assigned to π→π* transitions split by

exciton coupling. The weak effect at λ = 295–400 nm could be assigned to a n→π* transition of the carbonyl and ester groups in the chiral environment.[25–29]

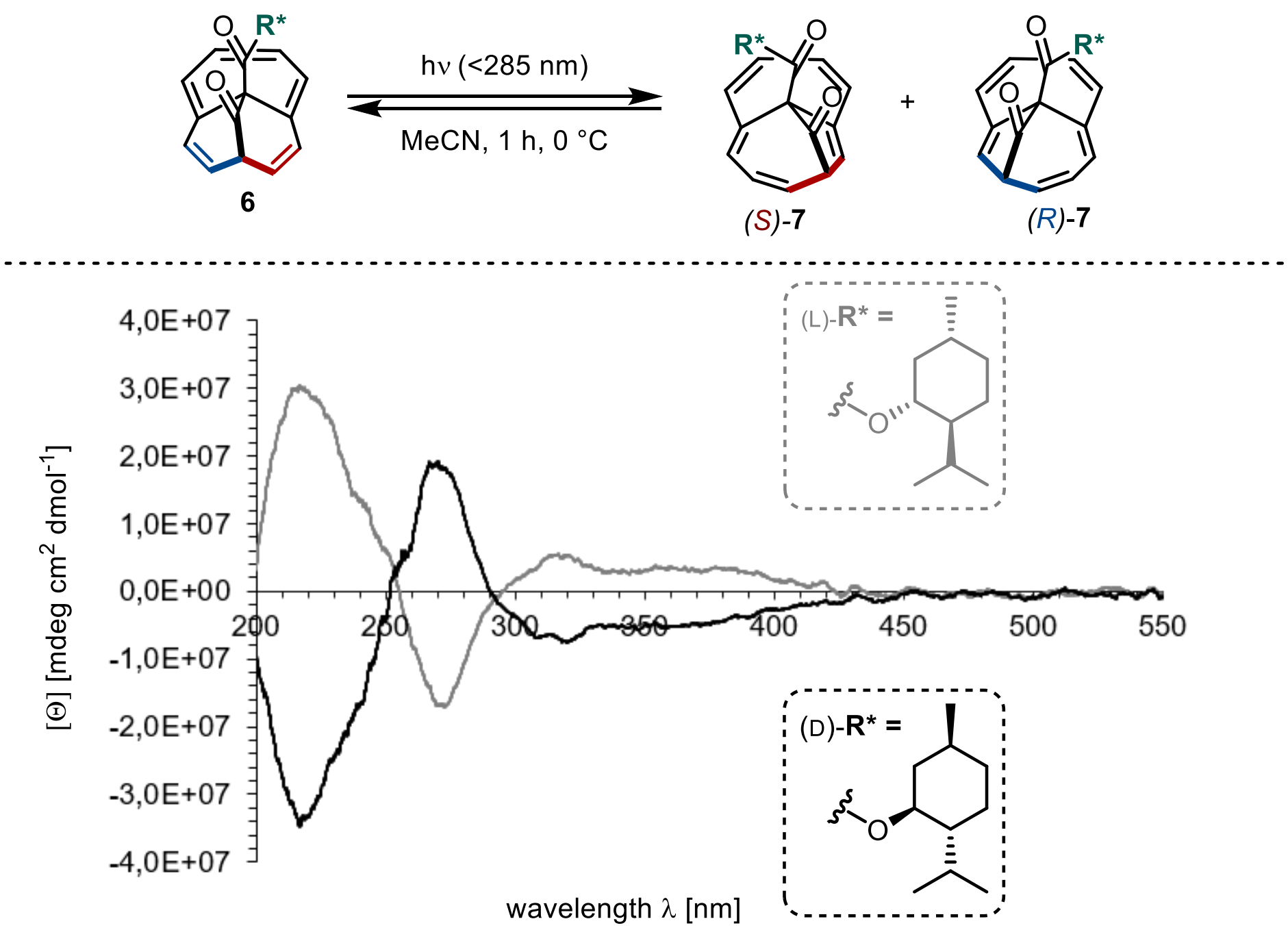

Figure S30. ECD spectra of the photoswitched mixture of (L)-**6**/(L)-**7** (grey graph) and (D)-**6**/(D)-**7** (black graph) at $PSS_{285\ nm}$.

Furthermore, the absorptive dissymmetry factor (g_{abs}) was determined to quantify the magnitude of the observed circular dichroism (CD). The g_{abs} factor is defined as the ratio of the differential molar extinction coefficient (Δε) to the molar extinction coefficient (ε), $g_{abs} = \Delta\varepsilon/\varepsilon$.[30] Since g_{abs} is transition-specific, its wavelength-dependence was evaluated by plotting the corresponding g_{abs} values as a function of the wavelength λ in nm (for homoannulene esters (L)-/(D)-**6** (Figure S31) and for photoswitched mixtures of (L)-/(D)-**6** and (L)-/(D)-**7**) (Figure S32). For homoannulene ester (L)-**6** a maximum g_{abs} value of $2.6 \cdot 10^{-4}$ at λ = 223 nm was determined, whereas the enantiomer (D)-**6** exhibited, at the corresponding wavelength, a g_{abs} value of $-2.4 \cdot 10^{-4}$.

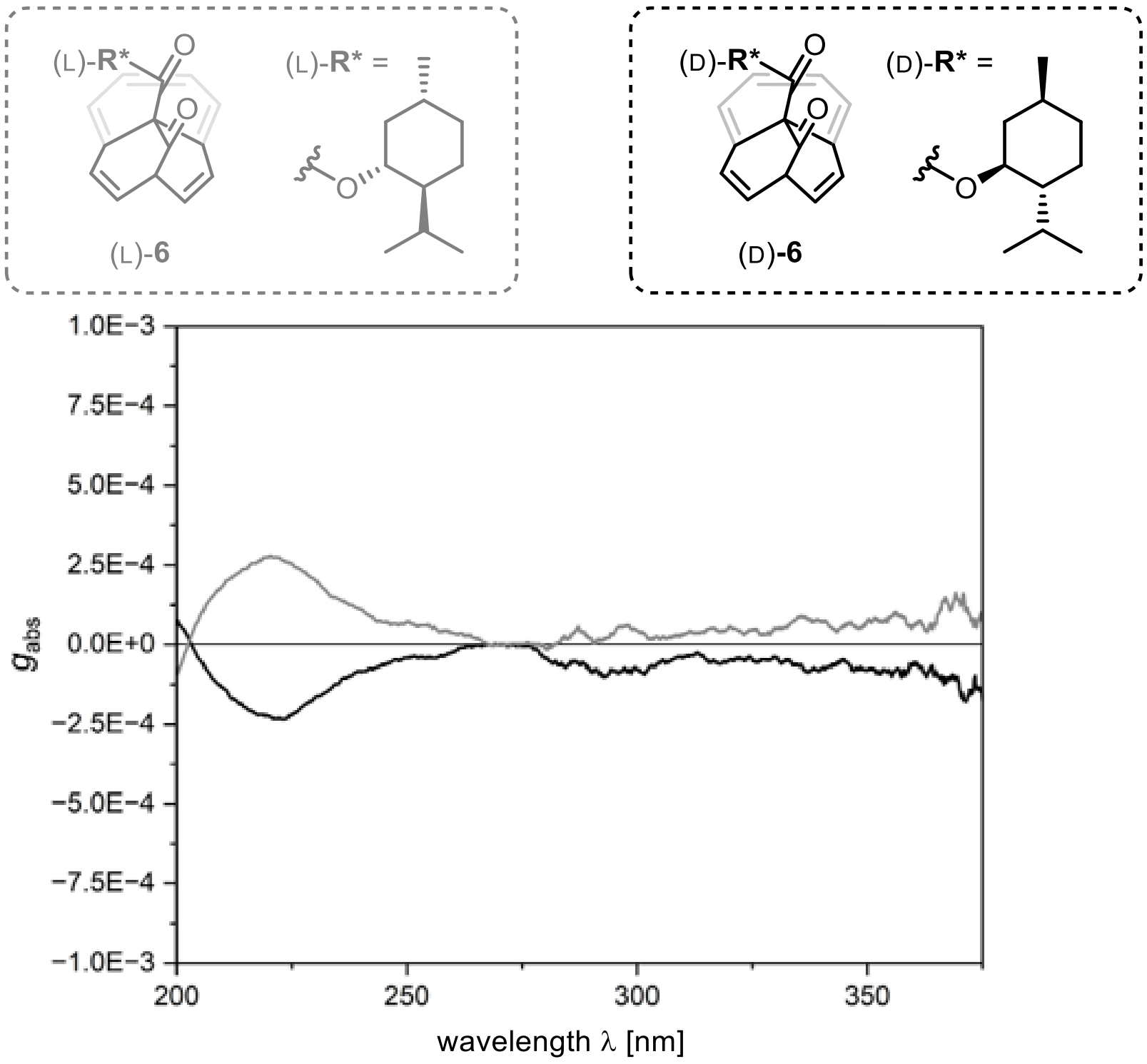

Figure S31. Plotted g_{abs} value against wavelength λ of for homoannulene esters (L)-**6** (grey graph) and (D)-**6** (black graph)**.**

For the photoswitched mixtures of homoannulene esters (L)-**6** and (D)-**6** showed two maxima in the dissymmetry factor g_{abs} at λ = 217 nm and 272 nm, which corresponds to the wavelength at which the CD signals reach its maximum and minimum, respectively. For the irradiated mixture of (L)-**6** and (L)-**7** $g_{abs,217\,nm} = 2.1 \cdot 10^{-4}$ and $g_{abs,272\,nm} = -1.8 \cdot 10^{-4}$ were determined. In contrast, the irradiated mixture of (D)-**6** and (D)-**7** exhibited $g_{abs,217\,nm} = -2.0 \cdot 10^{-4}$ and $g_{abs,272\,nm} = 1.5 \cdot 10^{-4}$. The opposite signs observed for the corresponding enantiomeric mixtures are consistent with their opposite chiroptical responses.

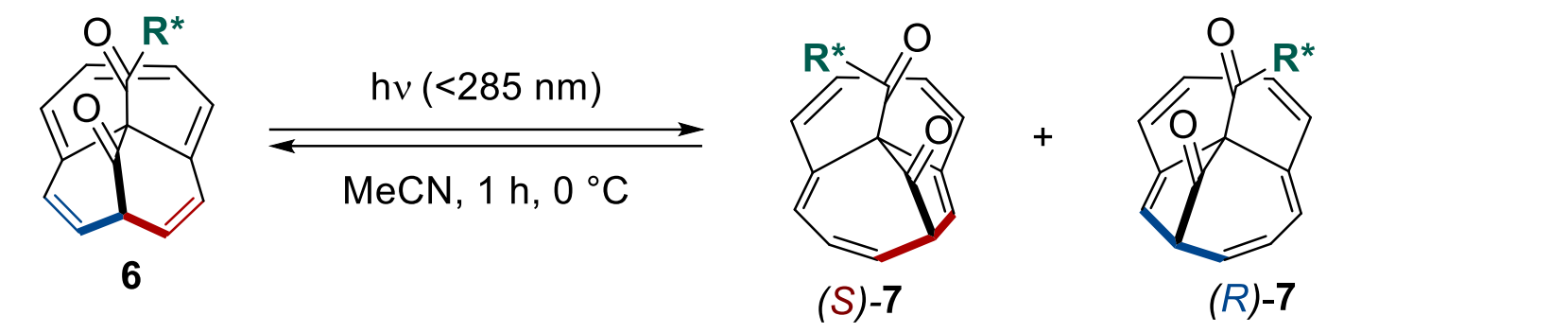

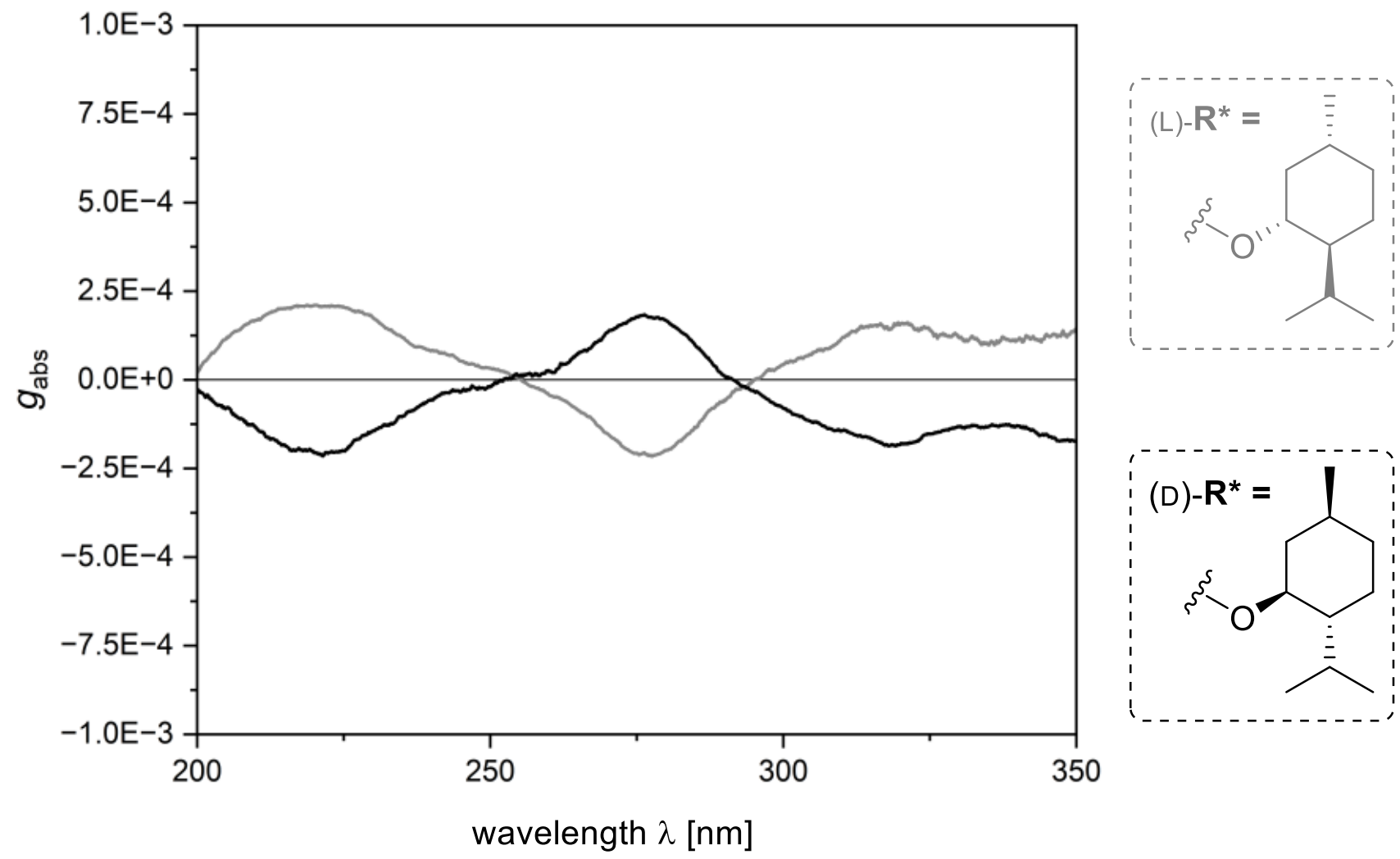

Figure S32. Plotted g_{abs} value against wavelength λ of the photoswitched mixture of (L)-**6**/(L)-**7** (grey graph) and (D)-**6**/(D)-**7** (black graph) at $PSS_{285\ nm}$.

1.7.8 Determination of absolute configuration of *iso*-homoannulenes 7

ECD measurements were carried out with a JASCO J-815 spectrometer in 1 mm pathlength cuvettes. Due to the low amounts of sample available for the measurements, the concentrations could only be approximated to c ≈ 0.5 mM. Irradiation experiments directly prior to the resolution of the diastereomers have been carried out with a vitronic (11 W, Oase) lamp.

The resolution of the diastereomeric photoproducts was performed on a Shimadzu HPLC system equipped with a SIL-20A autosampler, two LC-20AR pumps, a CTO-20AC column oven, an SPD-40 UV/Vis detector, and an FRC-10 fraction collector. Solvents were purchased as HPLC or p. A. grade and degassed prior to use. Solvents were additionally purified using intake filters. Separations were carried out with a semi-preparative Chiralpak IG in n-hexane/dichloromethane 95:5 with a flow rate of 3.5 mL/min and injections volumes of 10 µL per run. In order to collect sufficient amounts of sample for ECD analysis, the separation runs were repeated about 150 times. Fractions were collected in brown-glass flasks and the solvent was regularly removed by vacuum distillation on a dry-ice cooled rotovap. No further purity control was carried out before ECD measurements to avoid back-isomerization effects. For reasons of sustainability, the collected solvent was reused for the next separation runs.

UV/Vis and ECD spectra calculations were performed with the Gaussian 09 rev E software package[31] at CAM-B3LYP/6-311++G(2d,p)/IEFPCM(ACN) level of theory. Based on a systematic conformational search on force field level that considered all rotatable bonds as well as ring conformations of the menthol moiety,[32,33] two dominant conformers were identified among the ~20 optimized geometries of each photoproduct. At room temperature, they account for 65 and 30 % of the conformational distribution of (L,*S*)-**7** and 55 and 42 for (L,*R*)-**7**. In both cases, the key structural difference is the orientation of the menthol group, *i.e.*, the rotation along the O-C$^{(=O)}$-C$_{quart}$-C$^{(=O)}$ torsional angle (Cartesian coordinates of the structures are given in the Spectral and Chromatographic Appendix). All other conformers contribute with less than 2 % The dipole and rotational strengths from TD-DFT calculations (nstates = 50) were broadened with a Gaussian band shape of 0.3 eV HWHH, and the predicted excitation energies were shifted by +10 nm for better comparison with the experiment.

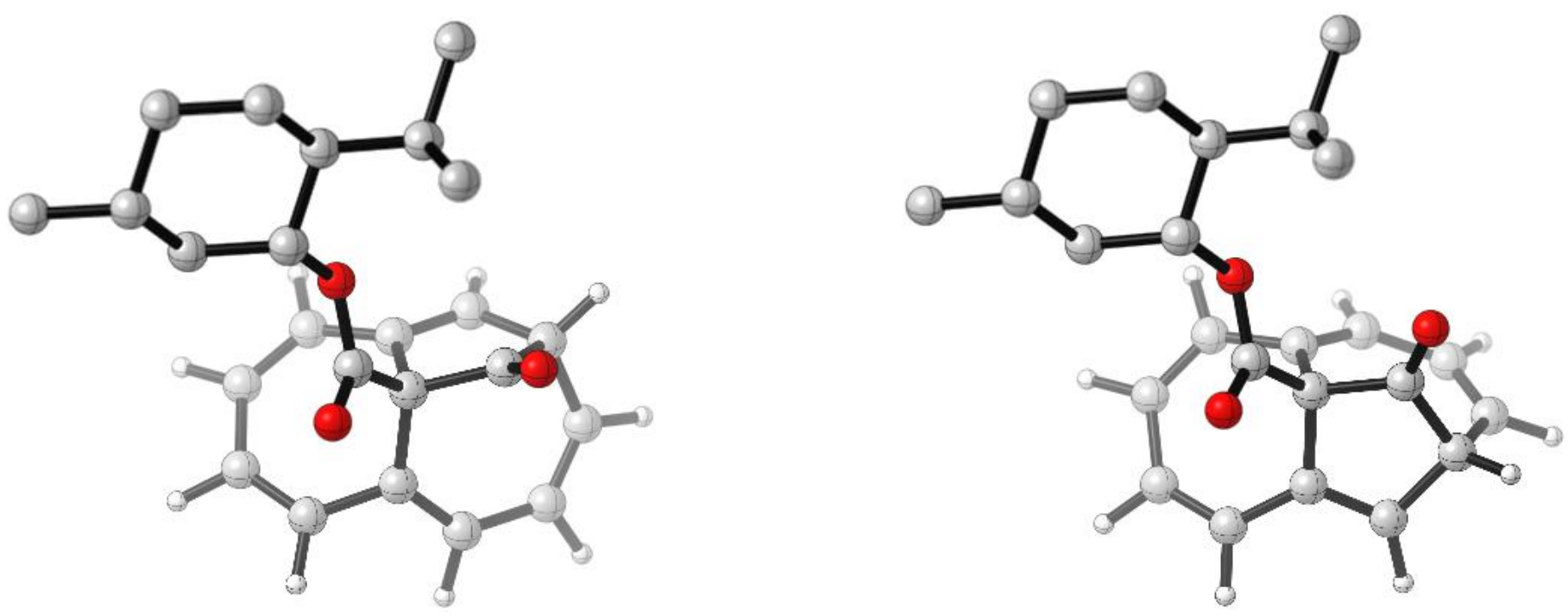

Figure S33. Lowest energy conformers of *iso*-homoannulene *(*L,*S)*-**7** (left) and *(*L,*R)*-**7** (right).

1.8 Investigation on rotational barriers in homoannulenes 4, 6 and *iso*-homoannulenes 7

Rotational barriers in homoannulene ester **4** and (L)-**6** as well as *iso*-homoannulene esters (L,*S*)-**7** and (L,*R*)-**7** were investigated experimentally and computationally. Variable temperature (VT) NMR experiments were conducted in order to estimate the point of coalescence and with that, derived from the Eyring equation, a barrier of rotation. The following equation was applied:[34,35]

$$\Delta G^{\ddagger}_{rot} = R \cdot T_C \cdot (22.96 + \ln \frac{T_C}{\Delta\nu})$$

Equation S1. Equation for determining rotational barriers.

R is defined as the gas constant (R = 8.314 J·K^{-1}·mol^{-1}), and T_C is the temperature at which coalescence was detected. The difference of the signals $\Delta\nu = \delta_a - \delta_b$ is applied to the equation in Hz and was determined using the ^{1}H NMR spectrum at room or low temperature with separation of the signals, that coalesce upon varying the temperature.[34,35] In principle, the equation shown can be applied when two singlets of equal intensity are observed at low temperature and the exchanging nuclei are not coupled to each other.[36] For an estimation of experimentally derived rotational barriers, we used the center of gravity of the signal that undergoes coalescence at higher temperature. The signals selected for the calculation of rotational barriers were chosen to have intensities as similar as possible. Within the context of this work it was decided to not do a complete line shape analysis as it would go beyond the scope of this context.[37] To complement the experimentally accessible data, computationally derived rotational barriers for plausible bond rotations in the respective (*iso*-)homoannulene esters **4**, **6** and **7** were determined as well.

1.8.1 Rotational barriers in homoannulene methyl ester 4

As previously experimentally shown,[14] homoannulene methyl ester **4** displays a hindered rotation of the ester group at room temperature, which results ultimately in a distinct AA'XX' system of the olefinic hydrogen atoms in homoannulene **4**. The corresponding rotational barrier of $\Delta G^{\ddagger}_{\mathbf{4}}$ = 76 kJ · mol^{-1} was determined.[38]

A computational analysis of the rotational barriers of individual bonds was carried out for homoannulene methyl ester **4**. Three rotations were considered in homoannulene ester **4** (Scheme S6), including the bond rotation of the $C_{carbonyl}$–O_{ester} – bond resulting in the s-*cis* and s-*trans* conformation of the methoxy ester (*Bond rotation A* in Scheme S6), and the rotation of the ester carbonyl group (bond rotation of $C_{carbonyl}$–$C_{bridgehead}$ bond each fixed in the s-*cis* and s-*trans* conformation, respectively (*Bond rotations B* and *C* in Scheme S6).

Bond rotation A:

· ***rotation of the $C_{carbonyl}$–O_{ester} bond***

Bond rotation B:

· ***rotation $C_{carbonyl}$–$C_{bridgehead}$ bond***

· ***s-cis conformation of the ester is fixed***

Bond rotation C:

· ***rotation $C_{carbonyl}$–$C_{bridgehead}$ bond***

· ***s-trans conformation of the ester is fixed***

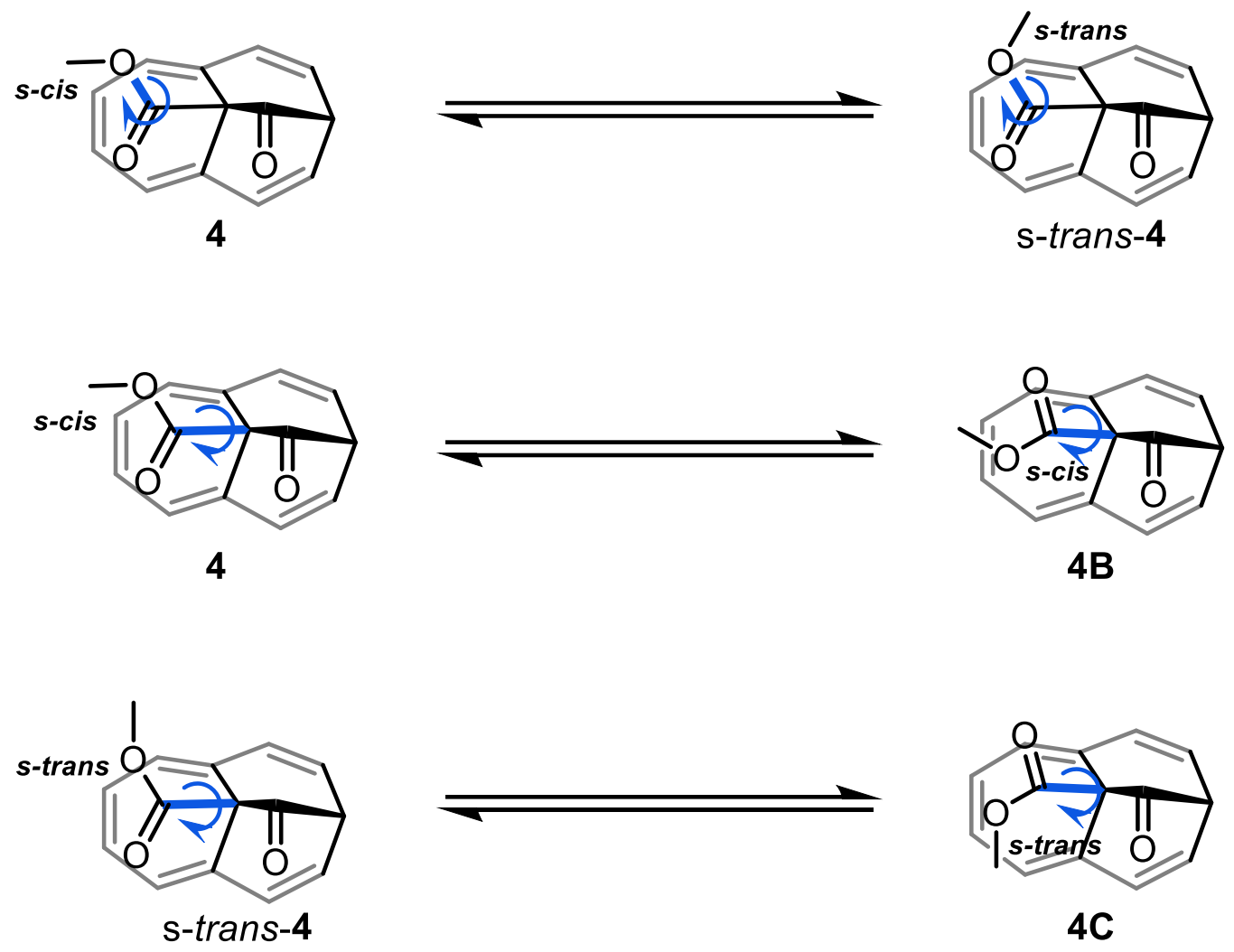

Scheme S6. Computationally investigated bond rotations of homoannulene ester **4**.

Scans of bond rotations were conducted conducted (Figure S34, Figure S35) using the TPSS-D3(BJ)/def2-SVP/IEFPCM($CHCl_3$) level of theory with a step size of 10°.

A rotation around the $C_{carbonyl}$–O_{ester} bond results in two minima structures, (s-*cis* and s-*trans* conformation, **4** and s-*trans*-**4** in Figure S34). The calculations indicated that the s-*cis* conformation of the ester in **4** is lower in energy compared to the s-*trans* conformation s-*trans*-**4** by ΔE_1 = 27 kJ mol^{-1}. Barriers of $\Delta E^{\ddagger}_{\mathbf{4},1}$ = 63 kJ mol^{-1} and $\Delta E^{\ddagger}_{\mathbf{4},2}$ = 54 kJ mol^{-1} between these two conformations were determined.

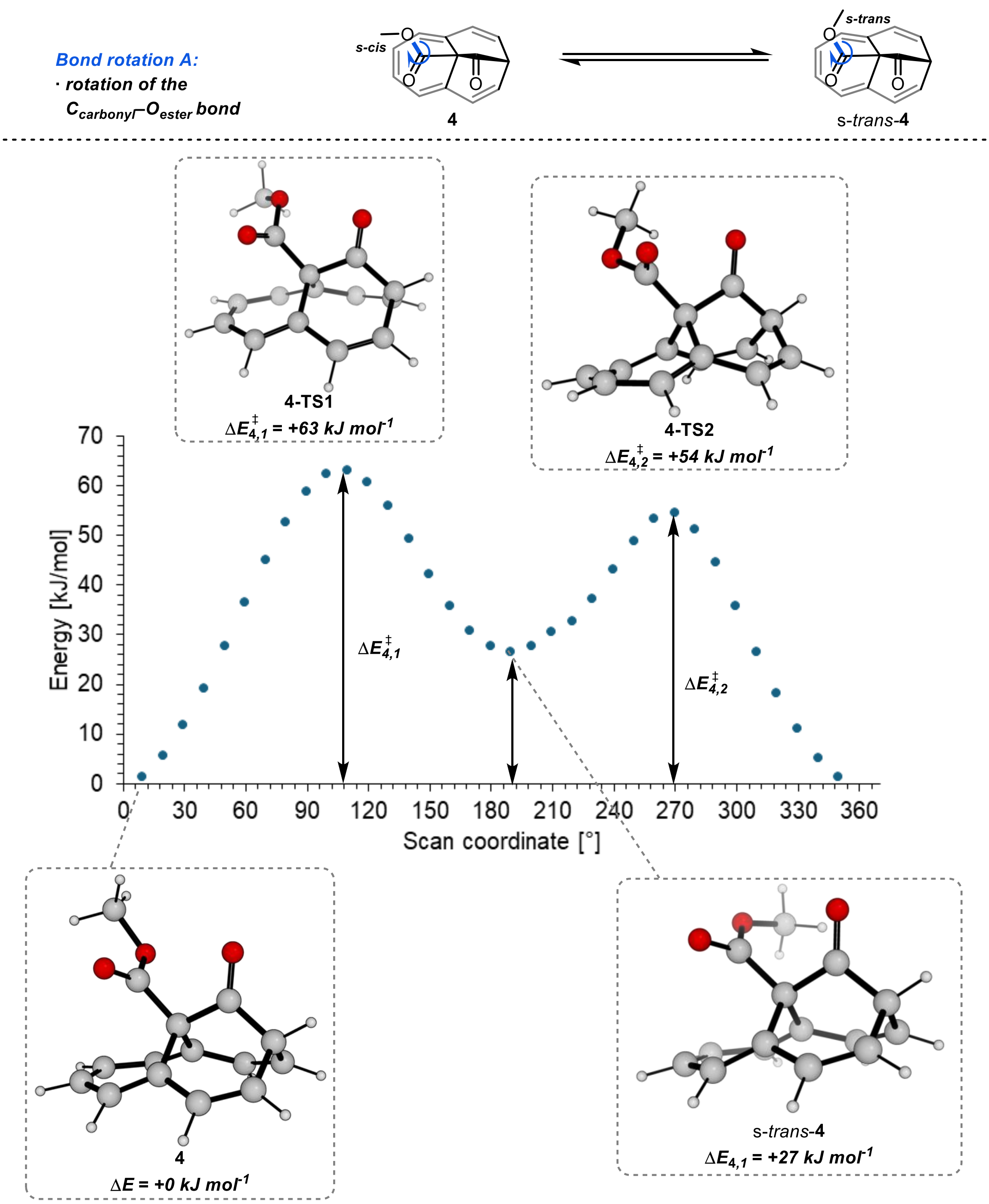

Figure S34. Scan of bond rotation $C_{carbonyl}$–C_{ester} in homoannulene methyl ester **4**. Scan was conducted on the TPSS-D3(BJ)/def2-SVP/IEFPCM($CHCl_3$) level of theory with a step size of 10°.

As the s-*trans* conformation was calculated to be significantly less stable than the s-*cis* conformation, only the bond rotation of the $C_{carbonyl}$–$C_{bridgehead}$ in homoannulene **4** in s-*cis* conformation was considered (Figure S35).

A rotation around the $C_{carbonyl}$–$O_{bridgehead}$ bond results in two minima structures (**4** and **4B**, Figure S35), which are equal in energy. This can be rationalized as conformers are enantiomers. The barriers of rotations were calculated to be $\Delta E^{\ddagger}_{4,3}$ = 67 kJ mol^{-1} and

$\Delta E^{‡}_{4,4}$ = 74 kJ mol^{-1} and are within the range of the experimentally observed rotational barrier in homoannulene **4**.

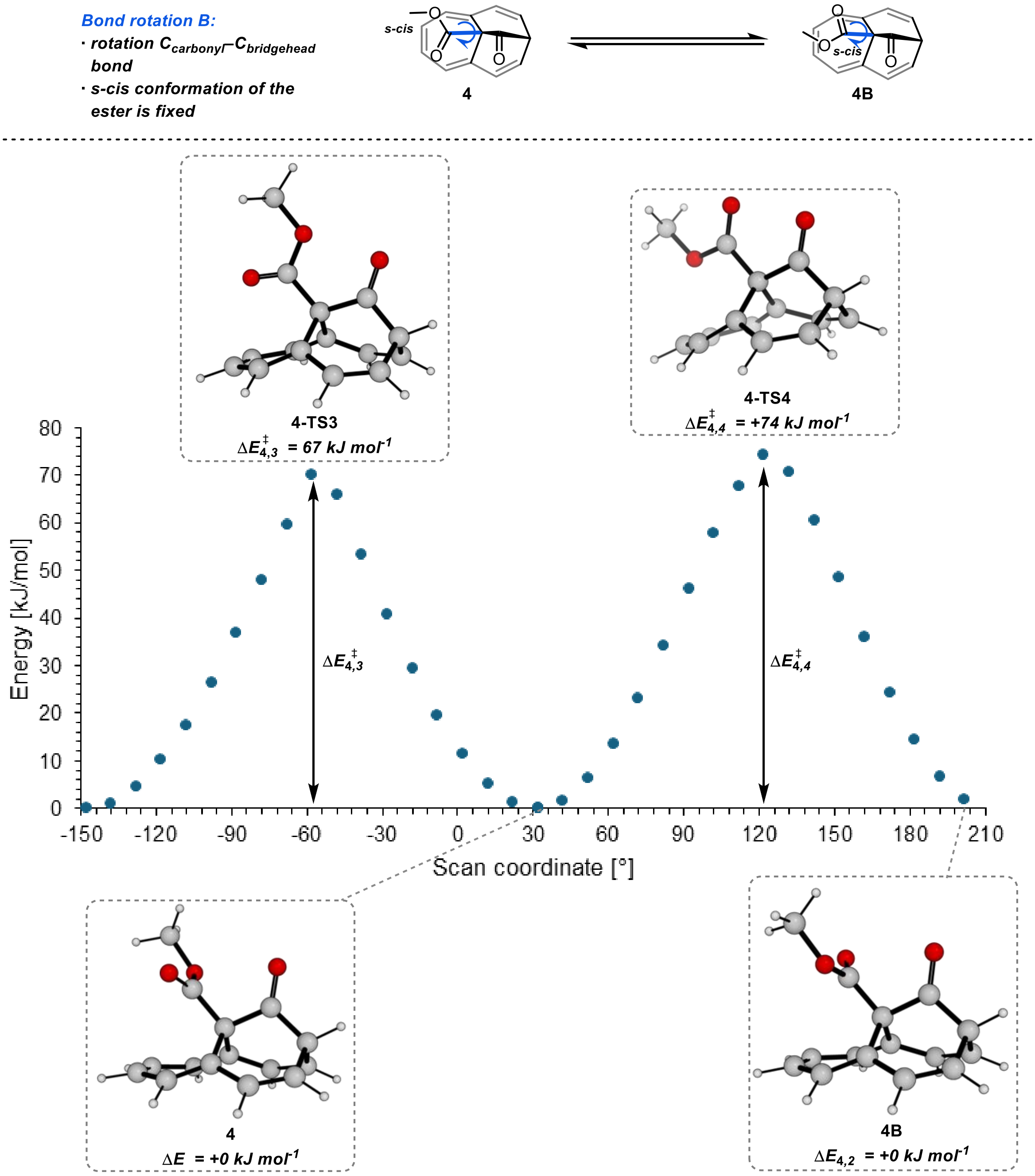

Figure S35. Scan of bond rotation C$_{carbonyl}$–C$_{bridgehead}$ in homoannulene methyl ester **4**. Scan was conducted on the TPSS-D3(BJ)/def2-SVP/IEFPCM($CHCl_3$) level of theory with a step size of 10°.

1.8.2 Rotational barriers in homoannulene menthyl ester 6

1.8.2.1 ^{1}H NMR spectroscopy

In the ^{1}H NMR of homoannulene menthyl esters **6** second signal sets for hydrogen atoms were observed (Figure S36). For instance, two signal sets were detected which were assigned to hydrogen atom H-10 of the menthyl substituent (δ = 4.20 and 4.38 ppm, blue box, Figure S36). The integrated area of the two doublet of triplets are of similar size (corresponding each to 0.5 hydrogen atoms).

As observed in homoannulene methyl ester **4** (see section 1.8.1), due to a hindered rotation in the ester group, the hydrogen atoms of the homoaromatic moiety were detected as a AA'XX' coupling system. The splitting of the respective signals in homoannulene **6** is comparable to the literature described homoannulene **4**. However, for homoannulene **6** the observed signals in the ^{1}H NMR spectrum displayed more signal sets than anticipated. As a hindered rotation of the ester moiety in menthyl homoannulene ester **6** at the given temperature would result in diastereomers, which are distinguishable in NMR spectroscopy, the second detected signal sets for hydrogen atoms in homoannulene **6** can be rationalized. Additionally, only in case the rate of the rotation is slow compared to the time scale of the NMR experiment,[34] a separate signal which can be assigned to rotamers would be in principle detectable.

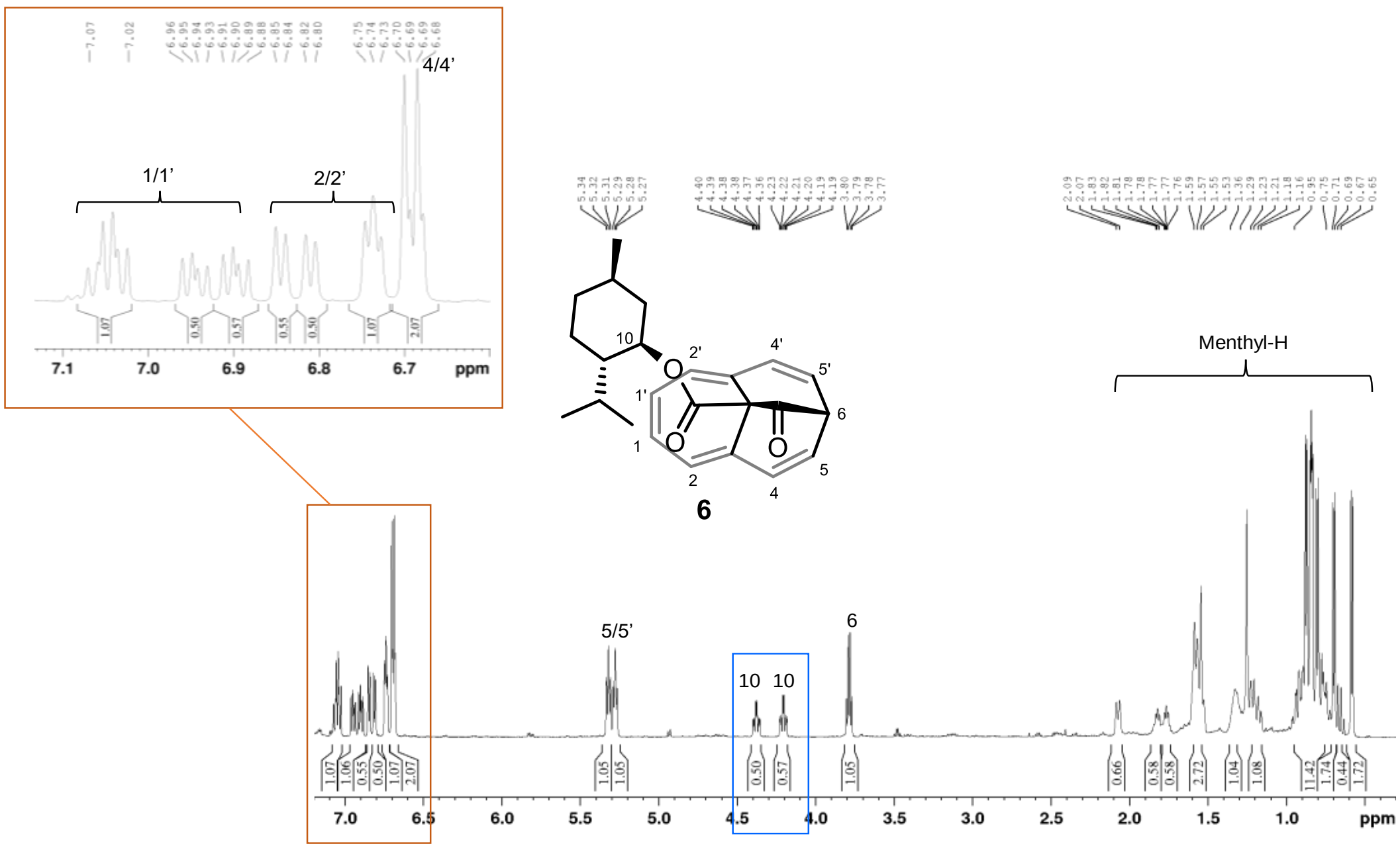

Figure S36. ^{1}H NMR spectrum of (L)-**6**. Inset: Zoom of detected signals from δ = 6.60–7.10 ppm (orange box). The spectrum was measured in $CDCl_3$ at 600 MHz.

1.8.2.2 EXSY experiments

EXSY (Exchange SpectroscopY) experiments were conducted in order to investigate whether chemical exchange between two hydrogen atoms can be detected on the NMR timescale at room temperature. The EXSY pulse sequence is the same as in the NOESY experiment and one can read out chemical exchange between H atoms with cross peaks that have the same phase as the diagonal (whereas NOE cross peaks have the opposite sign as the diagonal). If the rotational barrier can be overcome at room temperature, chemical exchange would be observed.

Chemical exchange was observed for hydrogen atoms of the menthyl substituent as well as the homoaromatic scaffold (Figure S37), e.g. the two doublet of triplets at d = 4.38 and 4.20 ppm, which could be assigned to the H-atoms H-10 of the menthyl substituent (named "EXSY 1" in Figure S37) and also for H-atom H-1/1' a cross peak with the same phase as the diagonal (named "EXSY 2" in Figure S37) was detected. The chemical exchange observed in the NOESY/EXSY spectrum could be rationalized by two rotamers, which interconvert in the NMR timescale at the temperature used.

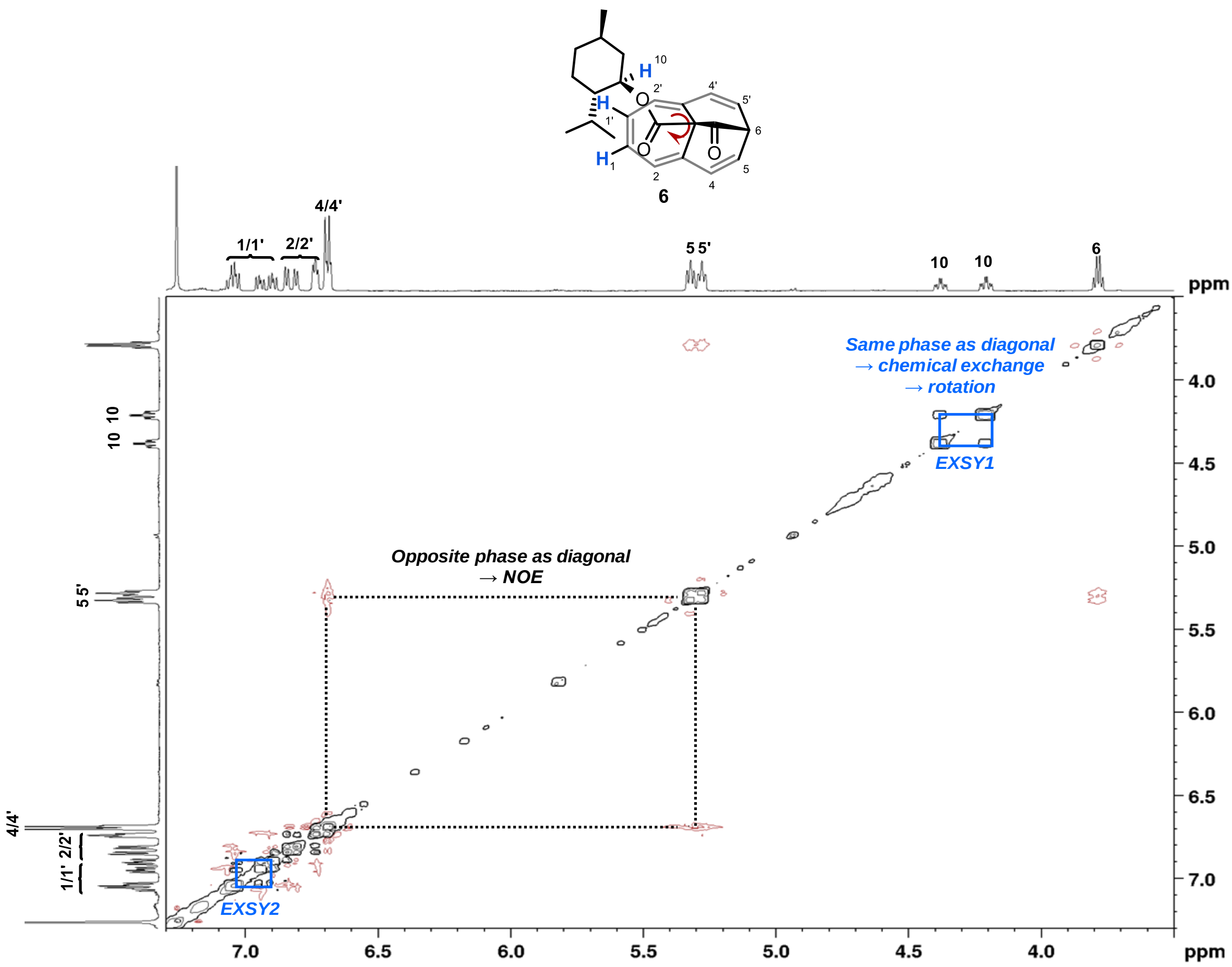

Figure S37. Section of measured NOESY (cross peaks with red color) and EXSY (cross peaks with black color, highlighted for H-1/1' and H-10 with blue box) spectrum of homoannulene (L)-**6.**

1.8.2.3 VT-NMR experiments

Variable temperature (VT) NMR experiments were conducted in order to investigate the barrier of bond rotations experimentally. Higher temperatures should increase the rate of rotation which then causes a merging of the former separate detected signal sets for one H-atom. In this vein, ^{1}H NMR spectra were measured from a temperature of 25 °C until 130 °C in *o*-dichlorobenzene-*d*$_4$ (Figure S38). After reaching the maximum temperature of 130 °C, the sample was cooled down to a temperature of 25 °C and another spectrum was recorded (orange spectrum, Figure S38). Coalescence was detected at a temperature of 110 °C (blue box, Figure S38). A rotational barrier $\Delta G^{\ddagger}_{rot}$ was determined to be $\Delta G^{\ddagger}_{rot}$ = 77 kJ · mol^{-1}.

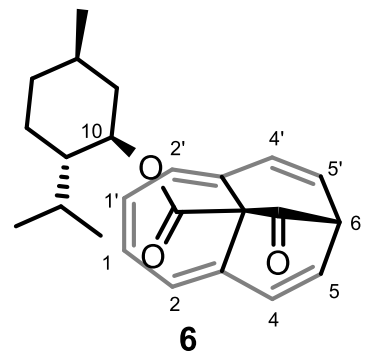

6

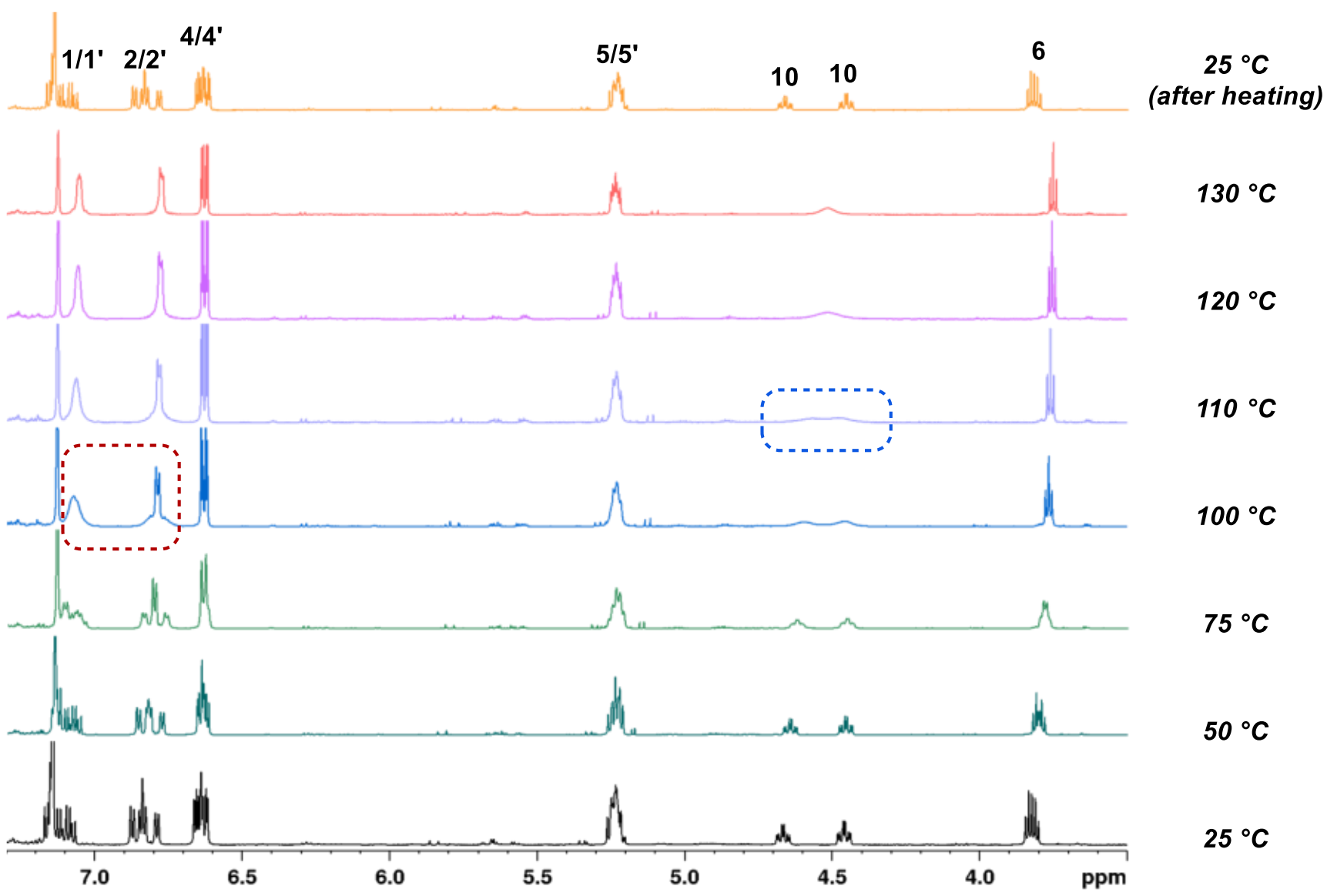

Figure S38. VT-NMR experiments in the range of 25 °C and 130 °C of (L)-**6**. Spectra were measured in *o*-dichlorobenzene-*d*$_4$ at 600 MHz.

Furthermore, it was anticipated that by decreasing the temperature the rate of rotation should be lowered and it was investigated whether bond rotations slow down at lower temperatures and distinct signals can be detected in the ^{1}H NMR spectrum. In this vein, ^{1}H NMR spectra in the range of 25 °C and –114 °C in THF-*d*$_8$ of homoannulene ester (L)-**6** were measured (Figure S39). A point of coalescence was detected at –114 °C, which equals an estimated rotational barrier $\Delta G^{\ddagger}_{rot,2}$ of $\Delta G^{\ddagger}_{rot,2}$ = 35 kJ · mol^{-1}.

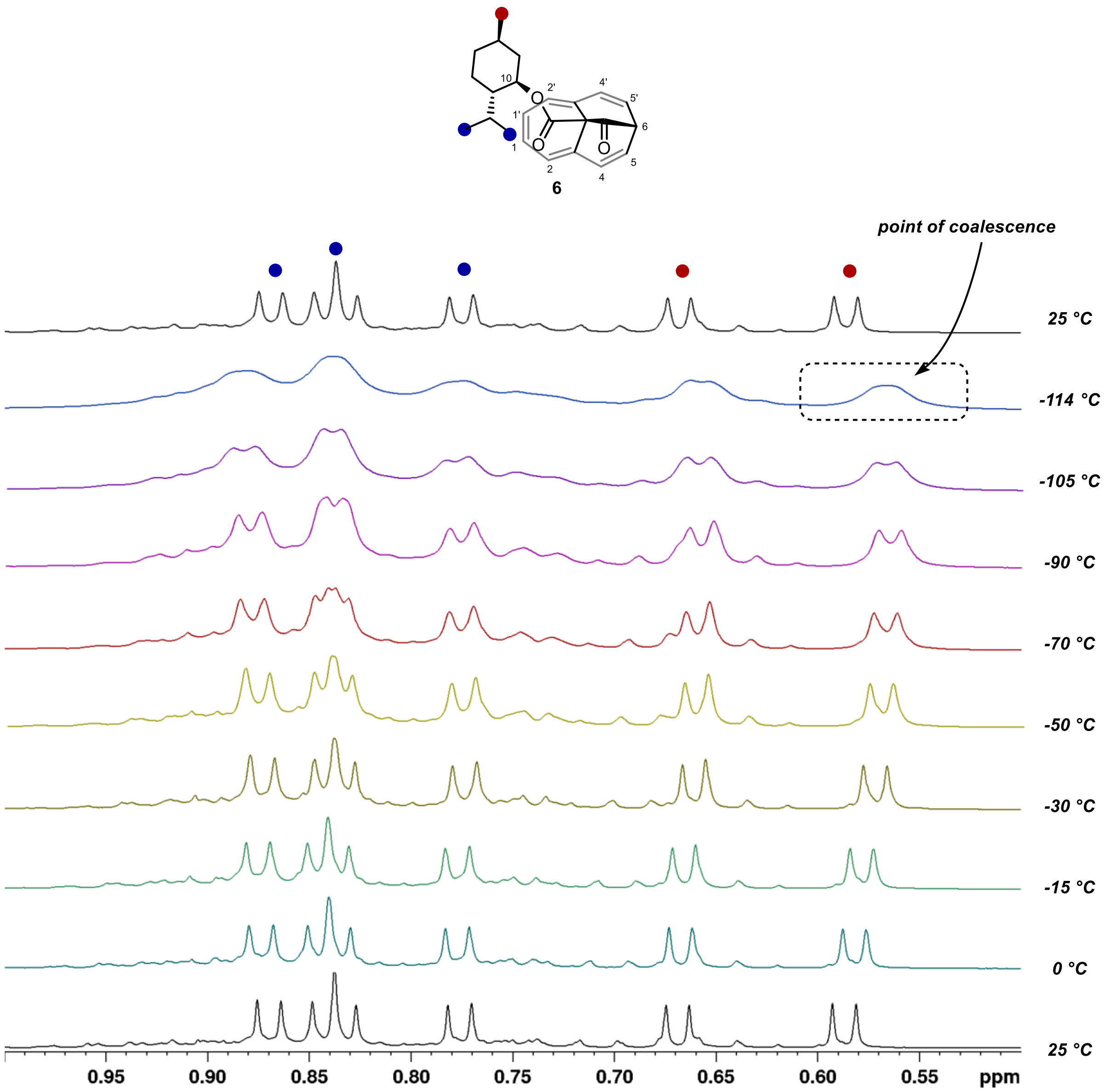

Figure S39. Spectra of VT-NMR experiments in the range of 25 °C and –114 °C. Spectra are shown from δ = 0.50 to 1.0 ppm. Spectra were measured in THF-d_8 at 600 MHz.

Compared to the signals in the region of δ = 0.50–1.0 ppm (see Figure S40) which contains signals that were associated with the menthyl substituent, in the region of δ = 4.0–7.2 ppm (^{1}H NMR signals of the homoannulene scaffold) no substantial change in the signal pattern was detected.

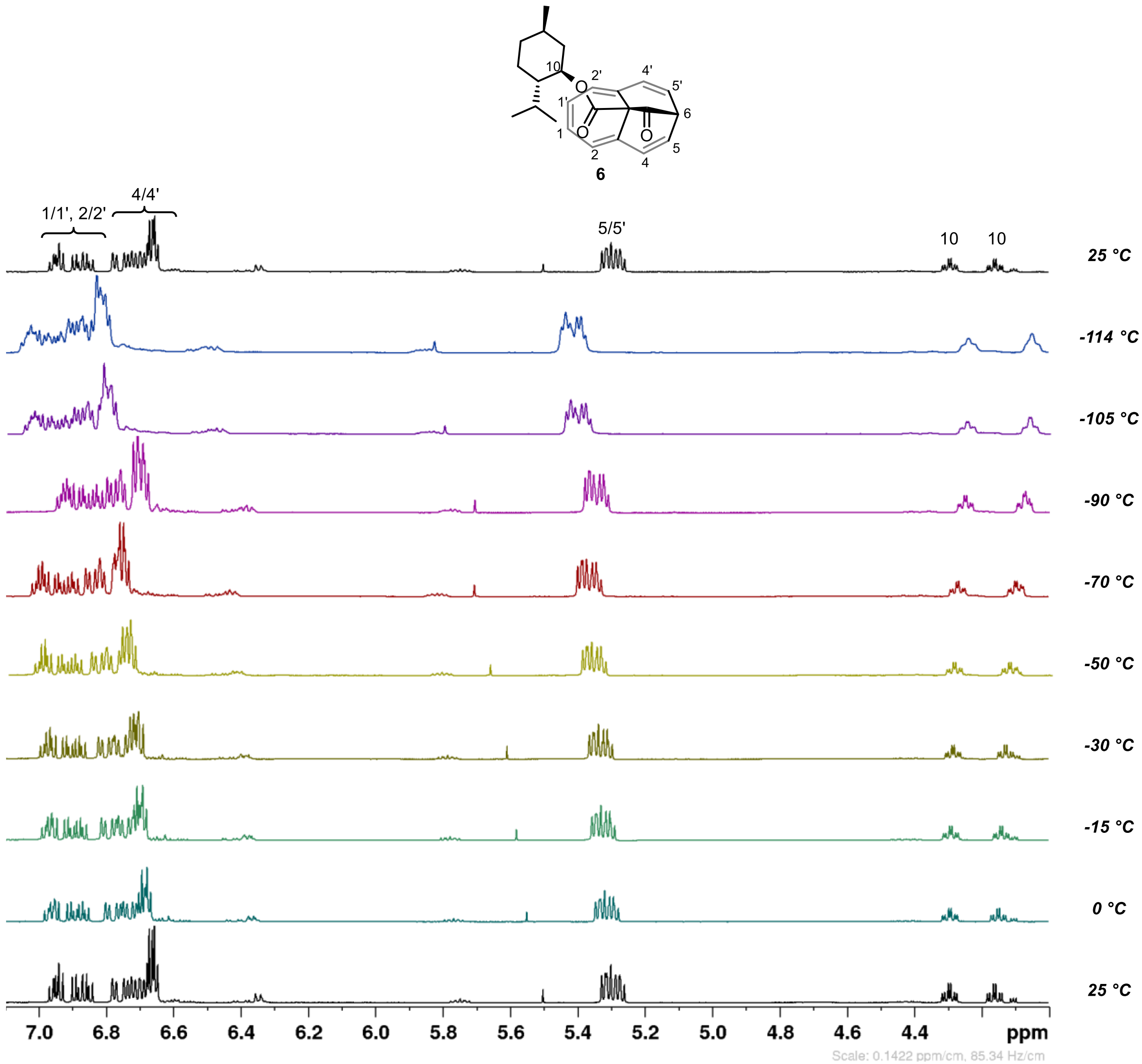

Figure S40. VT-NMR experiments in the range of 25 °C and –114 °C. Spectra are shown from δ = 4.0 to 7.2 ppm. Spectra were measured in THF-d_8 at 600 MHz..

1.8.2.4 Calculations on rotational barriers

The rotational barriers of homoannulene menthyl ester (L)-**6** were investigated computationally and experimentally. In homoannulene menthyl ester **6** various bond rotations were considered (Bond rotations A–C, Scheme S7). Bond rotation A depicts the bond rotation around the $C_{carbonyl}$–O_{ester} bond in homoannulene **6** (Equation A, Scheme S7). This rotation results in the two isomers with the ester being in s-*cis* (s-*cis* **6**) and s-*trans* (s-*trans* **6**) conformation, respectively. In the respective s-*cis* and s-*trans* conformation two more bond rotations can take place. First, the bond rotation around the $C_{carbonyl}$–$C_{bridgehead}$ bond, namely the rotation of the carboxyl group (Equations B, Scheme S7). In that, the s-*cis* and s-*trans* conformation is fixed. Second, in the respective s-*cis* and s-*trans* conformation a rotation around the O_{ester}–$C_{menthyl}$ bond is possible (Equations C, Scheme S7).

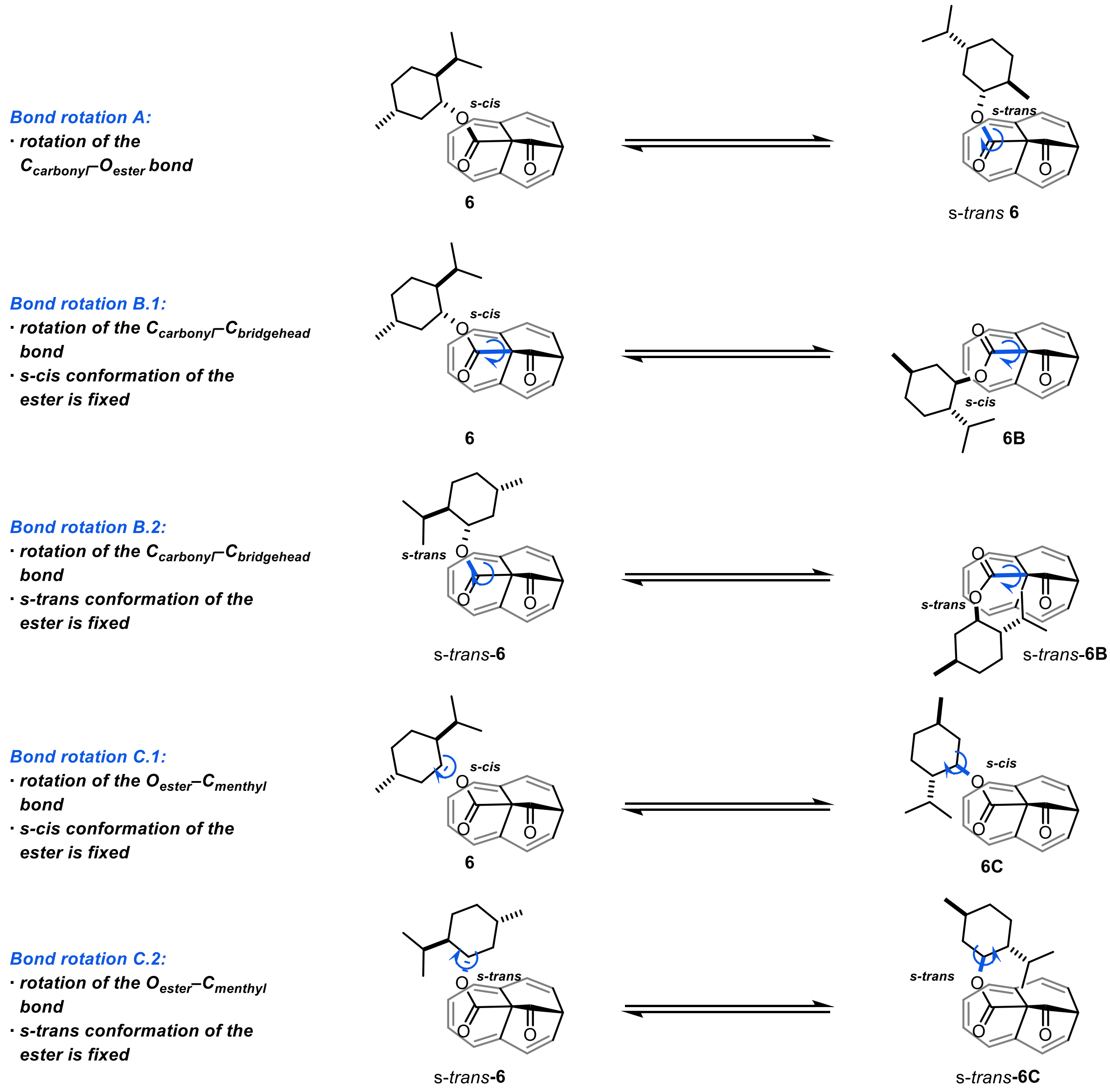

Scheme S7. Conceivable bond rotations in homoannulene esters **6**.

Scans of bond rotations were conducted (Figures S41, S42) using the TPSS-D3(BJ)/def2-SVP/IEFPCM($CHCl_3$) level of theory with a step size of 10°.

A rotation around the $C_{carbonyl}$–O_{ester} bond results in a minima structure in s-*cis* conformation and two minima structures in s-*trans* conformation (s-*trans*-**6** in Figure S41). The conformer lower in energy is depicted. The rotation of the menthyl substituent was observed together with the bond rotation of the $C_{carbonyl}$–O_{ester} bond. The calculations indicated that the s-*cis* conformation of the ester in s-*cis* **6** is lower in energy compared to the s-*trans* conformations s-*trans* **6** by $\Delta E_{\mathbf{6},2}$ = 39 kJ mol^{-1}. Barriers of $\Delta E^{\ddagger}_{\mathbf{6},1}$ = 58 kJ · mol^{-1} and $\Delta E^{\ddagger}_{\mathbf{6},2}$ = 50 kJ · mol^{-1} between the s-*cis* and s-*trans* conformations were determined. As the s-*trans* conformation of **6** was shown to be significantly less stable by $\Delta E_{\mathbf{6},1}$ = 39 kJ · mol^{-1} in the following calculations of bond rotation the ester was fixed in s-*cis* conformation (Equation B.1 and C.1 in Scheme

S7). The calculated barriers for this bond rotation are within the same range as for homoannulene methyl ester **4**.

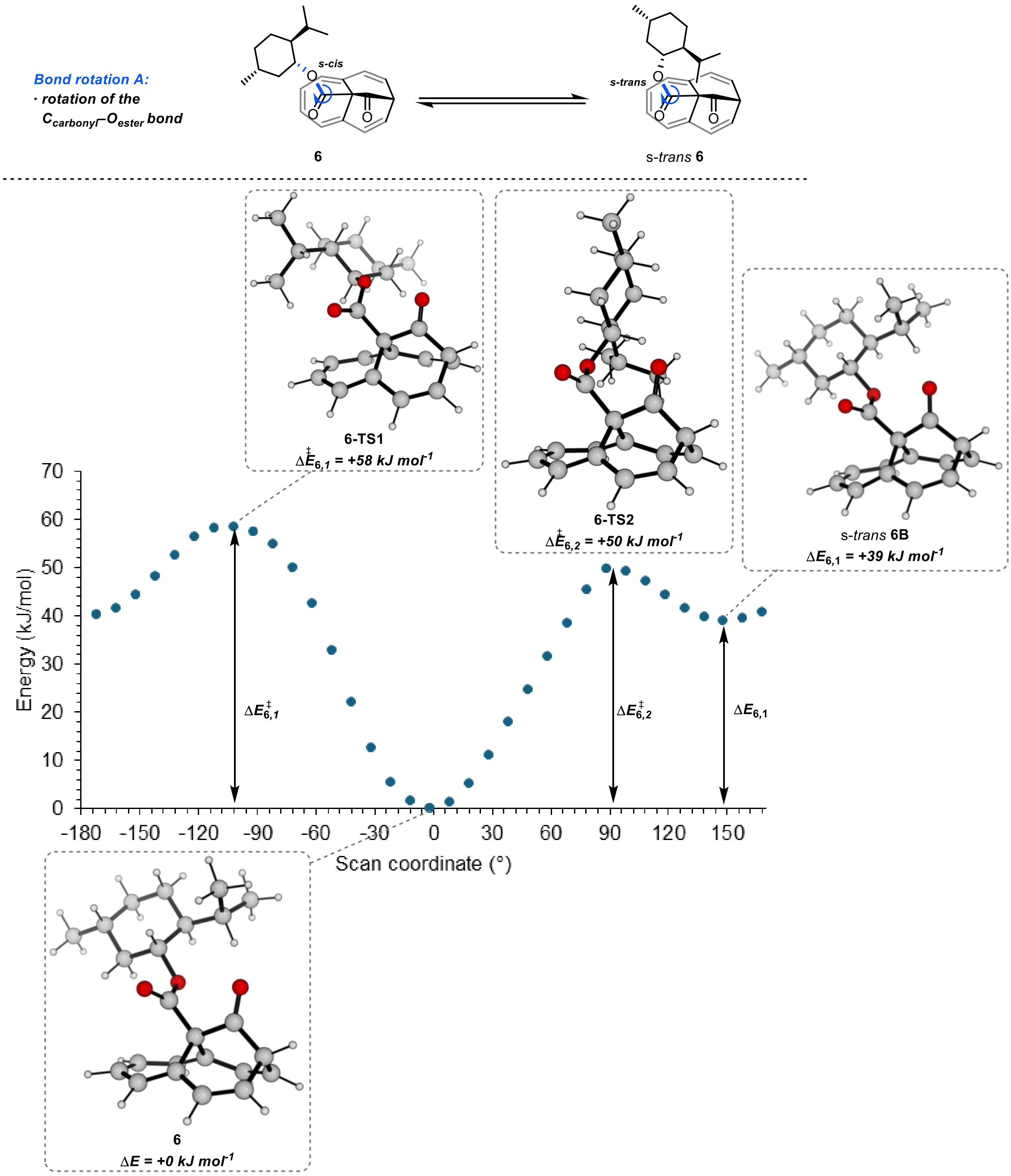

Figure S41. Scan of bond rotation $C_{carbonyl}$–C_{ester} in homoannulene menthyl ester (L)-**6**. Scan was conducted on the TPSS-D3(BJ)/def2-SVP/IEFPCM($CHCl_3$) level of theory with a step size of 10°.

A rotation around the $C_{carbonyl}$–$C_{bridgehead}$ bond results in two minima structures (**6** and **6B** in Figure S42), which are similar in energy. The barriers of rotations were calculated to be $\Delta E^{\ddagger}_{\mathbf{6},3}$ = 78 kJ · mol^{-1} and $\Delta E^{\ddagger}_{\mathbf{6},4}$ = 76 kJ · mol^{-1}. The lowest barrier for this bond rotation is higher in energy compared to the lowest bond rotation around the $C_{carbonyl}$–$C_{bridgehead}$ bond in homoannulene methyl ester **4** by ΔΔE = 9 kJ · mol^{-1} (see Section 1.8.1)

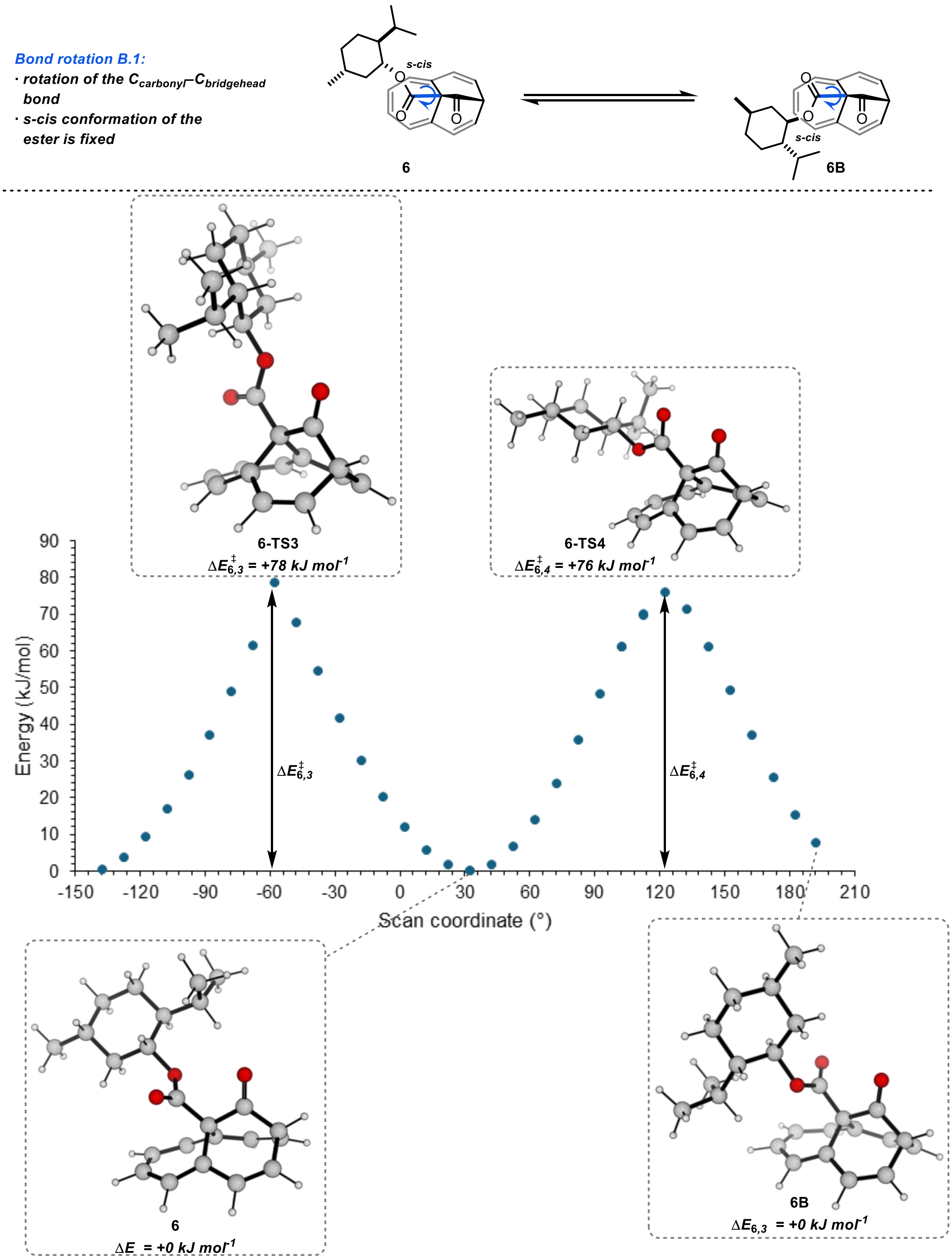

Figure S42. Scan of bond rotation $C_{carbonyl}$–$C_{bridgehead}$ in homoannulene menthyl ester (L)-**6**. Scan was conducted on the TPSS-D3(BJ)/def2-SVP/IEFPCM($CHCl_3$) level of theory with a step size of 10°.

Furthermore, a rotation of the O_{ester}–$C_{menthyl}$ bond in homoannulene ester **6** gives two minima structures (**6** and **6C**, Figure S43). The isomer **6C** is higher in energy by $\Delta E_{\mathbf{6},4}$ = 10 kJ · mol^{-1}. In this conformation the *iso*-propyl group of the menthyl alcohol moiety is pointing towards the 6π homoaromatic scaffold. In the more stable conformation, the menthyl group is directed

towards the 6π homoaromatic framework. The barriers connecting these two minima structures were determined to be $\Delta E^{\ddagger}_{6,6}$ = 53 kJ · mol^{-1} and $\Delta E^{\ddagger}_{6,6}$ = 29 kJ · mol^{-1}.

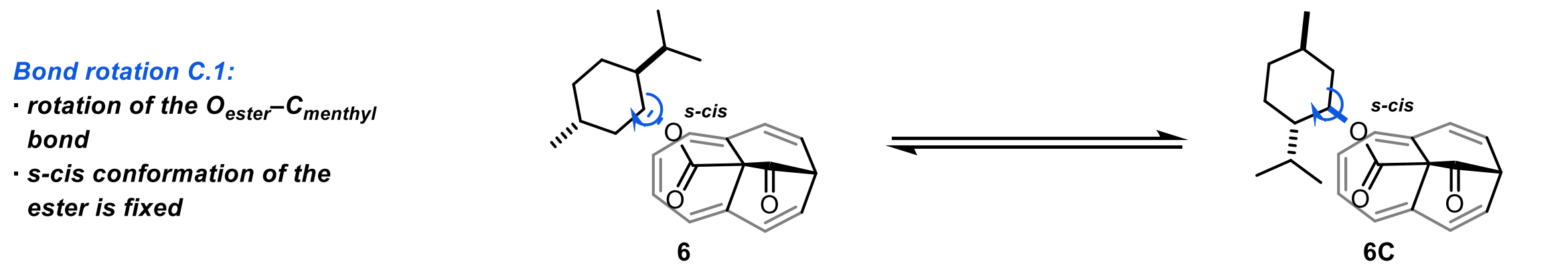

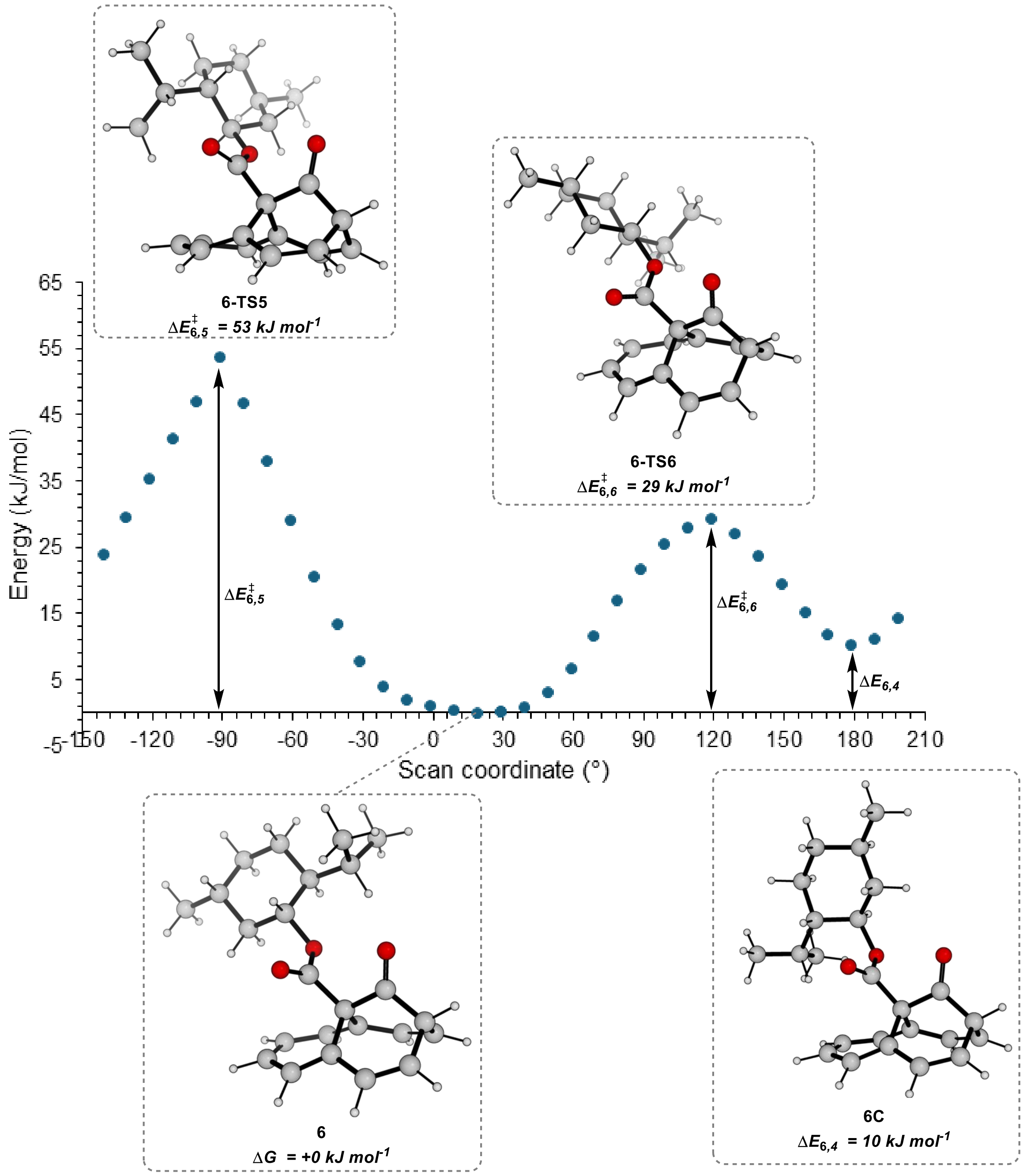

Figure S43. Scan of bond rotation O$_{ester}$–C$_{menthyl}$ in homoannulene menthyl ester (L)-**6**. Scan was conducted on the TPSS-D3(BJ)/def2-SVP/IEFPCM($CHCl_3$) level of theory with a step size of 10°.

1.8.3 Rotational barriers in *iso*-homoannulene menthyl esters (L)-7

The bond rotations that are conceivable in homoannulene menthyl esters (L)-**6** (see section 1.8) were considered for *iso*-homoannulene menthyl esters *(*L,*S)*-**7** and *(*L,*R)*-**7** as well. Next to experiments conducted to investigate the rotational barriers in esters **7** computations were carried out.

1.8.3.1 VT-NMR spectra

Iso-homoannulenes (L)-**7** were isolated as a mixture after irradiation and ^{1}H NMR spectra in *o*-dichlorobenzene-d_4 in a range from 25 °C to 130 °C were detected (Figure S44). Upon heating the sample no coalescence of NMR signals was detected neither in the (homo-)aromatic region, nor in the region of the NMR spectrum in which signals of the menthyl substituent are detected. With that it can be rationalized, that the rotational barriers of *iso*-homoannulenes **7** are below room temperature and too fast for the timescale of NMR. This is in stark contrast to the parent menthyl homoannulene ester **6**, for which the highest barrier of rotation was detected at a temperature of 100–110 °C which gives an estimated rotational barrier of about $\Delta G^{\ddagger}$ = 77 kJ · mol^{-1}.

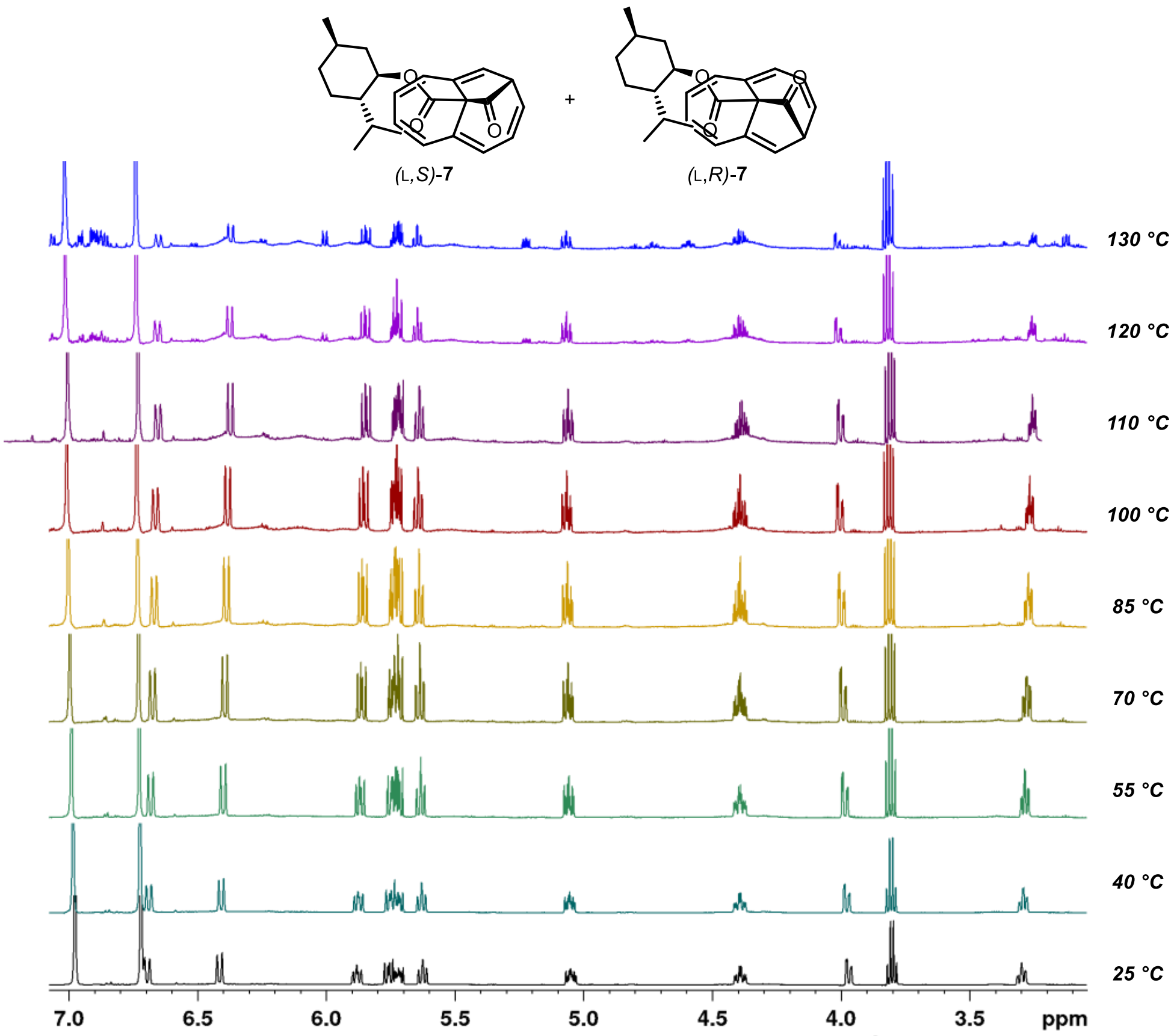

Figure S44. Spectra of VT-NMR experiments of (L)-**7** in the range of 25 °C and 130 °C. Spectra are shown from δ = 3.0–7.0 ppm. Spectra were measured in *o*-dichlorobenzene-d_4 at 600 MHz.

Additionally, VT-NMR experiments were conducted in THF-d_8 in the temperature range from 25 °C until –110 °C (Figure S45 and S46). A point of coalescence was detected for hydrogen atom H-10 at δ = 7.0 ppm of the *iso*-homoannulene scaffold at –60 °C (red box in Figure S45). Furthermore, at –50 °C a point of coalescence of H-5 was detected (red box in Figure S46). Compared to the ^{1}H NMR spectra detected of homoannulene **6** the highest barrier of rotation was estimated to be about $\Delta G^‡$ = 45 kJ · mol^{-1}.

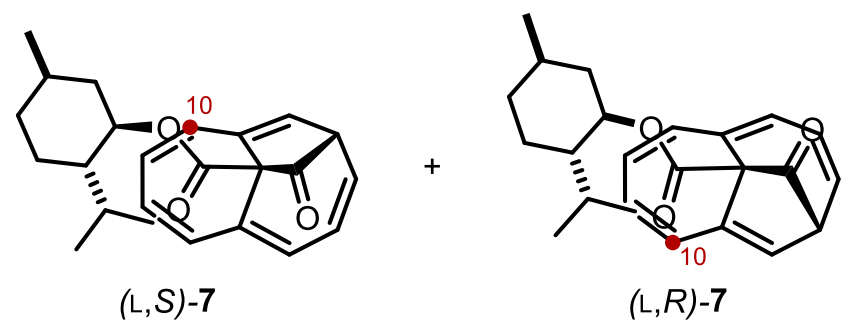

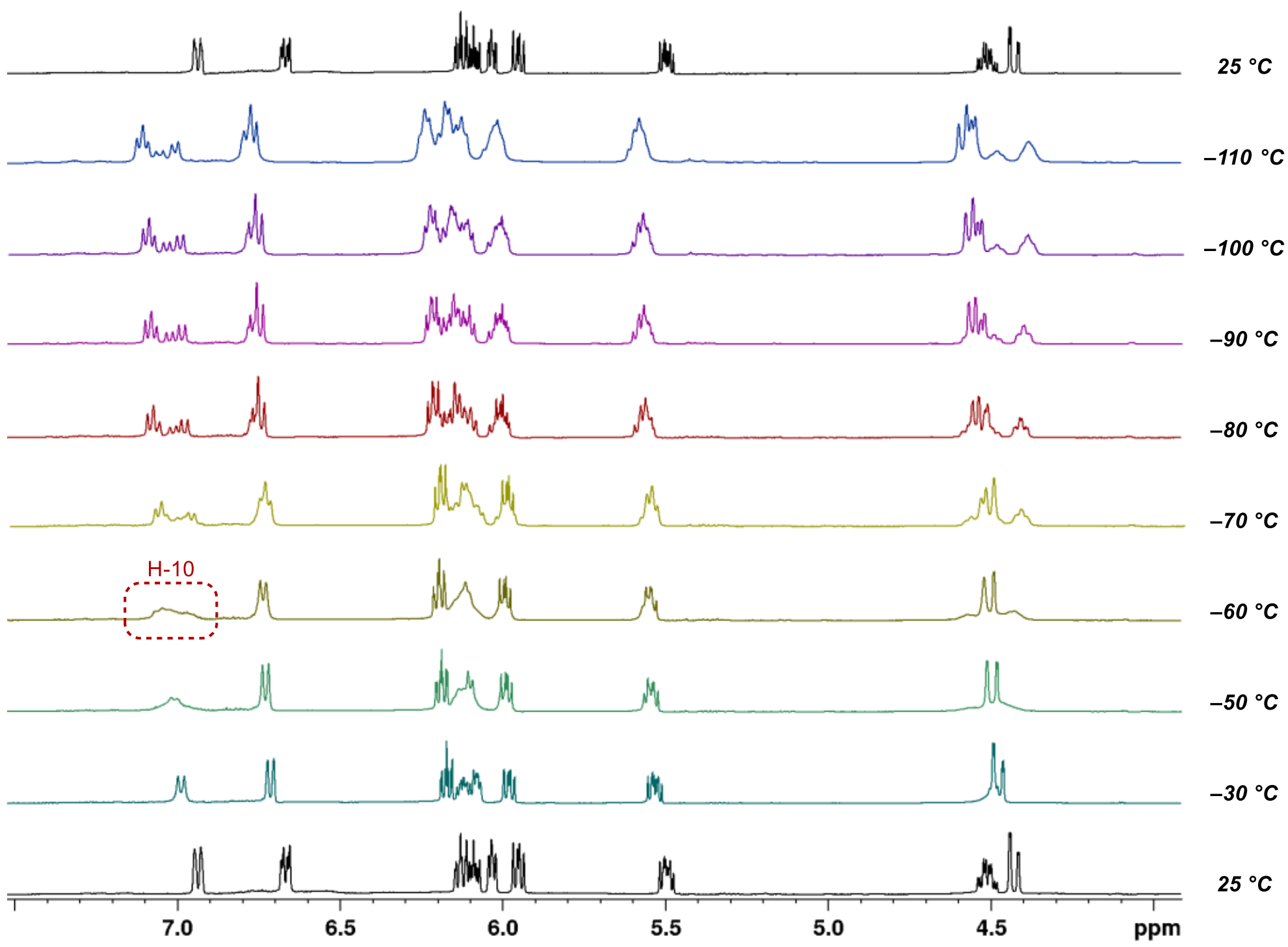

Figure S45. Spectra of VT-NMR experiments of (L)-**7** in the range of 25 °C and –110 °C. Spectra are shown from δ = 4.0–7.5 ppm. Spectra were measured in THF-d_8 at 600 MHz.

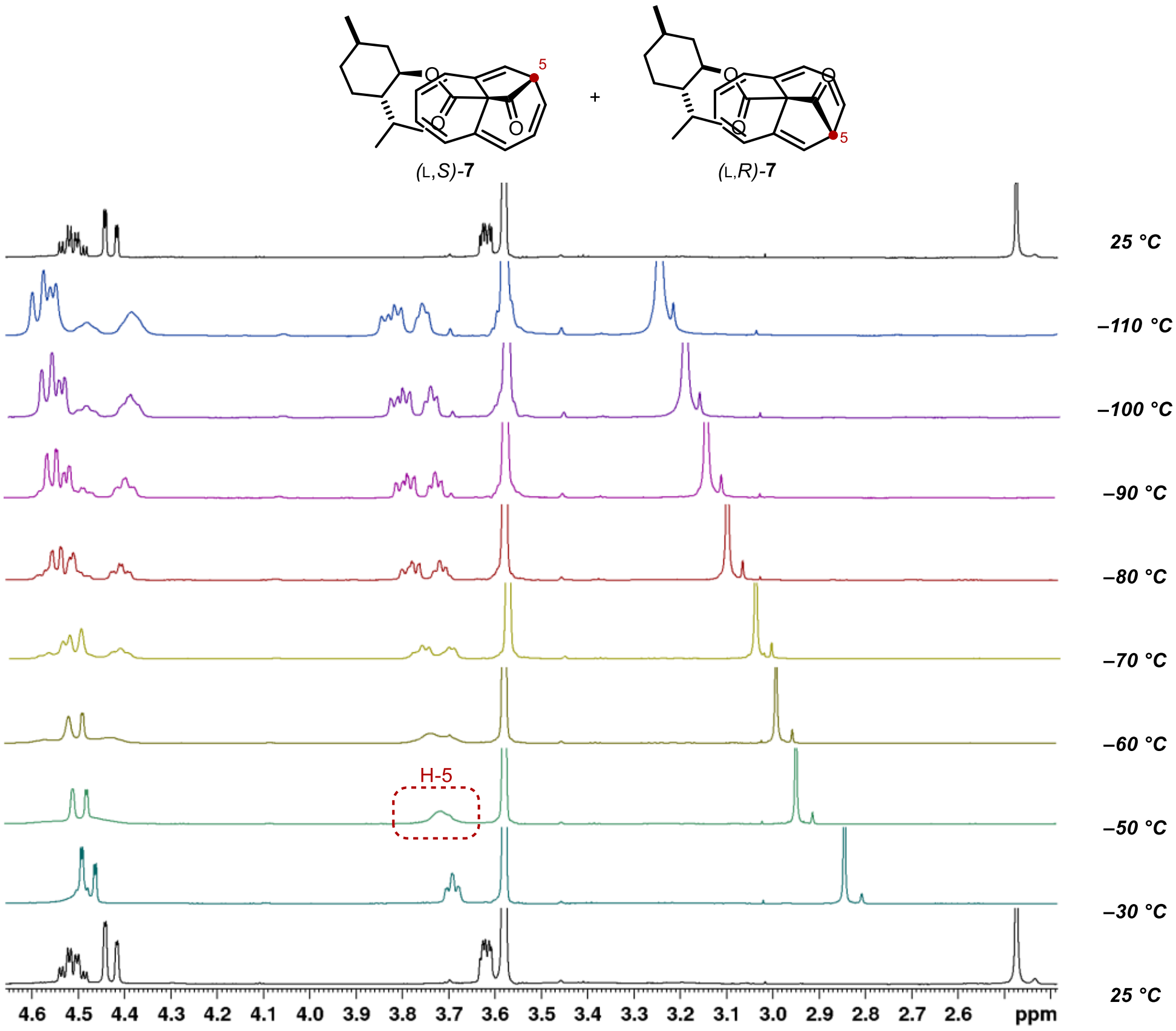

Figure S46. Spectra of VT-NMR experiments of *iso*-homoannulenes (L)-**7** in the range of 25 °C and –110 °C. Spectra are shown from δ = 2.4–4.7 ppm. Spectra were measured in THF-d_8 at 600 MHz.

1.8.3.2 Calculations on rotational barriers

For the two diastereomeric *iso*-homoannulenes *(*L,*S)*-**7** and *(*L,*R)*-**7** the rotational barriers were calculated using the TPSS-D3(BJ)/def2-SVP/IEFPCM($CHCl_3$) level of theory with a step size of 10° for every bond rotation considered. The same bond rotations as in homoannulene menthyl ester **6** were considered (see Scheme S7), namely the rotation around the $C_{carbonyl}$–O_{ester} bond (Equation A, Scheme S7), the bond rotation around the $C_{carbonyl}$–$C_{bridgehead}$ bond (Equation B, Scheme S7), rotation of the carboxyl group and finally the rotation around the O_{ester}–$C_{menthyl}$ bond (Equation C, Scheme S7). Bond rotations depicted in equations B and C can occur in the s-*trans* and s-*cis* conformation of the ester moiety.

*Iso-homoannulene (*L*,S)-**7***

A relaxed rotation of the $C_{carbonyl}$–O_{ester} bond was performed. Two minima structures in s-*cis* and one minimum structure in s-*trans* conformation were determined. Two minima corresponding to s-*cis* conformations and one minimum corresponding to an s-*trans* conformation were identified. The two s-*cis* structures differ in a manner that the

$C_{carbonyl}$–O_{ester}-bond rotation is complemented by a reorientation of the menthyl substituent ((L,*S*)-**7** compared to (L,*S*)-**7-2** in Figure S47). The calculations indicate that the s-*cis* conformation of (L,*S*)-**7** is energetically favored compared to the s-*trans* conformation by $\Delta E_{(L,S)\text{-}\mathbf{7},1}$ = 36 kJ mol^{-1}. Barriers of $\Delta E^{\ddagger}_{(L,S)\text{-}\mathbf{7}1}$ = 50 kJ · mol^{-1} and $\Delta E^{\ddagger}_{(L,S)\text{-}\mathbf{7},2}$ = 52 kJ · mol^{-1} were determined for interconversion between the s-*cis* and s-*trans* conformations. As the s-*trans* conformation of (L,*S*)-**7** was found to be significantly less stable, the ester moiety was constrained in the s-*cis* conformation for subsequent scans of rotation the $C_{carbonyl}$–$C_{bridgehead}$ bond and O_{ester}–$C_{menthyl}$ bond (compare Equation B.1 and C.1 in Scheme S7). The energy of the conformer in s-*trans* conformation as well as the connecting barriers are within the same range as calculated for homoannulene methyl ester **4** (see Figure S34) and homoannulene menthyl ester (L)-**6** (Figure S41).

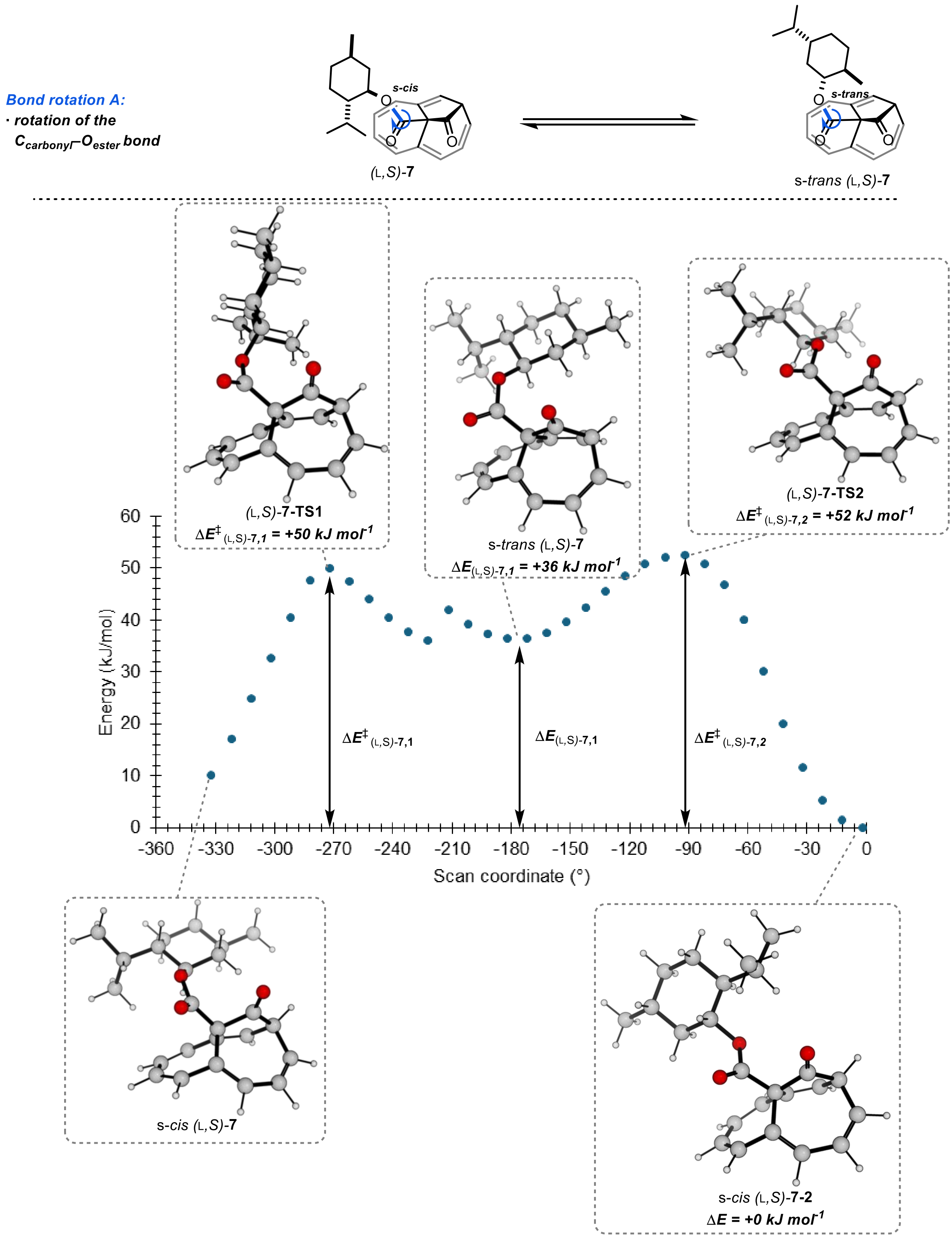

Figure S47. Scan of bond rotation $C_{carbonyl}$–C_{ester} in *iso*-homoannulene menthyl ester (L,*S*)-**7**. Scan was conducted on the TPSS-D3(BJ)/def2-SVP/IEFPCM($CHCl_3$) level of theory with a step size of 10°.

In the relaxed scan of this bond rotation, a release of strain is observed along the scan coordinate between −210° and −220° (Figure S48). At −210° on the scan coordinate axis, the isopropyl substituent of the menthyl moiety is positioned directly above the *iso*-homoannulene scaffold, resulting in significant steric congestion. Upon further rotation about the $C_{carbonyl}$–O_{ester} bond, the steric demand prevents the *iso*-propyl group from approaching the scaffold more closely. Consequently, the menthyl moiety reorients away from the underlying homoaromatic framework, thereby relieving steric strain and lowering the overall energy.

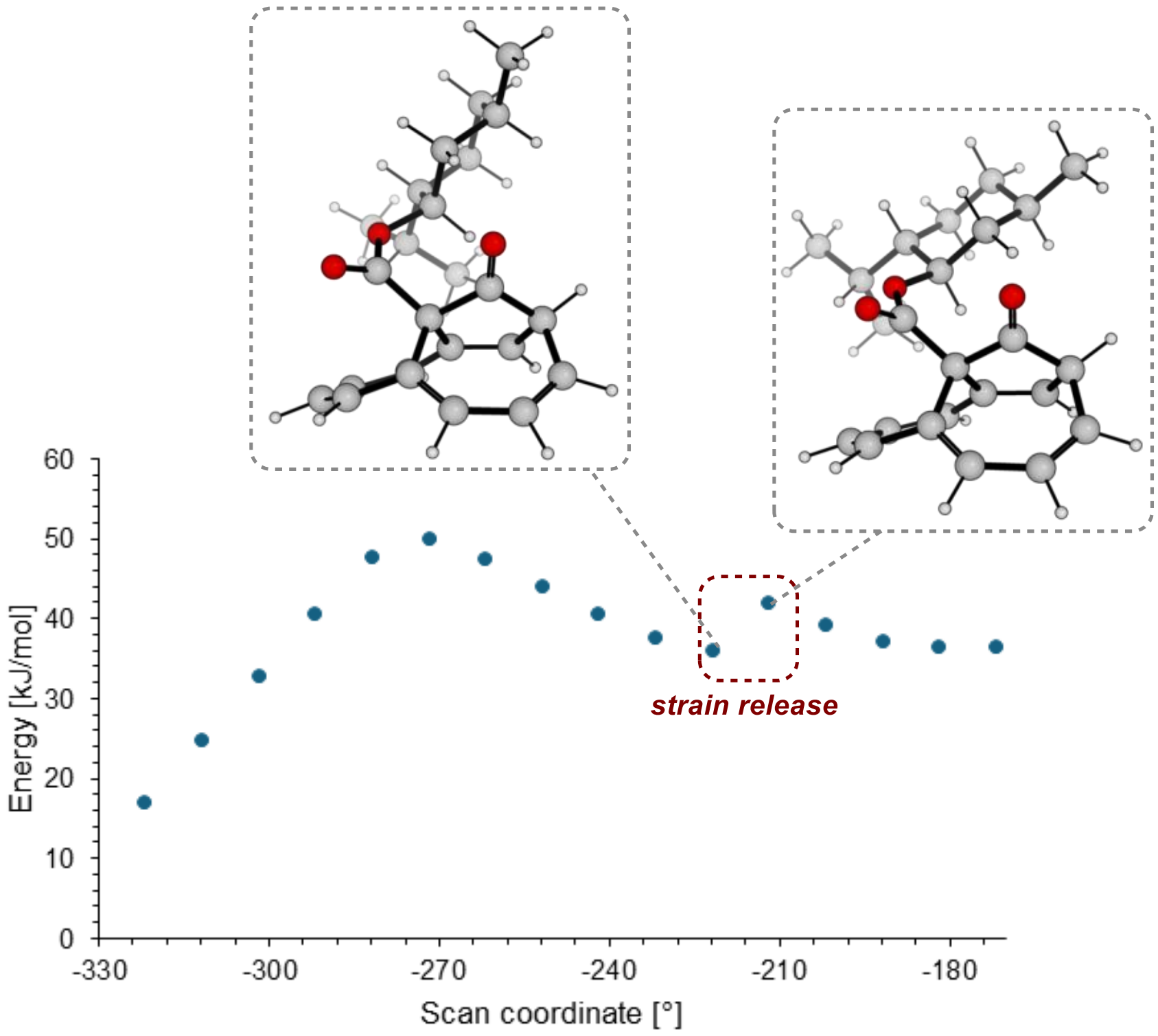

Figure S48. Strain release in *(*L*,S)*-**7** in bond rotation of the $C_{carbonyl}$–C_{ester} bond.

A rotation around the $C_{carbonyl}$–$C_{bridgehead}$ bond results in two minima structures ((L,*S)*-**7** and ((L,*S)*-**7B** in Figure S49) which are similar in energy. The barriers of rotations were calculated to be $\Delta E^{\ddagger}_{(L,S)\text{-}\mathbf{7},3}$ and $\Delta E^{\ddagger}_{(L,S)\text{-}\mathbf{7},4}$ = 43 kJ · mol^{-1} respectively. This value is in comparison to homoannulene ester (L)-**6** lower by $\Delta\Delta E^{\ddagger}$ =33 kJ · mol^{-1} (compare Figure S42) and within the same range as the barrier of bond rotation which was determined experimentally (see Section 1.8.3.1).

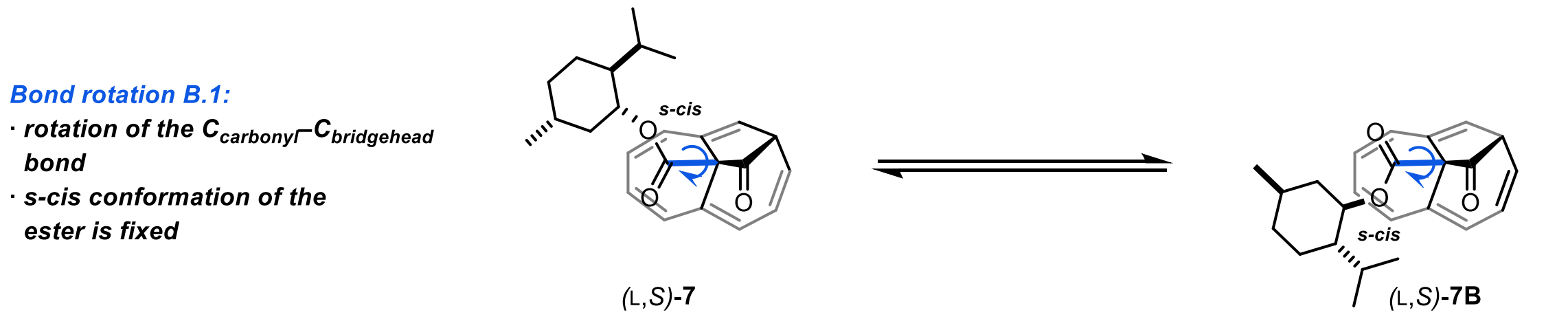

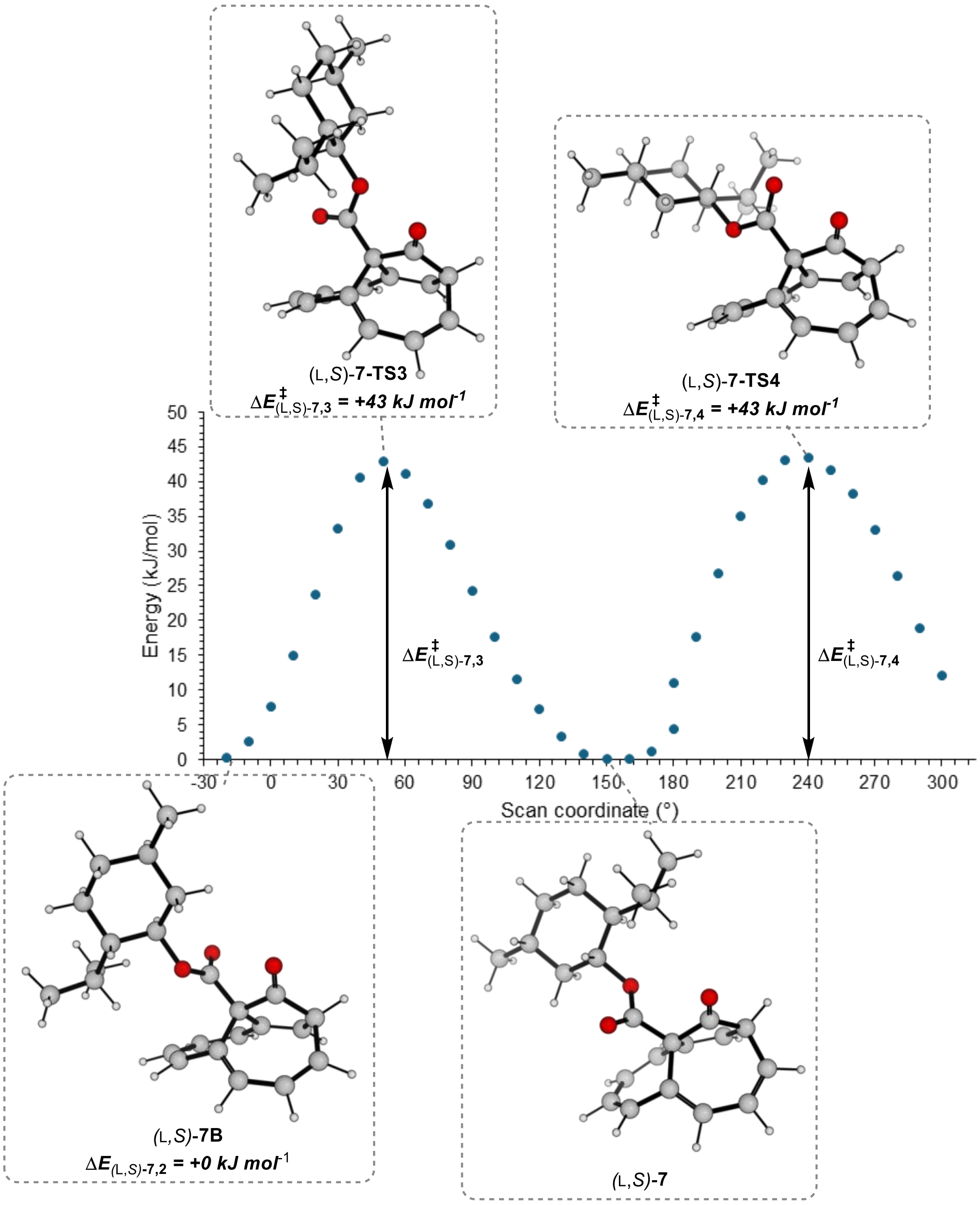

Figure S49. Scan of bond rotation $C_{carbonyl}$–$C_{bridgehead}$ in *iso*-homoannulene (L,*S*)-**7**. Scan was conducted on the TPSS-D3(BJ)/def2-SVP/IEFPCM($CHCl_3$) level of theory with a step size of 10°.

Furthermore, a rotation around the O_{ester}–$C_{menthyl}$ bond in *iso*-homoannulene ester (L,*S*)-**7** results in two minima structures ((L,*S*)-**7** and (L,*S*)-**7C**, Figure S50). The isomer (L,*S*)-**7C** is higher in energy by $\Delta E_{(L,S)\text{-}7,4}$ = 8 kJ · mol^{-1}. In this conformation the *iso*propyl group of the menthyl alcohol moiety is pointing towards the 10π homoaromatic scaffold. In the more stable conformation (L,*S*)-**7**, the menthyl group is directed towards the 10π homoaromatic framework.

The barriers connecting these two minima structures were determined to be $\Delta E^{\ddagger}_{(\text{L},S)\text{-}\mathbf{7},5}$ = 52 kJ · mol^{-1} and $\Delta E^{\ddagger}_{(\text{L},S)\text{-}\mathbf{7},6}$ = 28 kJ · mol^{-1}. These barriers are comparable to the values calculated for the rotation of O_{ester}–$C_{menthyl}$ bond in homoannulene ester **6** (compare Figure S43).

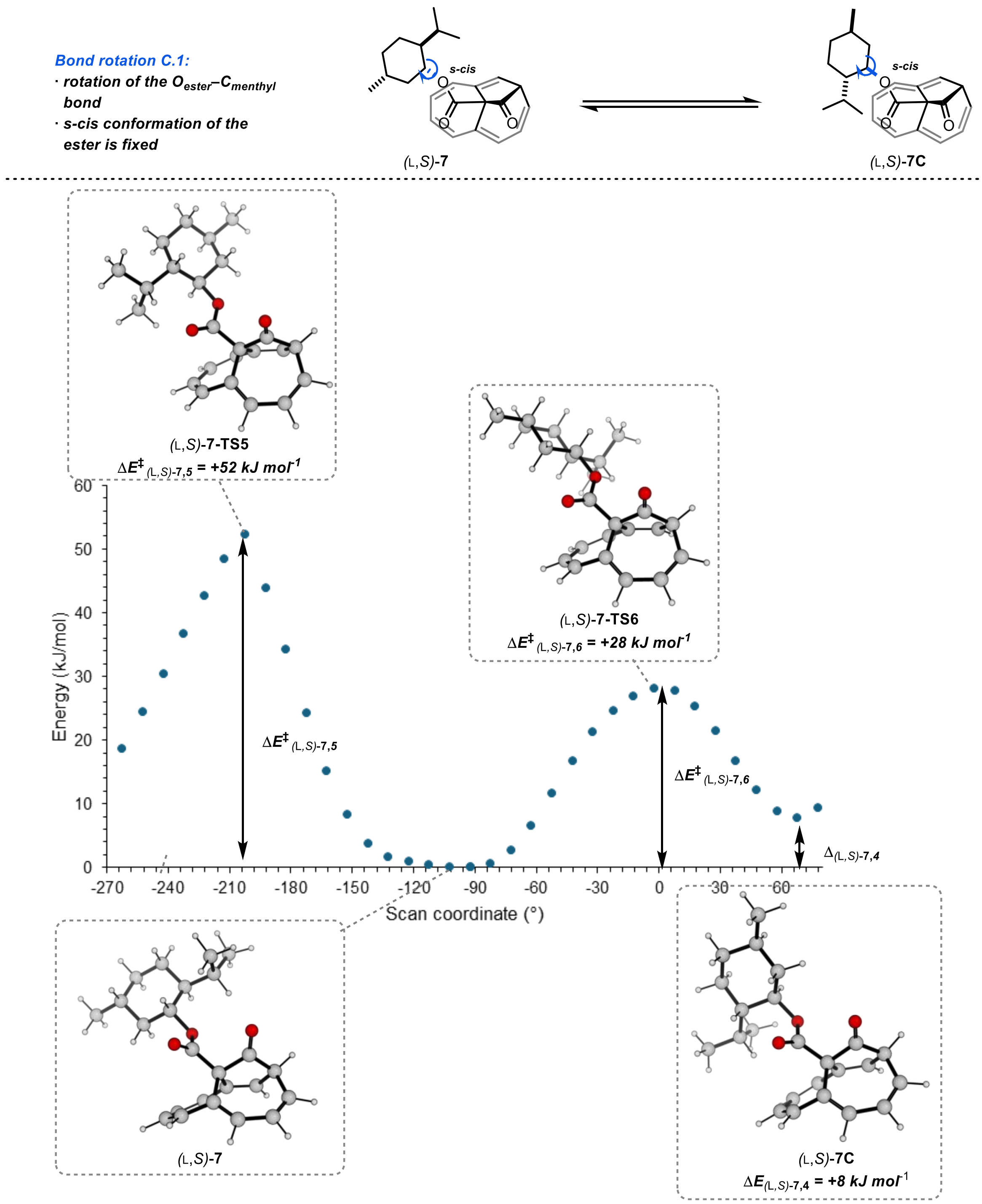

Figure S50. Scan of bond rotation O_{ester}–$C_{menthyl}$ in *iso*-homoannulene menthyl ester *(*L*,S)*-**7**. Scan was conducted on the TPSS-D3(BJ)/def2-SVP/IEFPCM($CHCl_3$) level of theory with a step size of 10°.

Iso-homoannulene ester ***(*L*,R)*-7**

A relaxed rotation of the $C_{carbonyl}$–O_{ester} bond was performed. One minimum structure in s-*cis* and one minimum structure in s-*trans* conformation were determined ((L,*R)*-**7** and s-*trans* (L,*R)*-**7** in Figure S51). The calculations indicate that the s-*cis* conformation of (L,*R)*-**7** is energetically favored over the s-*trans* conformation by $\Delta E_{(L,R)\text{-}7,1}$ = 36 kJ mol^{-1}. Barriers of $\Delta E^{\ddagger}_{(L,R)\text{-}7,1}$ = 54 kJ · mol^{-1} and $\Delta E^{\ddagger}_{(L,R)\text{-}7,2}$ = 51 kJ · mol^{-1} were determined for interconversion between the s-*cis* and s-*trans* conformations. As the s-*trans* conformation of (L,*R)*-**7** was found to be significantly less stable, the ester moiety was constrained in the *s-cis* conformation for subsequent scans of rotation the $C_{carbonyl}$–$C_{bridgehead}$ bond and O_{ester}–$C_{menthyl}$ bond (compare Equation B.1 and C.1 in Scheme S7). The barriers of this bond rotation as well as the energy difference of s-*trans* conformers to s-*cis* conformers is within the same range as for *iso*-homoannulene (L,*S)*-**7** (compare Figure S47).

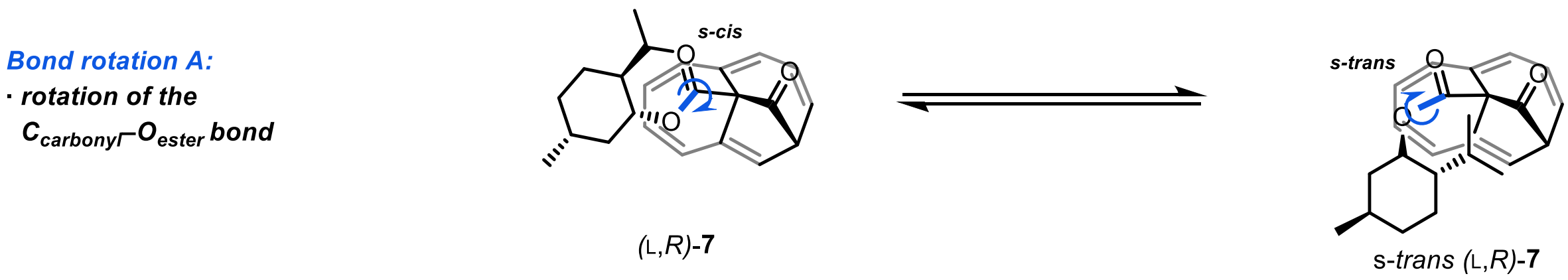

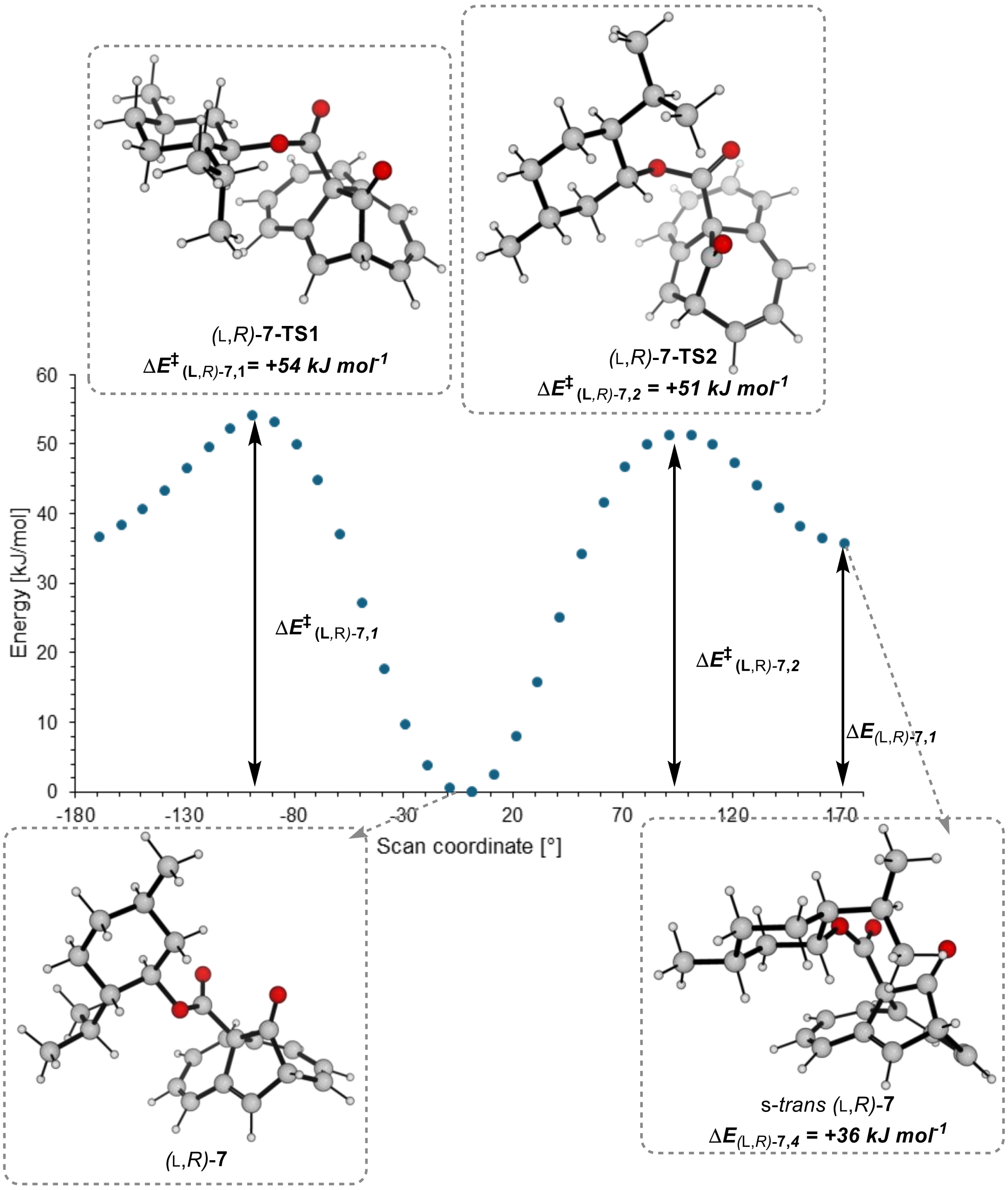

Figure S51. Scan of bond rotation C$_{carbonyl}$–C$_{ester}$ in *iso*-homoannulene menthyl ester *(*L,*R)*-**7**. Scan was conducted on the TPSS-D3(BJ)/def2-SVP/IEFPCM(CHCl$_3$) level of theory with a step size of 10°.

A rotation around the C$_{carbonyl}$–C$_{bridgehead}$ bond results in two minima structures (*(*L,*R)*-**7** and *(*L,*R)*-**7B** in Figure S52), which are similar in energy. The barriers of rotations were calculated to be $\Delta E^{\ddagger}_{(L,R)\text{-}7,3}$ = 47 kJ · mol^{-1} and $\Delta E^{\ddagger}_{(L,R)\text{-}7,4}$ = 45 kJ · mol^{-1}, which is in the same range as for *iso*-homoannulene menthyl ester *(*L,*S)*-**7** (compare: $\Delta E^{\ddagger}_{(L,S)\text{-}7,3/4}$ = 43 kJ · mol^{-1}). This value is in comparison to homoannulene ester **6** lower by $\Delta\Delta E^{\ddagger}$ = 31 kJ · mol^{-1} (compare Figure S42).

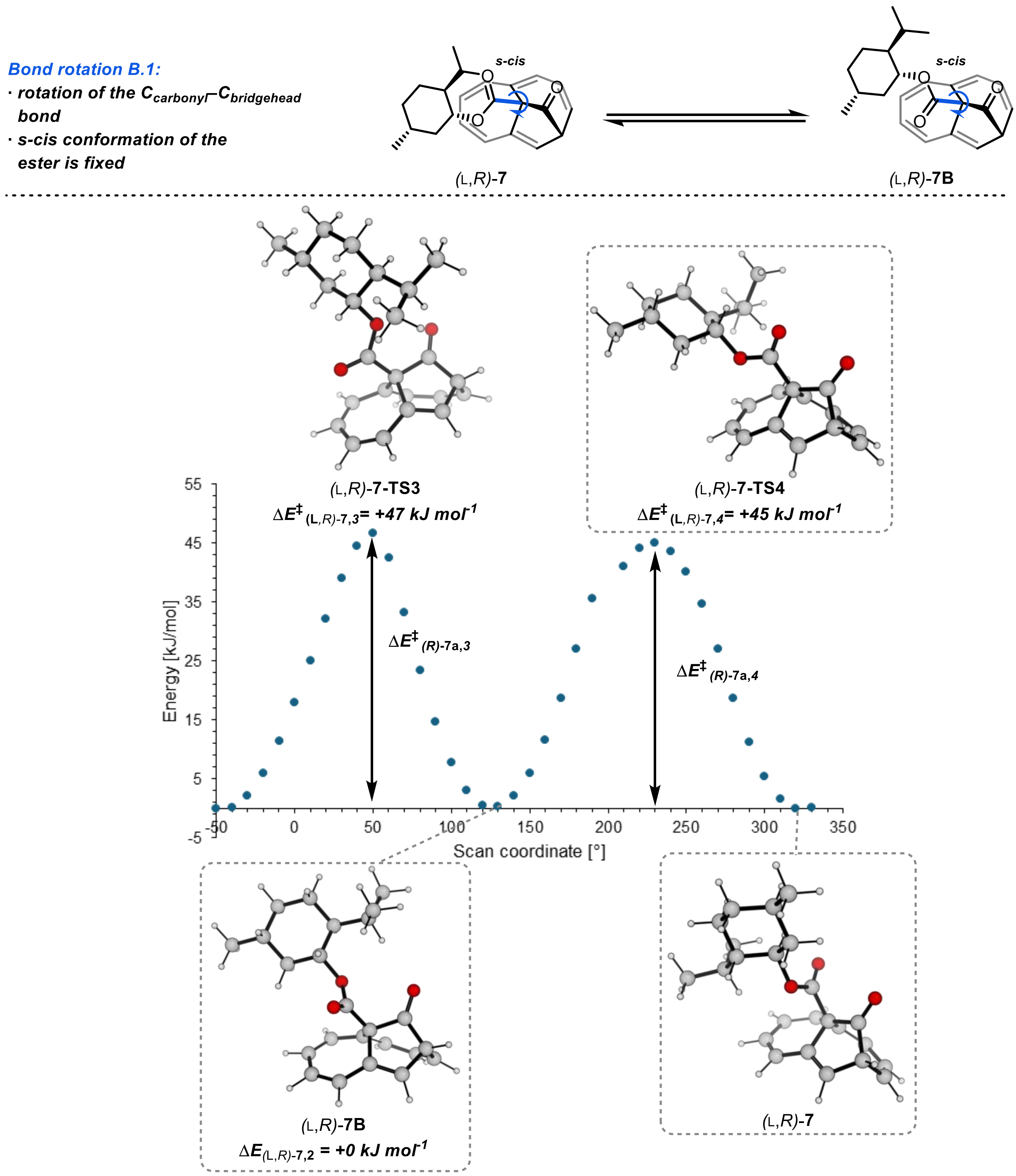

Figure S52. Scan of bond rotation $C_{carbonyl}$–$C_{bridgehead}$ in *iso*-homoannulene menthyl ester (L,*R*)-**7**. Scan was conducted on the TPSS-D3(BJ)/def2-SVP/IEFPCM($CHCl_3$) level of theory with a step size of 10°.

Furthermore, a rotation around the O_{ester}–$C_{menthyl}$ bond in *iso*-homoannulene ester (L,R)-**7** results in two minima structures ((L,*R*)-**7** and (L,*R*)-**7C**, Figure S53). The isomer (L,*R*)-**7C** is higher in energy by $\Delta E_{(L,R)-7,4}$ = +10 kJ · mol^{-1}. In this conformation the methyl group of the menthyl alcohol moiety is pointing towards the 10π homoaromatic scaffold. Whereas in the more stable conformation (L,*R*)-**7**, both the methyl and *iso*propyl group are located distant from the underlying homoaromatic 10π framework. The barriers connecting these two minima

structures were determined to be $\Delta E^{\ddagger}_{(L,R)\text{-}7,5}$ = 56 kJ · mol⁻¹ and $\Delta E^{\ddagger}_{(L,R)\text{-}7,6}$ = 30 kJ · mol⁻¹. These barriers are comparable to the values calculated for the rotation of O_{ester}–$C_{menthyl}$ bond in homoannulene ester **6** (compare Figure S43) and *iso*-homoannulene menthyl ester *(*L*,S)*-**7** (compare Figure S50).

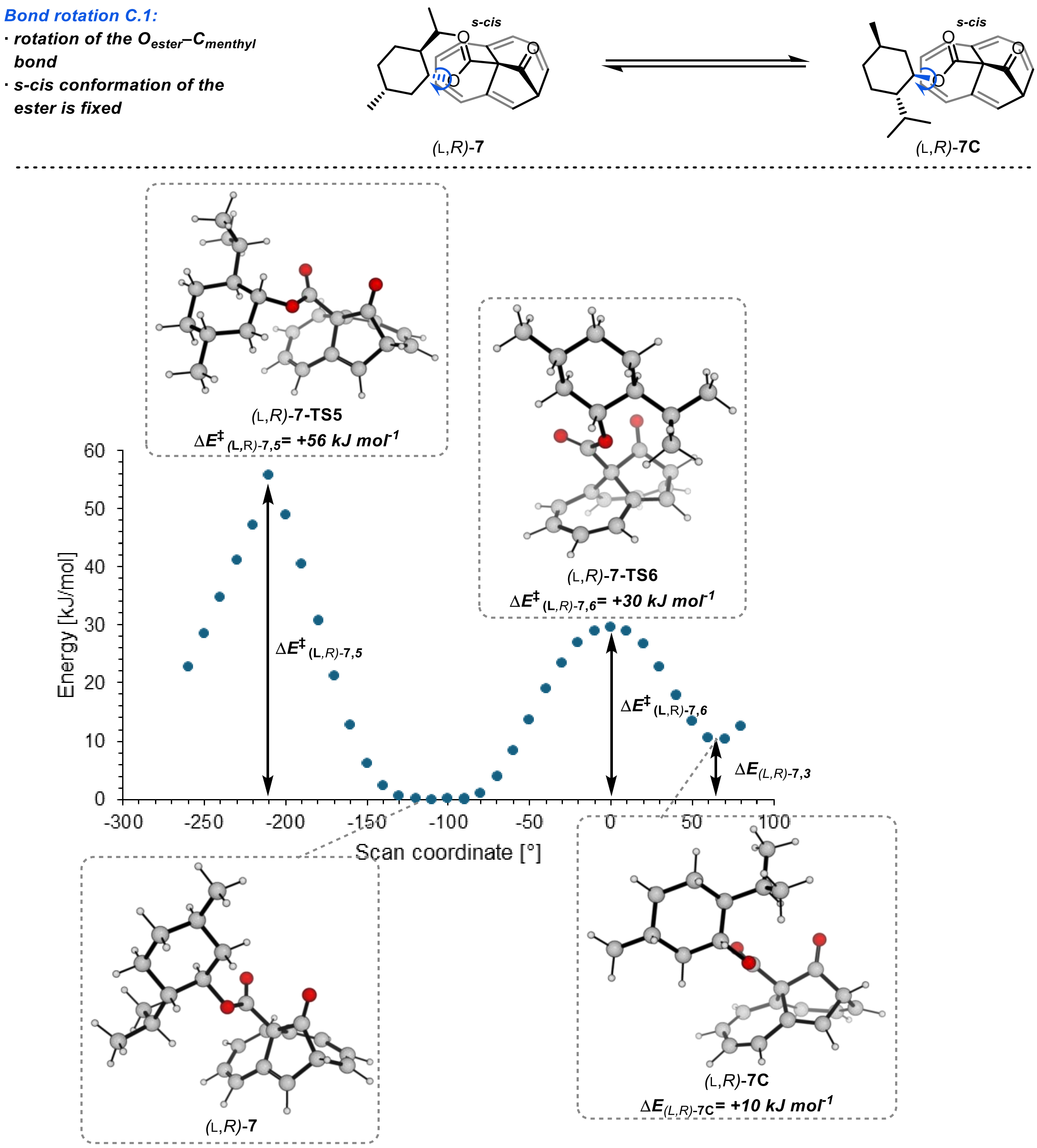

Figure S53. Scan of bond rotation O_{ester}–$C_{menthyl}$ in *iso*-homoannulene menthyl ester *(*L*,R)*-**7**. Scan was conducted on the TPSS-D3(BJ)/def2-SVP/IEFPCM($CHCl_3$) level of theory with a step size of 10°.

In summary the calculations of bond rotations in homoannulene ester **4**, (L)-**6** as well as *iso*-homoannulene esters (L,*S)*-**7** and *(L,R)*-**7** the rotation around the $C_{carbonyl}$–$C_{bridgehead}$ bond is in

a comparable range as the barrier which was determined experimentally by VT-NMR studies. Notably, the highest barrier detected for homoannulene methyl ester **4** is lower in energy as the one for homoannulene menthyl ester (L)-**6** by about $\Delta\Delta E = 9\ kJ\ mol^{-1}$. However, the highest rotational barrier for *iso*-homoannulenes *(*L*,S)*-**7** was found to be significantly lower by about $\Delta\Delta E = 31\ kJ \cdot mol^{-1}$ than the one in the parent homoannulene menthyl ester (L)-**6**, which rationalizes that no AA'XX' coupling system detected in ^1H NMR spectra of *iso*-homoannulene menthyl ester (L)-**7**.

1.9 Crystallographic data

Crystal structures of the asteranone (D)-**12** and homoannulene ester (L)-**6** were measured and the crystal structures were deposited *via* the joint CCDC/FIZ Karslruhe deposition service ((D)-**12**: CCDC 2531740; (L)-**6**: CCDC 2531744).

The following data were obtained for the X-ray single crystal measurement of asterane (D)-**12**: a clear colourless, plate-like specimen of $C_{24}H_{30}O_3$, approximate dimensions 0.060 mm x 0.100 mm x 0.230 mm, was used for the X-ray crystallographic analysis. The X-ray intensity data were measured (λ = 1.54178 Å). A total of 9516 frames were collected. The total exposure time was 8.14 hours. The frames were integrated with the Bruker SAINT software package using a narrow-frame algorithm. The integration of the data using a monoclinic unit cell yielded a total of 31193 reflections to a maximum θ angle of 74.40° (0.80 Å resolution), of which 4022 were independent (average redundancy 7.756, completeness = 99.9%, Rint = 3.29%, Rsig = 2.04%) and 3974 (98.81%) were greater than 2σ(F2). The final cell constants of a = 6.2917(2) Å, b = 9.9335(3) Å, c = 16.0822(4) Å, β = 99.6070(10)°, volume = 991.02(5) $Å^3$, are based upon the refinement of the XYZ-centroids of 9796 reflections above 20 σ(I) with 14.27° < 2θ < 149.0°. Data were corrected for absorption effects using the Multi-Scan method (SADABS). The ratio of minimum to maximum apparent transmission was 0.849. The calculated minimum and maximum transmission coefficients (based on crystal size) are 0.8700 and 0.9640. The structure was solved and refined using SHELXT 2018/2[39] of the Bruker SHELXTL Software Package, using the space group P 1 21 1, with Z = 2 for the formula unit, $C_{24}H_{30}O_3$. The final anisotropic full-matrix least-squares refinement on F2 with SHELXL-2013[40] with 247 variables converged at R1 = 2.88%, for the observed data and wR2 = 7.60% for all data. The goodness-of-fit was 1.029. The largest peak in the final difference electron density synthesis was 0.225 e-/Å3 and the largest hole was -0.153 $e^-/Å^3$ with an RMS deviation of 0.031 $e^-/Å^3$. On the basis of the final model, the calculated density was 1.228 g/cm^3 and F(000), 396 e^-.

The following data were obtained for the X-ray single crystal measurement of asterane (L)-**6**: a colorless, plate-like specimen of $C_{24}H_{28}O_3$, approximate dimensions 0.040 mm x 0.090 mm x 0.220 mm, was used for the X-ray crystallographic analysis. The X-ray intensity data were measured (λ = 1.54178 Å). A total of 1730 frames were collected. The total exposure time was 31.60 h. The frames were integrated with the Bruker SAINT software package using a narrow-frame algorithm. The integration of the data using an orthorhombic unit cell yielded a total of 85785 reflections to a maximum θ angle of 67.00° (0.84 Å resolution), of which 15093 were independent (average redundancy 5.684, completeness = 100.0%, Rint = 12.14%, Rsig = 7.46%) and 10957 (72.60%) were greater than 2σ(F2). The final cell constants of a = 14.3194(5) Å, b = 15.6982(5) Å, c = 37.7074(11) Å, volume = 8476.2(5) $Å^3$, are based upon the refinement of the XYZ-centroids of 9905 reflections above 20 σ(I) with 7.752° < 2θ < 140.1°. Data were corrected for absorption effects using the Multi-Scan method (SADABS). The ratio of minimum to maximum apparent transmission was 0.786. The calculated minimum and maximum transmission coefficients (based on crystal size) are 0.8750 and 0.9750. The structure was solved and refined using SHELXT 2018/2[39] of the Bruker SHELXTL Software Package, using the space group P 21 21 21, with Z = 16 for the formula unit, $C_{24}H_{28}O_3$. The final anisotropic full-matrix least-squares refinement on F2 with SHELXL-2013[40] with 985 variables converged at R1 = 5.86%, for the observed data and wR2 = 15.82% for all data. The goodness-of-fit was 1.074. The largest peak in the final difference electron density synthesis was 0.258 $e^-/Å^3$ and the largest hole was -0.229 $e^-/Å^3$ with an RMS deviation of 0.043 $e^-/Å^3$. On the basis of the final model, the calculated density was 1.142 g/cm^3 and F(000), 3136 e^-.

2. References

[1] D. Seebach, R. Imwinkelried, G. Stucky, "Optisch aktive Alkohole aus 1,3-Dioxan-4-onen: eine praktikable Variante der enantioselektiven Synthese unter nucleophiler Substitution an Acetal-Zentren" *Helv. Chim. Acta* **1987**, *70*, 448–464.

[2] W. C. Still, M. Kahn, A. Mitra, "Rapid chromatographic technique for preparative separations with moderate resolution" *J. Org. Chem.* **1978**, *43*, 2923–2925.

[3] G. R. Fulmer, A. J. M. Miller, N. H. Sherden, H. E. Gottlieb, A. Nudelman, B. M. Stoltz, J. E. Bercaw, K. I. Goldberg, "NMR Chemical Shifts of Trace Impurities: Common Laboratory Solvents, Organics, and Gases in Deuterated Solvents Relevant to the Organometallic Chemist" *Organometallics* **2010**, *29*, 2176–2179.

[4] H. E. Gottlieb, V. Kotlyar, A. Nudelman, "NMR Chemical Shifts of Common Laboratory Solvents as Trace Impurities" *J. Org. Chem.* **1997**, *62*, 7512–7515.

[5] S. Grimme, "Exploration of Chemical Compound, Conformer, and Reaction Space with Meta-Dynamics Simulations Based on Tight-Binding Quantum Chemical Calculations" *J. Chem. Theory Comput.* **2019**, *15*, 2847–2862.

[6] S. Spicher, S. Grimme, "Robust Atomistic Modeling of Materials, Organometallic, and Biochemical Systems" *Angew. Chem. Int. Ed.* **2020**, *59*, 15665–15673.

[7] J.-D. Chai, M. Head-Gordon, "Long-range corrected hybrid density functionals with damped atom–atom dispersion corrections" *Phys. Chem. Chem. Phys.* **2008**, *10*, 6615.

[8] M. J. Frisch, J. A. Pople, J. S. Binkley, "Self-consistent molecular orbital methods 25. Supplementary functions for Gaussian basis sets" *J. Chem. Phys.* **1984**, *80*, 3265–3269.

[9] E. Cancès, B. Mennucci, J. Tomasi, "A new integral equation formalism for the polarizable continuum model: Theoretical background and applications to isotropic and anisotropic dielectrics" *J. Chem. Phys.* **1997**, *107*, 3032–3041.

[10] J. Tao, J. P. Perdew, V. N. Staroverov, G. E. Scuseria, "Climbing the Density Functional Ladder: Nonempirical Meta–Generalized Gradient Approximation Designed for Molecules and Solids" *Phys. Rev. Lett.* **2003**, *91*, 146401.

[11] S. Grimme, S. Ehrlich, L. Goerigk, "Effect of the damping function in dispersion corrected density functional theory" *J. Comput. Chem.* **2011**, *32*, 1456–1465.

[12] F. Weigend, R. Ahlrichs, "Balanced basis sets of split valence, triple zeta valence and quadruple zeta valence quality for H to Rn: Design and assessment of accuracy" *Phys. Chem. Chem. Phys.* **2005**, *7*, 3297.

[13] Gaussian 16 Revision C.01, M. J. Frisch, G. W. Trucks, H. B. Schlegel, G. E. Scuseria, M. A. Robb, J. R. Cheeseman, G. Scalmani, V. Barone, G. A. Petersson, H. Nakatsuji, X. Li, M. Caricato, A. V. Marenich, J. Bloino, B. G. Janesko, R. Gomperts, B. Mennucci, H. P. Hratchian, J. V. Ortiz, A. F. Izmaylov, J. L. Sonnenberg, D. Williams-Young, F. Ding, F. Lipparini, F. Egidi, J. Goings, B. Peng, A. Petrone, T. Henderson, D. Ranasinghe, V. G. Zakrzewski, J. Gao, N. Rega, G. Zheng, W. Liang, M. Hada, M. Ehara, K. Toyota, R. Fukuda, J. Hasegawa, M. Ishida, T. Nakajima, Y. Honda, O. Kitao, H. Nakai, T. Vreven, K. Throssell, J. A. Montgomery, Jr., J. E. Peralta, F. Ogliaro, M. J. Bearpark, J. J. Heyd, E. N. Brothers, K. N. Kudin, V. N. Staroverov, T. A. Keith, R. Kobayashi, J. Normand, K. Raghavachari, A. P. Rendell, J. C. Burant, S. S. Iyengar, J. Tomasi, M. Cossi, J. M. Millam, M. Klene, C. Adamo, R. Cammi, J. W. Ochterski, R. L. Martin, K. Morokuma, O. Farkas, J. B. Foresman, and D. J. Fox, Gaussian, Inc., Wallingford CT, 2016. **n.d.**

[14] T. Tran Ngoc, N. Grabicki, E. Irran, O. Dumele, J. F. Teichert, "Photoswitching neutral homoaromatic hydrocarbons" *Nat. Chem.* **2023**, *15*, 377–385.

[15] T. Tran Ngoc, J. van der Welle, T. Rüffer, J. F. Teichert, "Synthesis of Stable Neutral Homoaromatic Hydrocarbons" *Synthesis* **2023**, *55*, 2658–2669.

[16] A. Menzek, A. Altundas, D. Gültekin, "A new, safe and convenient procedure for reduction of naphthalene and anthracene: synthesis of tetralin in a one-pot reaction" *J. Chem. Res.* **2003**, *2003*, 752–753.

[17] M. Tobisu, K. Yamakawa, T. Shimasaki, N. Chatani, "Nickel-catalyzed reductive cleavage of aryl–oxygen bonds in alkoxy- and pivaloxyarenes using hydrosilanes as a mild reducing agent" *Chem. Commun.* **2011**, *47*, 2946.

[18] M. D. Cagle, S. J. Firsan, E. J. Eisenbraun, "PURIFICATION OF 1,2-DIHYDRONAPHTHALENE- AN IMPORTANT INTERMEDIATE IN THE HYDROPROCESSING OF NAPHTHALENE" *Org. Prep. Proced. Int.* **1994**, *26*, 370–373.

[19] M. Yus, R. P. Herrera, A. Guijarro, "On the Mechanism of Arene-Catalyzed Lithiation: The Role of Arene Dianions—Naphthalene Radical Anion versus Naphthalene Dianion" *Chem. Eur. J.* **2002**, *8*, 2574.

[20] A. Temperini, L. Minuti, T. Morini, O. Rosati, F. Piazzolla, "Isopropenyl acetate: A cheap and general acylating agent of alcohols under metal-free conditions" *Tetrahedron Lett.* **2017**, *58*, 4051–4053.

[21] Sutrisno, H. W. Wijaya, R. Retnosari, D. E. E. Putri Thrissur, India, **2021**, p. 050047.

[22] At this stage, the directionality of the chiroptical switching was unknown, however, the conclusion that in the case of any stereoinduction a set of two enantiomers of the iso-homoannulenes **7** would form remains the same. Therefore, in principle, also the opposite set of enantiomers (L,S)-**7** / (D,R)-**7** could form.

[23] T. Tran Ngoc, N. Grabicki, E. Irran, O. Dumele, J. F. Teichert, "Photoswitching neutral homoaromatic hydrocarbons" *Nat. Chem.* **2023**, *15*, 377–385.

[24], "Prolonged irradiation of **6** at 285 nm for leads to decomposition of the homoannulene switch. See the Supporting Information for details." **n.d.**

[25] N. Berova, K. Nakanishi, R. W. Woody, Eds. , *Circular dichroism: principles and applications*, Wiley-VCH, New York Weinheim, **2000**.

[26] G. Barth, E. Bunnenberg, C. Djerassi, D. Elder, R. Records, "Magnetic circular dichroism studies. Part 10.—Investigations of some carbonyl compounds" *Symp Faraday Soc* **1969**, *3*, 49–60.

[27] N. Harada, K. Nakanishi, "Exciton chirality method and its application to configurational and conformational studies of natural products" *Acc. Chem. Res.* **1972**, *5*, 257–263.

[28] S. F. Mason, "Optical rotatory power" *Q. Rev. Chem. Soc.* **1963**, *17*, 20.

[29] D. Tolochenko, B. Albano, T. Tran Ngoc, T. Rüffer, S. A. Lopez, J. F. Teichert, **2026**, DOI: 10.26434/chemrxiv.15000722/v2.

[30] H. Tanaka, Y. Inoue, T. Mori, "Circularly Polarized Luminescence and Circular Dichroisms in Small Organic Molecules: Correlation between Excitation and Emission Dissymmetry Factors" *ChemPhotoChem* **2018**, *2*, 386–402.

[31] Gaussian 09, Rev E.01, M. J. Frisch, G. W. Trucks, H. B. Schlegel, G. E. Scuseria, M. A. Robb, J. R. Cheeseman, G. Scalmani, V. Barone, B. Mennucci, G. A. Petersson, H. Nakatsuji, M. Caricato, X. Li, H. P. Hratchian, A. F. Izmaylov, J. Bloino, G. Zheng, J. L. Sonnenberg, M. Hada, M. Ehara, K. Toyota, R. Fukuda, J. Hasegawa, M. Ishida, T. Nakajima, Y. Honda, O. Kitao, H. Nakai, T. Vreven, J. J. A. Montgomery, J. E. Peralta, F. Ogliaro, M. Bearpark, J. J. Heyd, E. Brothers, K. N. Kudin, V. N. Staroverov, T. Keith, R. Kobayashi, J. Normand, K. Raghavachari, A. Rendell, J. C. Burant, S. S. Iyengar, J. Tomasi, M. Cossi, N. Rega, J. M. Millam, M. Klene, J. E. Knox, J. B. Cross, V. Bakken, C.

Adamo, J. Jaramillo, R. Gomperts, R. E. Stratmann, O. Yazyev, A. J. Austin, R. Cammi, C. Pomelli, J. W. Ochterski, R. L. Martin, K. Morokuma, V. G. Zakrzewski, G. A. Voth, P. Salvador, J. J. Dannenberg, S. Dapprich, A. D. Daniels, O. Farkas, J. B. Foresman, J. V. Ortiz, J. Cioslowski and D. J. Fox, **2013**, *Wallingford CT, USA*.

[32] Spartan 14, Wavefunction Inc., Irvine, CA, USA **2014**.

[33] T. A. Halgren, "Merck molecular force field. I. Basis, form, scope, parameterization, and performance of MMFF94" *J. Comput. Chem.* **1996**, *17*, 490–519.

[34] H. Günther, *NMR spectroscopy: basic principles, concepts and applications in chemistry*, Wiley-VCH Verlag GmbH & Co. KGaA, Weinheim, **2013**.

[35] M. T. Huggins, T. Kesharwani, J. Buttrick, C. Nicholson, "Variable Temperature NMR Experiment Studying Restricted Bond Rotation" *J. Chem. Educ.* **2020**, *97*, 1425–1429.

[36] H. Friebolin, *Basic one- and two-dimensional NMR spectroscopy*, Wiley-VCH, Weinheim, **2011**.

[37] H. Kessler, "Detection of Hindered Rotation and Inversion by NMR Spectroscopy" *Angew. Chem. Int. Ed. Engl.* **1970**, *9*, 219–235.

[38] Trung Tran Ngoc, Synthesis and Investigation of Neutral Homoaromatic Molecules and Their Photoswitching Behavior, Dissertation, Technische Universität Chemnitz, **2024**.

[39] G. M. Sheldrick, "Crystal structure refinement with SHELXL" *Acta Crystallogr. Sect. C Struct. Chem.* **2015**, *71*, 3–8.

[40] G. M. Sheldrick, "A short history of *SHELX*" *Acta Crystallogr. A* **2008**, *64*, 112–122.

Spectral and Chromatographic Appendix

Chiroptical Switching by Stereoselective Photochemical Sigmatropic Rearrangements – Experimental Realization of Calderoselectivity

Anne Krenkel,[a] Frederike Beyer,[b] Malte Feßner,[b] Trung Tran Ngoc,[a] Tobias Rüffer,[a] , Dominik Hornig,[a] Martin Breugst,[a] Christian Merten,[b] Johannes F. Teichert[a]*

(a) Institut für Chemie, Technische Universität Chemnitz, 09111 Chemnitz, Germany;
(b) Ruhr Universität Bochum, Fakultät für Chemie und Biochemie, Organische Chemie II, Universitätsstraße 150, 44801 Bochum, Germany.

johannes.teichert@chemie.tu-chemnitz.de

1. Crystallographic data

D-Menthyl ester triasteranone ((D)-12)

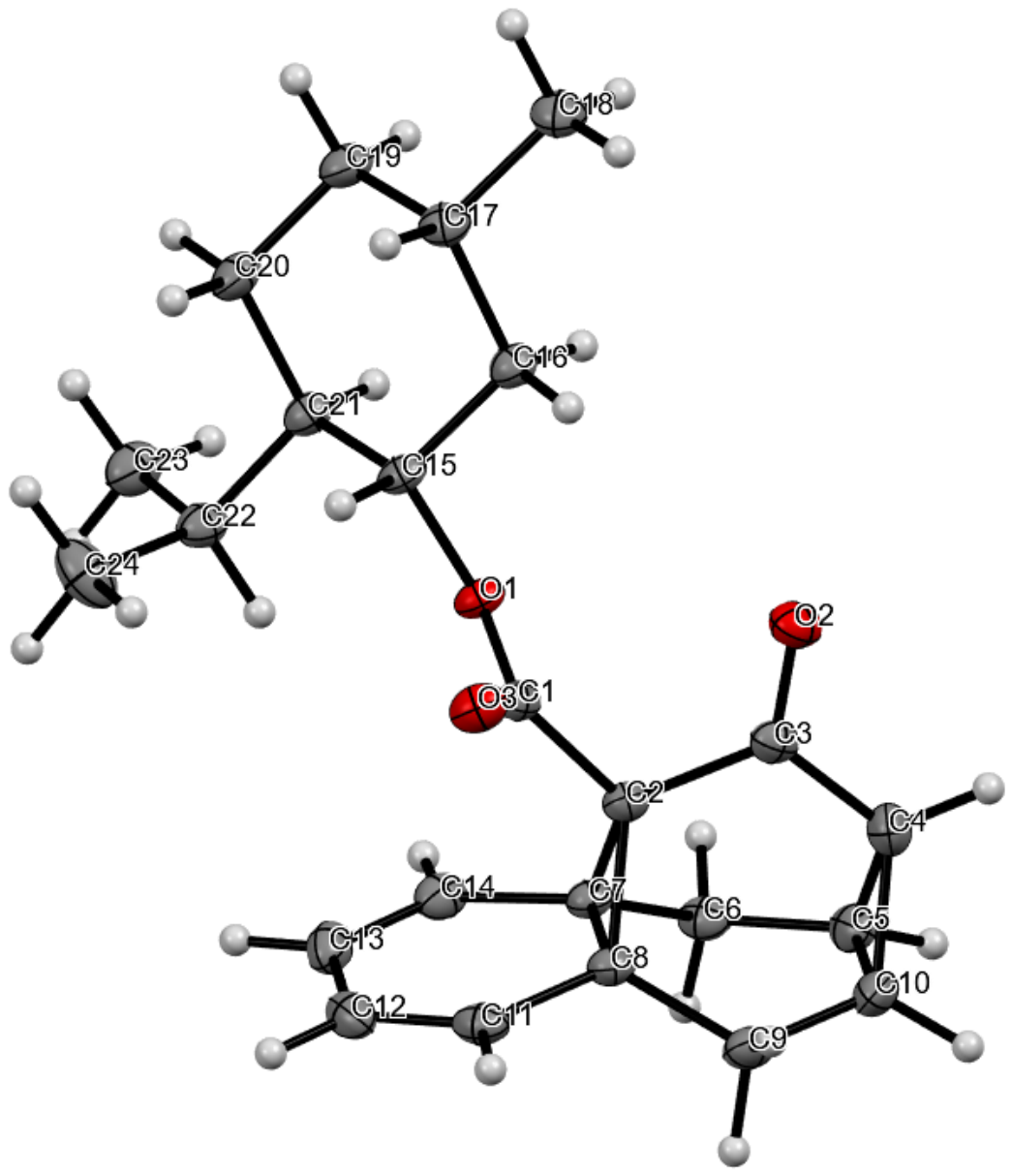

	CCDC 2531740
Empirical formula	$C_{24}H_{30}O_3$
Formula weight	366.48
Temperature	100 K
Wavelength	1.54178 A
Crystal system, space group	Monoclinic, P 21
Unit cell dimensions	a = 6.2917(2) A α = 90°
	b = 9.9335(3) A β = 99.607(1)°
	c = 16.0822(4) A γ = 90°
Volume	991.02(5) A^3
Z, Calculated density	2.1.228 Mg/m^3
Absorption coefficient	0.623 mm^{-1}
F(*000*)	396
Crystal size	0.230 x 0.100 x 0.060 mm
Theta range for data collection	5.254 to 74.396°
Limiting indices	-7<=h<=7, -12<=k<=12, -20<=l<=20
Reflections collected / unique	31186 / 4019 [R(int) = 0.0329]

Completeness to θ = 67.679	99.7 %
Absorption correction	Semi-empirical from equivalents
Max. and min. transmission	0.96 and 0.82
Refinement method	Full-matrix least-squares on F^2
Data / restraints / parameters	4019 / 1 / 247
Goodness-of-fit on F^2	1.029
Final R indices [$I>2\sigma$(I)]	R1 = 0.0288, wR2 = 0.0757
R indices (all data)	R1 = 0.0291, wR2 = 0.0760
Absolute structure parameter	-0.01(7)
Largest diff. peak and hole	0.225 and –0.153 e^{-} A^{-3}

Table S1. Bond lengths [A] and angles [deg]

C(1)-O(3)	1.204(2)
C(1)-O(1)	1.3388(19)
C(1)-C(2)	1.509(2)
C(2)-C(3)	1.493(2)
C(2)-C(8)	1.544(2)
C(2)-C(7)	1.551(2)
C(3)-O(2)	1.223(2)
C(3)-C(4)	1.466(2)
C(4)-C(5)	1.528(3)
C(4)-C(10)	1.534(2)
C(4)-H(4)	1.0000
C(5)-C(10)	1.488(3)
C(5)-C(6)	1.504(2)
C(5)-H(5)	1.0000
C(6)-C(7)	1.513(2)
C(6)-H(6A)	0.9900
C(6)-H(6B)	0.9900
C(7)-C(14)	1.477(2)
C(7)-C(8)	1.526(2)
C(8)-C(11)	1.480(2)
C(8)-C(9)	1.518(2)
C(9)-C(10)	1.510(3)
C(9)-H(9A)	0.9900

C(9)-H(9B)	0.9900
C(10)-H(10)	1.0000
C(11)-C(12)	1.333(3)
C(11)-H(11)	0.9500
C(12)-C(13)	1.452(3)
C(12)-H(12)	0.9500
C(13)-C(14)	1.338(3)
C(13)-H(13)	0.9500
C(14)-H(14)	0.9500
C(15)-O(1)	1.4703(18)
C(15)-C(16)	1.525(2)
C(15)-C(21)	1.528(2)
C(15)-H(15)	1.0000
C(16)-C(17)	1.532(2)
C(16)-H(16A)	0.9900
C(16)-H(16B)	0.9900
C(17)-C(18)	1.527(2)
C(17)-C(19)	1.529(2)
C(17)-H(17)	1.0000
C(18)-H(18A)	0.9800
C(18)-H(18B)	0.9800
C(18)-H(18C)	0.9800
C(19)-C(20)	1.523(2)
C(19)-H(19A)	0.9900
C(19)-H(19B)	0.9900
C(20)-C(21)	1.536(2)
C(20)-H(20A)	0.9900
C(20)-H(20B)	0.9900
C(21)-C(22)	1.545(2)
C(21)-H(21)	1.0000
C(22)-C(24)	1.530(3)
C(22)-C(23)	1.532(2)
C(22)-H(22)	1.0000
C(23)-H(23A)	0.9800
C(23)-H(23B)	0.9800
C(23)-H(23C)	0.9800
C(24)-H(24A)	0.9800

C(24)-H(24B)	0.9800
C(24)-H(24C)	0.9800

O(3)-C(1)-O(1)	124.83(15)
O(3)-C(1)-C(2)	124.00(14)
O(1)-C(1)-C(2)	111.04(13)
C(3)-C(2)-C(1)	111.36(13)
C(3)-C(2)-C(8)	116.61(13)
C(1)-C(2)-C(8)	120.98(13)
C(3)-C(2)-C(7)	117.22(13)
C(1)-C(2)-C(7)	122.58(13)
C(8)-C(2)-C(7)	59.09(10)
O(2)-C(3)-C(4)	123.02(15)
O(2)-C(3)-C(2)	120.15(15)
C(4)-C(3)-C(2)	116.83(14)
C(3)-C(4)-C(5)	118.25(14)
C(3)-C(4)-C(10)	116.63(15)
C(5)-C(4)-C(10)	58.13(12)
C(3)-C(4)-H(4)	116.9
C(5)-C(4)-H(4)	116.9
C(10)-C(4)-H(4)	116.9
C(10)-C(5)-C(6)	120.27(15)
C(10)-C(5)-C(4)	61.17(12)
C(6)-C(5)-C(4)	117.80(15)
C(10)-C(5)-H(5)	115.6
C(6)-C(5)-H(5)	115.6
C(4)-C(5)-H(5)	115.6
C(5)-C(6)-C(7)	113.00(15)
C(5)-C(6)-H(6A)	109.0
C(7)-C(6)-H(6A)	109.0
C(5)-C(6)-H(6B)	109.0
C(7)-C(6)-H(6B)	109.0
H(6A)-C(6)-H(6B)	107.8
C(14)-C(7)-C(6)	115.17(15)
C(14)-C(7)-C(8)	116.58(14)
C(6)-C(7)-C(8)	118.92(14)
C(14)-C(7)-C(2)	117.86(14)

C(6)-C(7)-C(2)	117.17(13)
C(8)-C(7)-C(2)	60.21(10)
C(11)-C(8)-C(9)	115.27(14)
C(11)-C(8)-C(7)	116.07(14)
C(9)-C(8)-C(7)	119.15(14)
C(11)-C(8)-C(2)	118.63(13)
C(9)-C(8)-C(2)	116.24(14)
C(7)-C(8)-C(2)	60.70(10)
C(10)-C(9)-C(8)	113.15(14)
C(10)-C(9)-H(9A)	108.9
C(8)-C(9)-H(9A)	108.9
C(10)-C(9)-H(9B)	108.9
C(8)-C(9)-H(9B)	108.9
H(9A)-C(9)-H(9B)	107.8
C(5)-C(10)-C(9)	119.85(15)
C(5)-C(10)-C(4)	60.71(12)
C(9)-C(10)-C(4)	117.29(14)
C(5)-C(10)-H(10)	115.9
C(9)-C(10)-H(10)	115.9
C(4)-C(10)-H(10)	115.9
C(12)-C(11)-C(8)	122.54(16)
C(12)-C(11)-H(11)	118.7
C(8)-C(11)-H(11)	118.7
C(11)-C(12)-C(13)	121.36(16)
C(11)-C(12)-H(12)	119.3
C(13)-C(12)-H(12)	119.3
C(14)-C(13)-C(12)	121.10(16)
C(14)-C(13)-H(13)	119.5
C(12)-C(13)-H(13)	119.5
C(13)-C(14)-C(7)	122.30(16)
C(13)-C(14)-H(14)	118.9
C(7)-C(14)-H(14)	118.9
O(1)-C(15)-C(16)	107.68(12)
O(1)-C(15)-C(21)	107.04(12)
C(16)-C(15)-C(21)	112.24(13)
O(1)-C(15)-H(15)	109.9
C(16)-C(15)-H(15)	109.9

C(21)-C(15)-H(15)	109.9
C(15)-C(16)-C(17)	112.18(13)
C(15)-C(16)-H(16A)	109.2
C(17)-C(16)-H(16A)	109.2
C(15)-C(16)-H(16B)	109.2
C(17)-C(16)-H(16B)	109.2
H(16A)-C(16)-H(16B)	107.9
C(18)-C(17)-C(19)	110.56(14)
C(18)-C(17)-C(16)	111.17(14)
C(19)-C(17)-C(16)	110.30(13)
C(18)-C(17)-H(17)	108.2
C(19)-C(17)-H(17)	108.2
C(16)-C(17)-H(17)	108.2
C(17)-C(18)-H(18A)	109.5
C(17)-C(18)-H(18B)	109.5
H(18A)-C(18)-H(18B)	109.5
C(17)-C(18)-H(18C)	109.5
H(18A)-C(18)-H(18C)	109.5
H(18B)-C(18)-H(18C)	109.5
C(20)-C(19)-C(17)	111.75(14)
C(20)-C(19)-H(19A)	109.3
C(17)-C(19)-H(19A)	109.3
C(20)-C(19)-H(19B)	109.3
C(17)-C(19)-H(19B)	109.3
H(19A)-C(19)-H(19B)	107.9
C(19)-C(20)-C(21)	111.66(14)
C(19)-C(20)-H(20A)	109.3
C(21)-C(20)-H(20A)	109.3
C(19)-C(20)-H(20B)	109.3
C(21)-C(20)-H(20B)	109.3
H(20A)-C(20)-H(20B)	108.0
C(15)-C(21)-C(20)	107.95(13)
C(15)-C(21)-C(22)	113.33(13)
C(20)-C(21)-C(22)	114.11(14)
C(15)-C(21)-H(21)	107.0
C(20)-C(21)-H(21)	107.0
C(22)-C(21)-H(21)	107.0

C(24)-C(22)-C(23)	110.40(16)
C(24)-C(22)-C(21)	113.88(14)
C(23)-C(22)-C(21)	110.76(14)
C(24)-C(22)-H(22)	107.2
C(23)-C(22)-H(22)	107.2
C(21)-C(22)-H(22)	107.2
C(22)-C(23)-H(23A)	109.5
C(22)-C(23)-H(23B)	109.5
H(23A)-C(23)-H(23B)	109.5
C(22)-C(23)-H(23C)	109.5
H(23A)-C(23)-H(23C)	109.5
H(23B)-C(23)-H(23C)	109.5

C(22)-C(24)-H(24A)	109.5
C(22)-C(24)-H(24B)	109.5
H(24A)-C(24)-H(24B)	109.5
C(22)-C(24)-H(24C)	109.5
H(24A)-C(24)-H(24C)	109.5
H(24B)-C(24)-H(24C)	109.5
C(1)-O(1)-C(15)	116.72(12)

(1*R*,2*S*,5*R*)-2-Isopropyl-5-methylcyclohexyl 1-oxo-1,2-dihydro-9a*H*-2,9-ethenobenzo[7]annulen-9a-carboxylate ((L)-6)

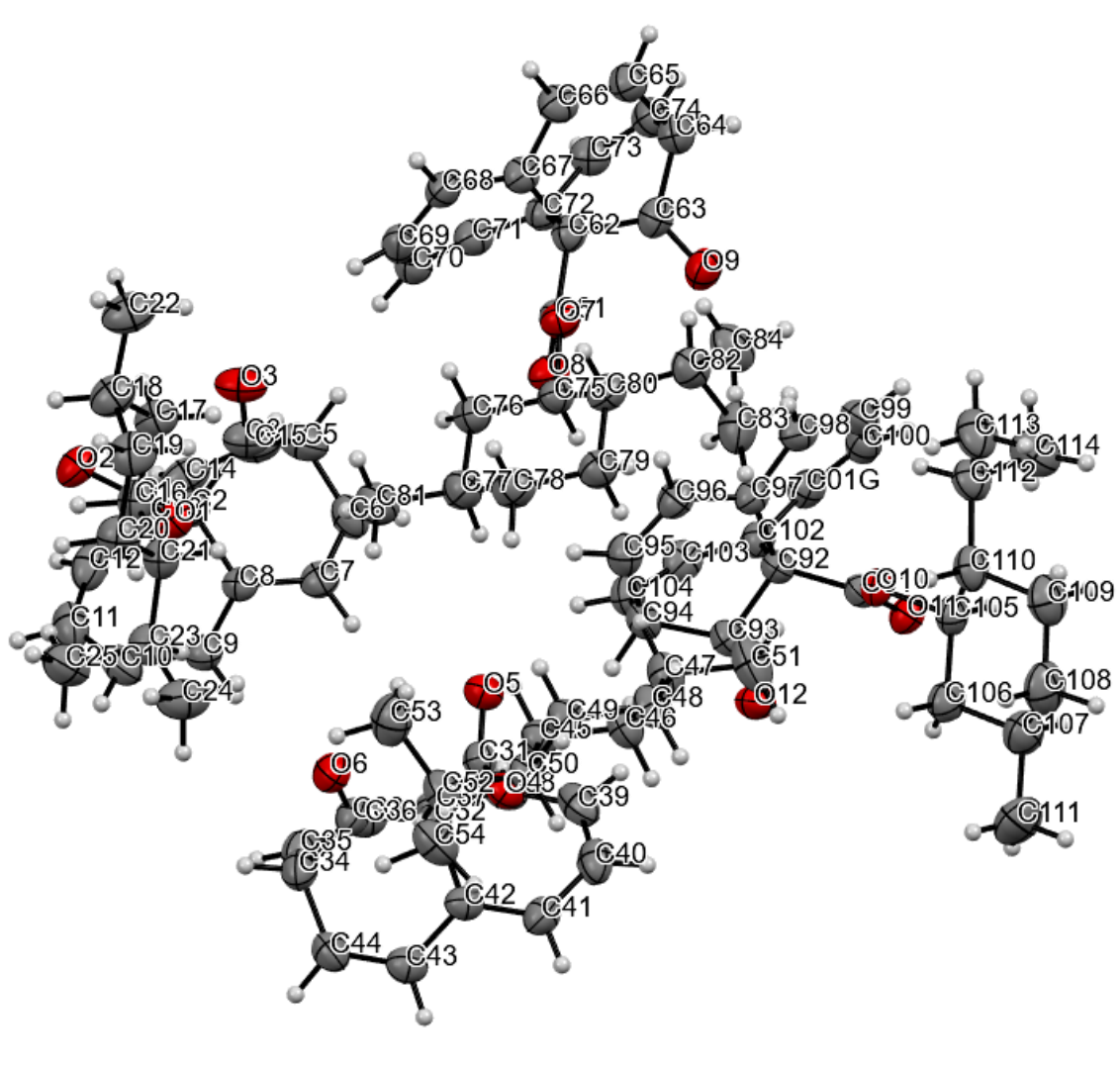

CCDC 2531744

Empirical formula	$C_{24}H_{28}O_3$ [+1/4 C_6H_{12}]
Formula weight	364.46
Temperature	100 K
Wavelength	1.54178 A
Crystal system, space group	Orthorhombic, P 21 21 21
Unit cell dimensions	a = 14.3194(5) A α = 90°
	b = 15.6982(5) A β = 90°
	c = 37.7074(11) A γ = 90°
Volume	8476.2(5) A^3
Z, Calculated density	16, 1.142 Mg/m^3
Absorption coefficient	0.583 mm^{-1}
F(*000*)	3136
Crystal size	0.220 x 0.090 x 0.040 mm
Theta range for data collection	3.049 to 66.996°
Limiting indices	-17<=h<=16, -18<=k<=17, -44<=l<=45
Reflections collected / unique	85785 / 15093 [R(int) = 0.1214]
Completeness to θ = 67.679	98.5 %
Absorption correction	Semi-empirical from equivalents
Max. and min. transmission	0.97 and 0.77
Refinement method	Full-matrix least-squares on F^2
Data / restraints / parameters	15093 / 0 / 985
Goodness-of-fit on F^2	1.074
Final R indices [I>2σ(I)]	R1 = 0.0586, wR2 = 0.1435
R indices (all data)	R1 = 0.0852, wR2 = 0.1582
Absolute structure parameter	-0.10(15)
Extinction coefficient	n/a
Largest diff. peak and hole	0.258 and -0.229 e^-A^{-3}

Table S2. Bond lengths [A] and angles [deg] for platon_sq.

C(1)-O(2)	1.205(6)
C(1)-O(1)	1.335(5)
C(1)-C(2)	1.528(7)
C(01G)-C(102)	1.363(7)

C(01G)-C(100)	1.395(7)
C(01G)-H(01G)	0.9500
C(2)-C(13)	1.499(7)
C(2)-C(8)	1.531(6)
C(2)-C(3)	1.532(7)
C(3)-O(3)	1.216(6)
C(3)-C(5)	1.487(8)
C(5)-C(15)	1.522(8)
C(5)-C(6)	1.524(8)
C(5)-H(5)	1.0000
C(6)-C(7)	1.324(7)
C(6)-H(6)	0.9500
C(7)-C(8)	1.452(7)
C(7)-H(7)	0.9500
C(8)-C(9)	1.359(7)
C(9)-C(10)	1.419(7)
C(9)-H(9)	0.9500
C(10)-C(11)	1.366(8)
C(10)-H(10)	0.9500
C(11)-C(12)	1.401(8)
C(11)-H(11)	0.9500
C(12)-C(13)	1.382(8)
C(12)-H(12)	0.9500
C(13)-C(14)	1.444(8)
C(14)-C(15)	1.330(9)
C(14)-H(14)	0.9500
C(15)-H(15)	0.9500
C(16)-O(1)	1.458(5)
C(16)-C(21)	1.511(7)
C(16)-C(17)	1.520(7)
C(16)-H(16)	1.0000
C(17)-C(18)	1.530(7)
C(17)-H(17A)	0.9900
C(17)-H(17B)	0.9900
C(18)-C(22)	1.522(8)
C(18)-C(19)	1.532(8)
C(18)-H(18)	1.0000

C(19)-C(20)	1.518(8)
C(19)-H(19A)	0.9900
C(19)-H(19B)	0.9900
C(20)-C(21)	1.541(7)
C(20)-H(20A)	0.9900
C(20)-H(20B)	0.9900
C(21)-C(23)	1.547(7)
C(21)-H(21)	1.0000
C(22)-H(22A)	0.9800
C(22)-H(22B)	0.9800
C(22)-H(22C)	0.9800
C(23)-C(25)	1.520(8)
C(23)-C(24)	1.530(7)
C(23)-H(23)	1.0000
C(24)-H(24A)	0.9800
C(24)-H(24B)	0.9800
C(24)-H(24C)	0.9800
C(25)-H(25A)	0.9800
C(25)-H(25B)	0.9800
C(25)-H(25C)	0.9800
C(31)-O(5)	1.208(6)
C(31)-O(4)	1.330(5)
C(31)-C(32)	1.527(6)
C(32)-C(33)	1.514(7)
C(32)-C(37)	1.521(6)
C(32)-C(42)	1.523(6)
C(33)-O(6)	1.224(6)
C(33)-C(34)	1.477(7)
C(34)-C(35)	1.503(8)
C(34)-C(44)	1.519(8)
C(34)-H(34)	1.0000
C(35)-C(36)	1.340(8)
C(35)-H(35)	0.9500
C(36)-C(37)	1.438(7)
C(36)-H(36)	0.9500
C(37)-C(38)	1.355(7)
C(38)-C(39)	1.407(8)

C(38)-H(38)	0.9500
C(39)-C(40)	1.377(8)
C(39)-H(39)	0.9500
C(40)-C(41)	1.407(8)
C(40)-H(40)	0.9500
C(41)-C(42)	1.362(7)
C(41)-H(41)	0.9500
C(42)-C(43)	1.449(7)
C(43)-C(44)	1.338(7)
C(43)-H(43)	0.9500
C(44)-H(44)	0.9500
C(45)-O(4)	1.465(5)
C(45)-C(46)	1.515(7)
C(45)-C(50)	1.537(7)
C(45)-H(45)	1.0000
C(46)-C(47)	1.533(7)
C(46)-H(46A)	0.9900
C(46)-H(46B)	0.9900
C(47)-C(51)	1.526(8)
C(47)-C(48)	1.527(7)
C(47)-H(47)	1.0000
C(48)-C(49)	1.519(7)
C(48)-H(48A)	0.9900
C(48)-H(48B)	0.9900
C(49)-C(50)	1.527(7)
C(49)-H(49A)	0.9900
C(49)-H(49B)	0.9900
C(50)-C(52)	1.537(7)
C(50)-H(50)	1.0000
C(51)-H(51A)	0.9800
C(51)-H(51B)	0.9800
C(51)-H(51C)	0.9800
C(52)-C(53)	1.516(8)
C(52)-C(54)	1.523(7)
C(52)-H(52)	1.0000
C(53)-H(53A)	0.9800
C(53)-H(53B)	0.9800

C(53)-H(53C)	0.9800
C(54)-H(54A)	0.9800
C(54)-H(54B)	0.9800
C(54)-H(54C)	0.9800
C(61)-O(8)	1.214(6)
C(61)-O(7)	1.337(6)
C(61)-C(62)	1.525(7)
C(62)-C(67)	1.519(7)
C(62)-C(72)	1.520(6)
C(62)-C(63)	1.521(7)
C(63)-O(9)	1.219(6)
C(63)-C(64)	1.497(7)
C(64)-C(74)	1.508(7)
C(64)-C(65)	1.527(7)
C(64)-H(64)	1.0000
C(65)-C(66)	1.331(7)
C(65)-H(65)	0.9500
C(66)-C(67)	1.433(7)
C(66)-H(66)	0.9500
C(67)-C(68)	1.384(7)
C(68)-C(69)	1.412(7)
C(68)-H(68)	0.9500
C(69)-C(70)	1.381(7)
C(69)-H(69)	0.9500
C(70)-C(71)	1.396(7)
C(70)-H(70)	0.9500
C(71)-C(72)	1.351(7)
C(71)-H(71)	0.9500
C(72)-C(73)	1.456(7)
C(73)-C(74)	1.351(7)
C(73)-H(73)	0.9500
C(74)-H(74)	0.9500
C(75)-O(7)	1.463(6)
C(75)-C(76)	1.498(7)
C(75)-C(80)	1.534(7)
C(75)-H(75)	1.0000
C(76)-C(77)	1.530(7)

C(76)-H(76A)	0.9900
C(76)-H(76B)	0.9900
C(77)-C(78)	1.512(8)
C(77)-C(81)	1.529(8)
C(77)-H(77)	1.0000
C(78)-C(79)	1.527(8)
C(78)-H(78A)	0.9900
C(78)-H(78B)	0.9900
C(79)-C(80)	1.530(7)
C(79)-H(79A)	0.9900
C(79)-H(79B)	0.9900
C(80)-C(82)	1.533(7)
C(80)-H(80)	1.0000
C(81)-H(81A)	0.9800
C(81)-H(81B)	0.9800
C(81)-H(81C)	0.9800
C(82)-C(84)	1.521(7)
C(82)-C(83)	1.523(8)
C(82)-H(82)	1.0000
C(83)-H(83A)	0.9800
C(83)-H(83B)	0.9800
C(83)-H(83C)	0.9800
C(84)-H(84A)	0.9800
C(84)-H(84B)	0.9800
C(84)-H(84C)	0.9800
C(91)-O(11)	1.221(5)
C(91)-O(10)	1.334(5)
C(91)-C(92)	1.516(6)
C(92)-C(97)	1.518(6)
C(92)-C(102)	1.521(6)
C(92)-C(93)	1.528(6)
C(93)-O(12)	1.220(6)
C(93)-C(94)	1.483(7)
C(94)-C(95)	1.505(7)
C(94)-C(104)	1.514(7)
C(94)-H(94)	1.0000
C(95)-C(96)	1.332(7)

C(95)-H(95)	0.9500
C(96)-C(97)	1.445(7)
C(96)-H(96)	0.9500
C(97)-C(98)	1.366(7)
C(98)-C(99)	1.420(8)
C(98)-H(98)	0.9500
C(99)-C(100)	1.379(8)
C(99)-H(99)	0.9500
C(100)-H(100)	0.9500
C(102)-C(103)	1.445(7)
C(103)-C(104)	1.337(7)
C(103)-H(103)	0.9500
C(104)-H(104)	0.9500
C(105)-O(10)	1.462(5)
C(105)-C(106)	1.515(7)
C(105)-C(110)	1.529(7)
C(105)-H(105)	1.0000
C(106)-C(107)	1.535(7)
C(106)-H(10E)	0.9900
C(106)-H(10F)	0.9900
C(107)-C(108)	1.509(9)
C(107)-C(111)	1.538(9)
C(107)-H(107)	1.0000
C(108)-C(109)	1.535(9)
C(108)-H(10G)	0.9900
C(108)-H(10H)	0.9900
C(109)-C(110)	1.538(7)
C(109)-H(10I)	0.9900
C(109)-H(10J)	0.9900
C(110)-C(112)	1.544(8)
C(110)-H(110)	1.0000
C(111)-H(11A)	0.9800
C(111)-H(11B)	0.9800
C(111)-H(11C)	0.9800
C(112)-C(113)	1.520(7)
C(112)-C(114)	1.528(8)
C(112)-H(112)	1.0000

C(113)-H(11D)	0.9800
C(113)-H(11E)	0.9800
C(113)-H(11F)	0.9800
C(114)-H(11G)	0.9800
C(114)-H(11H)	0.9800
C(114)-H(11I)	0.9800
O(2)-C(1)-O(1)	125.5(4)
O(2)-C(1)-C(2)	123.6(4)
O(1)-C(1)-C(2)	110.7(4)
C(102)-C(01G)-C(100)	125.7(5)
C(102)-C(01G)-H(01G)	117.1
C(100)-C(01G)-H(01G)	117.1
C(13)-C(2)-C(1)	115.6(4)
C(13)-C(2)-C(8)	101.0(4)
C(1)-C(2)-C(8)	115.5(4)
C(13)-C(2)-C(3)	109.4(4)
C(1)-C(2)-C(3)	105.5(4)
C(8)-C(2)-C(3)	109.9(4)
O(3)-C(3)-C(5)	124.3(5)
O(3)-C(3)-C(2)	123.2(5)
C(5)-C(3)-C(2)	112.4(4)
C(3)-C(5)-C(15)	106.3(5)
C(3)-C(5)-C(6)	108.6(4)
C(15)-C(5)-C(6)	109.8(4)
C(3)-C(5)-H(5)	110.7
C(15)-C(5)-H(5)	110.7
C(6)-C(5)-H(5)	110.7
C(7)-C(6)-C(5)	120.9(5)
C(7)-C(6)-H(6)	119.5
C(5)-C(6)-H(6)	119.6
C(6)-C(7)-C(8)	123.7(5)
C(6)-C(7)-H(7)	118.1
C(8)-C(7)-H(7)	118.1
C(9)-C(8)-C(7)	123.6(4)
C(9)-C(8)-C(2)	118.5(4)
C(7)-C(8)-C(2)	117.3(4)
C(8)-C(9)-C(10)	126.1(5)

C(8)-C(9)-H(9)	116.9
C(10)-C(9)-H(9)	117.0
C(11)-C(10)-C(9)	124.8(5)
C(11)-C(10)-H(10)	117.6
C(9)-C(10)-H(10)	117.6
C(10)-C(11)-C(12)	125.7(5)
C(10)-C(11)-H(11)	117.1
C(12)-C(11)-H(11)	117.1
C(13)-C(12)-C(11)	125.7(5)
C(13)-C(12)-H(12)	117.1
C(11)-C(12)-H(12)	117.1
C(12)-C(13)-C(14)	122.5(5)
C(12)-C(13)-C(2)	119.0(5)
C(14)-C(13)-C(2)	118.0(5)
C(15)-C(14)-C(13)	123.0(5)
C(15)-C(14)-H(14)	118.5
C(13)-C(14)-H(14)	118.5
C(14)-C(15)-C(5)	121.2(5)
C(14)-C(15)-H(15)	119.4
C(5)-C(15)-H(15)	119.4
O(1)-C(16)-C(21)	108.6(4)
O(1)-C(16)-C(17)	109.5(4)
C(21)-C(16)-C(17)	110.7(4)
O(1)-C(16)-H(16)	109.3
C(21)-C(16)-H(16)	109.3
C(17)-C(16)-H(16)	109.3
C(16)-C(17)-C(18)	110.5(4)
C(16)-C(17)-H(17A)	109.5
C(18)-C(17)-H(17A)	109.5
C(16)-C(17)-H(17B)	109.5
C(18)-C(17)-H(17B)	109.5
H(17A)-C(17)-H(17B)	108.1
C(22)-C(18)-C(17)	112.6(5)
C(22)-C(18)-C(19)	111.5(5)
C(17)-C(18)-C(19)	108.8(4)
C(22)-C(18)-H(18)	107.9
C(17)-C(18)-H(18)	107.9

C(19)-C(18)-H(18)	107.9
C(20)-C(19)-C(18)	112.1(5)
C(20)-C(19)-H(19A)	109.2
C(18)-C(19)-H(19A)	109.2
C(20)-C(19)-H(19B)	109.2
C(18)-C(19)-H(19B)	109.2
H(19A)-C(19)-H(19B)	107.9
C(19)-C(20)-C(21)	113.0(5)
C(19)-C(20)-H(20A)	109.0
C(21)-C(20)-H(20A)	109.0
C(19)-C(20)-H(20B)	109.0
C(21)-C(20)-H(20B)	109.0
H(20A)-C(20)-H(20B)	107.8
C(16)-C(21)-C(20)	107.3(4)
C(16)-C(21)-C(23)	113.3(4)
C(20)-C(21)-C(23)	113.6(4)
C(16)-C(21)-H(21)	107.4
C(20)-C(21)-H(21)	107.4
C(23)-C(21)-H(21)	107.4
C(18)-C(22)-H(22A)	109.5
C(18)-C(22)-H(22B)	109.5
H(22A)-C(22)-H(22B)	109.5
C(18)-C(22)-H(22C)	109.5
H(22A)-C(22)-H(22C)	109.5
H(22B)-C(22)-H(22C)	109.5
C(25)-C(23)-C(24)	111.8(5)
C(25)-C(23)-C(21)	113.7(5)
C(24)-C(23)-C(21)	110.5(4)
C(25)-C(23)-H(23)	106.8
C(24)-C(23)-H(23)	106.8
C(21)-C(23)-H(23)	106.8
C(23)-C(24)-H(24A)	109.5
C(23)-C(24)-H(24B)	109.5
H(24A)-C(24)-H(24B)	109.5
C(23)-C(24)-H(24C)	109.5
H(24A)-C(24)-H(24C)	109.5
H(24B)-C(24)-H(24C)	109.5

C(23)-C(25)-H(25A)	109.5
C(23)-C(25)-H(25B)	109.5
H(25A)-C(25)-H(25B)	109.5
C(23)-C(25)-H(25C)	109.5
H(25A)-C(25)-H(25C)	109.5
H(25B)-C(25)-H(25C)	109.5
O(5)-C(31)-O(4)	125.0(4)
O(5)-C(31)-C(32)	123.1(4)
O(4)-C(31)-C(32)	111.4(4)
C(33)-C(32)-C(37)	109.9(4)
C(33)-C(32)-C(42)	109.8(4)
C(37)-C(32)-C(42)	99.9(4)
C(33)-C(32)-C(31)	105.3(4)
C(37)-C(32)-C(31)	114.8(4)
C(42)-C(32)-C(31)	117.1(4)
O(6)-C(33)-C(34)	123.6(5)
O(6)-C(33)-C(32)	123.1(4)
C(34)-C(33)-C(32)	113.3(4)
C(33)-C(34)-C(35)	108.1(5)
C(33)-C(34)-C(44)	107.9(4)
C(35)-C(34)-C(44)	107.6(4)
C(33)-C(34)-H(34)	111.1
C(35)-C(34)-H(34)	111.1
C(44)-C(34)-H(34)	111.1
C(36)-C(35)-C(34)	121.4(5)
C(36)-C(35)-H(35)	119.3
C(34)-C(35)-H(35)	119.3
C(35)-C(36)-C(37)	123.2(5)
C(35)-C(36)-H(36)	118.4
C(37)-C(36)-H(36)	118.4
C(38)-C(37)-C(36)	123.1(5)
C(38)-C(37)-C(32)	119.1(4)
C(36)-C(37)-C(32)	117.3(4)
C(37)-C(38)-C(39)	125.7(5)
C(37)-C(38)-H(38)	117.2
C(39)-C(38)-H(38)	117.2
C(40)-C(39)-C(38)	124.6(5)

C(40)-C(39)-H(39)	117.7
C(38)-C(39)-H(39)	117.7
C(39)-C(40)-C(41)	125.9(5)
C(39)-C(40)-H(40)	117.0
C(41)-C(40)-H(40)	117.0
C(42)-C(41)-C(40)	125.5(5)
C(42)-C(41)-H(41)	117.2
C(40)-C(41)-H(41)	117.2
C(41)-C(42)-C(43)	123.9(4)
C(41)-C(42)-C(32)	117.9(4)
C(43)-C(42)-C(32)	117.8(4)
C(44)-C(43)-C(42)	122.8(5)
C(44)-C(43)-H(43)	118.6
C(42)-C(43)-H(43)	118.6
C(43)-C(44)-C(34)	121.1(5)
C(43)-C(44)-H(44)	119.5
C(34)-C(44)-H(44)	119.5
O(4)-C(45)-C(46)	109.3(4)
O(4)-C(45)-C(50)	107.9(4)
C(46)-C(45)-C(50)	111.7(4)
O(4)-C(45)-H(45)	109.3
C(46)-C(45)-H(45)	109.3
C(50)-C(45)-H(45)	109.3
C(45)-C(46)-C(47)	111.9(4)
C(45)-C(46)-H(46A)	109.2
C(47)-C(46)-H(46A)	109.2
C(45)-C(46)-H(46B)	109.2
C(47)-C(46)-H(46B)	109.2
H(46A)-C(46)-H(46B)	107.9
C(51)-C(47)-C(48)	112.4(5)
C(51)-C(47)-C(46)	111.3(4)
C(48)-C(47)-C(46)	108.8(4)
C(51)-C(47)-H(47)	108.1
C(48)-C(47)-H(47)	108.1
C(46)-C(47)-H(47)	108.1
C(49)-C(48)-C(47)	112.5(4)
C(49)-C(48)-H(48A)	109.1

C(47)-C(48)-H(48A)	109.1
C(49)-C(48)-H(48B)	109.1
C(47)-C(48)-H(48B)	109.1
H(48A)-C(48)-H(48B)	107.8
C(48)-C(49)-C(50)	112.1(4)
C(48)-C(49)-H(49A)	109.2
C(50)-C(49)-H(49A)	109.2
C(48)-C(49)-H(49B)	109.2
C(50)-C(49)-H(49B)	109.2
H(49A)-C(49)-H(49B)	107.9
C(49)-C(50)-C(45)	107.7(4)
C(49)-C(50)-C(52)	114.5(4)
C(45)-C(50)-C(52)	113.2(4)
C(49)-C(50)-H(50)	107.0
C(45)-C(50)-H(50)	107.0
C(52)-C(50)-H(50)	107.0
C(47)-C(51)-H(51A)	109.5
C(47)-C(51)-H(51B)	109.5
H(51A)-C(51)-H(51B)	109.5
C(47)-C(51)-H(51C)	109.5
H(51A)-C(51)-H(51C)	109.5
H(51B)-C(51)-H(51C)	109.5
C(53)-C(52)-C(54)	109.7(5)
C(53)-C(52)-C(50)	114.0(5)
C(54)-C(52)-C(50)	110.9(4)
C(53)-C(52)-H(52)	107.3
C(54)-C(52)-H(52)	107.3
C(50)-C(52)-H(52)	107.3
C(52)-C(53)-H(53A)	109.5
C(52)-C(53)-H(53B)	109.5
H(53A)-C(53)-H(53B)	109.5
C(52)-C(53)-H(53C)	109.5
H(53A)-C(53)-H(53C)	109.5
H(53B)-C(53)-H(53C)	109.5
C(52)-C(54)-H(54A)	109.5
C(52)-C(54)-H(54B)	109.5
H(54A)-C(54)-H(54B)	109.5

C(52)-C(54)-H(54C)	109.5
H(54A)-C(54)-H(54C)	109.5
H(54B)-C(54)-H(54C)	109.5
O(8)-C(61)-O(7)	124.9(4)
O(8)-C(61)-C(62)	125.5(4)
O(7)-C(61)-C(62)	109.4(4)
C(67)-C(62)-C(72)	99.9(4)
C(67)-C(62)-C(63)	112.1(4)
C(72)-C(62)-C(63)	107.8(4)
C(67)-C(62)-C(61)	113.0(4)
C(72)-C(62)-C(61)	117.0(4)
C(63)-C(62)-C(61)	106.9(4)
O(9)-C(63)-C(64)	124.5(5)
O(9)-C(63)-C(62)	122.5(5)
C(64)-C(63)-C(62)	112.8(4)
C(63)-C(64)-C(74)	106.3(4)
C(63)-C(64)-C(65)	109.0(4)
C(74)-C(64)-C(65)	107.9(4)
C(63)-C(64)-H(64)	111.2
C(74)-C(64)-H(64)	111.2
C(65)-C(64)-H(64)	111.2
C(66)-C(65)-C(64)	121.3(5)
C(66)-C(65)-H(65)	119.4
C(64)-C(65)-H(65)	119.4
C(65)-C(66)-C(67)	123.2(5)
C(65)-C(66)-H(66)	118.4
C(67)-C(66)-H(66)	118.4
C(68)-C(67)-C(66)	123.1(5)
C(68)-C(67)-C(62)	118.3(4)
C(66)-C(67)-C(62)	118.2(4)
C(67)-C(68)-C(69)	125.6(5)
C(67)-C(68)-H(68)	117.2
C(69)-C(68)-H(68)	117.2
C(70)-C(69)-C(68)	124.5(5)
C(70)-C(69)-H(69)	117.7
C(68)-C(69)-H(69)	117.7
C(69)-C(70)-C(71)	125.6(5)

C(69)-C(70)-H(70)	117.2
C(71)-C(70)-H(70)	117.2
C(72)-C(71)-C(70)	125.3(5)
C(72)-C(71)-H(71)	117.4
C(70)-C(71)-H(71)	117.4
C(71)-C(72)-C(73)	122.5(4)
C(71)-C(72)-C(62)	119.7(4)
C(73)-C(72)-C(62)	117.0(4)
C(74)-C(73)-C(72)	123.5(5)
C(74)-C(73)-H(73)	118.3
C(72)-C(73)-H(73)	118.3
C(73)-C(74)-C(64)	120.9(5)
C(73)-C(74)-H(74)	119.6
C(64)-C(74)-H(74)	119.6
O(7)-C(75)-C(76)	109.3(4)
O(7)-C(75)-C(80)	106.7(4)
C(76)-C(75)-C(80)	113.0(4)
O(7)-C(75)-H(75)	109.2
C(76)-C(75)-H(75)	109.2
C(80)-C(75)-H(75)	109.2
C(75)-C(76)-C(77)	113.1(4)
C(75)-C(76)-H(76A)	109.0
C(77)-C(76)-H(76A)	109.0
C(75)-C(76)-H(76B)	109.0
C(77)-C(76)-H(76B)	109.0
H(76A)-C(76)-H(76B)	107.8
C(78)-C(77)-C(81)	111.5(5)
C(78)-C(77)-C(76)	110.4(5)
C(81)-C(77)-C(76)	111.6(4)
C(78)-C(77)-H(77)	107.7
C(81)-C(77)-H(77)	107.7
C(76)-C(77)-H(77)	107.7
C(77)-C(78)-C(79)	112.6(4)
C(77)-C(78)-H(78A)	109.1
C(79)-C(78)-H(78A)	109.1
C(77)-C(78)-H(78B)	109.1
C(79)-C(78)-H(78B)	109.1

H(78A)-C(78)-H(78B)	107.8
C(78)-C(79)-C(80)	112.5(4)
C(78)-C(79)-H(79A)	109.1
C(80)-C(79)-H(79A)	109.1
C(78)-C(79)-H(79B)	109.1
C(80)-C(79)-H(79B)	109.1
H(79A)-C(79)-H(79B)	107.8
C(79)-C(80)-C(82)	114.6(4)
C(79)-C(80)-C(75)	107.9(4)
C(82)-C(80)-C(75)	114.3(4)
C(79)-C(80)-H(80)	106.5
C(82)-C(80)-H(80)	106.5
C(75)-C(80)-H(80)	106.5
C(77)-C(81)-H(81A)	109.5
C(77)-C(81)-H(81B)	109.5
H(81A)-C(81)-H(81B)	109.5
C(77)-C(81)-H(81C)	109.5
H(81A)-C(81)-H(81C)	109.5
H(81B)-C(81)-H(81C)	109.5
C(84)-C(82)-C(83)	110.6(5)
C(84)-C(82)-C(80)	110.8(4)
C(83)-C(82)-C(80)	113.8(5)
C(84)-C(82)-H(82)	107.1
C(83)-C(82)-H(82)	107.1
C(80)-C(82)-H(82)	107.1
C(82)-C(83)-H(83A)	109.5
C(82)-C(83)-H(83B)	109.5
H(83A)-C(83)-H(83B)	109.5
C(82)-C(83)-H(83C)	109.5
H(83A)-C(83)-H(83C)	109.5
H(83B)-C(83)-H(83C)	109.5
C(82)-C(84)-H(84A)	109.5
C(82)-C(84)-H(84B)	109.5
H(84A)-C(84)-H(84B)	109.5
C(82)-C(84)-H(84C)	109.5
H(84A)-C(84)-H(84C)	109.5
H(84B)-C(84)-H(84C)	109.5

O(11)-C(91)-O(10)	123.8(4)
O(11)-C(91)-C(92)	126.0(4)
O(10)-C(91)-C(92)	110.0(4)
C(91)-C(92)-C(97)	113.9(4)
C(91)-C(92)-C(102)	117.1(4)
C(97)-C(92)-C(102)	100.2(4)
C(91)-C(92)-C(93)	106.4(4)
C(97)-C(92)-C(93)	111.7(4)
C(102)-C(92)-C(93)	107.5(4)
O(12)-C(93)-C(94)	125.0(4)
O(12)-C(93)-C(92)	122.7(4)
C(94)-C(93)-C(92)	112.3(4)
C(93)-C(94)-C(95)	109.0(4)
C(93)-C(94)-C(104)	107.1(4)
C(95)-C(94)-C(104)	108.6(4)
C(93)-C(94)-H(94)	110.7
C(95)-C(94)-H(94)	110.7
C(104)-C(94)-H(94)	110.7
C(96)-C(95)-C(94)	122.1(5)
C(96)-C(95)-H(95)	118.9
C(94)-C(95)-H(95)	118.9
C(95)-C(96)-C(97)	122.1(5)
C(95)-C(96)-H(96)	119.0
C(97)-C(96)-H(96)	119.0
C(98)-C(97)-C(96)	123.2(4)
C(98)-C(97)-C(92)	118.6(4)
C(96)-C(97)-C(92)	117.9(4)
C(97)-C(98)-C(99)	124.8(5)
C(97)-C(98)-H(98)	117.6
C(99)-C(98)-H(98)	117.6
C(100)-C(99)-C(98)	125.8(5)
C(100)-C(99)-H(99)	117.1
C(98)-C(99)-H(99)	117.1
C(99)-C(100)-C(01G)	125.0(5)
C(99)-C(100)-H(100)	117.5
C(01G)-C(100)-H(100)	117.5
C(01G)-C(102)-C(103)	122.9(4)

C(01G)-C(102)-C(92)	118.4(4)
C(103)-C(102)-C(92)	118.1(4)
C(104)-C(103)-C(102)	123.0(4)
C(104)-C(103)-H(103)	118.5
C(102)-C(103)-H(103)	118.5
C(103)-C(104)-C(94)	120.7(4)
C(103)-C(104)-H(104)	119.6
C(94)-C(104)-H(104)	119.6
O(10)-C(105)-C(106)	109.0(4)
O(10)-C(105)-C(110)	106.4(4)
C(106)-C(105)-C(110)	112.0(4)
O(10)-C(105)-H(105)	109.8
C(106)-C(105)-H(105)	109.8
C(110)-C(105)-H(105)	109.8
C(105)-C(106)-C(107)	110.6(4)
C(105)-C(106)-H(10E)	109.5
C(107)-C(106)-H(10E)	109.5
C(105)-C(106)-H(10F)	109.5
C(107)-C(106)-H(10F)	109.5
H(10E)-C(106)-H(10F)	108.1
C(108)-C(107)-C(106)	109.5(5)
C(108)-C(107)-C(111)	113.0(5)
C(106)-C(107)-C(111)	110.2(5)
C(108)-C(107)-H(107)	108.0
C(106)-C(107)-H(107)	108.0
C(111)-C(107)-H(107)	108.0
C(107)-C(108)-C(109)	111.8(5)
C(107)-C(108)-H(10G)	109.3
C(109)-C(108)-H(10G)	109.2
C(107)-C(108)-H(10H)	109.2
C(109)-C(108)-H(10H)	109.2
H(10G)-C(108)-H(10H)	107.9
C(108)-C(109)-C(110)	112.7(5)
C(108)-C(109)-H(10I)	109.0
C(110)-C(109)-H(10I)	109.0
C(108)-C(109)-H(10J)	109.0
C(110)-C(109)-H(10J)	109.0

H(10I)-C(109)-H(10J)	107.8
C(105)-C(110)-C(109)	107.9(4)
C(105)-C(110)-C(112)	112.7(4)
C(109)-C(110)-C(112)	114.0(4)
C(105)-C(110)-H(110)	107.3
C(109)-C(110)-H(110)	107.3
C(112)-C(110)-H(110)	107.3
C(107)-C(111)-H(11A)	109.5
C(107)-C(111)-H(11B)	109.5
H(11A)-C(111)-H(11B)	109.5
C(107)-C(111)-H(11C)	109.5
H(11A)-C(111)-H(11C)	109.5
H(11B)-C(111)-H(11C)	109.5
C(113)-C(112)-C(114)	110.0(5)
C(113)-C(112)-C(110)	112.1(5)
C(114)-C(112)-C(110)	113.8(5)
C(113)-C(112)-H(112)	106.9
C(114)-C(112)-H(112)	106.9
C(110)-C(112)-H(112)	106.9
C(112)-C(113)-H(11D)	109.5
C(112)-C(113)-H(11E)	109.5
H(11D)-C(113)-H(11E)	109.5
C(112)-C(113)-H(11F)	109.5
H(11D)-C(113)-H(11F)	109.5
H(11E)-C(113)-H(11F)	109.5
C(112)-C(114)-H(11G)	109.5
C(112)-C(114)-H(11H)	109.5
H(11G)-C(114)-H(11H)	109.5
C(112)-C(114)-H(11I)	109.5
H(11G)-C(114)-H(11I)	109.5
H(11H)-C(114)-H(11I)	109.5
C(1)-O(1)-C(16)	116.4(4)
C(31)-O(4)-C(45)	117.5(4)
C(61)-O(7)-C(75)	118.6(4)
C(91)-O(10)-C(105)	118.8(4)

2. Spectra

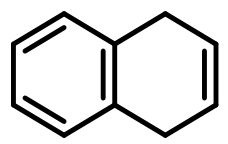

SI1

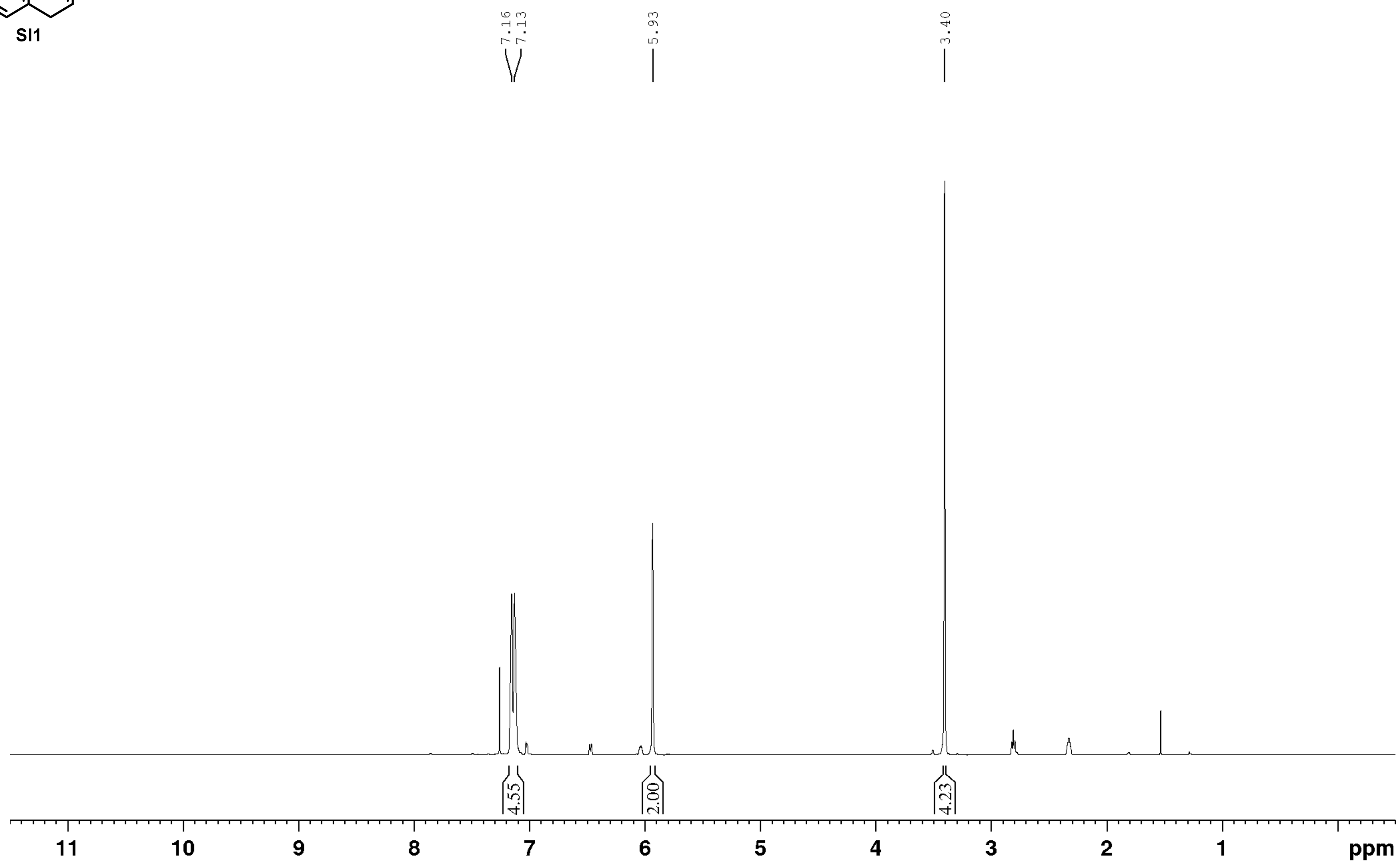

Figure S1: ^{1}H NMR spectrum of **SI1**.

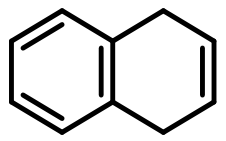

SI1

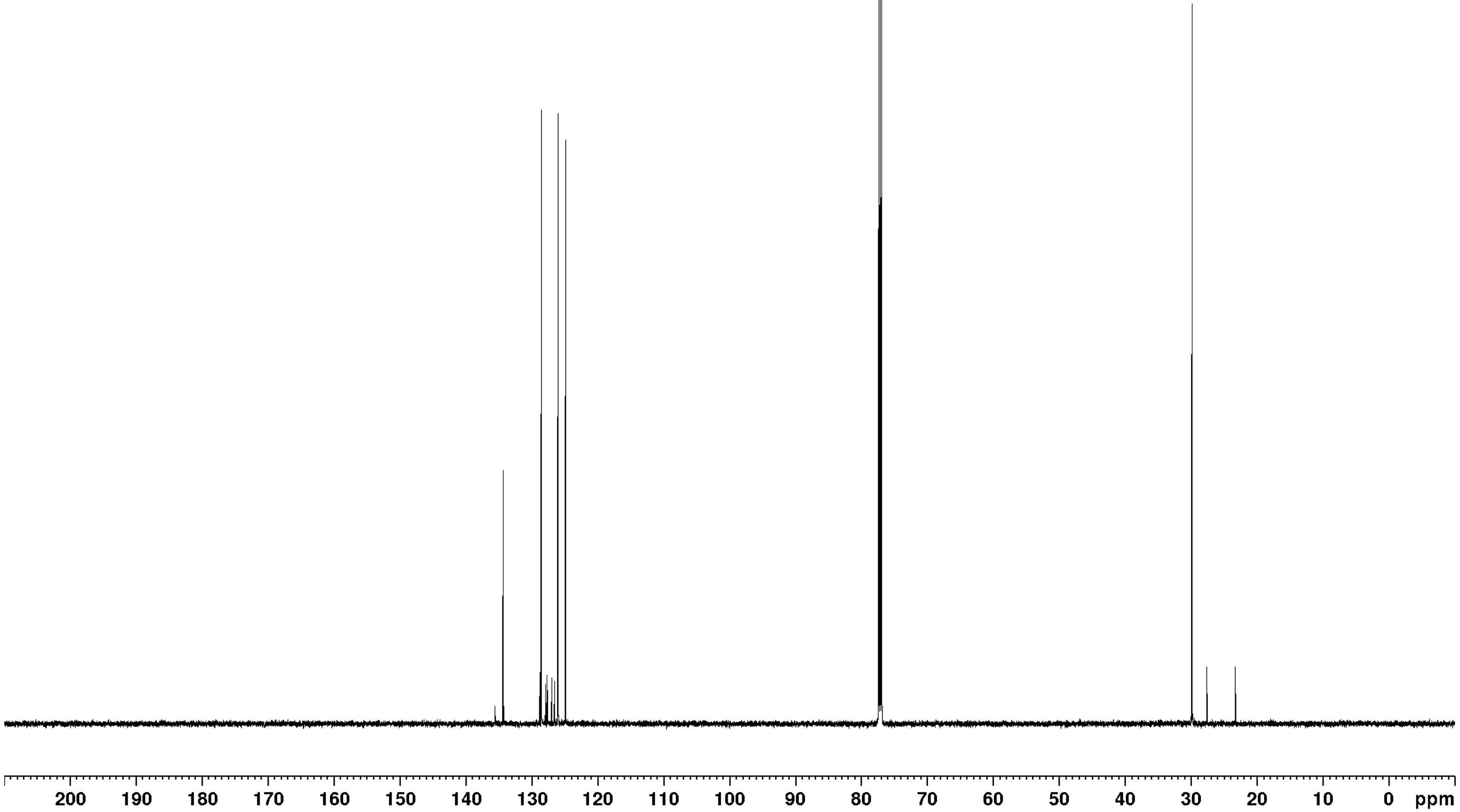

Figure S2: ^{13}C NMR spectrum of **SI1**.

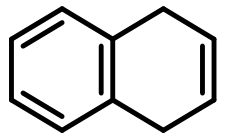

SI1

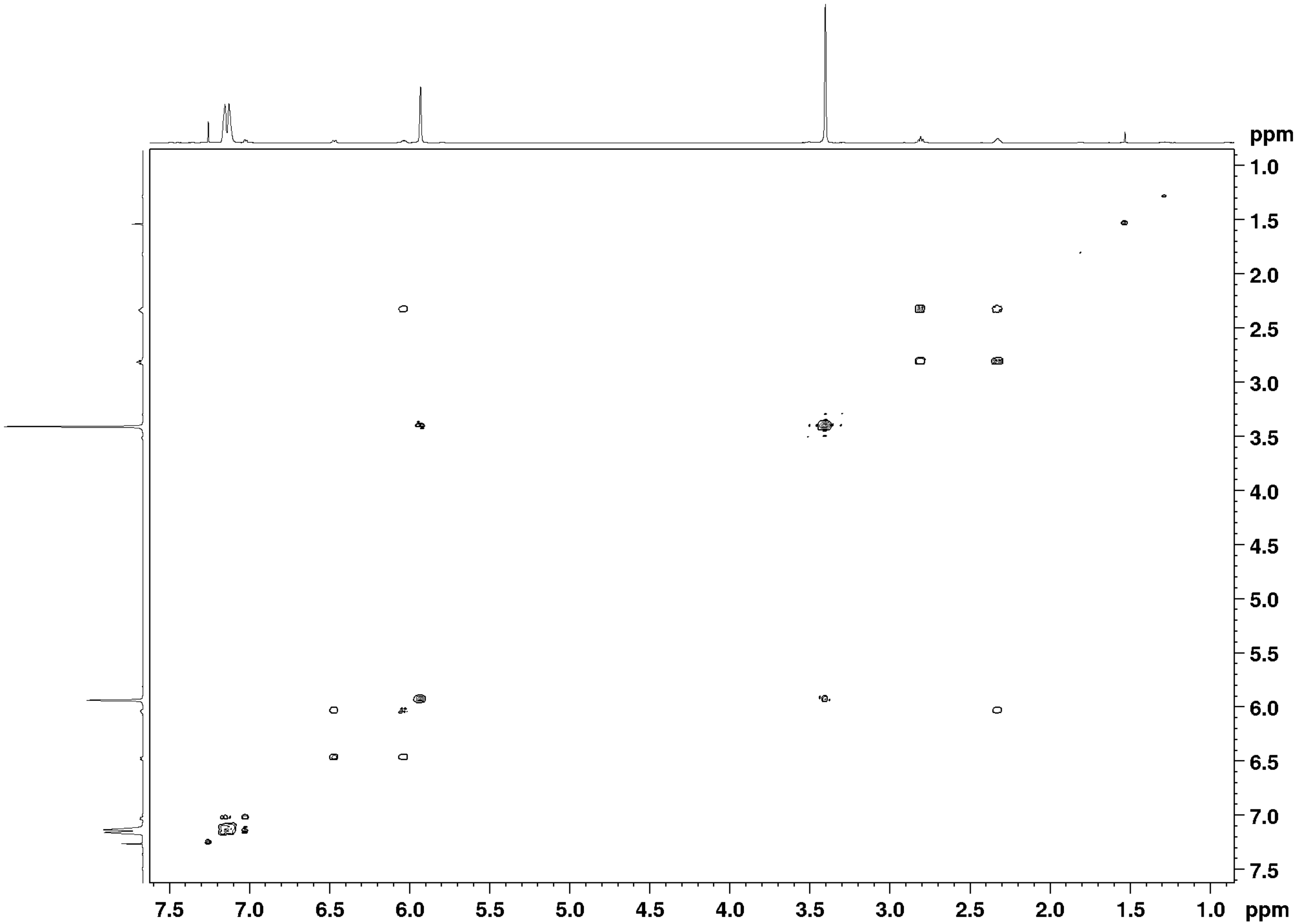

Figure S3: ^{1}H, ^{1}H COSY NMR spectrum of **SI1**.

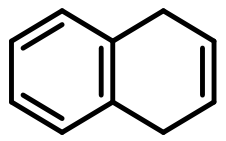

SI1

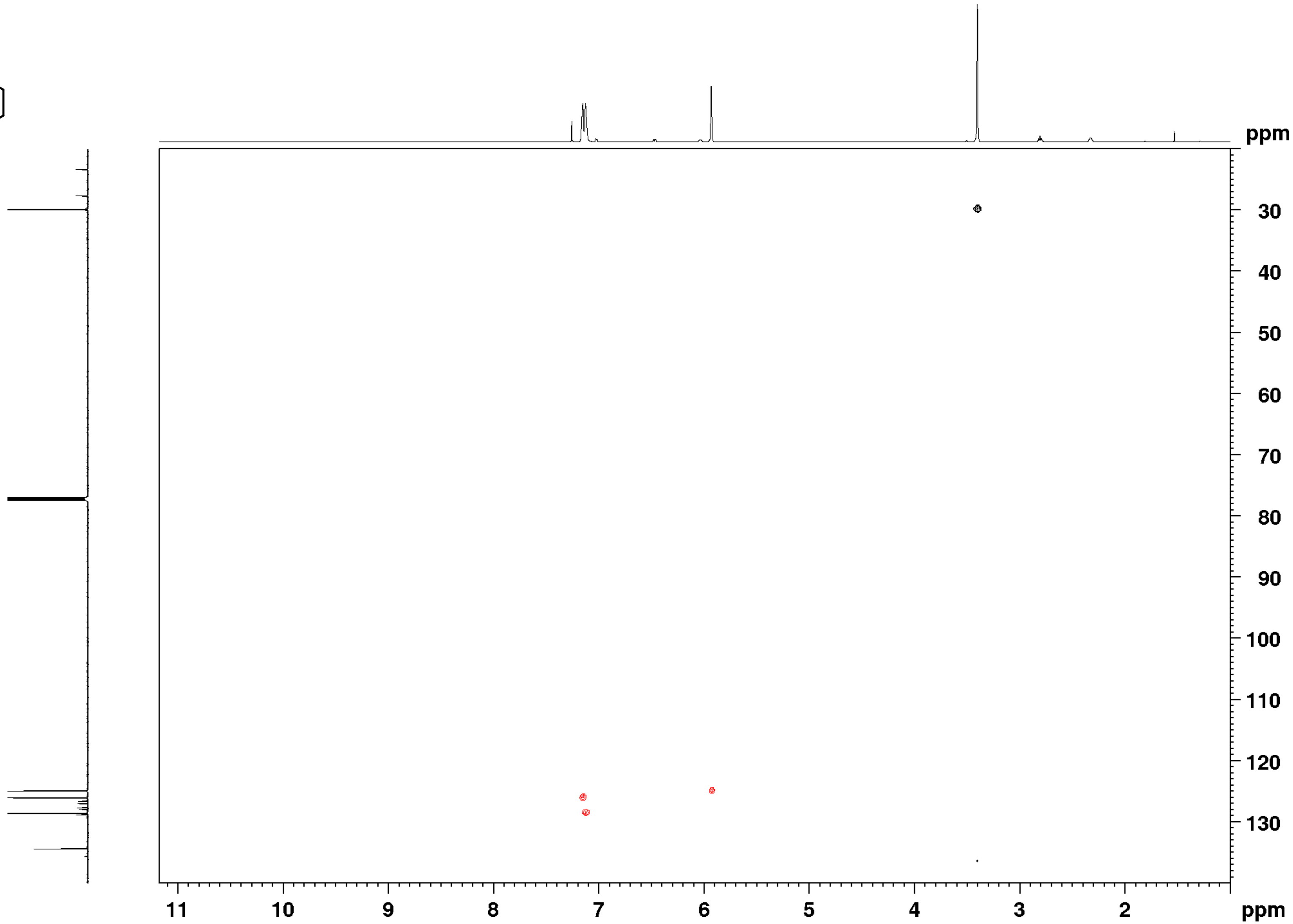

Figure S4: ^{1}H, ^{13}C HSQC spectrum of **SI1**.

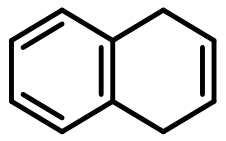

SI1

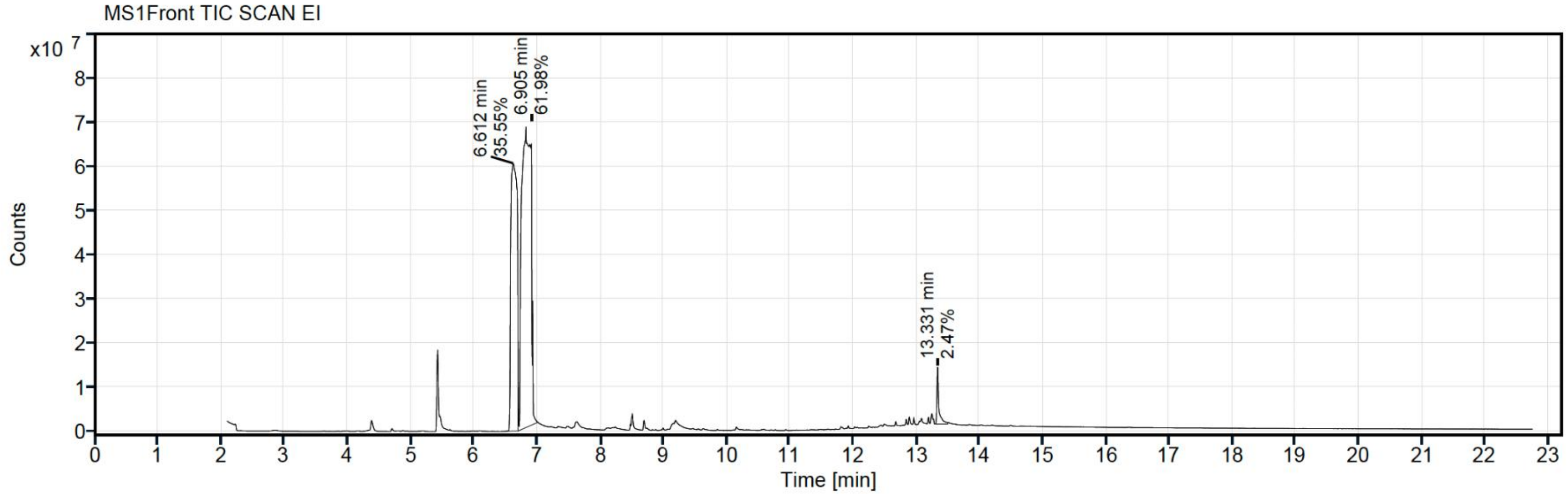

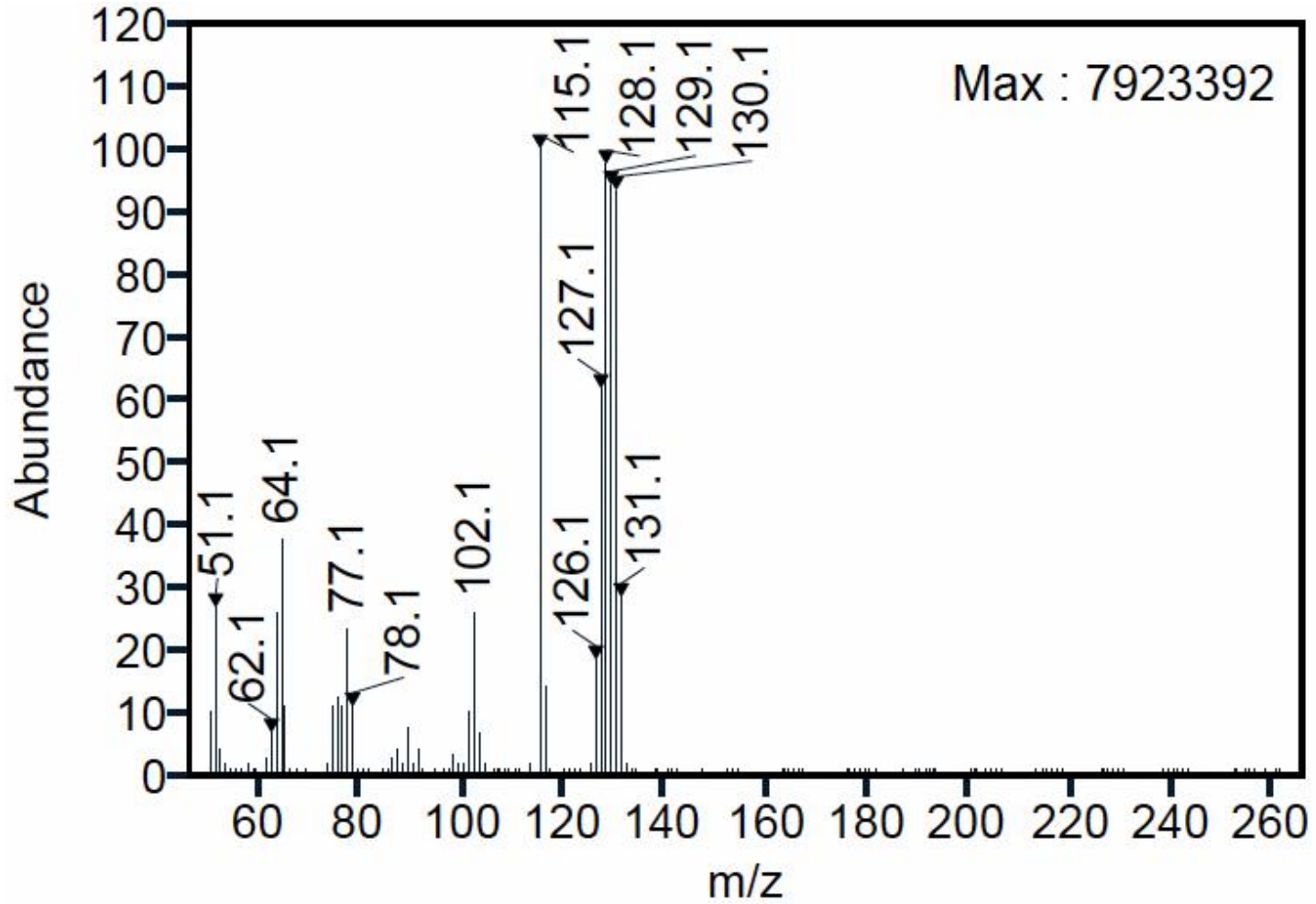

Figure S5: GC-MS TIC Scan and GC-MS spectrum of **SI1**.

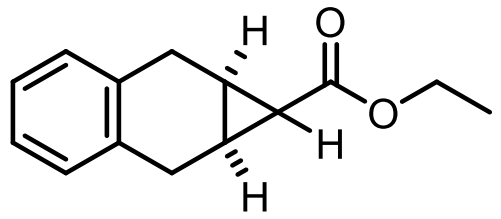

SI2

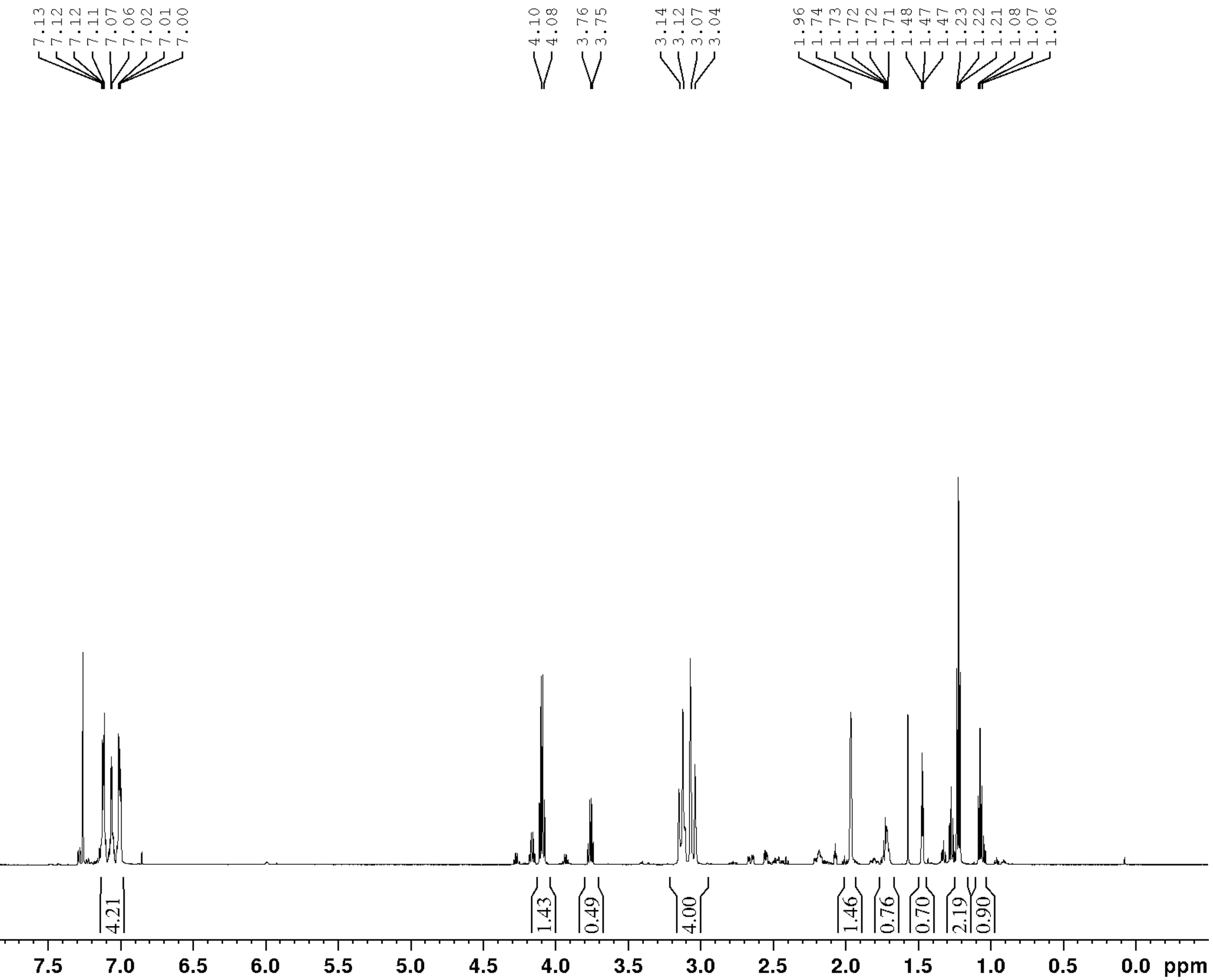

Figure S6: ^{1}H NMR spectrum of **SI2**.

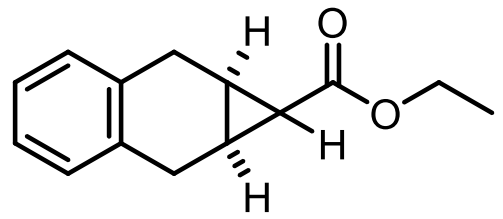

SI2

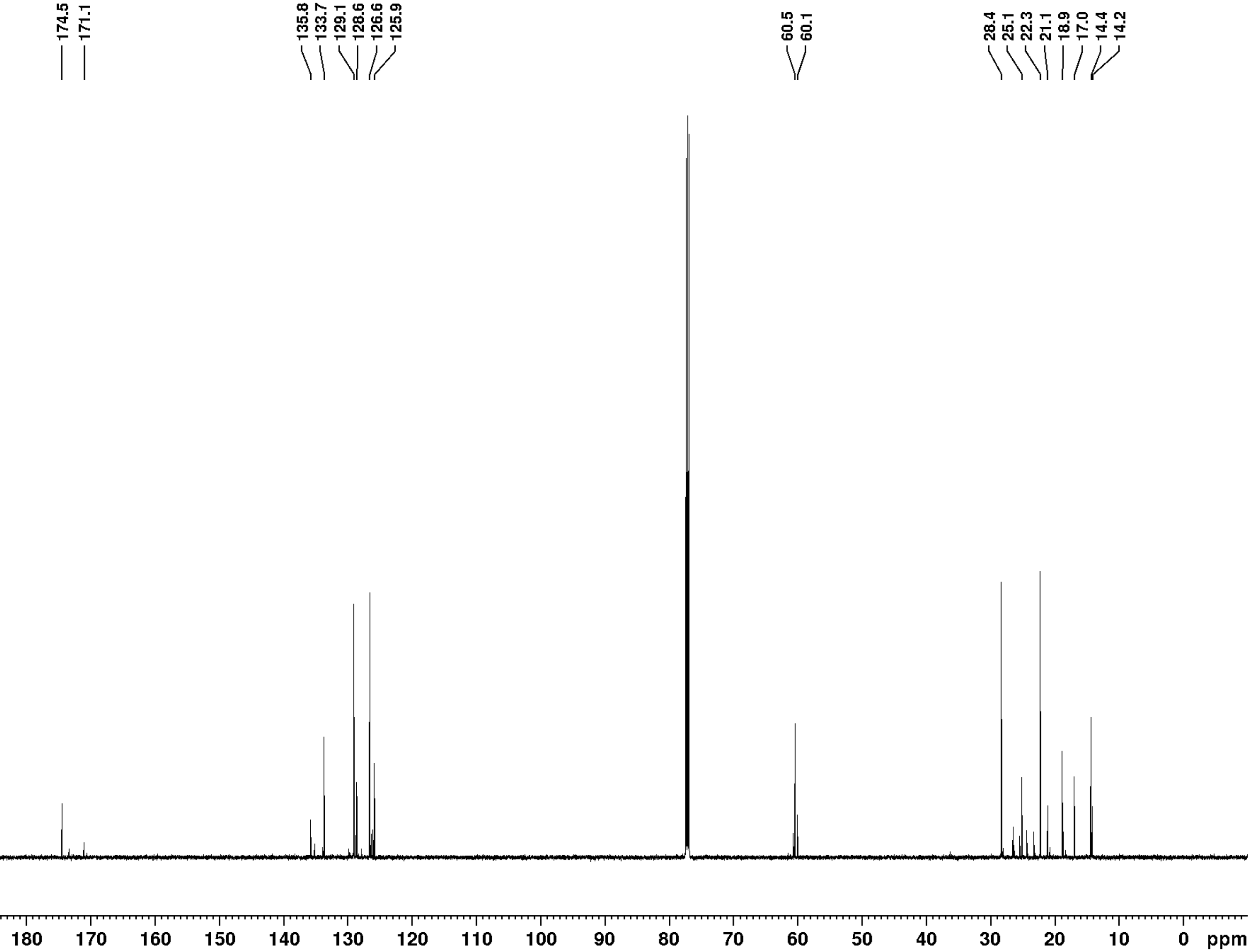

Figure S7: ^{13}C NMR spectrum of **SI2**.

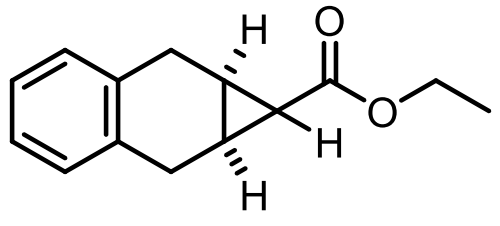

SI2

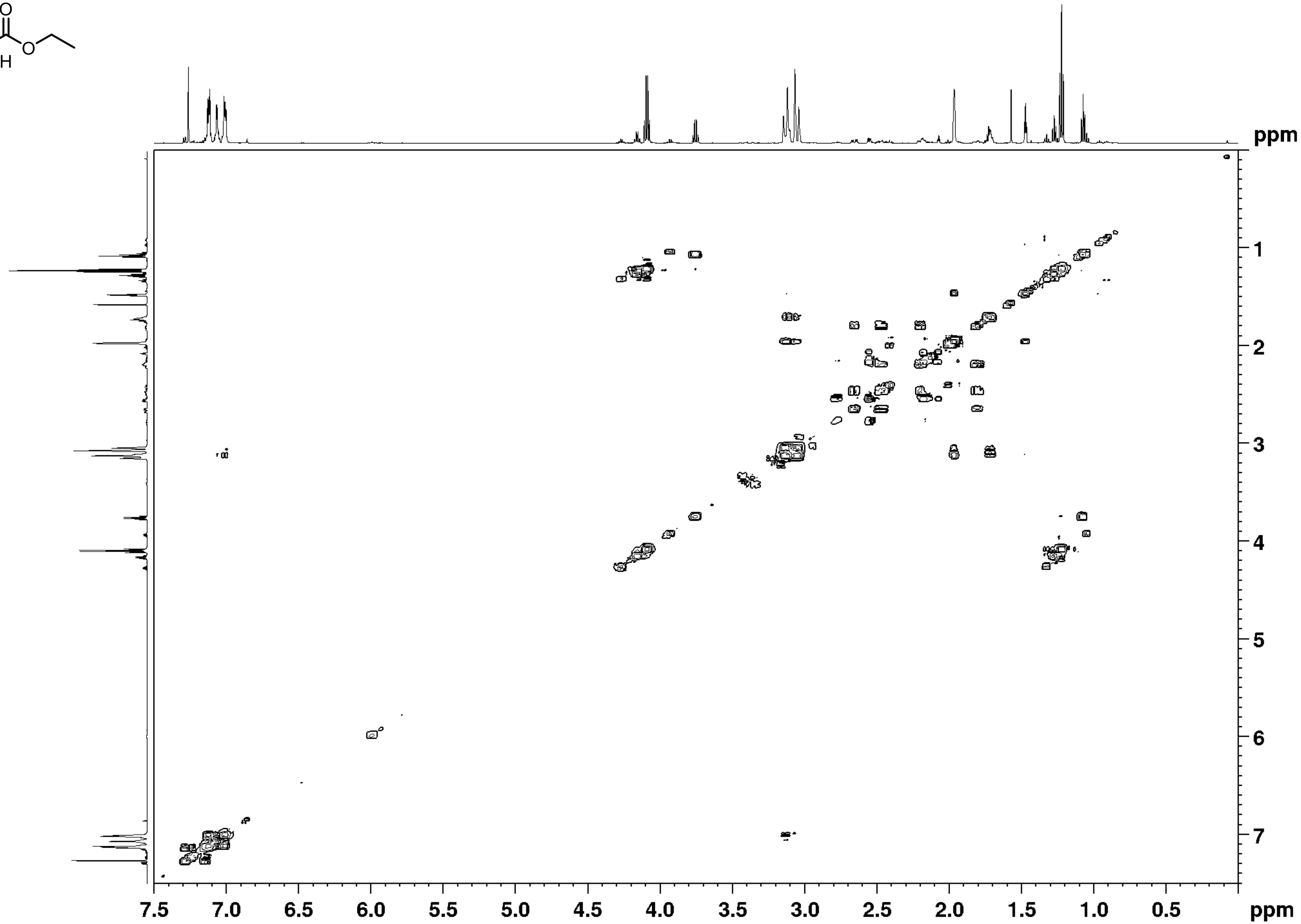

Figure S8: ^{1}H, ^{1}H COSY spectrum of **SI2**.

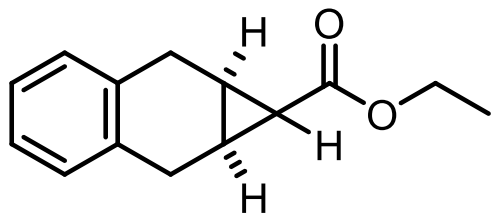

SI2

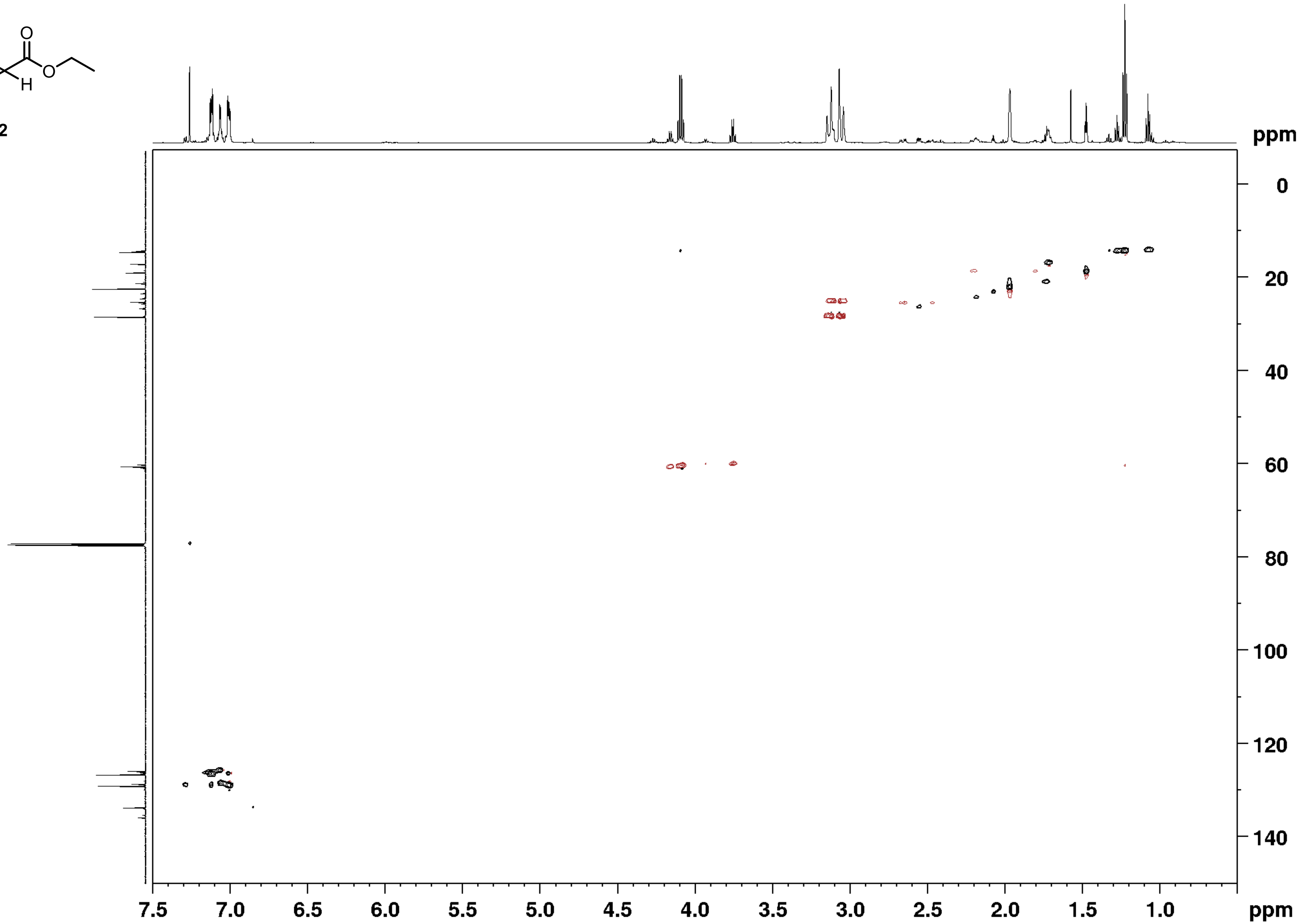

Figure S9: ^{1}H, ^{13}C HSQC spectrum of **SI2**.

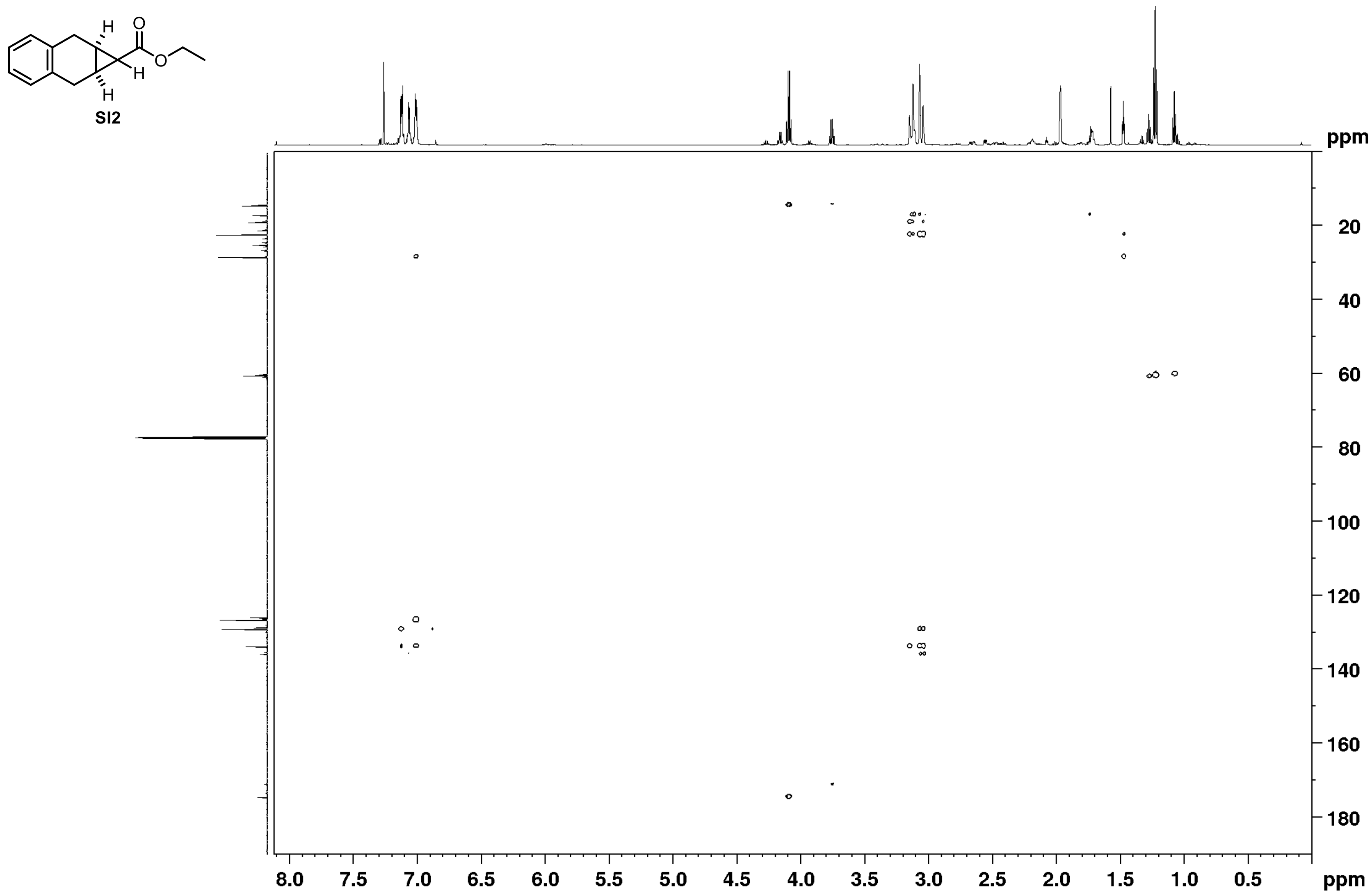

Figure S10: ^{1}H, ^{13}C HMBC spectrum of **SI2**.

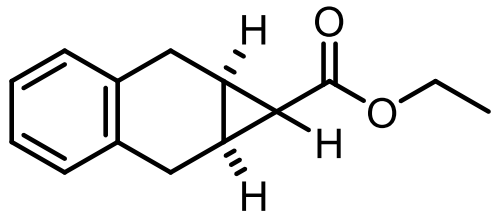

SI2

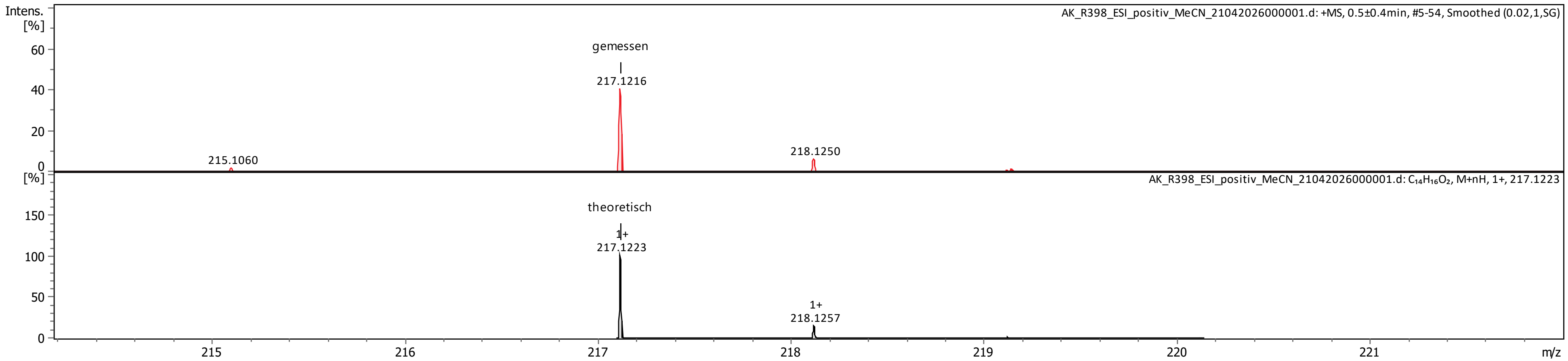

Figure.2.11: HRMS chromatogram of **SI2**.

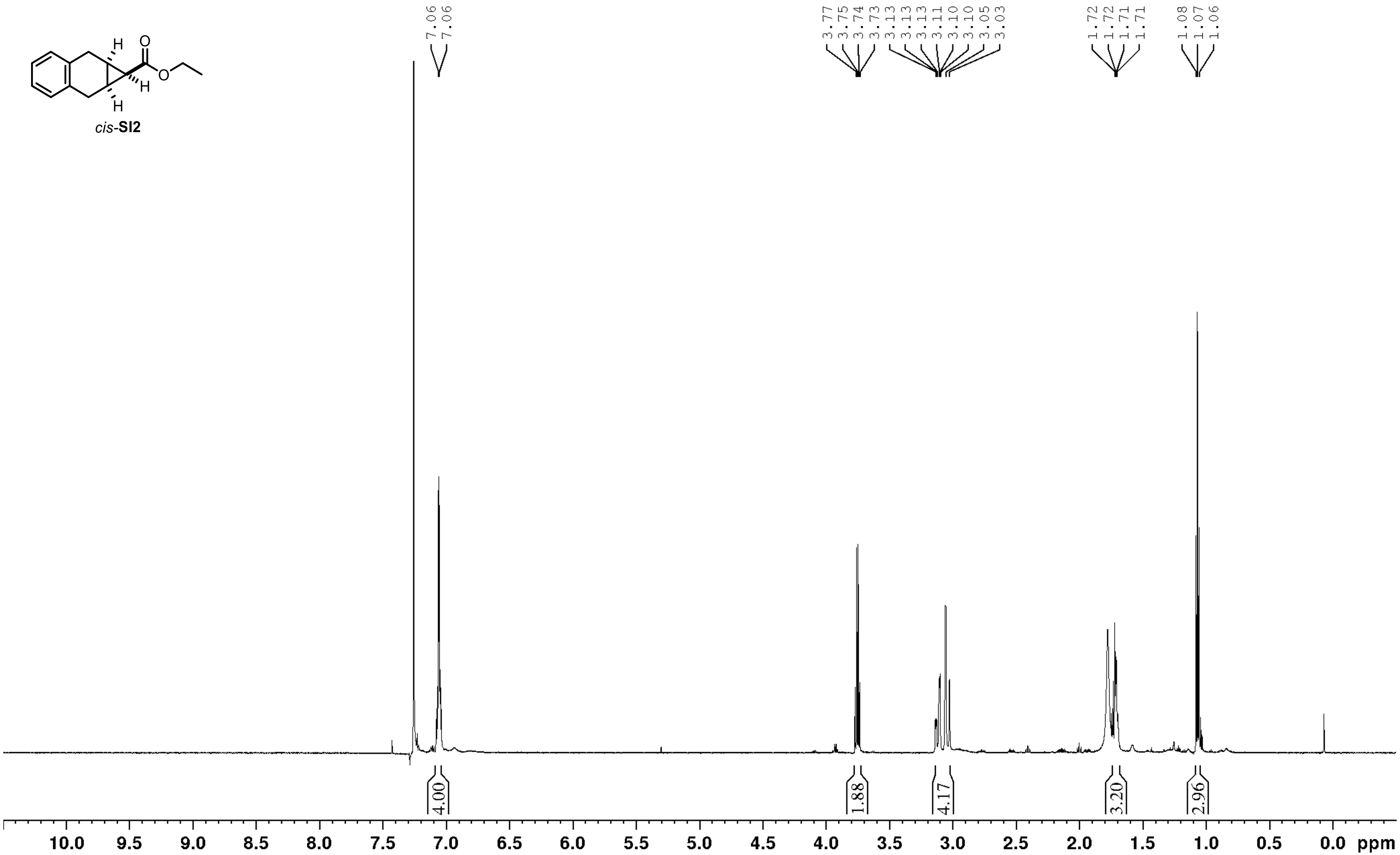

Figure S12: ^{1}H NMR of *cis*-**SI2**.

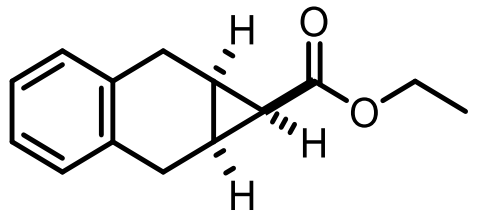

cis-**SI2**

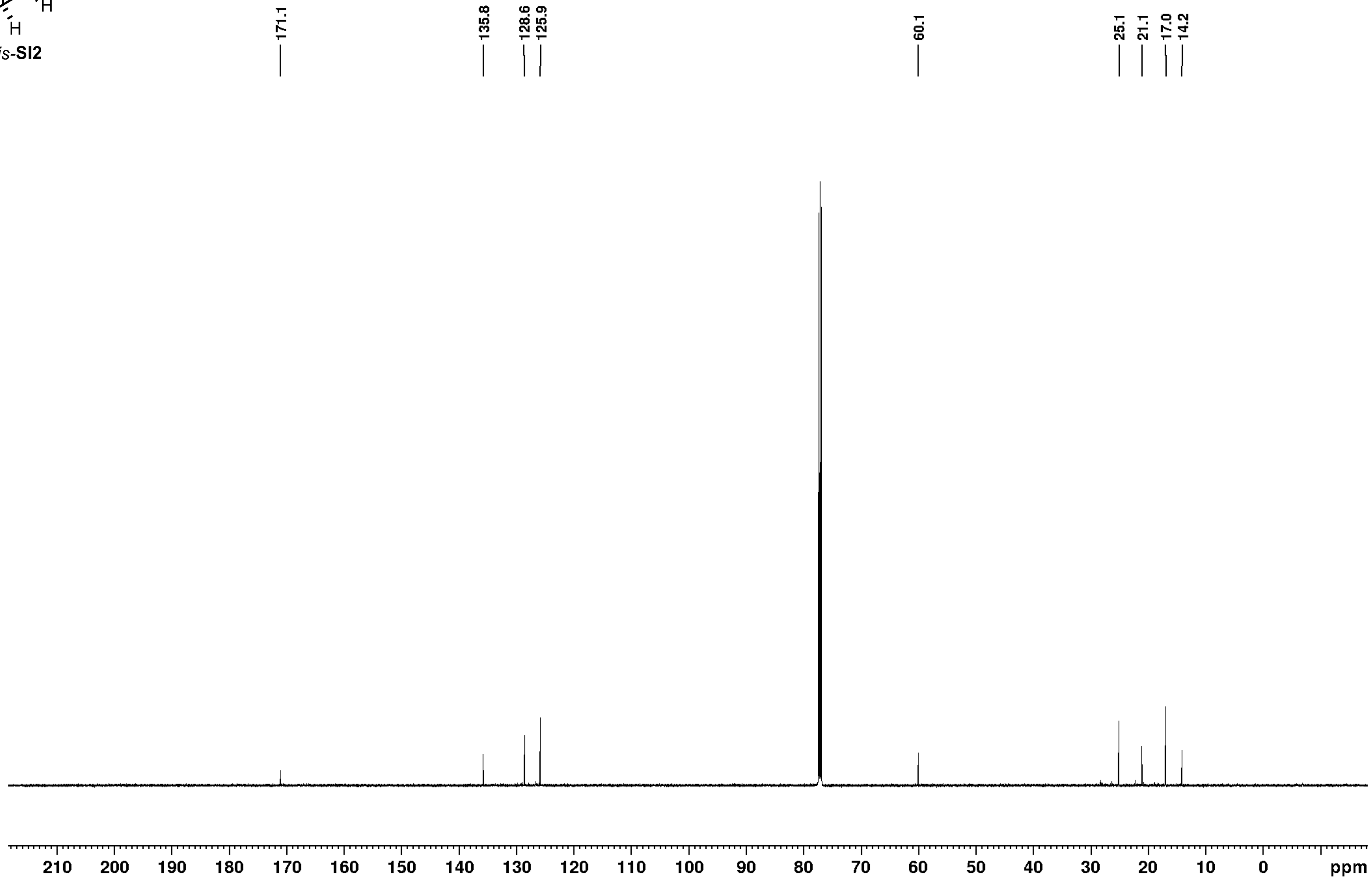

Figure S13: ^{13}C NMR spectrum of *cis*-**SI2**.

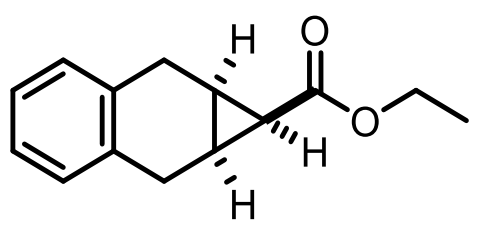

cis-**SI2**

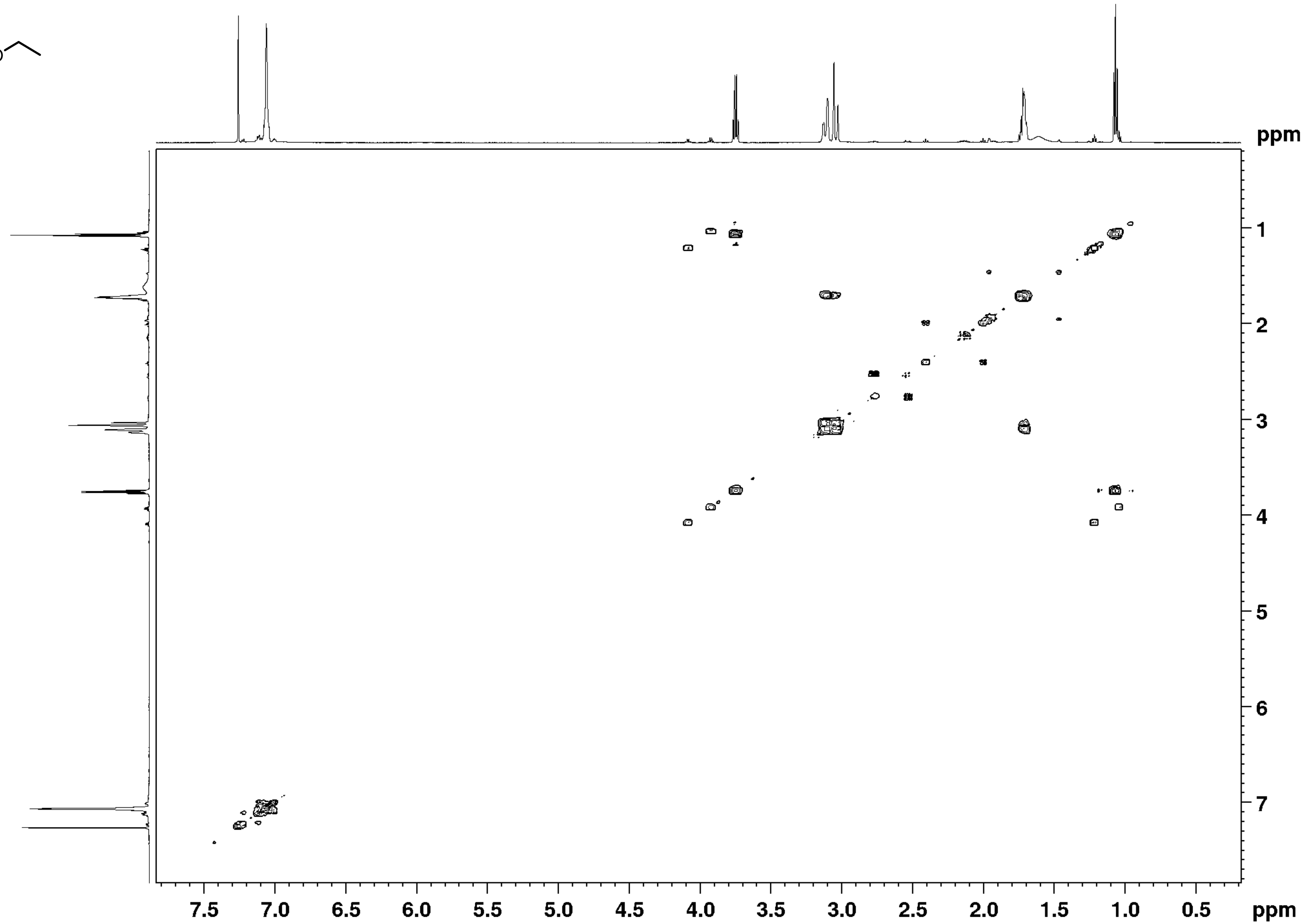

Figure S14: ^{1}H, ^{1}H COSY spectrum of *cis*-**SI2**.

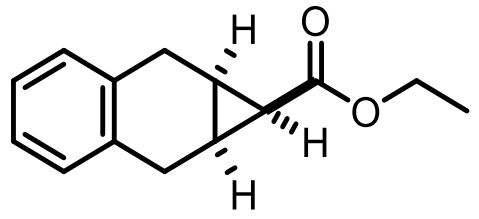

cis-**SI2**

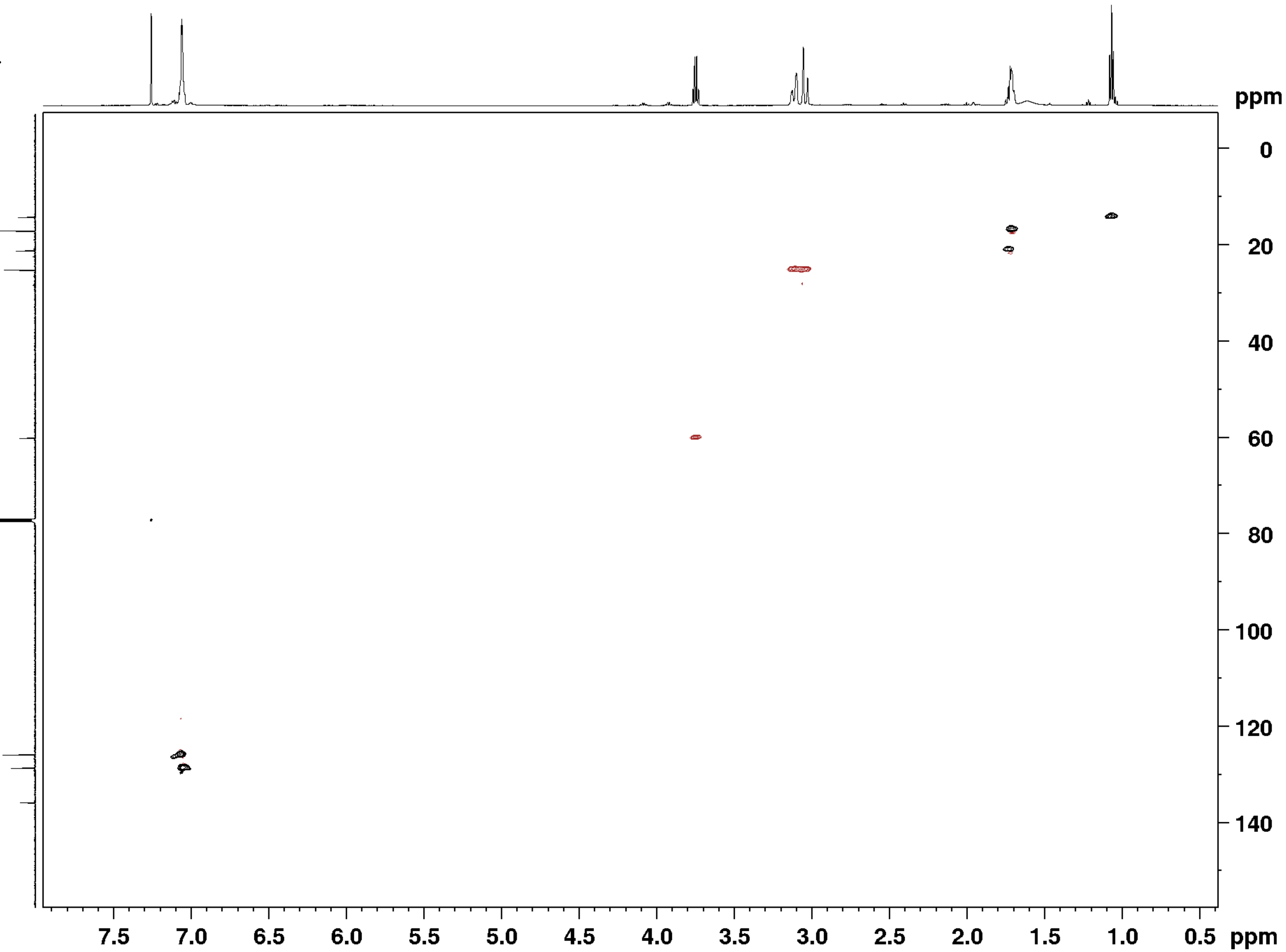

Figure S15: ^{1}H, ^{13}C HSQC spectrum of *cis*-**SI2**.

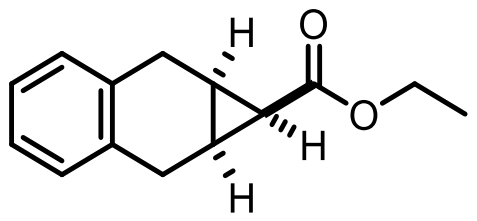

cis-**SI2**

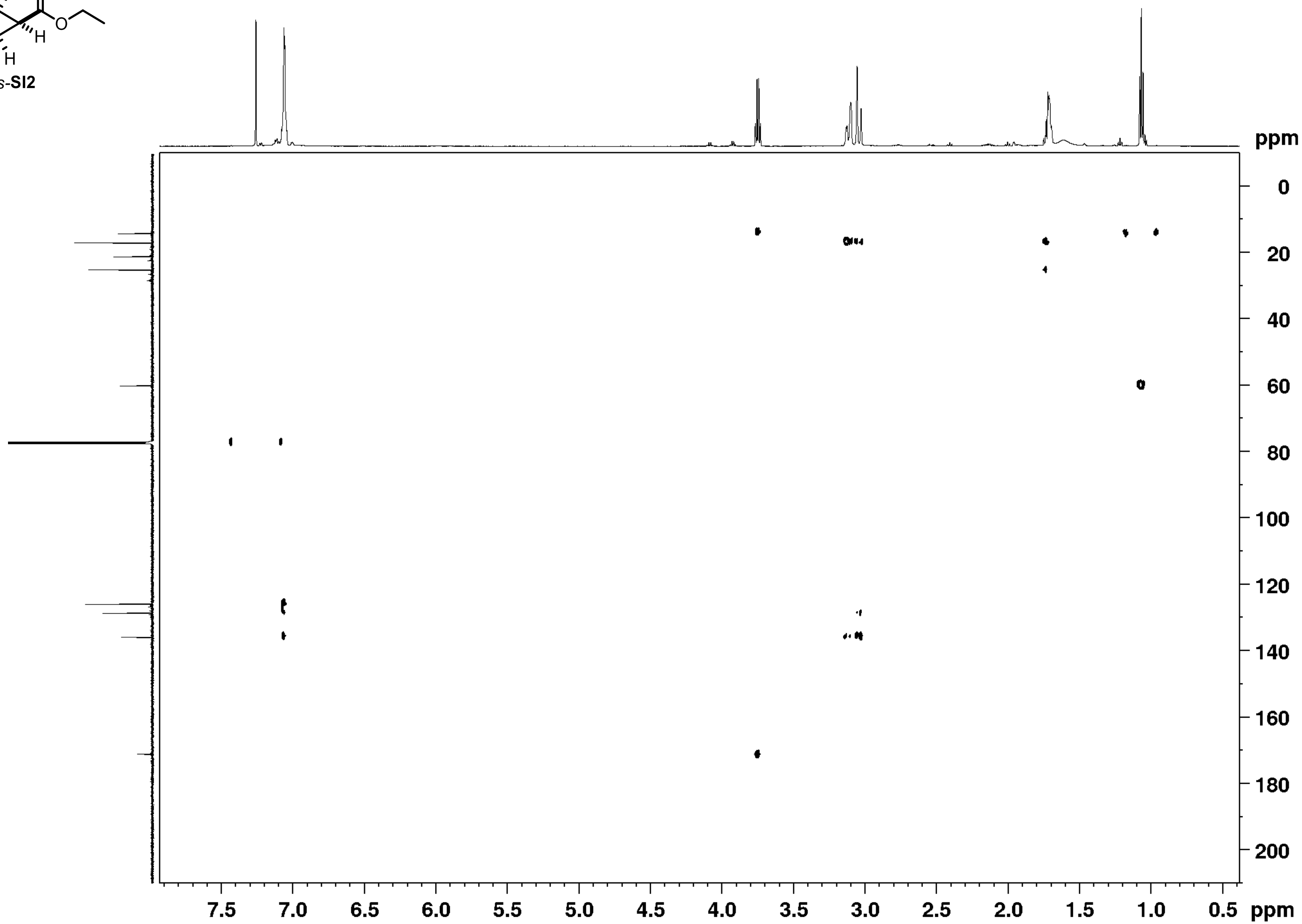

Figure S16: ^{1}H, ^{13}C HMBC spectrum of *cis*-**SI2**.

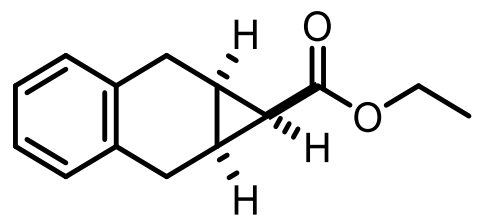

cis-**SI2**

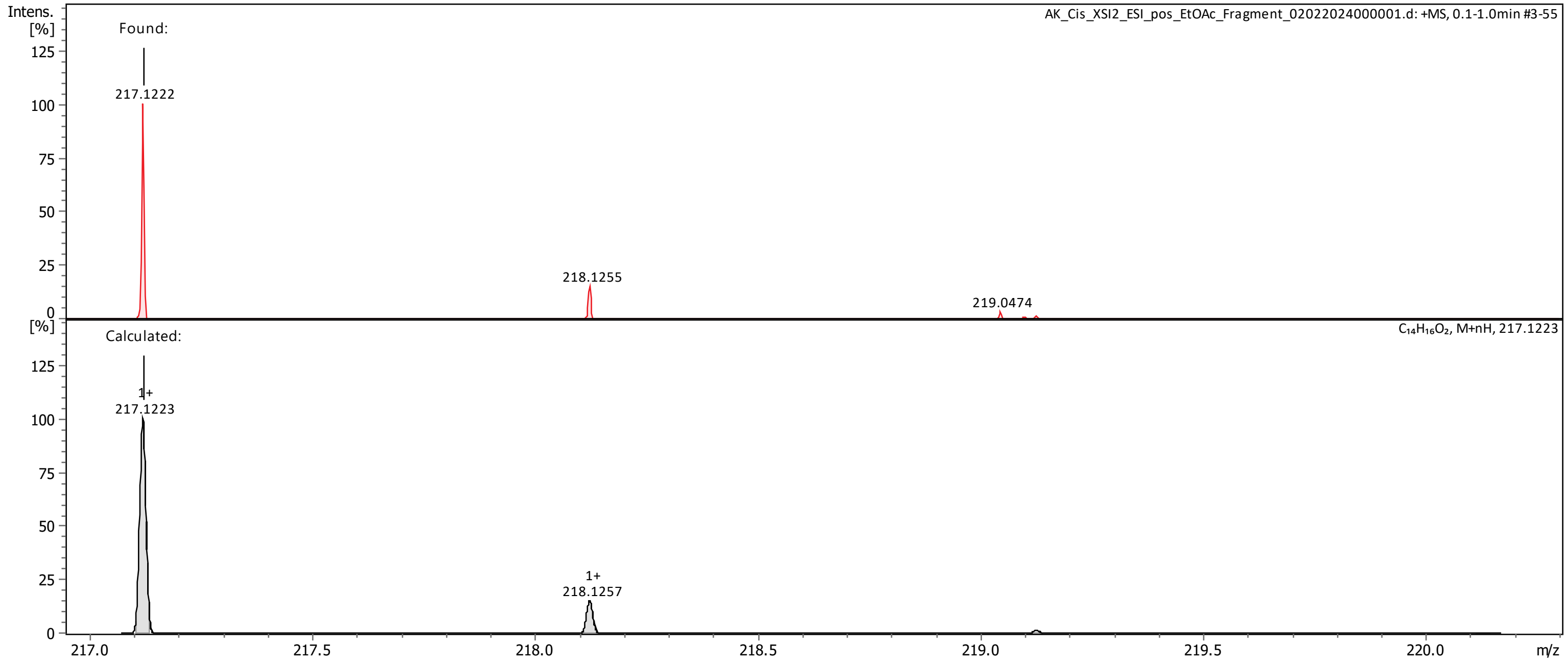

Figure S17: HRMS spectrum of *cis*-**SI2**.

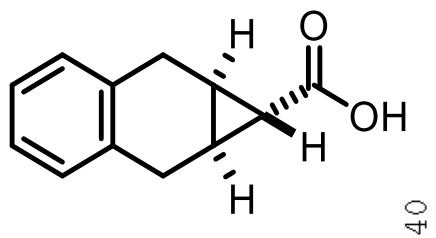

trans-**9**

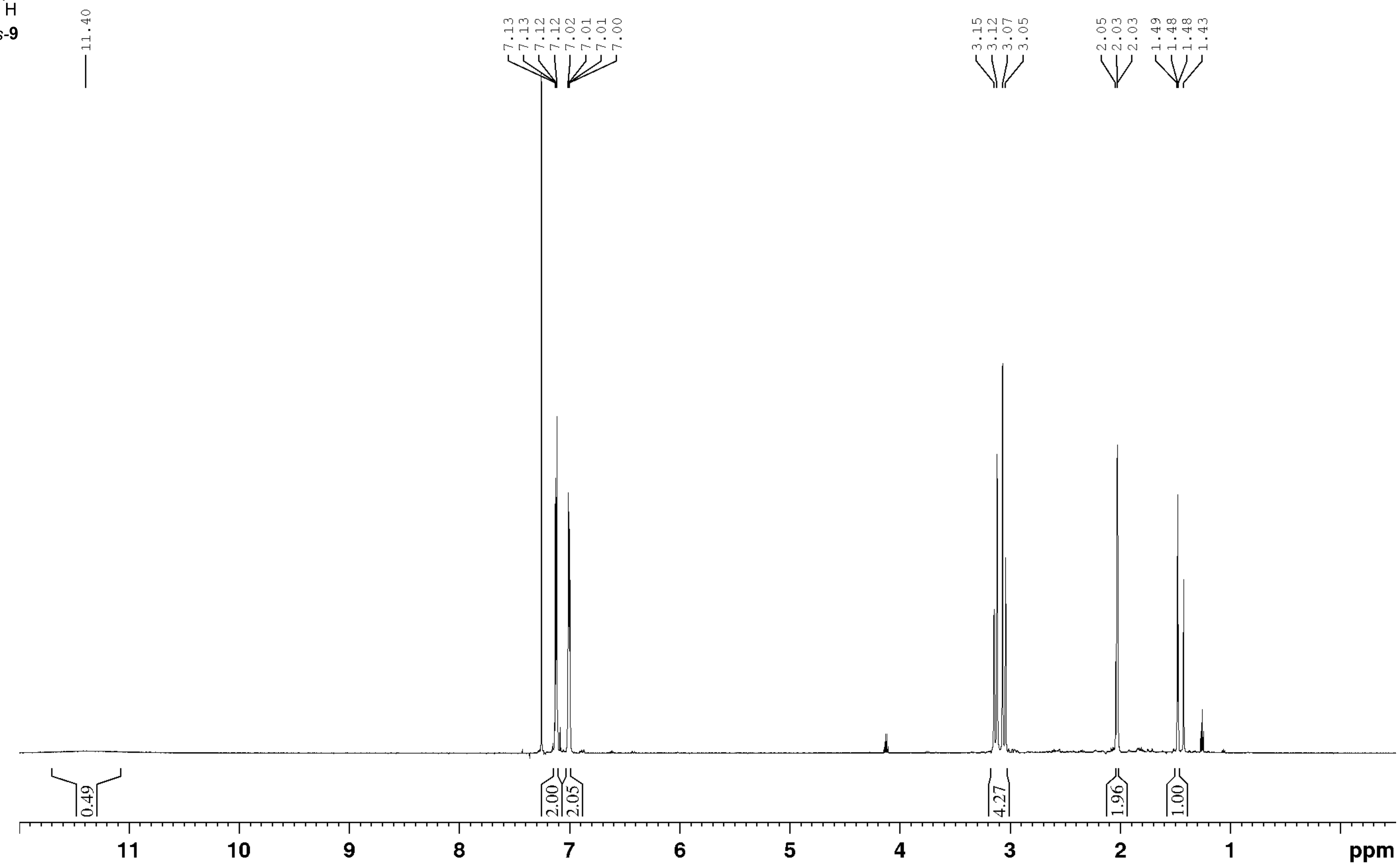

Figure S18: ^{1}H NMR spectrum of *trans*-**9**.

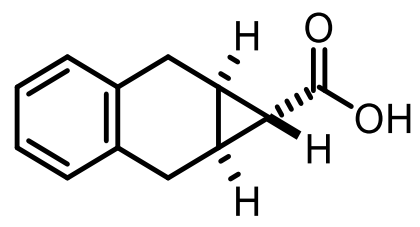
trans-**9**

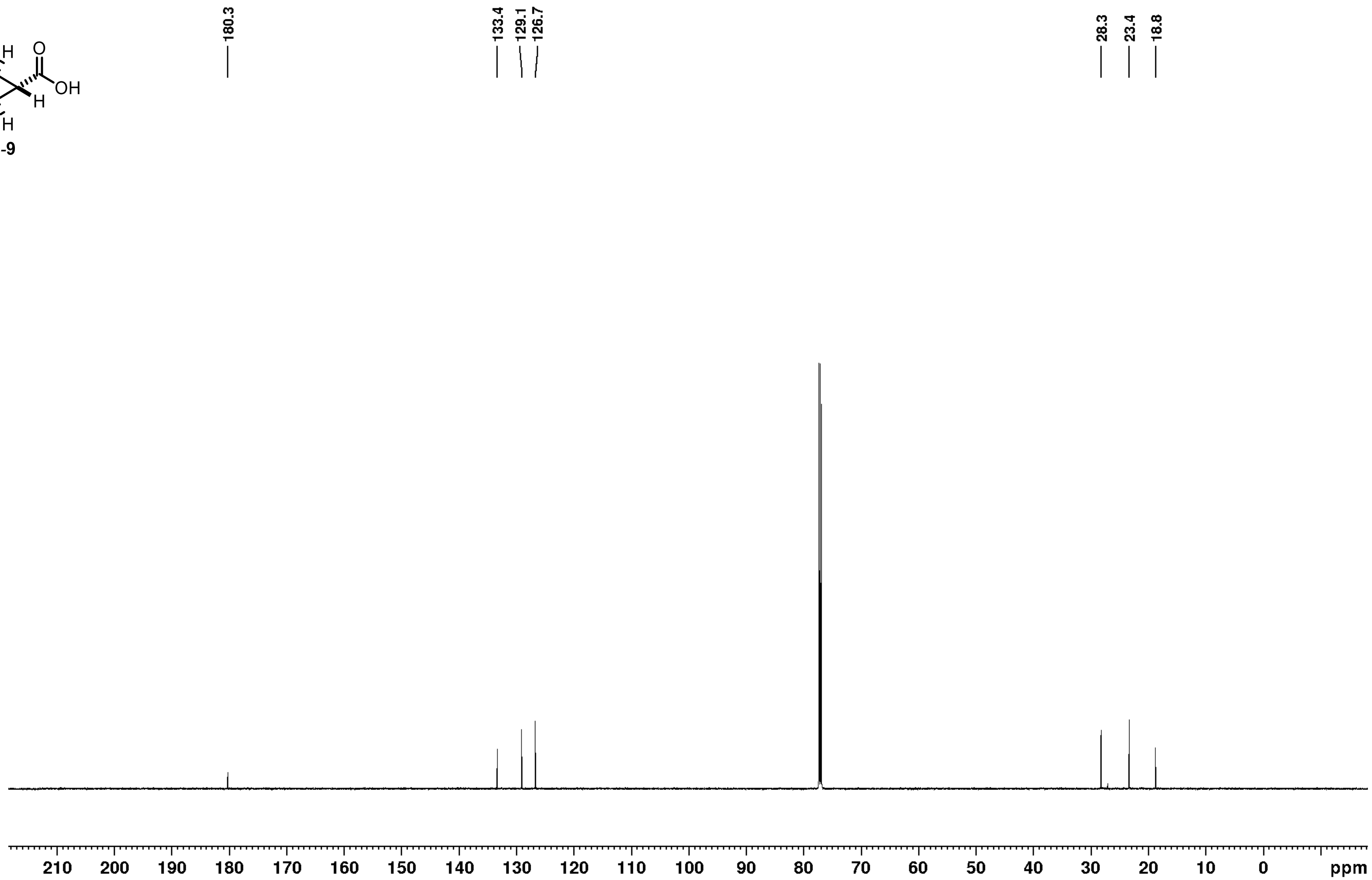

Figure S19: ^{13}C NMR spectrum of *trans*-**9**.

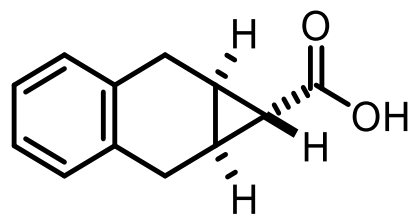

trans-**9**

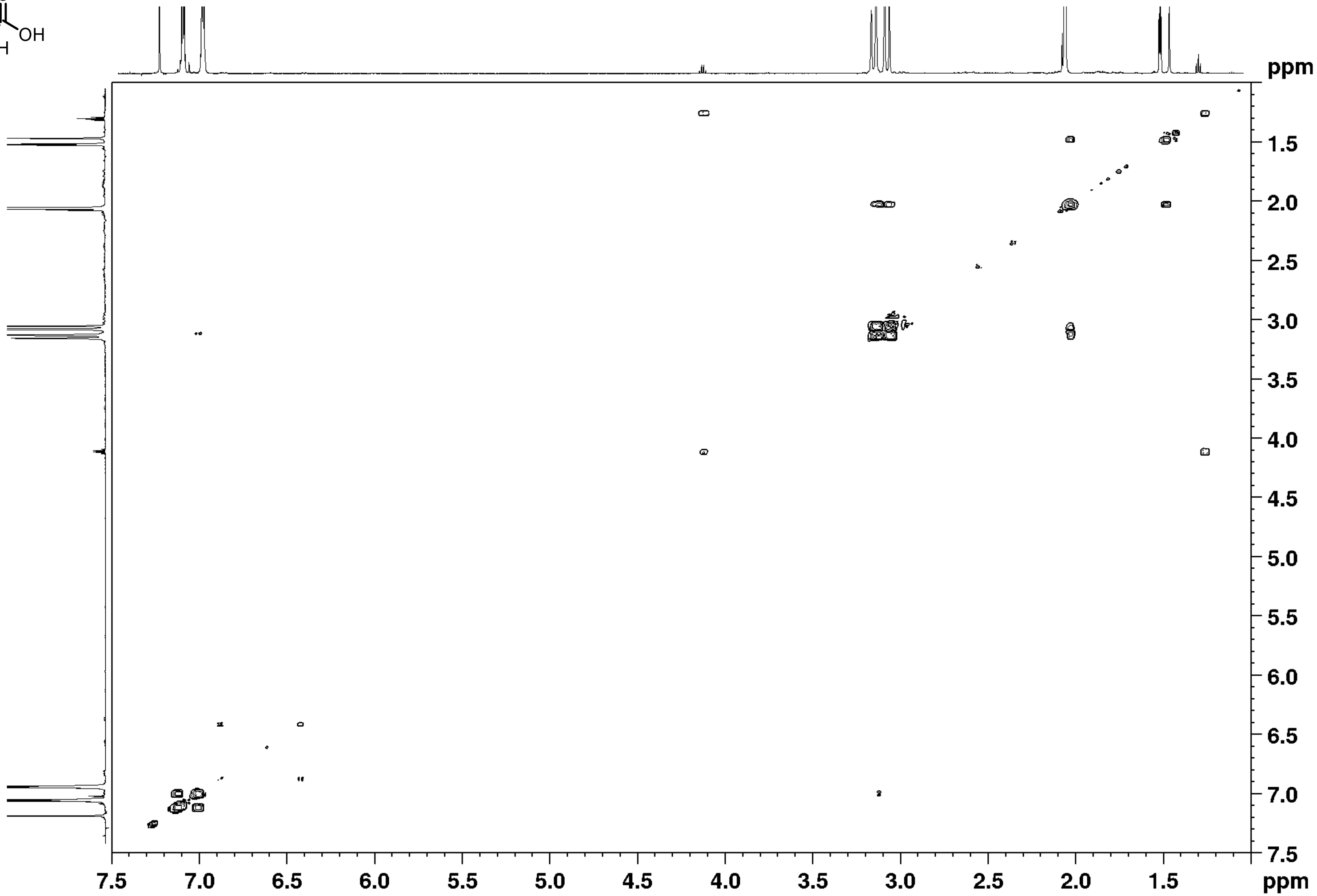

Figure S20: ^{1}H, ^{1}H COSY spectrum of *trans*-**9**.

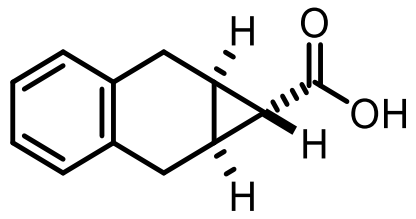

trans-**9**

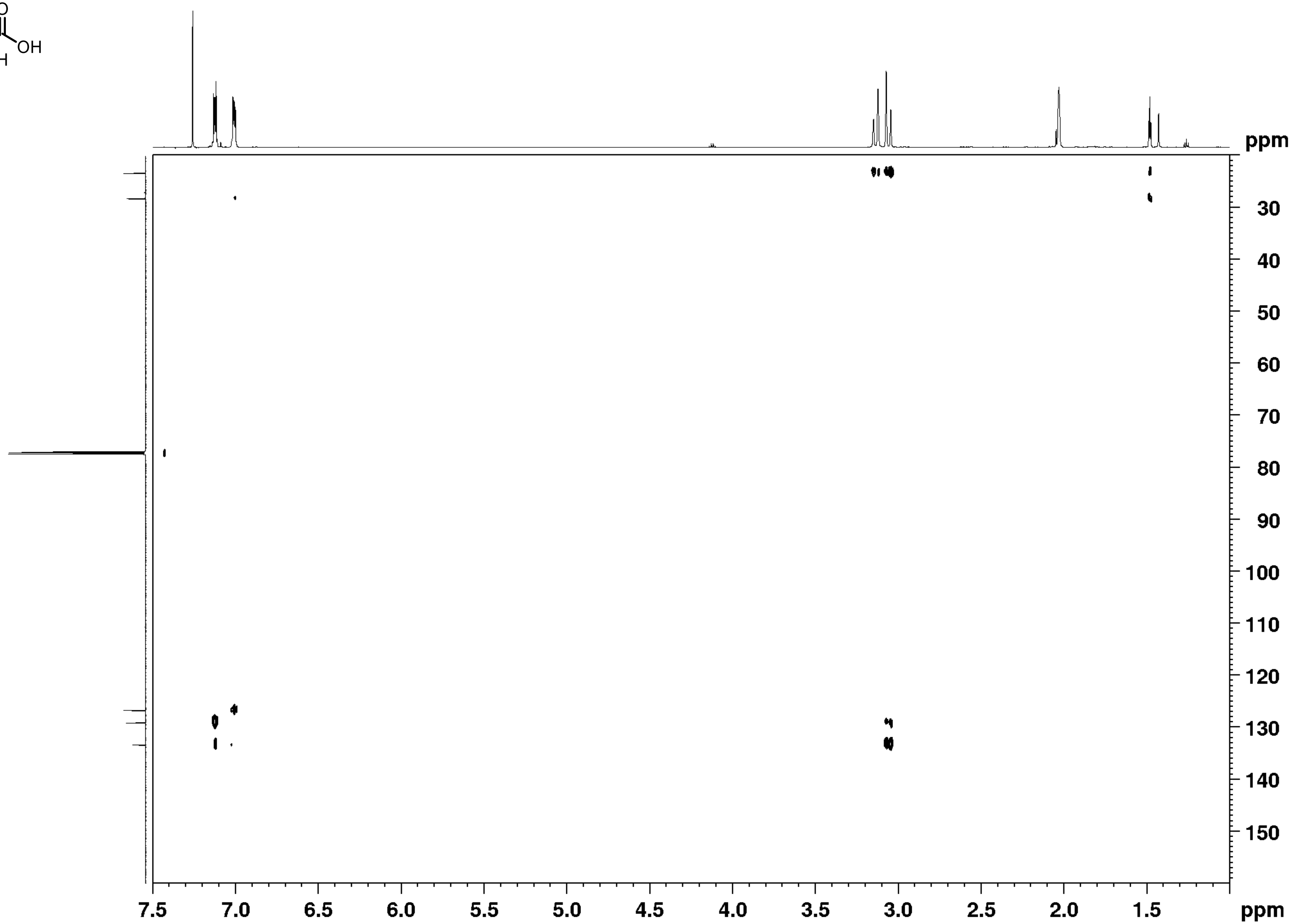

Figure S21: ^{1}H, ^{13}C HMBC spectrum of *trans*-**9**.

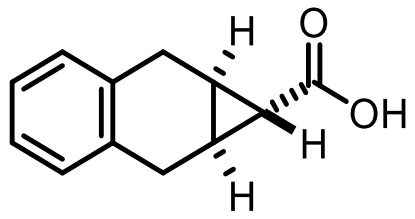

trans-**9**

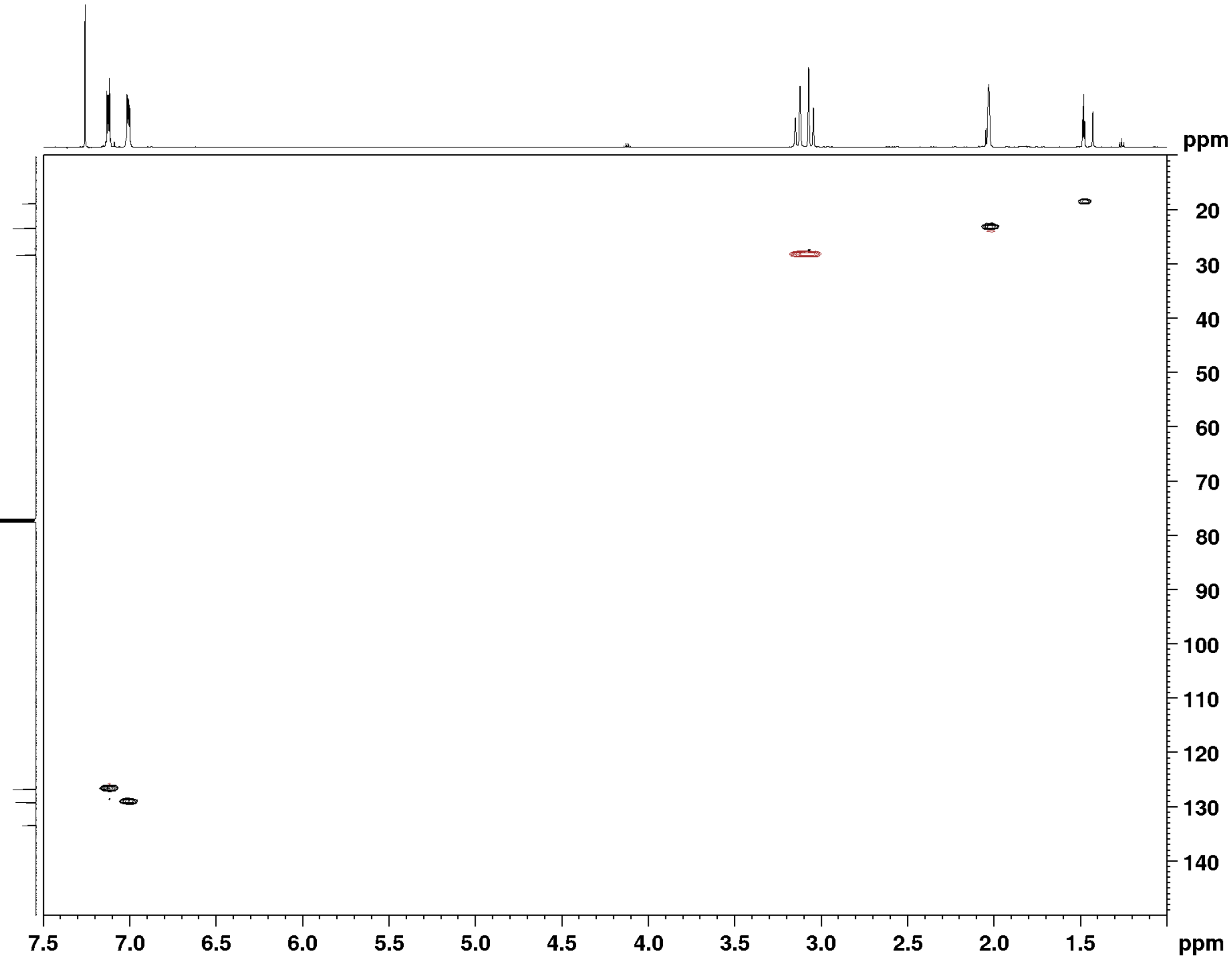

Figure S22: ^{1}H, ^{13}C HSQC spectrum of *trans*-**9**.

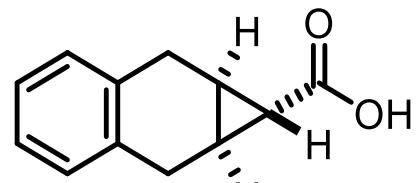

trans-9

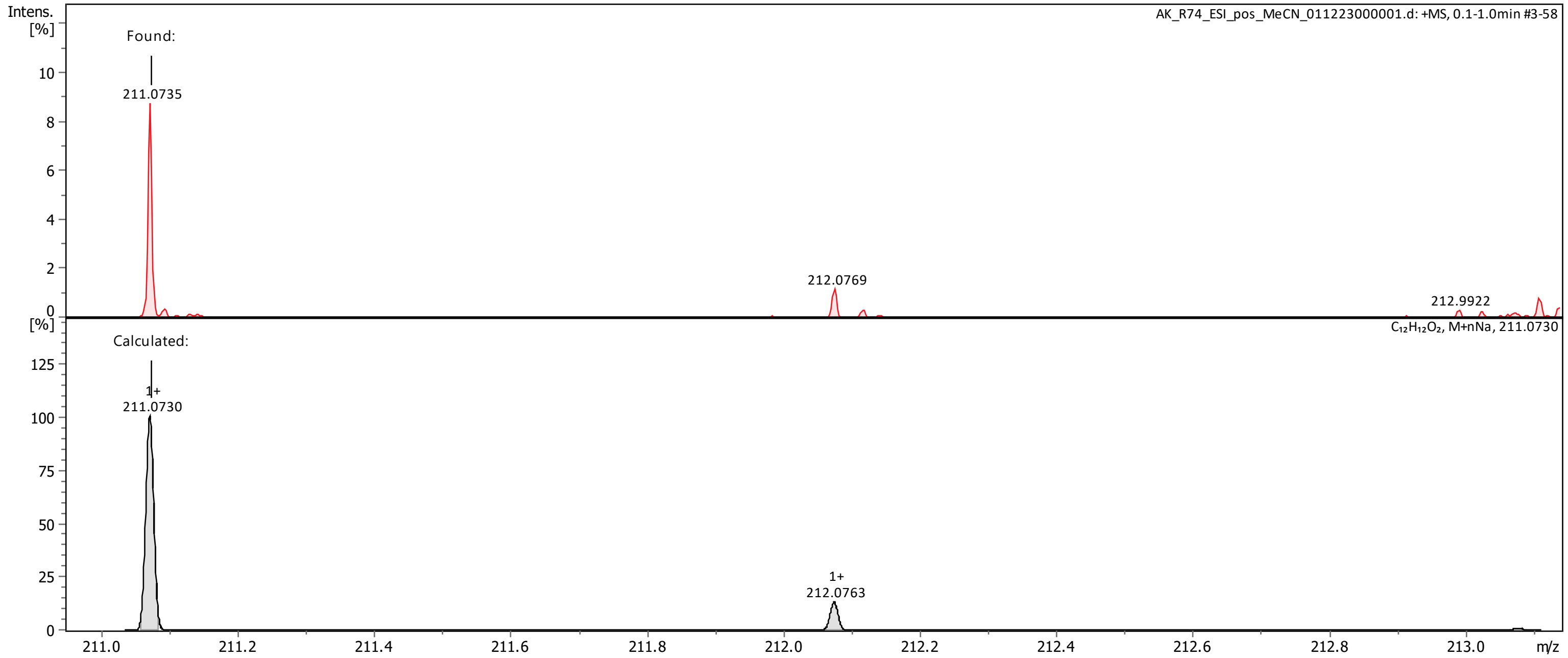

Figure S23: HRMS spectrum of *trans*-**9**.

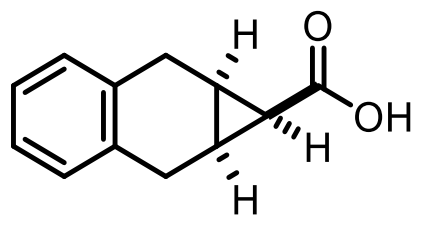

cis-**9**

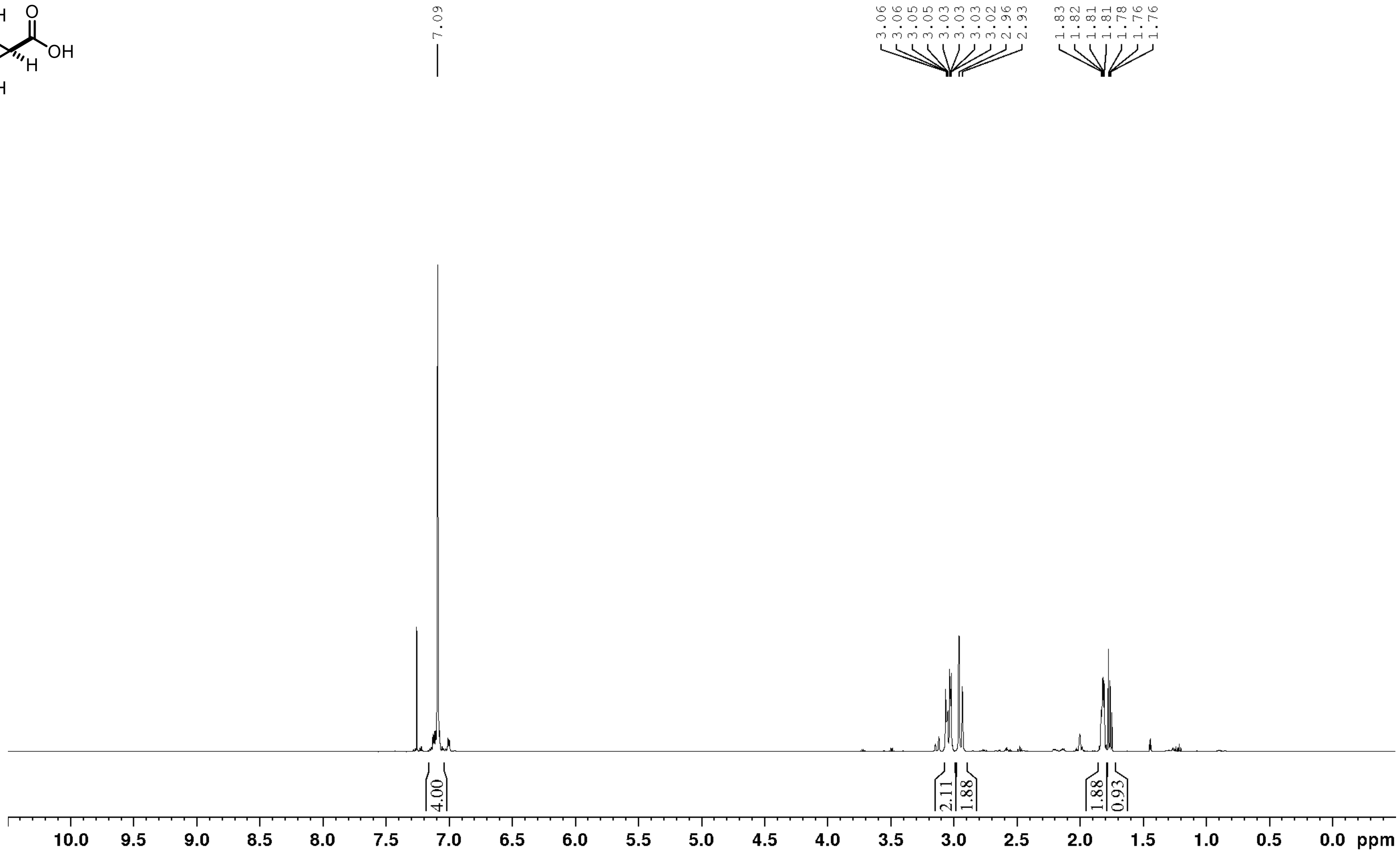

Figure S24: ^{1}H NMR spectrum of *cis*-**9**.

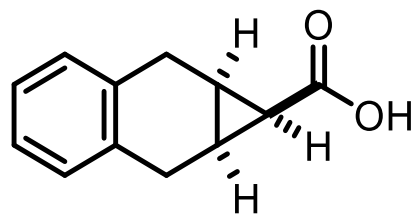

cis-**9**

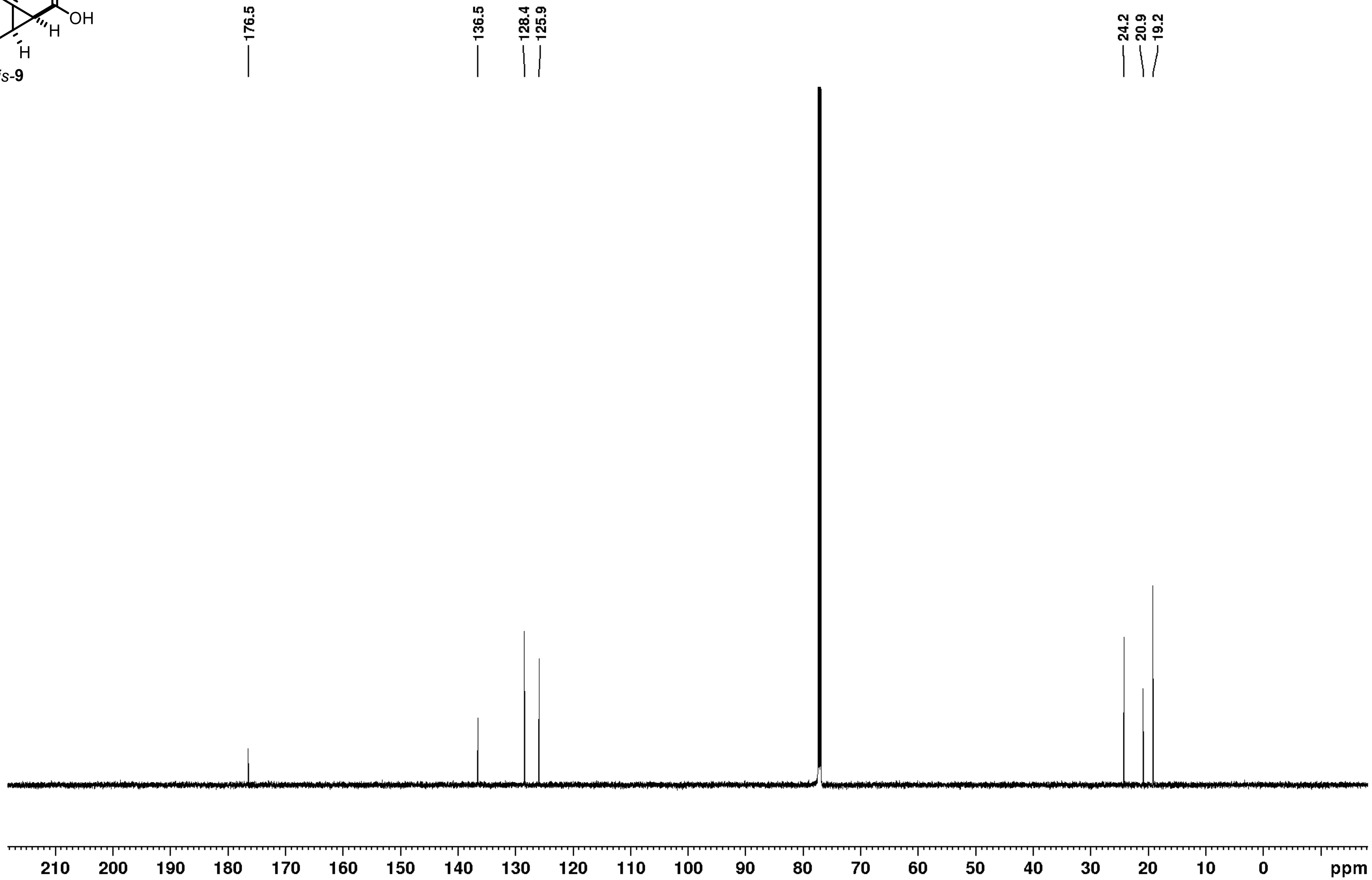

Figure S25: ^{13}C NMR spectrum of *cis*-**9**.

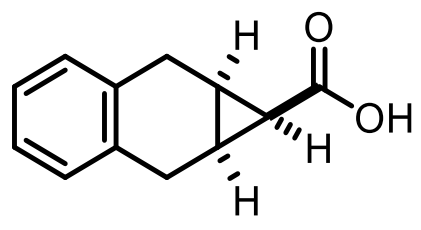

cis-**9**

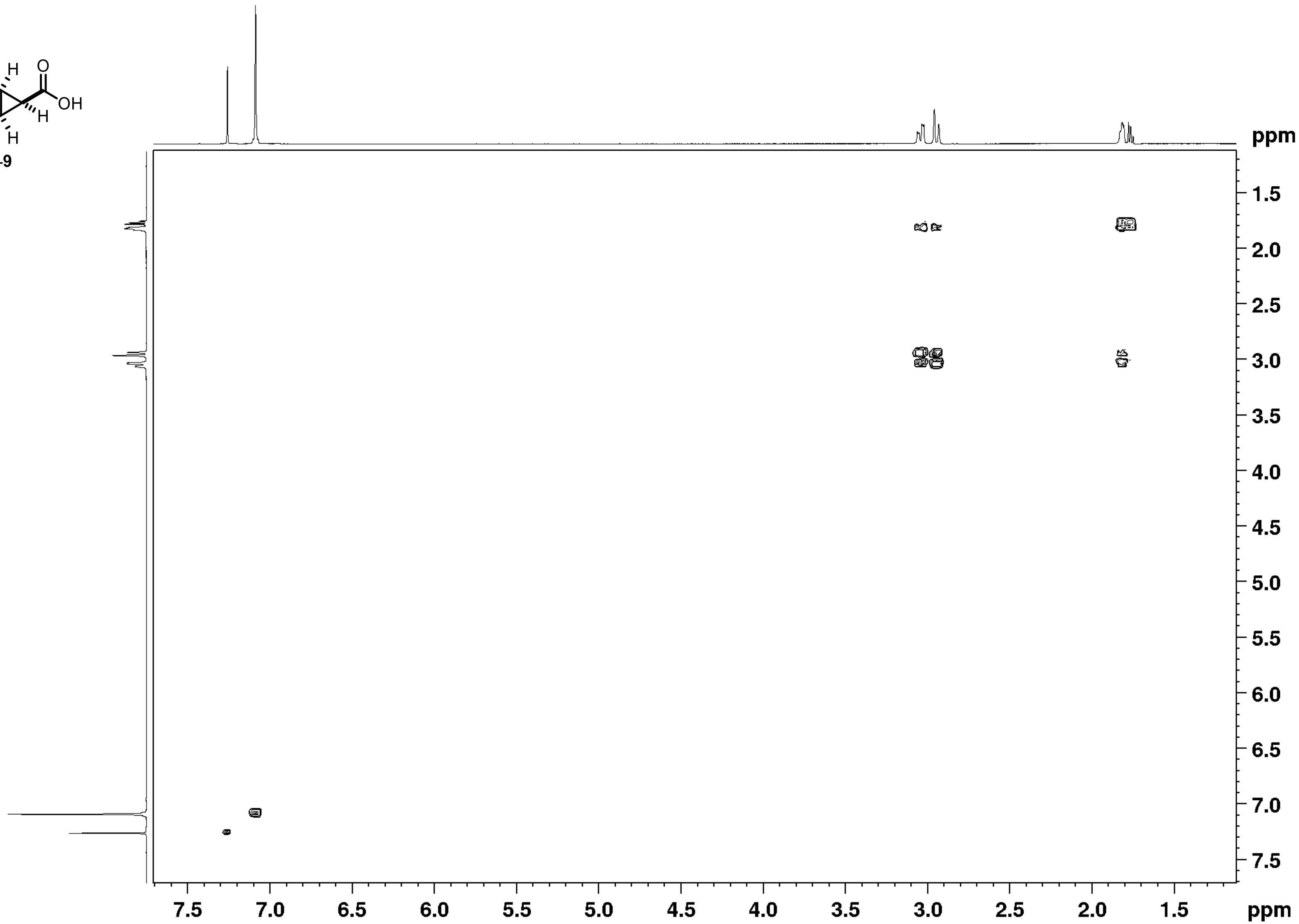

Figure S26: ^1H, ^1H COSY spectrum of *cis*-**9**.

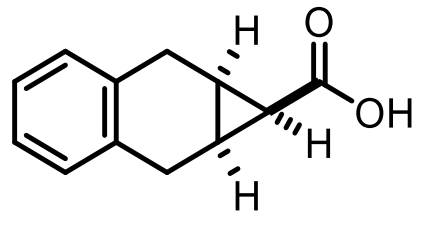

cis-**9**

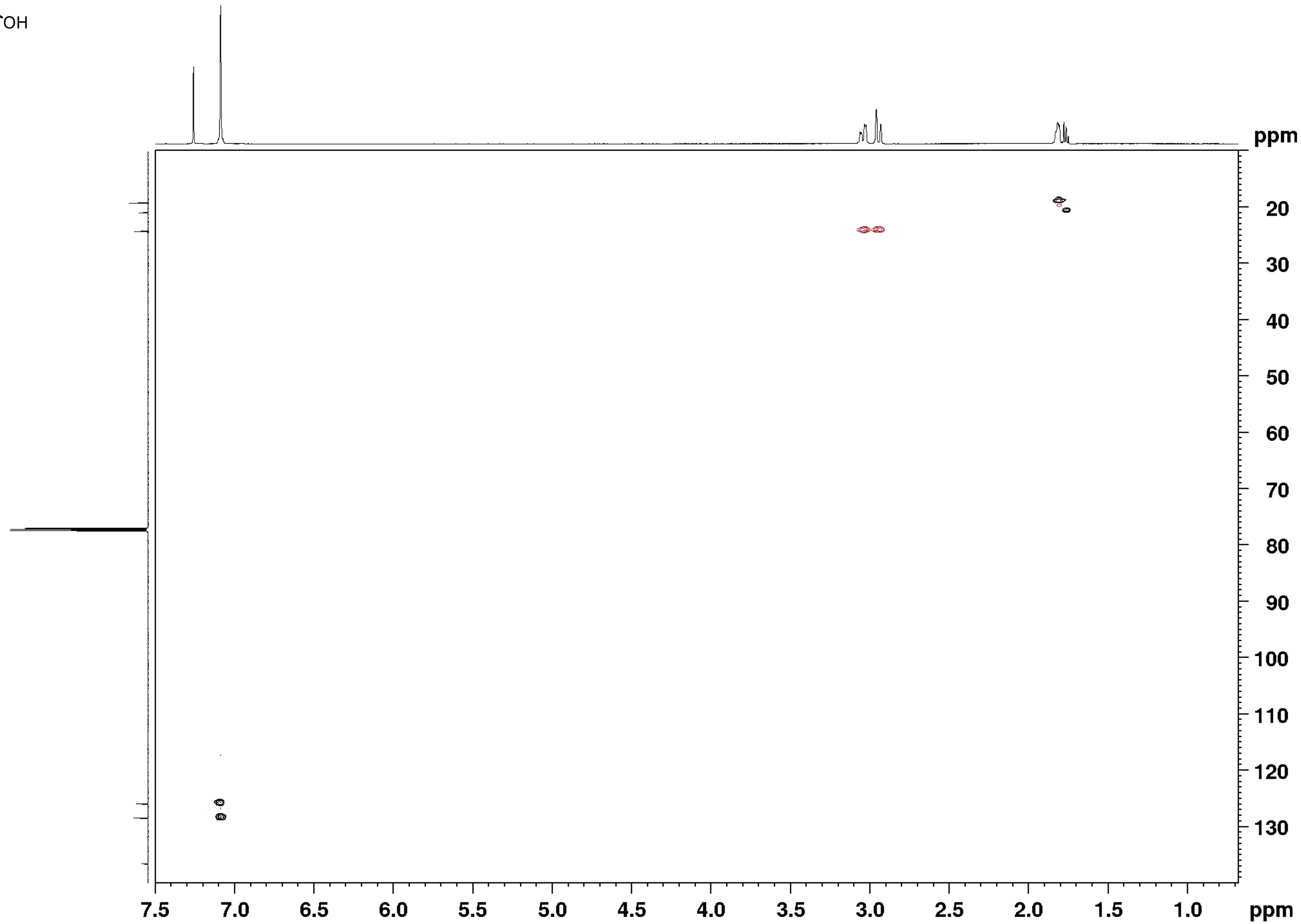

Figure S27: ^{1}H, ^{13}C HSQC spectrum of *cis*-**9**.

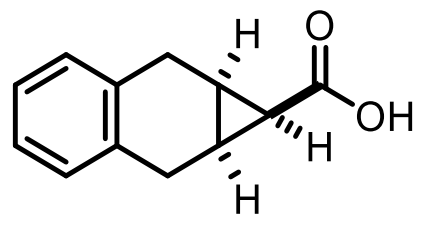

cis-**9**

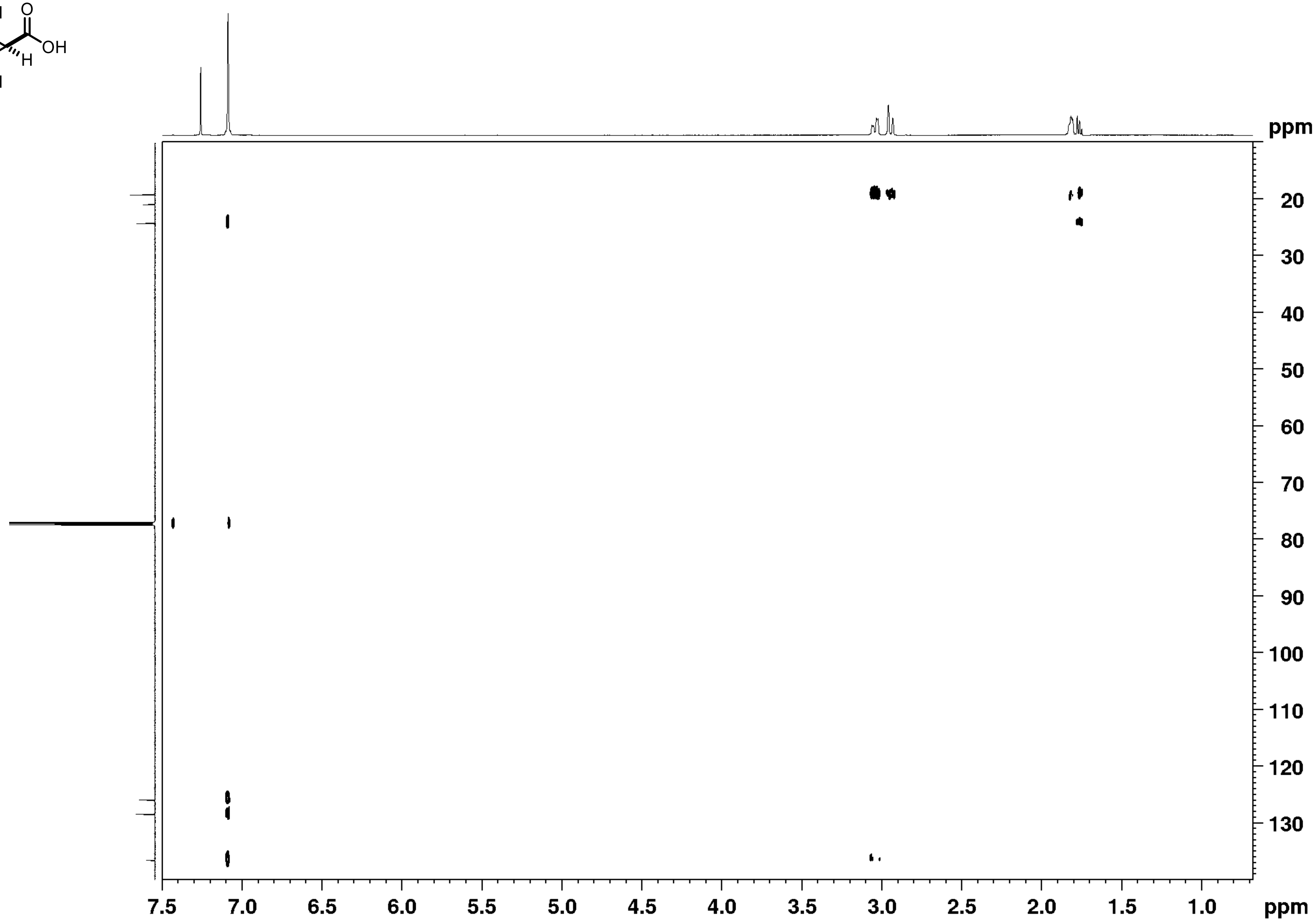

Figure S28: ^{1}H, ^{13}C HMBC spectrum of *cis*-**9**.

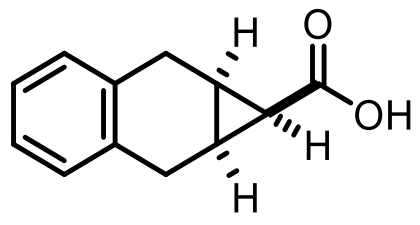

cis-**9**

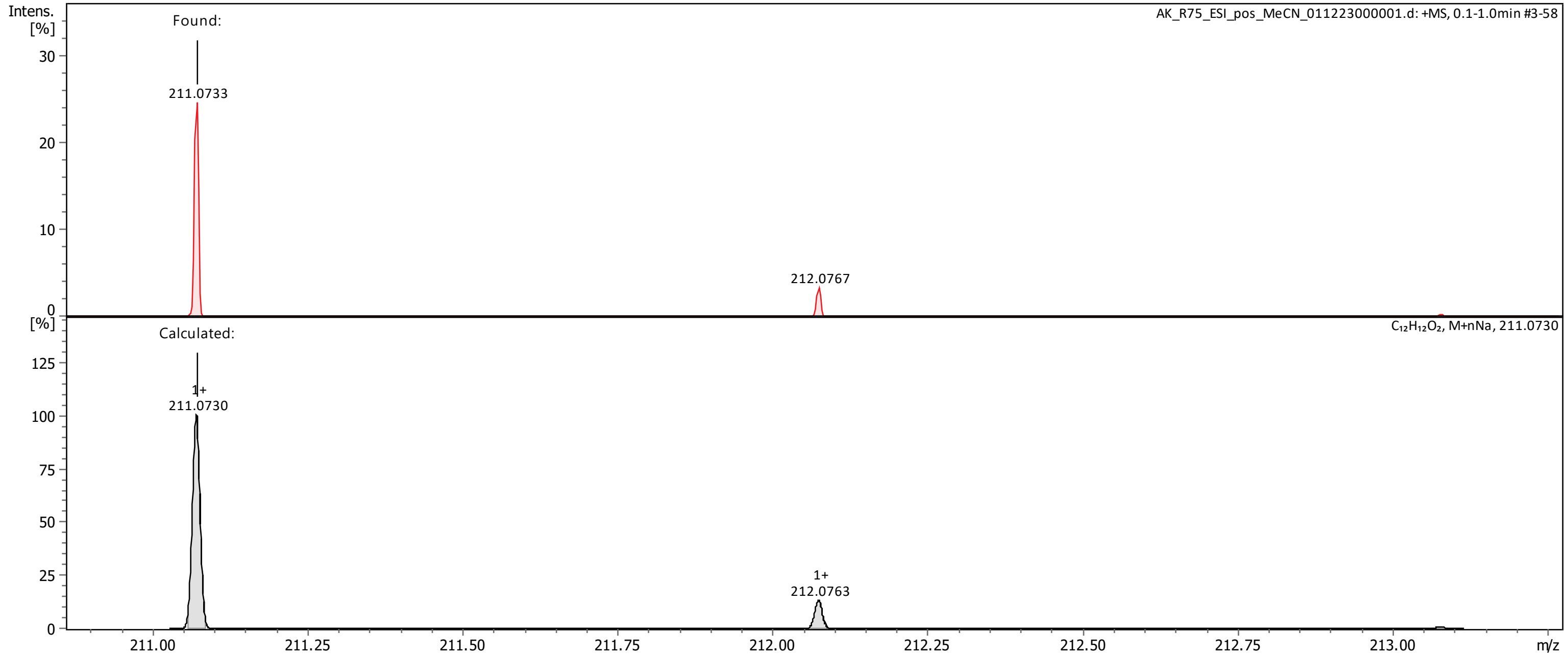

Figure S29: HRMS spectrum of *cis*-**9**.

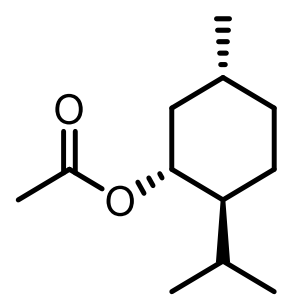

(L)-**SI3**

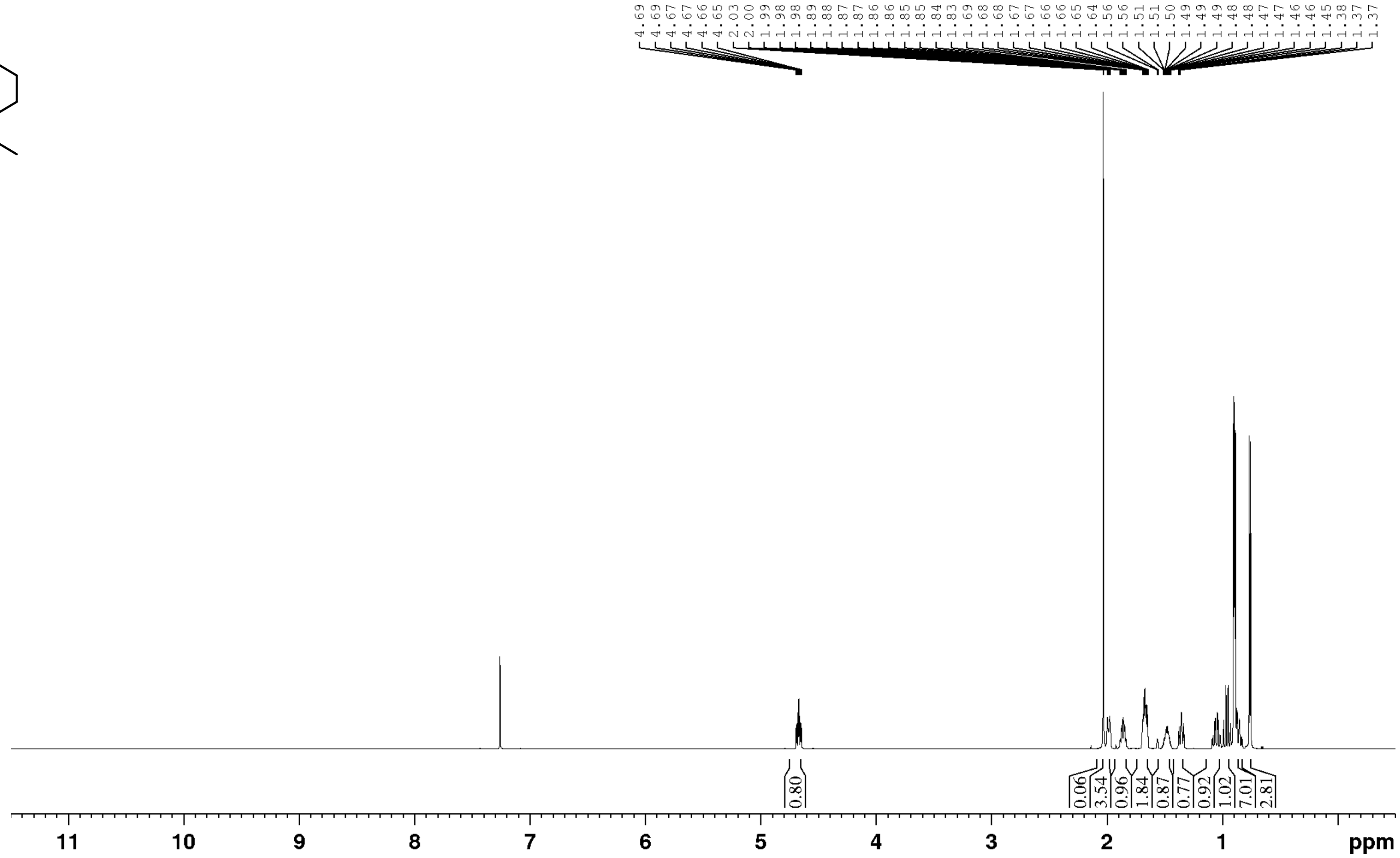

Figure S30: ^{1}H NMR spectrum of (L)-**SI3**.

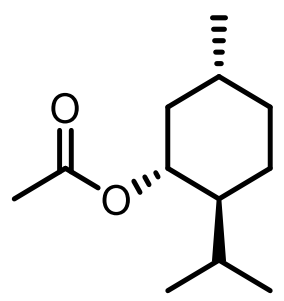

(L)-**SI3**

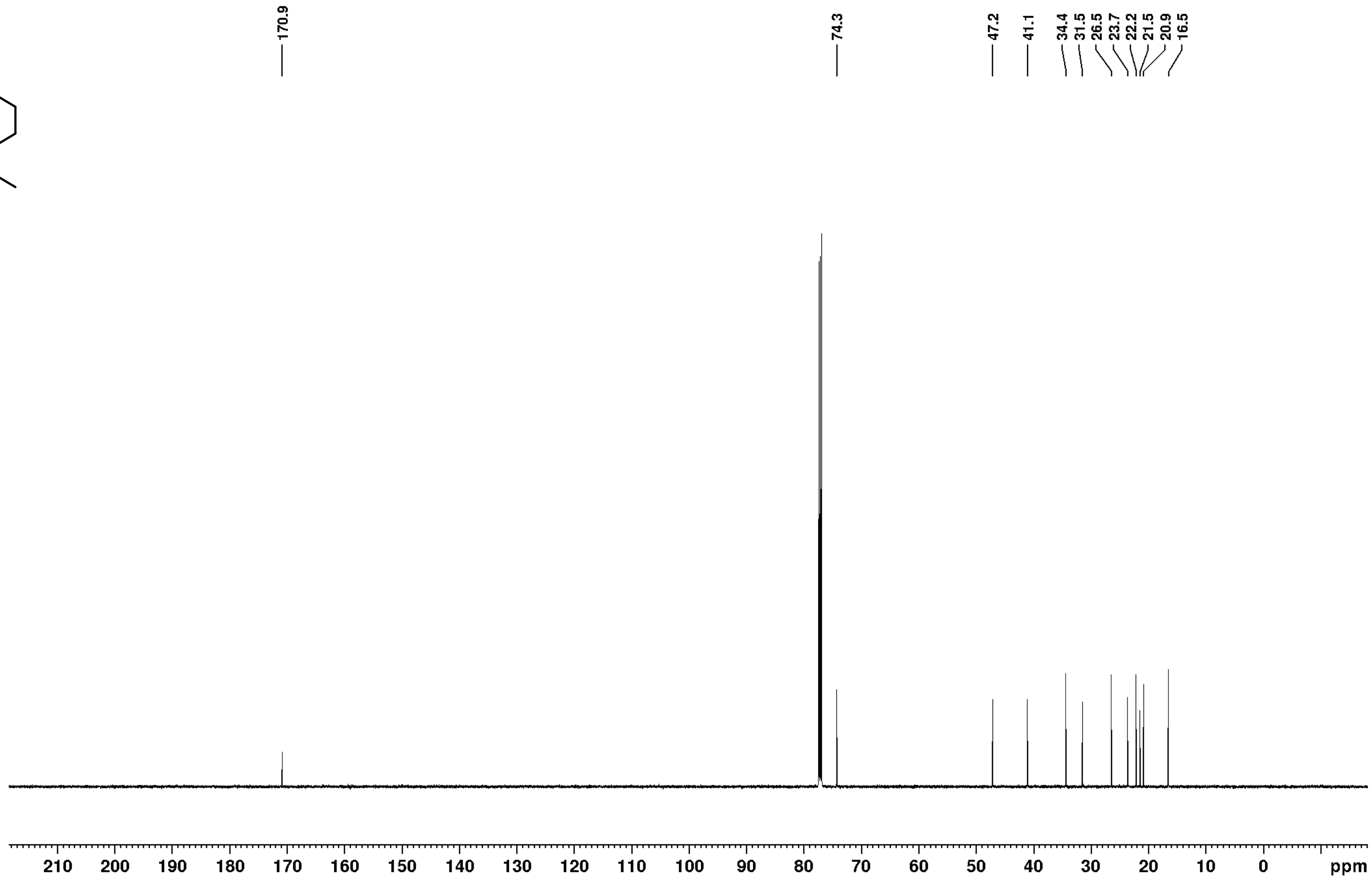

Figure S31: ^{13}C NMR spectrum of (L)-**SI3**.

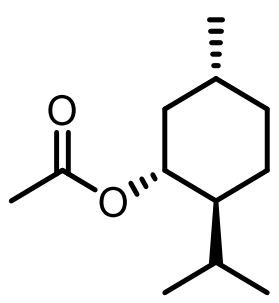

(L)-**SI3**

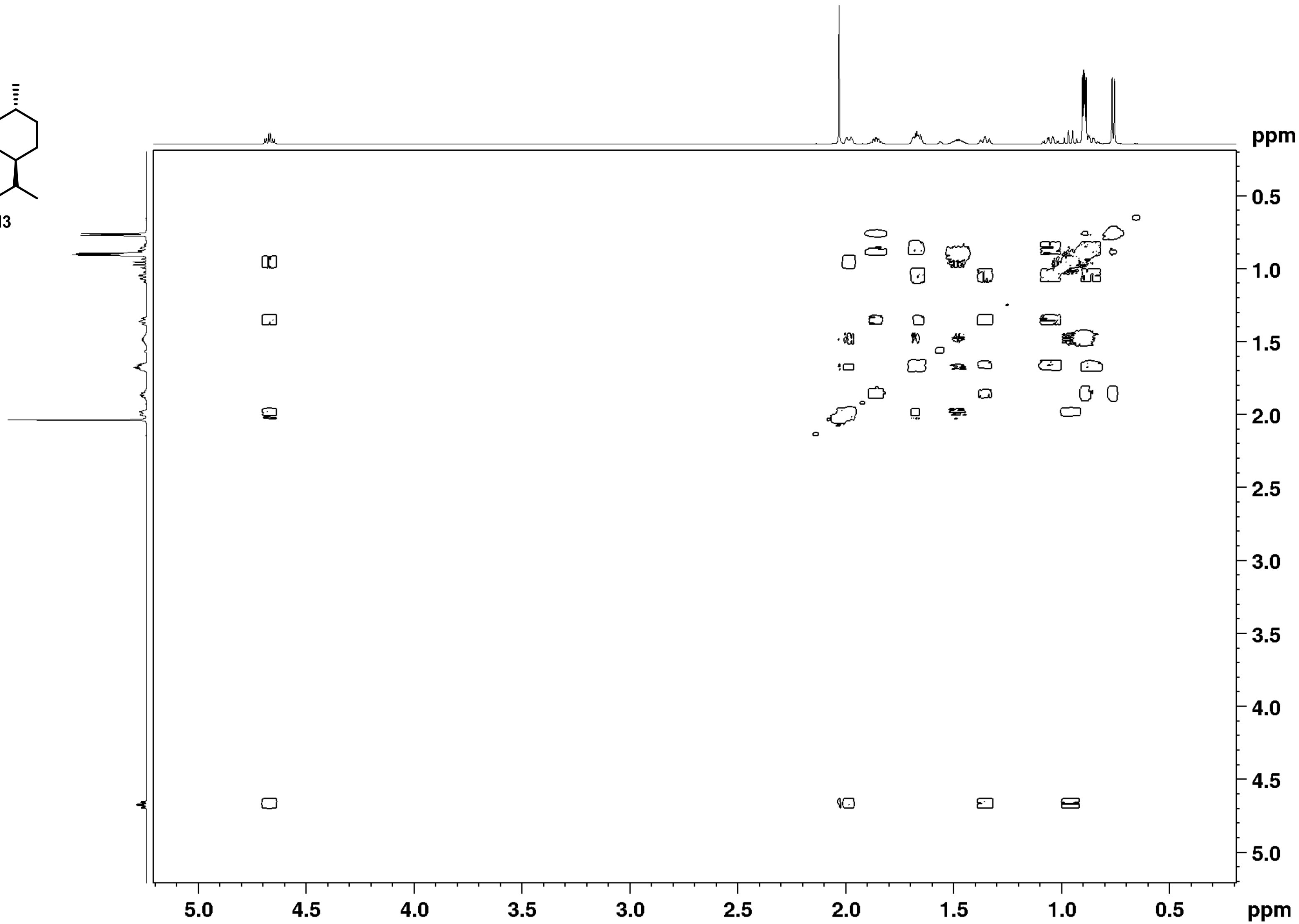

Figure S32: ^{1}H, ^{1}H COSY spectrum of (L)-**SI3**.

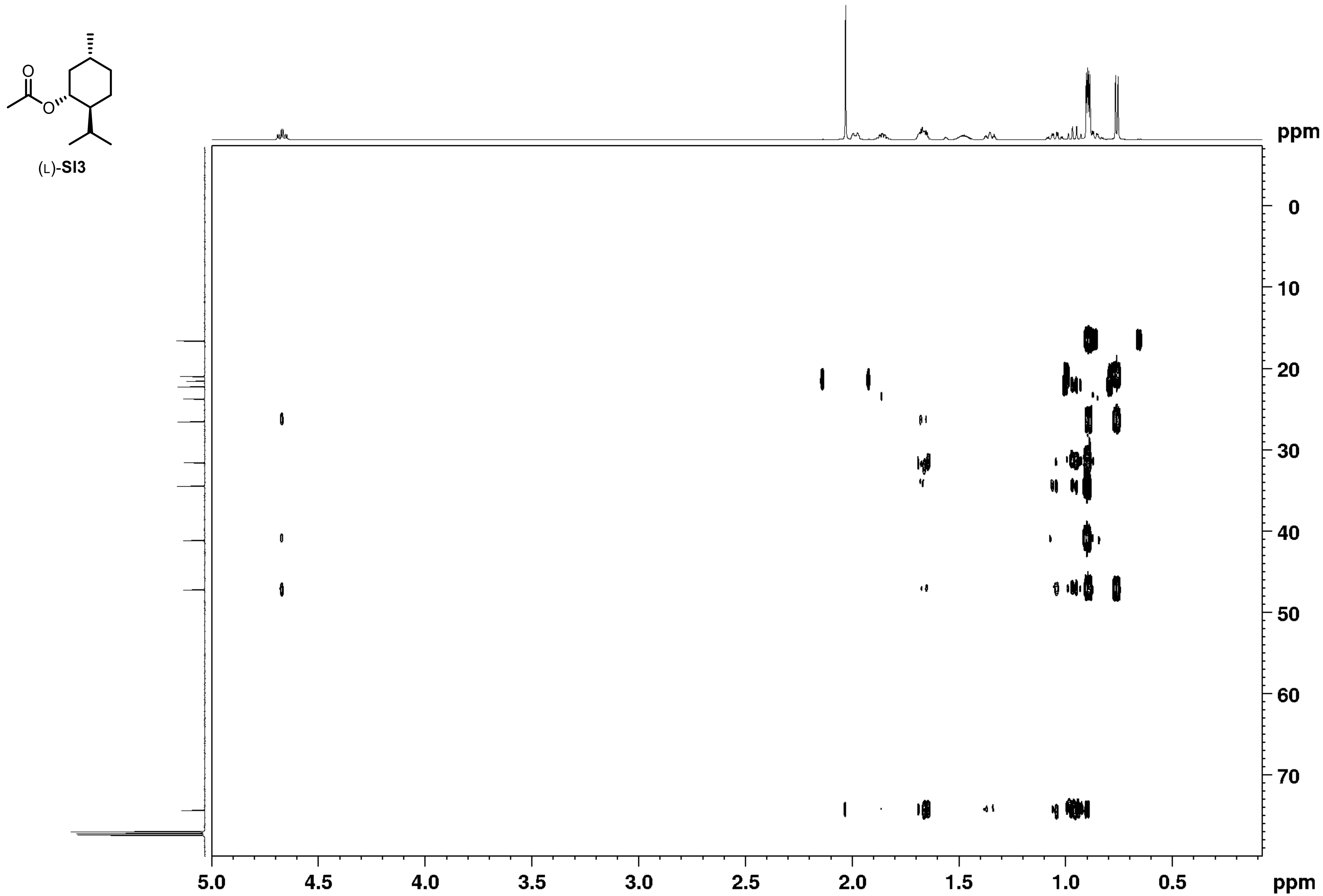

Figure S33: ^{1}H, ^{13}C HMBC spectrum of (L)-**SI3**.

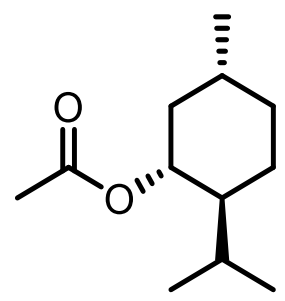

(L)-**SI3**

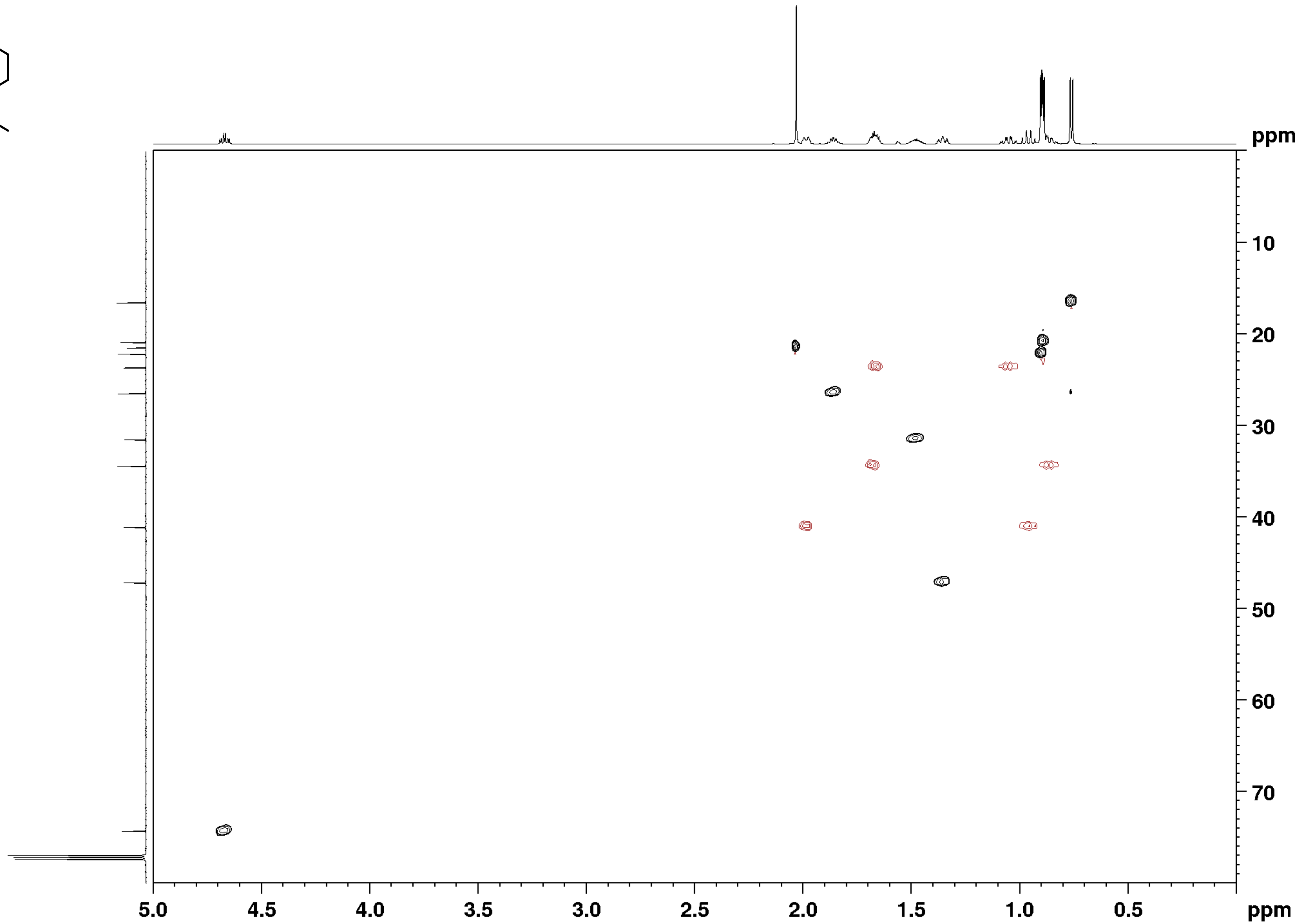

Figure S34: ^{1}H, ^{13}C HSQC spectrum of (L)-**SI3**.

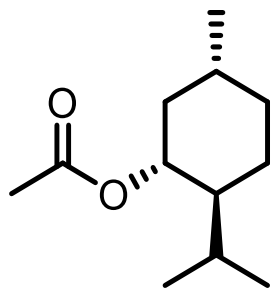

(L)-**SI3**

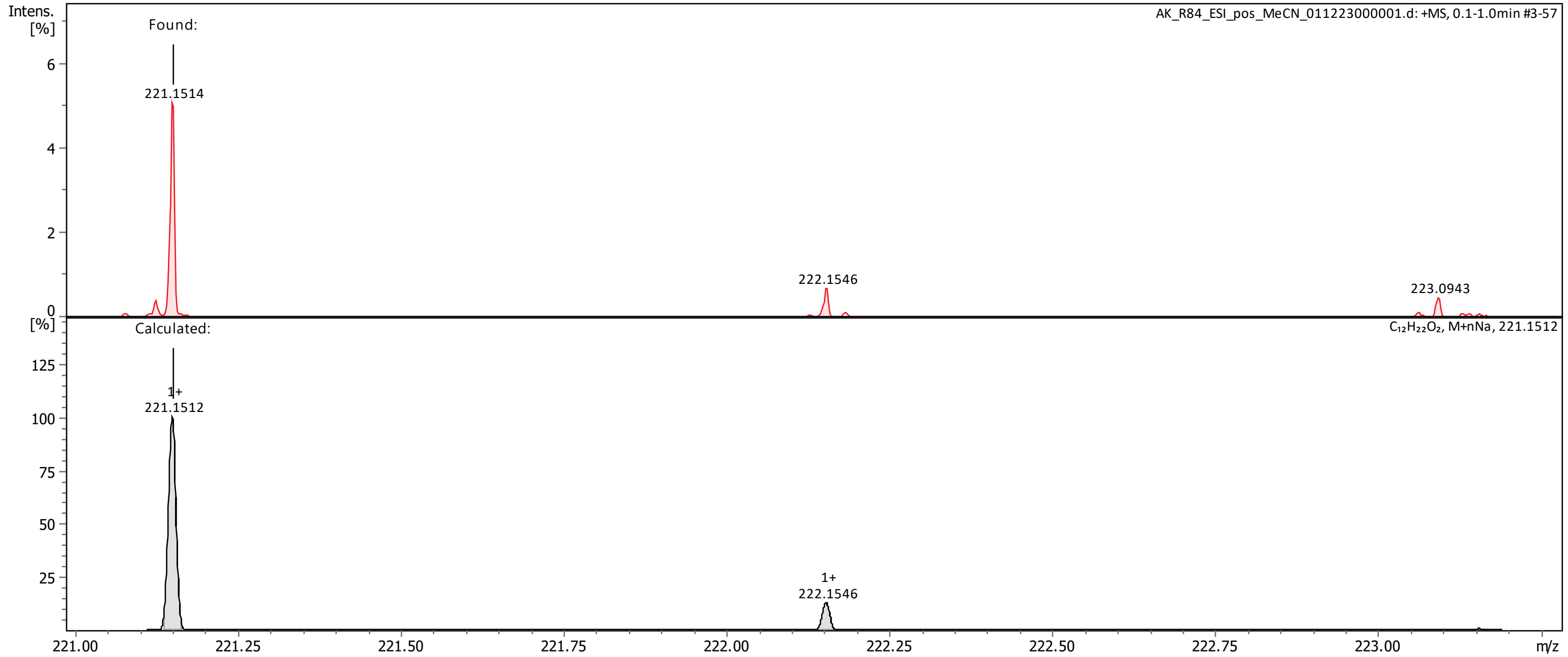

Figure S35: HRMS spectrum of (L)-**SI3**.

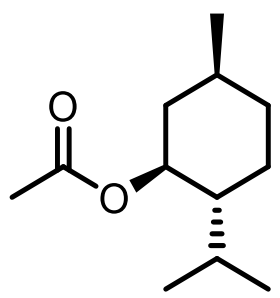

(D)-**SI3**

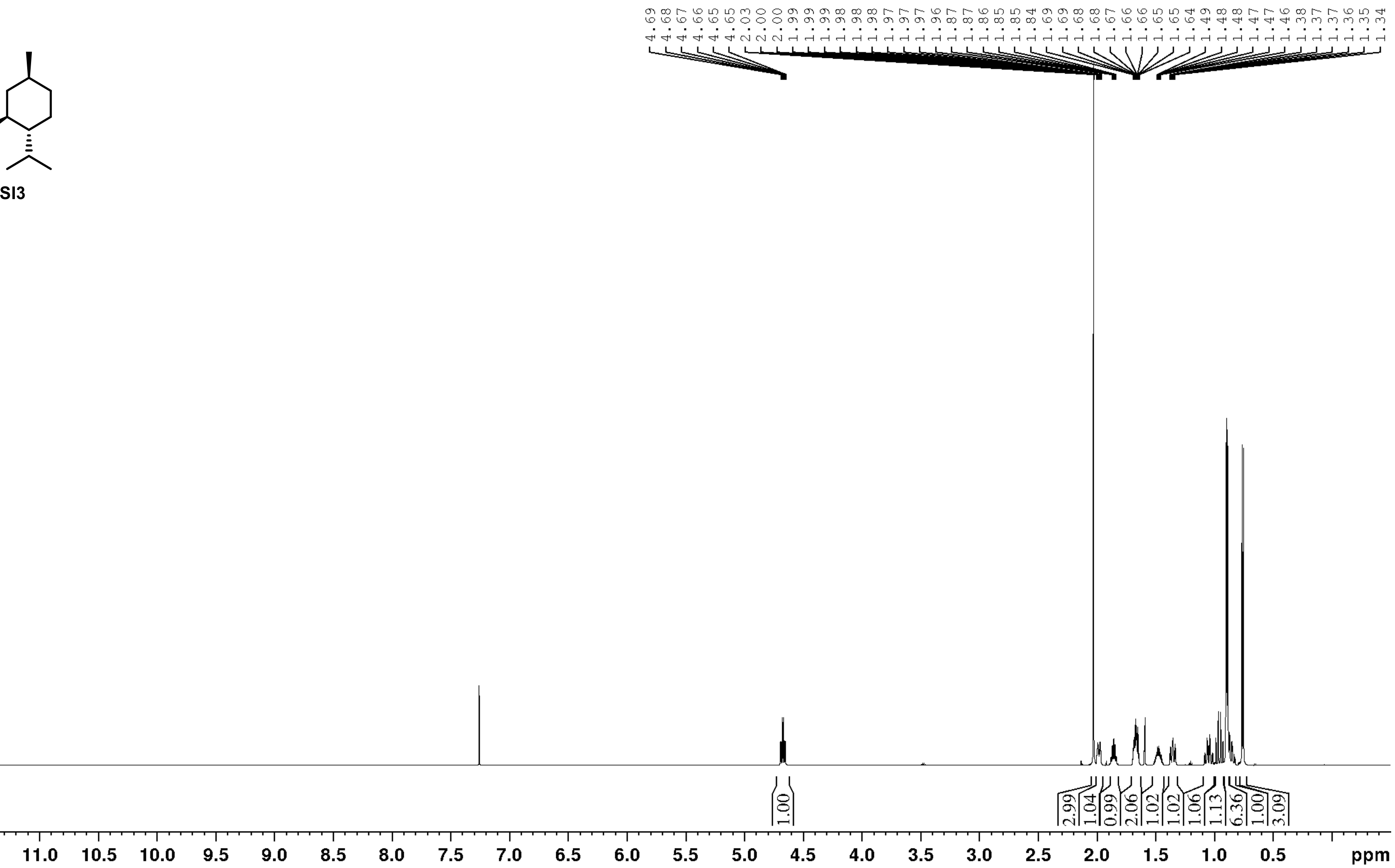

Figure S36: ^{1}H NMR spectrum, of (D)-**SI3**.

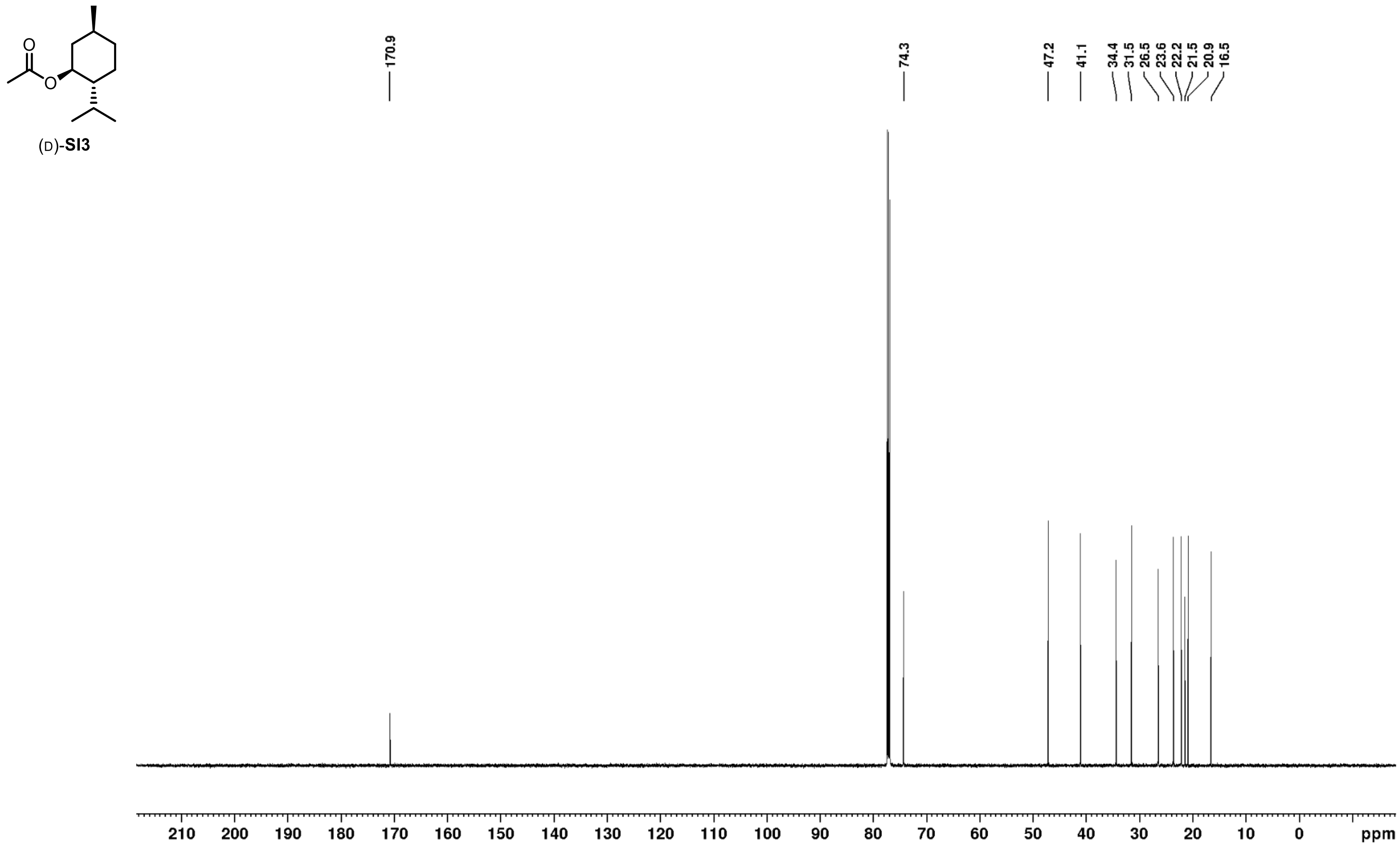

Figure S1: ^{13}C NMR spectrum of (D)-**SI3**.

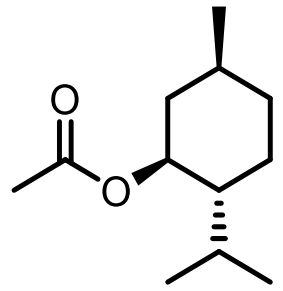

(D)-**SI3**

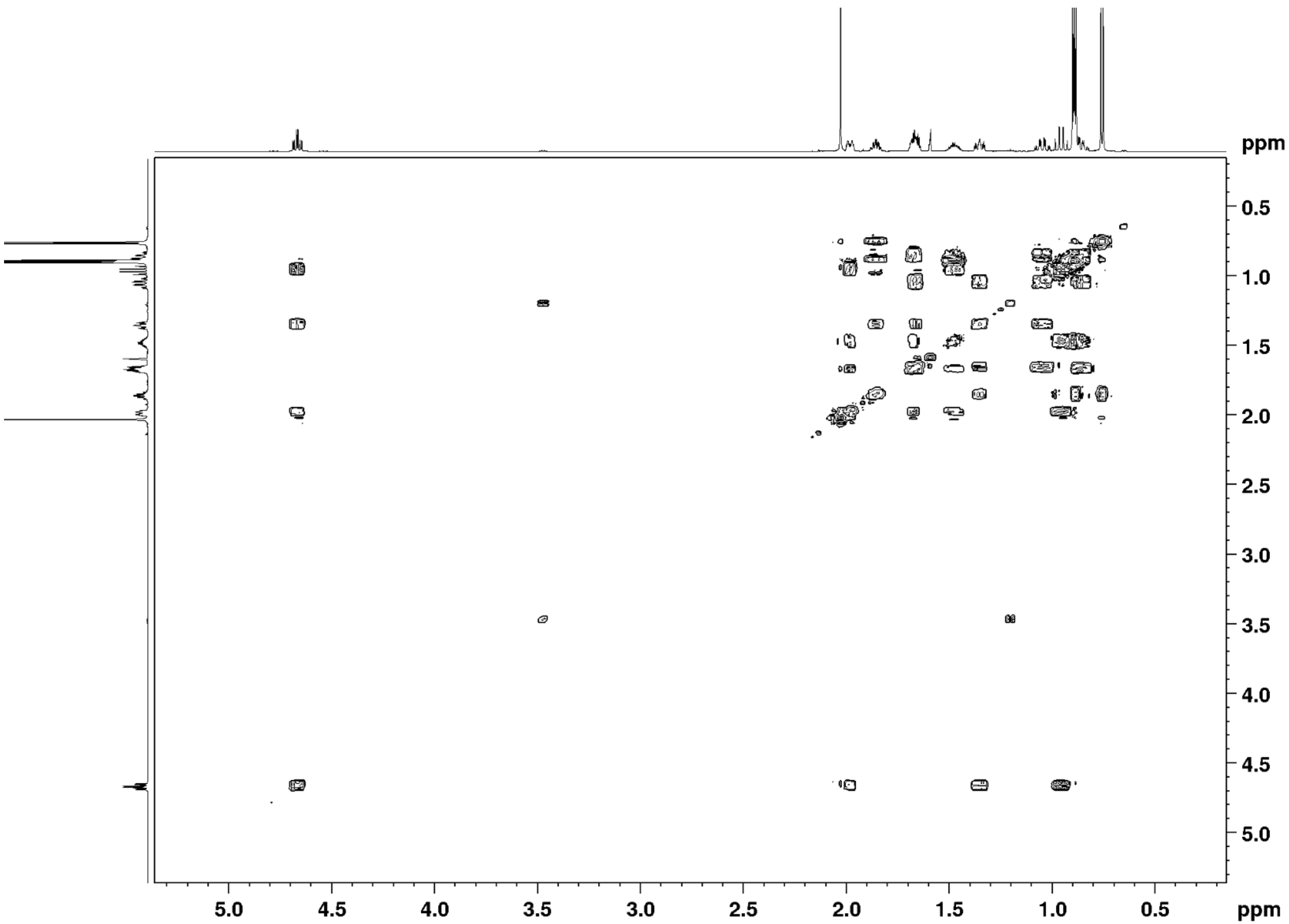

Figure S38: ^{1}H, ^{1}H COSY spectrum of (D)-**SI3**.

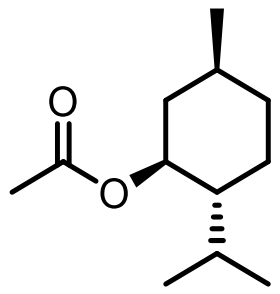

(D)-**SI3**

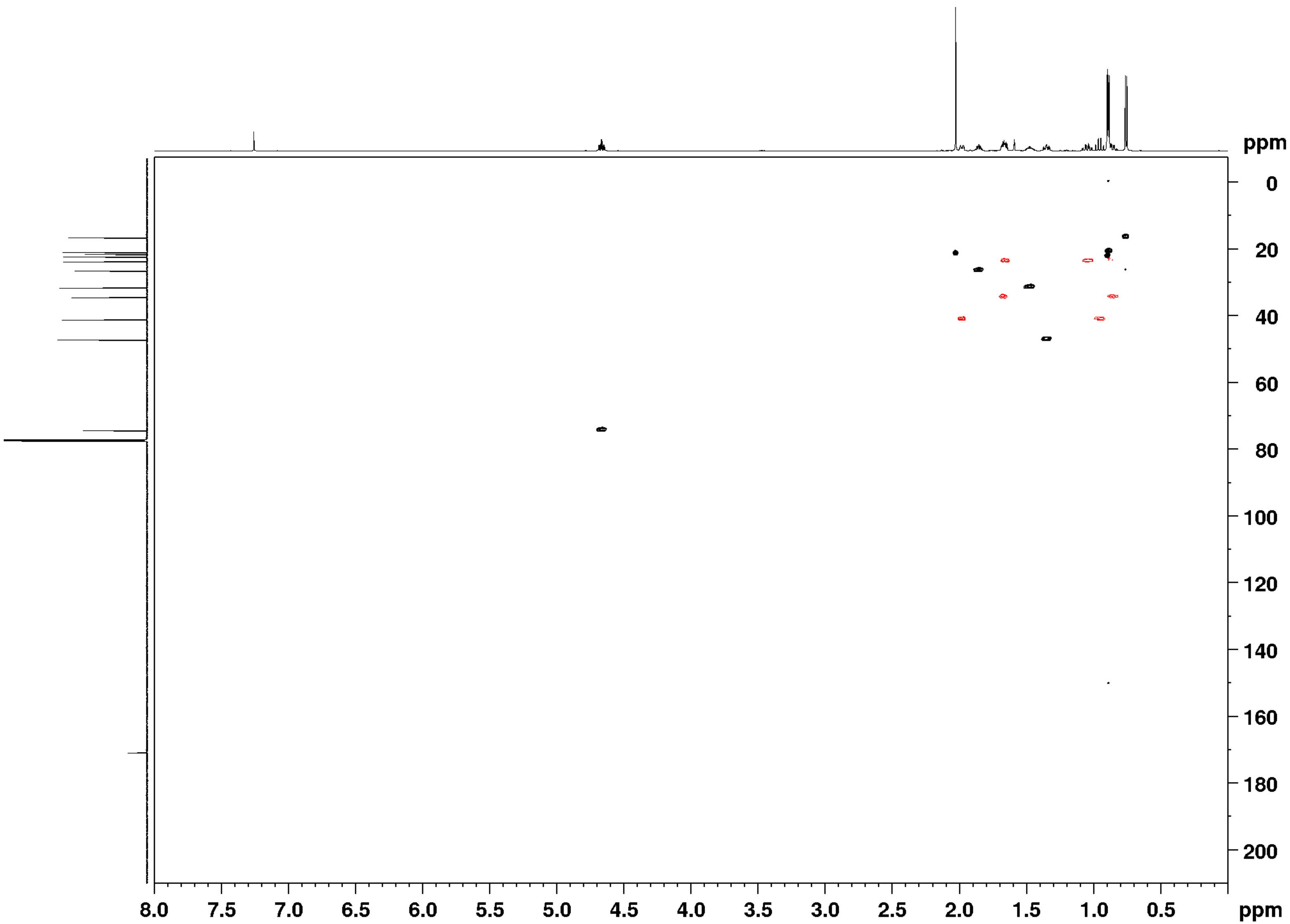

Figure S39: ^{1}H, ^{13}C HSQC spectrum of (D)-**SI3**.

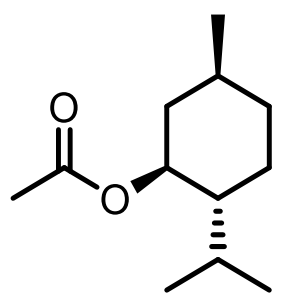

(D)-**SI3**

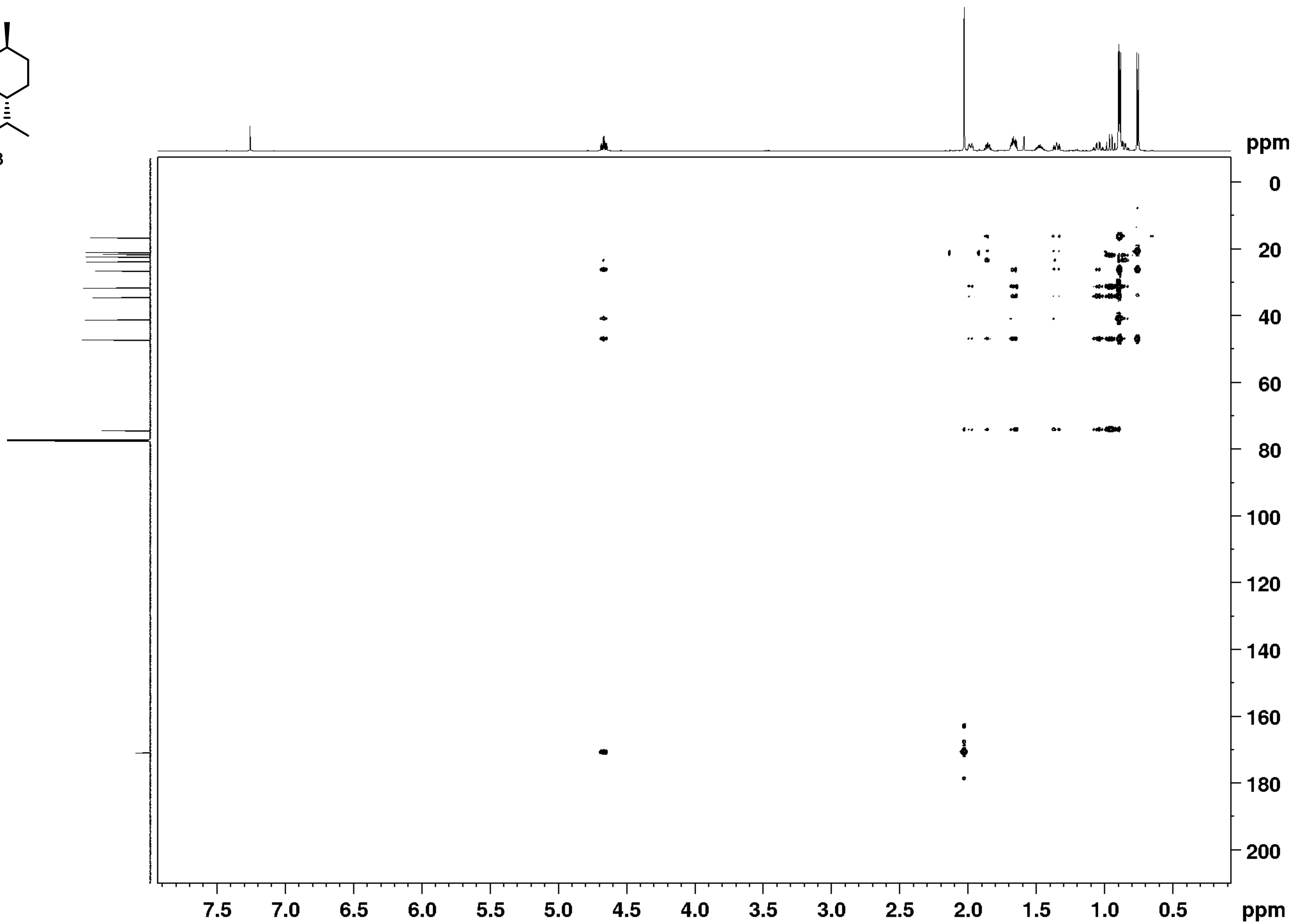

Figure S40: ^{1}H, 13C HMBC spectrum of (D)-**SI3**.

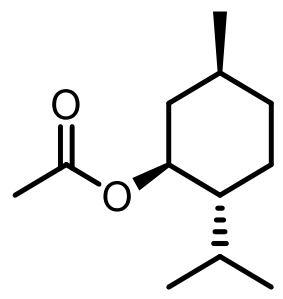

(D)-**SI3**

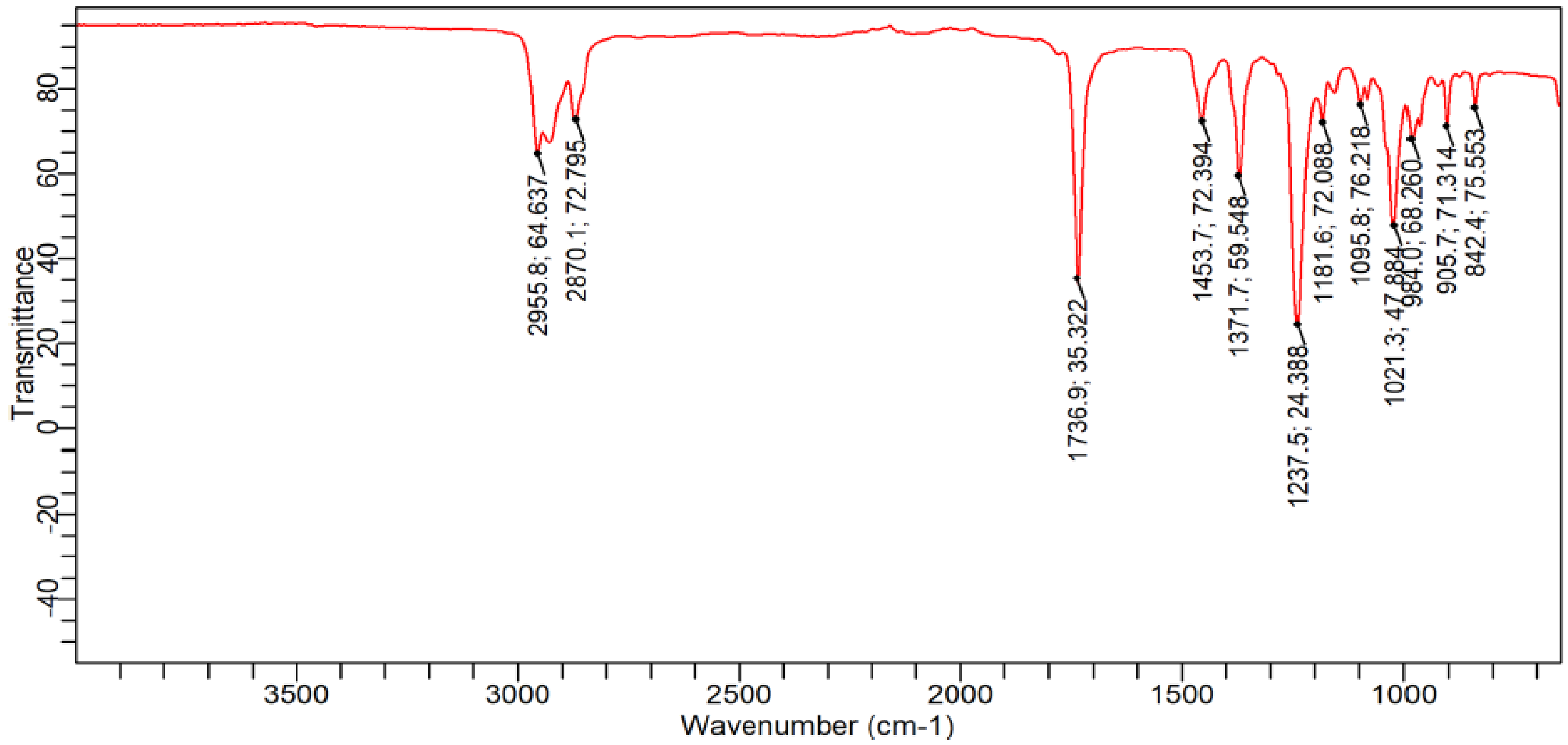

Figure S41: IR spectrum of (D)-**SI3**.

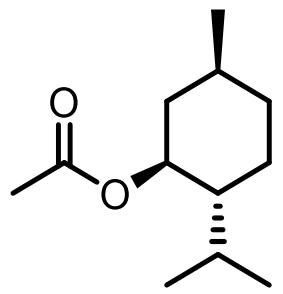

(D)-**SI3**

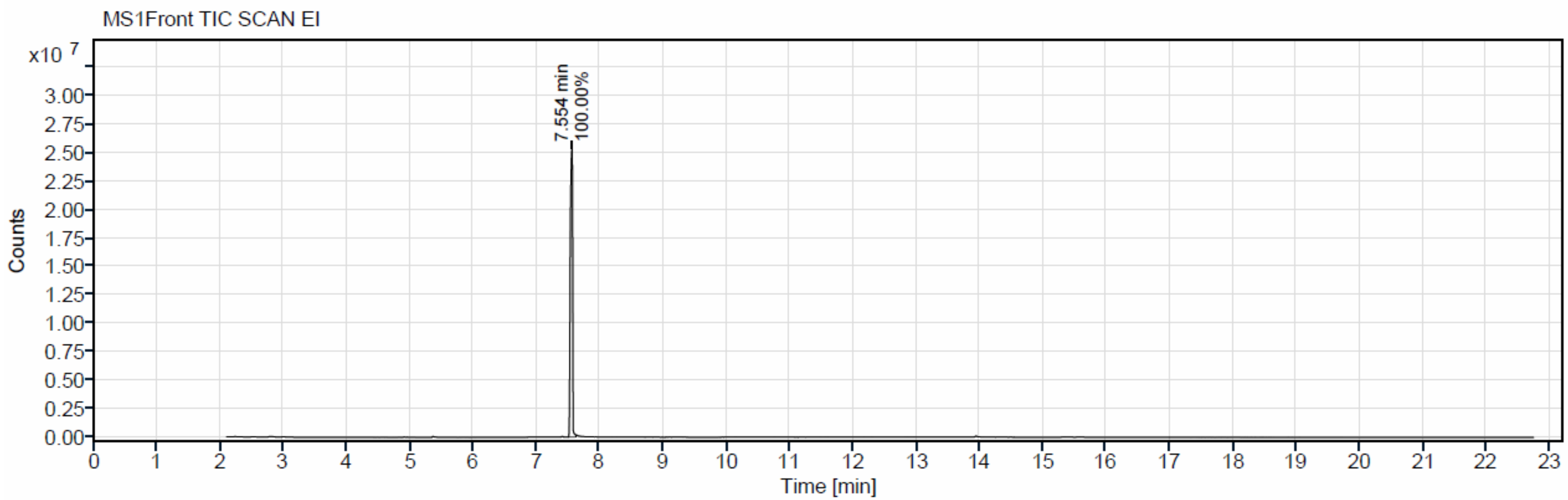

Signal: MS1Front TIC SCAN EI

RT	m/z	Spectral purity (%)	Peak area	Area %
7.554			63950000	100.00

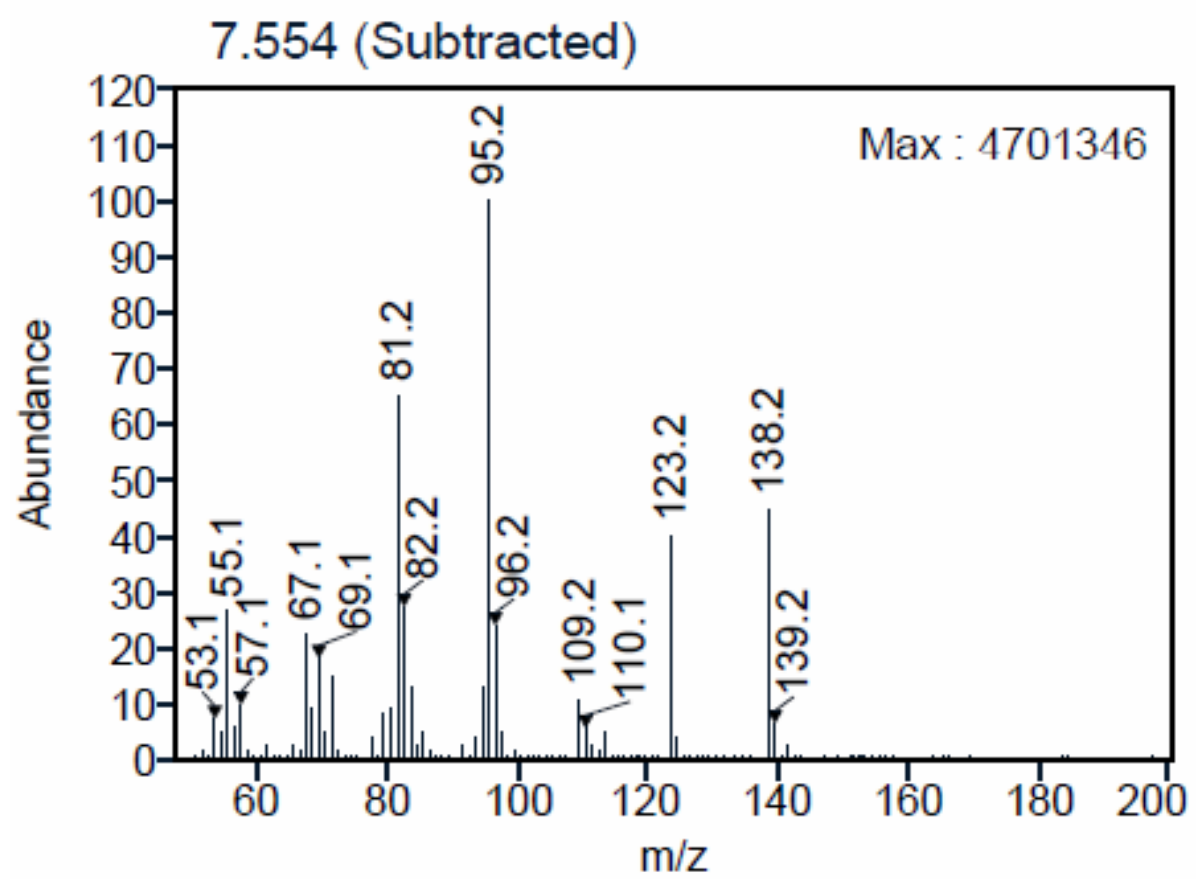

Figure S42: GC-MS TIC Scan and GC-MS spectrum of (D)-**SI3**.

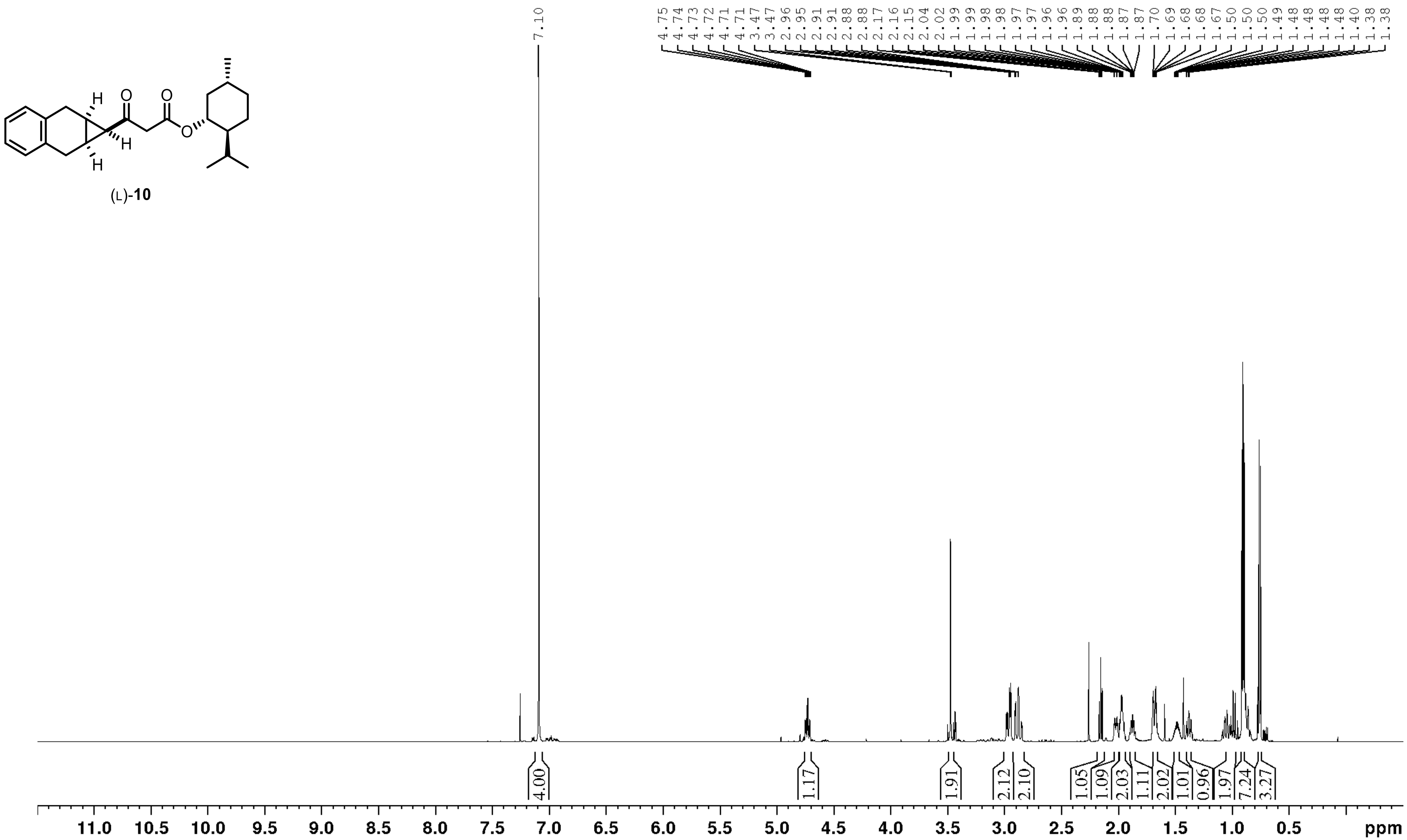

Figure S43: ^{1}H NMR spectrum of (L)-**10**.

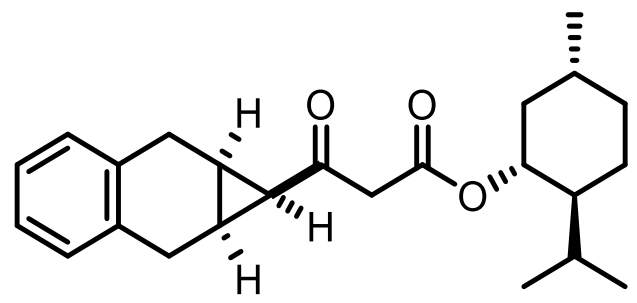

(L)-**10**

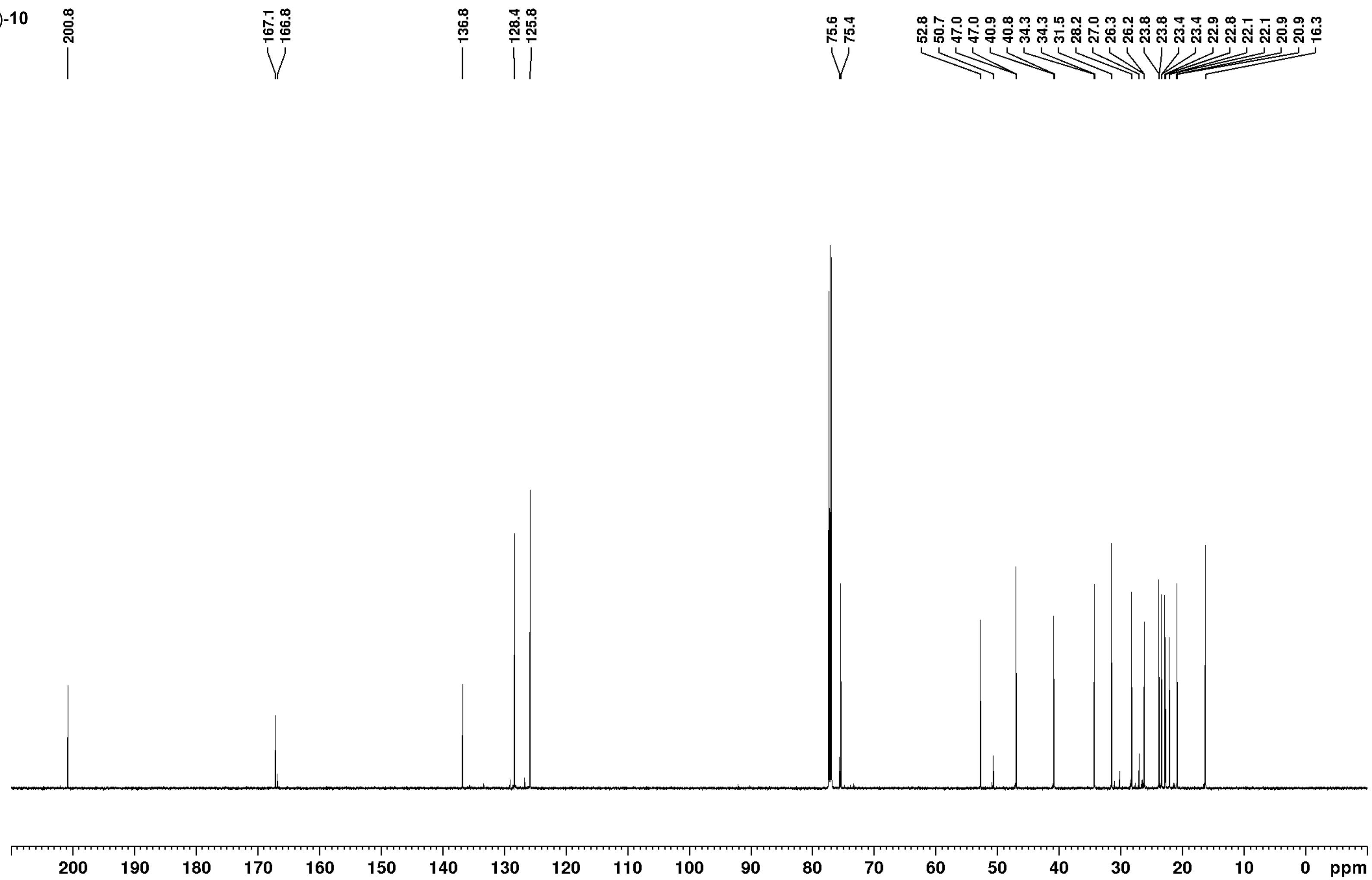

Figure S44: ^{13}C NMR spectrum (L)-**10**.

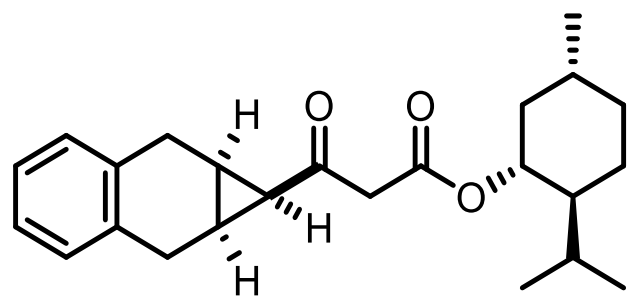

(L)-**10**

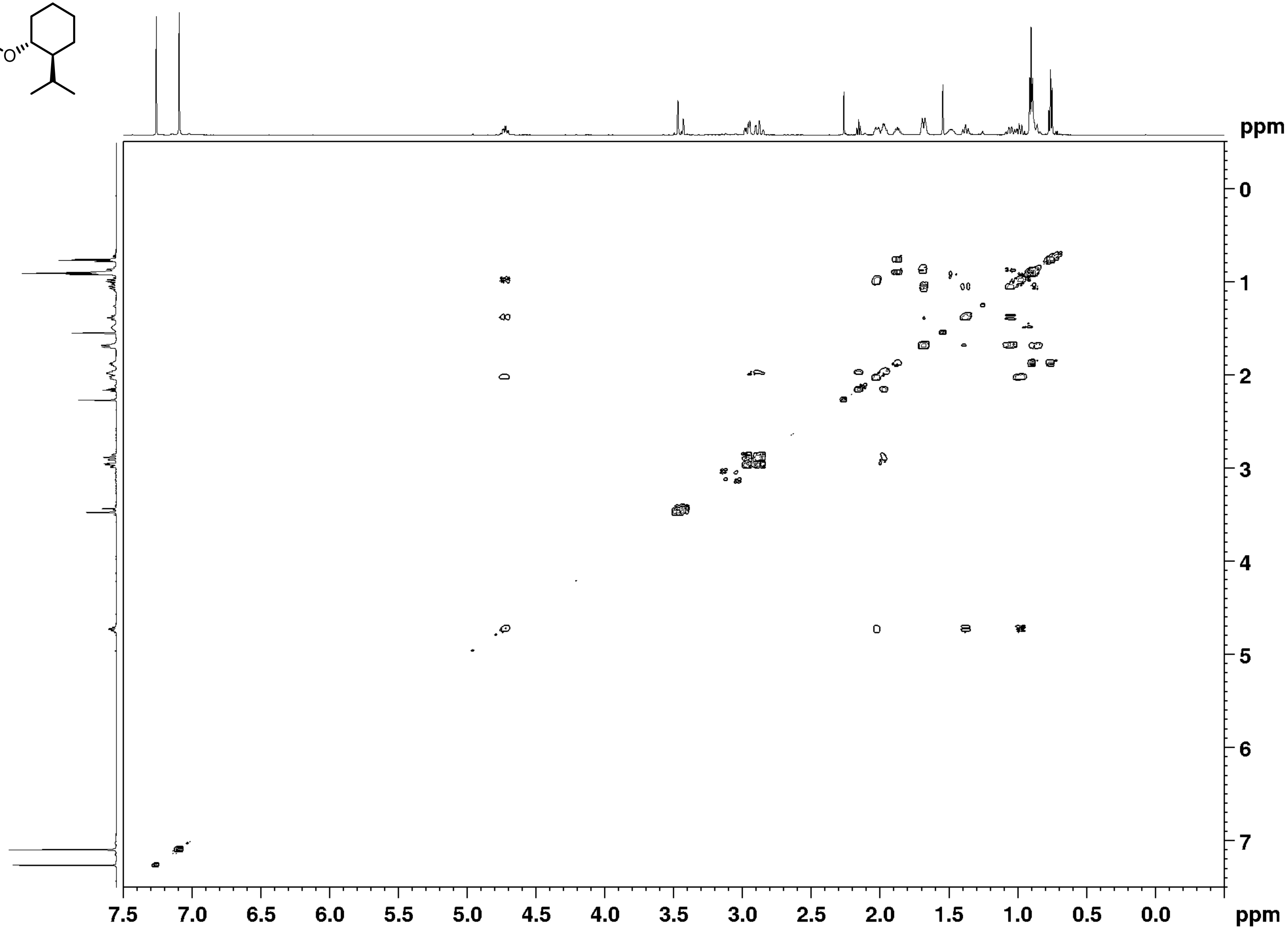

Figure S45: ^{1}H, ^{1}H COSY spectrum of (L)-**10**.

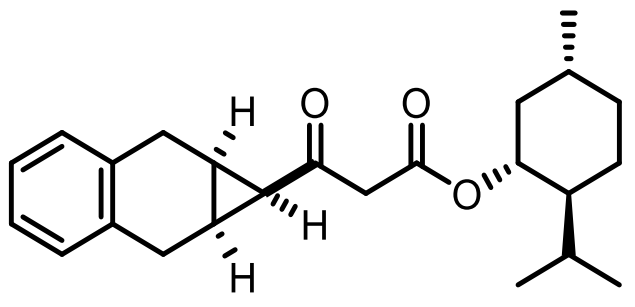

(L)-**10**

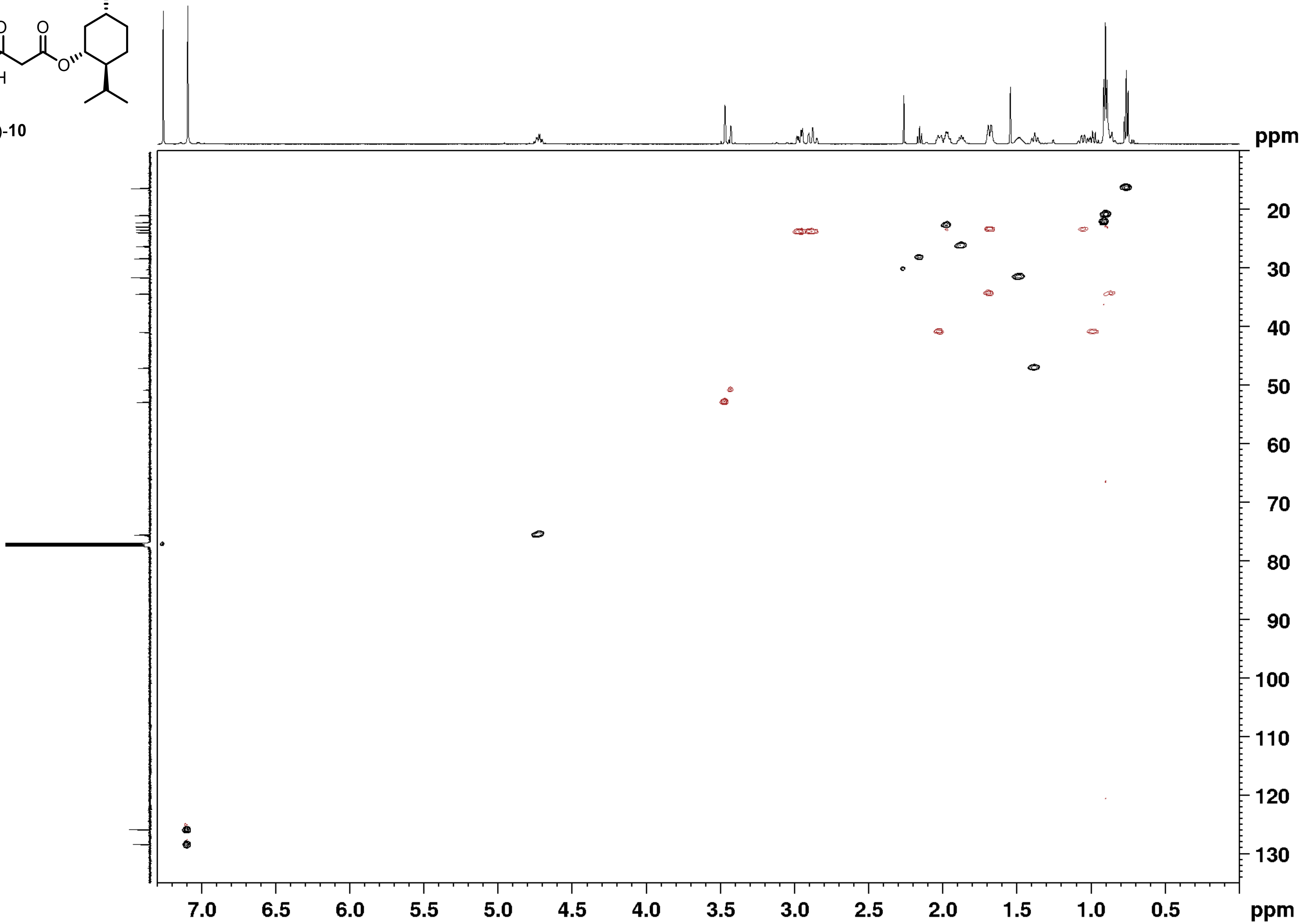

Figure S46: ^{1}H, ^{13}C HSQC spectrum of (L)-**10**.

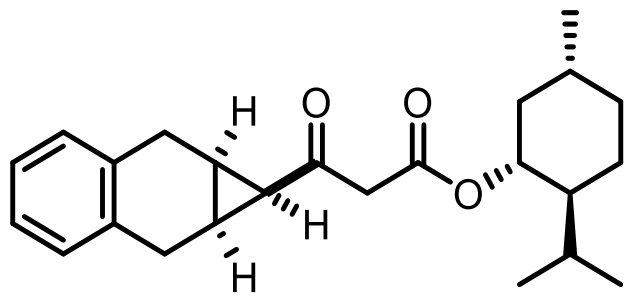

(L)-**10**

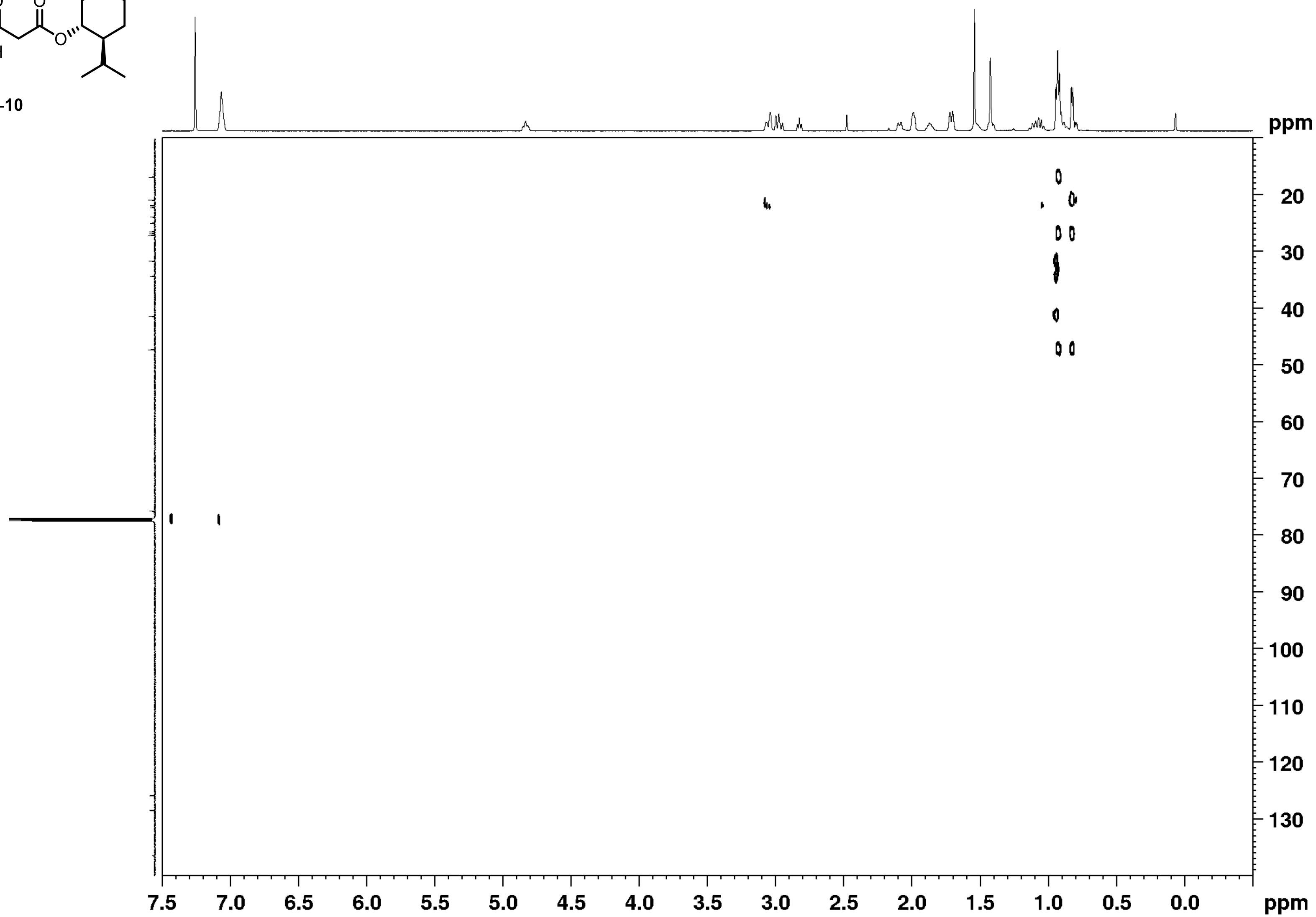

Figure S47: ^{1}H, ^{13}C HMBC spectrum of (L)-**10**.

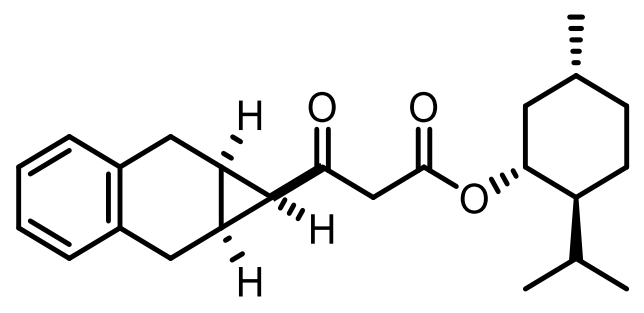

(L)-**10**

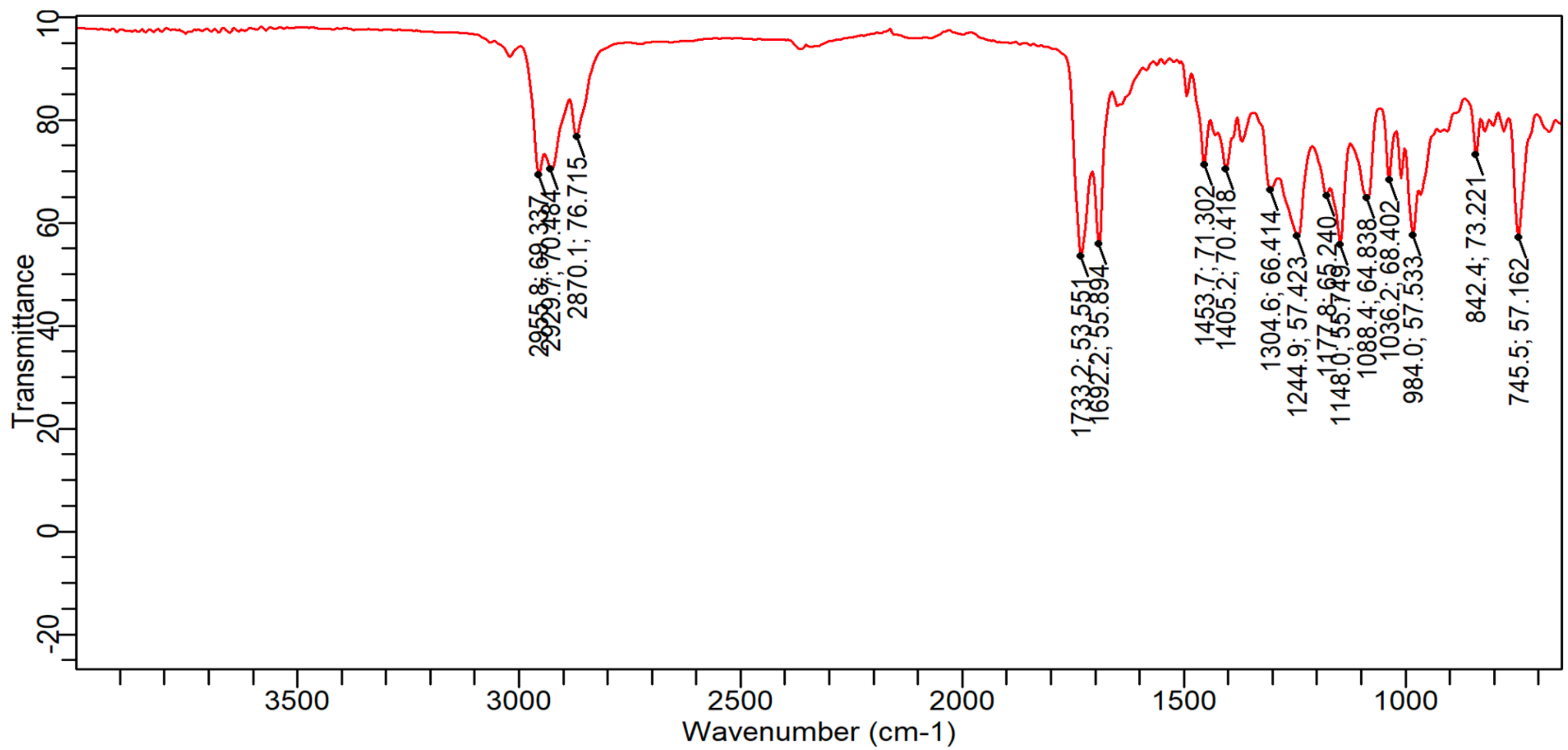

Figure S48: IR spectrum of (L)-**10**.

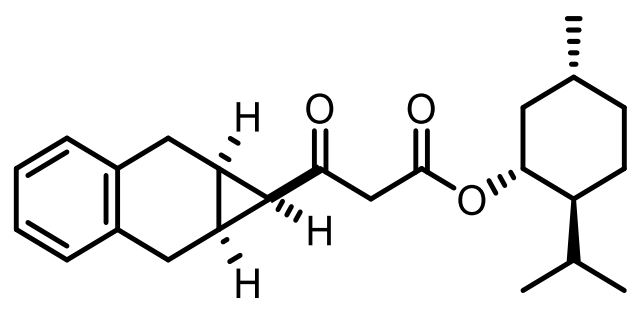

(L)-**10**

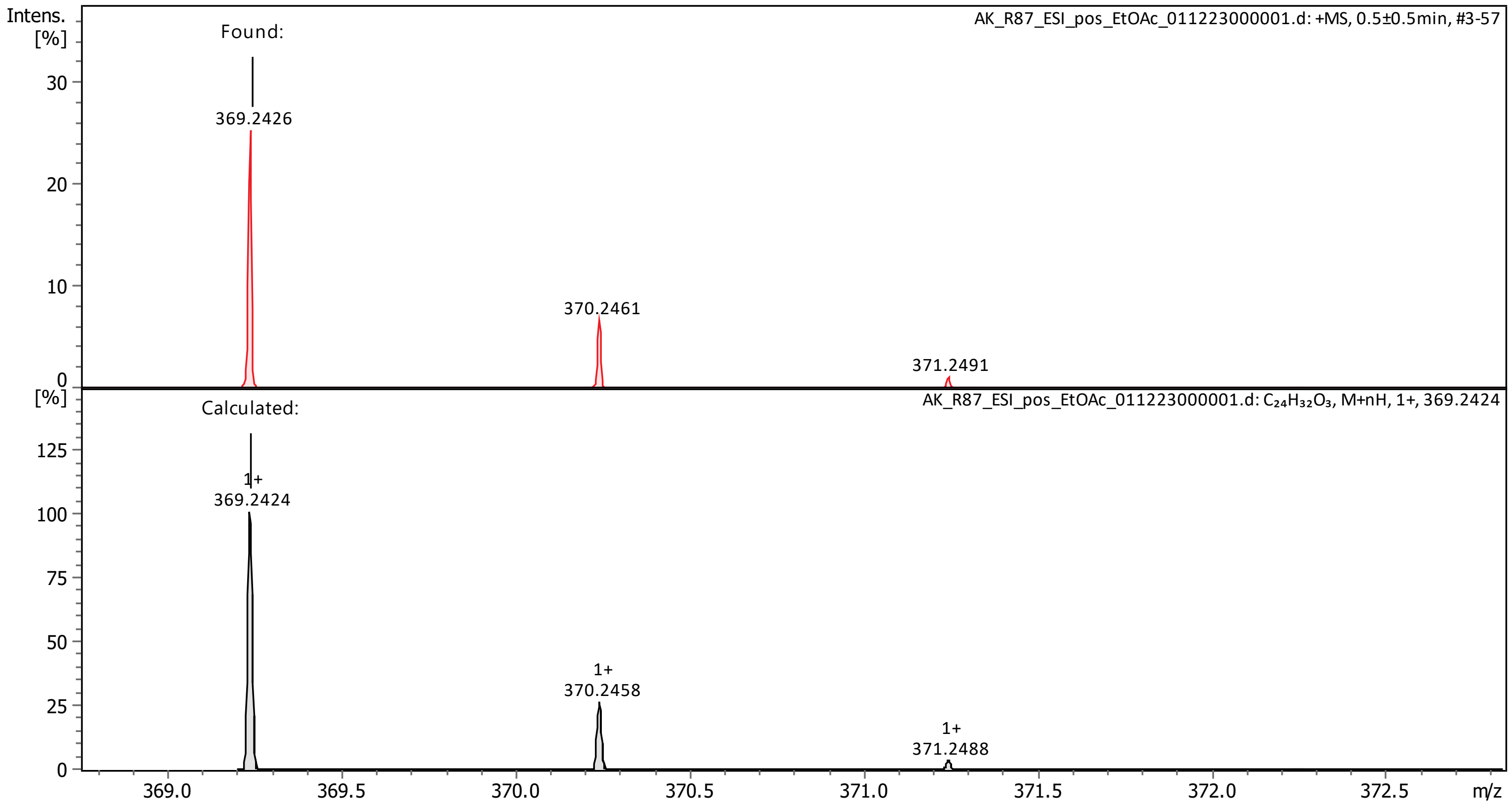

Figure S49: HRMS spectrum of (L)-**10**.

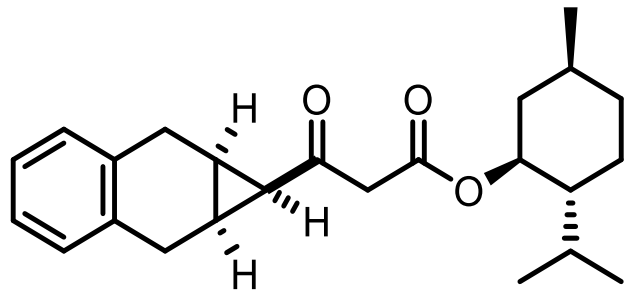

(D)-**10**

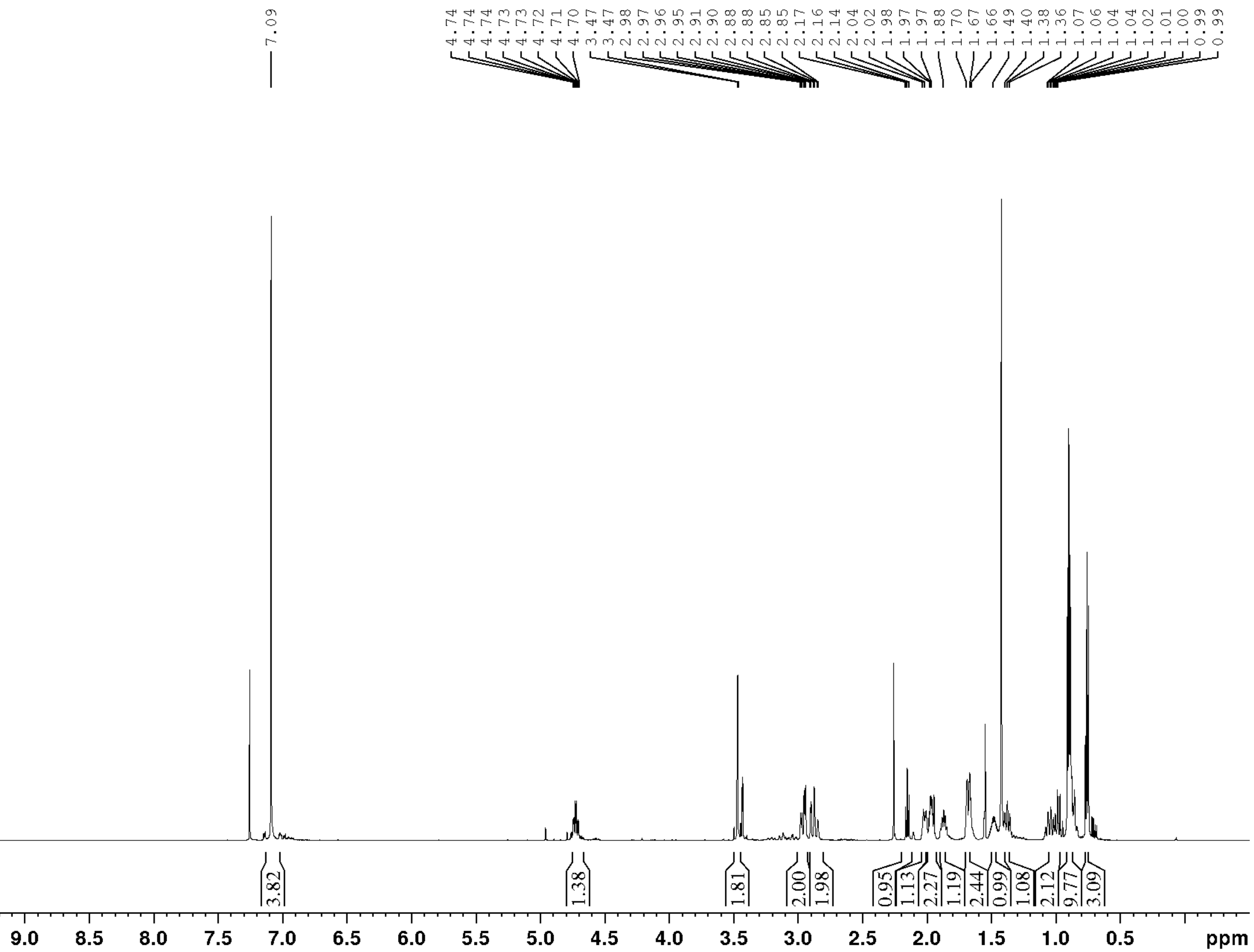

Figure S50: ^{1}H NMR spectrum of (D)-**10**.

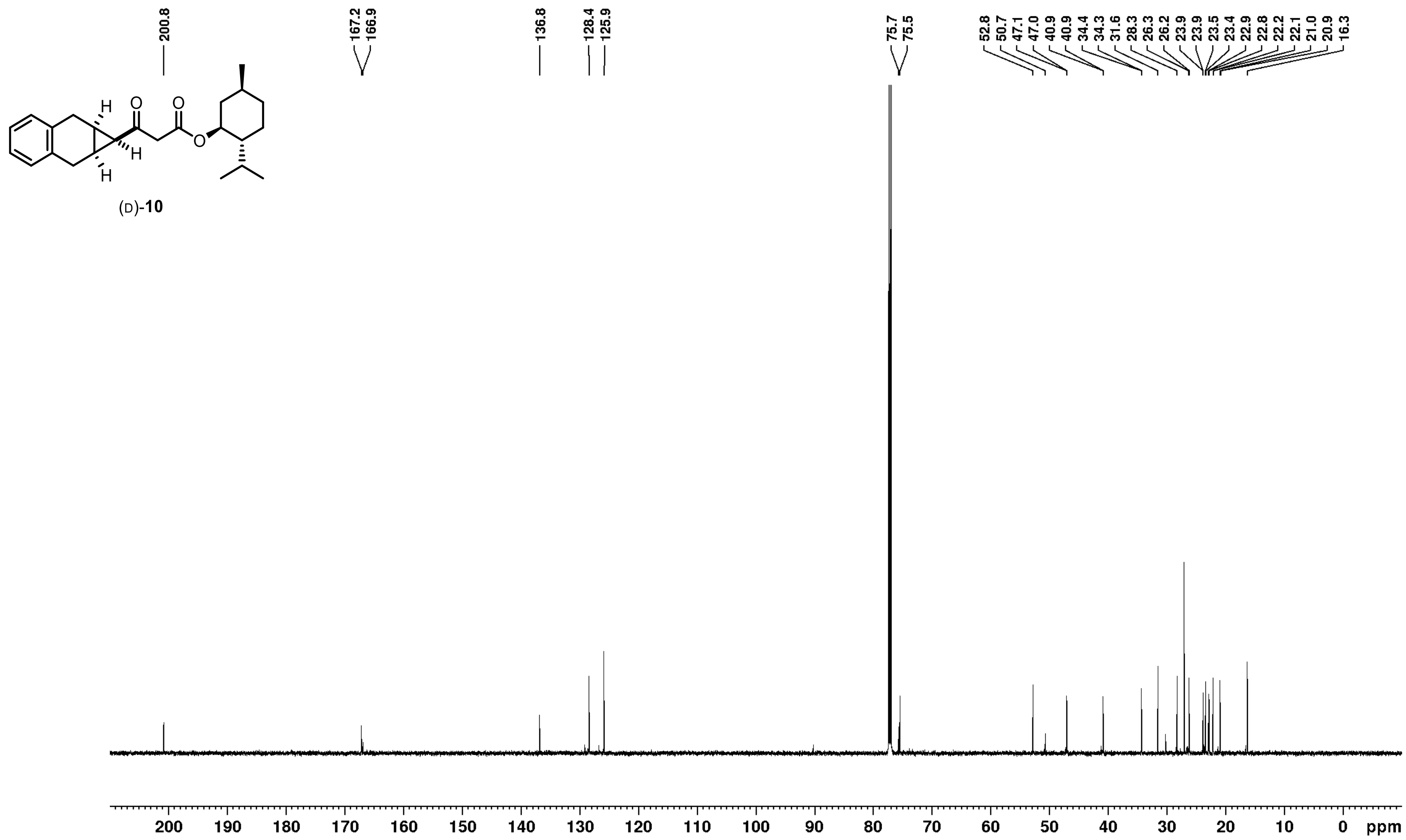

Figure S51: ^{13}C NMR spectrum of (D)-**10**.

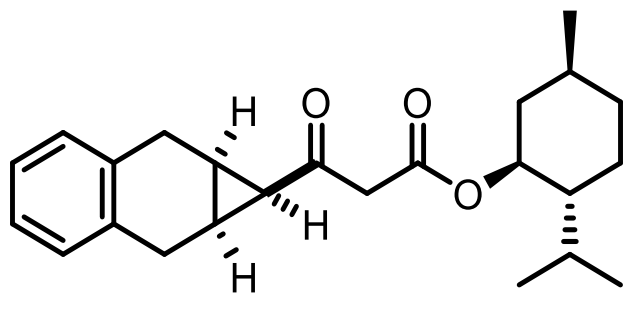

(D)-**10**

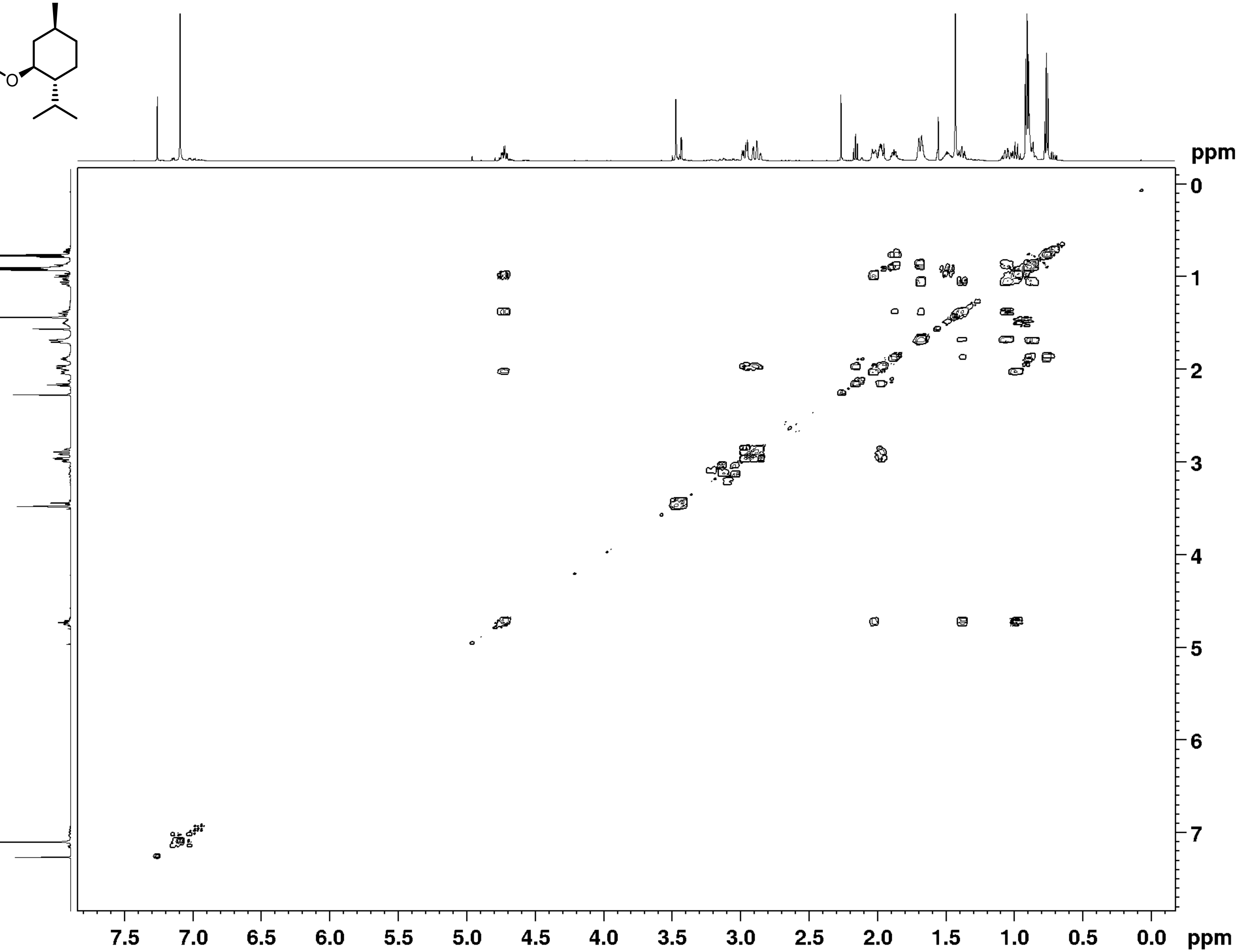

Figure S52: ^{1}H, ^{1}H COSY spectrum of (D)-**10**.

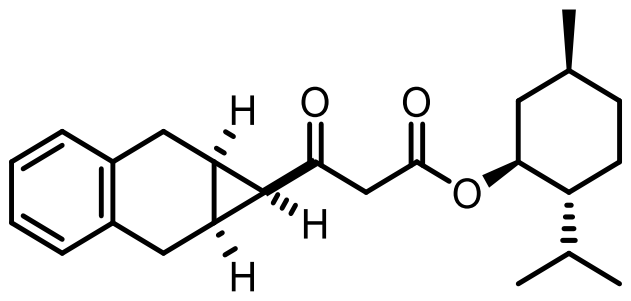

(D)-**10**

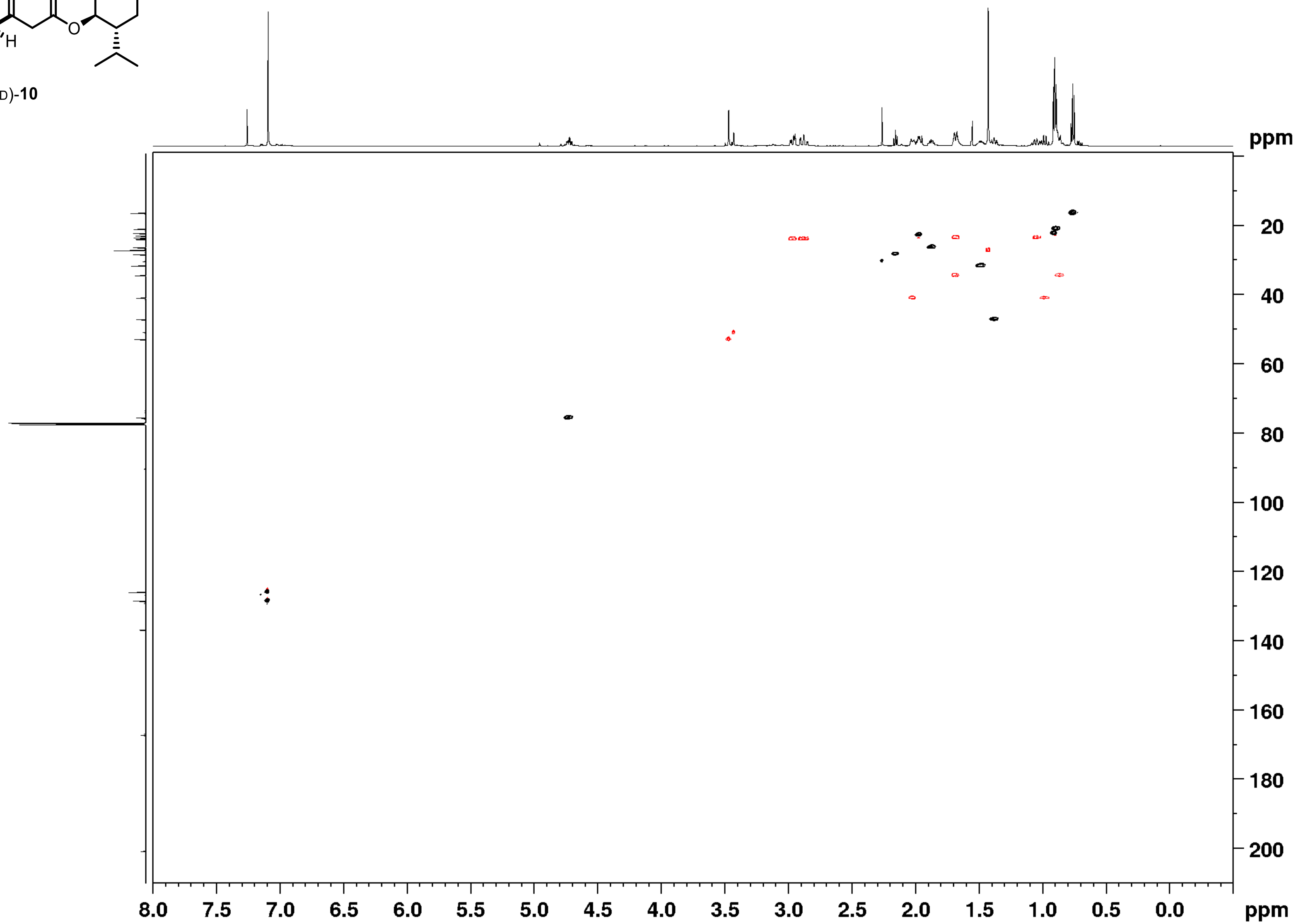

Figure S53: ^{1}H, ^{13}C HSQC spectrum of (D)-**10**.

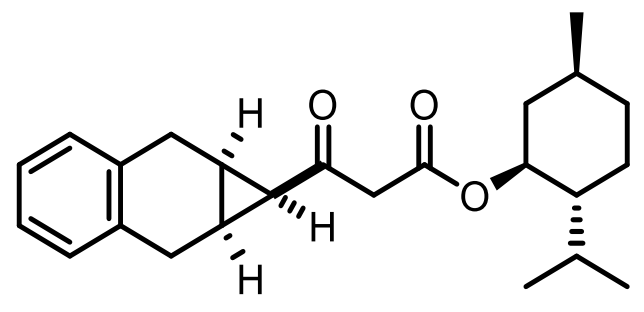

(D)-**10**

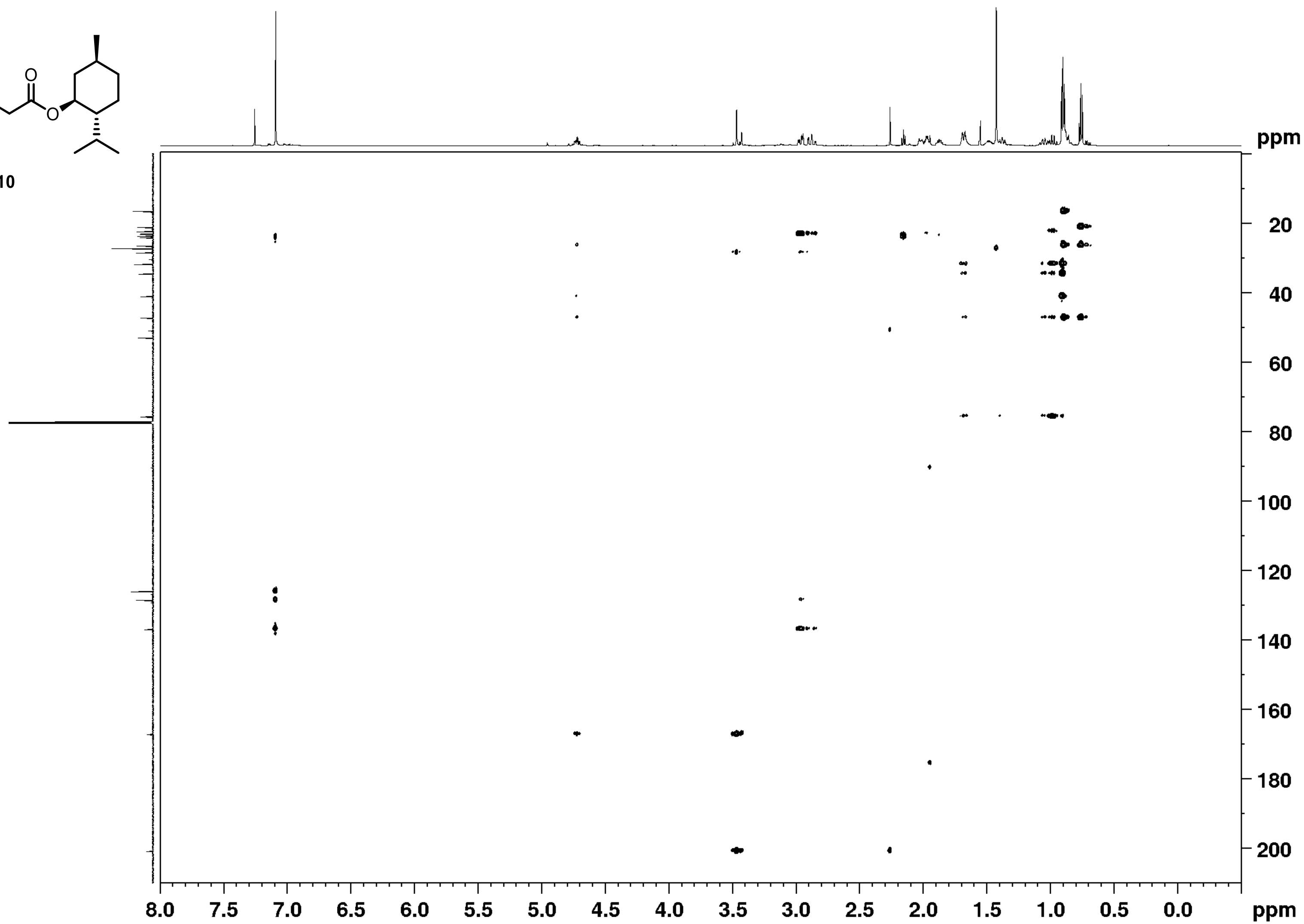

Figure S54: ^{1}H, ^{13}C HMBC spectrum of (D)-**10**.

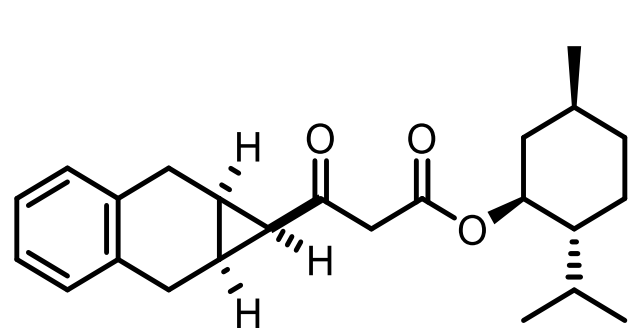

(D)-**10**

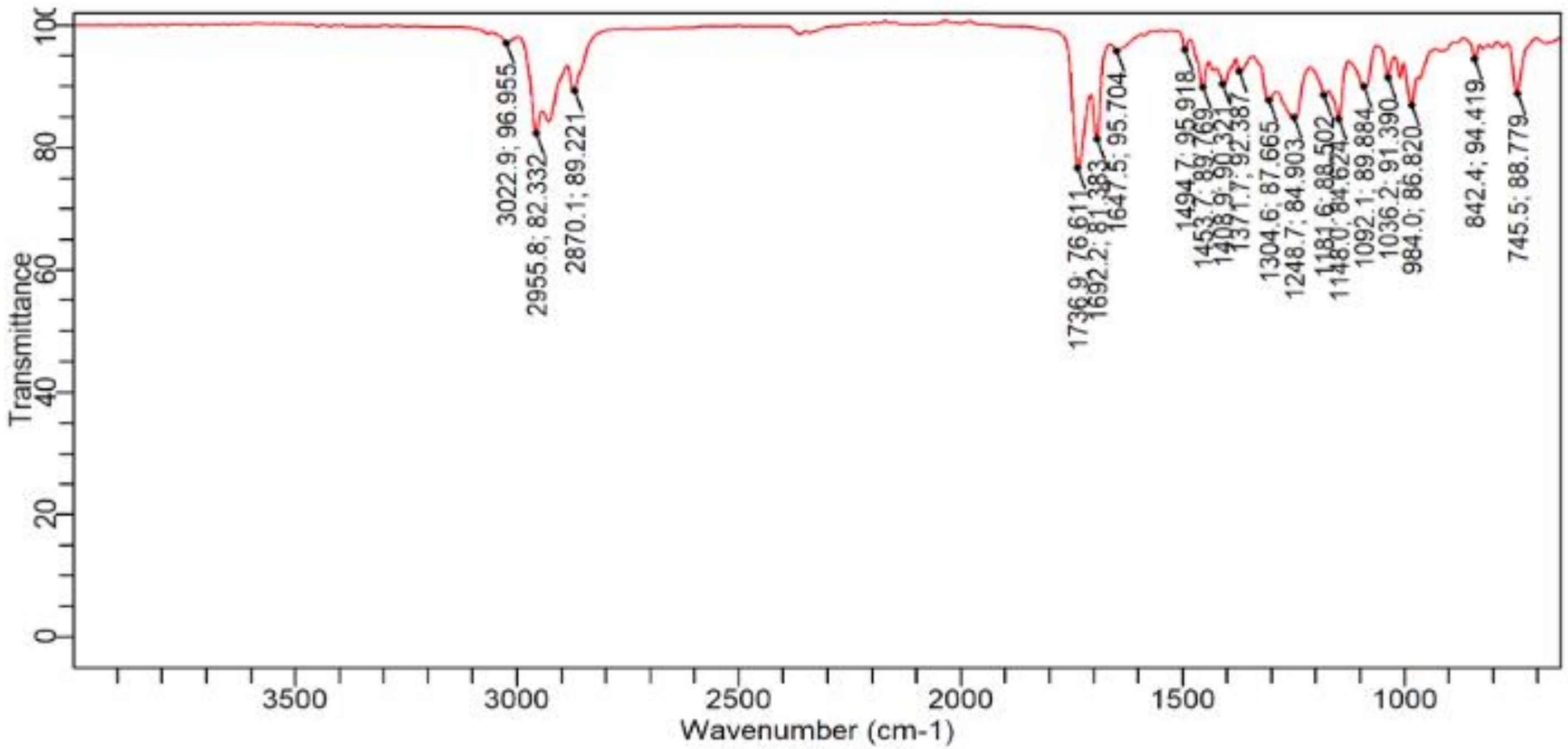

Figure S55: IR spectrum of (D)-**10**.

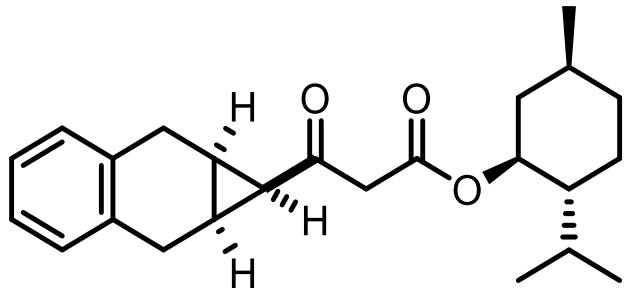

(D)-**10**

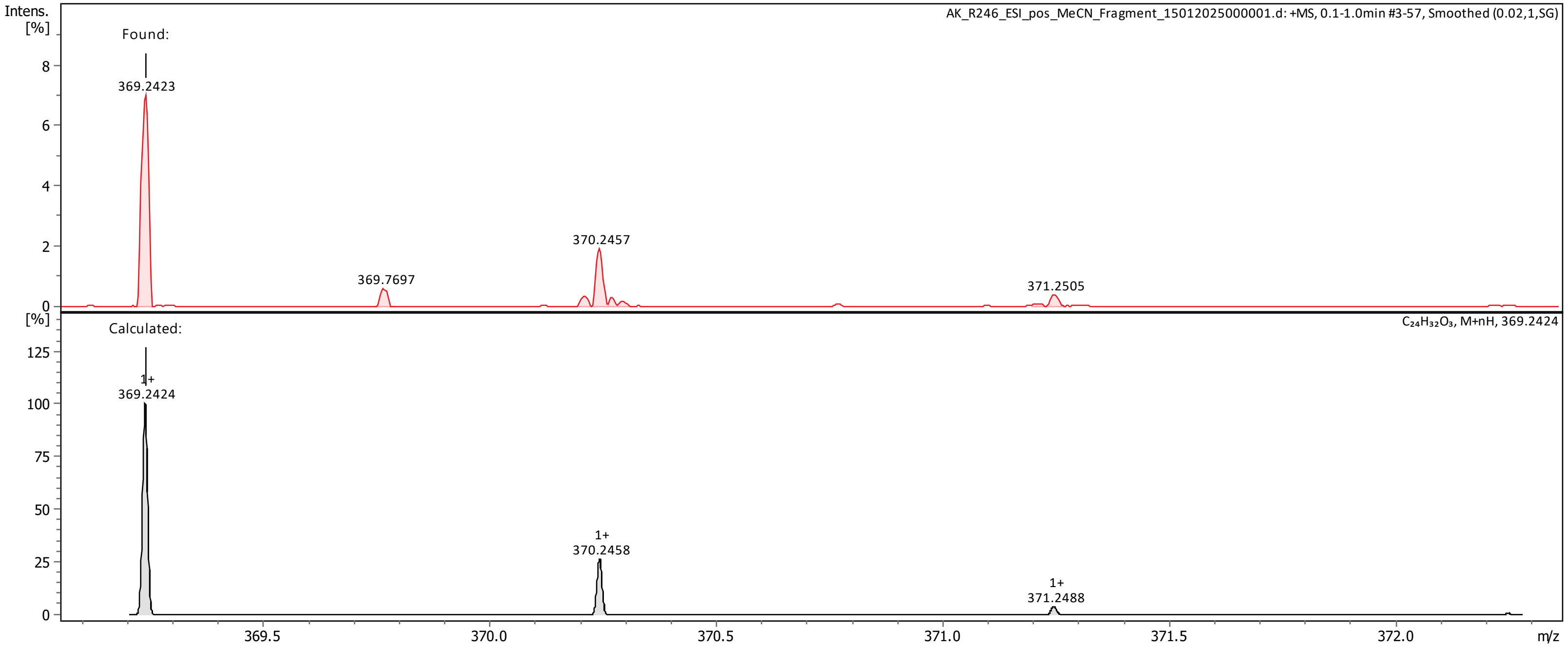

Figure S56: HRMS chromatogram of (D)-**10**.

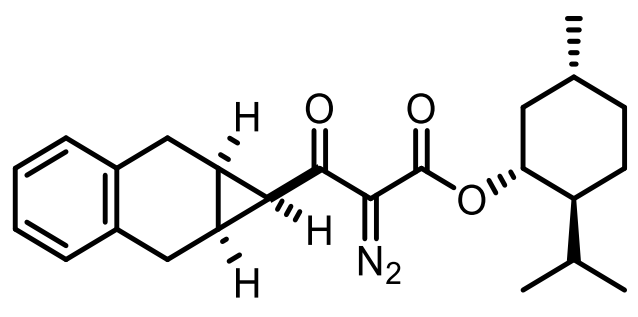

(L)-**11**

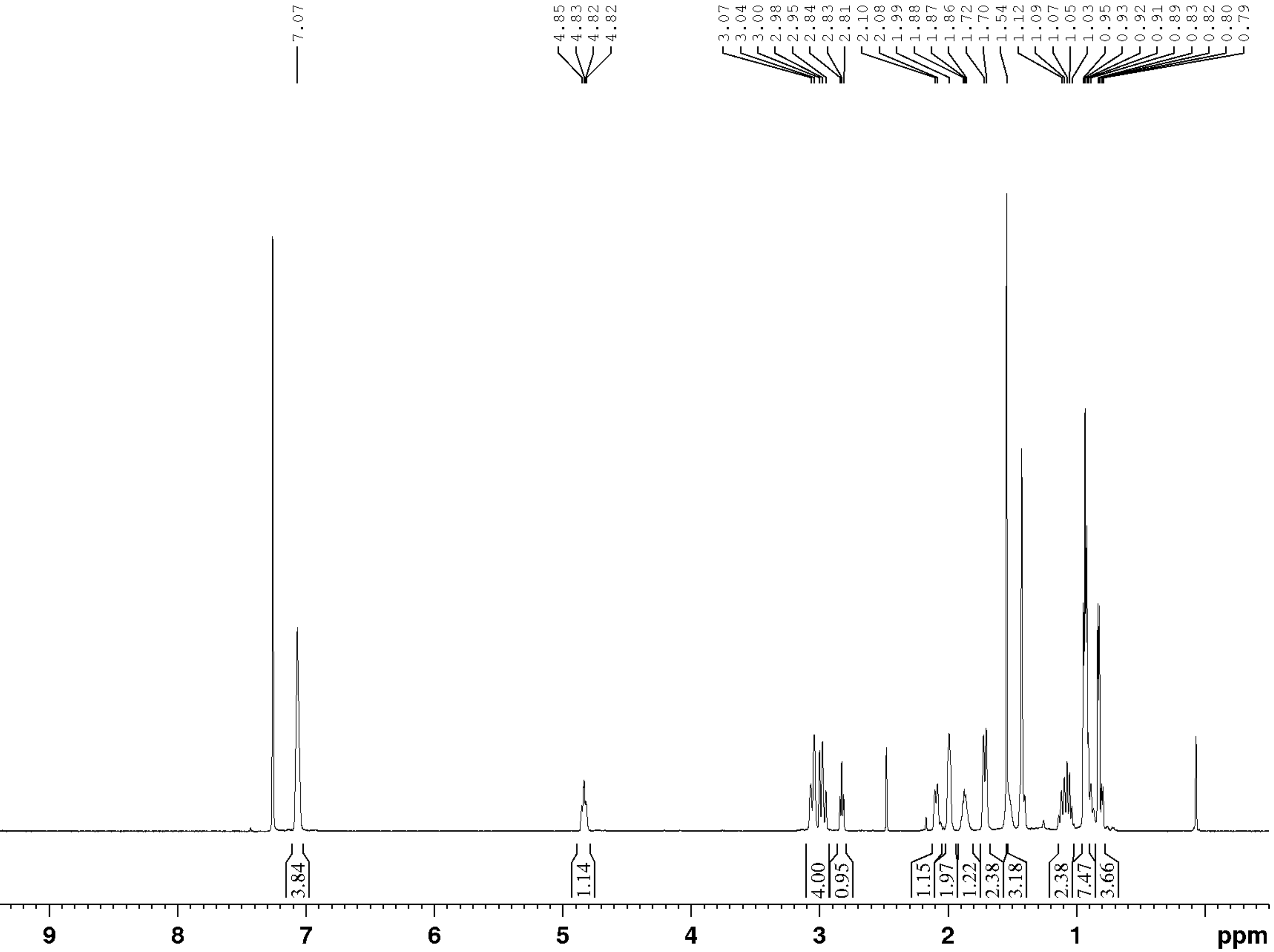

Figure S57: ^{1}H NMR spectrum of (L)-**11**.

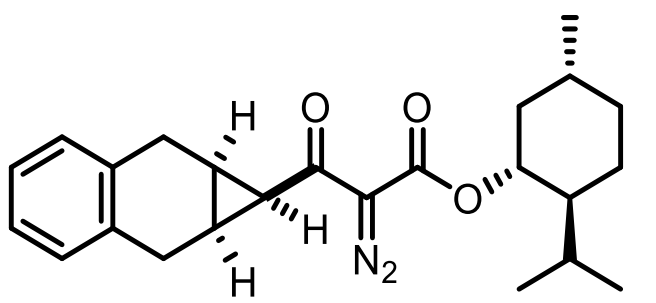

(L)-**11**

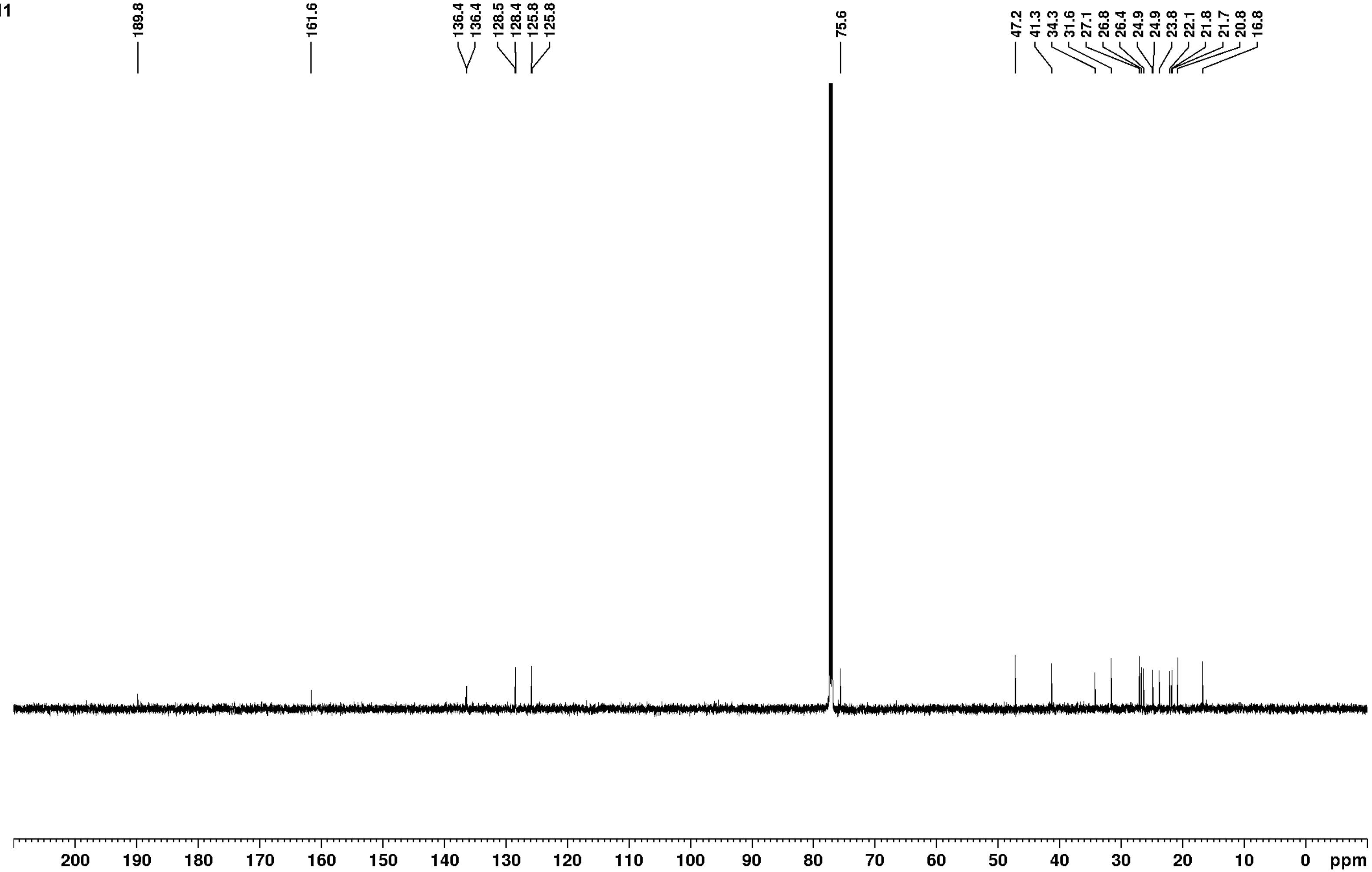

Figure S58: ^{13}C NMR spectrum of (L)-**11**.

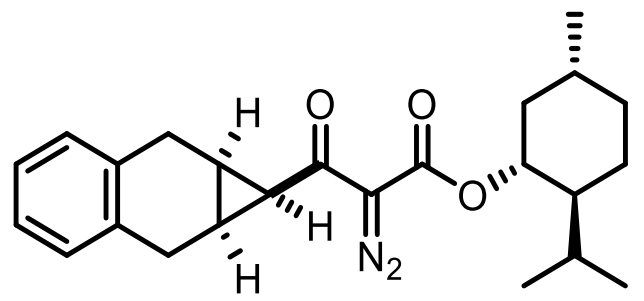

(L)-**11**

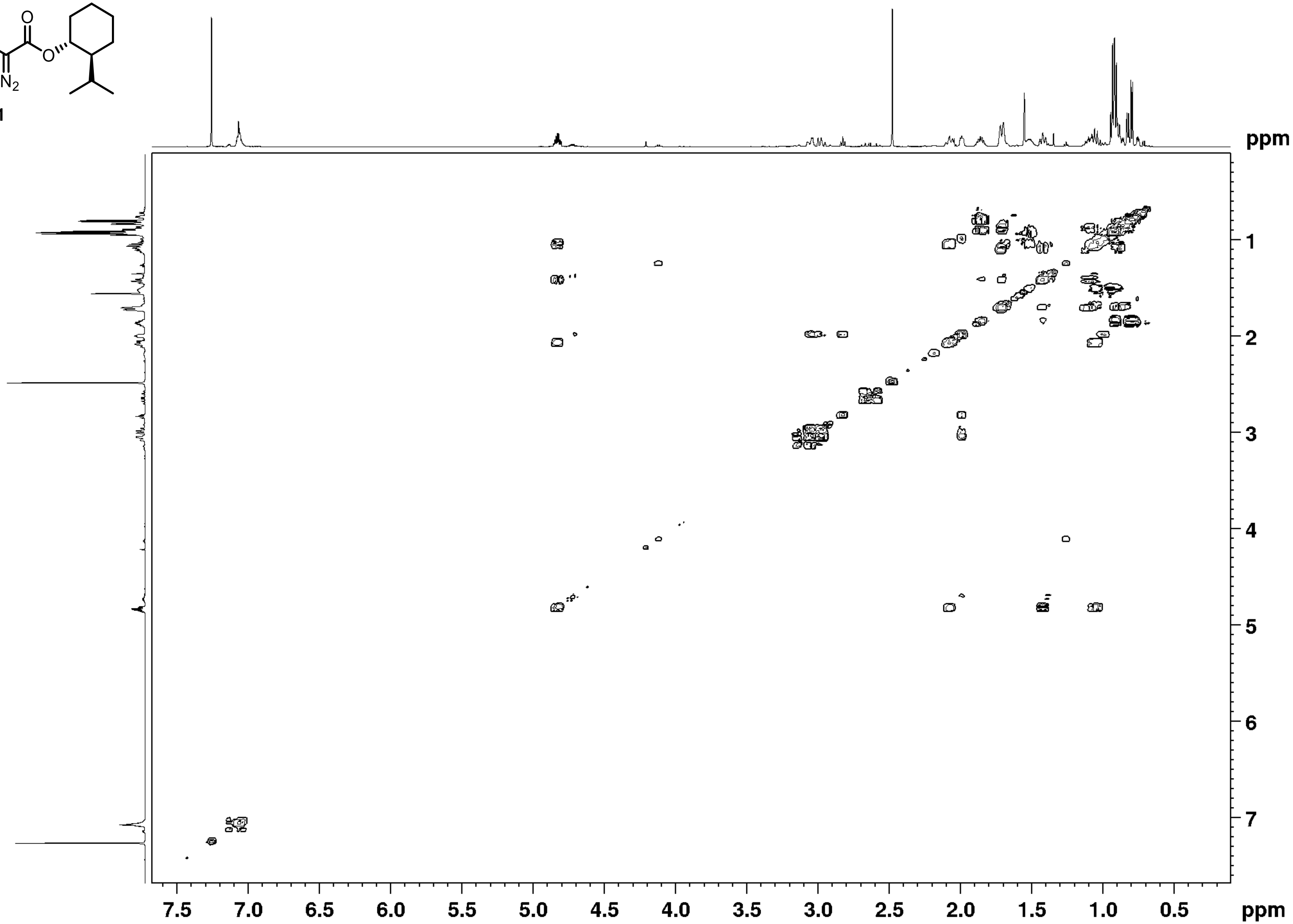

Figure S59: ^{1}H, ^{1}H COSY spectrum of (L)-**11**.

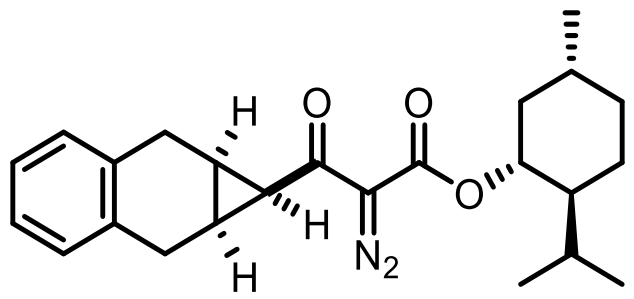

(L)-**11**

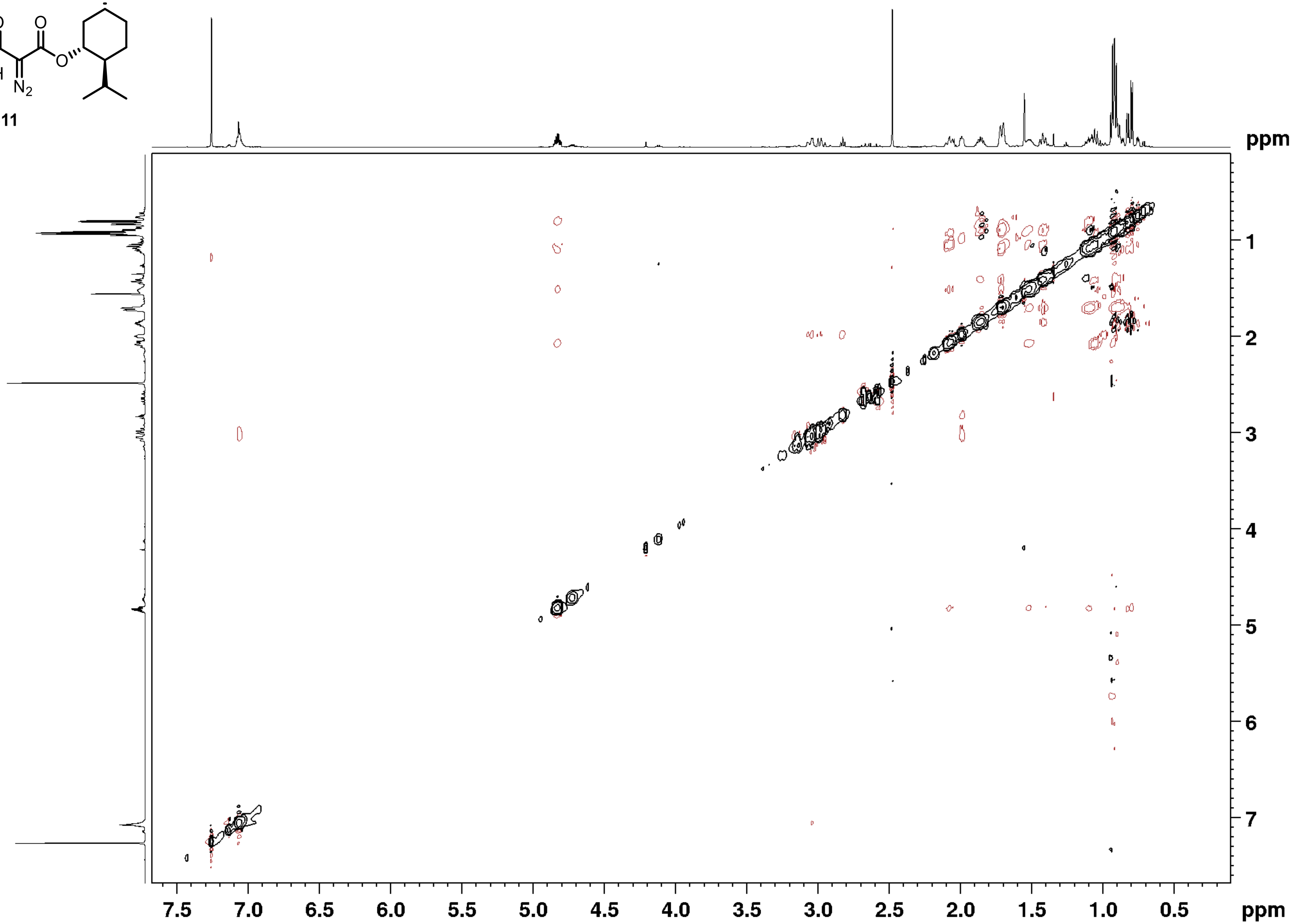

Figure S60: ^{1}H, ^{1}H NOESY spectrum of (L)-**11**.

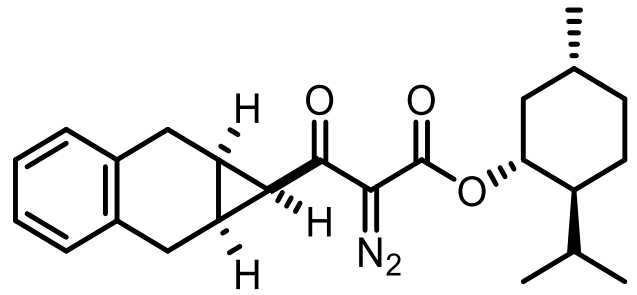

(L)-**11**

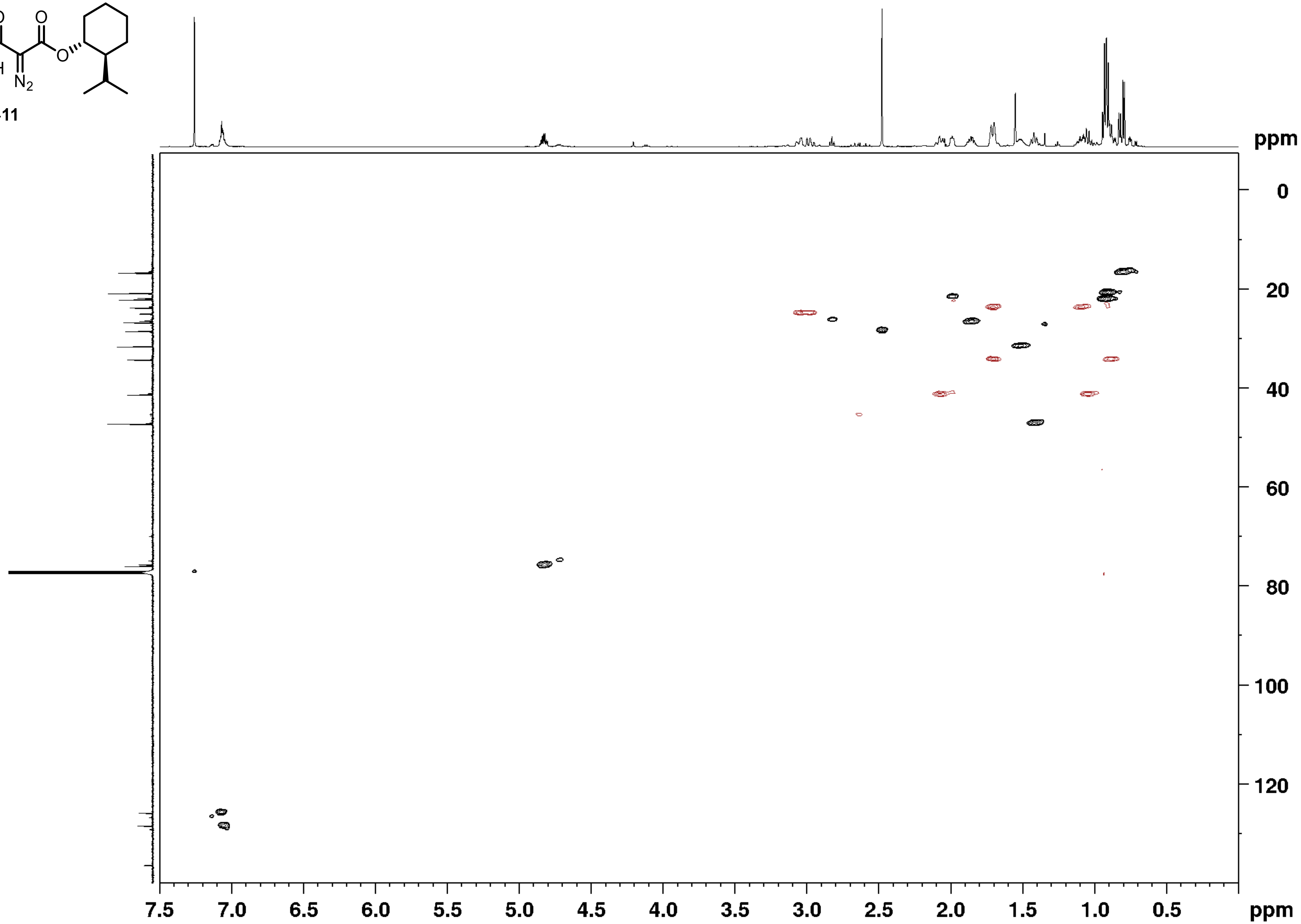

Figure S61: ^{1}H, ^{13}C HSQC spectrum of (L)-**11**.

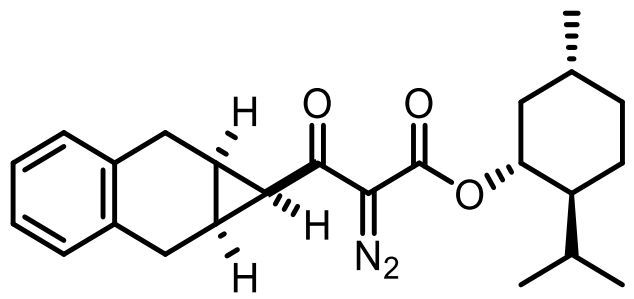

(L)-**11**

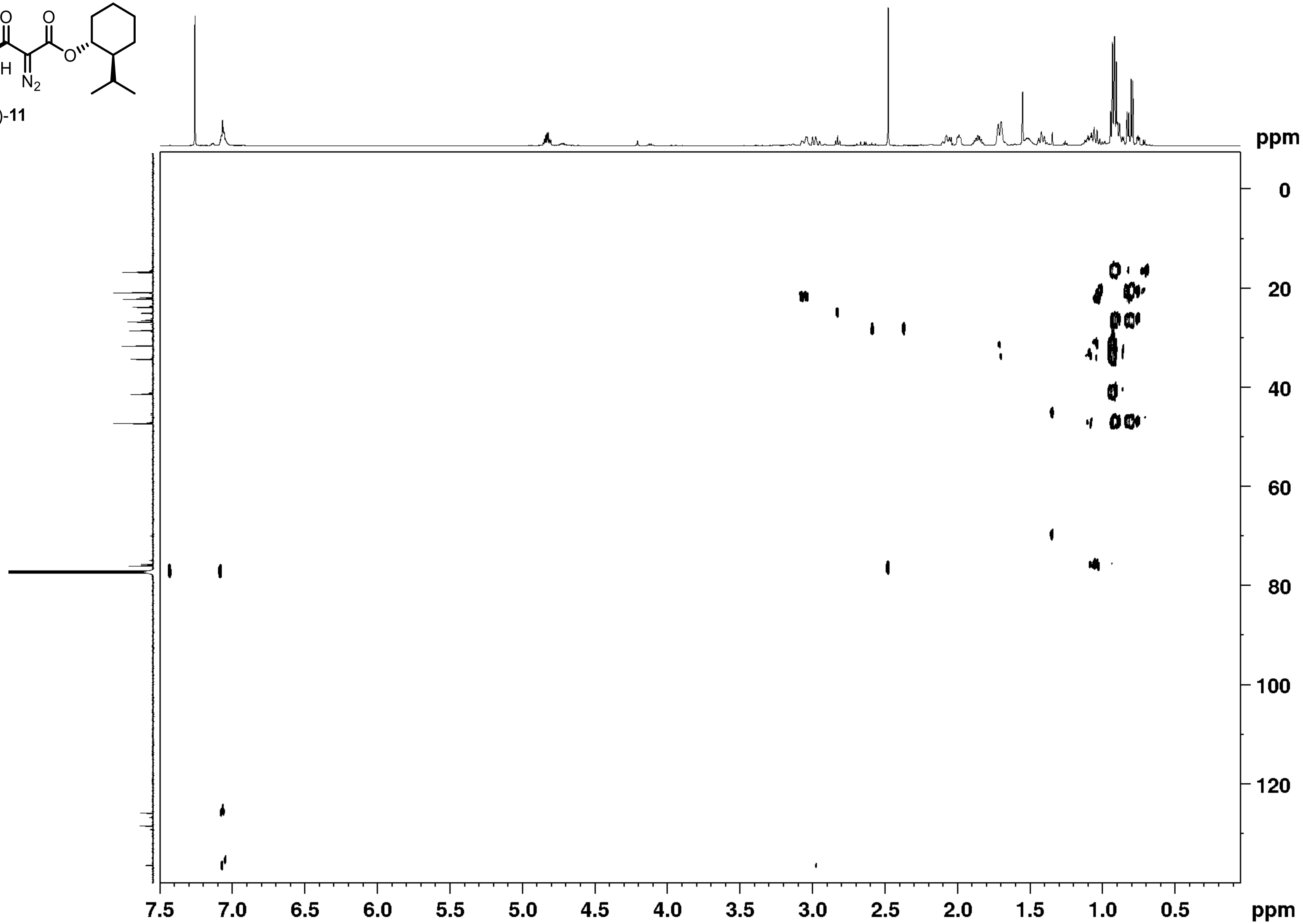

Figure S62: ^{1}H, ^{13}C HMBC spectrum of (L)-**11**.

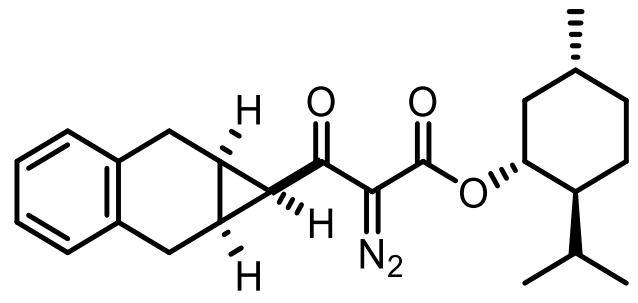

(L)-**11**

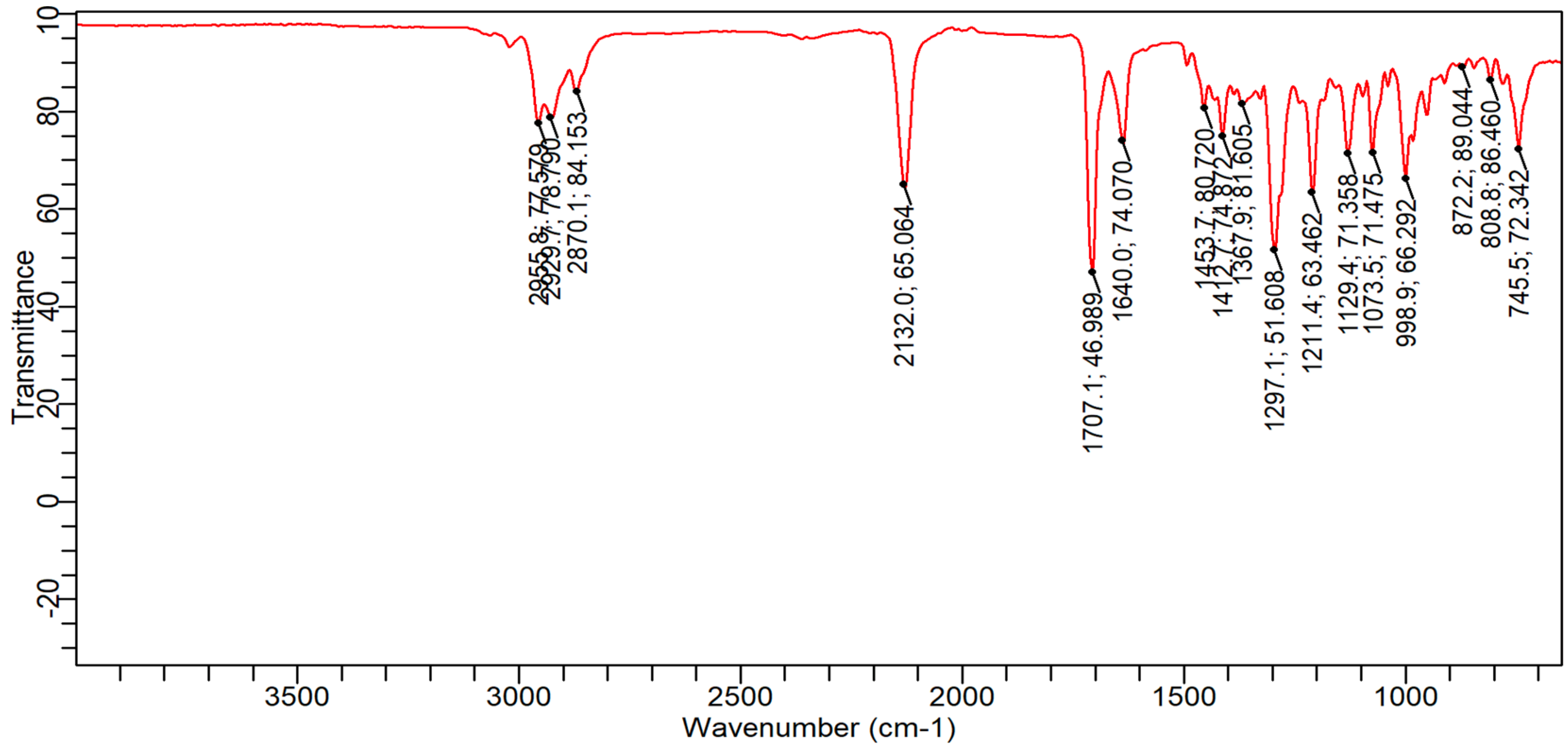

Figure S63: IR spectrum of (L)-**11**.

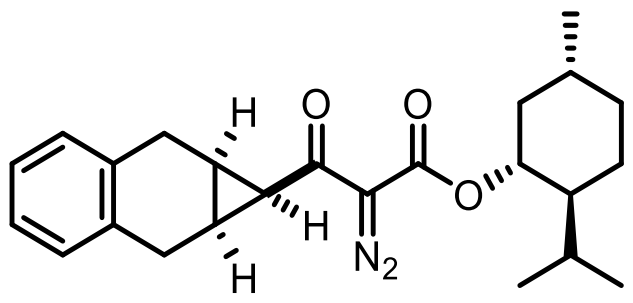

(L)-**11**

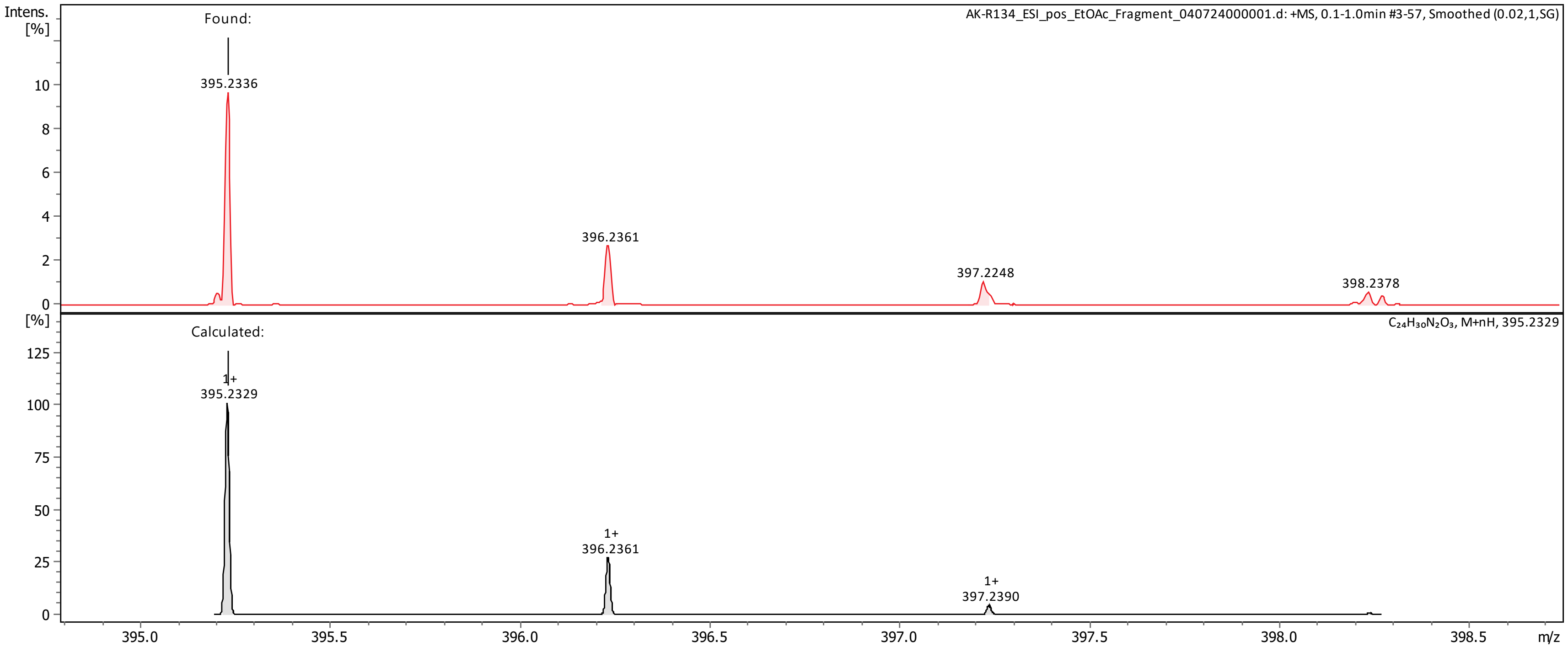

Figure S64: HRMS chromatogram of (L)-**11**.

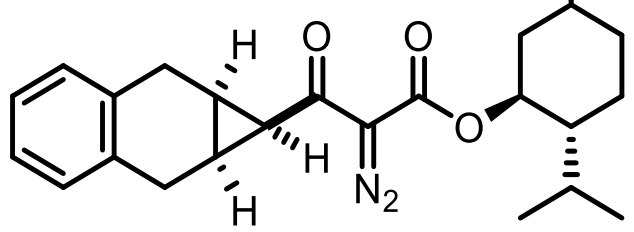

(D)-**11**

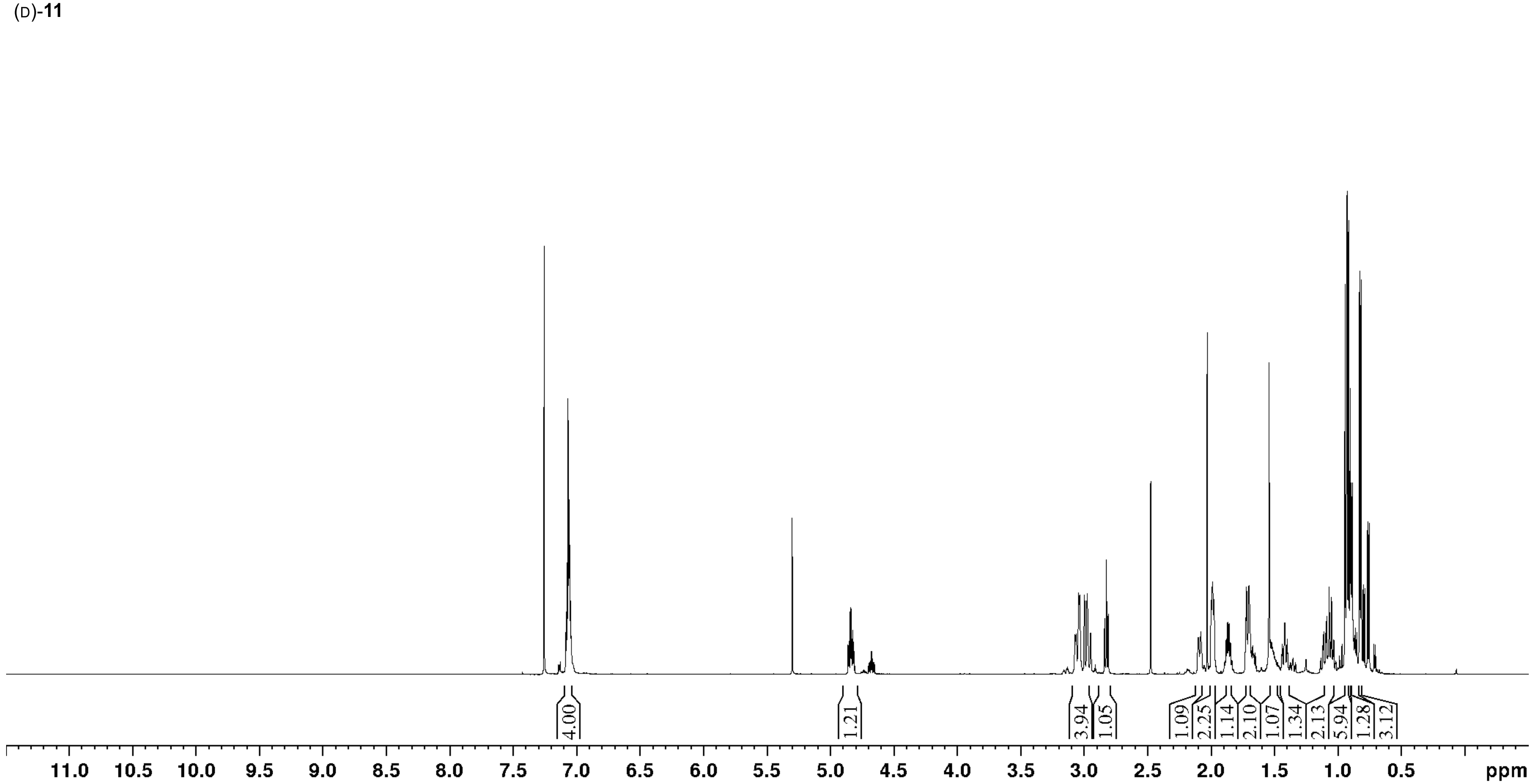

Figure S65: ^{1}H NMR spectrum of (D)-**11**.

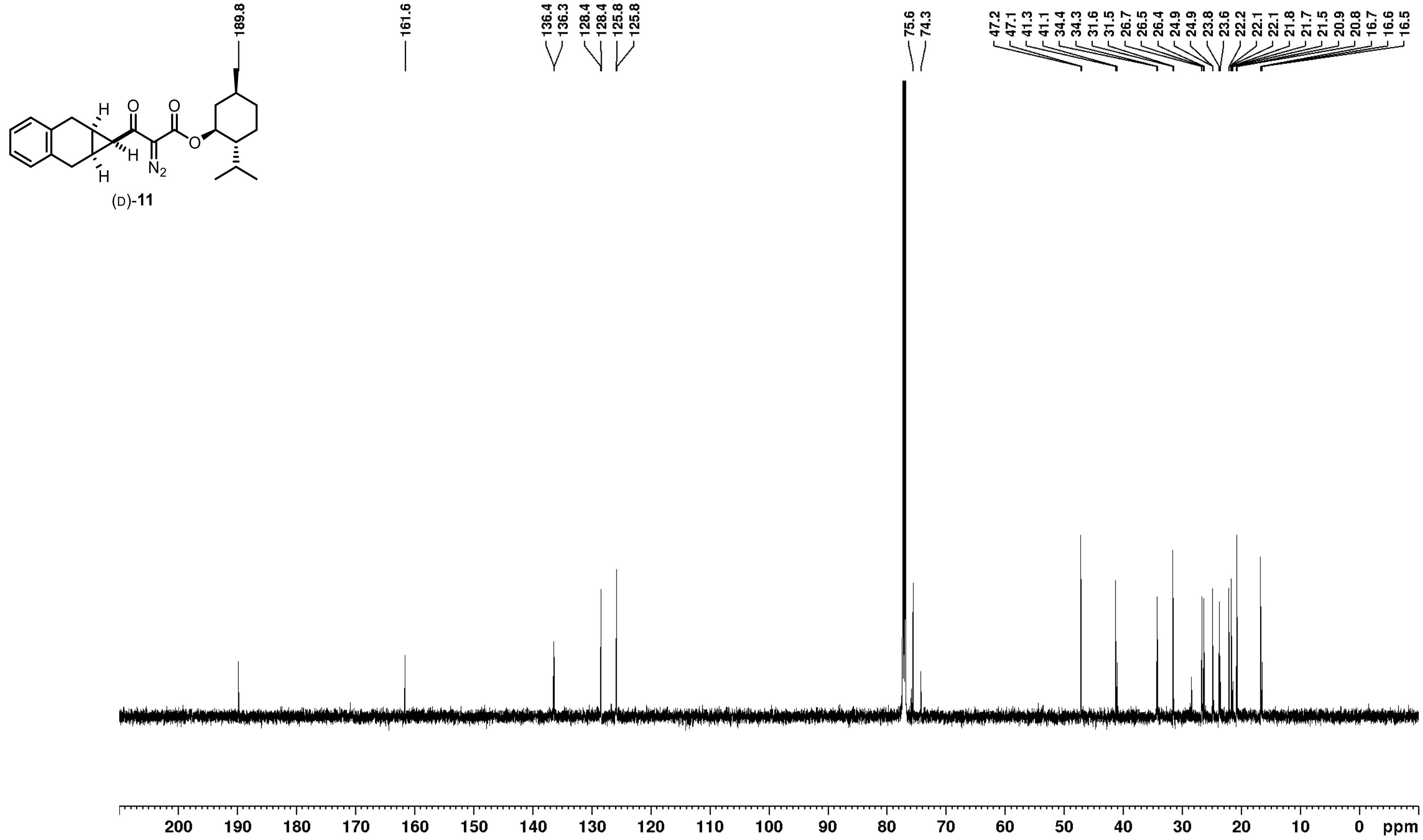

Figure S66: ^{13}C NMR spectrum of (D)-**11**.

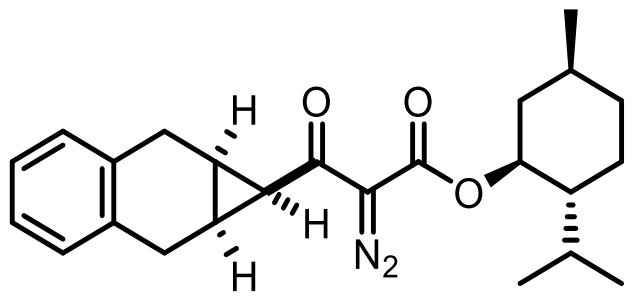

(D)-**11**

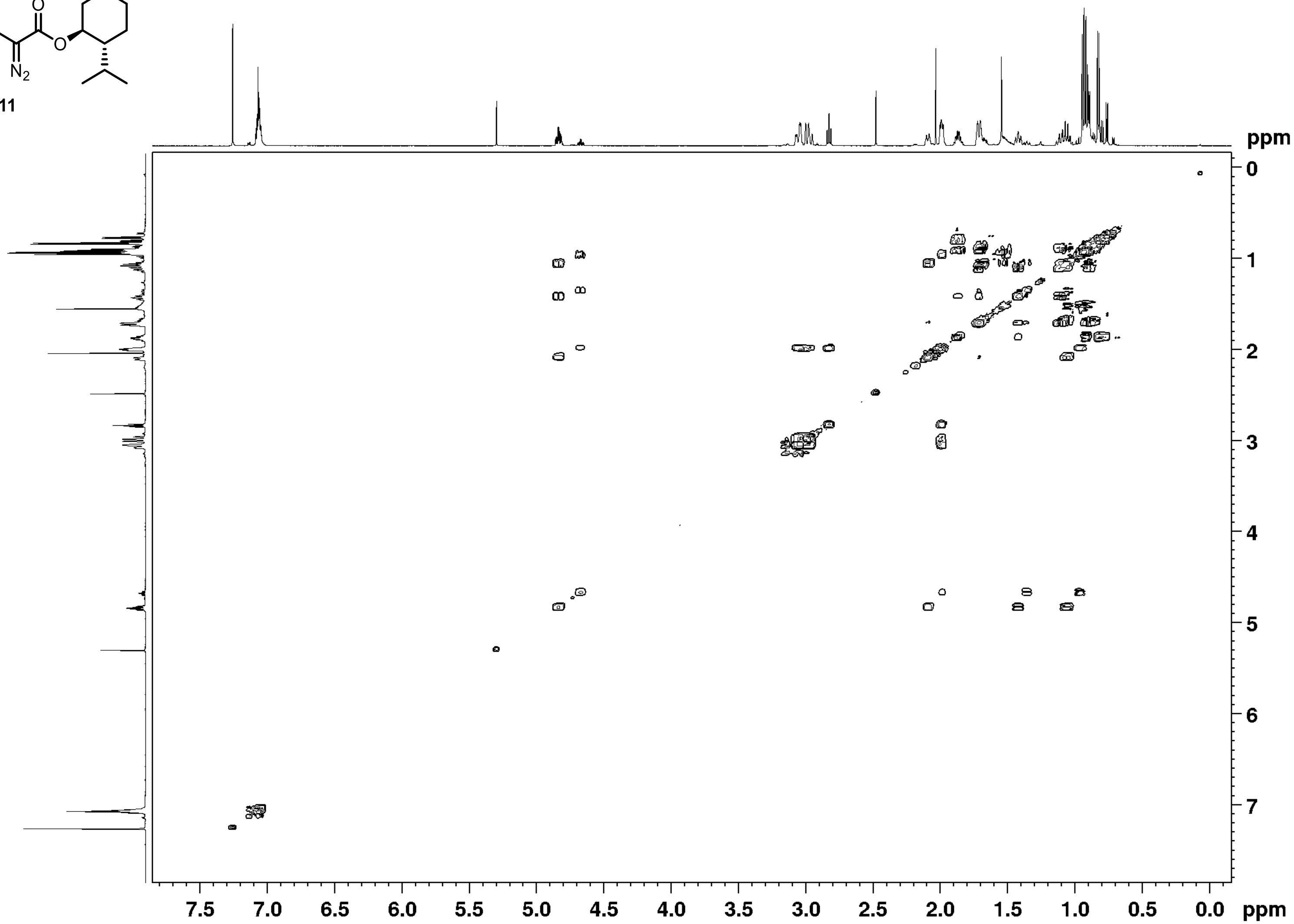

Figure S67: ^{1}H, ^{1}H COSY spectrum of (D)-**11**.

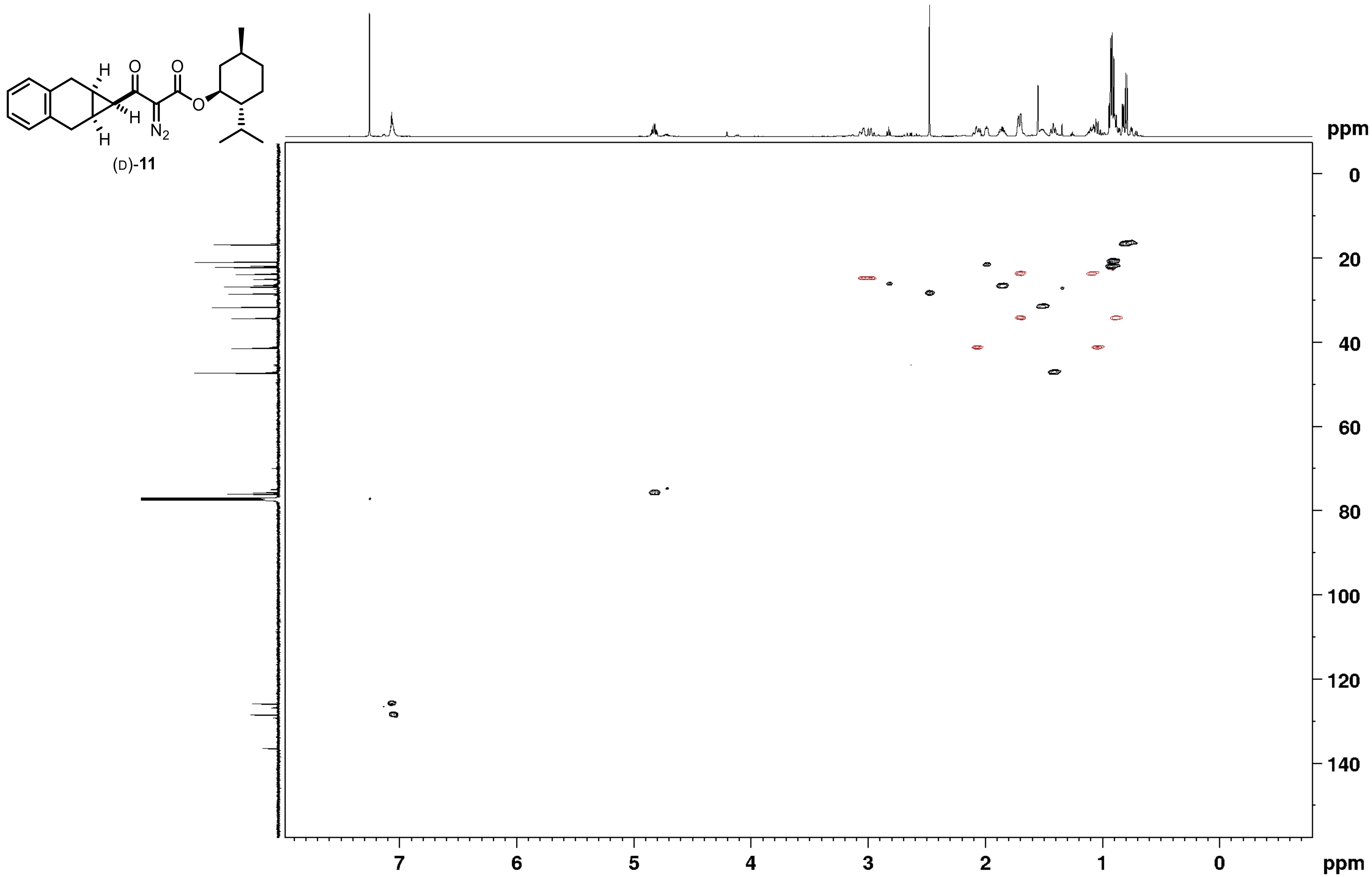

Figure S68: ^{1}H, ^{13}C HSQC spectrum of (D)-**11**.

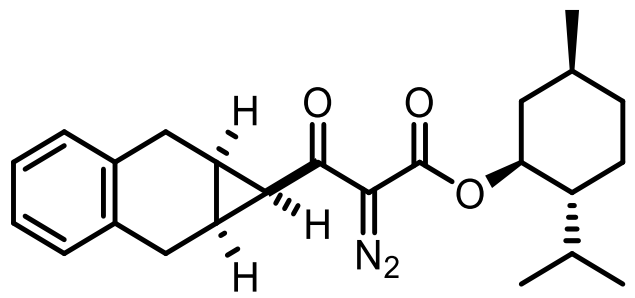

(D)-**11**

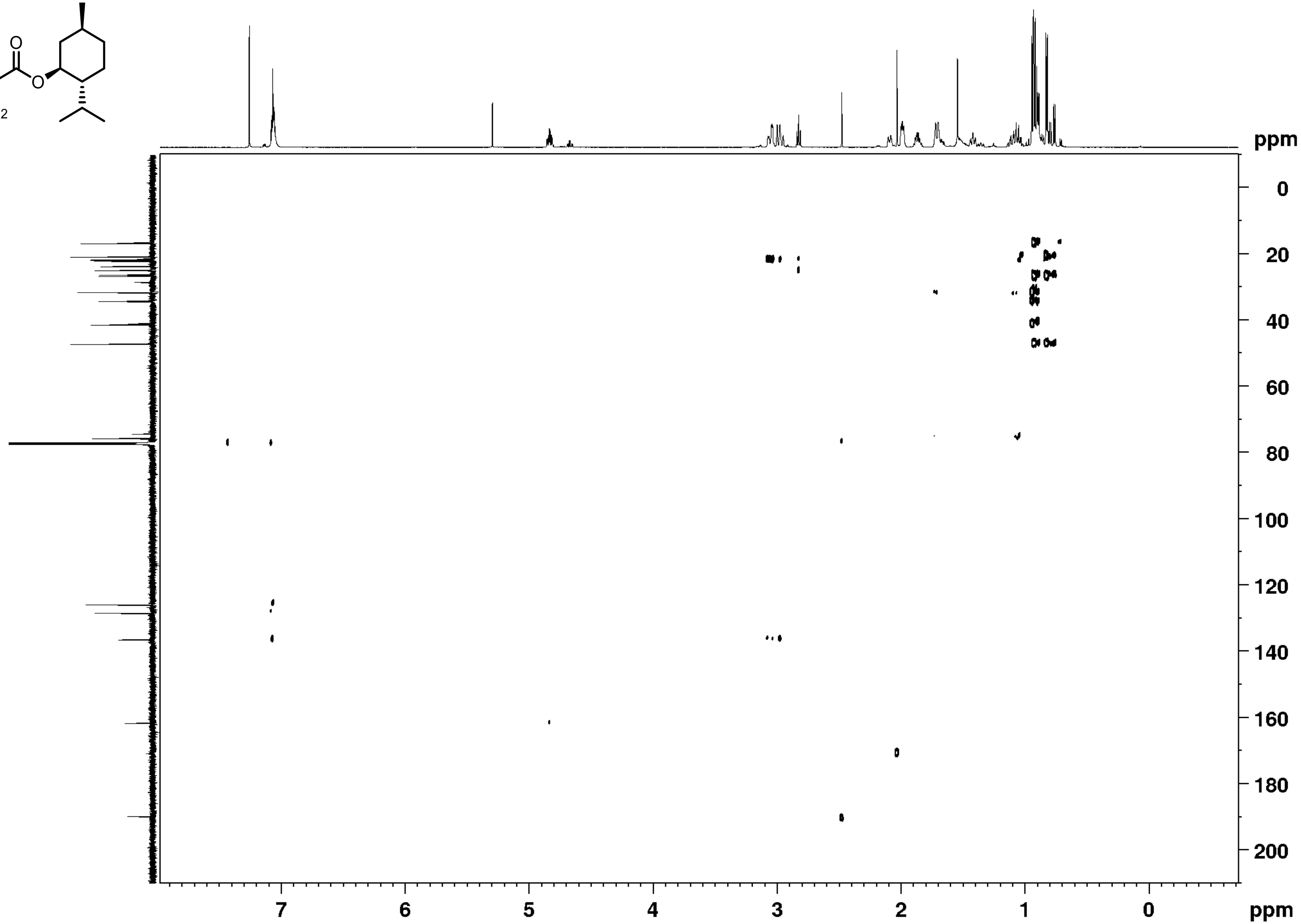

Figure S69: ^{1}H, ^{13}C HMBC spectrum of (D)-**11**.

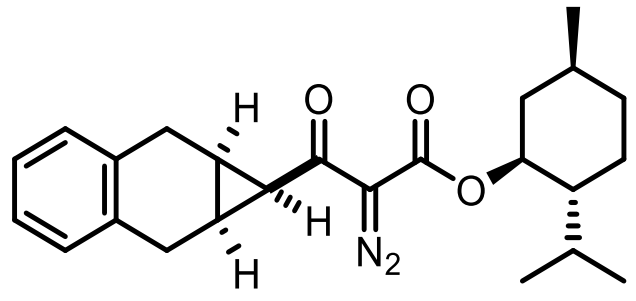

(D)-**11**

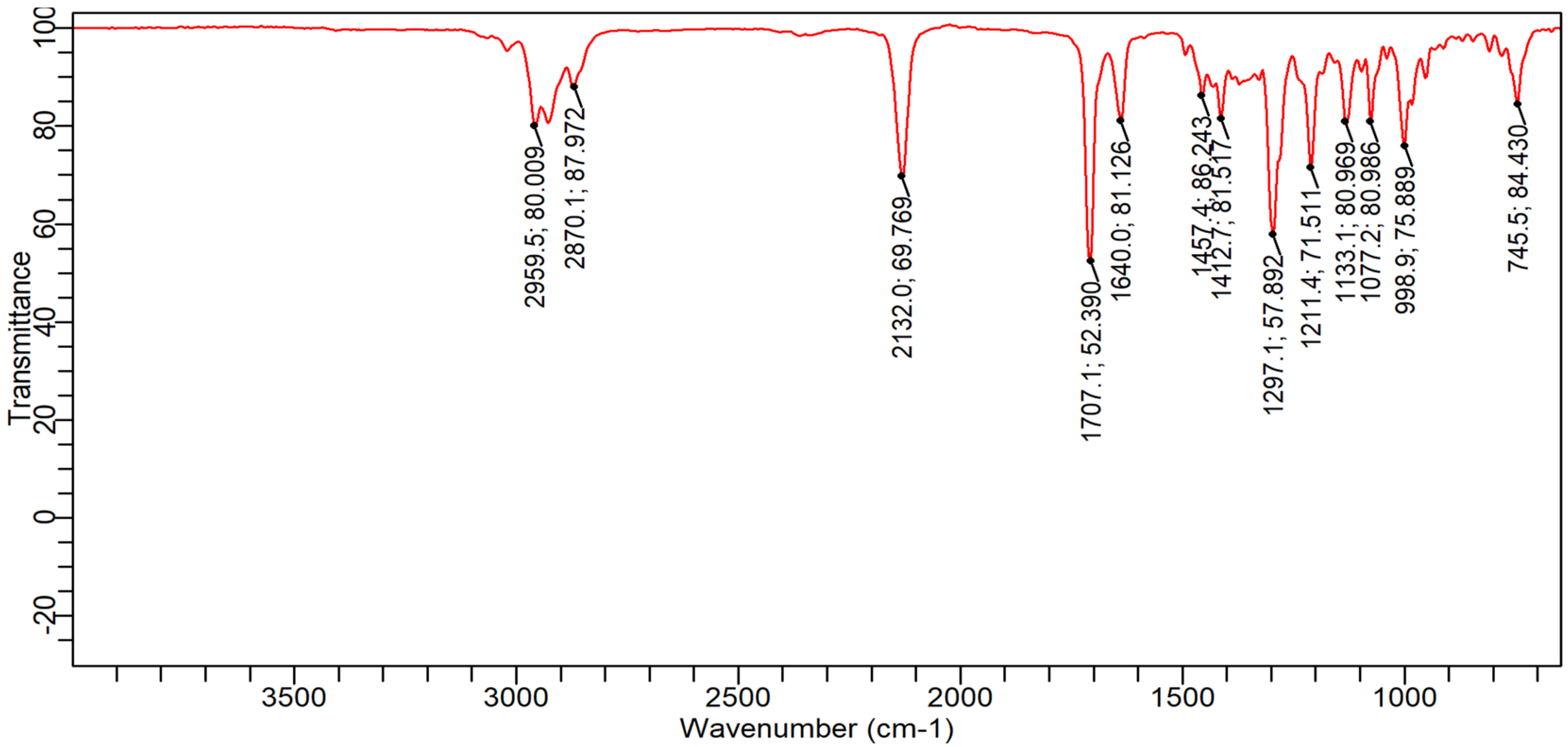

Figure S70: IR spectrum of (D)-**11**.

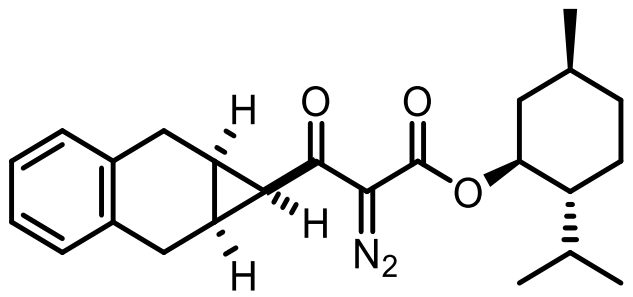

(D)-**11**

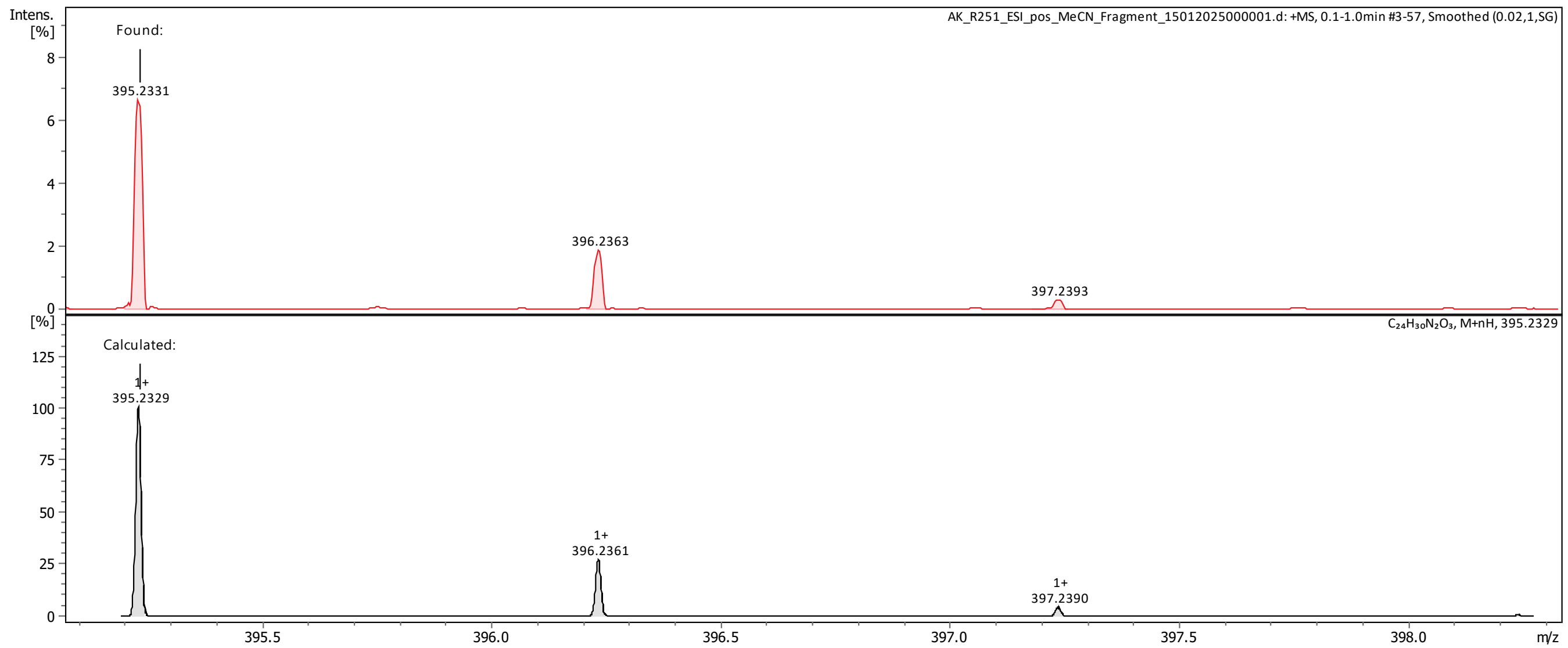

Figure S71: HRMS spectrum of (D)-**11**.

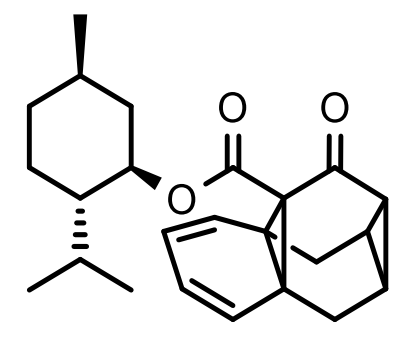

(L)-**12**

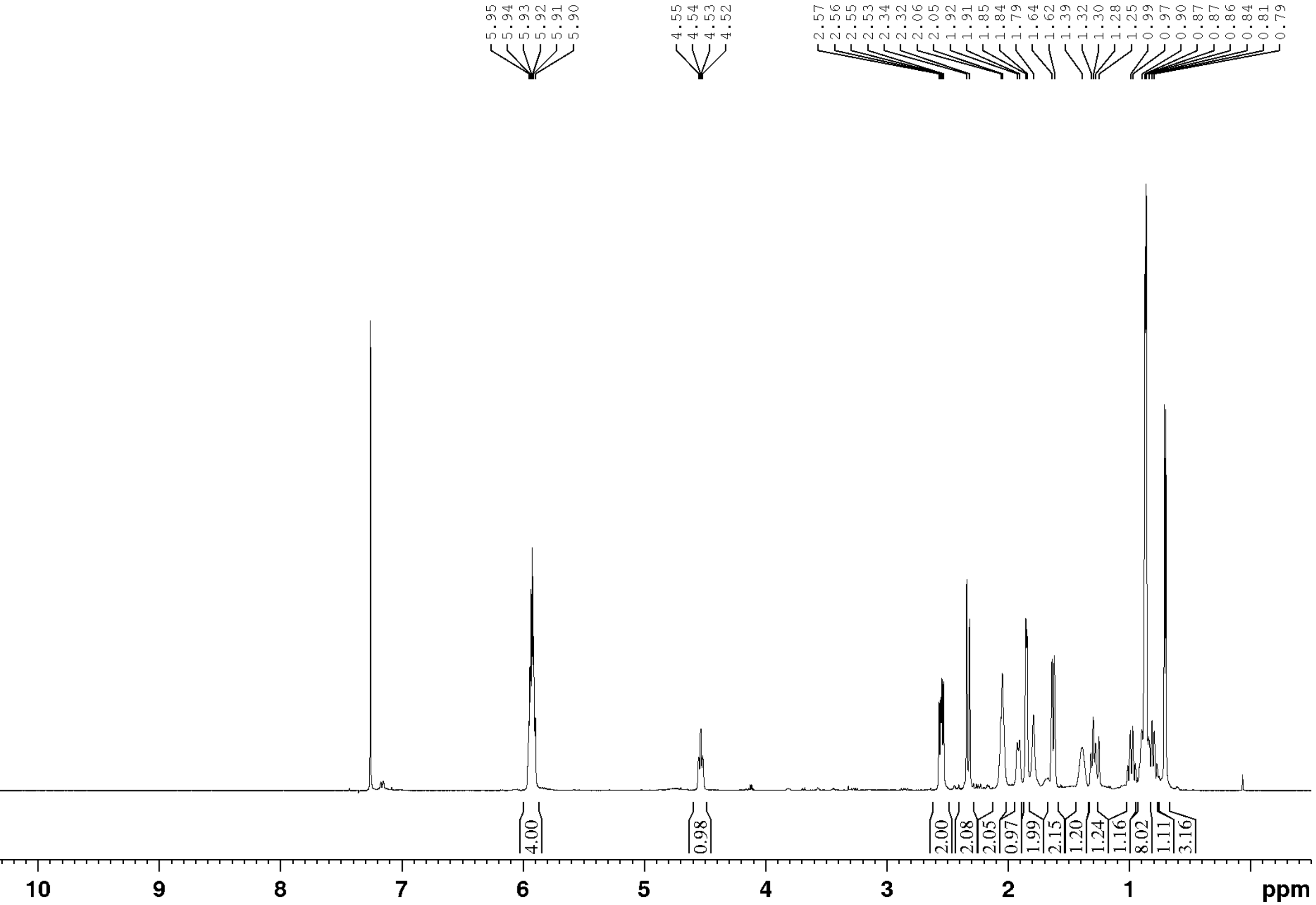

Figure S72: ^{1}H NMR spectrum of (L)-**12**.

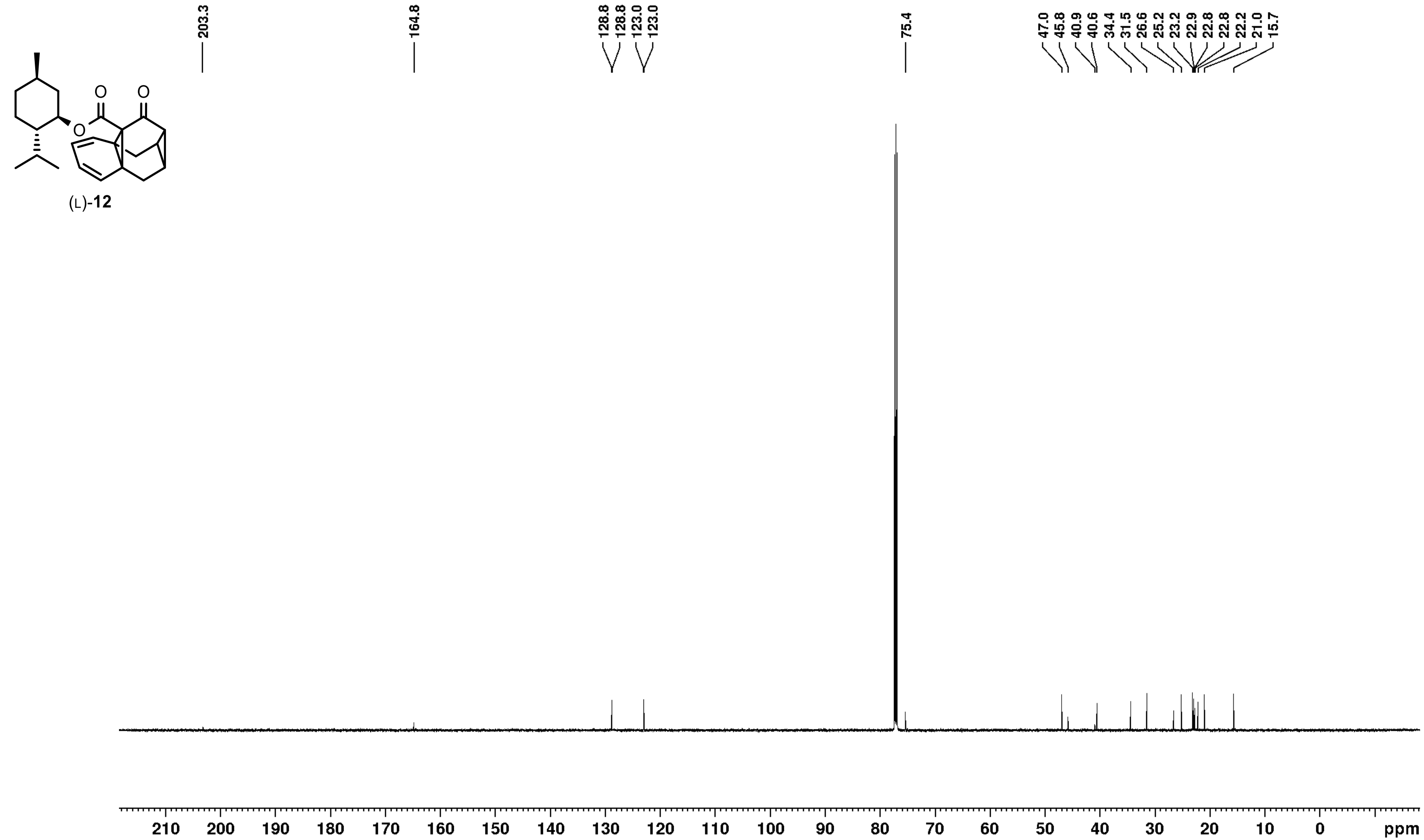

Figure S73: ^{13}C NMR spectrum of (L)-**12**.

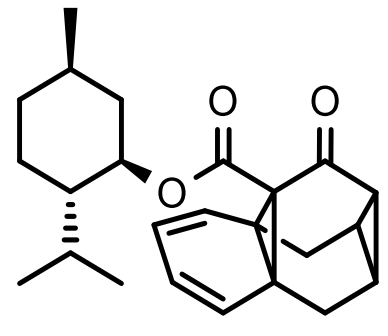

(L)-12

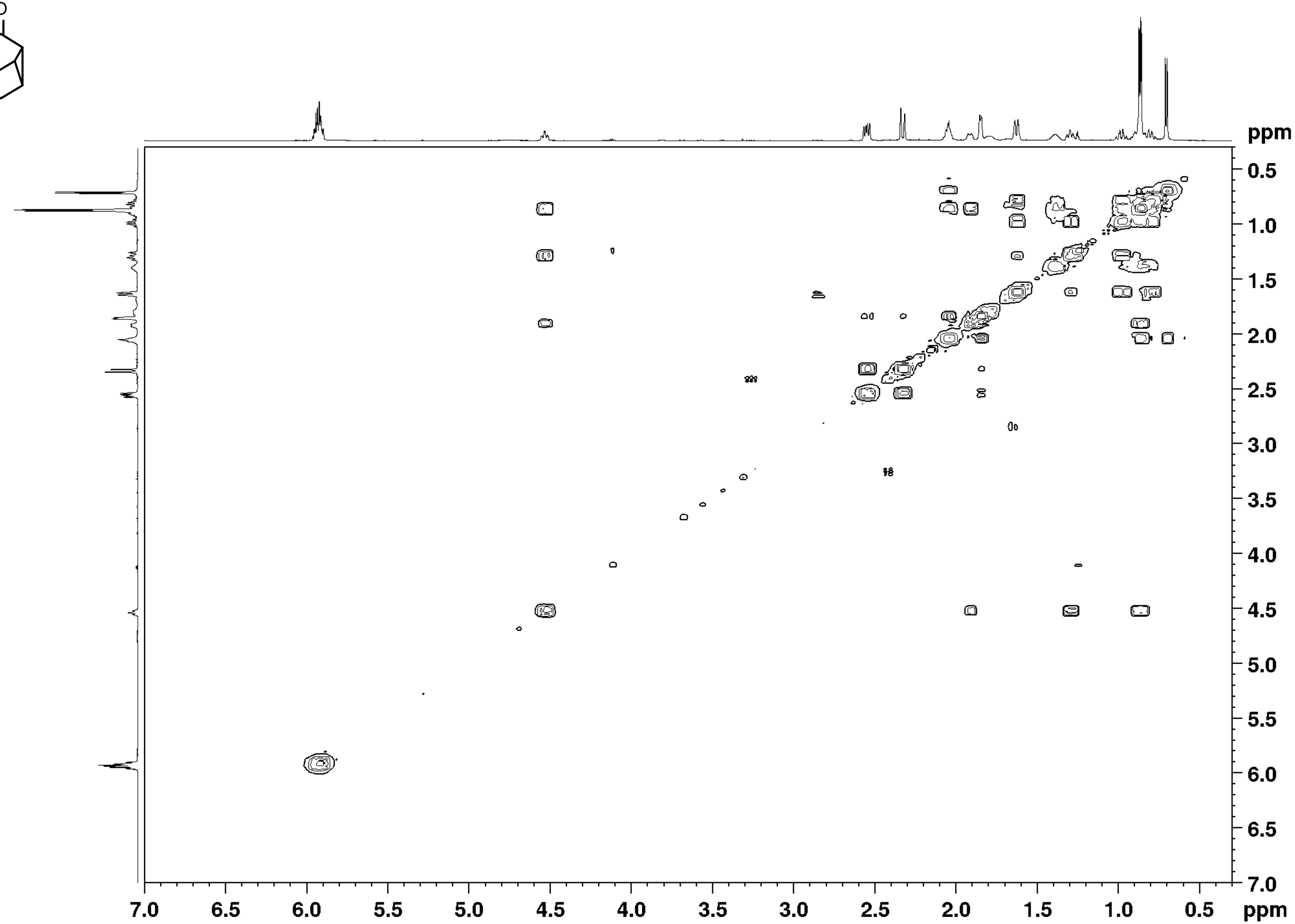

Figure S74: ^{1}H, ^{1}H COSY spectrum of (L)-**12**.

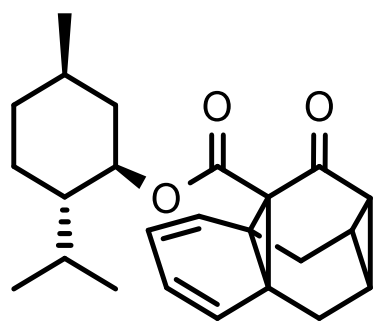

(L)-**12**

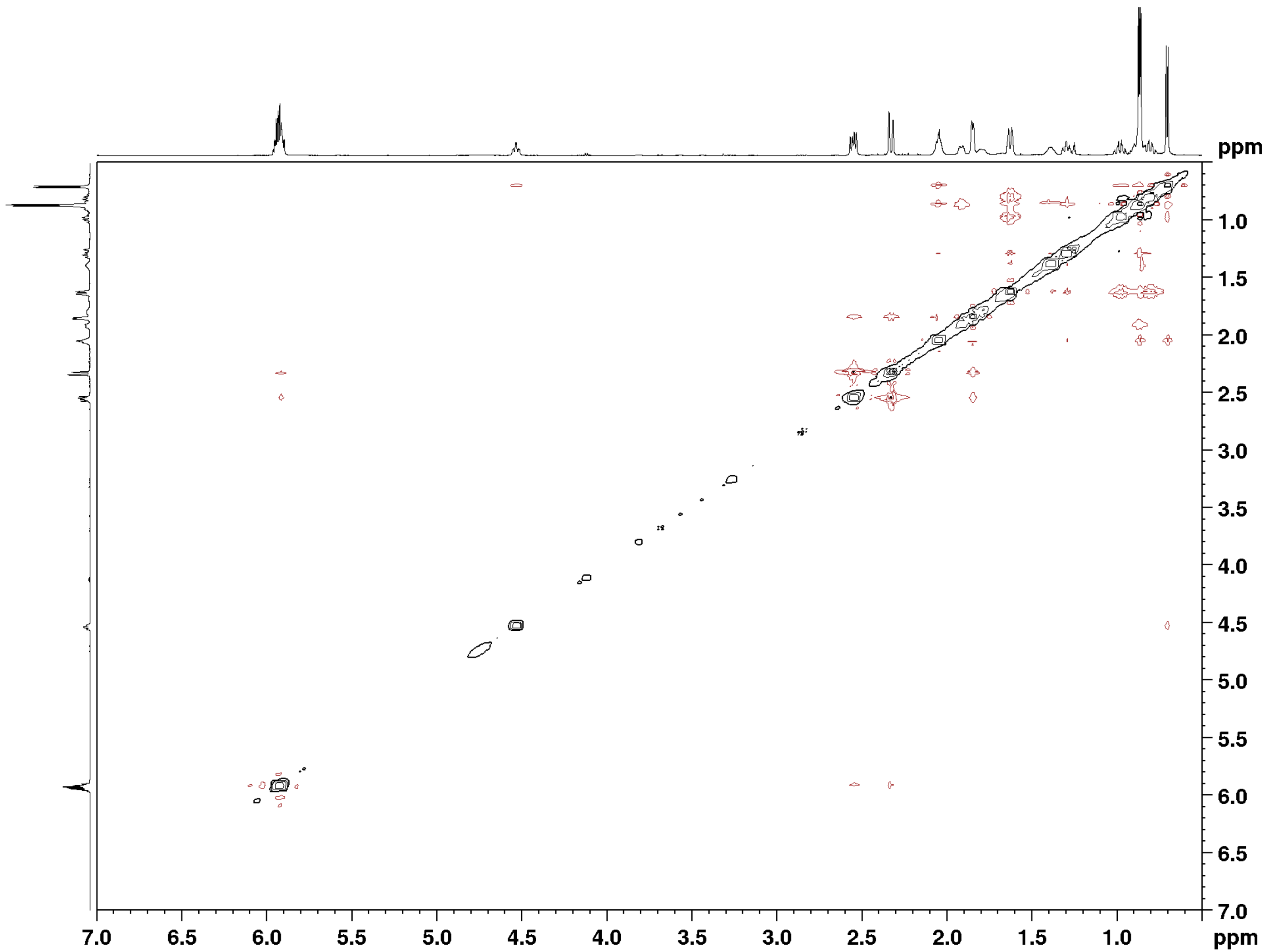

Figure S75: ^1H, ^1H NOESY spectrum of (L)-**12**.

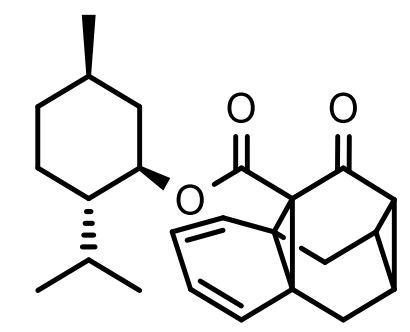

(L)-**12**

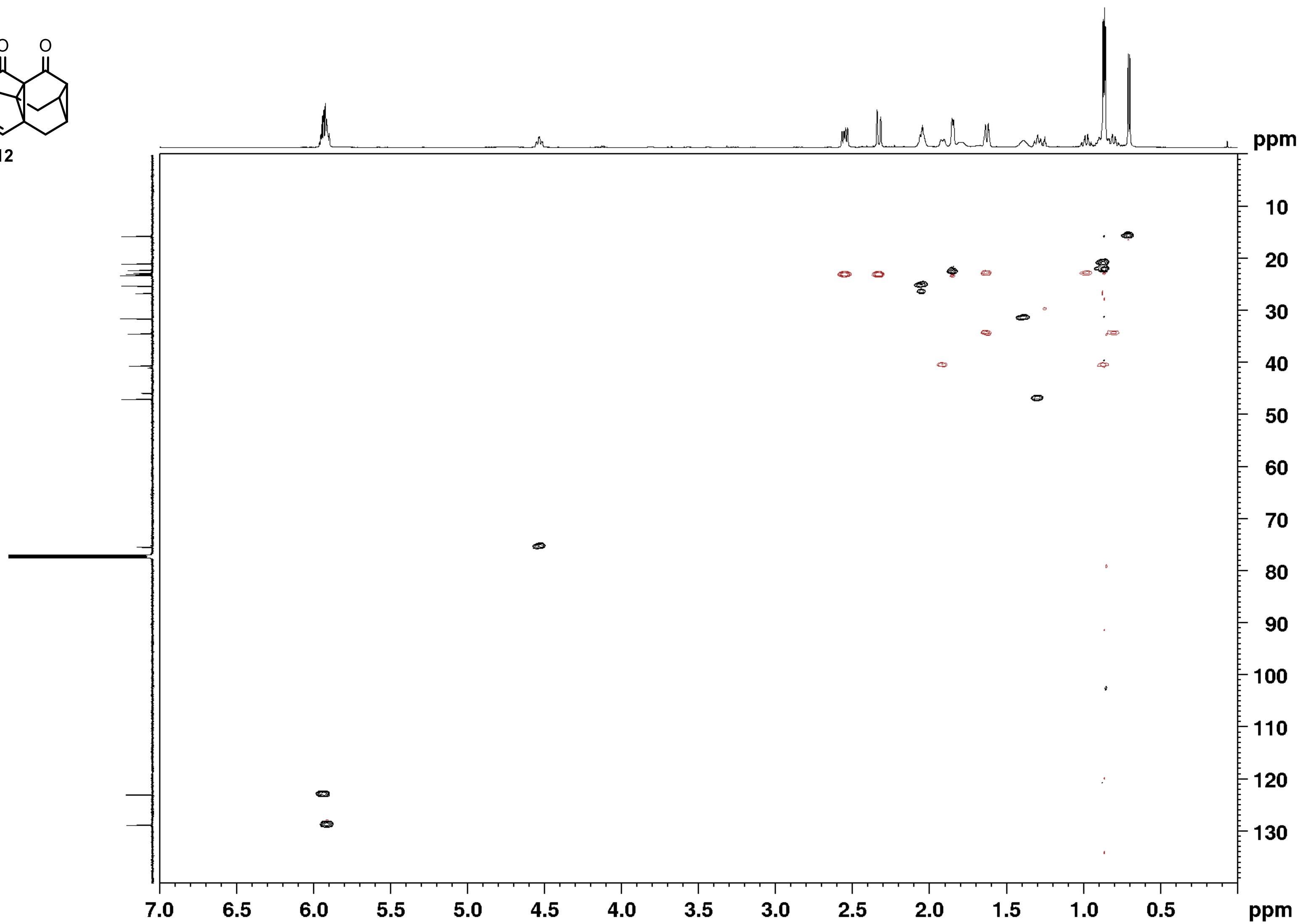

Figure S76: ^{1}H, ^{13}C HSQC spectrum of (L)-**12**.

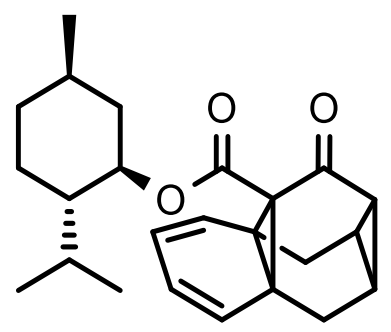

(L)-**12**

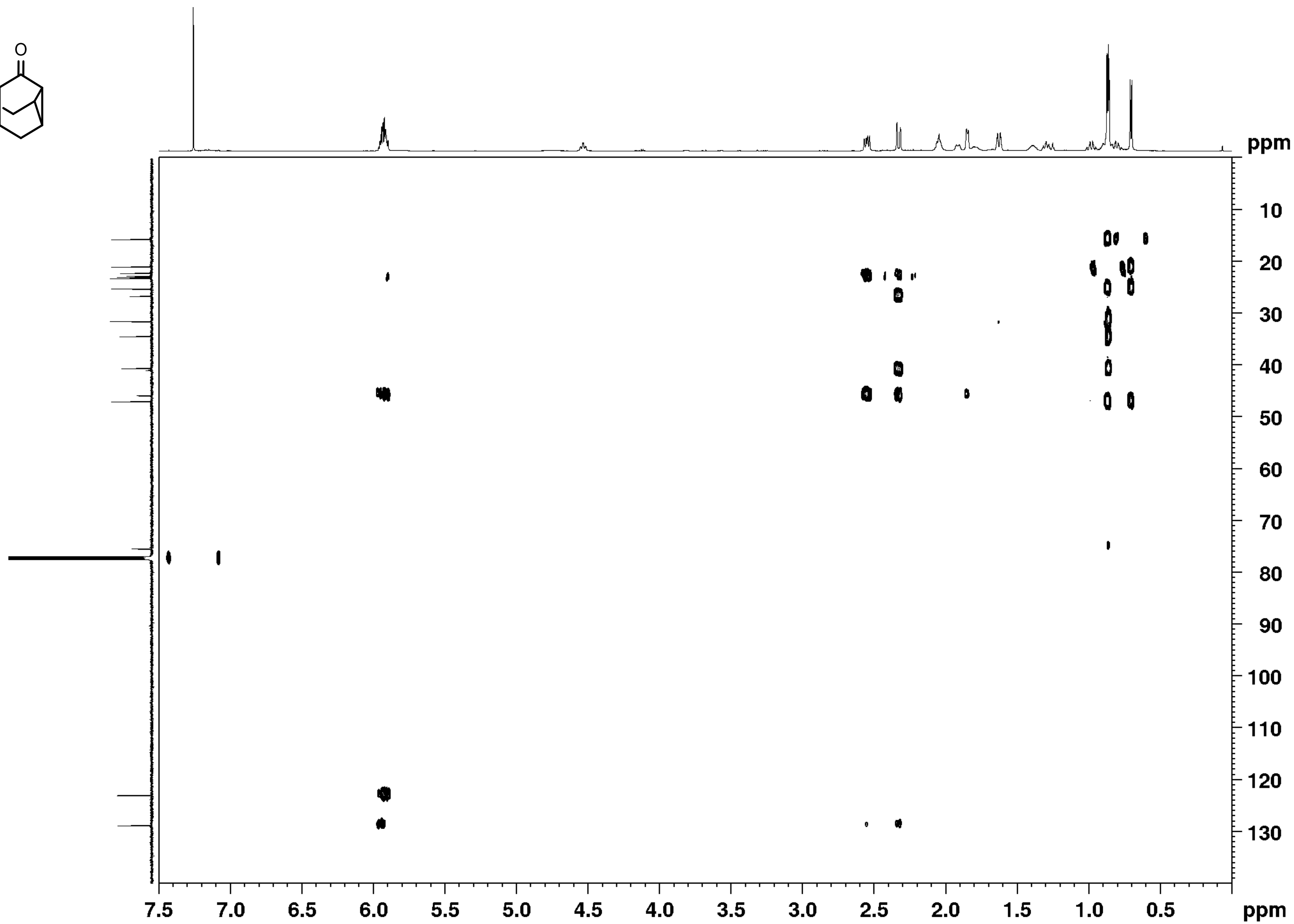

Figure S77: ^{1}H, ^{13}C HMBC spectrum of (L)-**12**.

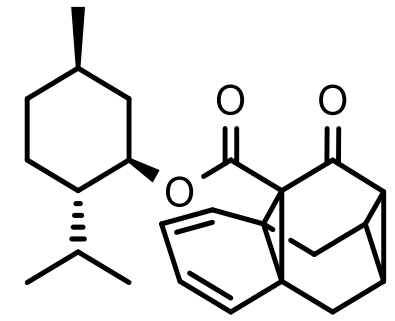

(L)-**12**

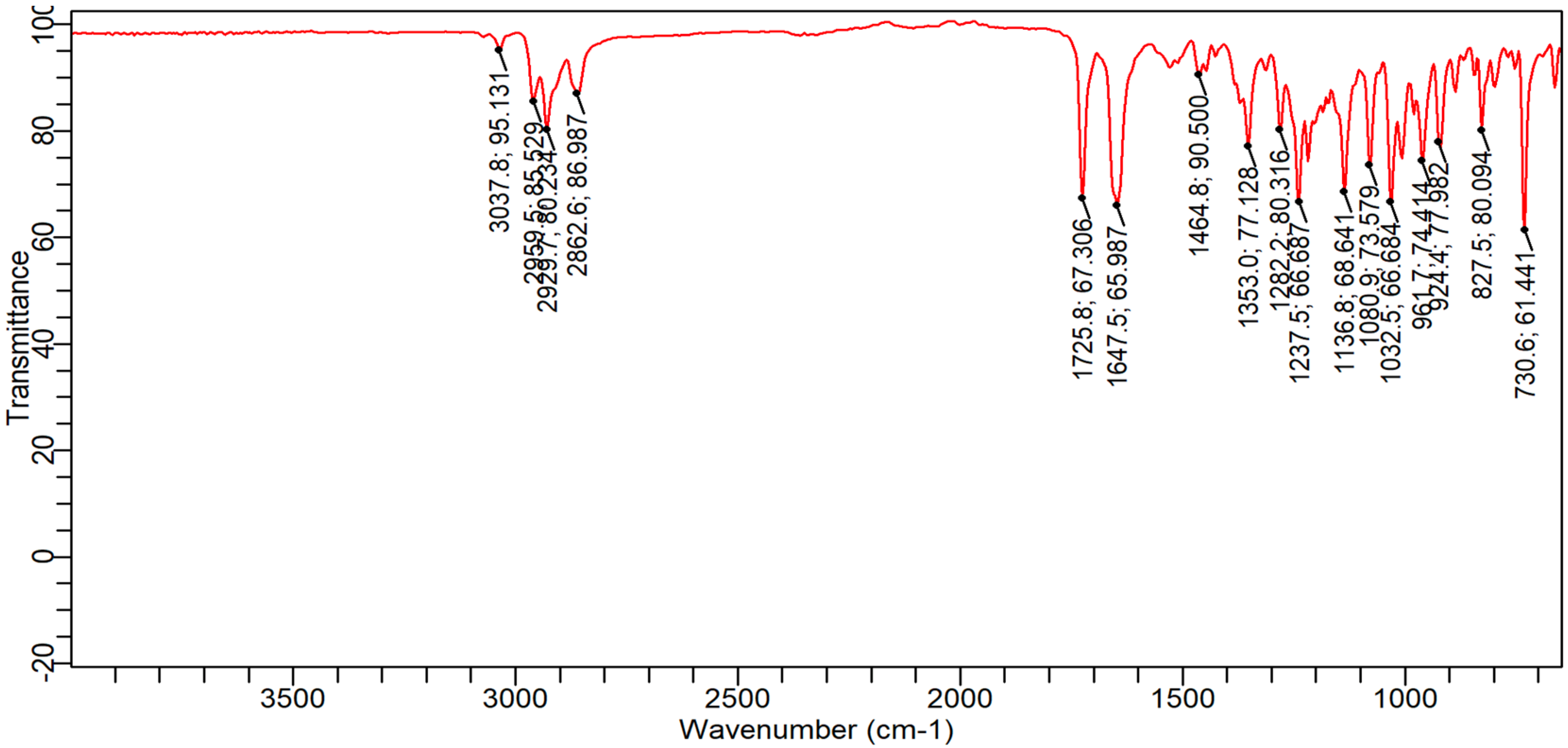

Figure S78: IR spectrum of (L)-**12**.

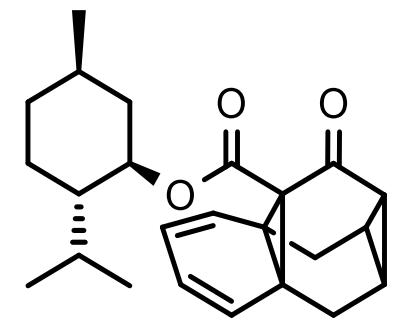

(L)-**12**

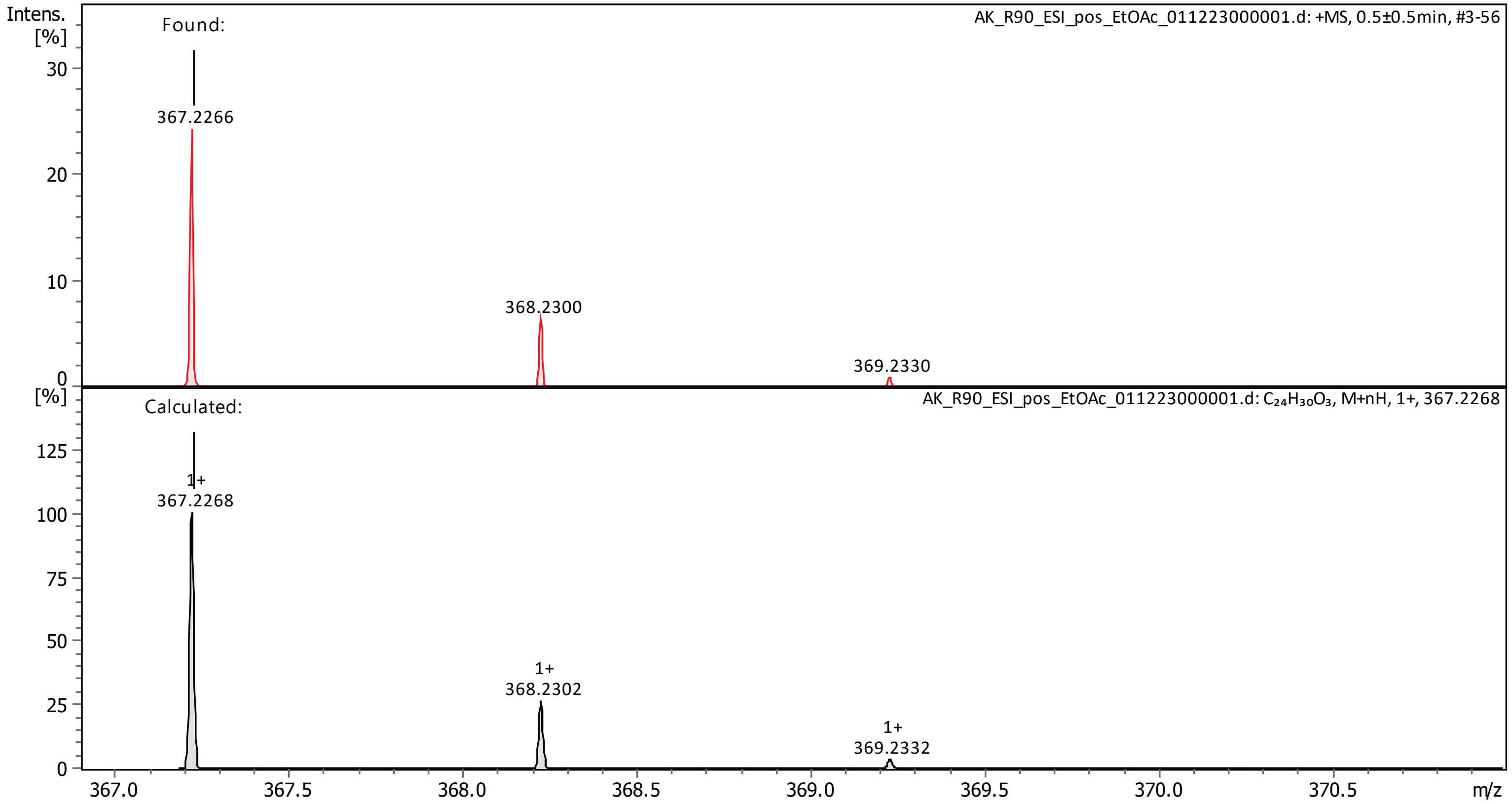

Figure S79: HRMS spectrum of (L)-**12**.

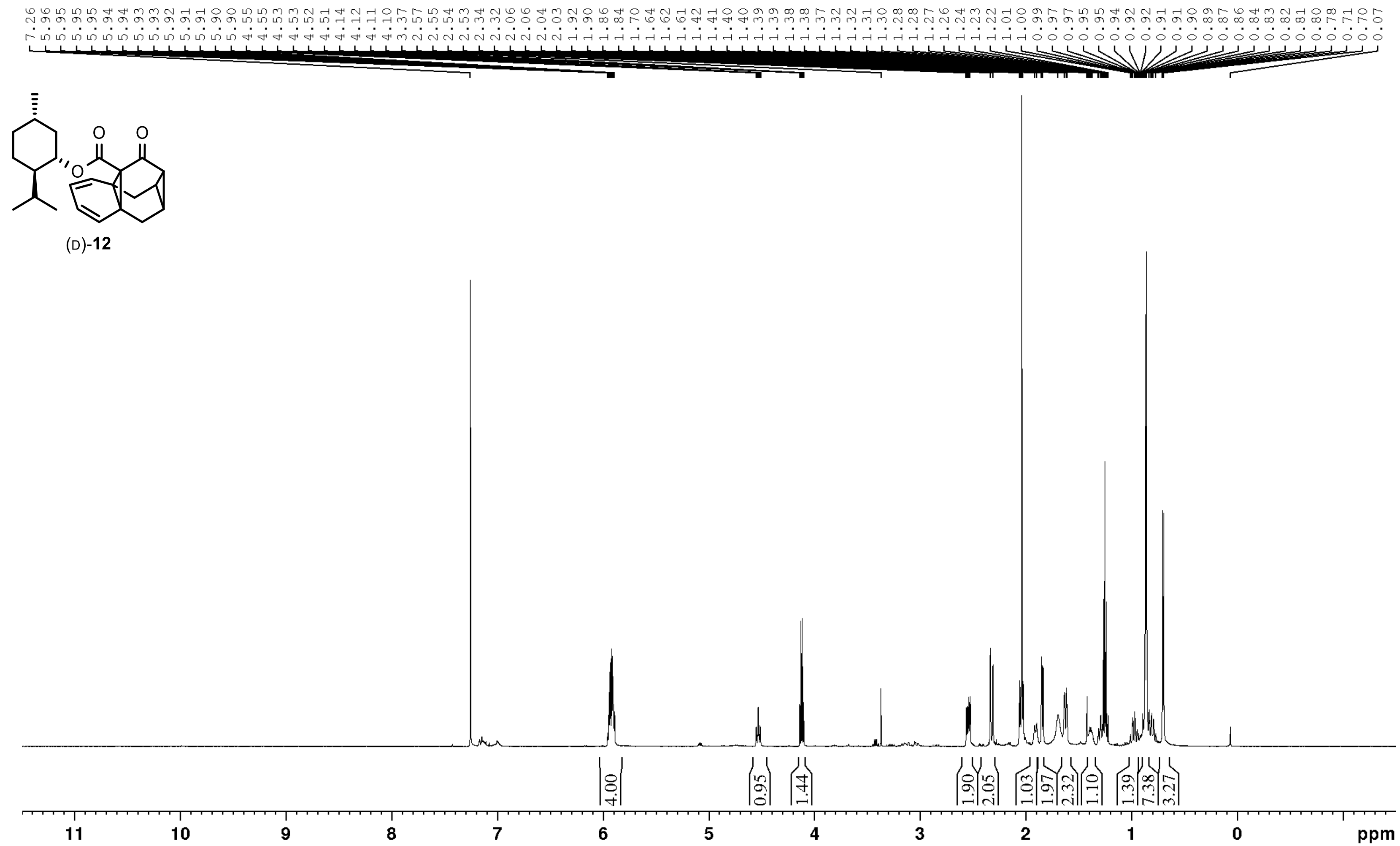

Figure S80: ^{1}H NMR spectrum of (D)-**12**.

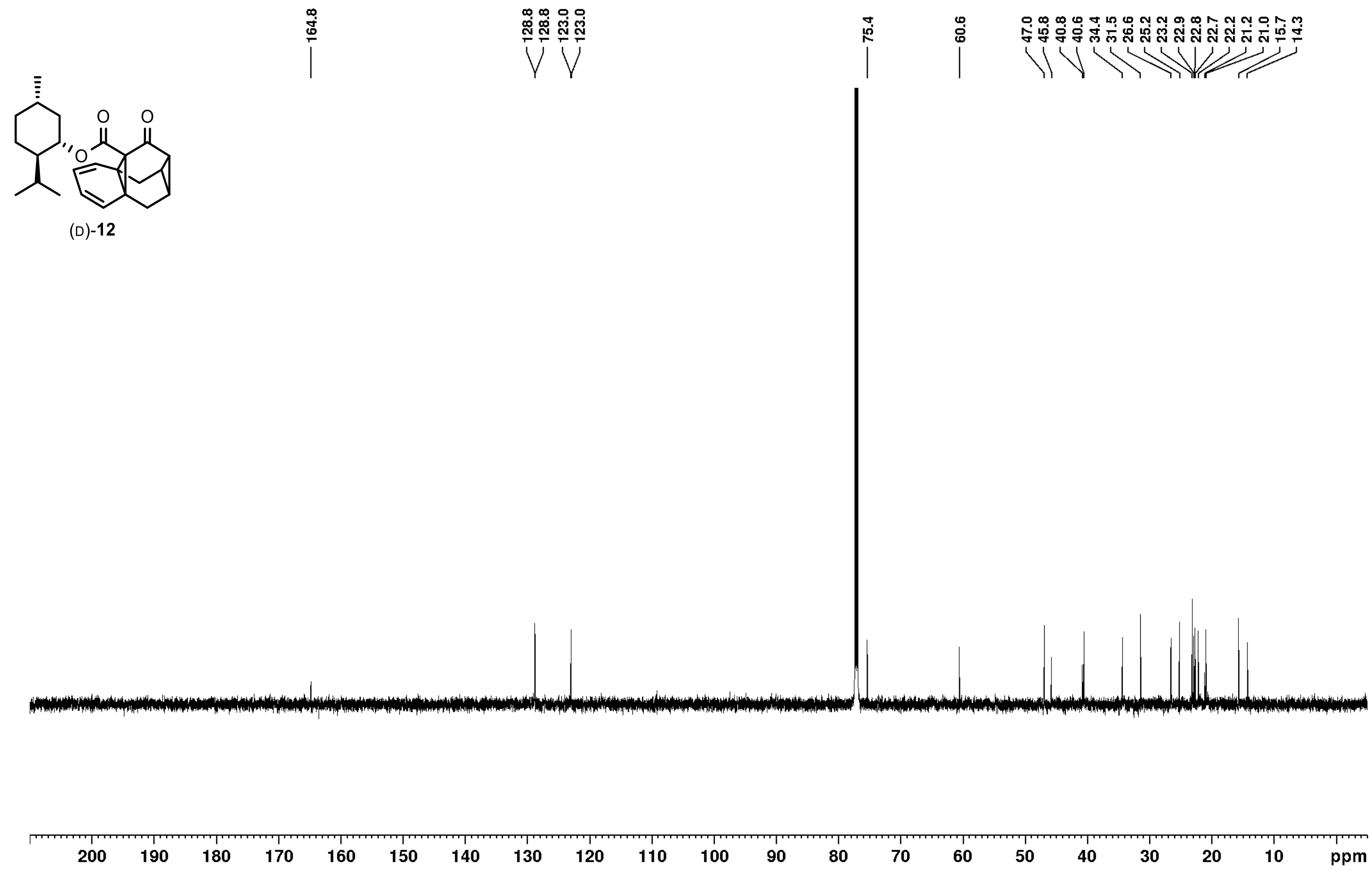

Figure S81: ^{13}C NMR spectrum of (D)-**12**.

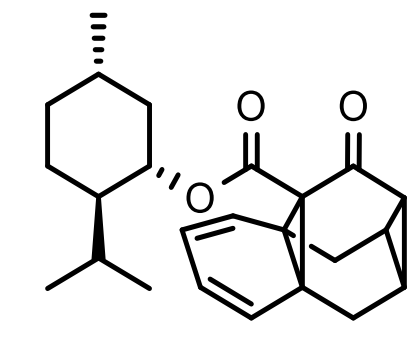

(D)-**12**

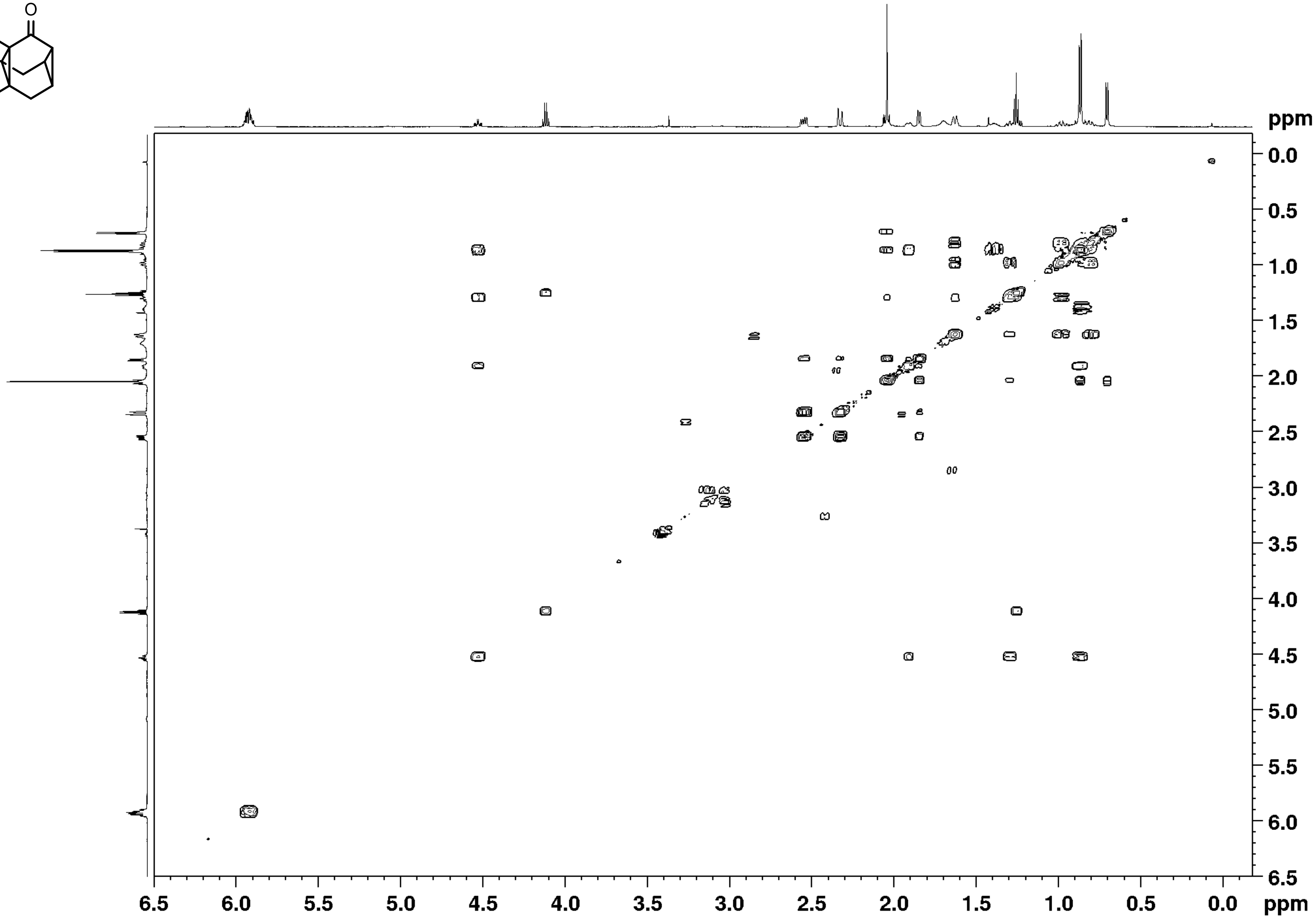

Figure S82: ^{1}H, ^{1}H COSY spectrum of (D)-**12**.

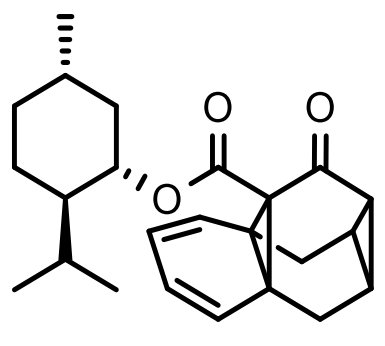

(D)-12

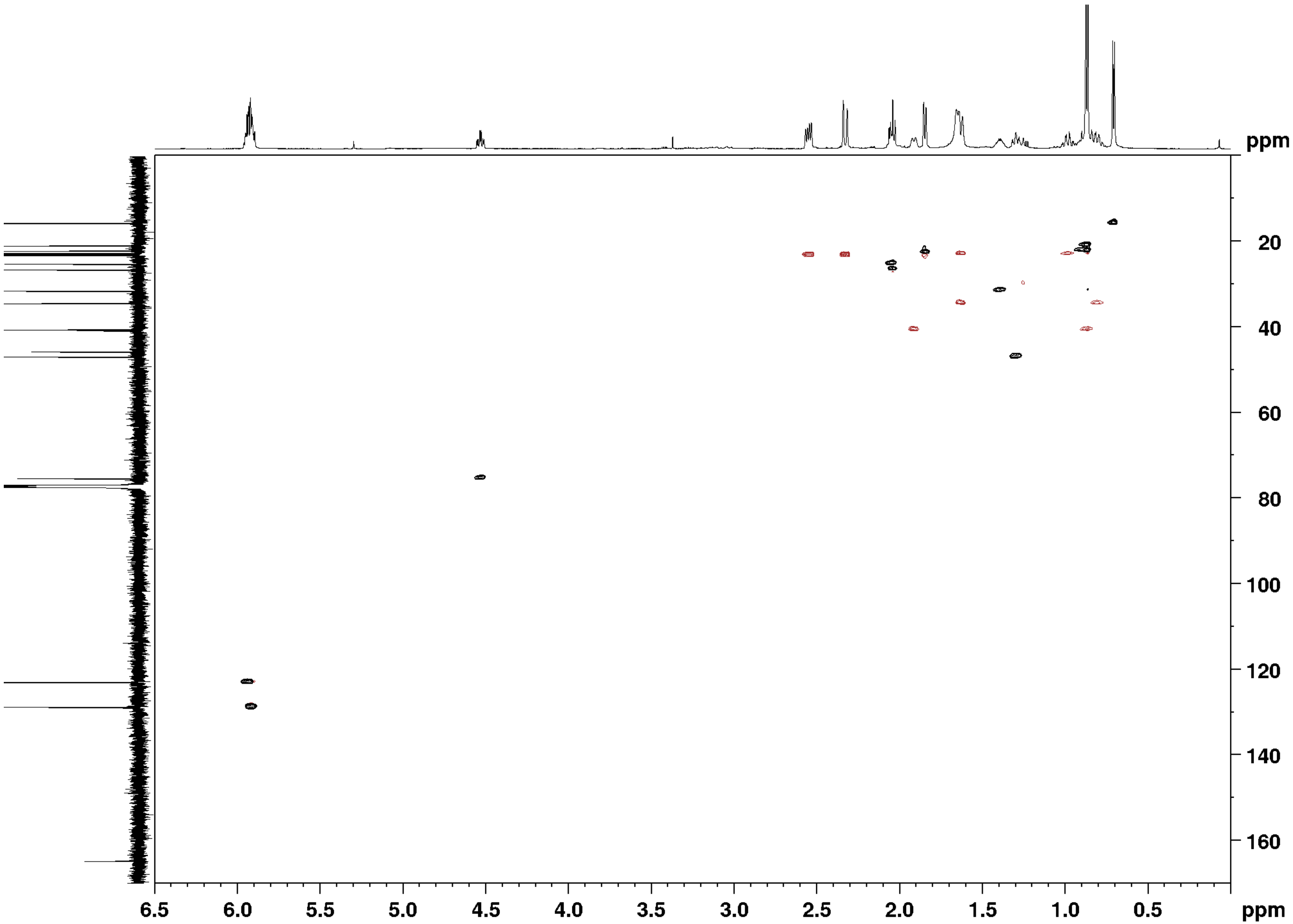

Figure S83: ^{1}H, ^{13}C HSQC spectrum of (D)-**12**.

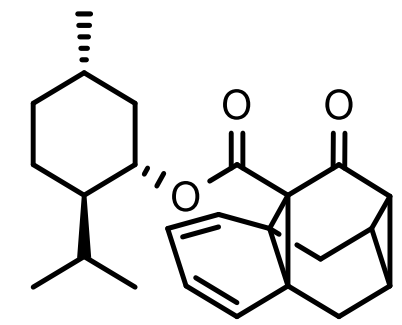

(D)-**12**

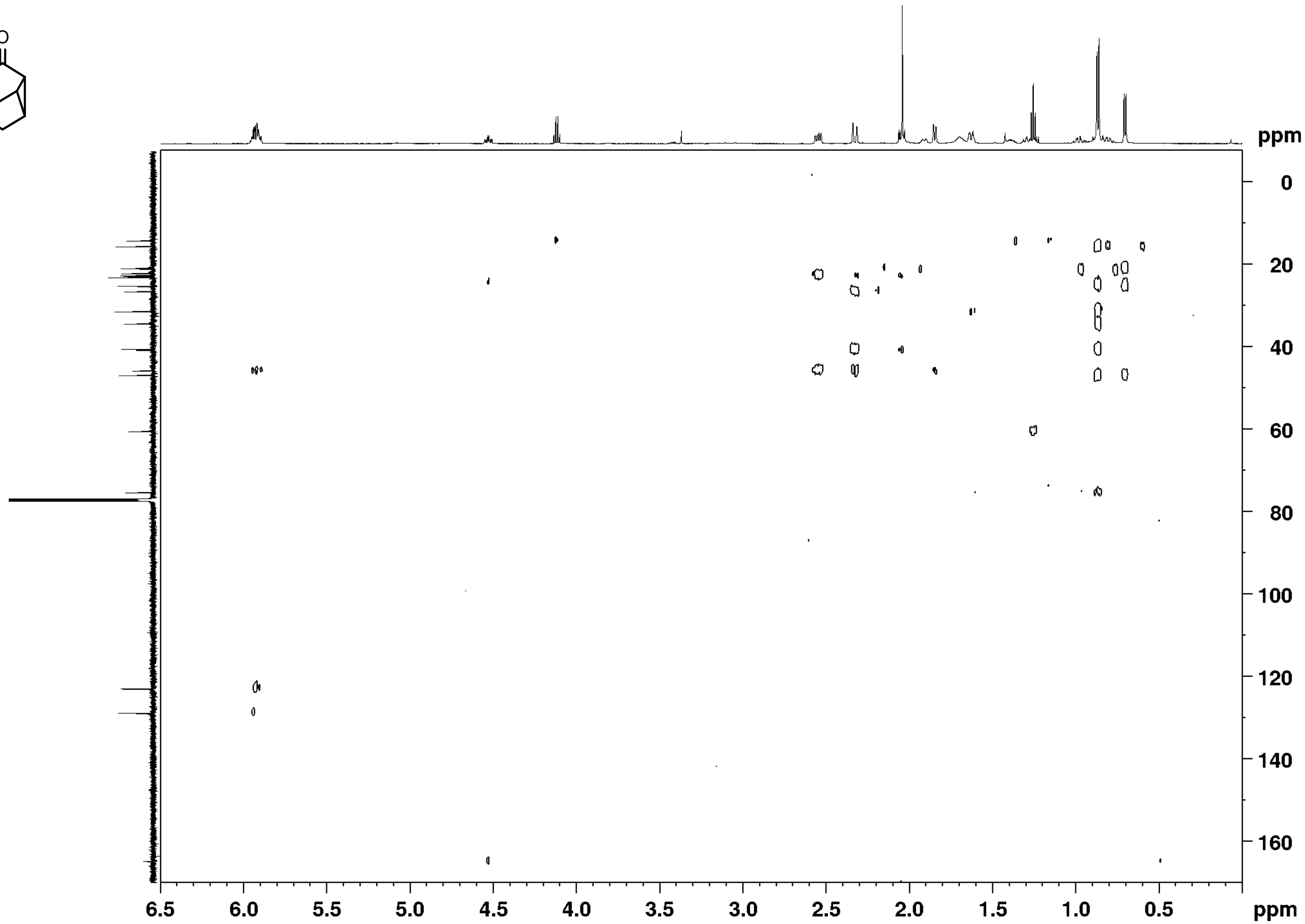

Figure S84: ^{1}H, ^{13}C HMBC spectrum of (D)-**12**.

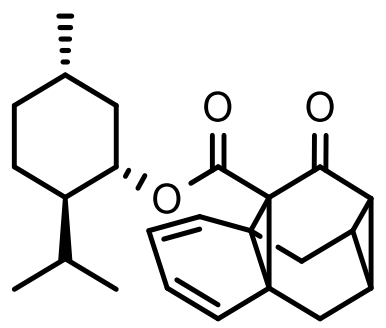

(D)-**12**

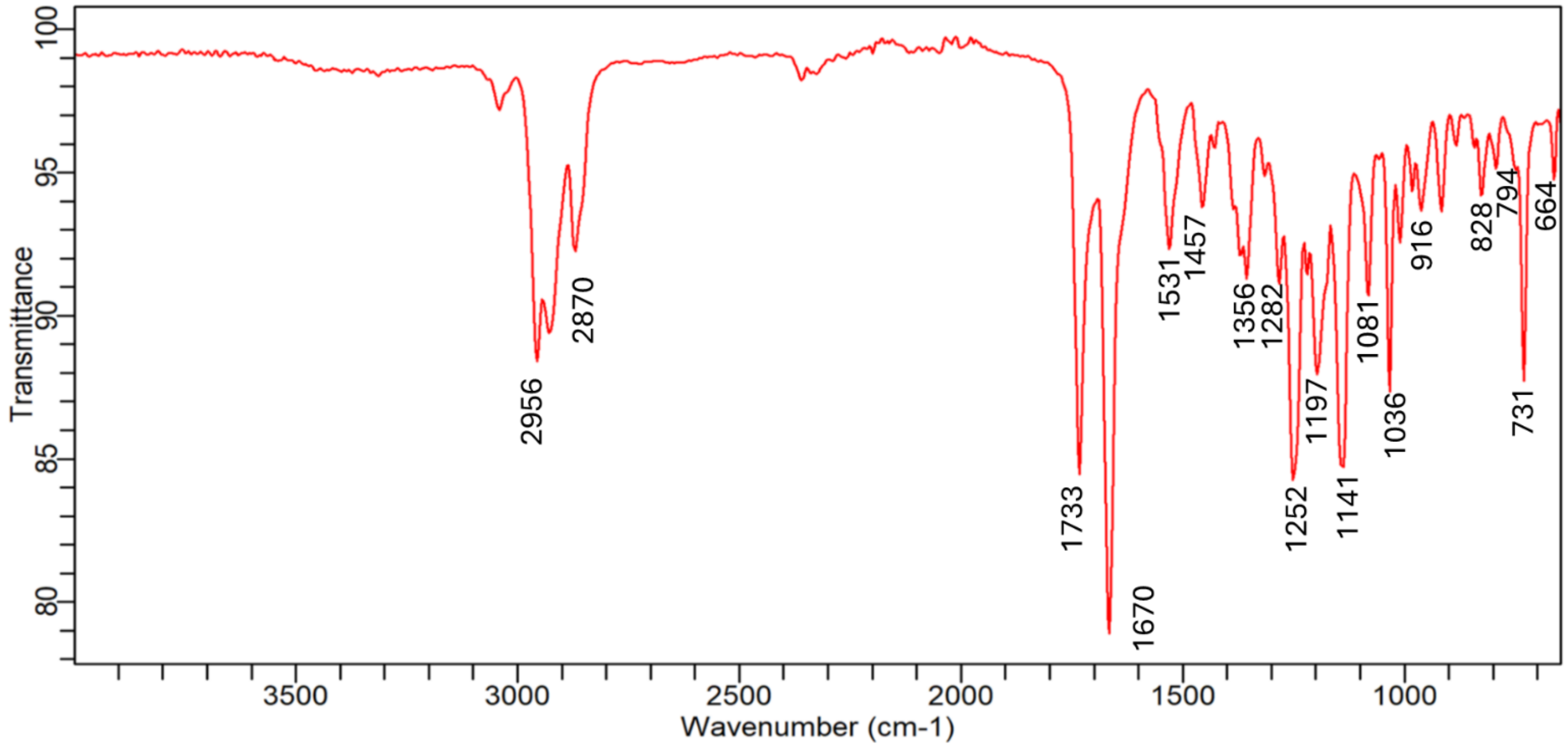

Figure S85: IR spectrum of (D)-**12**.

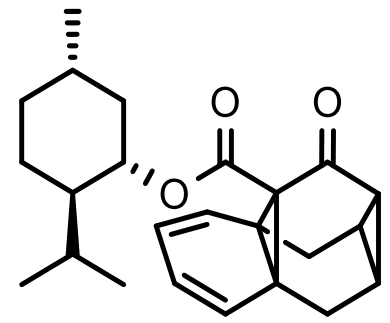

(D)-**12**

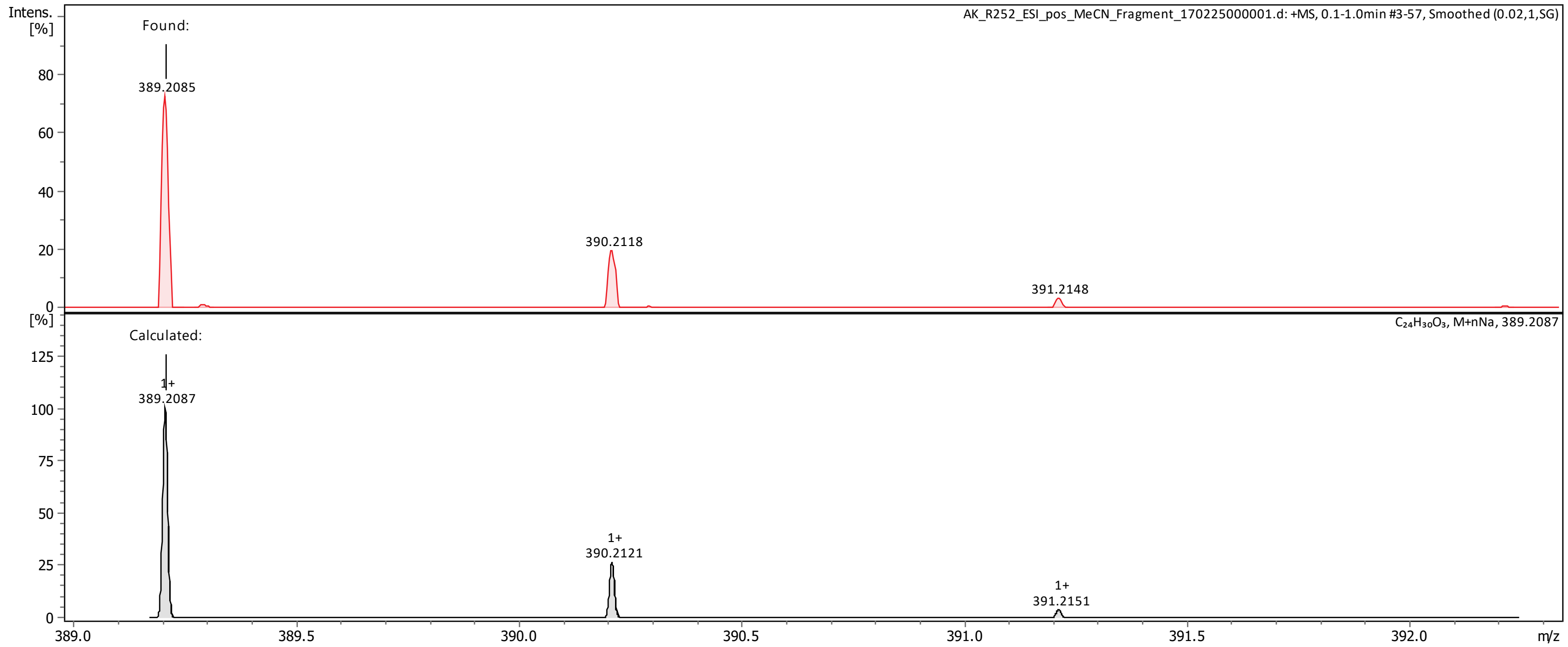

Figure S86: HRMS spectrum of (D)-**12**.

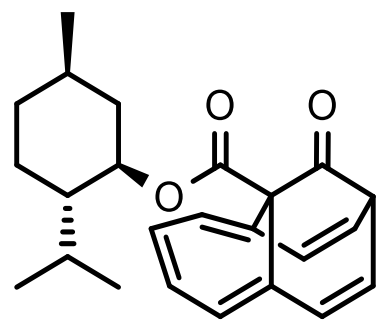

(L)-**6**

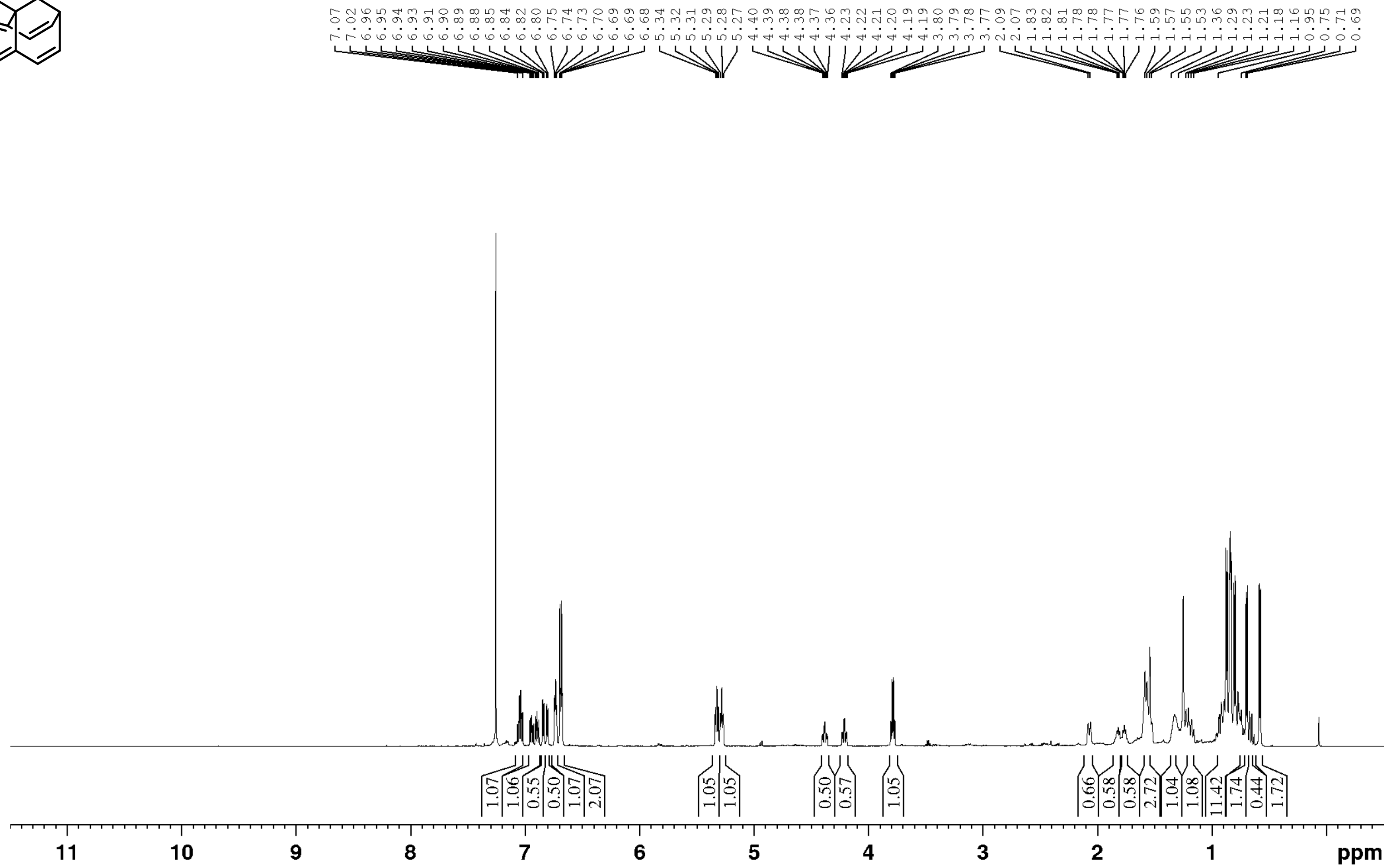

Figure S87: ^1H NMR spectrum of (L)-**6**.

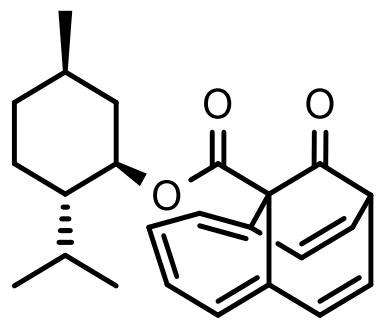
(L)-6

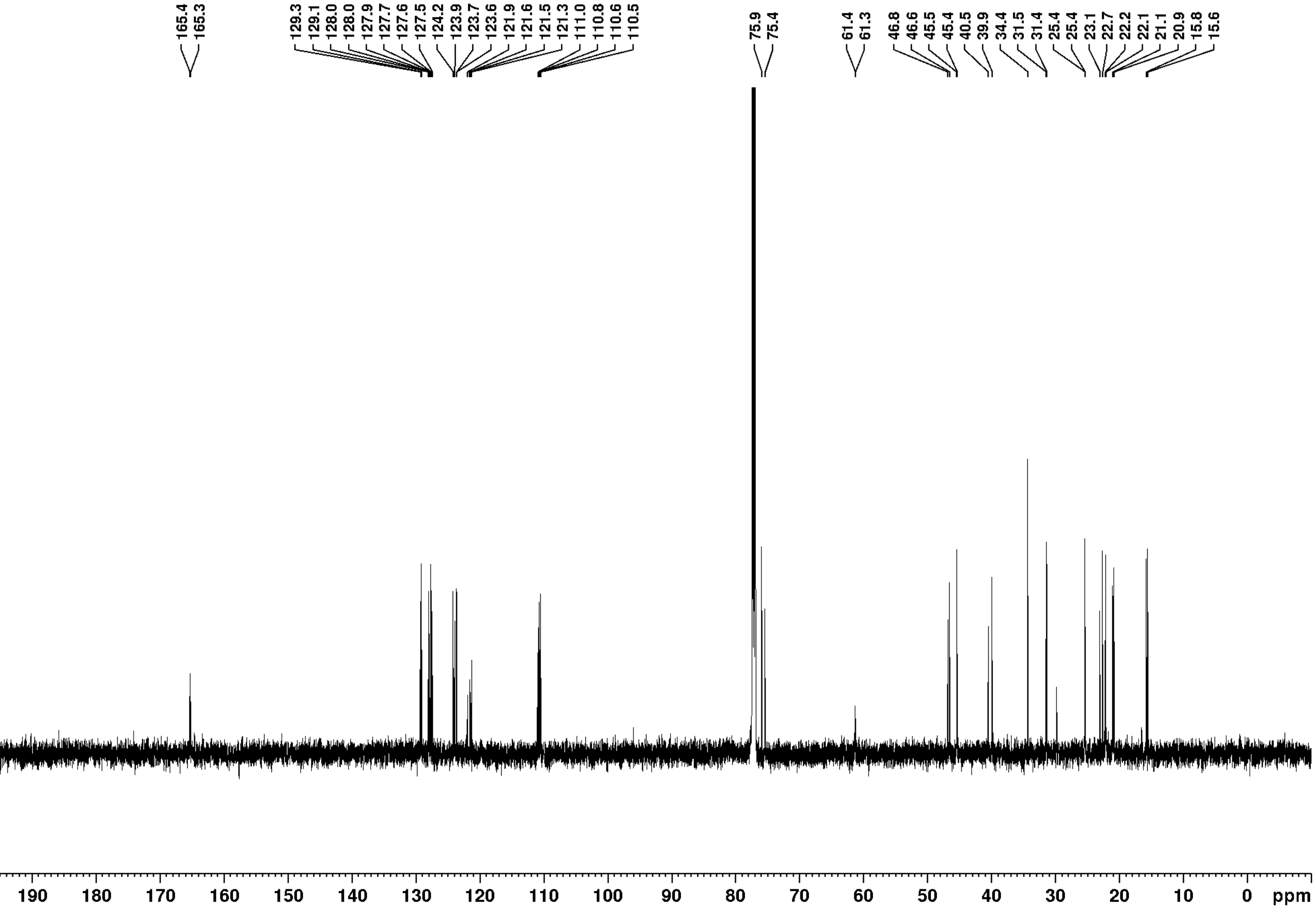

Figure S88: ^{13}C NMR spectrum of (L)-**6**.

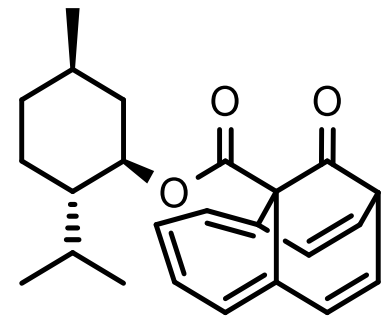

(L)-**6**

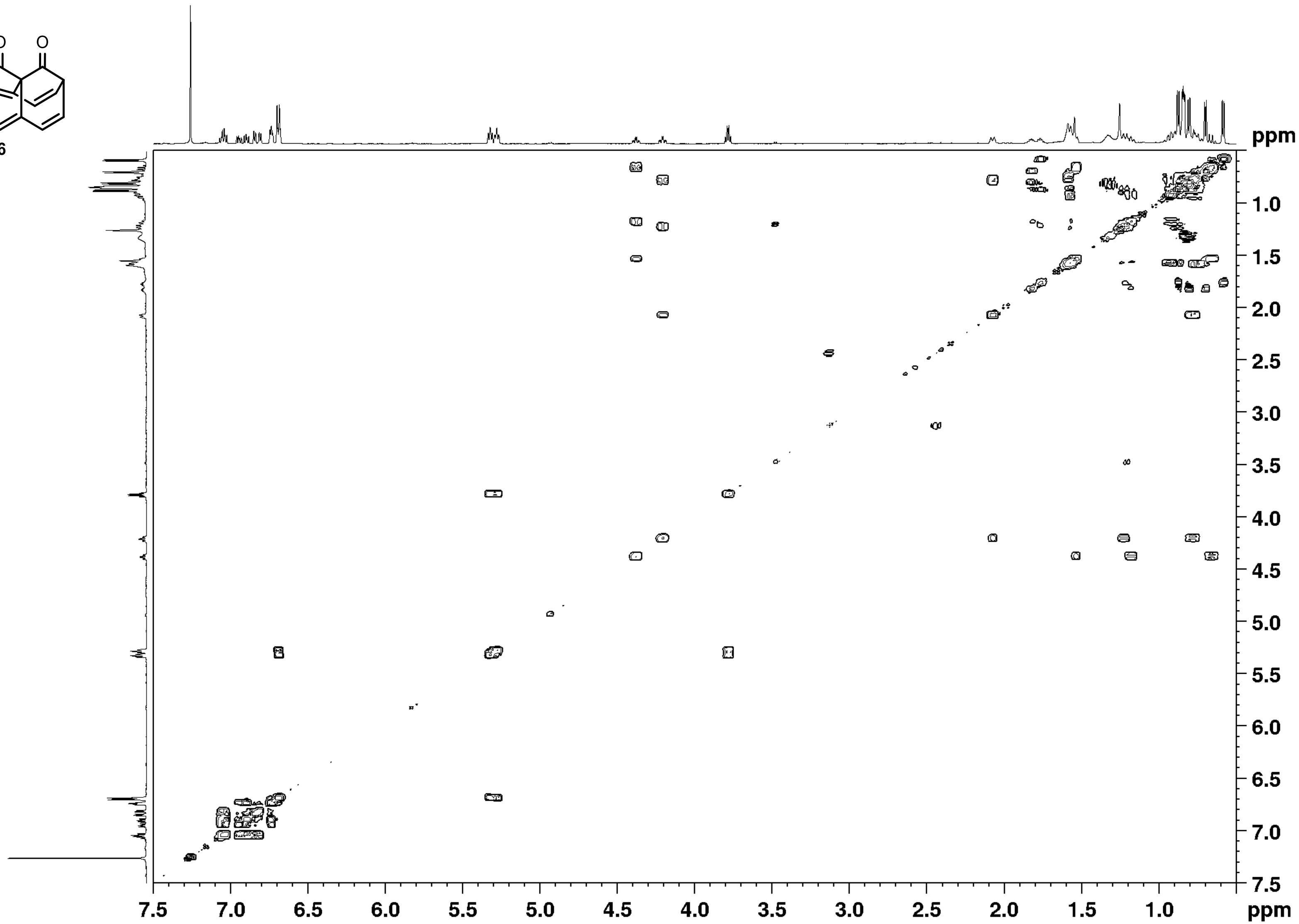

Figure S89: ^{1}H, ^{1}H COSY spectrum of (L)-**6**.

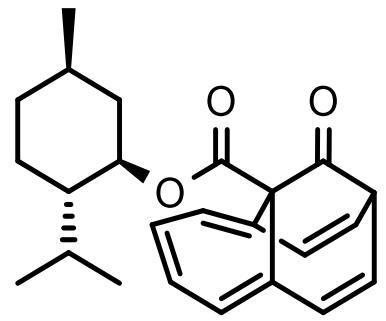

(L)-**6**

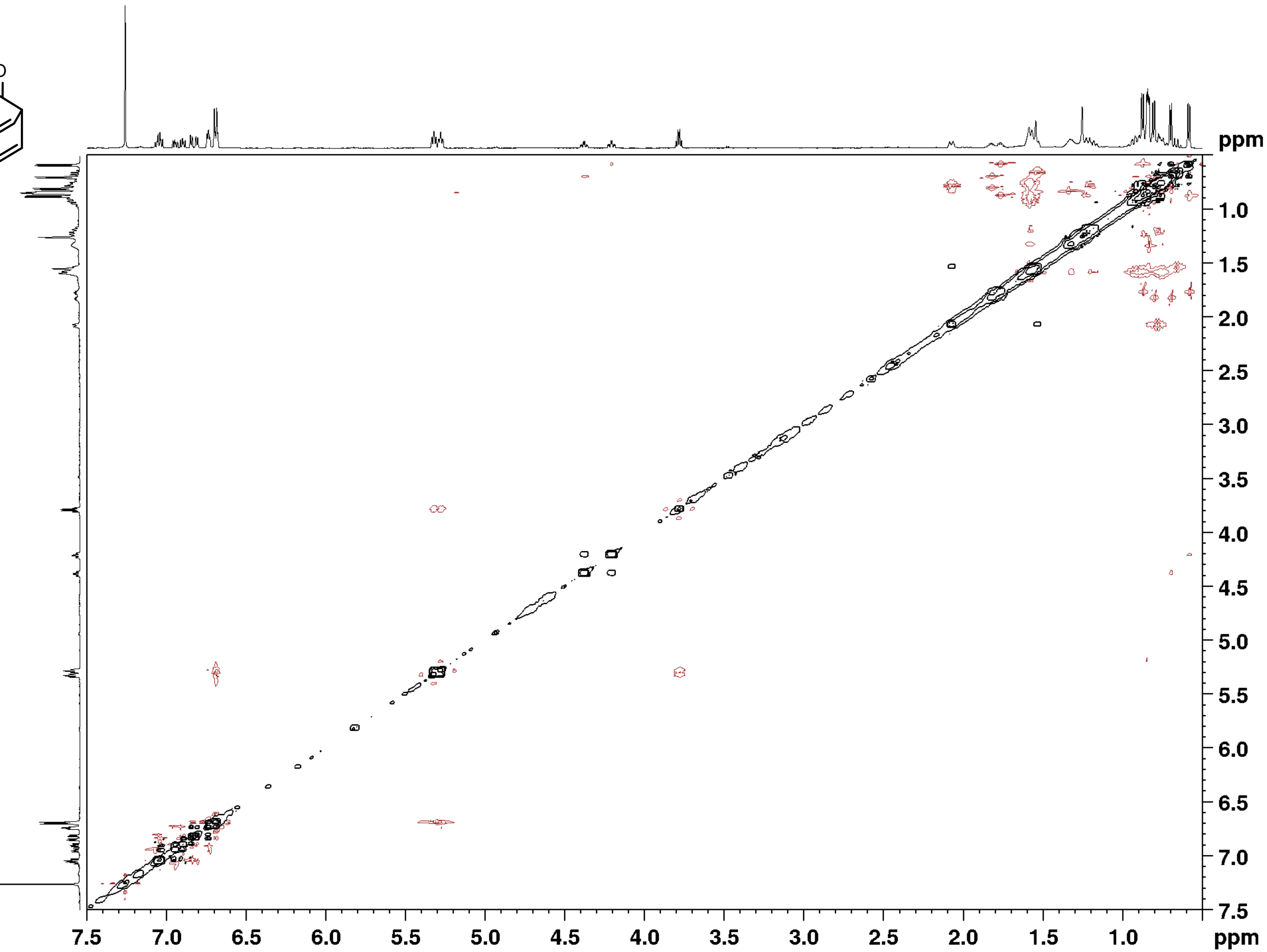

Figure S90: ^{1}H, ^{1}H NOESY spectrum of (L)-**6**.

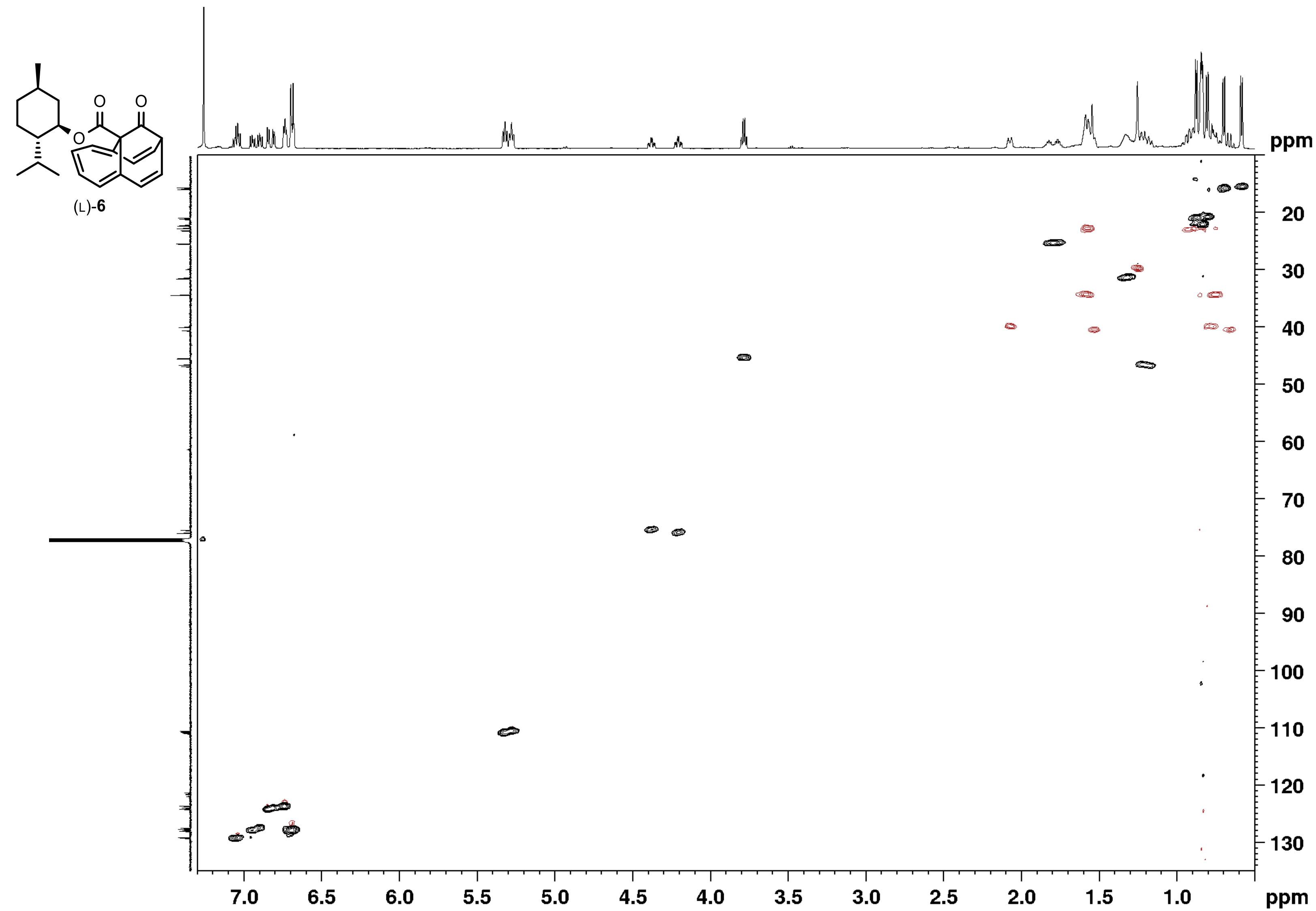

Figure S91: ^{1}H, ^{13}C HSQC spectrum of (L)-**6**.

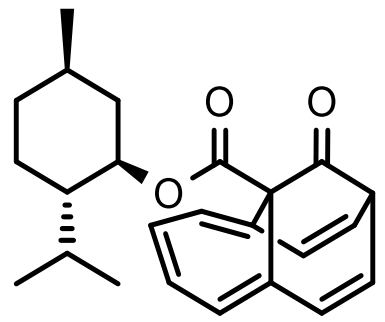

(L)-**6**

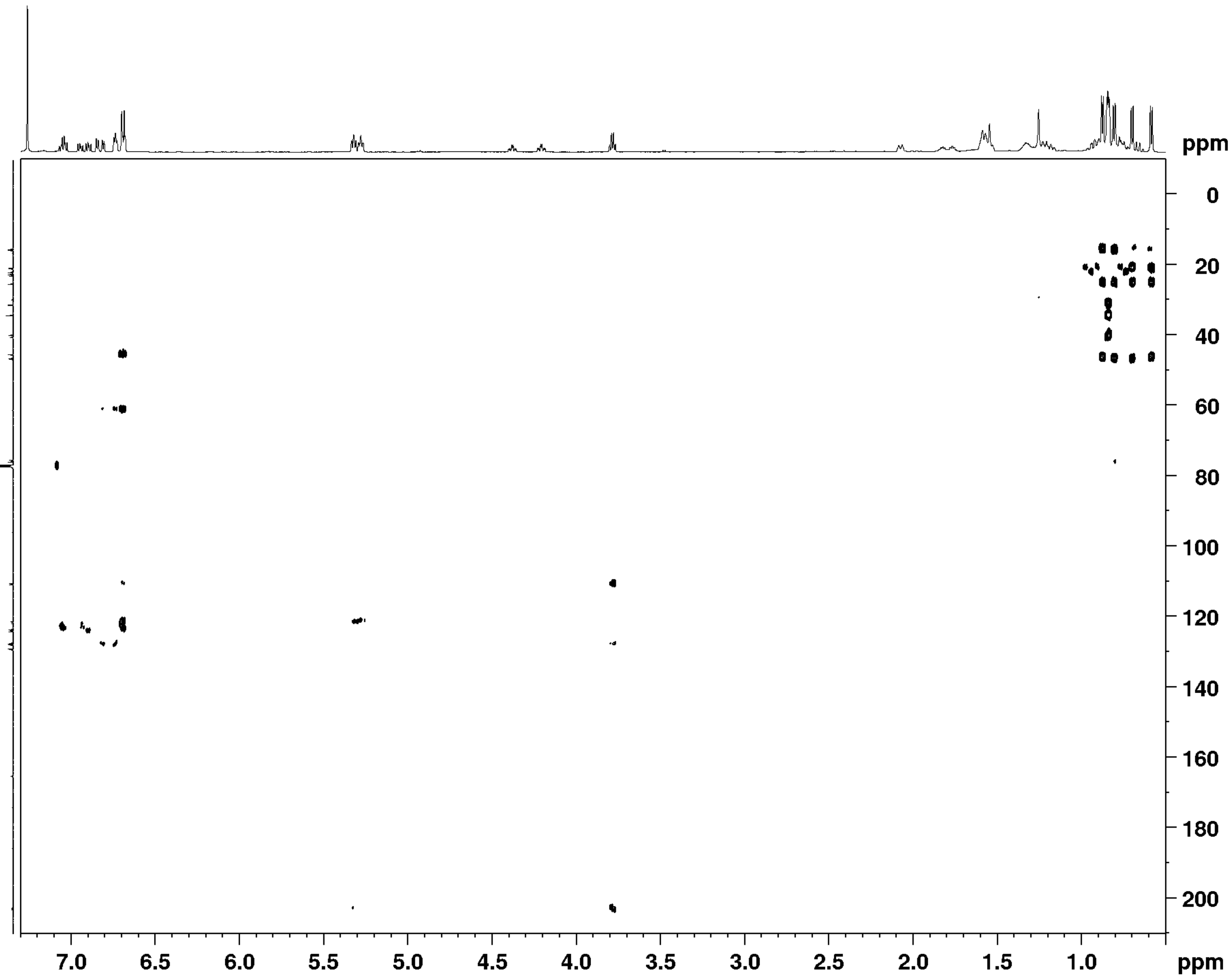

Figure S92: ^{1}H, ^{13}C HMBC spectrum of (L)-**6**.

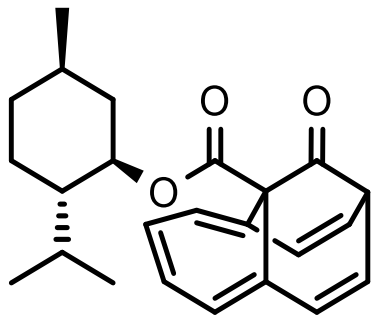

(L)-**6**

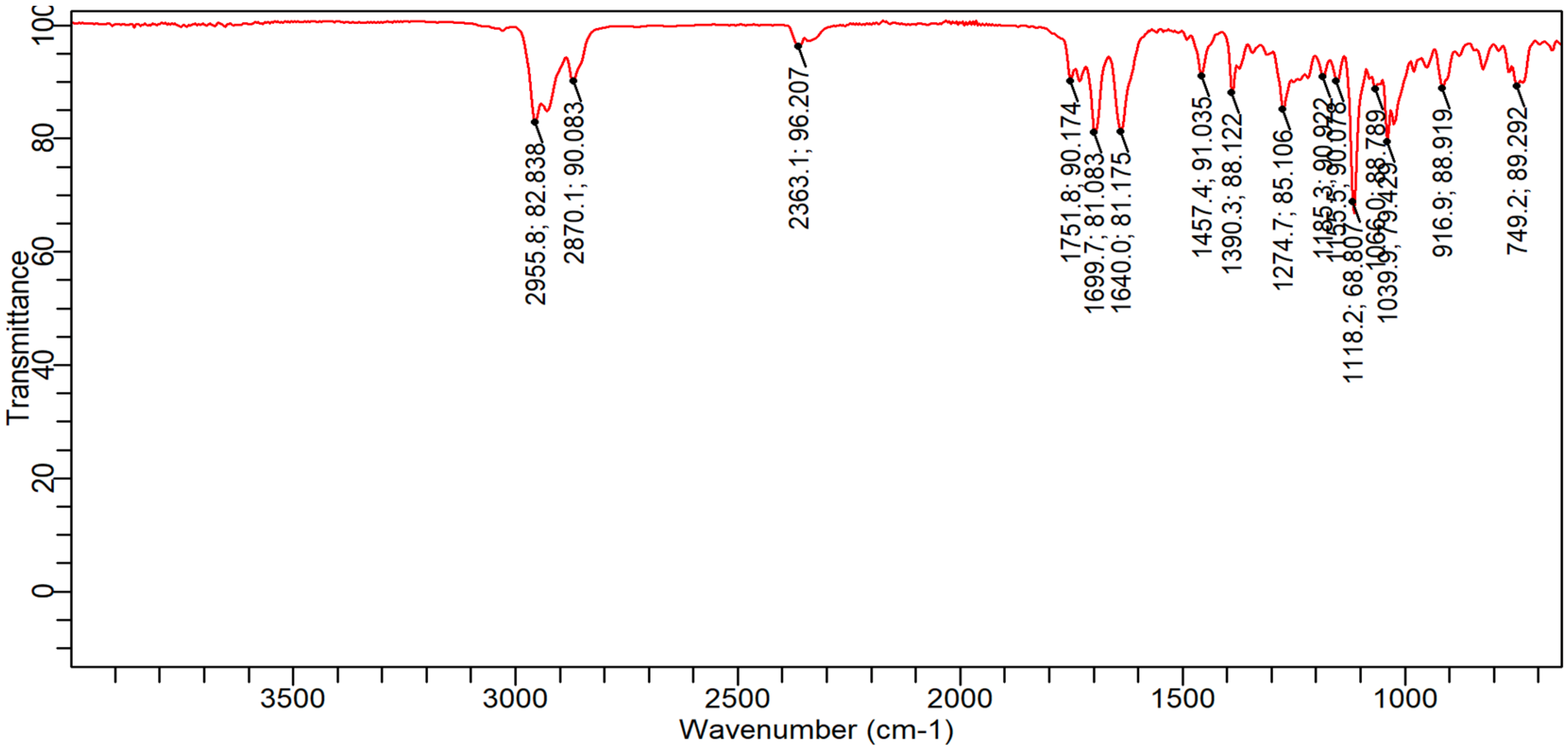

Figure S93: IR spectrum of (L)-**6**.

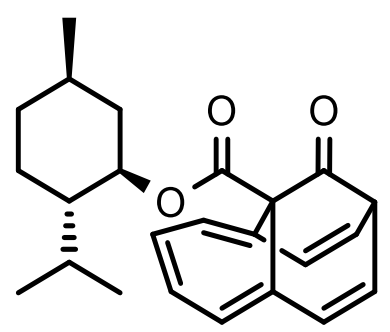

(L)-**6**

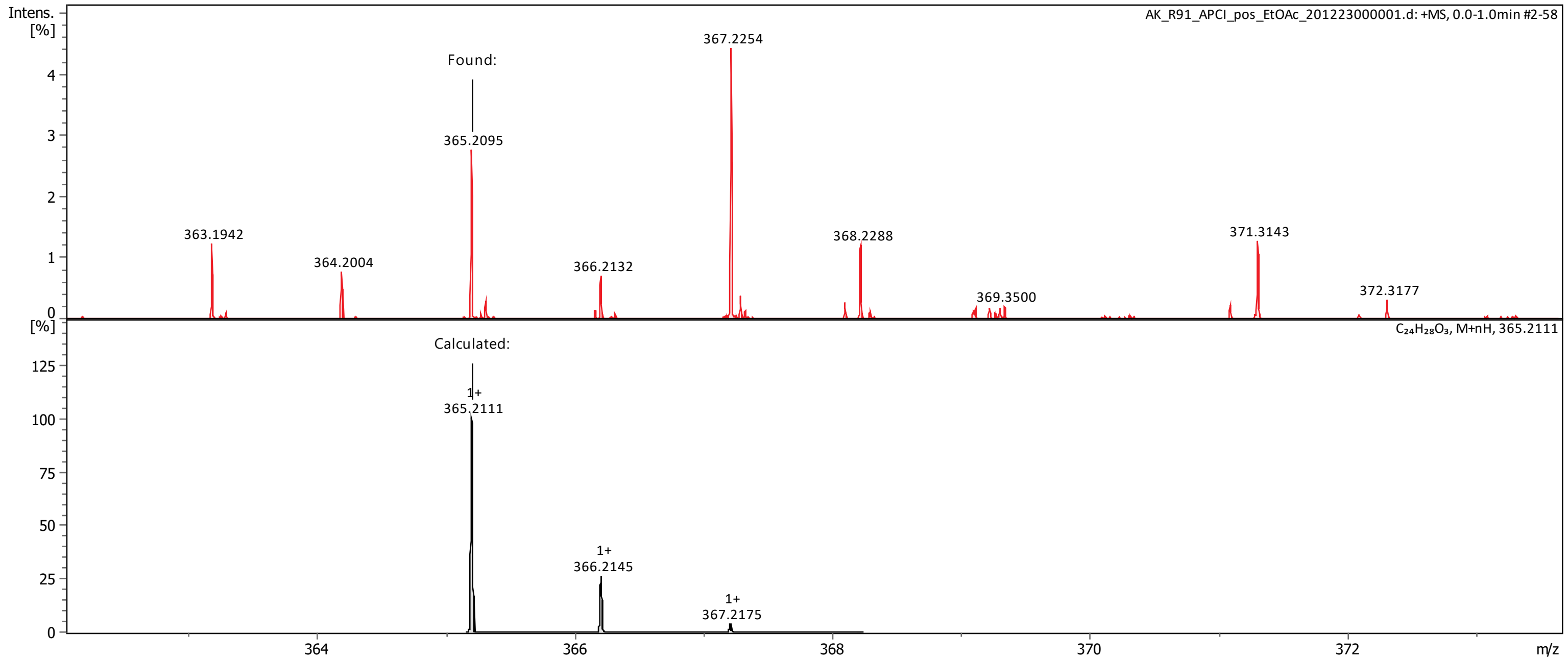

Figure S94: HRMS spectrum of (L)-**6**.

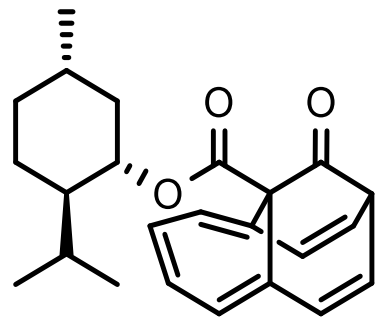

(D)-**6**

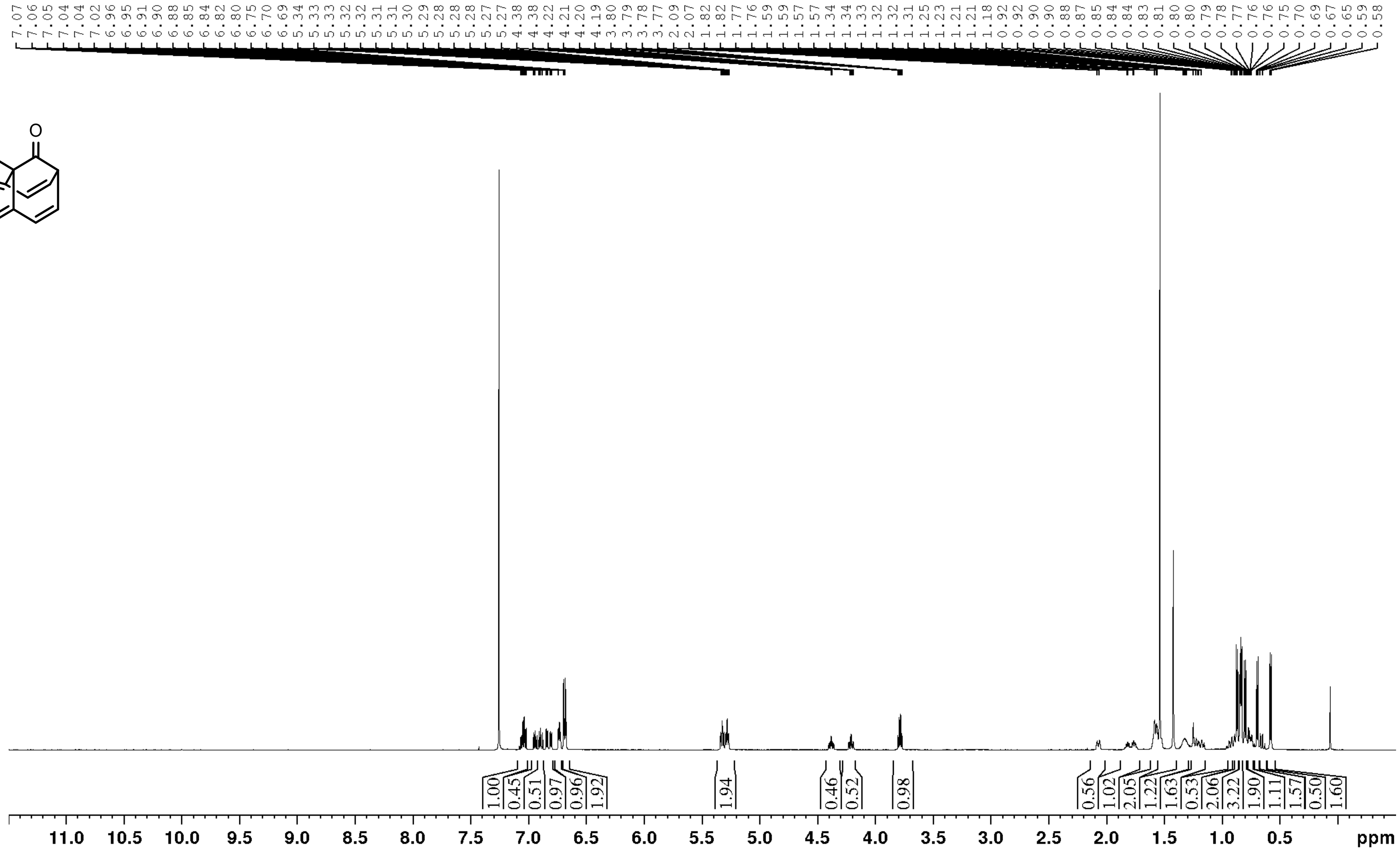

Figure S95: ^{1}H NMR spectrum of (D)-**6**.

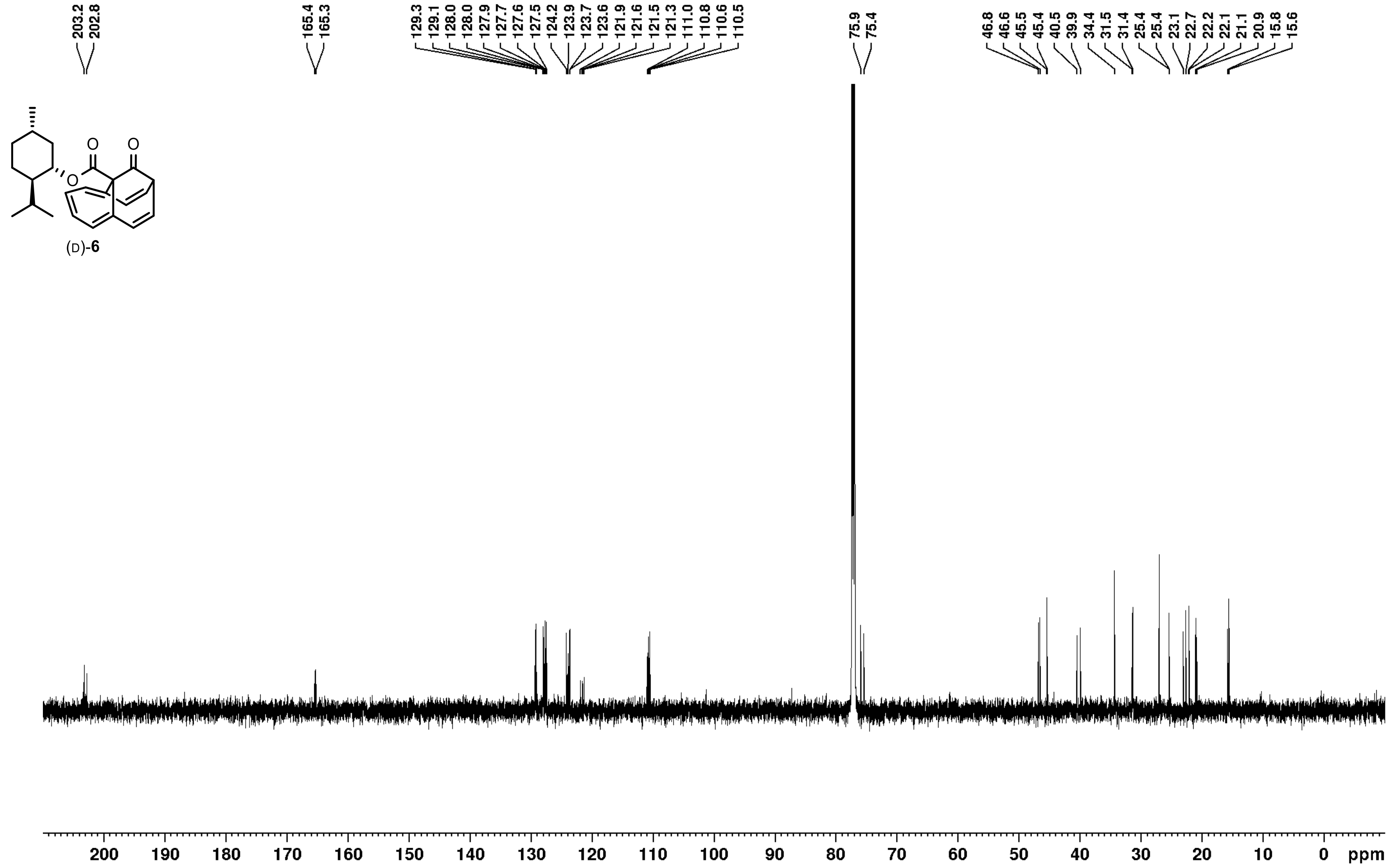

Figure S96: ^{13}C NMR spectrum of (D)-**6**.

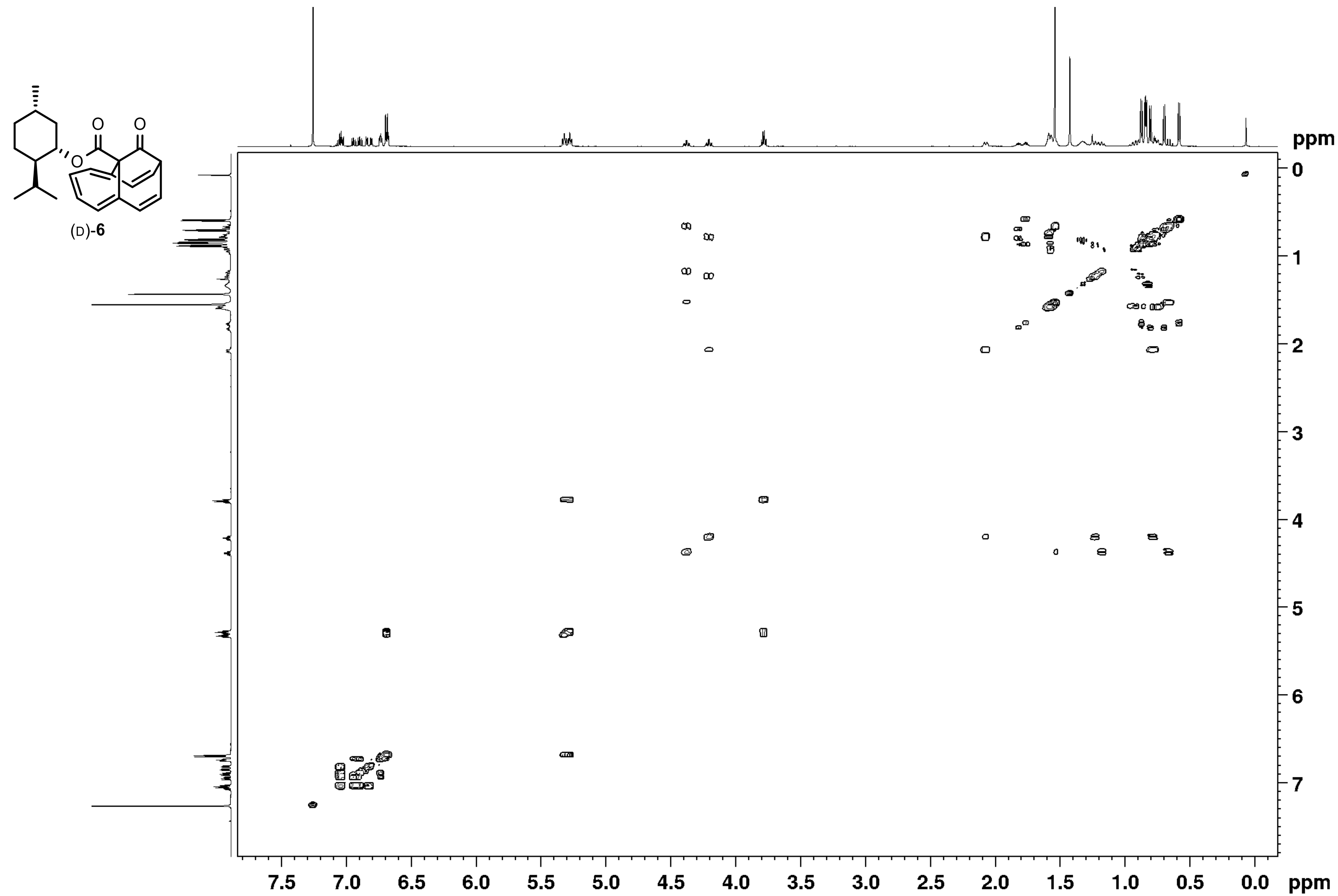

Figure S97: ^{1}H, ^{1}H COSY spectrum of (D)-**6**.

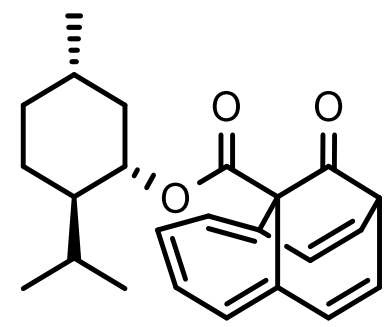

(D)-**6**

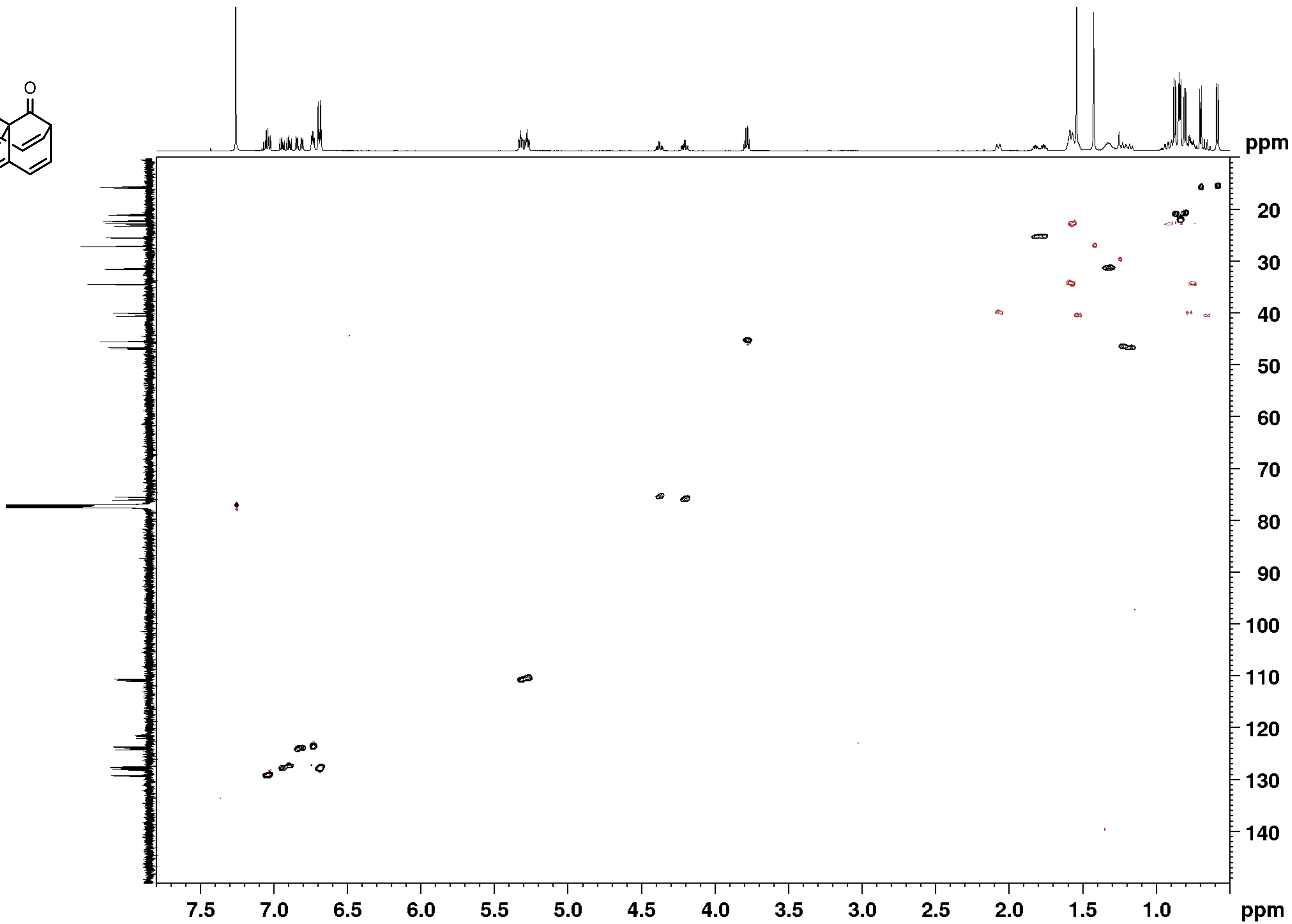

Figure S98: ^{1}H, ^{13}C HSQC spectrum of (D)-**6**.

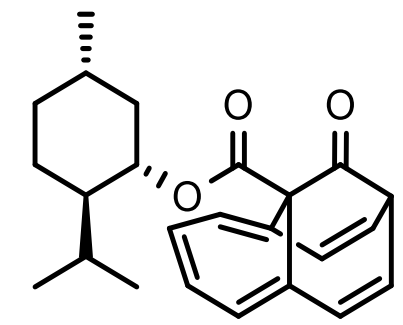

(D)-**6**

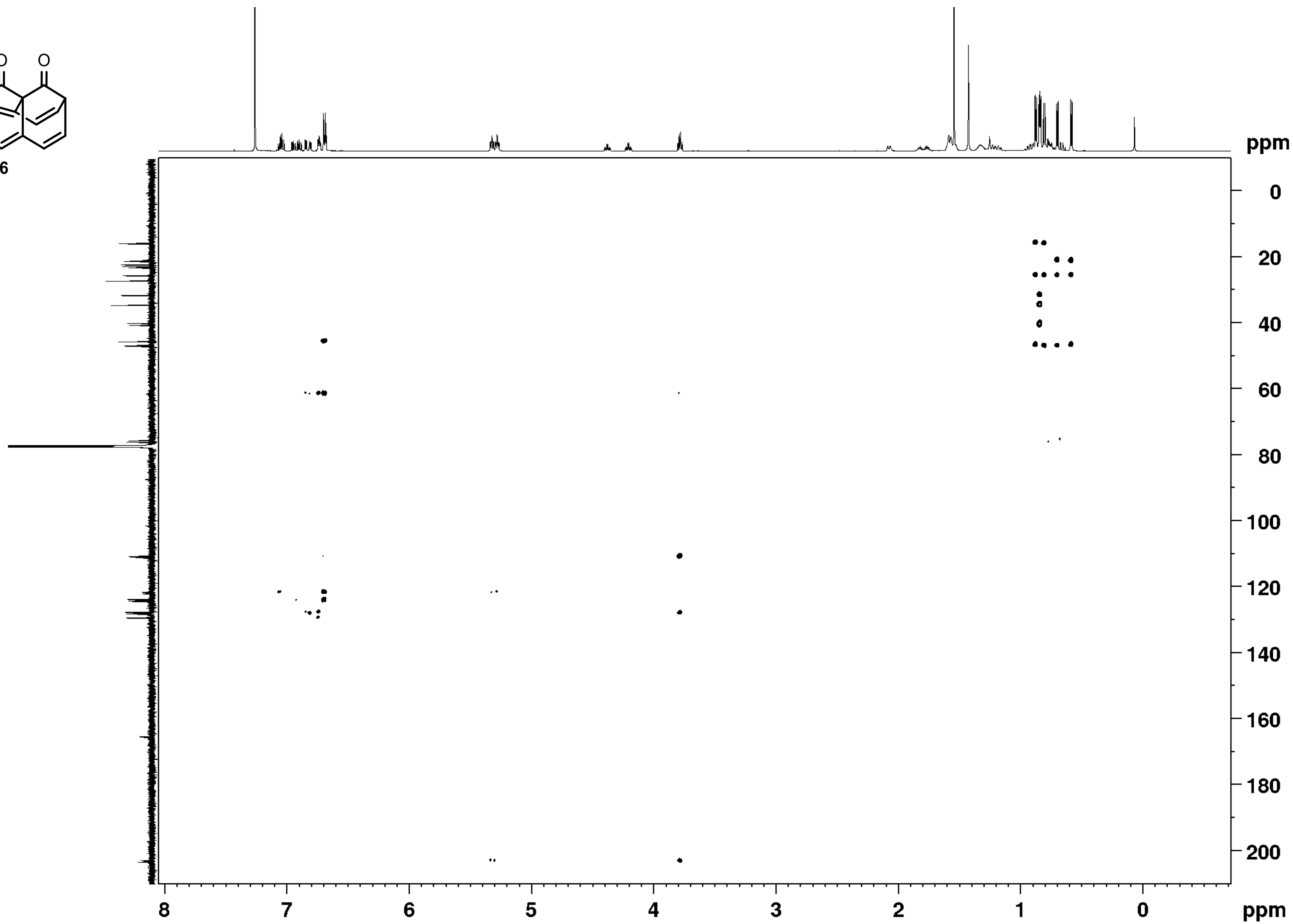

Figure S99: ^{1}H, ^{13}C HMBC spectrum of (D)-**6**.

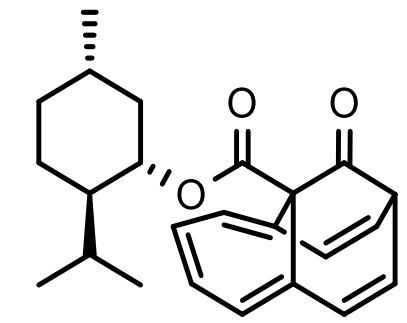

(D)-**6**

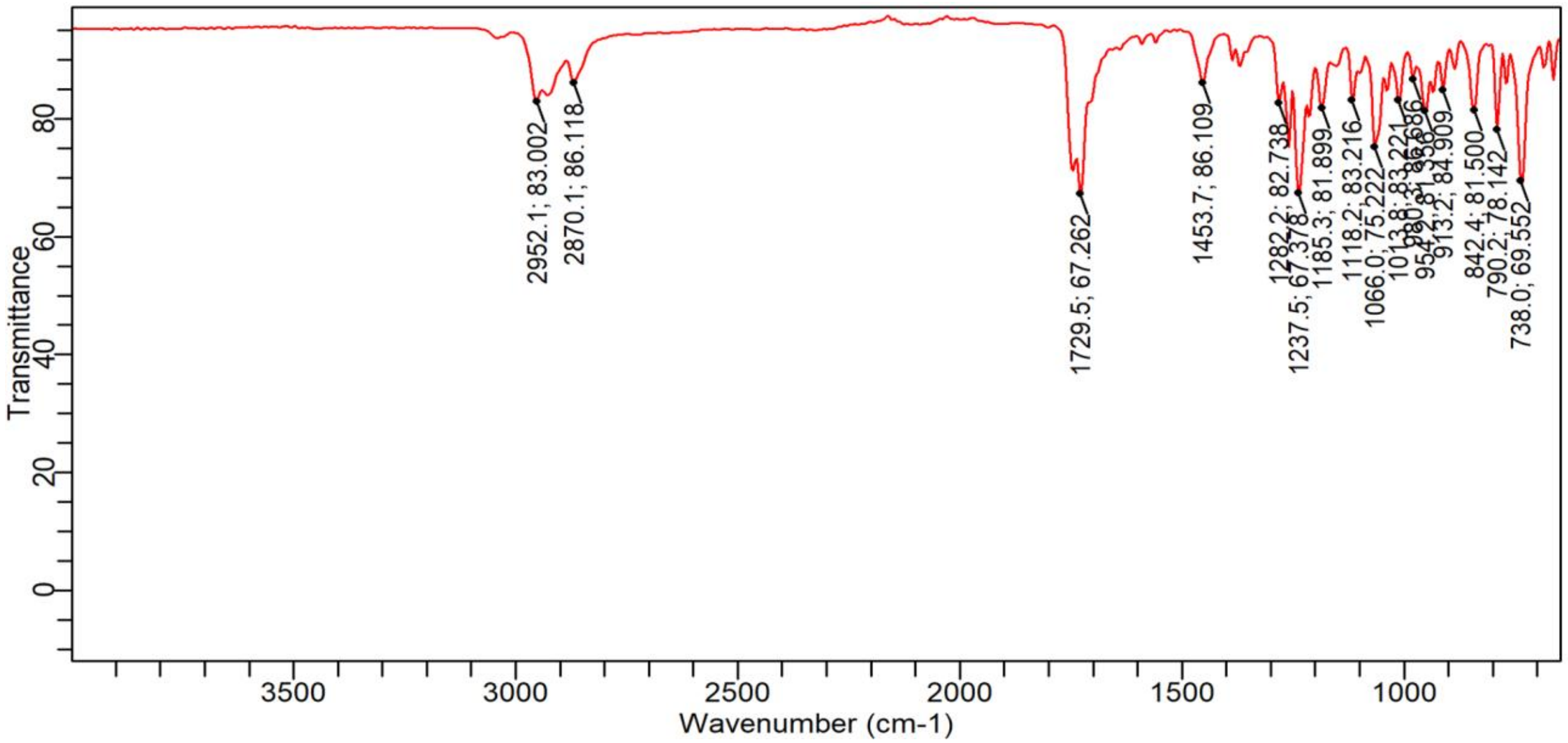

Figure S100: IR spectrum of (D)-**6**.

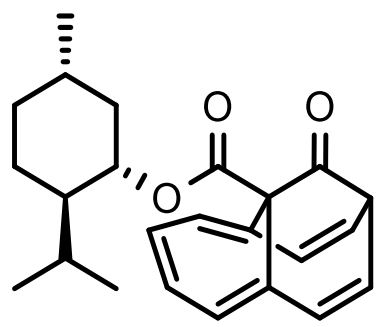

(D)-**6**

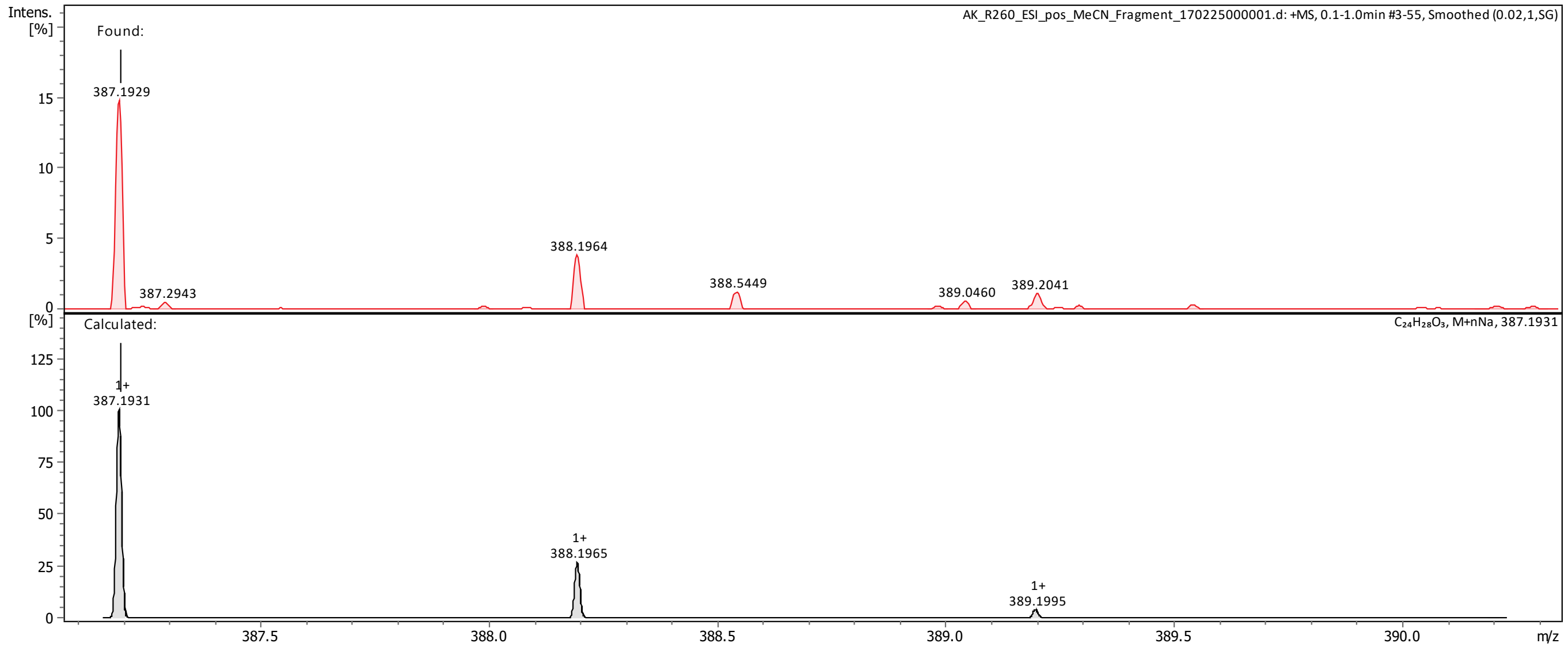

Figure S101: HRMS spectrum of (D)-**6**.

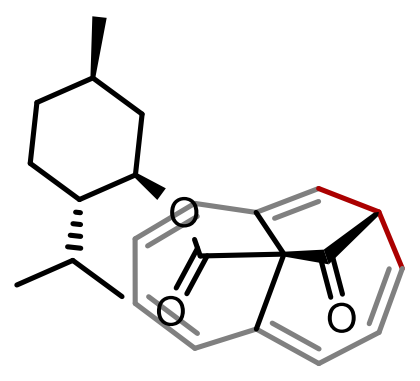

(L,*S*)-**7**

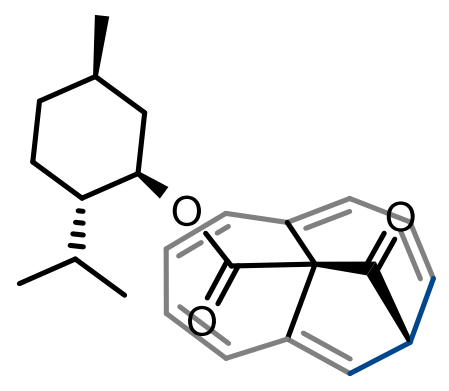

(L,*R*)-**7**

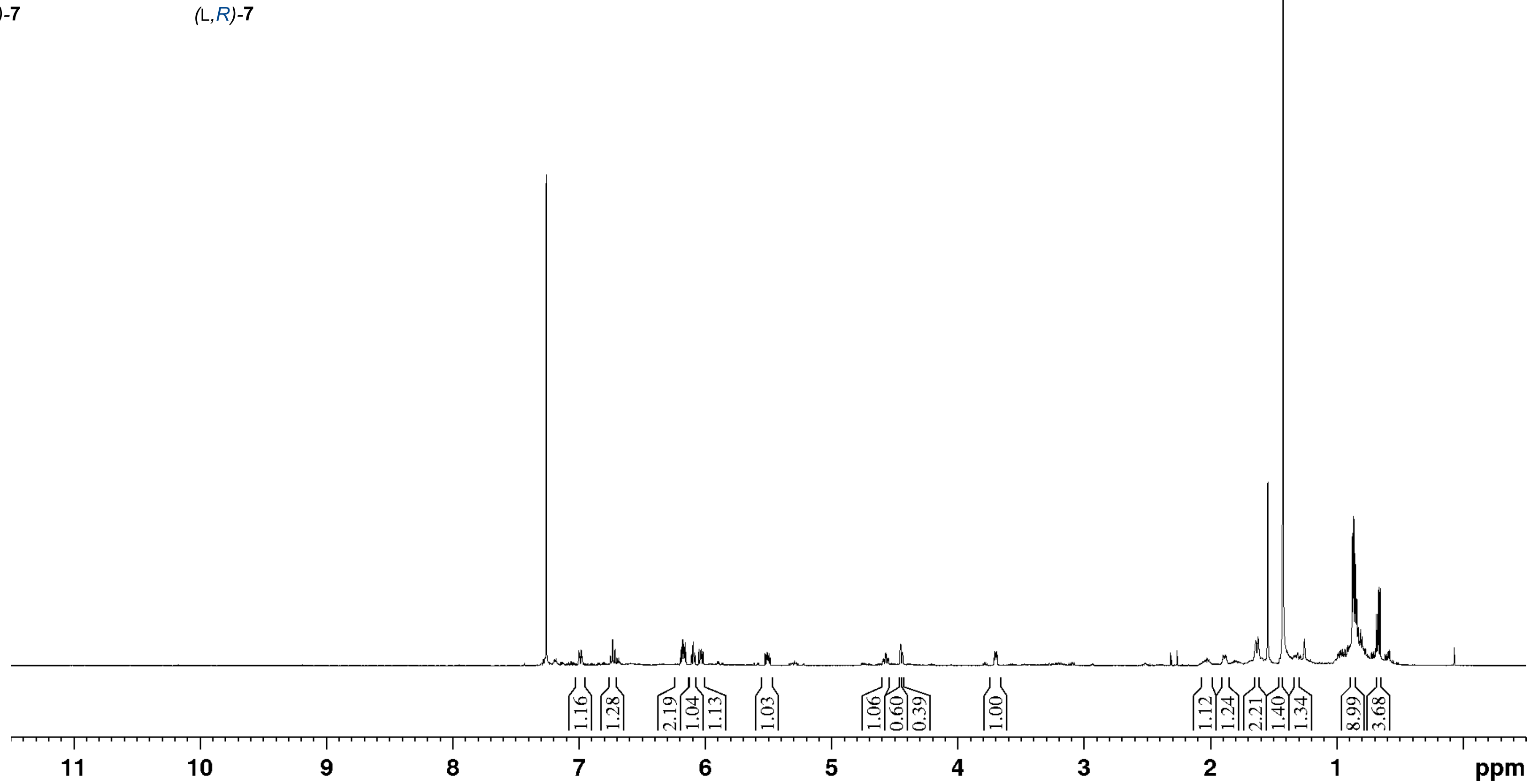

Figure S102: ^{1}H NMR spectrum of (L)-**7**.

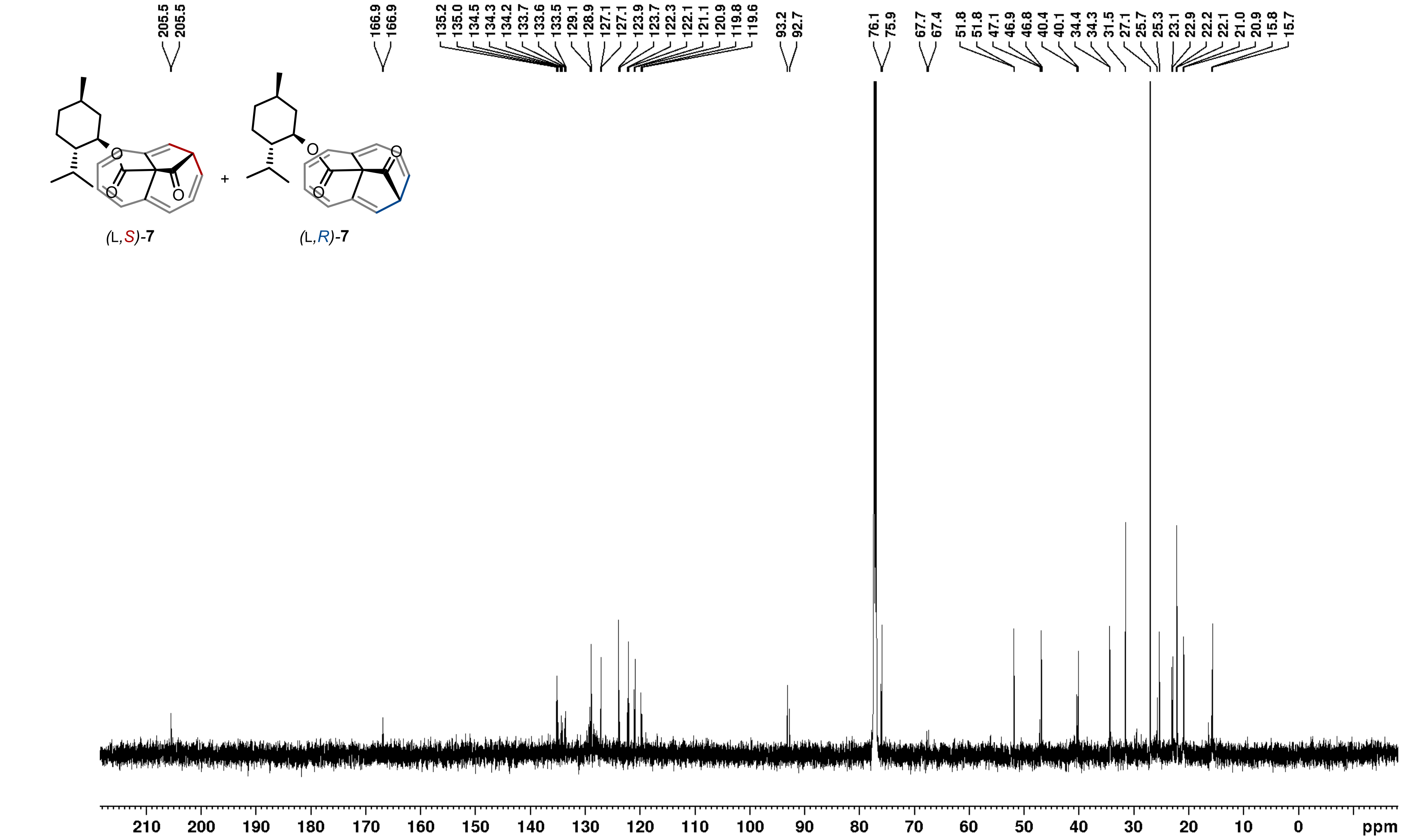

Figure S103: ^{13}C NMR spectrum of (L)-**7**.

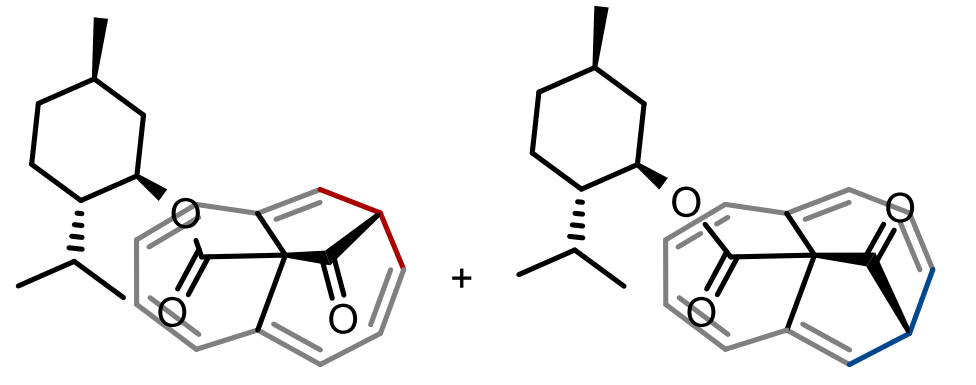

(L,*S*)-**7** + (L,*R*)-**7**

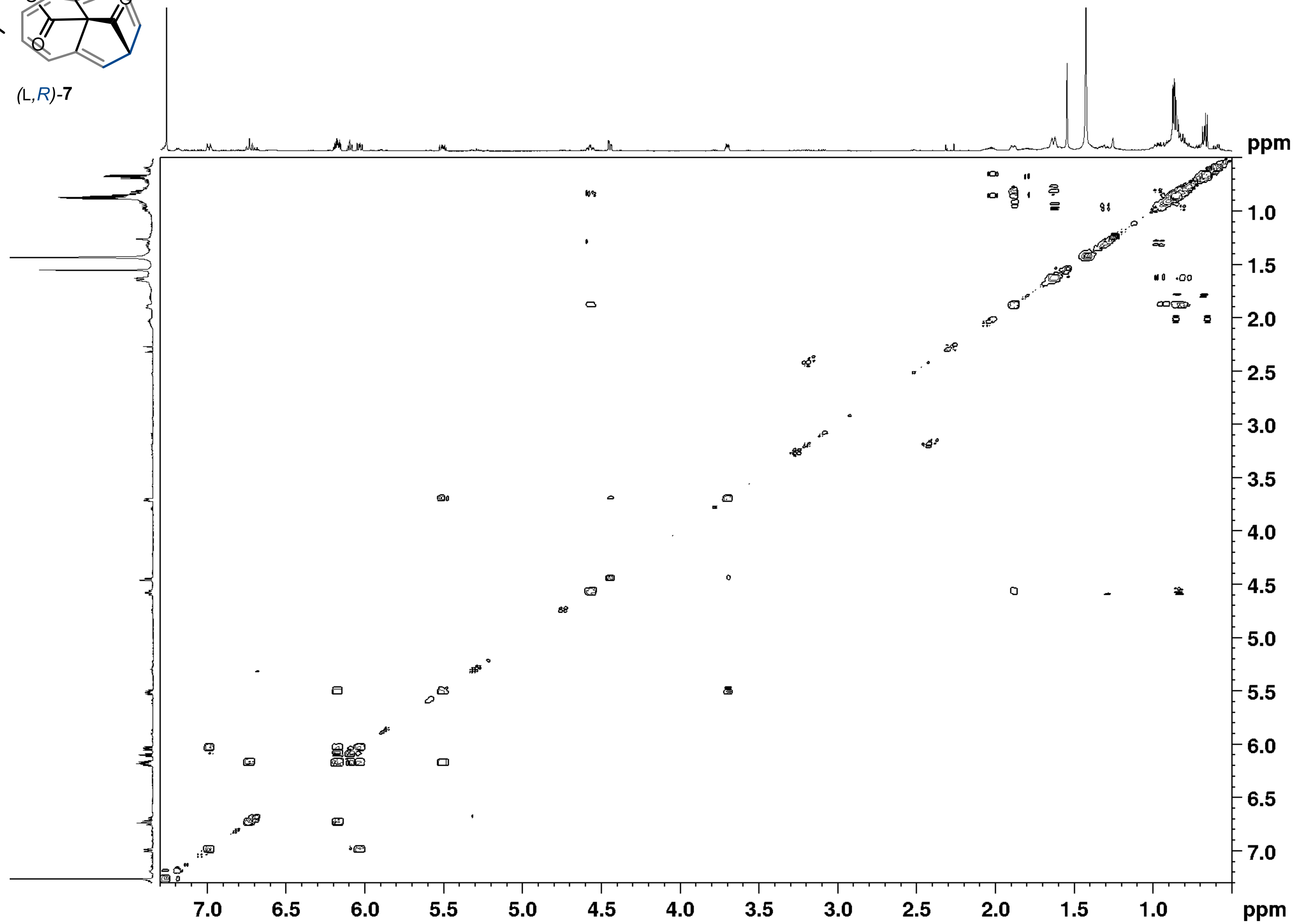

Figure S104: ^{1}H, ^{1}H COSY spectrum of (L)-**7**.

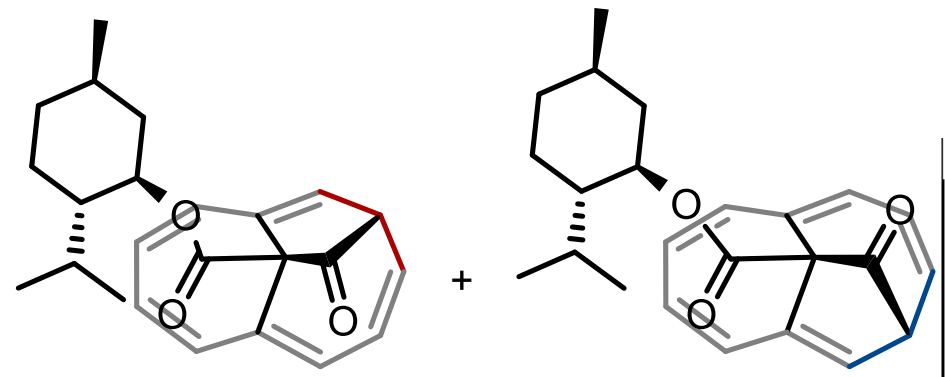

(L,*S*)-**7** (L,*R*)-**7**

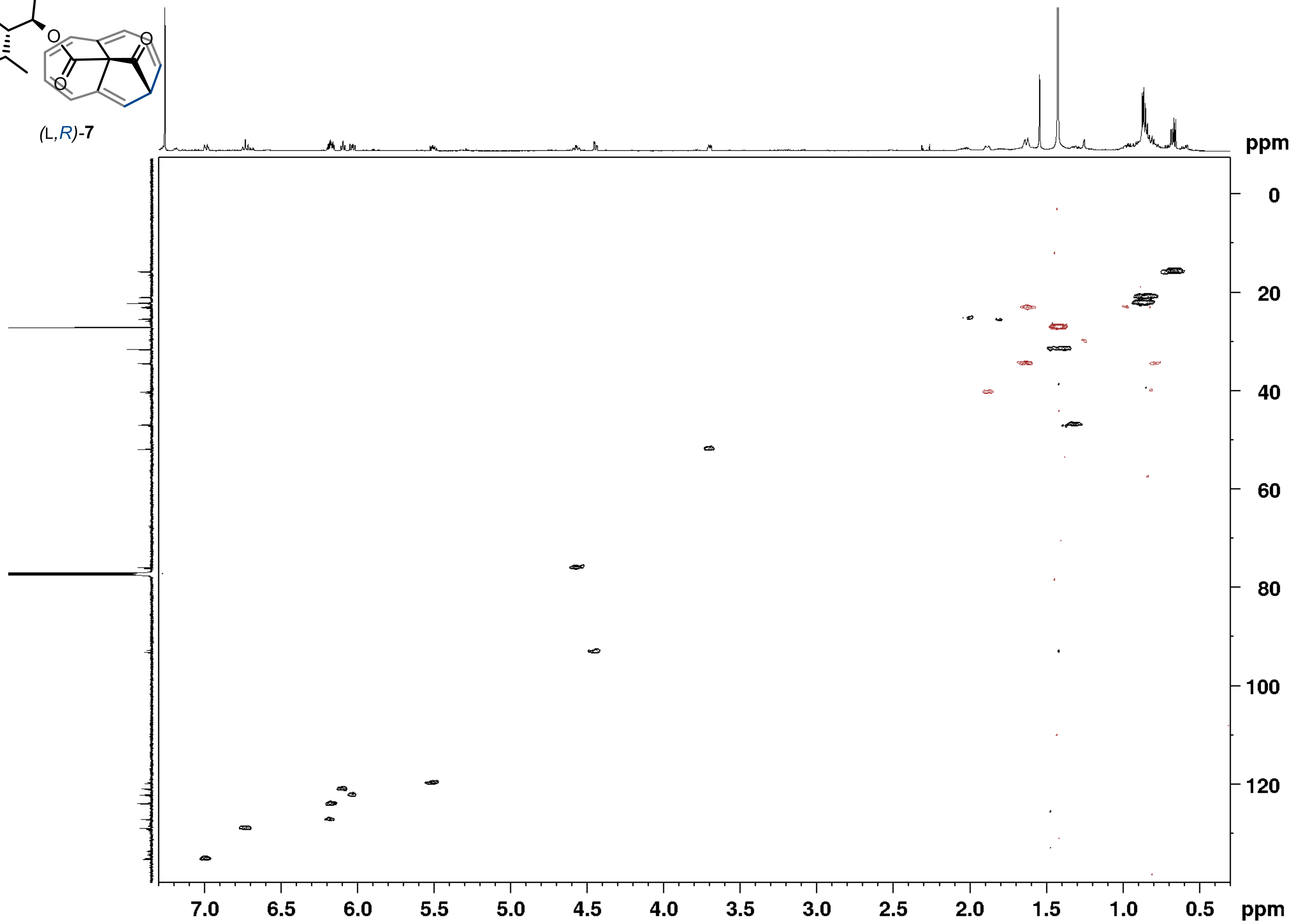

Figure S105: ^{1}H, ^{13}C HSQC spectrum of (L)-**7**.

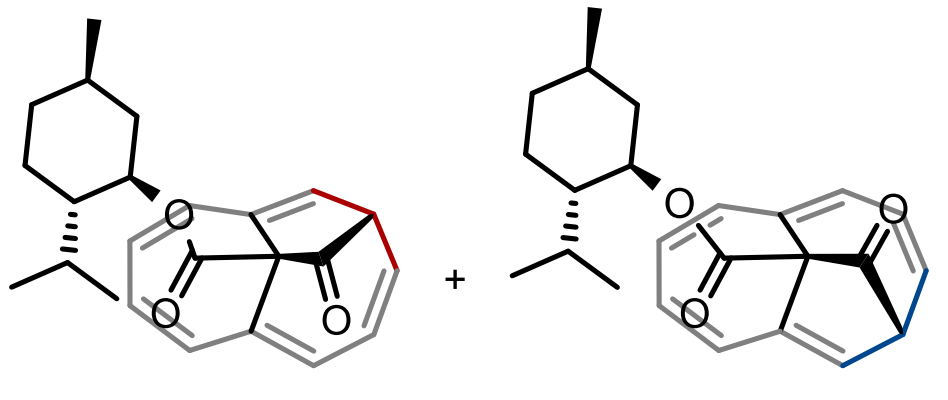

(L,*S*)-**7** + (L,*R*)-**7**

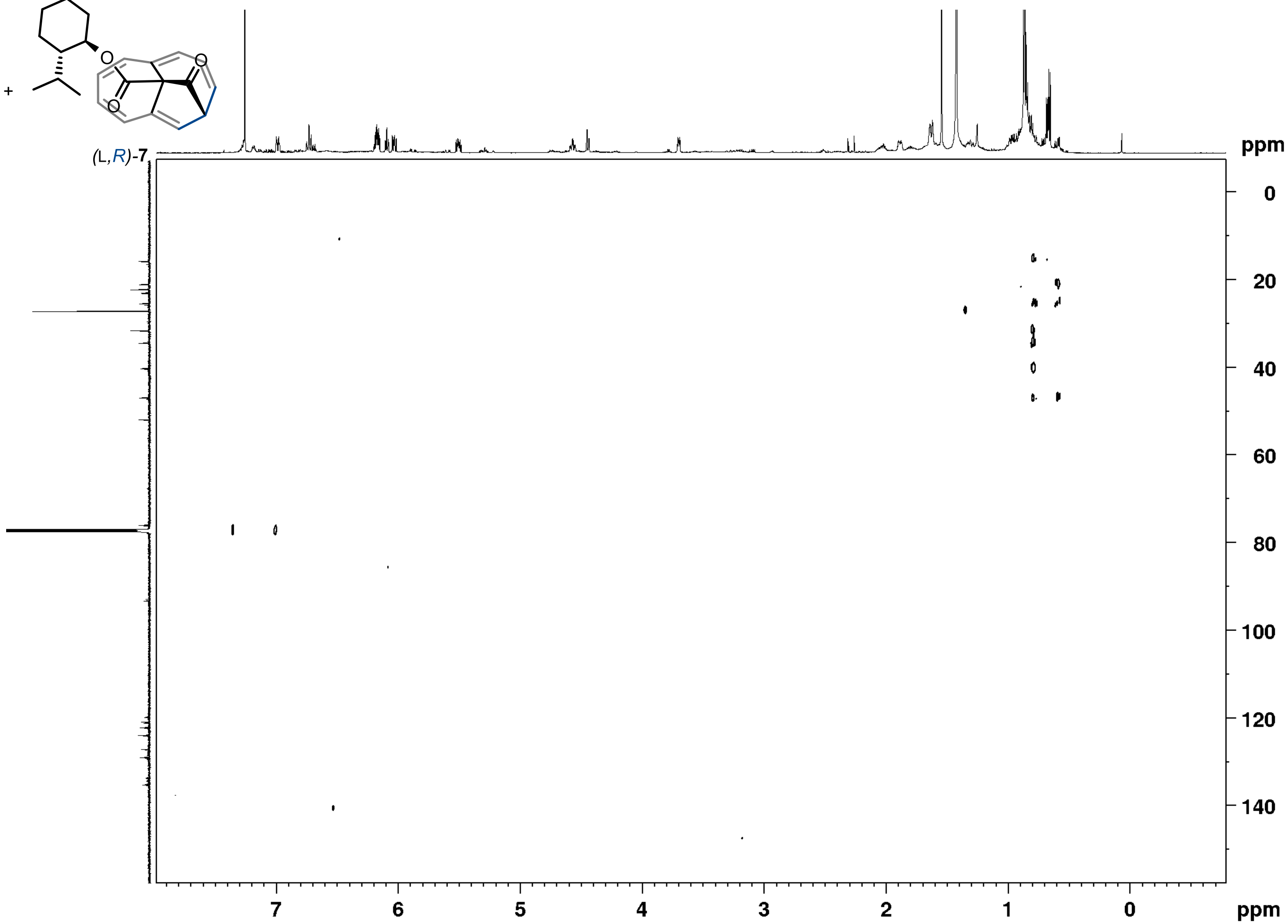

Figure S106: ^{1}H, ^{13}C HMBC spectrum of (L)-**7**.

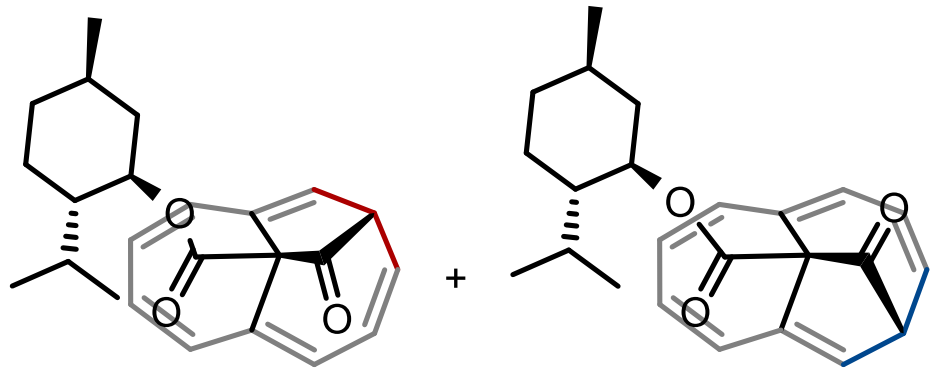

(L,*S*)-**7** (L,*R*)-**7**

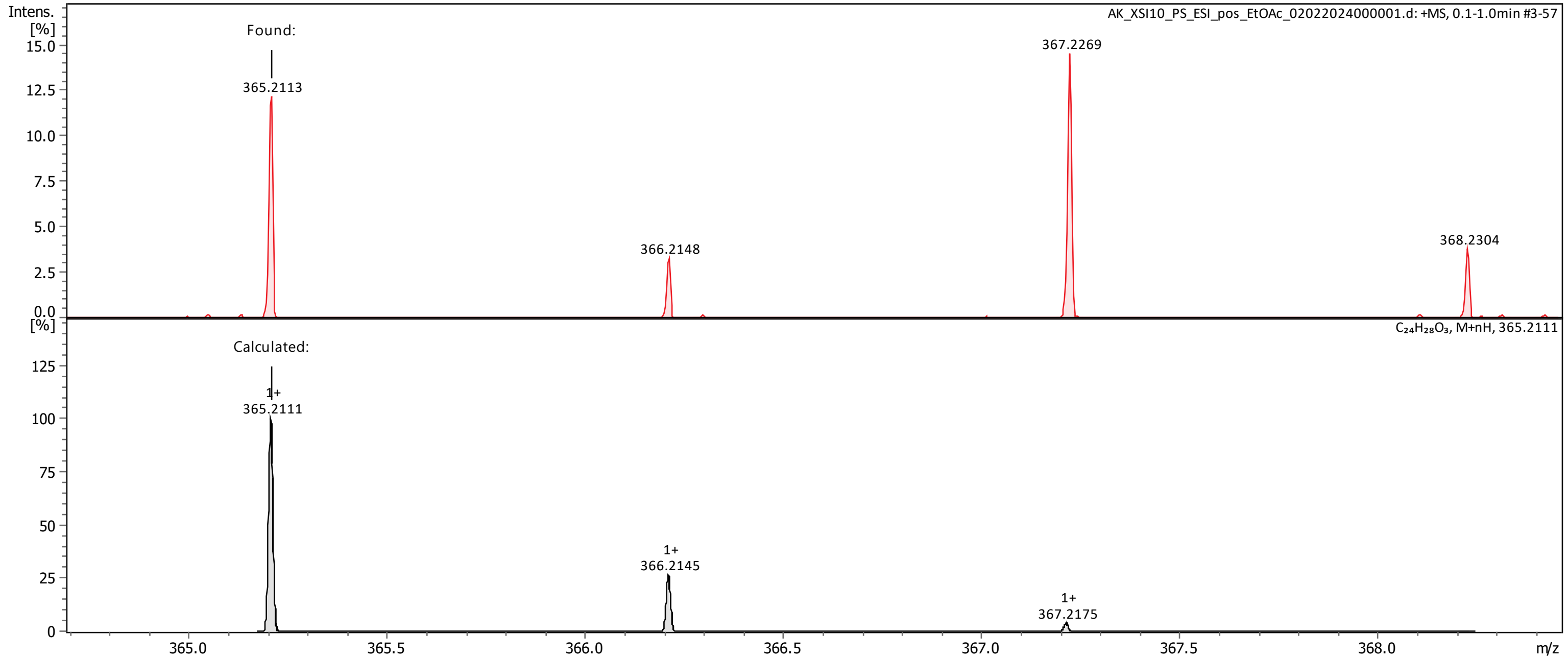

Figure S107: HRMS spectrum of (L)-**7**.

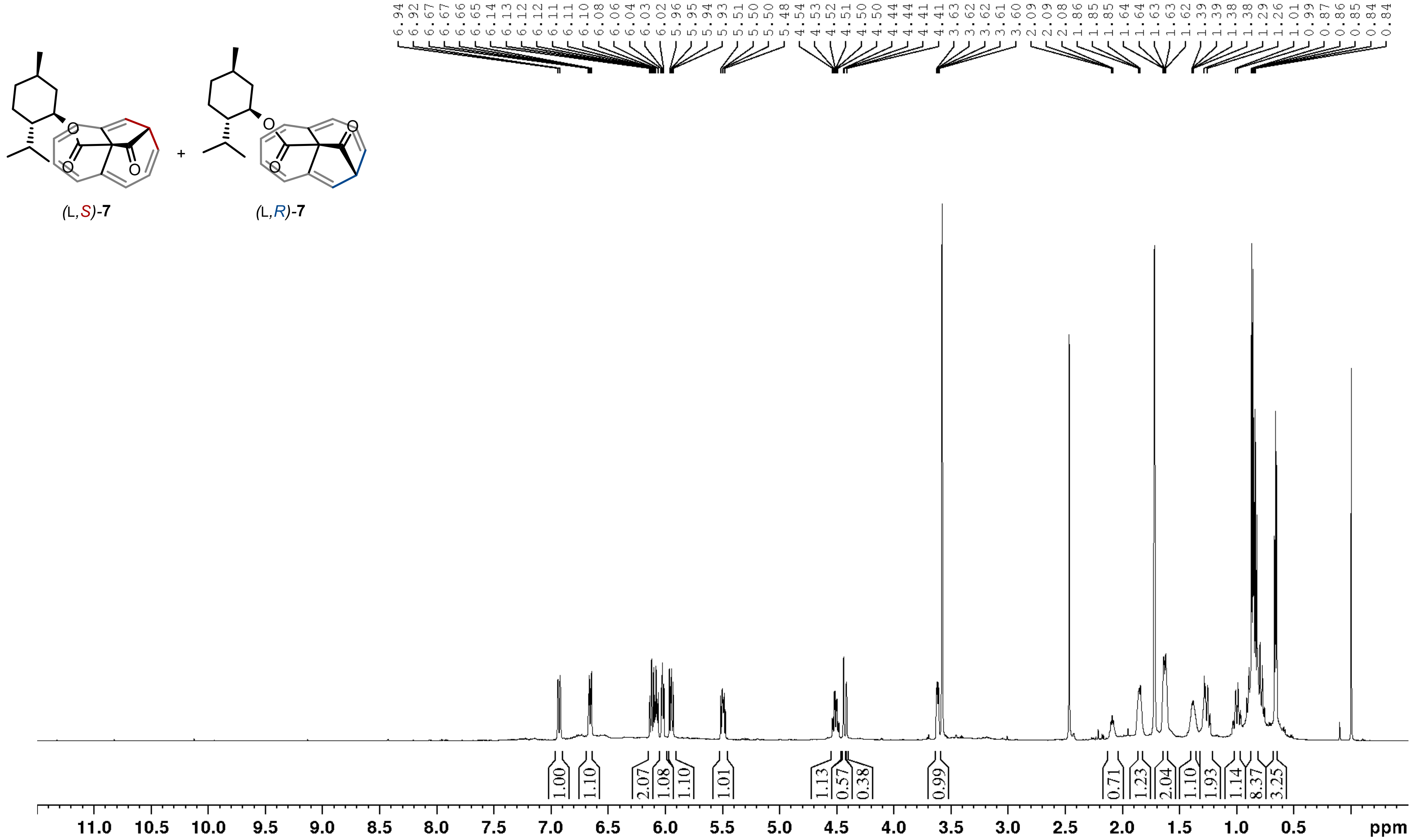

Figure S108: ^{1}H NMR spectrum of (L)-**7** in THF-d_8.

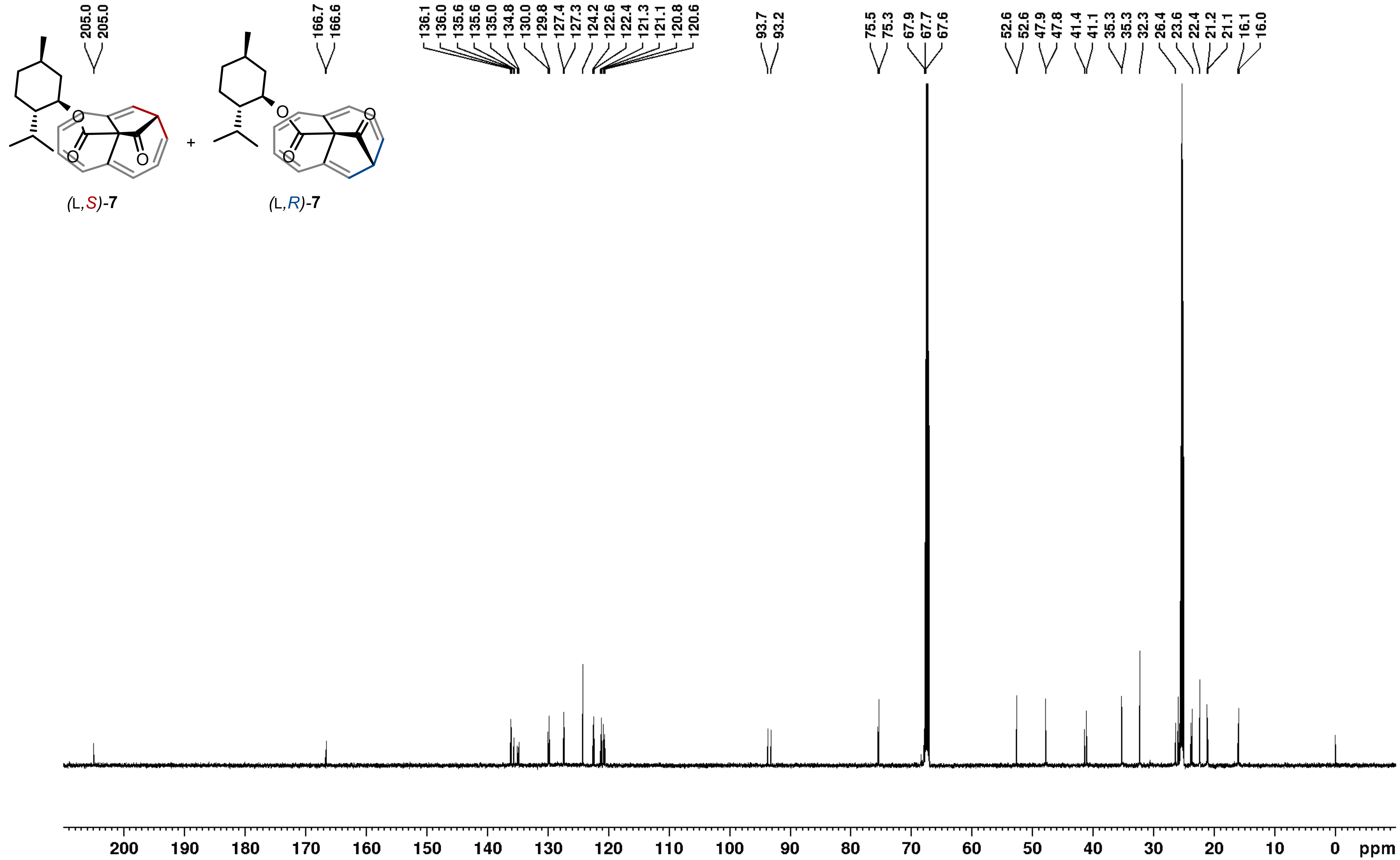

Figure S109: ^{13}C NMR spectrum of (L)-**7** in THF-d_8.

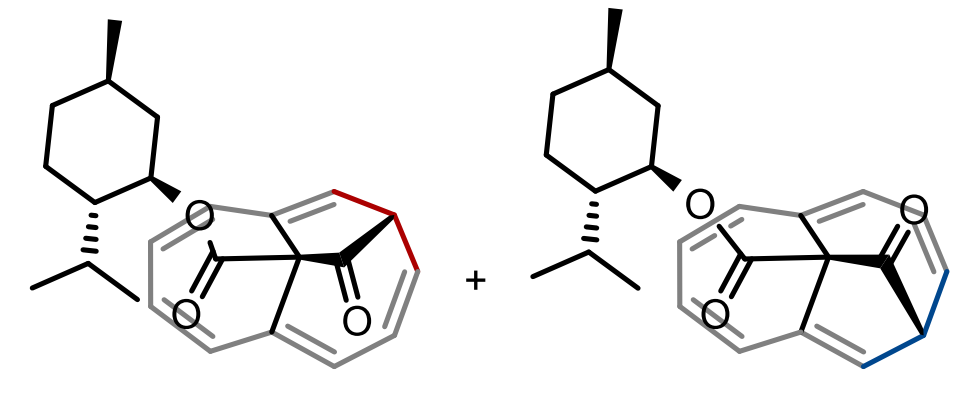

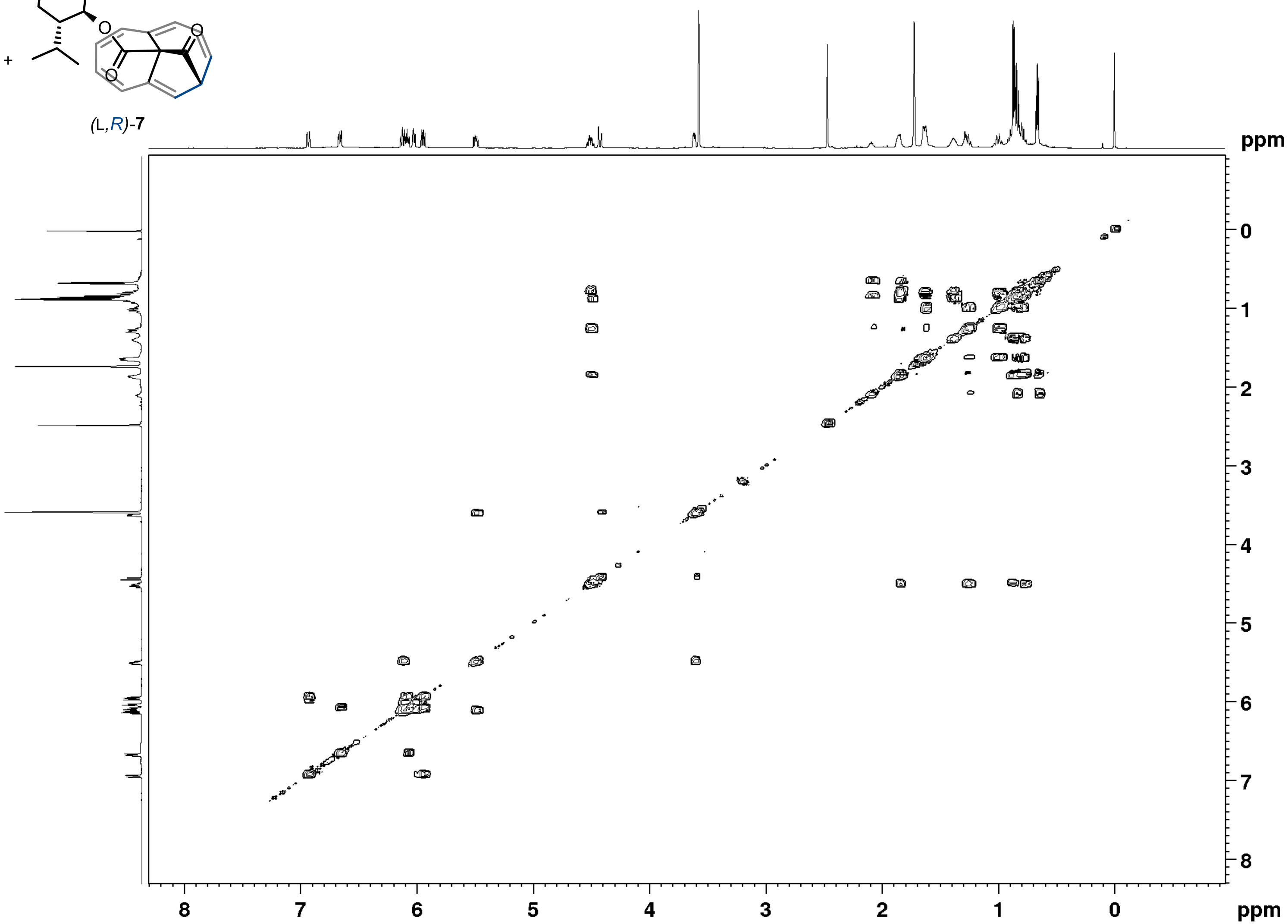

Figure S110: ^{1}H, ^{1}H COSY spectrum of (L)-**7** measured in THF-d_8.

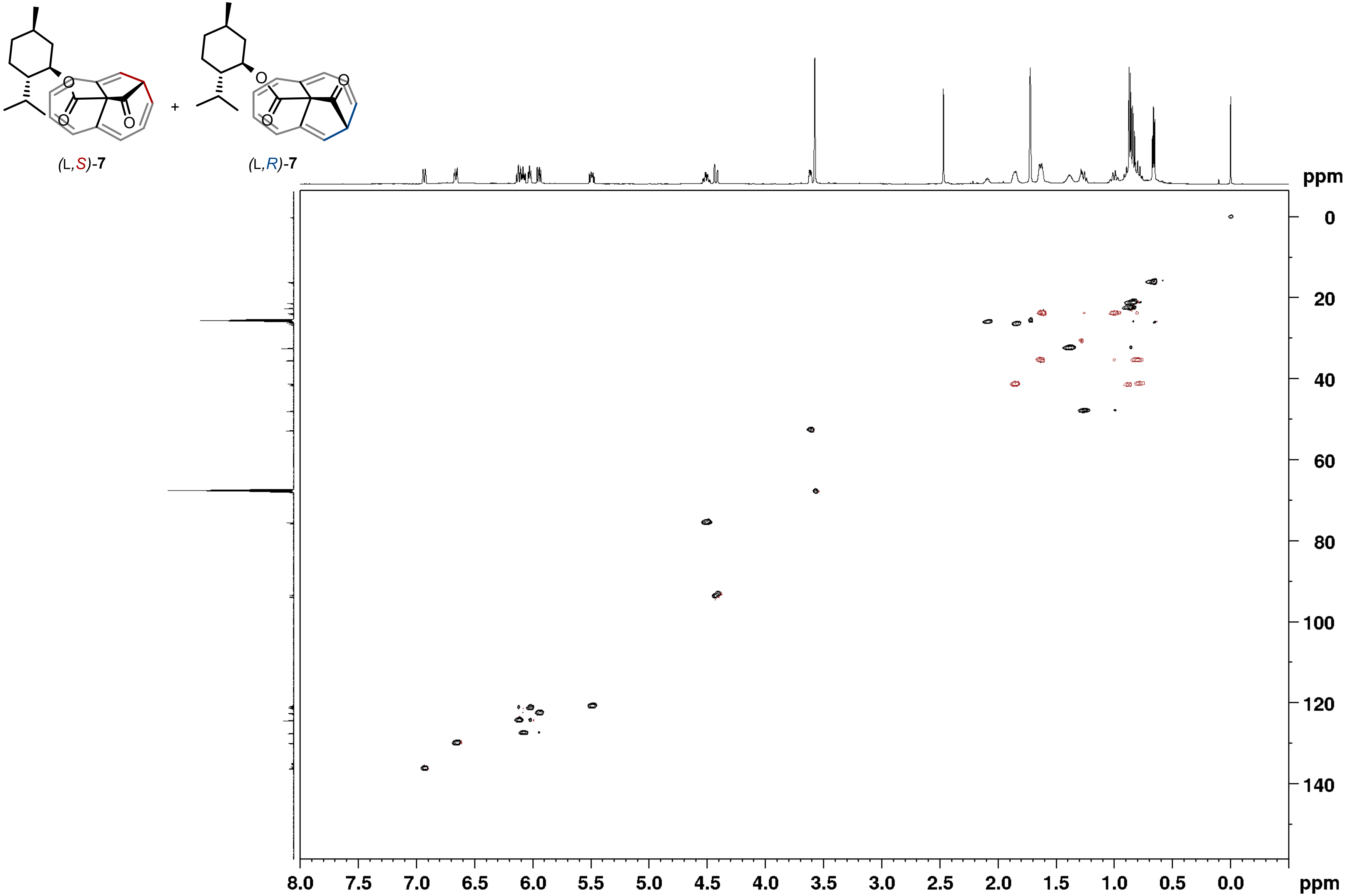

Figure S111: ^{1}H, ^{13}C HSQC spectrum of (L)-**7** measured in THF-d_8.

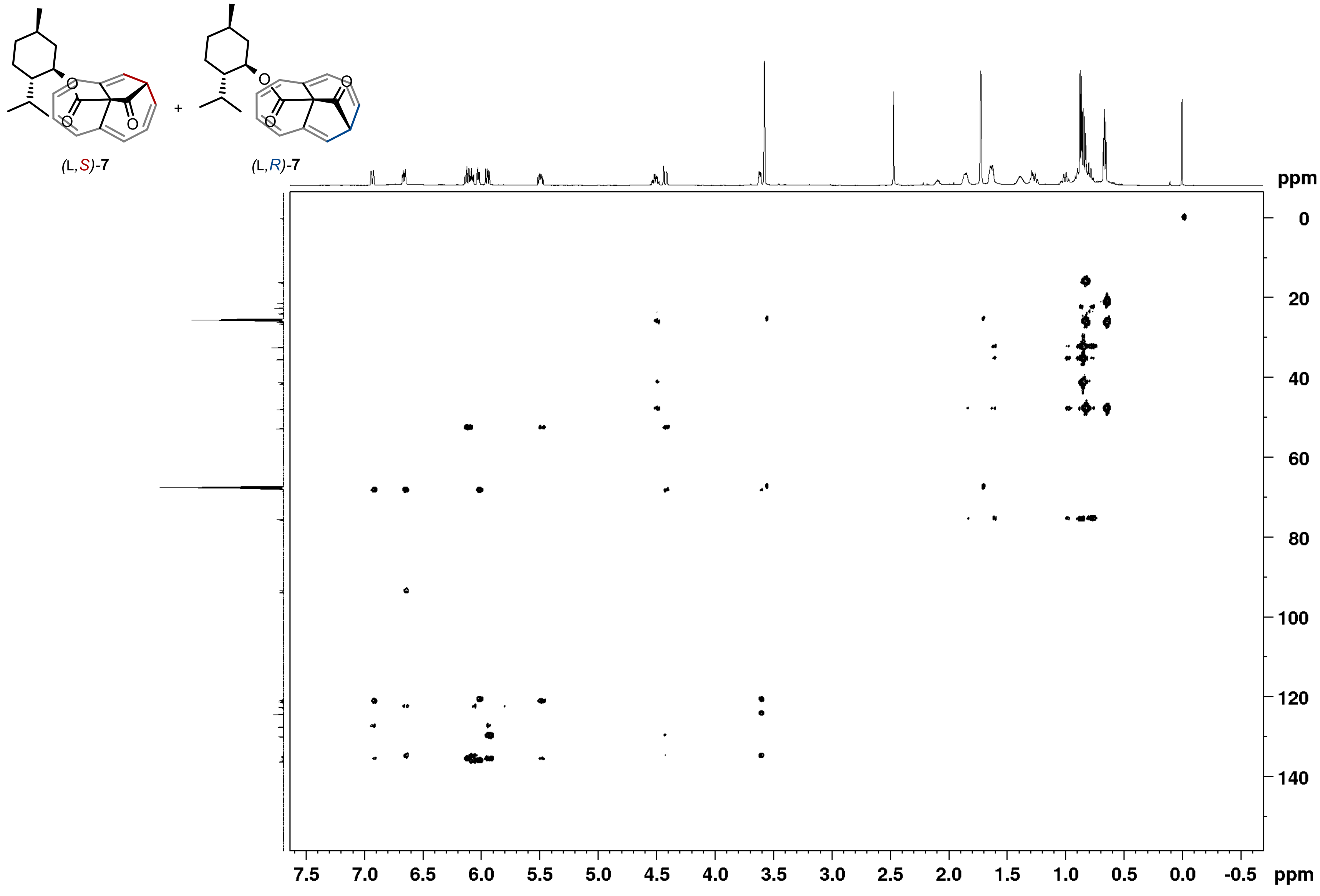

Figure S112: ^{1}H, ^{13}C HMBC spectrum of (L)-**7** measured in THF-d_8.

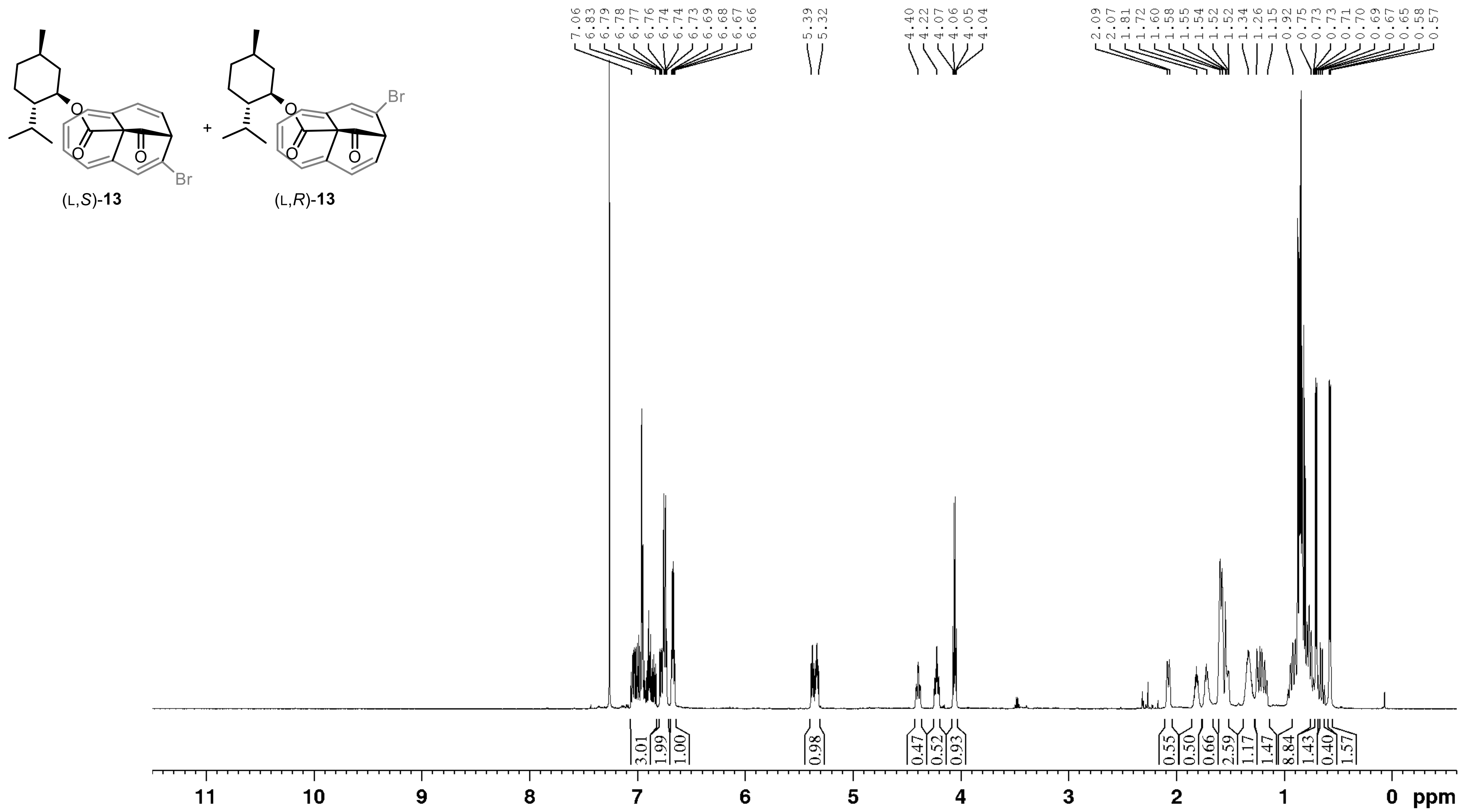

Figure S113: ^{1}H NMR spectrum of (L)-**13**.

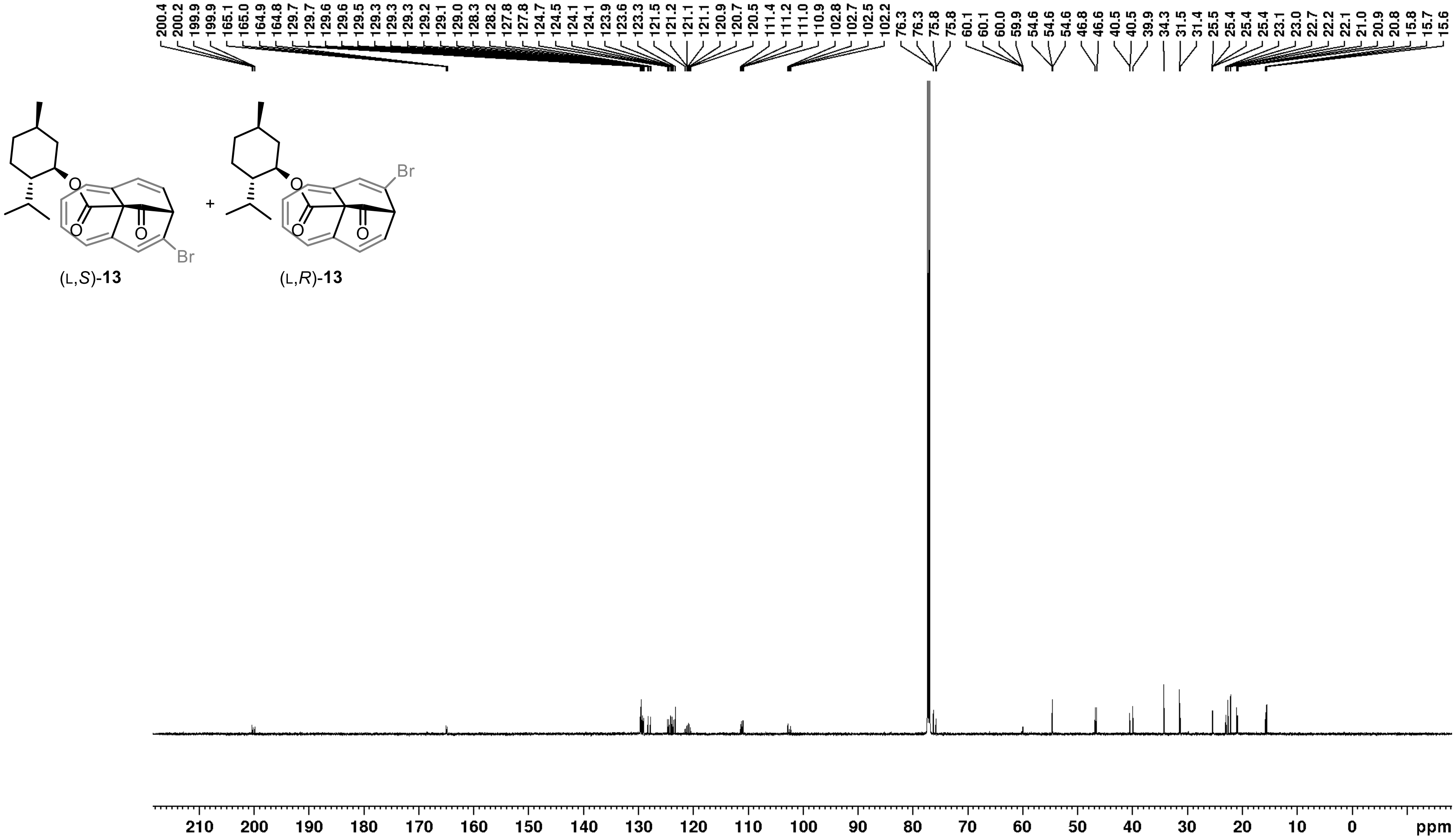

Figure S114: ^{13}C NMR spectrum of (L)-**13**.

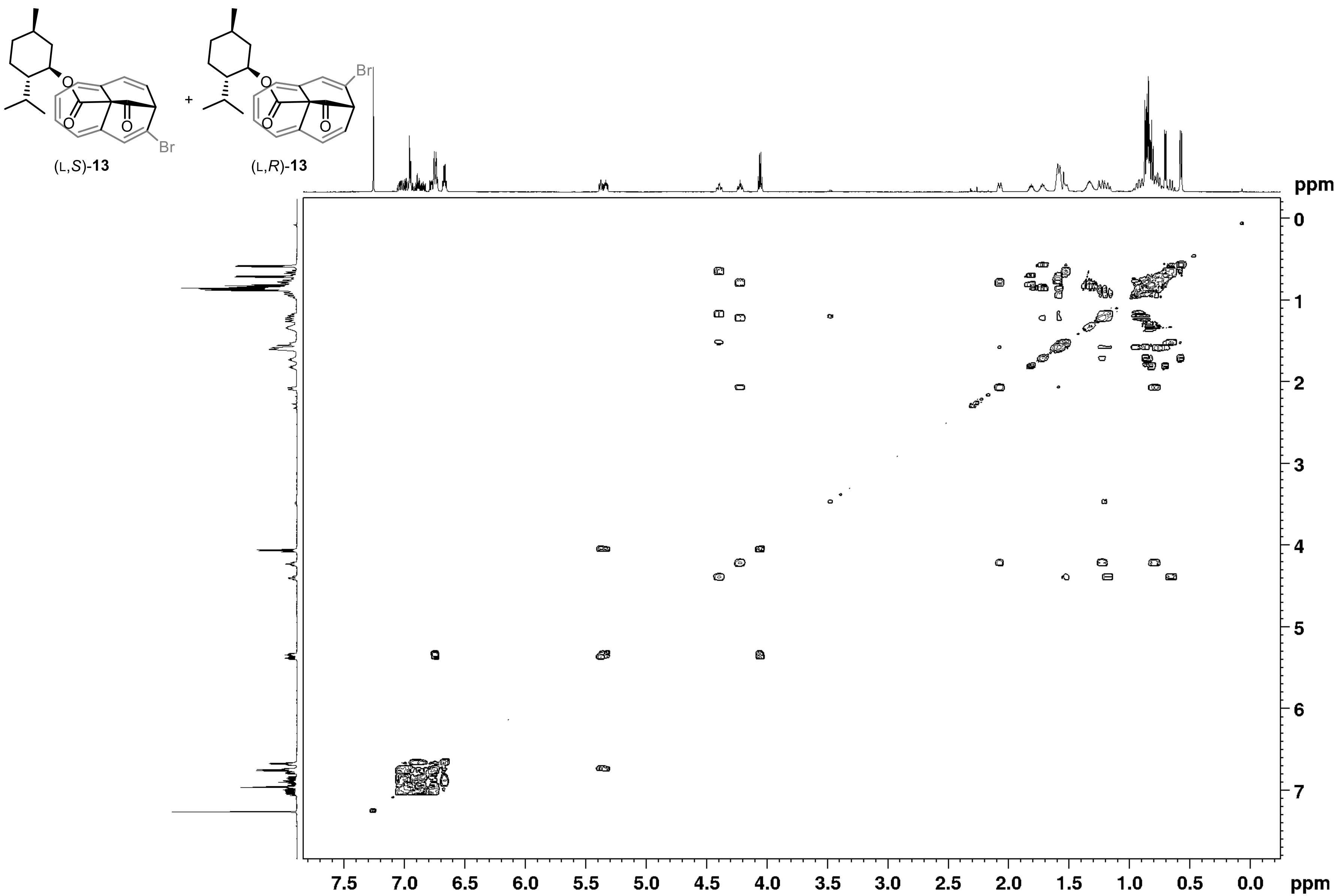

Figure S115: ^{1}H, ^{1}H COSY spectrum of (L)-**13**.

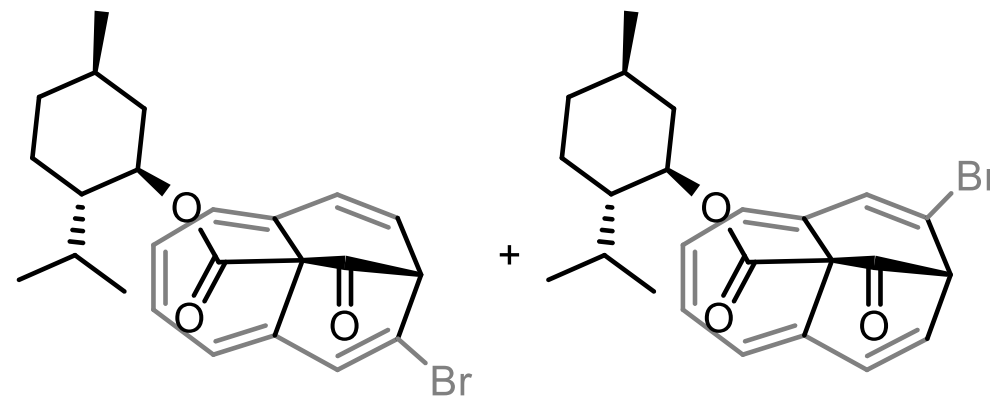

(L,*S*)-**13** (L,*R*)-**13**

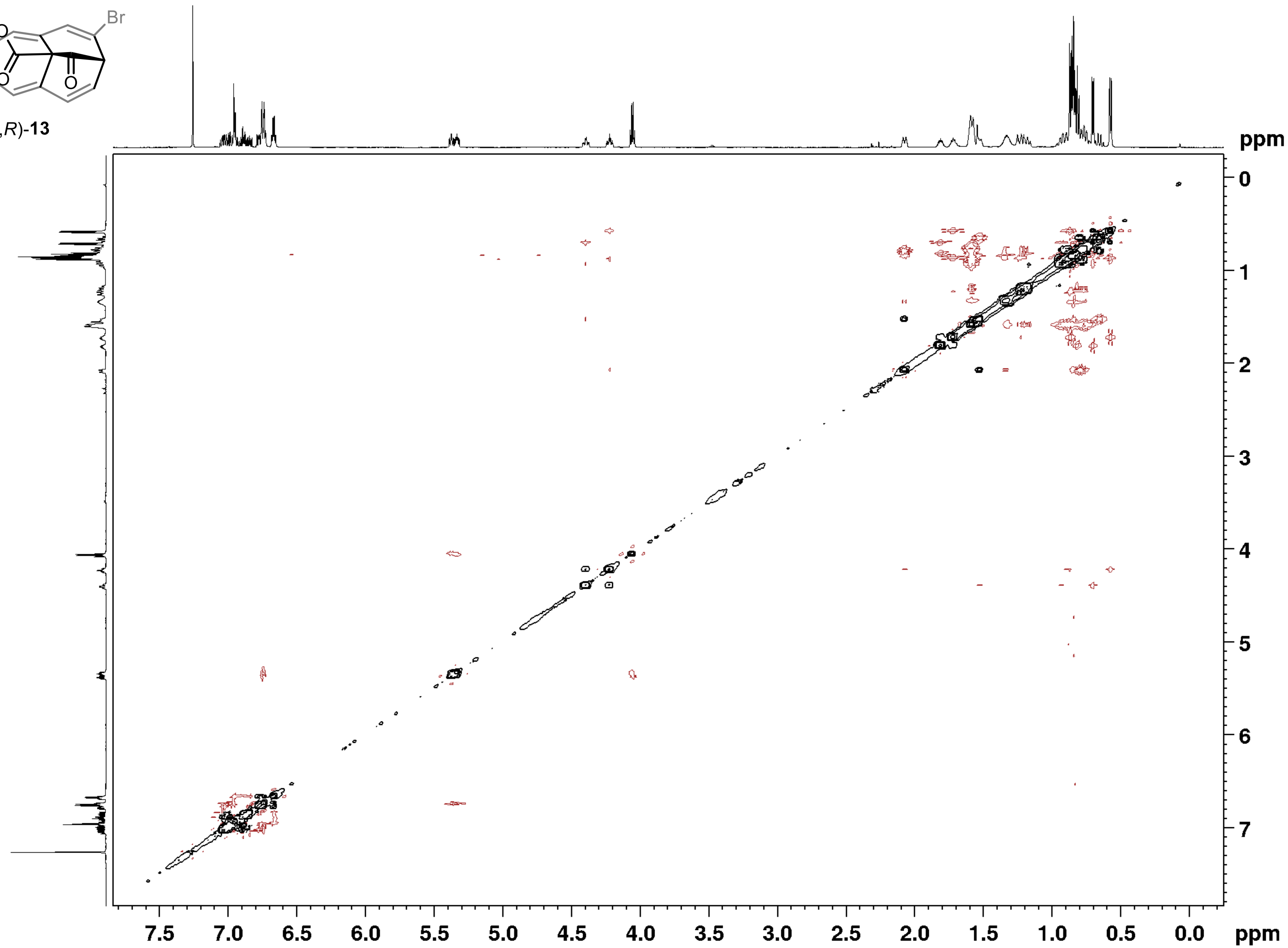

Figure S116: ^{1}H, ^{1}H NOESY spectrum of (L)-**13**.

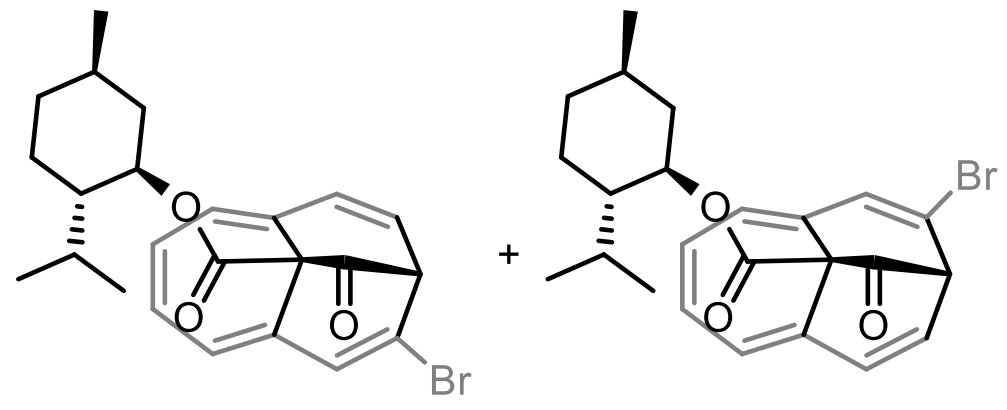

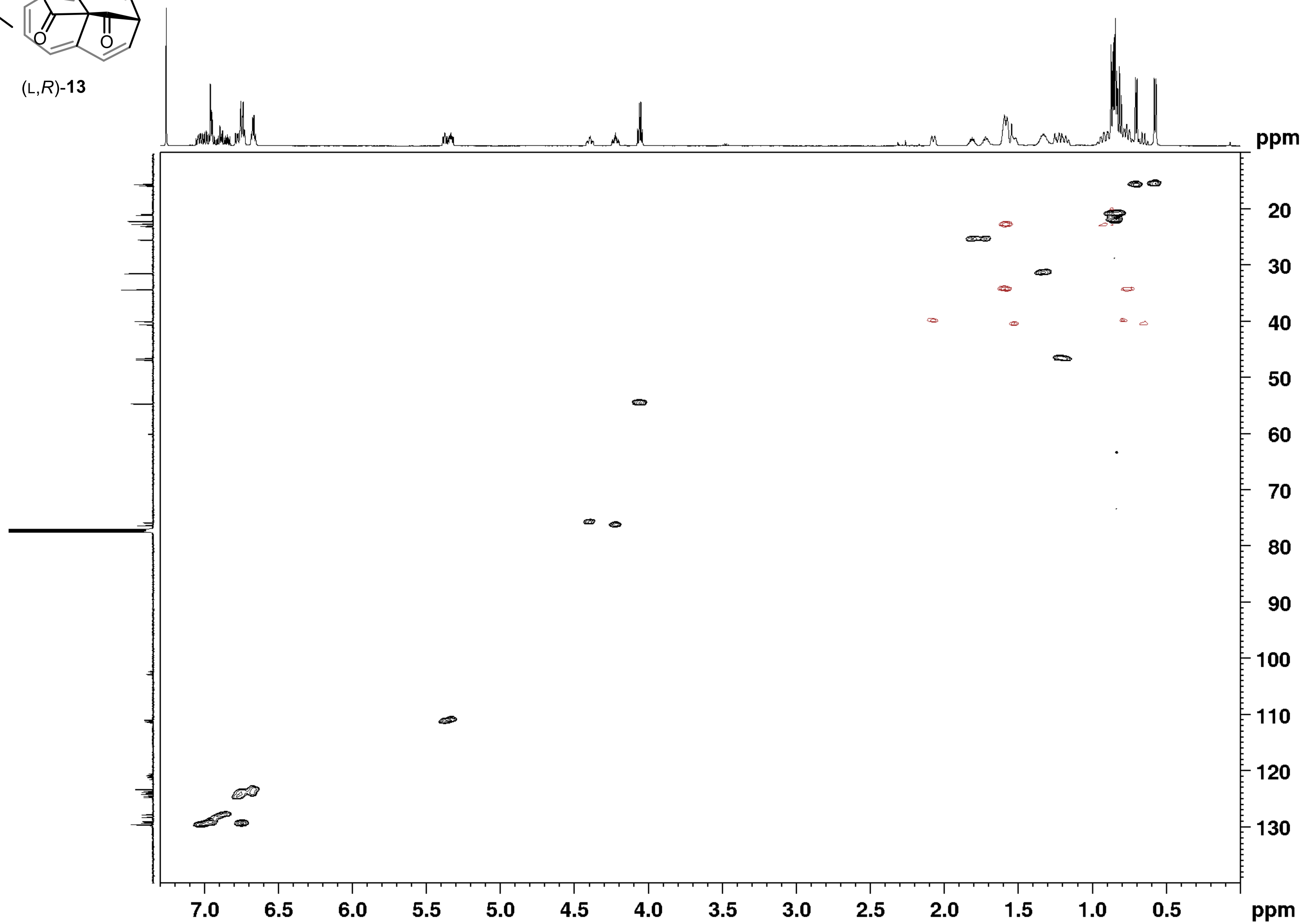

Figure S117: ^{1}H, ^{13}C HSQC spectrum of (L)-**13**.

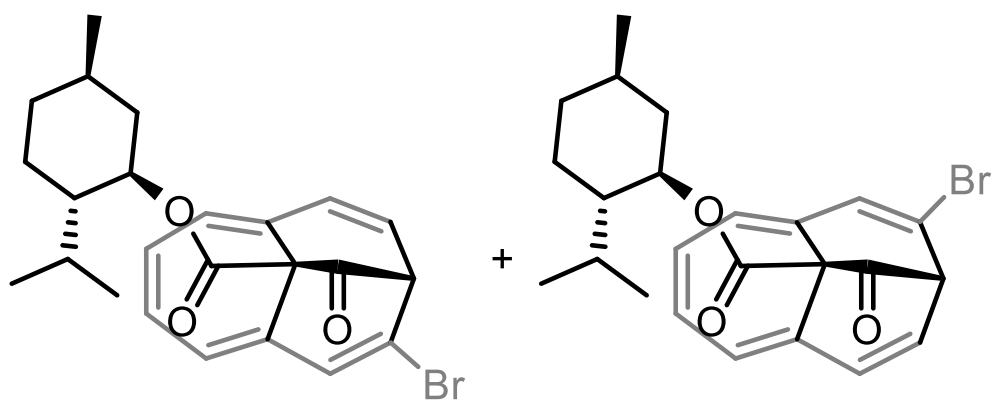

(L,*S*)-**13** (L,*R*)-**13**

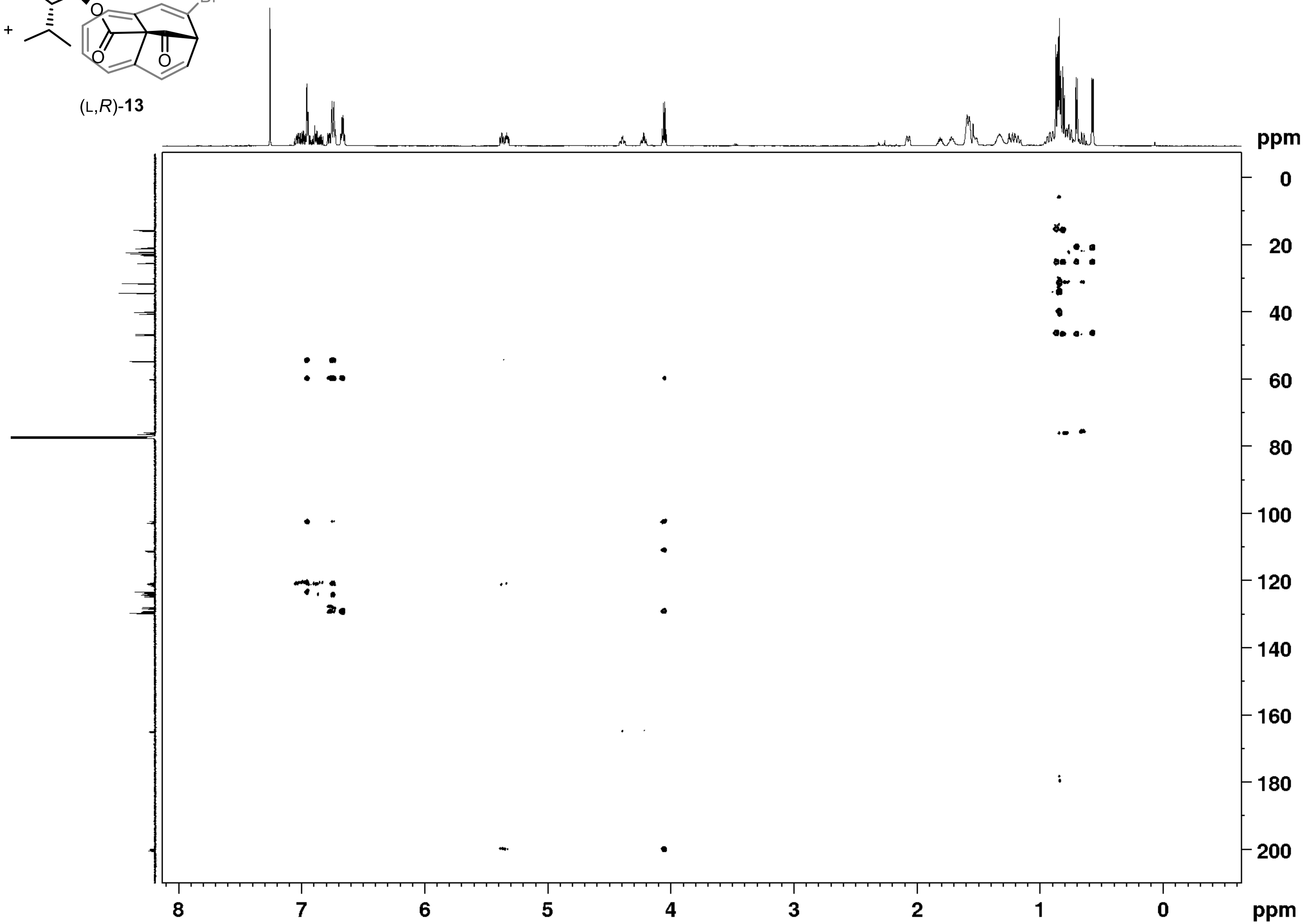

Figure S118: ^{1}H, ^{13}C HMBC spectrum of (L)-**13**.

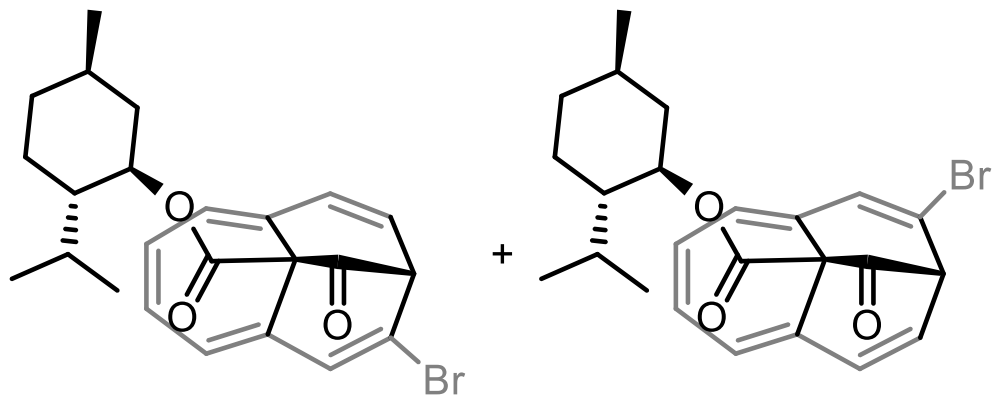

(L,*S*)-**13** (L,*R*)-**13**

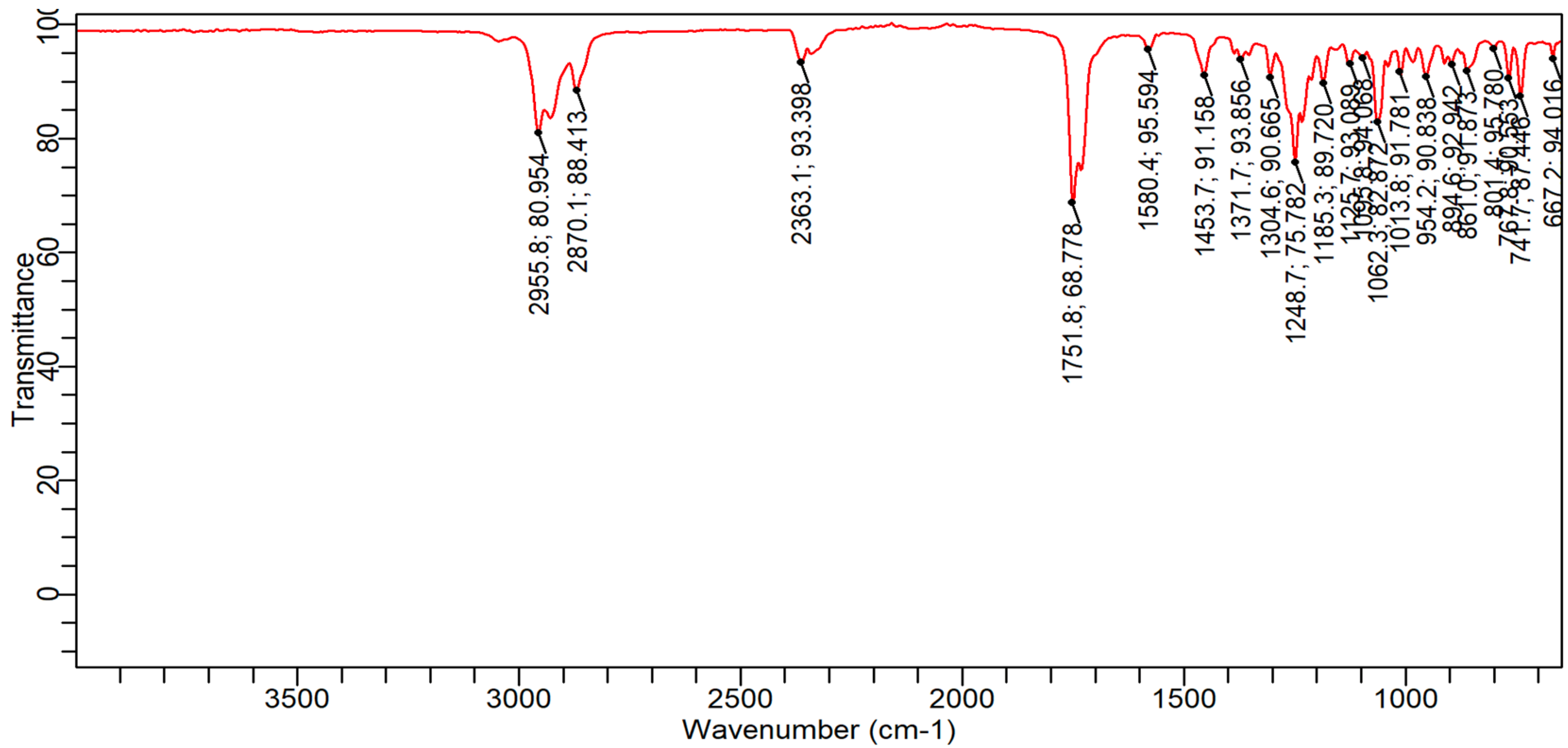

Figure S119: IR spectrum of (L)-**13**.

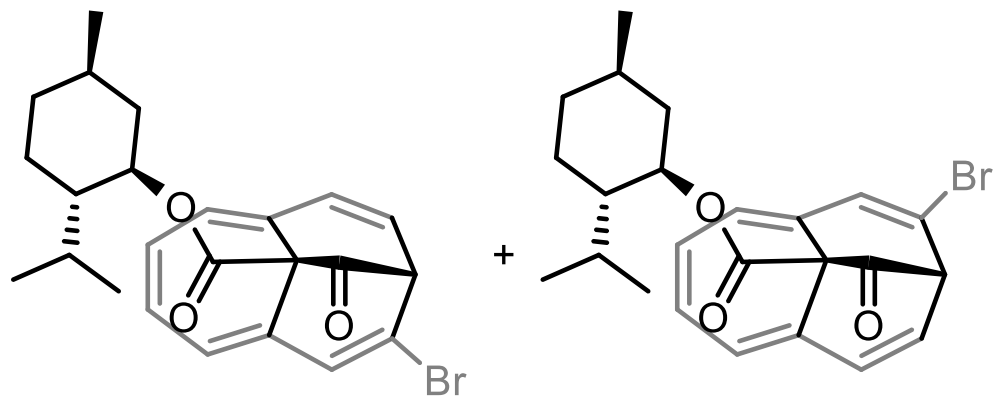

(L,*S*)-**13** (L,*R*)-**13**

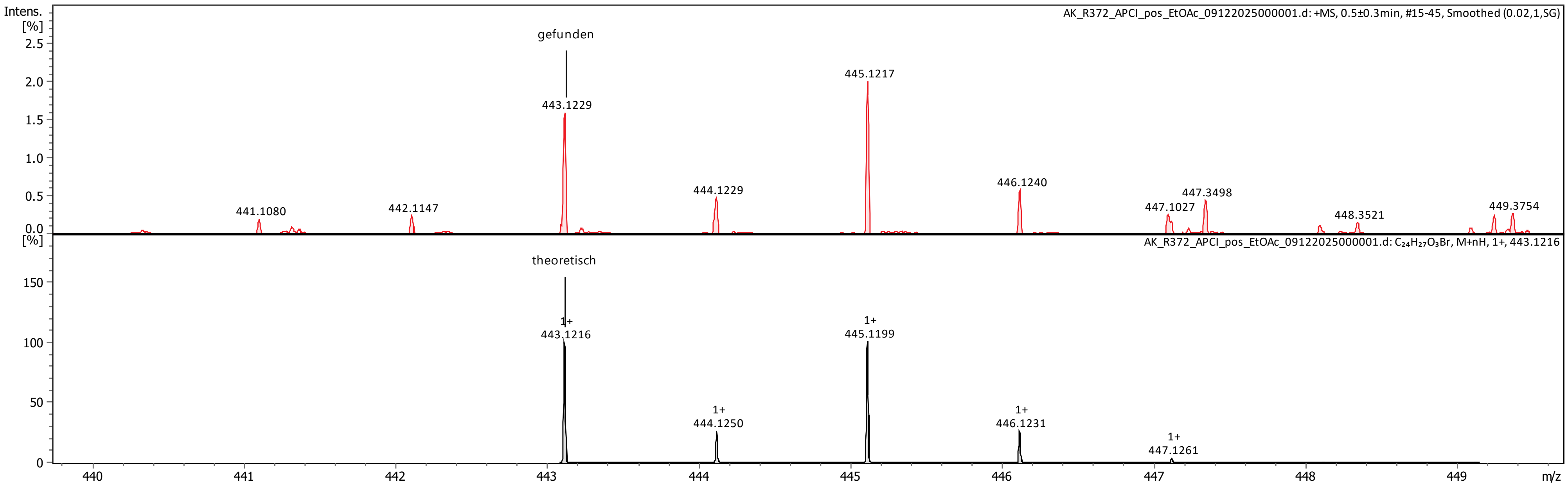

Figure S120: HRMS spectrum of (L)-**13**.

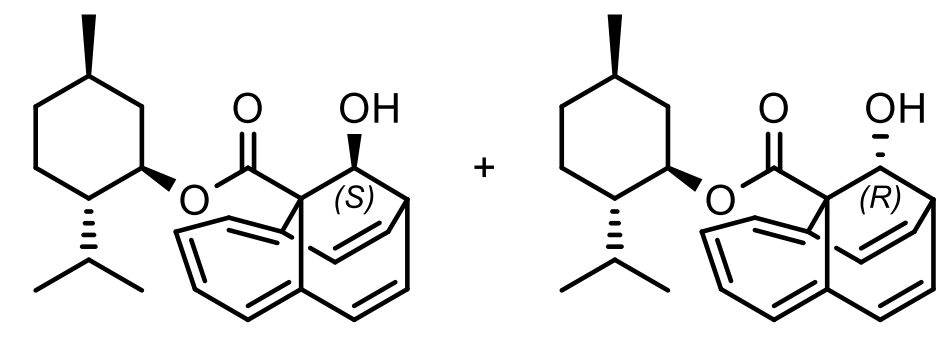

(L,*S*)-**14** (L,*R*)-**14**

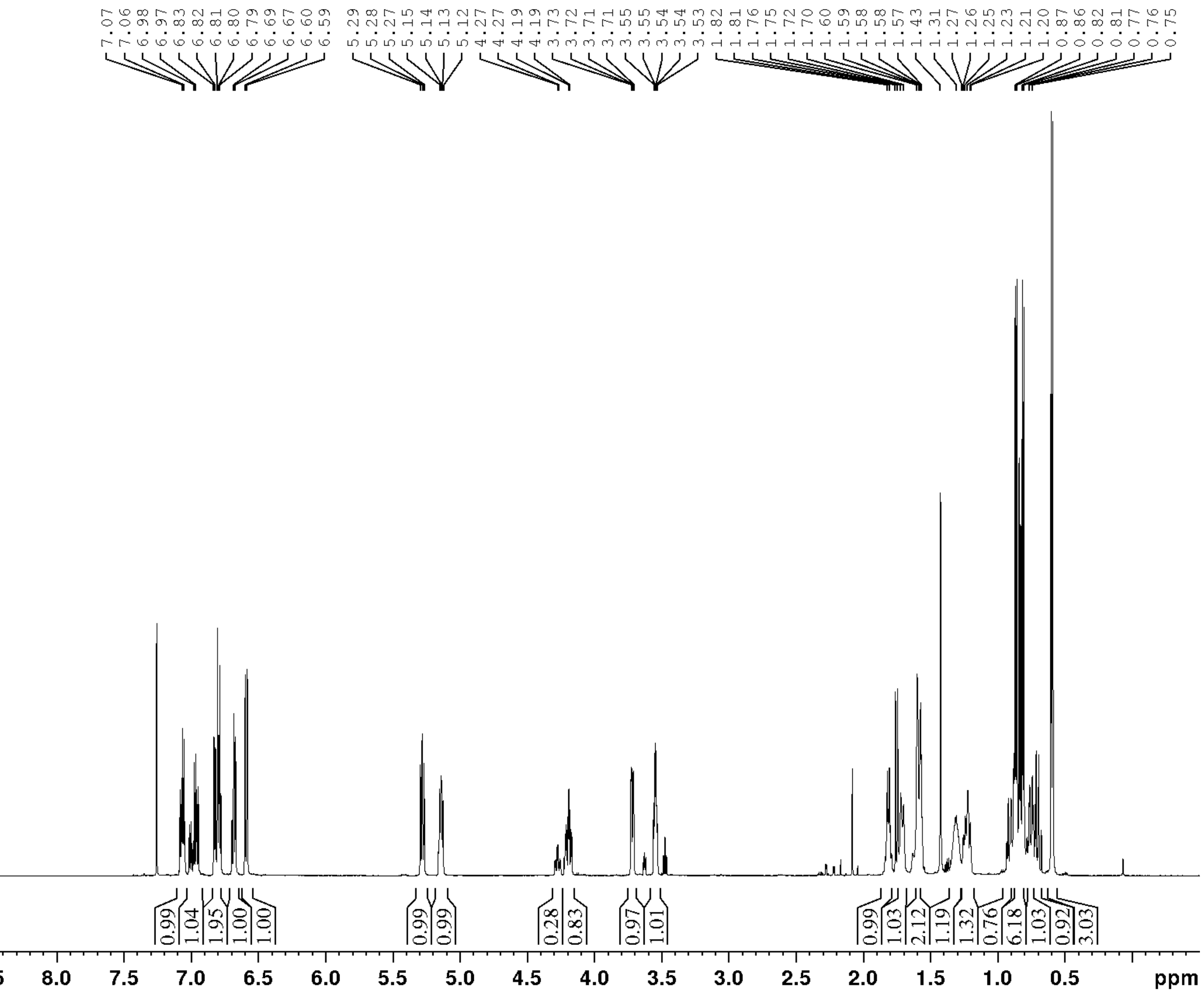

Figure S121: ^{1}H NMR spectrum of (L)-**14**.

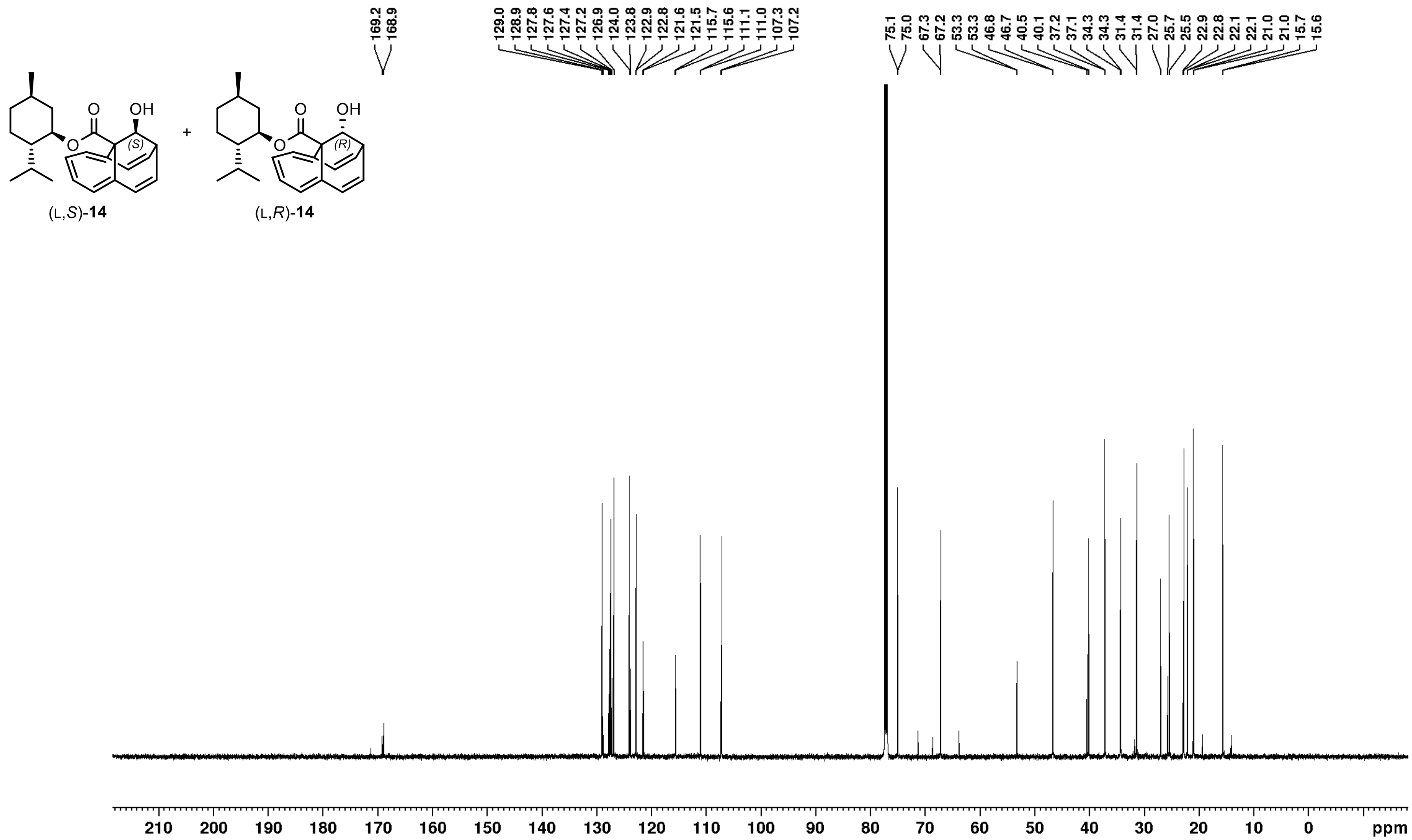

Figure S122: ^{13}C NMR spectrum of (L)-**14**.

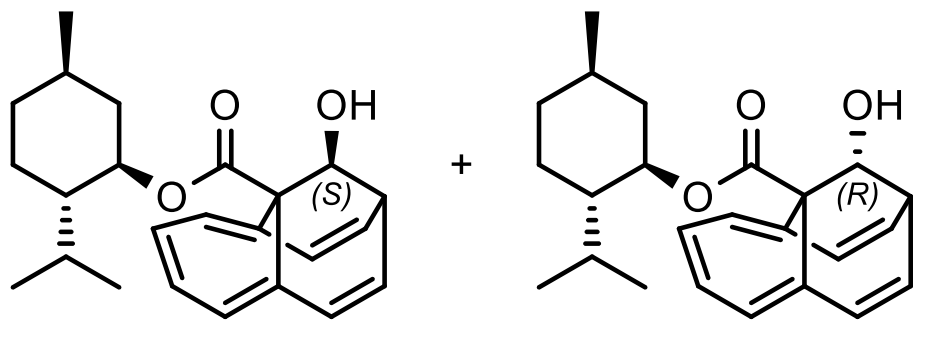

(L,*S*)-**14** + (L,*R*)-**14**

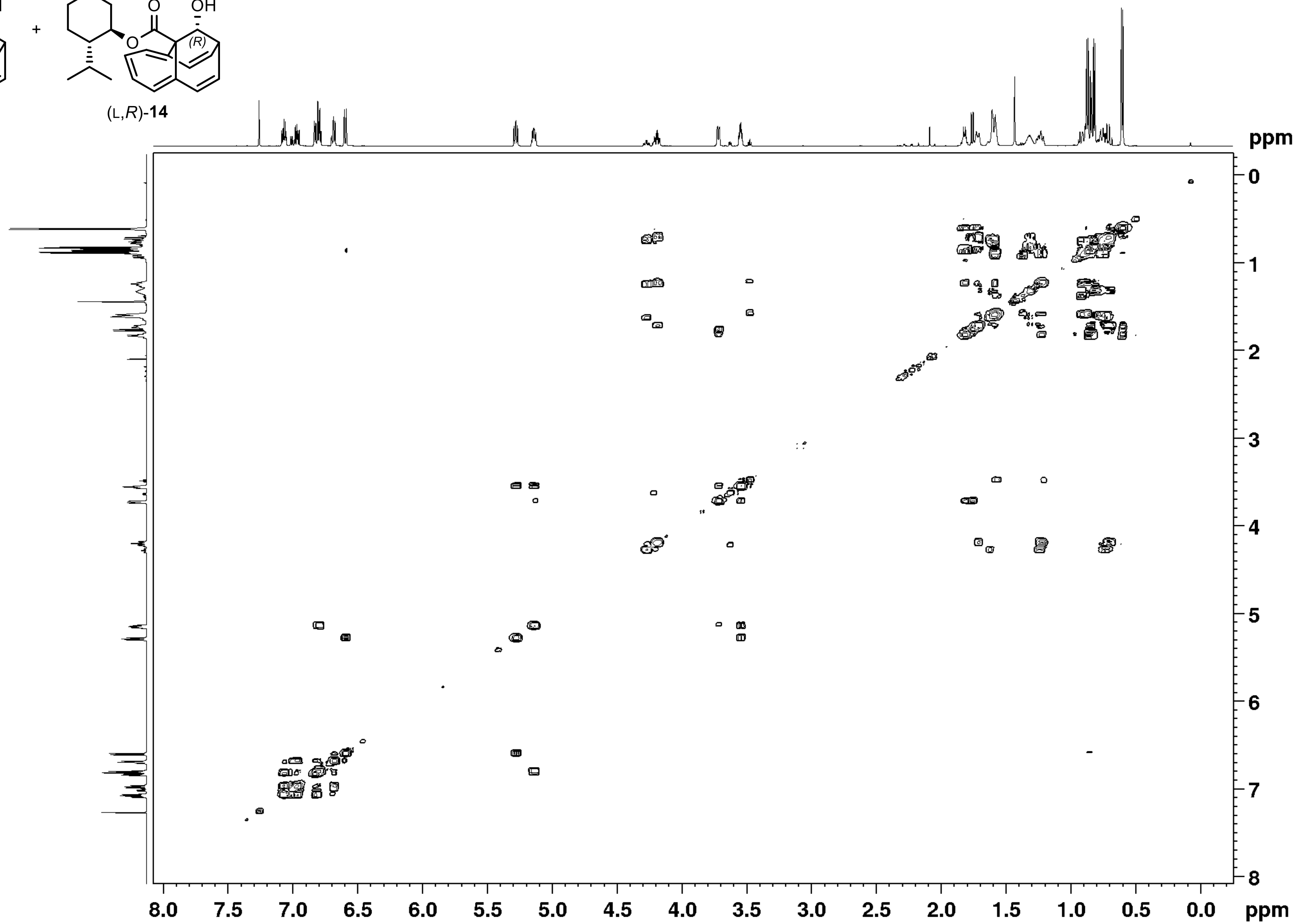

Figure S123:^{1}H, ^{1}H, COSY spectrum of (L)-**14**.

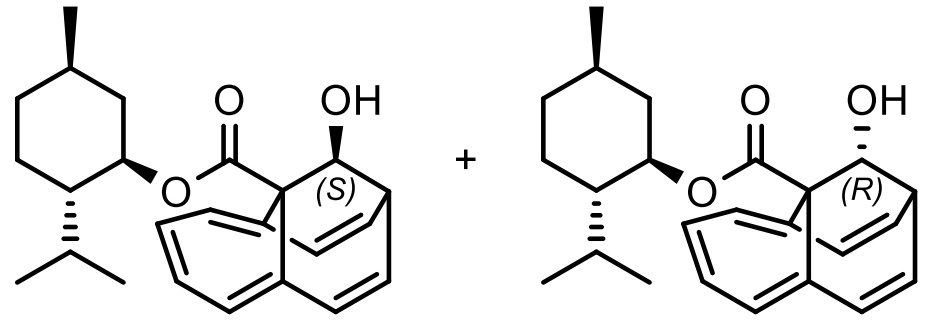

(L,S)-**14** + (L,R)-**14**

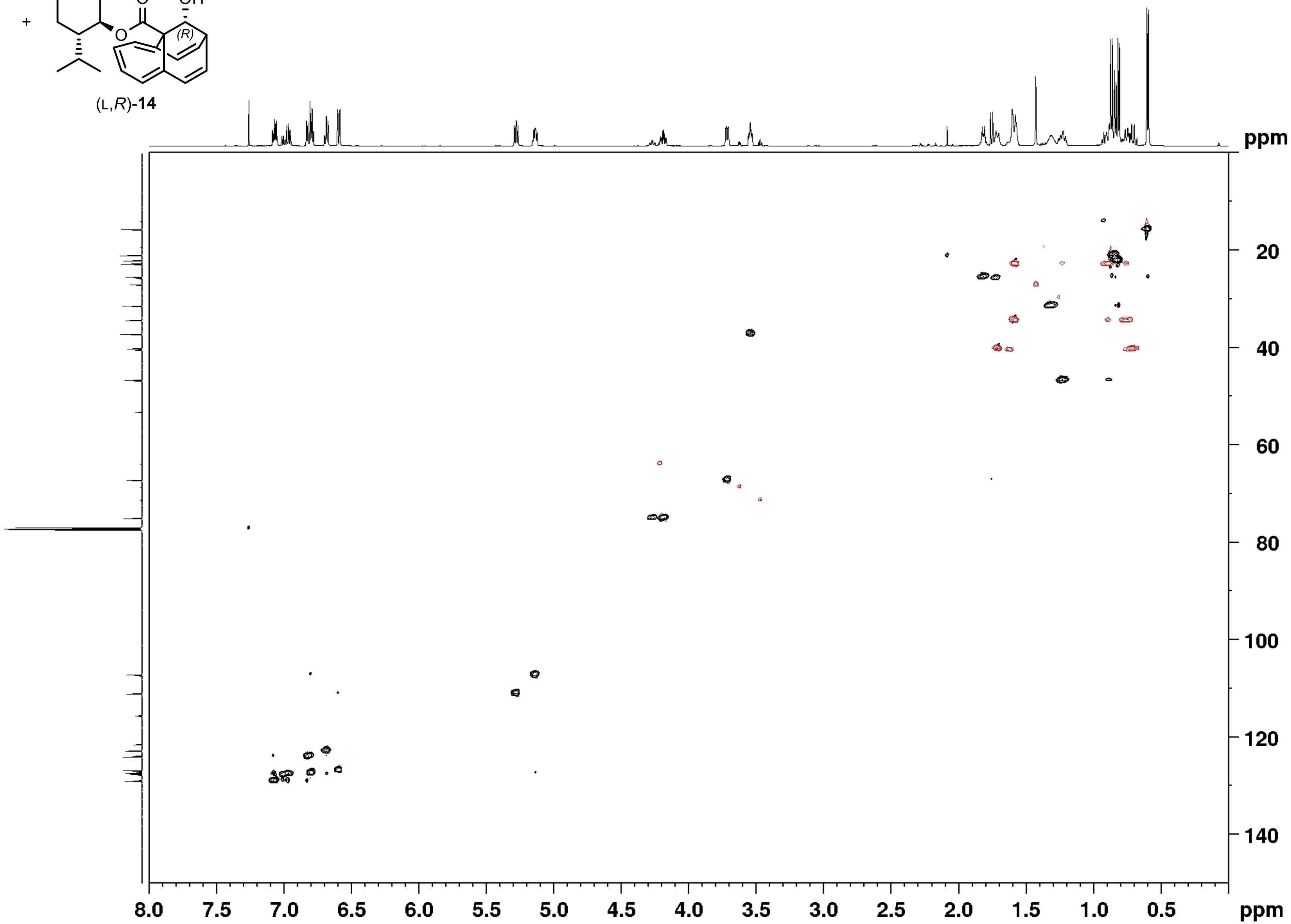

Figure S124: ^{1}H, ^{13}C HSQC spectrum of (L)-**14**.

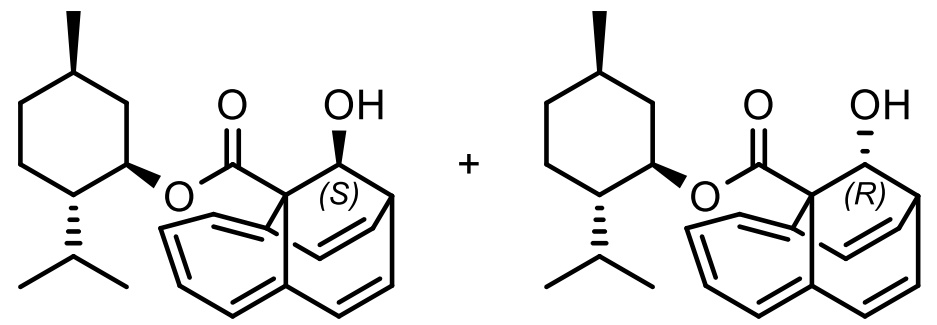

(L,*S*)-**14** (L,*R*)-**14**

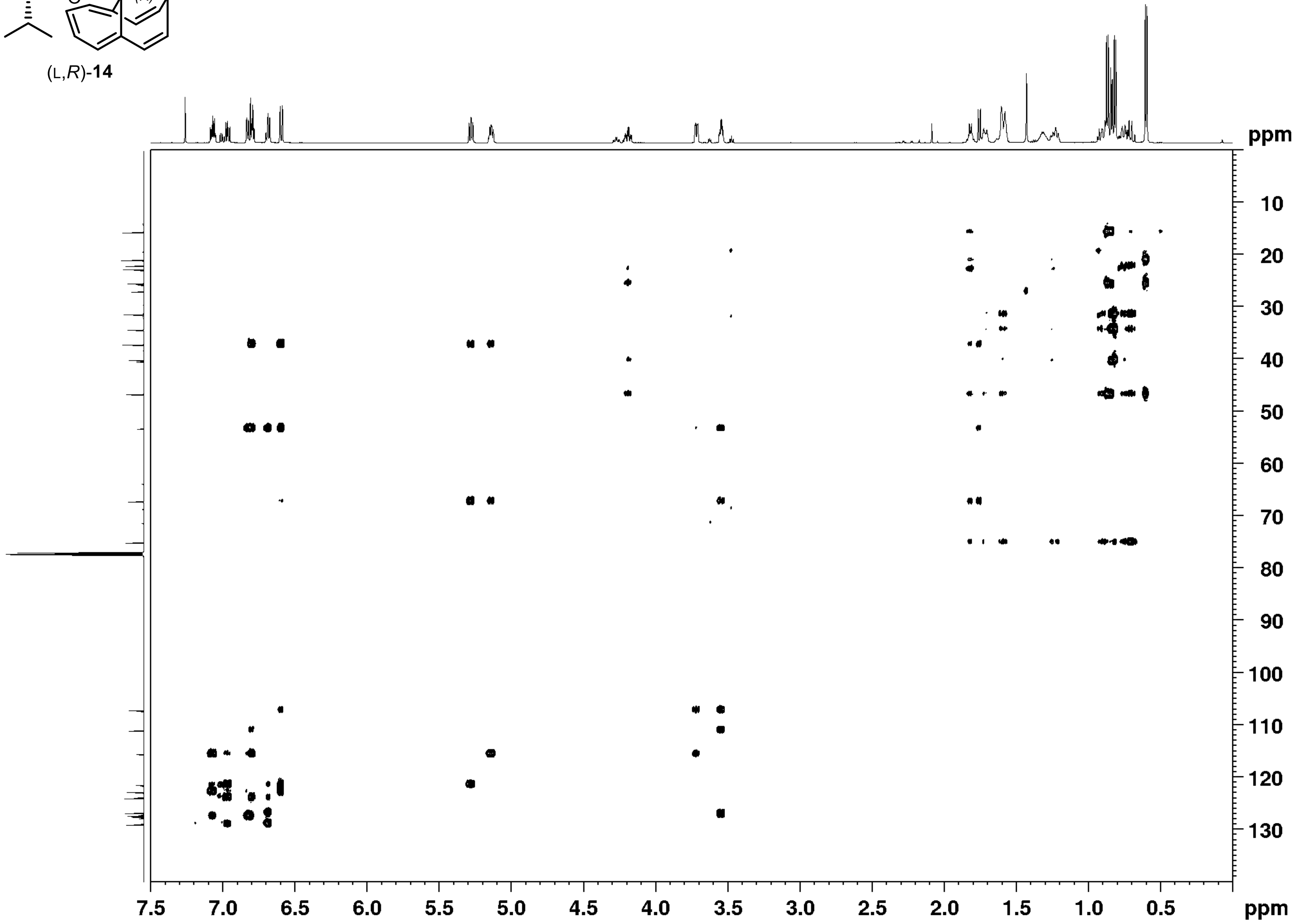

Figure S125: ^{1}H, ^{13}C HMBC spectrum of (L)-**14**.

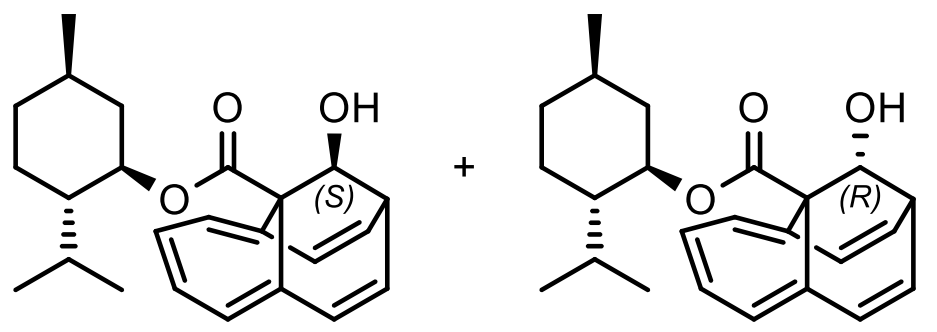

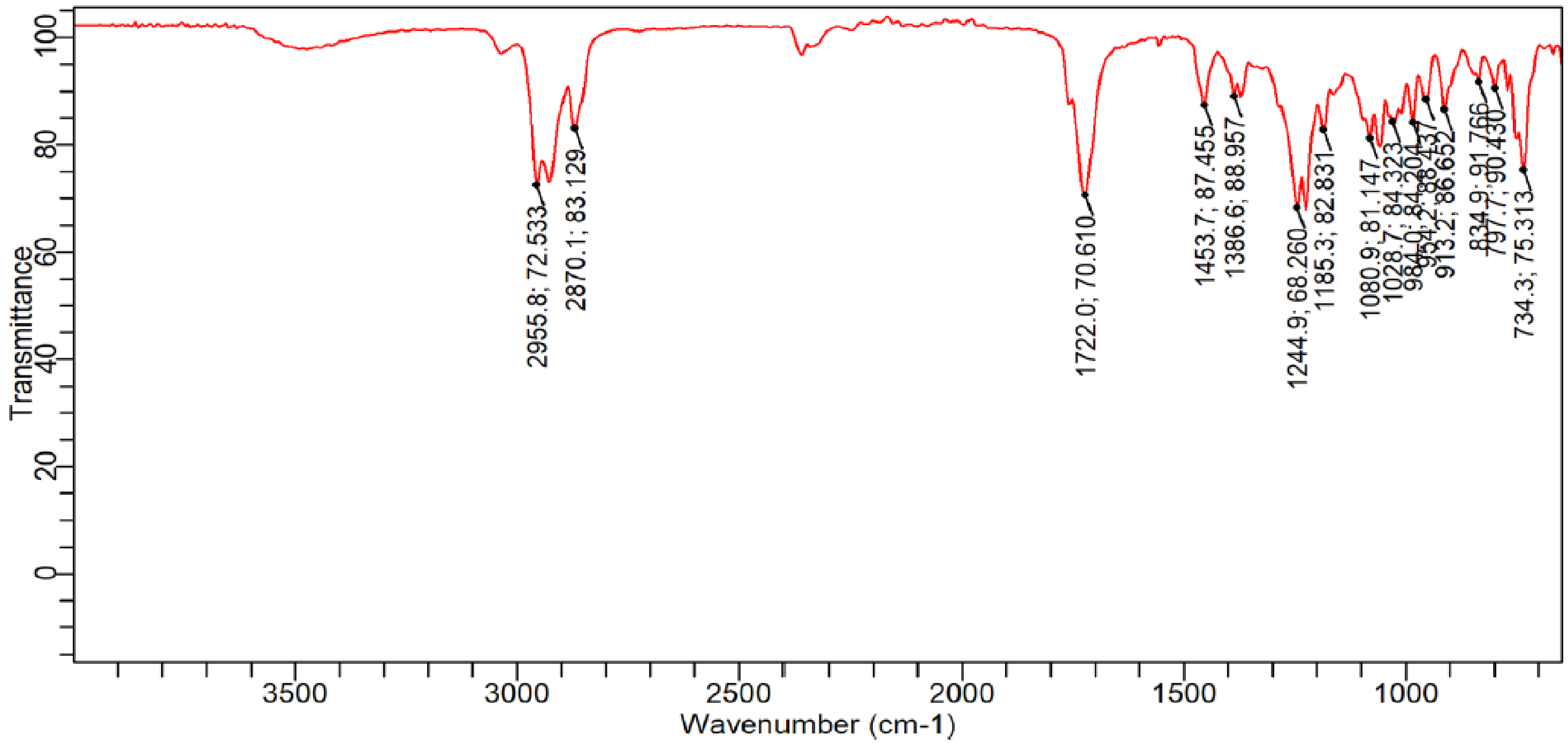

Figure S126: IR spectrum of (L)-**14**.

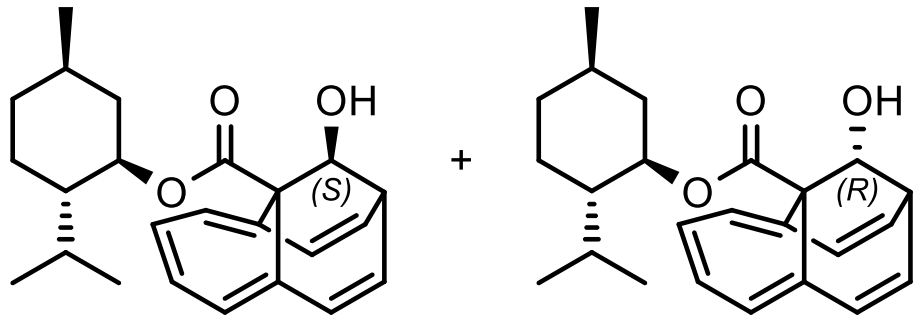

(L,*S*)-**14** (L,*R*)-**14**

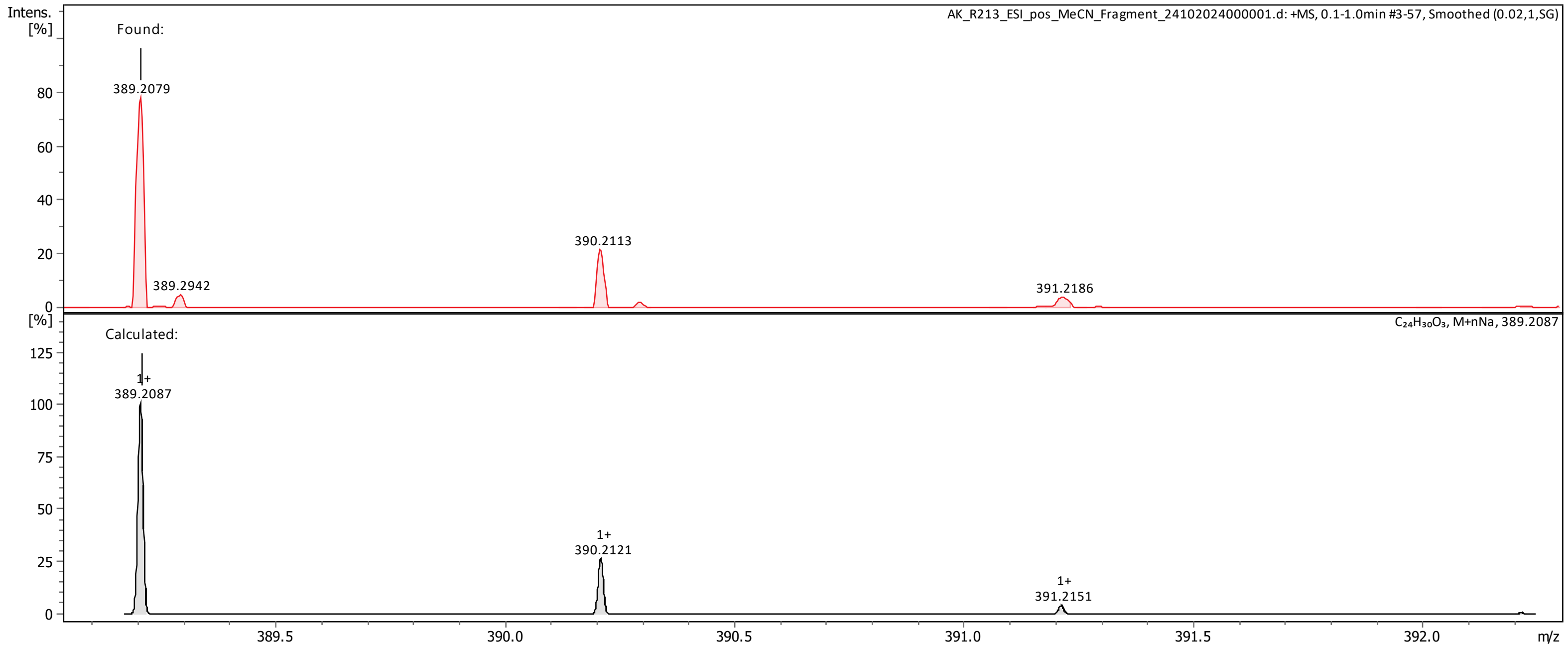

Figure S127: HRMS spectrum of (L)-**14**.

3. Chromatograms and additional HPLC data

Naphthalene calibration curve

Naphthalene solutions were prepared in MeCN and subjected to HPLC analysis on CHIRALPAK AD-H column. As eluent *n*-heptane/*i*PrOH = 90:10 with a flow rate of 0.4 mL · min^{-1} was used. Naphthalene was detected at a retention time of t = 8.4 min (see chromatograms below)

Table S3. concentration of naphthalene solutions in MeCN which were used for the calibration curve.

Entry	c [mol/L]	Area [mAU s]
1	0.00195	2437.232
2	0.00398	5044.042
3	0.00609	7240.297
4	0.00796	9668.03
5	0.00991	11849.103

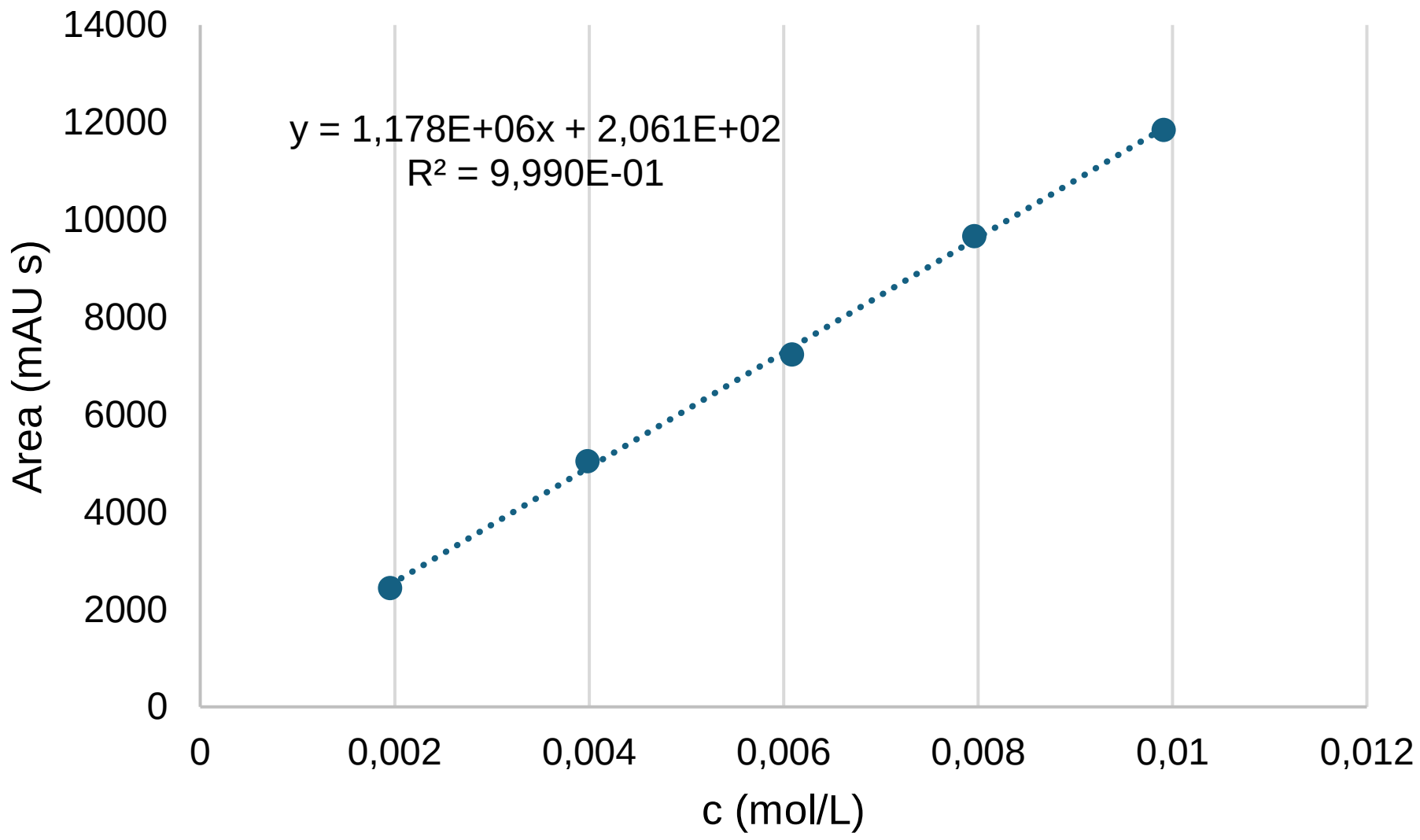

Figure S3.1 Calibration curve for naphthalene in MeCN. CHIRALPAK AD-H was used with *n*-heptane/*i*PrOH = 90:10 and a flow rate of 0.4 mL · min^{-1} at 40 °C.

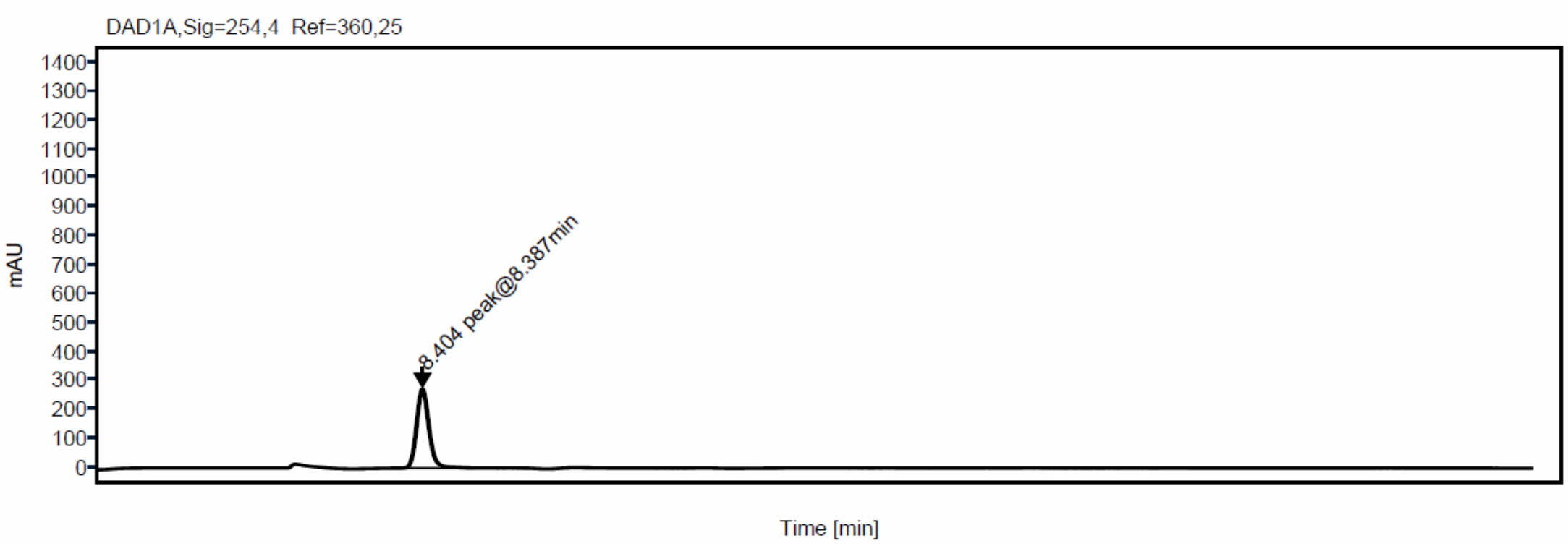

Signal:	DAD1A,Sig=254,4 Ref=360,25		
**Name**	**RT [min]**	**Area**	**Peak Area Percent**
peak@8.387min	8.40	2437.232	100.00

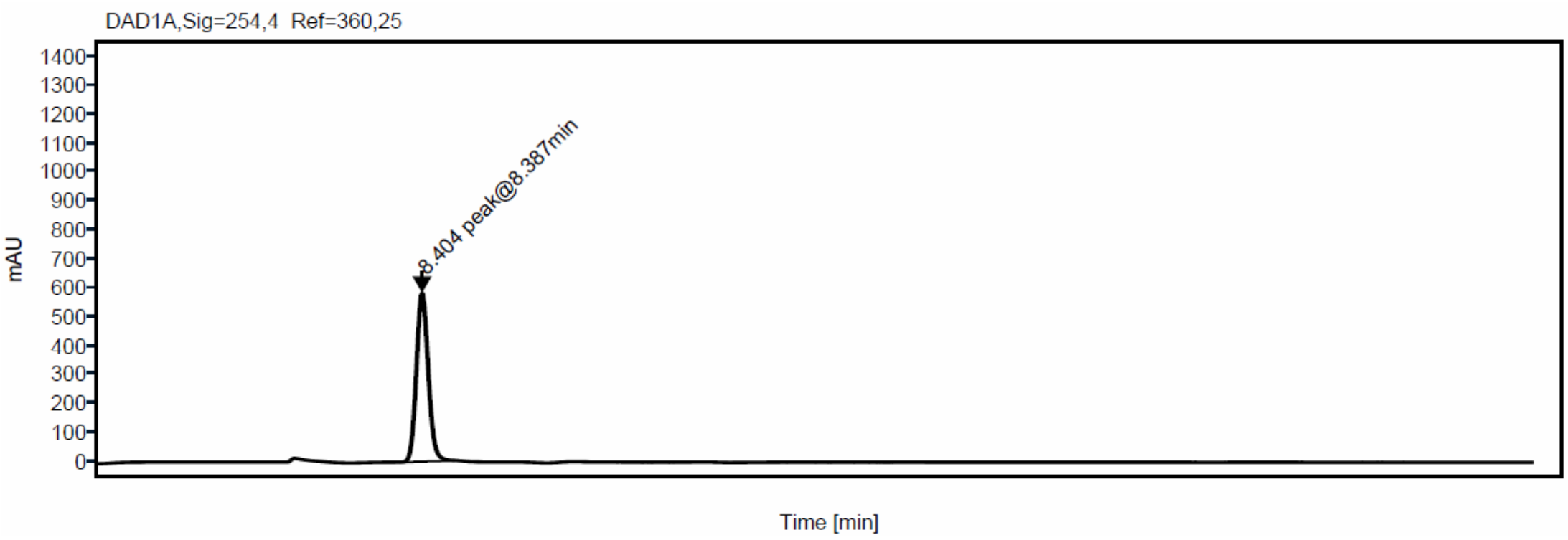

Signal:	DAD1A,Sig=254,4 Ref=360,25		
**Name**	**RT [min]**	**Area**	**Peak Area Percent**
peak@8.387min	8.40	5044.042	100.00

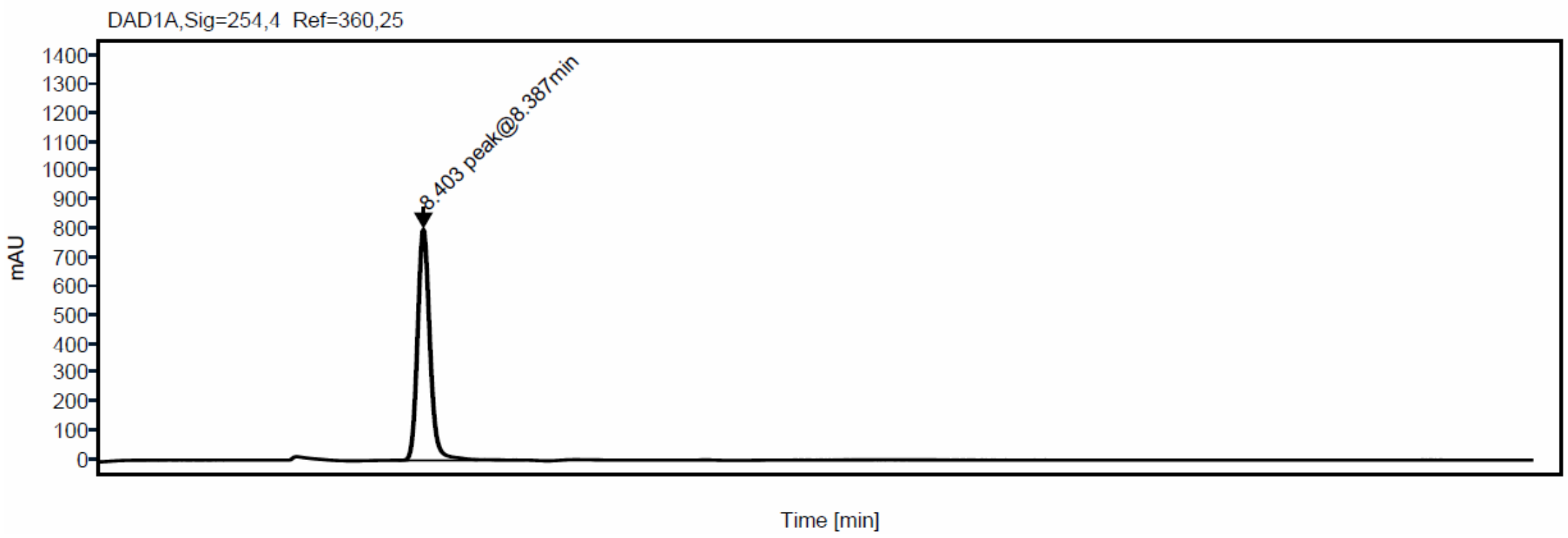

Signal:	DAD1A,Sig=254,4 Ref=360,25		
**Name**	**RT [min]**	**Area**	**Peak Area Percent**
peak@8.387min	8.40	7240.297	100.00

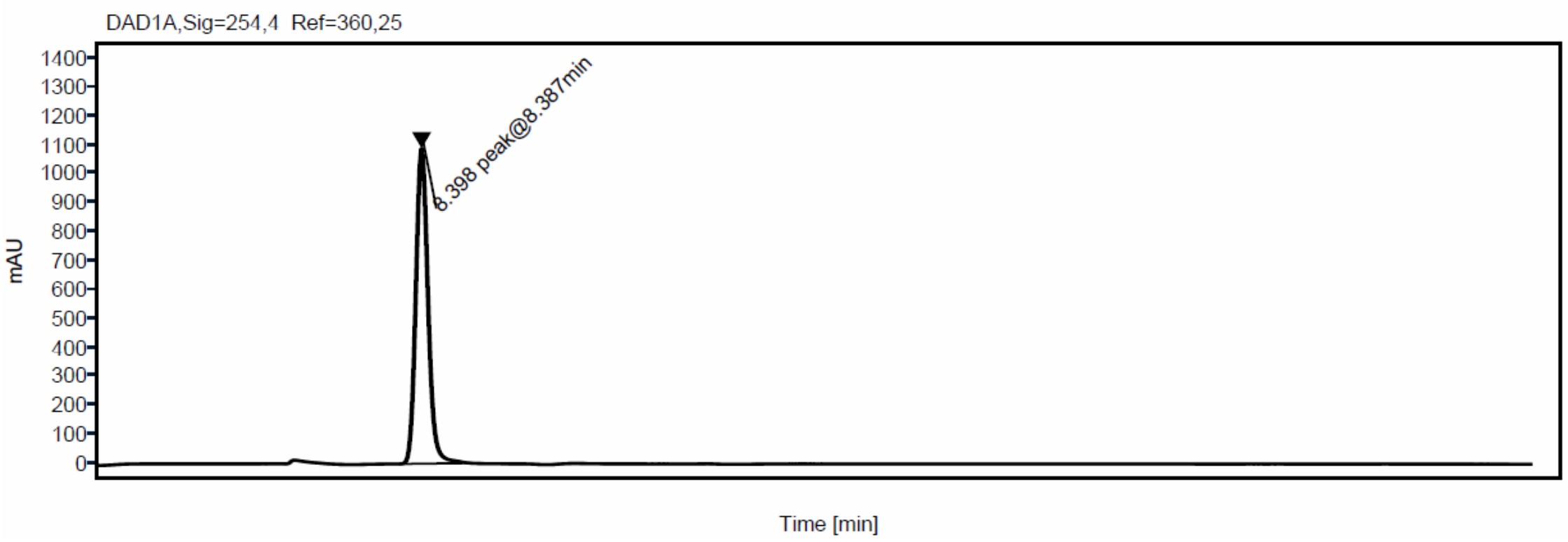

Signal: DAD1A,Sig=254,4 Ref=360,25

Name	RT [min]	Area	Peak Area Percent
peak@8.387min	8.40	9668.030	100.00

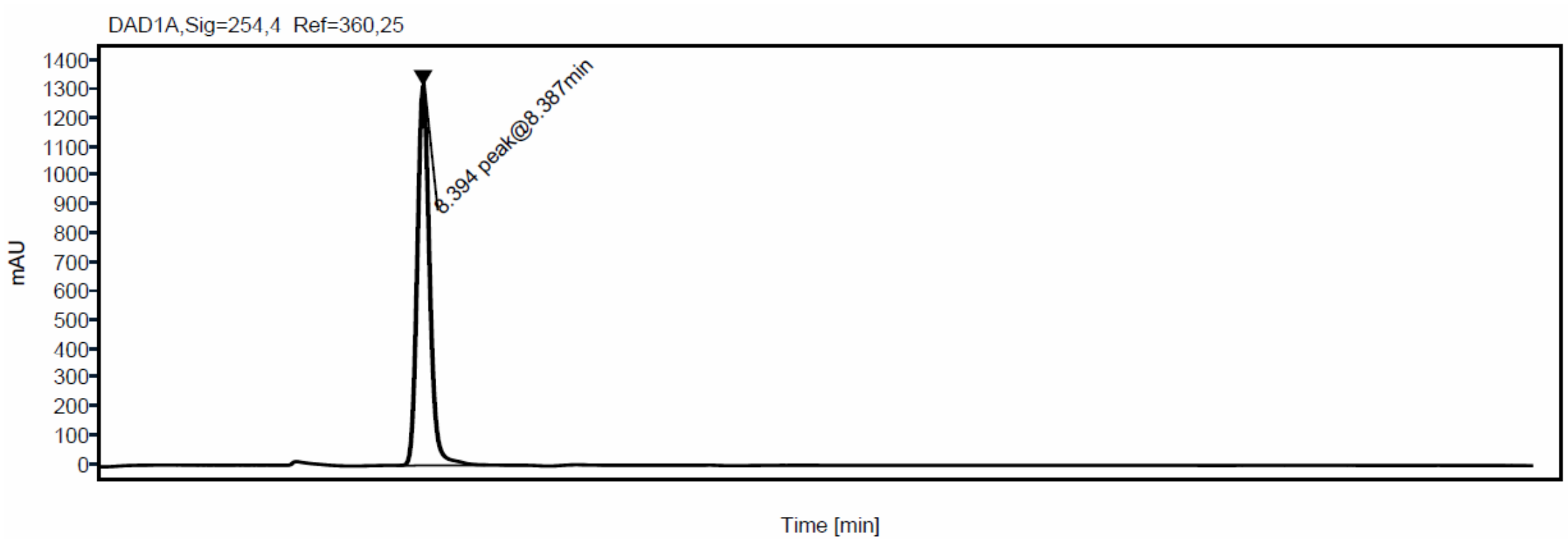

Signal: DAD1A,Sig=254,4 Ref=360,25

Name	RT [min]	Area	Peak Area Percent
peak@8.387min	8.39	11849.103	100.00

Irradiation of homoannulene (L)-6 at 280 and 430 nm ("*photoswitching in small scale*")

To investigate the development of ratio of homoannulene (L)-**6** to *iso*homoannulene (L)-**7** as well as the ratio of the two formed *iso*-homoannulenes (L,*R*)-**7** to (L,*S*)-**7** as a function of irradiation time until $PSS_{280\ nm}$ (forward switching process, Equation S2) and $PSS_{430\ nm}$ (backward switching process, Equation S3) samples were taken and subjected to HPLC analysis on a chiral stationary phase. For the HPLC analysis CHIRALPAK AD-H with *n*-heptane/*i*PrOH = 90:10, a flowrate of 0.4 mL · min^{-1} at 40 °C was used. Additionally, naphthalene was used as an internal standard, which was detected at t = 8.4 min.

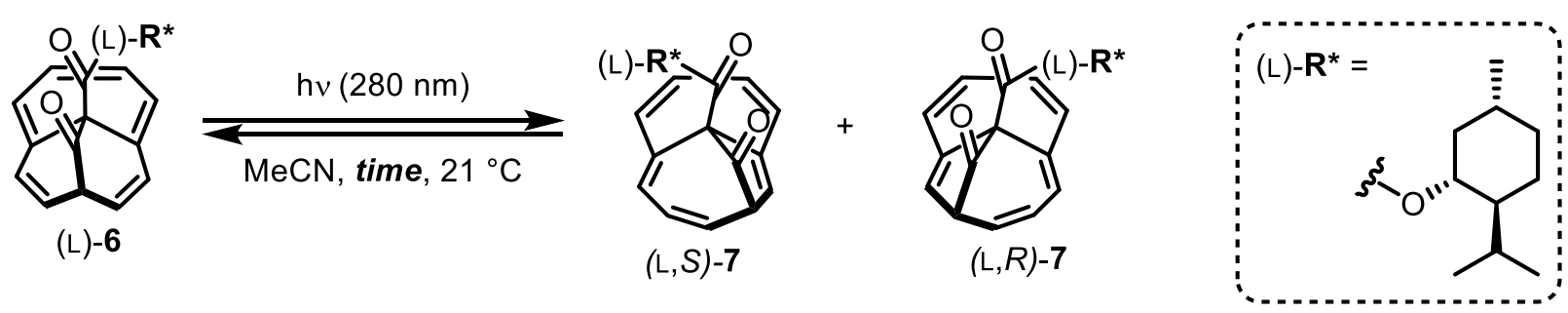

Equation S2. Photoswitching of homoannulene (L)-**6** using an LED of λ = 280 nm.

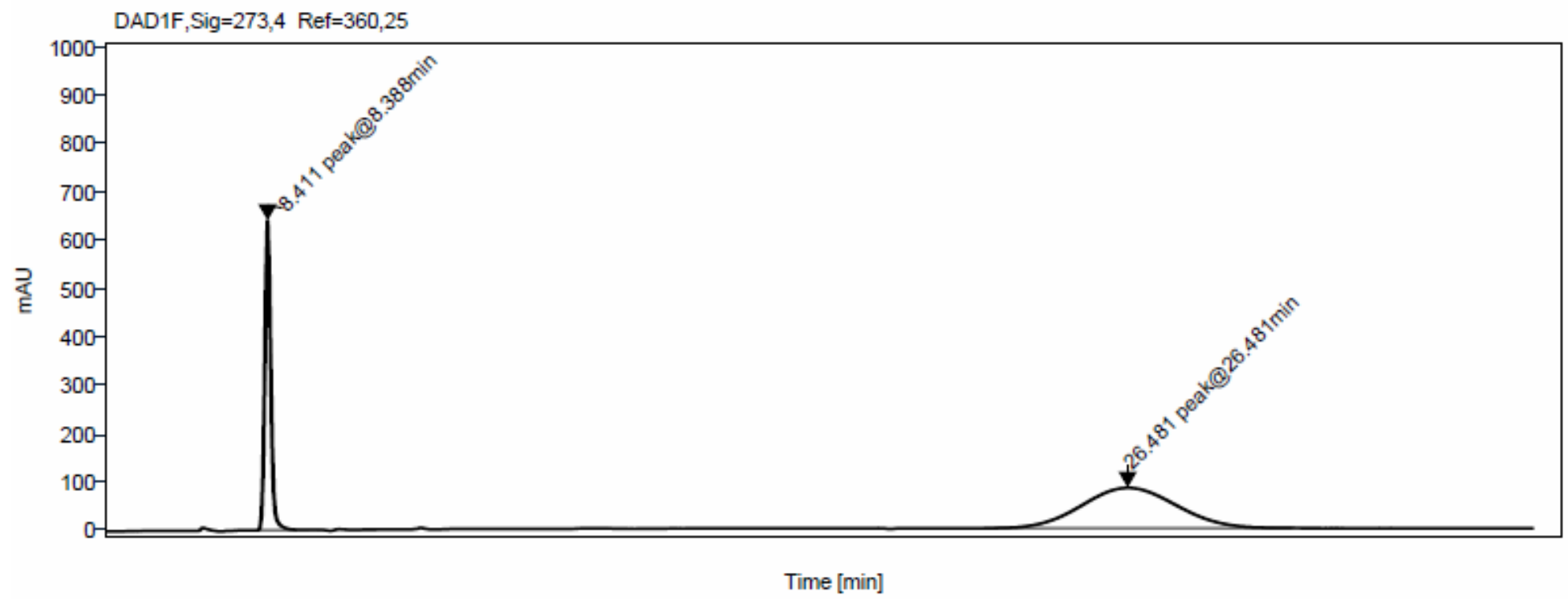

Signal:	DAD1F,Sig=273,4 Ref=360,25		
**Name**	**RT [min]**	**Area**	**Peak Area Percent**
peak@8.388min	8.41	5557.961	32.48
peak@26.481min	26.48	11555.301	67.52

Figure S3.2. Chromatogram of homoannulene (L)-**6** prior to photoswitching.

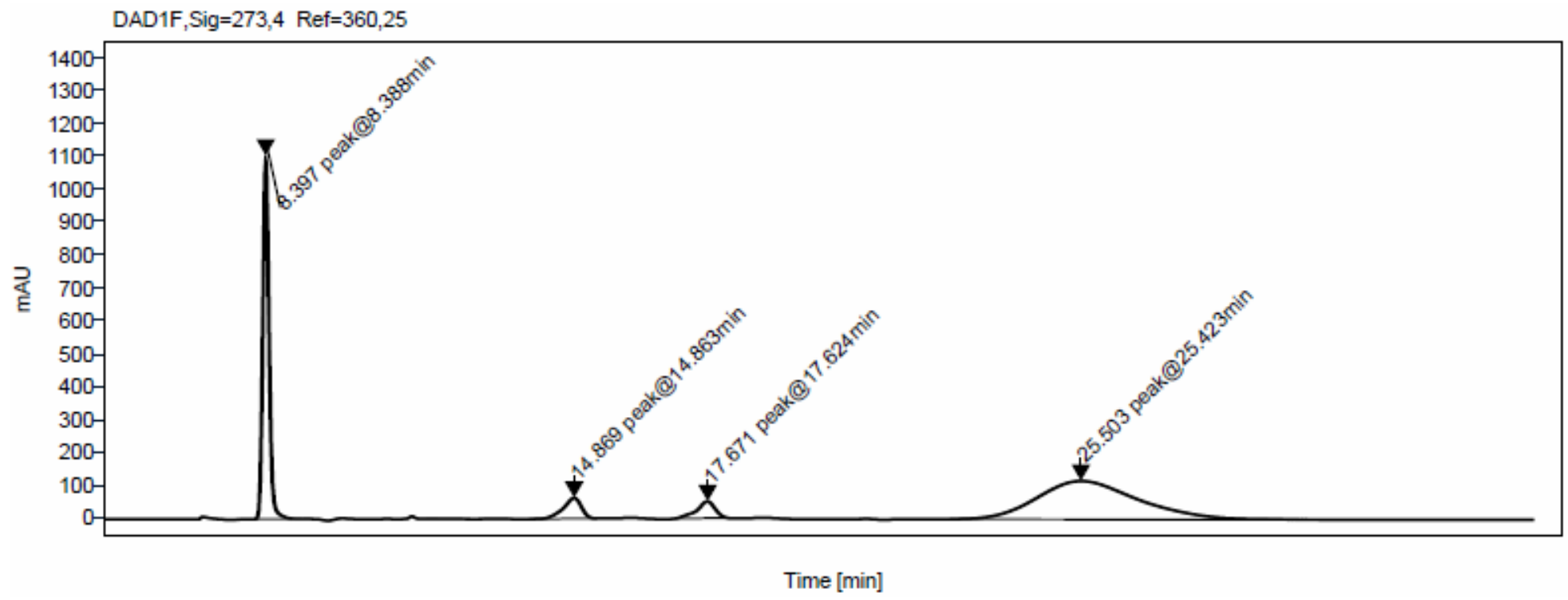

Signal:	DAD1F,Sig=273,4 Ref=360,25		
**Name**	**RT [min]**	**Area**	**Peak Area Percent**
peak@8.388min	8.40	9941.769	33.22
peak@14.863min	14.87	1666.239	5.57
peak@17.624min	17.67	1376.510	4.60
peak@25.423min	25.50	16941.498	56.61

Figure S3.3. Chromatogram of irradiated sample of homoannulene (L)-**6** after 30 min of irradiation at λ = 280 nm.

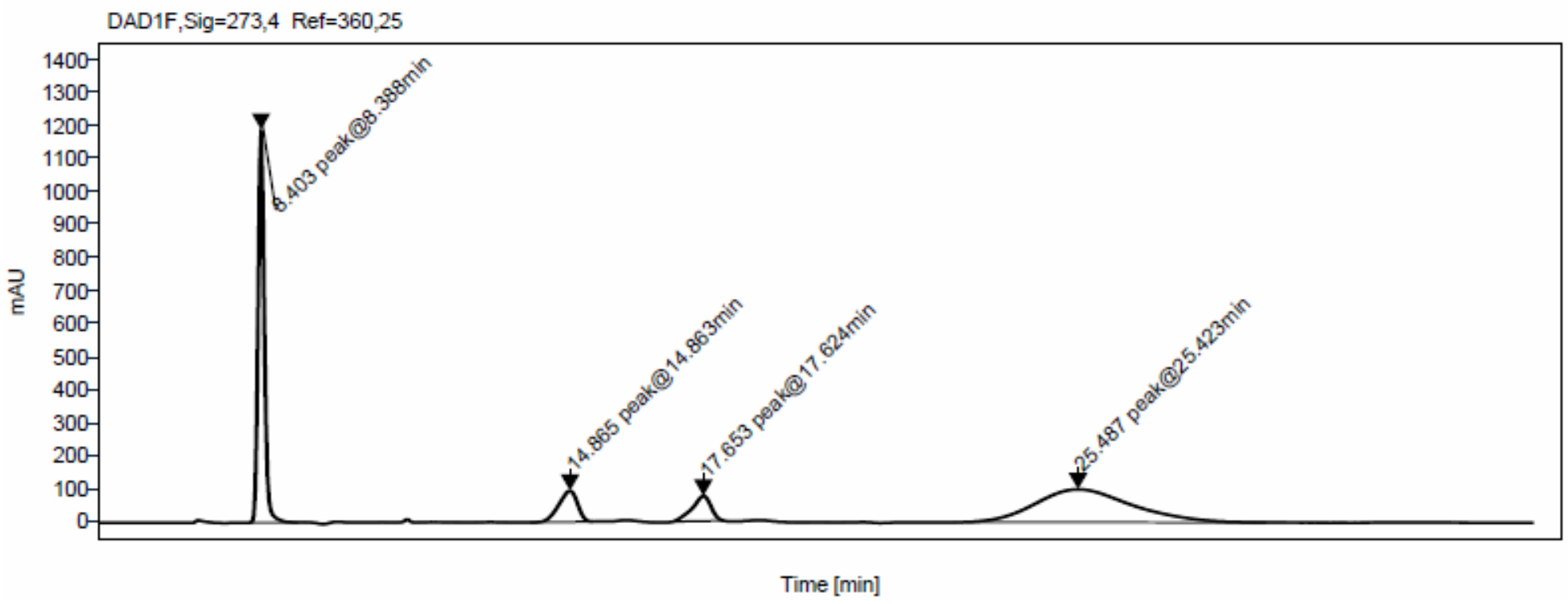

Signal: DAD1F,Sig=273,4 Ref=360,25

Name	RT [min]	Area	Peak Area Percent
peak@8.388min	8.40	10795.970	36.70
peak@14.863min	14.87	2576.247	8.76
peak@17.624min	17.65	2073.081	7.05
peak@25.423min	25.49	13969.211	47.49

Figure S3.4. Chromatogram of irradiated sample of homoannulene (L)-**6** after 60 min of irradiation at λ = 280 nm.

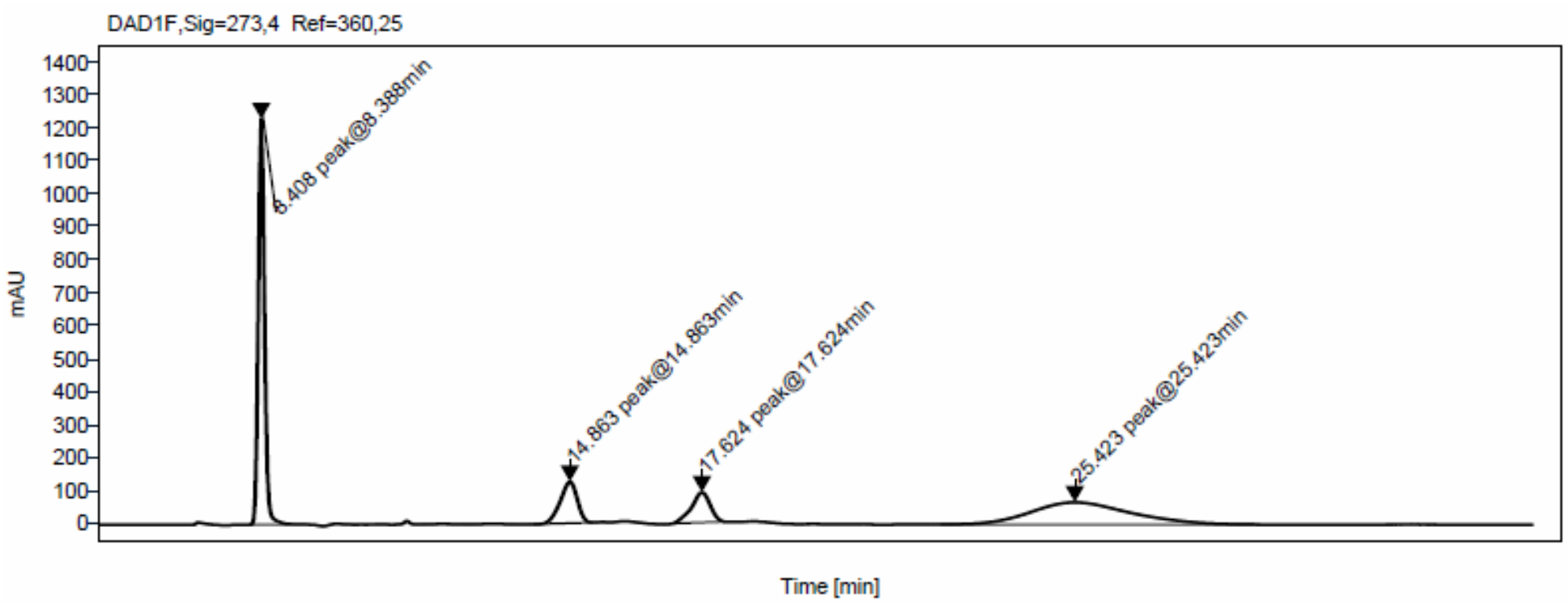

Signal: DAD1F,Sig=273,4 Ref=360,25

Name	RT [min]	Area	Peak Area Percent
peak@8.388min	8.41	11069.776	41.95
peak@14.863min	14.86	3102.609	11.76
peak@17.624min	17.62	2480.519	9.40
peak@25.423min	25.42	9737.143	36.90

Figure S3.5. Chromatogram of irradiated sample of homoannulene (L)-**6** after 120 min of irradiation at λ = 280 nm.

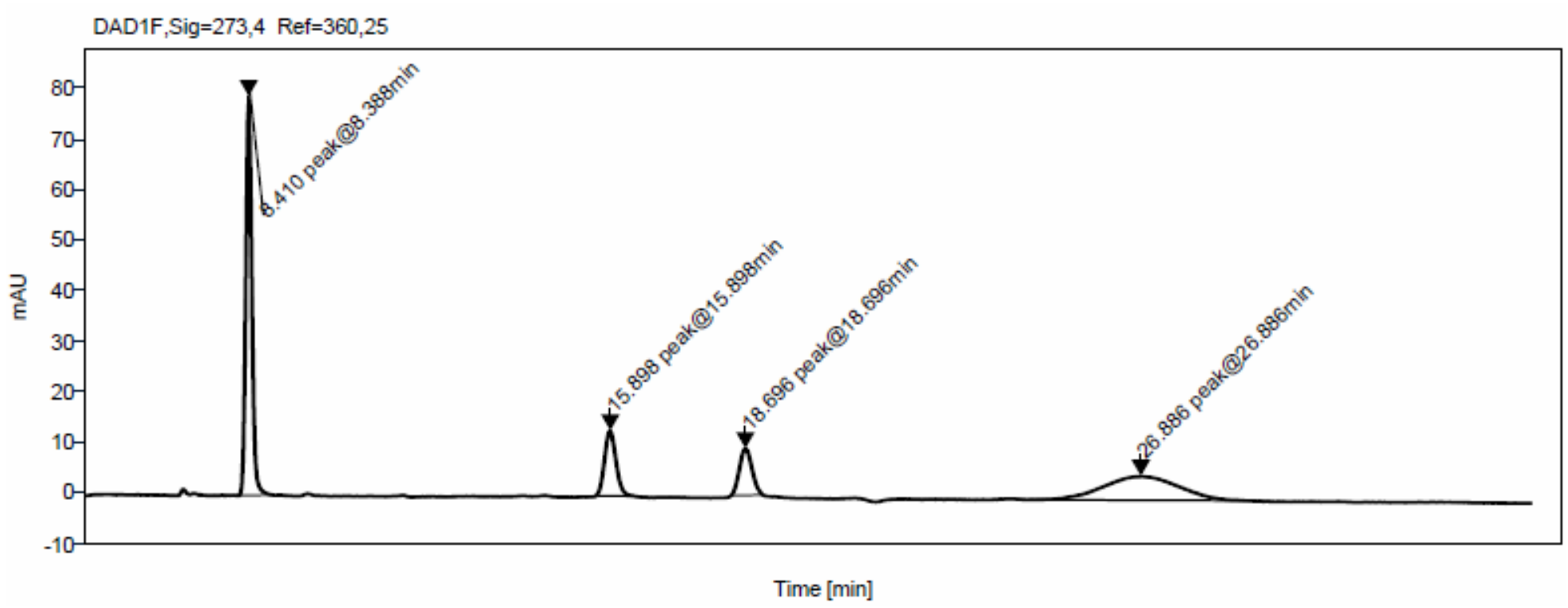

Signal: DAD1F,Sig=273,4 Ref=360,25

Name	RT [min]	Area	Peak Area Percent
peak@8.388min	8.41	684.237	43.43
peak@15.898min	15.90	211.351	13.41
peak@18.696min	18.70	166.953	10.60
peak@26.886min	26.89	512.991	32.56

Figure S3.6. Chromatogram of irradiated sample of homoannulene (L)-**6** after 180 min of irradiation at λ = 280 nm.

A sample of homoannulene (L)-**6** and *iso*homoannulenes (L)-**7** which was irradiated for 3 h with an LED of λ = 280 nm was subsequently irradiated with an LED of λ = 430 nm until $PSS_{430\ nm}$ was reached (Equation S3). Samples were taken and subjected to HPLC analysis on a chiral stationary phase. For the HPLC analysis CHIRALPAK AD-H with *n*-heptane/*i*PrOH = 90:10, a flowrate of 0.4 mL min^{-1} at 40 °C was used.

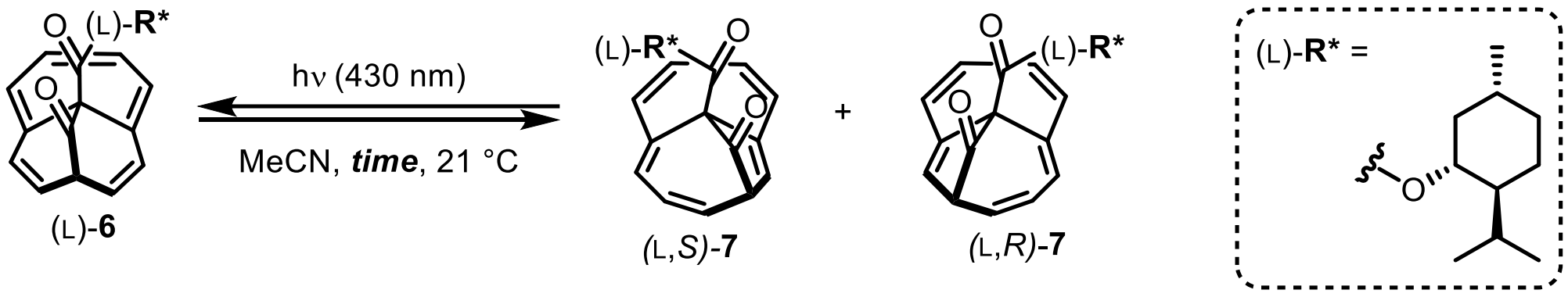

Equation S3. Photoswitching of homoannulene (L)-**6** using an LED of λ = 430 nm.

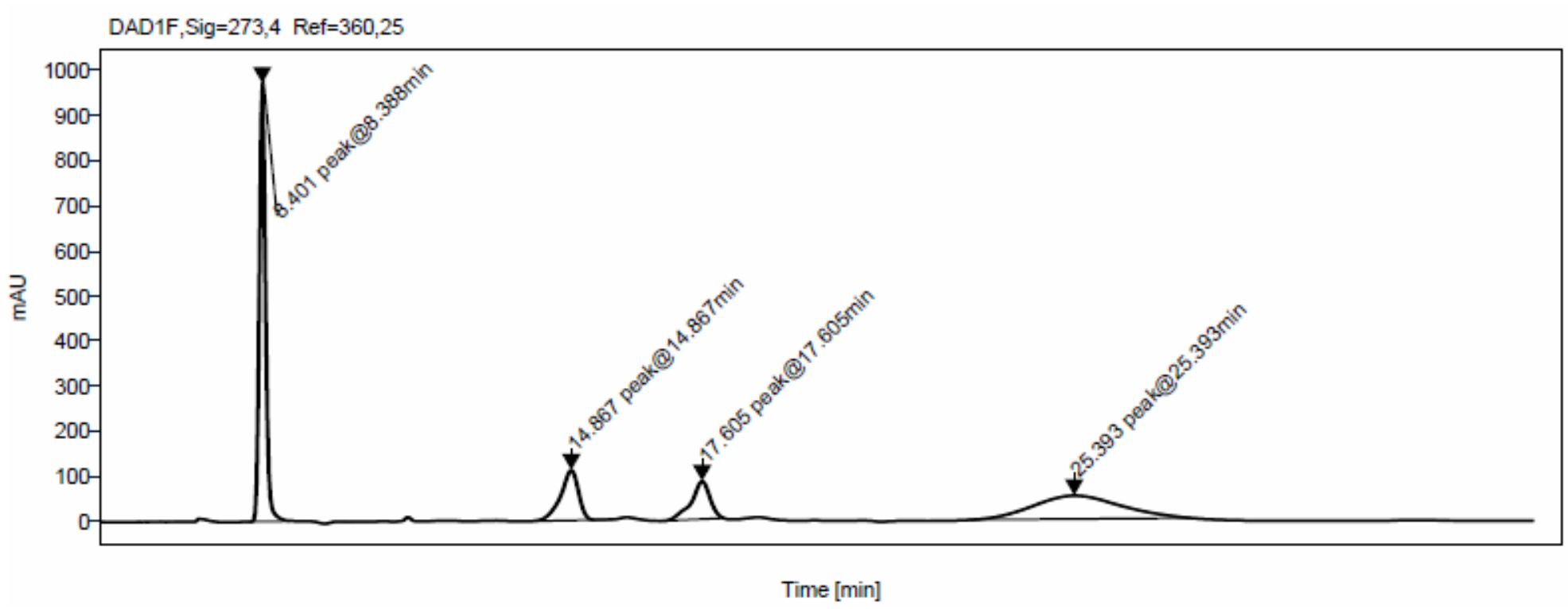

Signal: DAD1F,Sig=273,4 Ref=360,25

Name	RT [min]	Area	Peak Area Percent
peak@8.388min	8.40	8761.000	42.53
peak@14.867min	14.87	2900.139	14.08
peak@17.605min	17.60	2303.333	11.18
peak@25.393min	25.39	6634.188	32.21

Figure S3.7. Chromatogram of irradiated sample of the mixture of homoannulene (L)-**6** and *iso*homoannulenes (L)-**7** obtained at $PSS_{280\ nm}$.

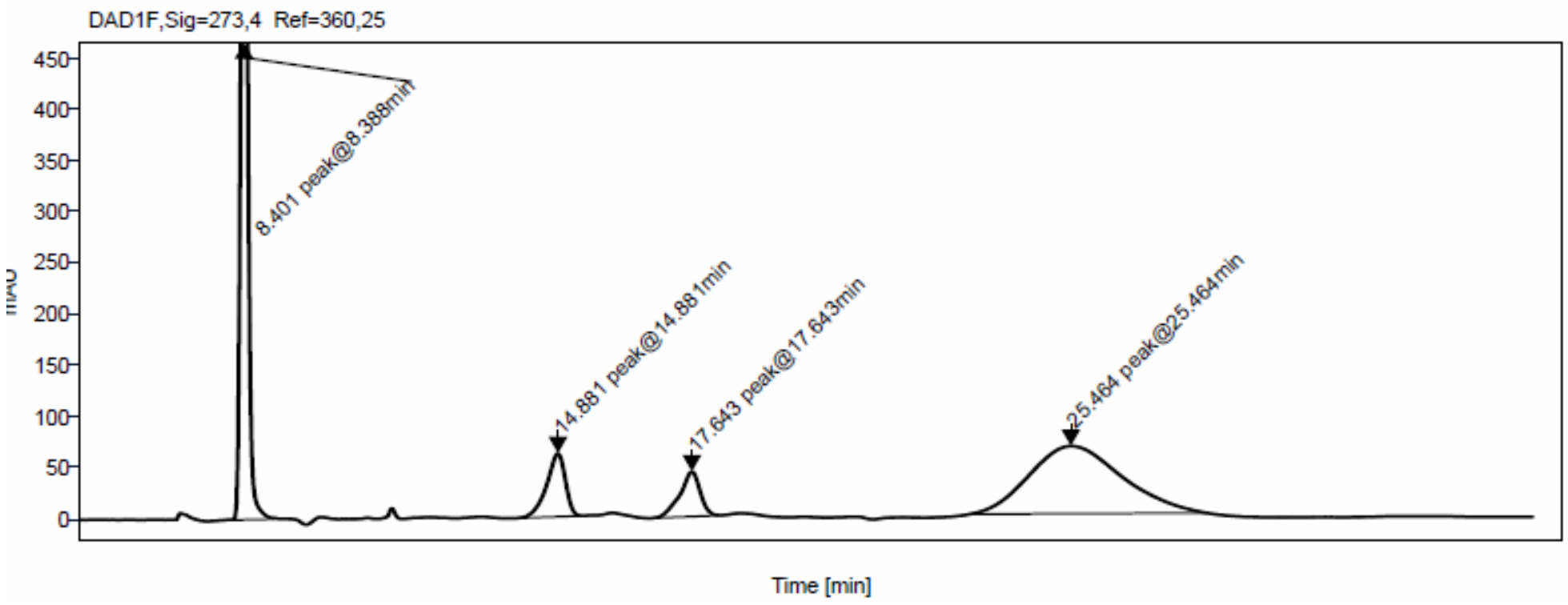

Signal: DAD1F,Sig=273,4 Ref=360,25

Name	RT [min]	Area	Peak Area Percent
peak@8.388min	8.40	9192.005	44.02
peak@14.881min	14.88	1663.096	7.96
peak@17.643min	17.64	1275.817	6.11
peak@25.464min	25.46	8751.664	41.91

Figure S3.8. Chromatogram of irradiated sample of the mixture of homoannulene (L)-**6** and *iso*homoannulenes (L)-**7** obtained at $PSS_{280\ nm}$ after 15 min of irradiation at λ = 430 nm.

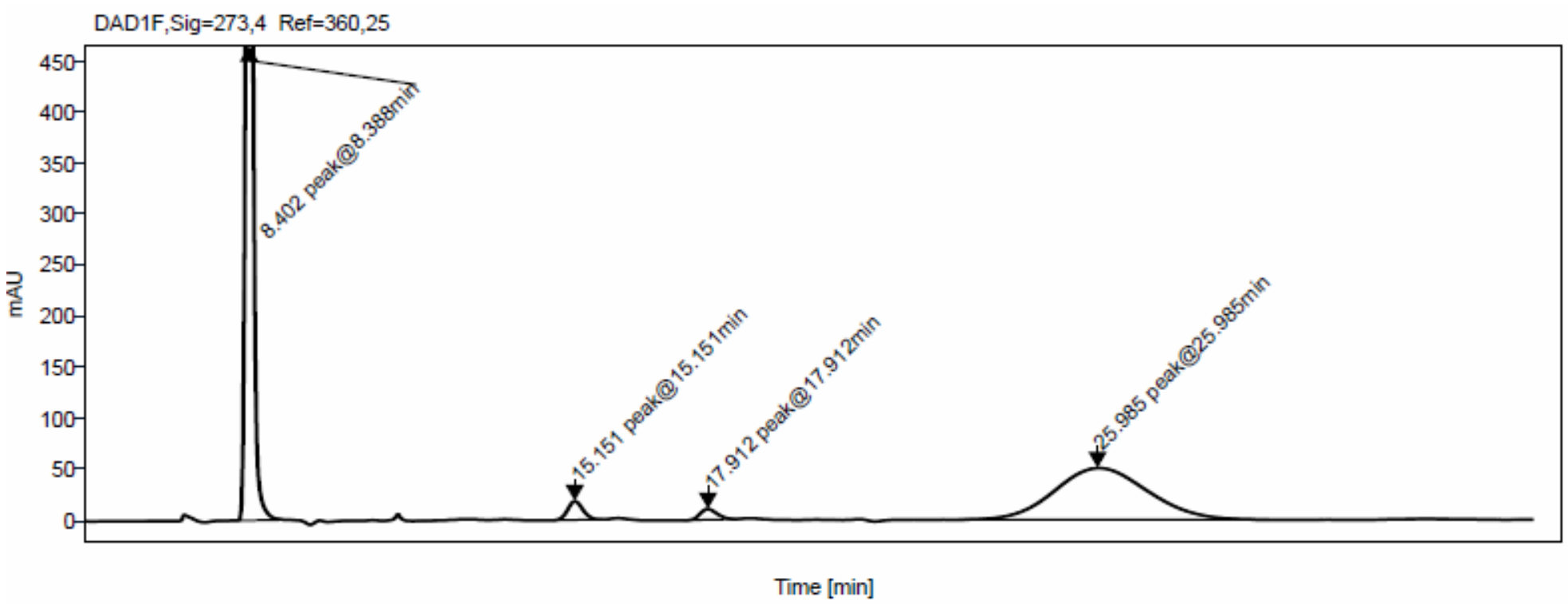

Signal: DAD1F,Sig=273,4 Ref=360,25

Name	RT [min]	Area	Peak Area Percent
peak@8.388min	8.40	11656.075	60.91
peak@15.151min	15.15	398.257	2.08
peak@17.912min	17.91	251.037	1.31
peak@25.985min	25.98	6830.738	35.70

Figure S3.9. Chromatogram of irradiated sample of the mixture of homoannulene (L)-**6** and *iso*homoannulenes (L)-**7** obtained at $PSS_{280\ nm}$ after 30 min of irradiation at λ = 430 nm.

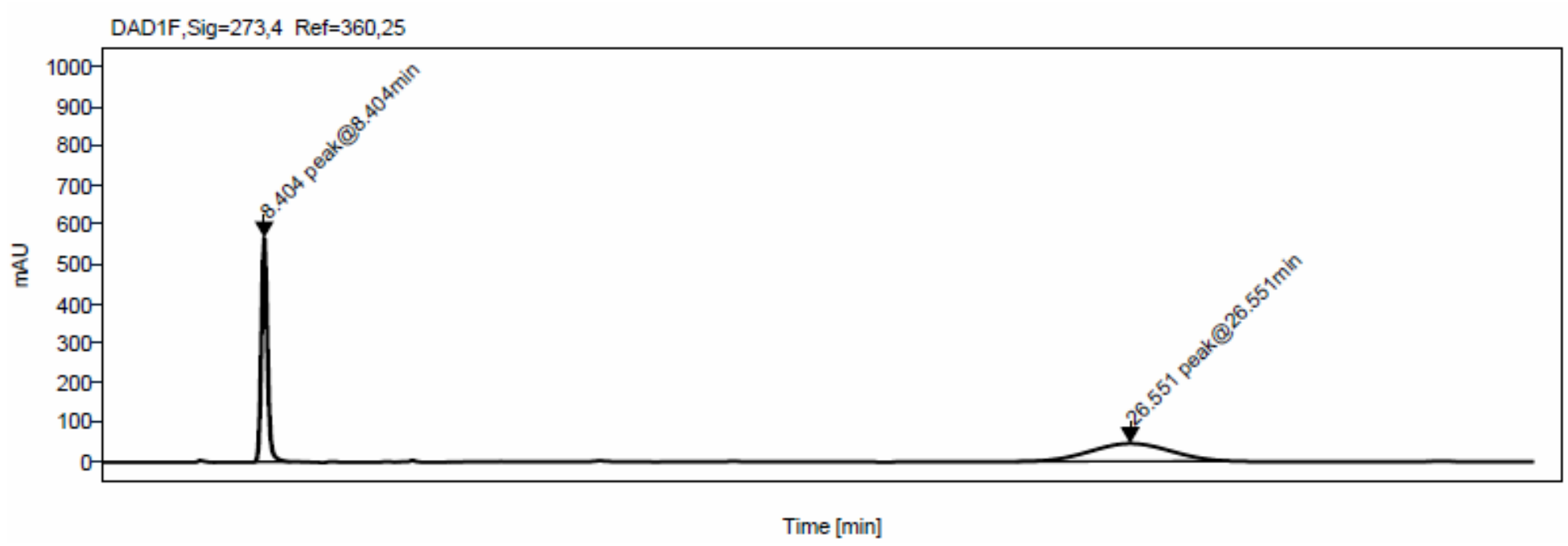

Signal: DAD1F,Sig=273,4 Ref=360,25

Name	RT [min]	Area	Peak Area Percent
peak@8.404min	8.40	4998.144	48.91
peak@26.551min	26.55	5220.071	51.09

Figure S3.10. Chromatogram of irradiated sample of the mixture of homoannulene (L)-**6** and *iso*homoannulenes (L)-**7** obtained at PSS$_{280\ nm}$ after 60 min of irradiation at λ = 430 nm.

Photoswitch-cycling using LEDs ("*photoswitching in small scale*")

To display multiple rounds of photoswitching of homoannulene (L)-**6** the compound was analysed by means of HPLC after every step of photoswitching. One cycle of photoswitching consists of 3 h irradiation at λ = 280 nm and 1 h irradiation at λ = 430 nm (Equation S4).

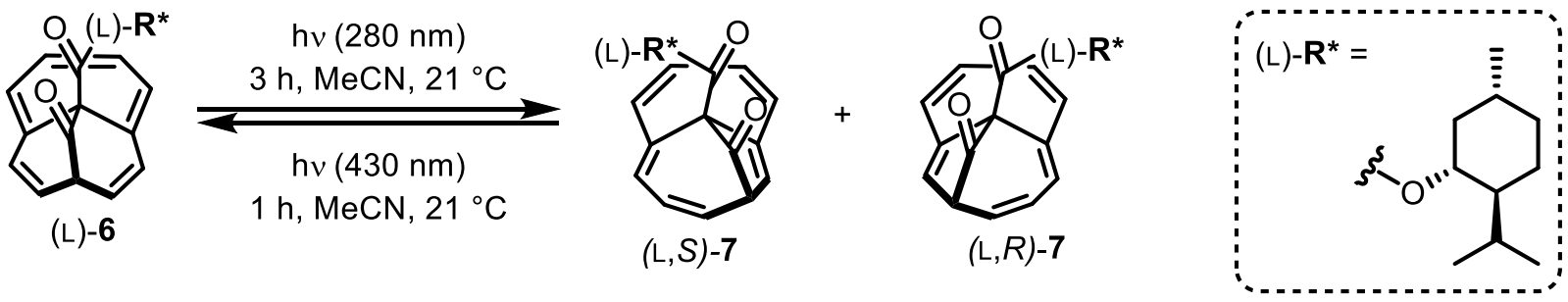

Equation S4. Forward and backward switching of homoannulene (L)-**6** by using LEDs. One cycle of photoswitching consists of 3 h irradiation at λ = 280 nm and 1 h irradiation at λ = 430 nm.

Samples were taken and subjected to HPLC analysis on a chiral stationary phase. For the HPLC analysis CHIRALPAK AD-H with *n*-heptane/*i*PrOH = 90:10, a flowrate of 0.4 mL · min^{-1} at 40 °C was used.

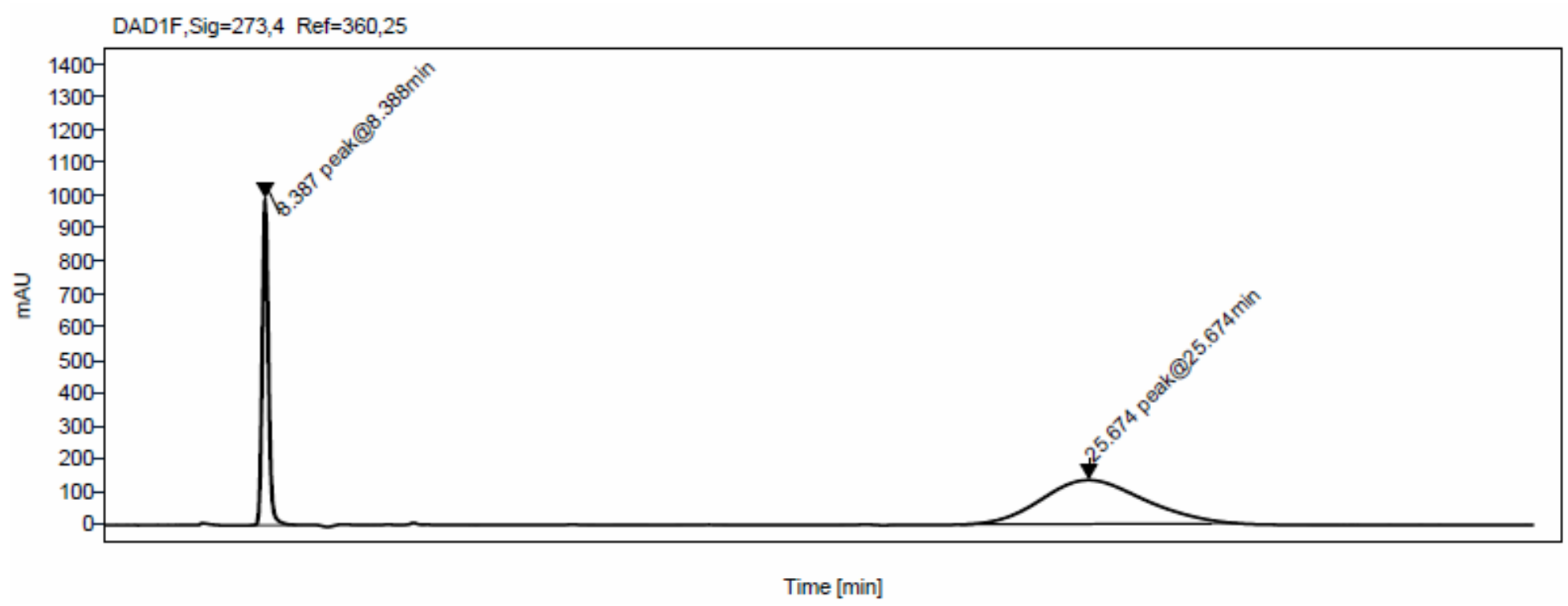

Signal: DAD1F,Sig=273,4 Ref=360,25

Name	RT [min]	Area	Peak Area Percent
peak@8.388min	8.39	8790.649	30.53
peak@25.674min	25.67	20002.907	69.47

Figure S3.11. Chromatogram of homoannulene (L)-**6** prior to photoswitching.

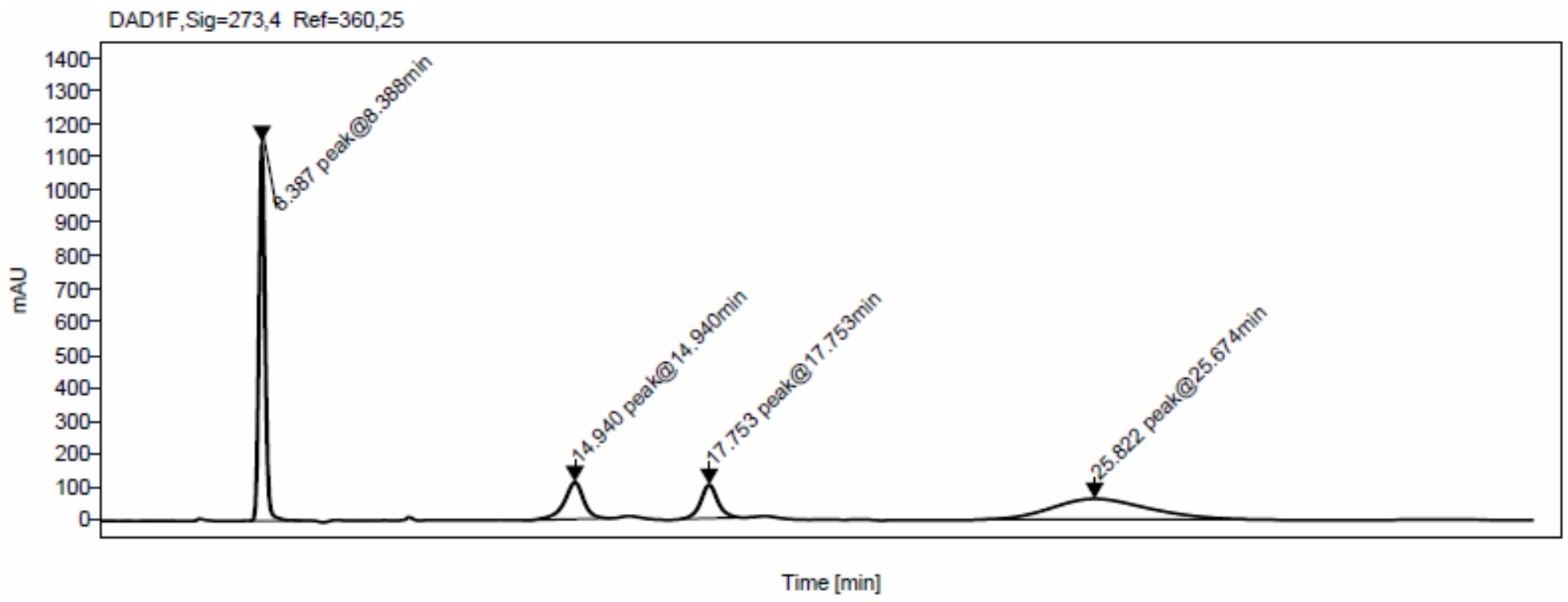

Signal:	DAD1F,Sig=273,4 Ref=360,25		
**Name**	**RT [min]**	**Area**	**Peak Area Percent**
peak@8.388min	8.39	10077.082	41.07
peak@14.940min	14.94	3172.239	12.93
peak@17.753min	17.75	2521.169	10.27
peak@25.674min	25.82	8768.818	35.73

Figure S3.12. First cycle of photoswitching: Chromatogram of a sample taken after irradiation at λ = 280 nm after 180 min.

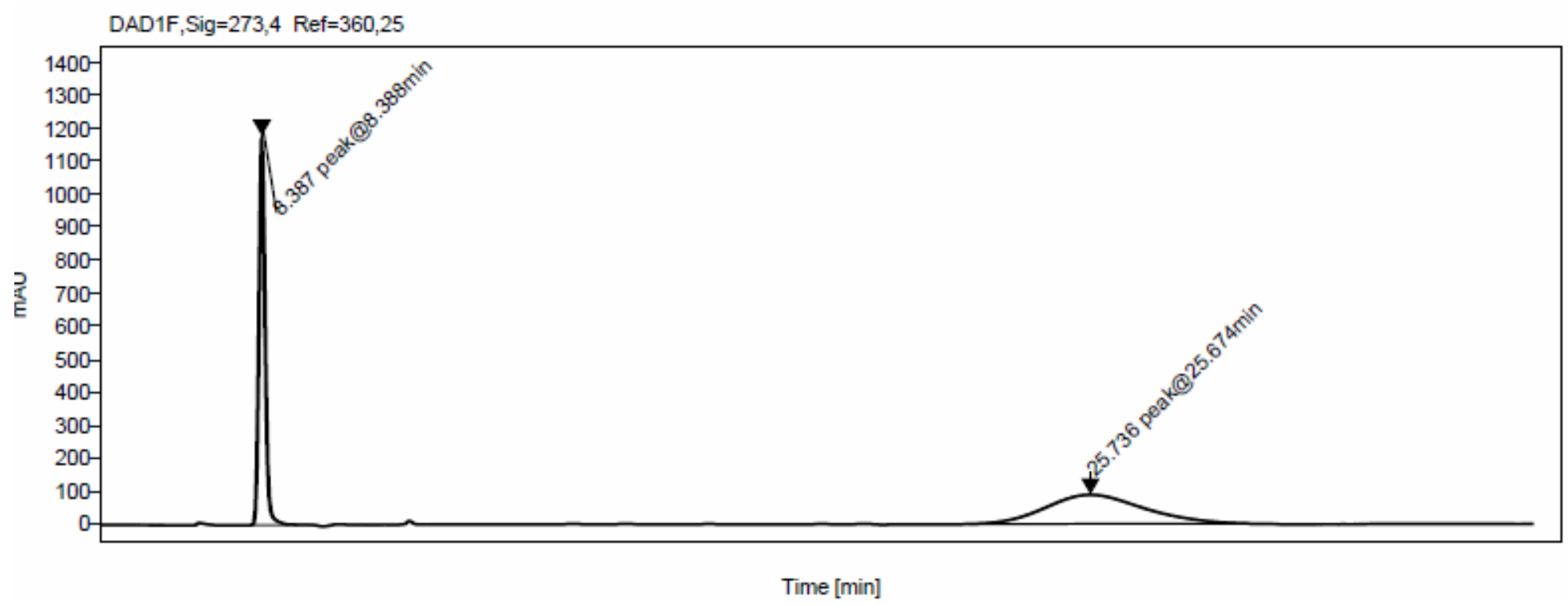

Signal:	DAD1F,Sig=273,4 Ref=360,25		
**Name**	**RT [min]**	**Area**	**Peak Area Percent**
peak@8.388min	8.39	10480.086	45.48
peak@25.674min	25.74	12565.407	54.52

Figure S3.13. First cycle of photoswitching: Chromatogram of a sample taken after irradiation at λ = 430 nm after 60 min.

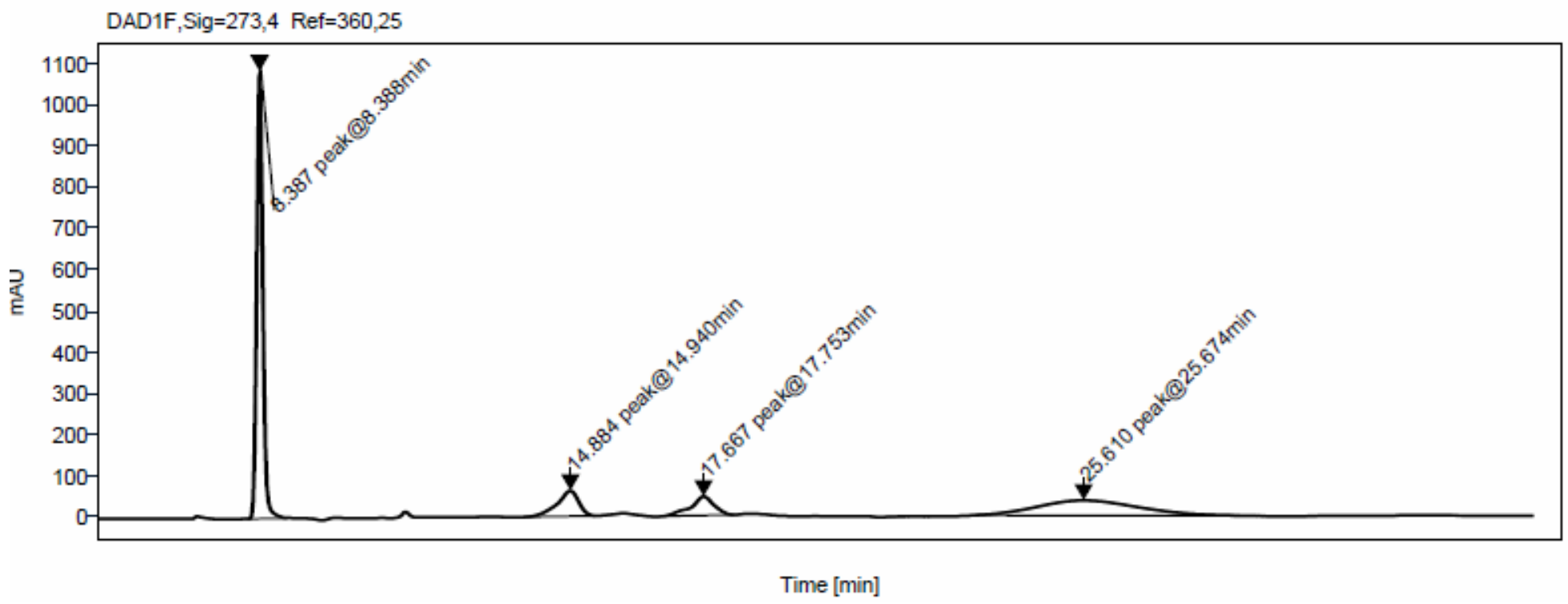

Signal: DAD1F,Sig=273,4 Ref=360,25

Name	RT [min]	Area	Peak Area Percent
peak@8.388min	8.39	9646.730	52.21
peak@14.940min	14.88	1949.564	10.55
peak@17.753min	17.67	1538.624	8.33
peak@25.674min	25.61	5343.090	28.92

Figure S3.14. Second cycle of photoswitching: Chromatogram of a sample taken after irradiation at λ = 280 nm after 180 min.

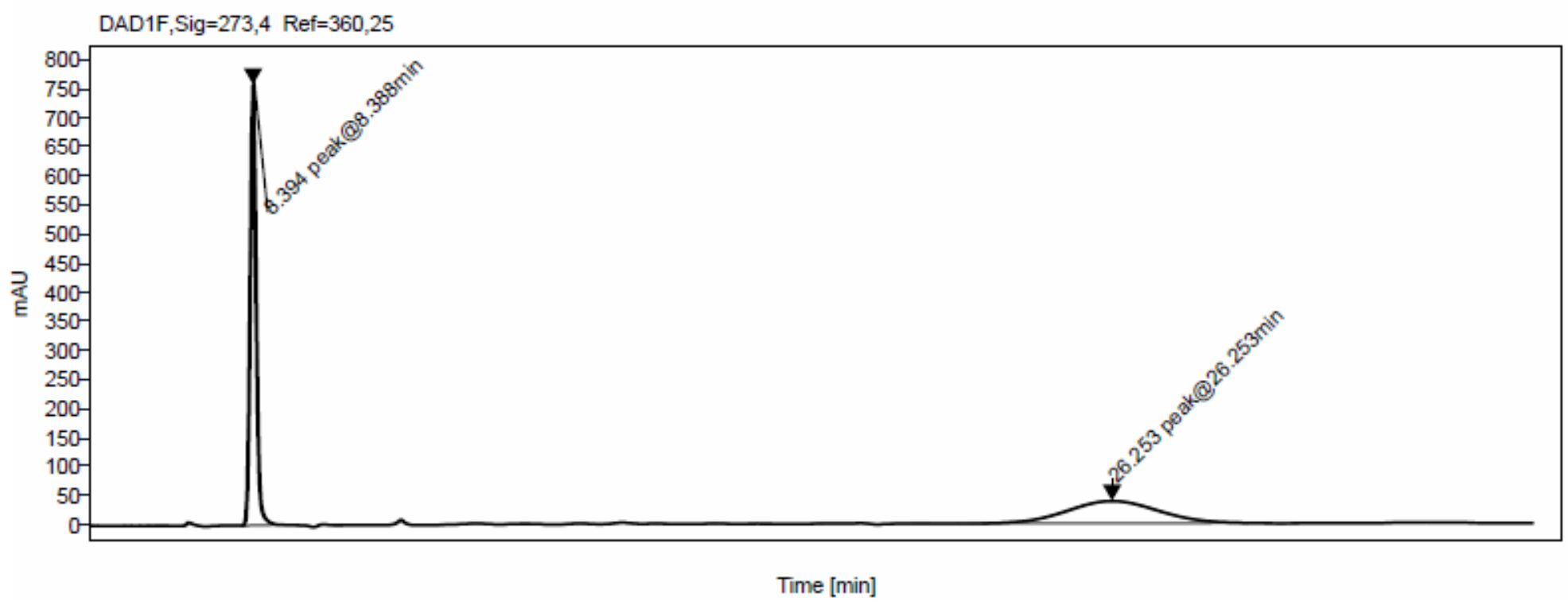

Signal: DAD1F,Sig=273,4 Ref=360,25

Name	RT [min]	Area	Peak Area Percent
peak@8.388min	8.39	6520.266	56.47
peak@26.253min	26.25	5025.894	43.53

Figure S3.15. Second cycle of photoswitching: Chromatogram of a sample taken after irradiation at λ = 430 nm after 60 min.

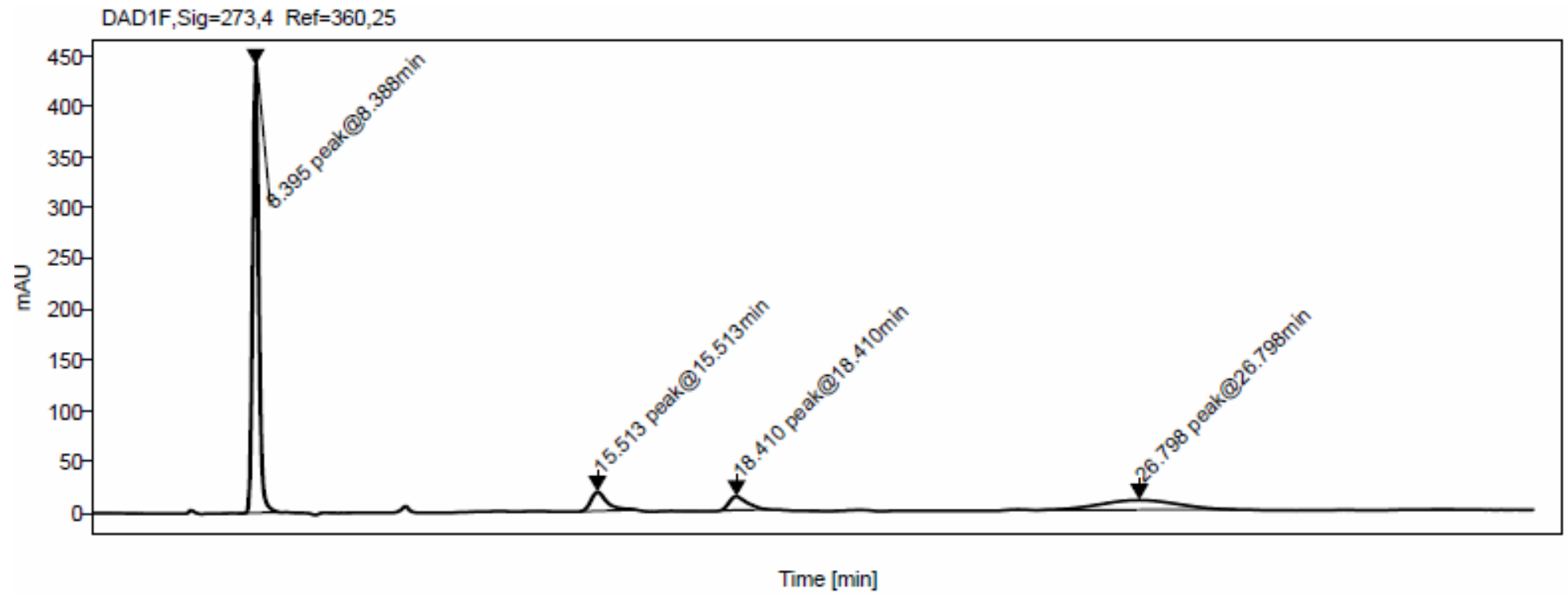

Signal: DAD1F,Sig=273,4 Ref=360,25

Name	RT [min]	Area	Peak Area Percent
peak@8.388min	8.40	3828.894	67.39
peak@15.513min	15.51	439.886	7.74
peak@18.410min	18.41	354.261	6.24
peak@26.798min	26.80	1058.523	18.63

Figure S3.16. Third cycle of photoswitching: Chromatogram of a sample taken after irradiation at λ = 280 nm after 180 min.

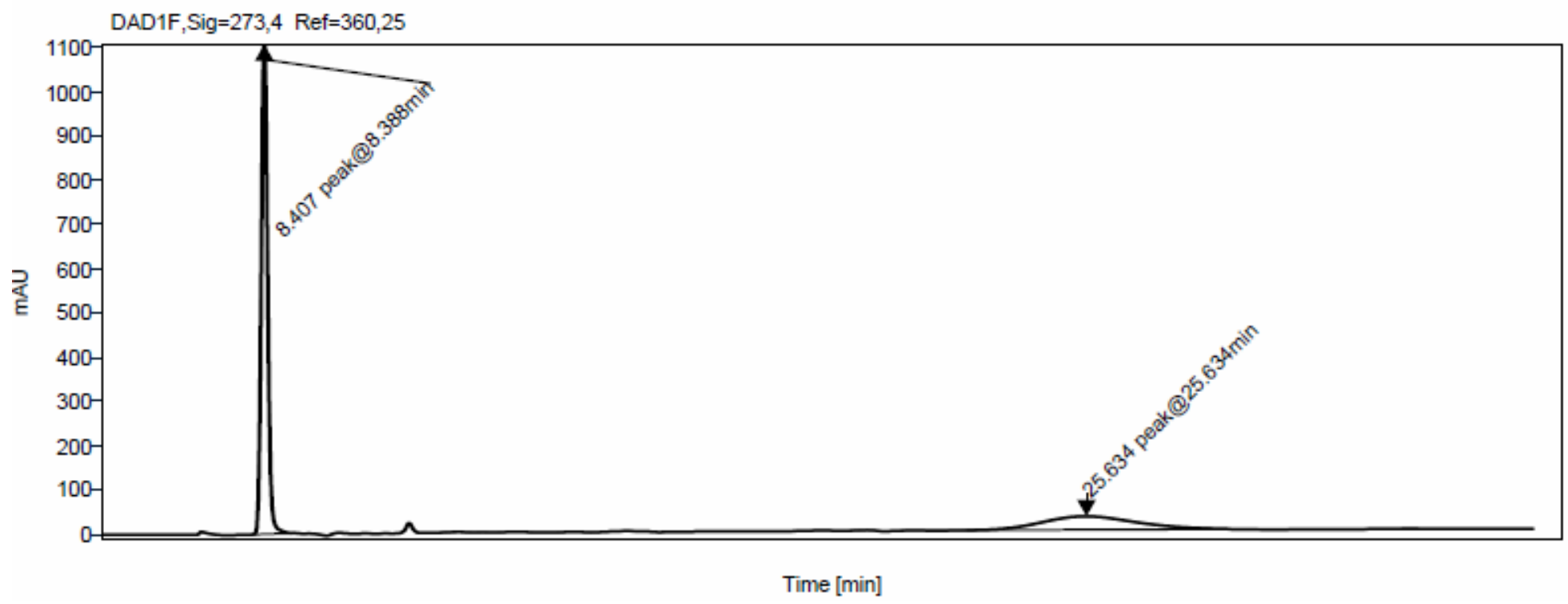

Signal: DAD1F,Sig=273,4 Ref=360,25

Name	RT [min]	Area	Peak Area Percent
peak@8.388min	8.41	10379.409	71.28
peak@25.634min	25.63	4182.543	28.72

Figure S3.17. Third cycle of photoswitching: Chromatogram of a sample taken after irradiation at λ = 430 nm after 60 min.

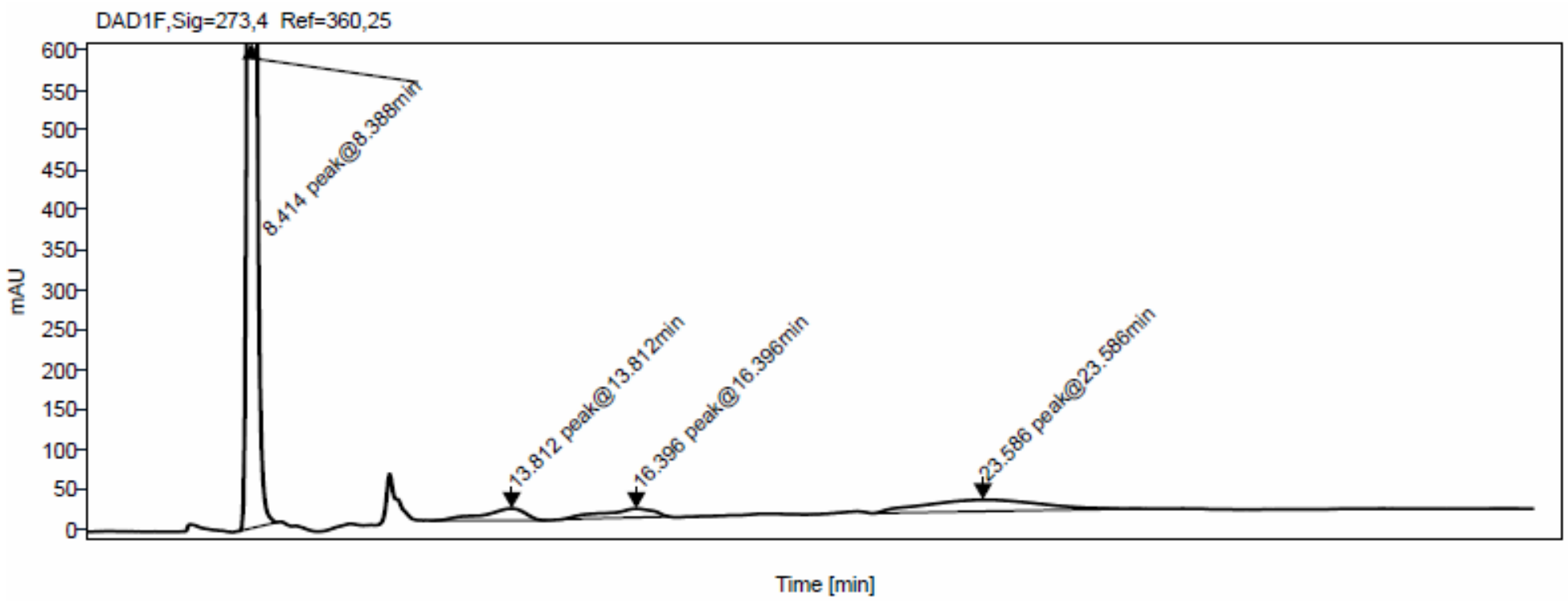

Signal: DAD1F,Sig=273,4 Ref=360,25

Name	RT [min]	Area	Peak Area Percent
peak@8.388min	8.41	23236.134	84.81
peak@13.812min	13.81	898.963	3.28
peak@16.396min	16.40	815.811	2.98
peak@23.586min	23.59	2445.998	8.93

Figure S3.18. Fourth cycle of photoswitching: Chromatogram of a sample taken after irradiation at λ = 280 nm after 180 min.

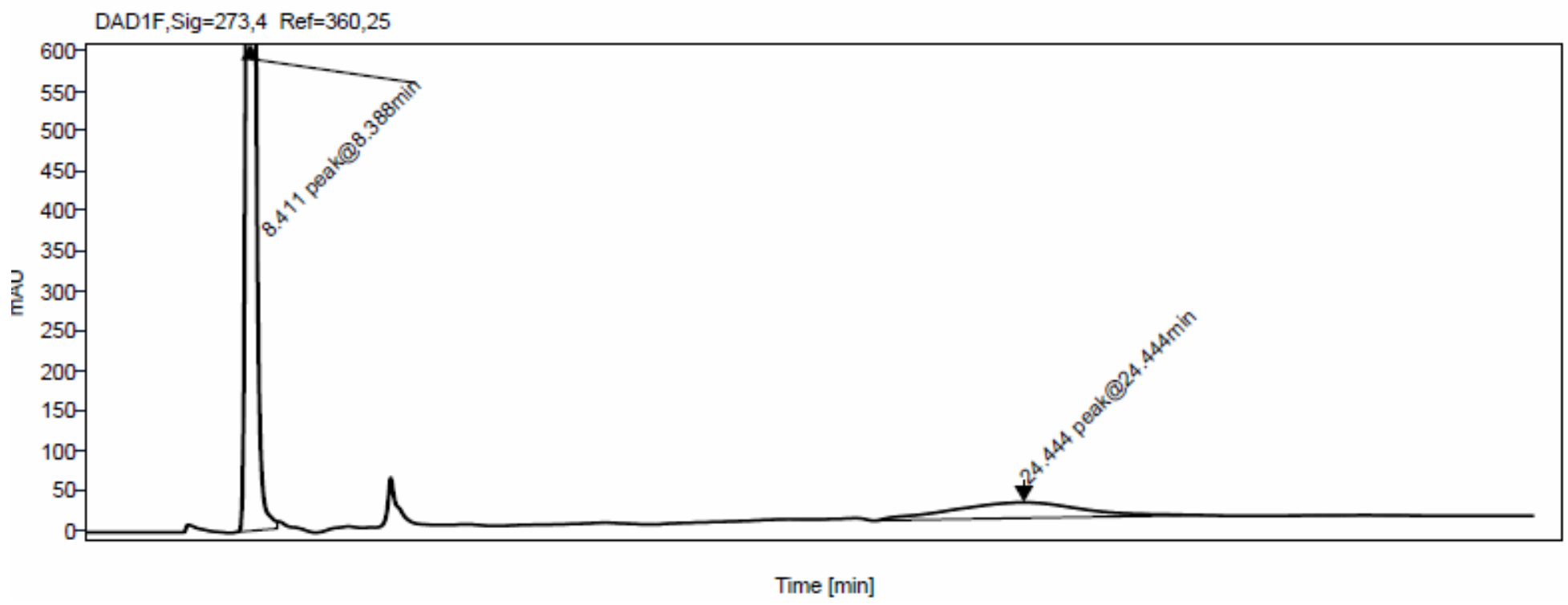

Signal: DAD1F,Sig=273,4 Ref=360,25

Name	RT [min]	Area	Peak Area Percent
peak@8.388min	8.41	23498.913	86.49
peak@24.444min	24.44	3669.743	13.51

Figure S3.19: Fourth cycle of photoswitching: Chromatogram of a sample taken after irradiation at λ = 430 nm after 60 min.

4. Computational geometry optimized coordinates of the investigated structures

Coordinates of *iso*-homoannulenes (L)-7 for determination of absolute configuration

***(*L*,S)*-7_c1** (HF = -1156.76201 hartree)

C	1.93174100	-0.11782300	-0.34216500
C	2.42910000	-1.51905500	-0.03128800
C	2.72620200	0.96009100	0.38615400
C	3.91049500	-1.68637200	-0.36384600
H	1.83124800	-2.24701500	-0.58360000
H	2.27316200	-1.71252600	1.03537900
O	0.54729400	-0.01321000	0.09291800
H	1.94730300	0.05277300	-1.41831500
C	4.20841800	0.79019600	0.03340500
C	2.18261900	2.38342700	0.14717000
H	2.61588000	0.75557200	1.45833100
C	4.72248100	-0.60990500	0.35114300
C	4.39587700	-3.08850500	-0.02196200
H	4.03329100	-1.53592600	-1.44339100
C	-0.40715000	-0.36334800	-0.75699200
H	4.79957300	1.53290100	0.57104800
H	4.35641200	0.98740300	-1.03253500
C	2.77893200	3.38088900	1.13865900
C	2.35872200	2.88654700	-1.28490100
H	1.10933300	2.33875100	0.34778500
H	5.77581600	-0.69272700	0.07103600
H	4.66996900	-0.78444400	1.43212000
H	3.82380700	-3.84854200	-0.55801300
H	5.44870400	-3.21365000	-0.28277900
H	4.29110600	-3.28379100	1.04861900
C	-1.78651900	-0.08803200	-0.17549100
O	-0.22314600	-0.75910800	-1.87872900
H	3.84781500	3.52867600	0.97086100
H	2.29598500	4.35478500	1.03769500
H	2.64388000	3.04571800	2.16936500
H	1.85811000	3.84896500	-1.40652300
H	3.41193600	3.03318700	-1.53255500
H	1.93247900	2.20308400	-2.02110800
C	-2.87461900	-0.95303300	-0.78138000
C	-1.97563000	-0.24554200	1.31490300
C	-4.14354000	-0.54489800	-0.96720900
C	-2.53641000	-2.36895500	-0.91880400
C	-2.61173200	0.82191600	1.82476100
C	-1.63144300	-1.48697300	1.97409000
C	-4.82186800	0.64735500	-0.48323300
H	-4.81357900	-1.28279600	-1.39661700
C	-1.90284800	-3.08520800	0.02287200
H	-2.87150800	-2.87476100	-1.81739300
C	-2.95091700	1.83763500	0.76913400
H	-2.94603500	0.90716100	2.85013400

C	-1.53595500	-2.68770300	1.37638000
H	-1.44109300	-1.43076000	3.04000300
C	-4.37900600	1.63661900	0.30728000
H	-5.87931100	0.67188200	-0.72112800
H	-1.73138200	-4.13235000	-0.19831300
H	-2.77585600	2.87582500	1.04599900
H	-1.22406000	-3.51249800	2.00734000
H	-5.09721500	2.37022400	0.65282000
C	-2.05933100	1.40931600	-0.36615200
O	-1.61808300	2.09644900	-1.24436900

***(L,S)*-7_c2 (HF = -1156.7484395 hartree)**

C	1.91883500	-0.24498100	-0.30578700
C	2.27437400	-1.71960100	-0.21436100
C	2.78306800	0.61765100	0.60640000
C	3.74370600	-1.97437700	-0.54469700
H	1.63048900	-2.29165200	-0.88439300
H	2.06859500	-2.06184400	0.80505300
O	0.52973400	-0.07541100	0.10236800
H	1.99469400	0.09281600	-1.33910400
C	4.25526100	0.36223900	0.26110200
C	2.38855100	2.10778800	0.59433500
H	2.61499200	0.25460200	1.62792600
C	4.62731000	-1.11343600	0.35313300
C	4.08376200	-3.45420200	-0.43102900
H	3.91643000	-1.66282400	-1.58219700
C	-0.41075700	-0.30493600	-0.79945300
H	4.89230000	0.94711300	0.92602800
H	4.45923100	0.71503200	-0.75432400
C	3.04710100	2.86884700	1.74360300
C	2.65499300	2.81431300	-0.73402800
H	1.31112200	2.13794400	0.76764900
H	5.67766800	-1.25064300	0.08319800
H	4.52147000	-1.45575700	1.38915900
H	3.46219400	-4.05668600	-1.09656400
H	5.12880300	-3.63769400	-0.68881600
H	3.92361200	-3.81058200	0.59002700
C	-1.80950600	-0.27190800	-0.19746500
O	-0.20603100	-0.59457500	-1.95158300
H	4.12934500	2.93957300	1.61605400
H	2.65908700	3.88791200	1.79859100
H	2.85298300	2.38498000	2.70339200
H	2.25527100	3.82979600	-0.70334600
H	3.72408600	2.89123900	-0.94197400
H	2.18482600	2.30529700	-1.57716900
C	-2.04397800	0.81058300	0.83967500
C	-2.93941100	-0.08530800	-1.18292600
C	-2.88197300	0.68494600	1.88529400
C	-1.49816100	2.12267600	0.49612000
C	-3.90741100	-0.99115700	-0.96760300
C	-2.96048100	1.04797400	-2.08275900
C	-3.87650100	-0.33810100	2.16744100
H	-2.95478200	1.55326100	2.53234400
C	-1.57740800	2.67792800	-0.72365900
H	-1.05319300	2.69942700	1.29914300
C	-3.58412500	-1.90498200	0.18138200

H	-4.86060500	-1.00570900	-1.47917400
C	-2.30453400	2.20552700	-1.89495600
H	-3.56368000	0.94149800	-2.97736200
C	-4.25355500	-1.40238500	1.44312700
H	-4.46922700	-0.13568200	3.05239300
H	-1.14326500	3.66503200	-0.83375500
H	-3.80848100	-2.95759200	0.01733100
H	-2.39081500	2.94126900	-2.68660900
H	-5.11267700	-1.96820800	1.78243100
C	-2.10481500	-1.67618700	0.34712400
O	-1.29827000	-2.44568700	0.78846400

***(*L*,R)*-7_c1 (HF = -1156.7494018 hartree)**

C	-1.92293400	0.09590200	-0.34252700
C	-2.40887200	1.49877300	-0.01995500
C	-2.70364600	-0.97972100	0.40384500
C	-3.89750800	1.67093900	-0.31535100
H	-1.82286400	2.22545300	-0.58615500
H	-2.22653000	1.69191500	1.04248500
O	-0.52870000	-0.01462300	0.06105900
H	-1.96313000	-0.07457200	-1.41819600
C	-4.19427300	-0.80240100	0.09249300
C	-2.17480500	-2.40516300	0.14501500
H	-2.56238200	-0.77812000	1.47296800
C	-4.69450400	0.59943300	0.42329600
C	-4.36838500	3.07595700	0.03492700
H	-4.04878200	1.51810400	-1.39098700
C	0.40411300	0.36050400	-0.79700800
H	-4.77325200	-1.54370000	0.64537800
H	-4.37193600	-0.99849900	-0.96904600
C	-2.72166400	-3.39530800	1.17174300
C	-2.42943100	-2.91508900	-1.27297400
H	-1.09240100	-2.36137300	0.28331600
H	-5.75478900	0.68614500	0.17190300
H	-4.61167000	0.77528600	1.50217000
H	-3.80745100	3.83222500	-0.51798600
H	-5.42715400	3.20463200	-0.19878300
H	-4.23508000	3.27385500	1.10186100
C	1.80753200	0.10710000	-0.26234500
O	0.19540600	0.77464700	-1.90986900
H	-3.80031900	-3.53254700	1.06854600
H	-2.25556500	-4.37445200	1.04465600
H	-2.52210600	-3.06030600	2.19202200
H	-1.92755800	-3.87330100	-1.42039400
H	-3.49368500	-3.07198900	-1.46020900
H	-2.05222400	-2.23008400	-2.03438500
C	2.91089300	0.91572200	-0.90370400
C	1.98261000	0.35978200	1.22372900
C	3.93847100	0.12169000	-1.24655100
C	2.84878100	2.36129200	-0.93826800
C	2.83941900	-0.31809400	2.00884700
C	1.34106700	1.57744600	1.71973900
C	3.68862800	-1.31681800	-0.88774000
H	4.88493800	0.47424200	-1.63446500
C	2.12203600	3.13113000	-0.11072000
H	3.44899700	2.85108900	-1.69679200

C	3.90093000	-1.24549300	1.64911000
H	2.86268700	-0.01144200	3.04973300
C	1.37365000	2.75884700	1.08347900
H	0.85324200	1.52315900	2.68657800
C	4.33716200	-1.63681700	0.44255500
H	3.98004600	-2.04292000	-1.64471000
H	2.15361600	4.19896400	-0.29593800
H	4.48876300	-1.58255800	2.49540100
H	0.86903500	3.58459700	1.57173800
H	5.23379500	-2.24315800	0.39959600
C	2.19893000	-1.31998500	-0.66650200
O	1.44636600	-2.24232300	-0.81007800

***(L,R)*-7_c2 (HF = -1156.7487916 hartree)**

C	1.92316300	-0.26301700	-0.32688400
C	2.28312100	-1.73661100	-0.24667000
C	2.79711300	0.60164000	0.57355900
C	3.74927700	-1.98601300	-0.59526800
H	1.63215400	-2.30625400	-0.91252100
H	2.08969200	-2.08452900	0.77373500
O	0.54310600	-0.10093900	0.10819200
H	1.97907300	0.07821500	-1.36023200
C	4.26464600	0.35309400	0.20571600
C	2.39092900	2.08902300	0.57006200
H	2.64626900	0.23553200	1.59674700
C	4.64196200	-1.12196100	0.29103400
C	4.09628700	-3.46458300	-0.48674300
H	3.90707800	-1.67340700	-1.63477000
C	-0.41858300	-0.27390100	-0.78744300
H	4.90992300	0.93916400	0.86163500
H	4.45211200	0.70748100	-0.81234700
C	3.05995000	2.85195700	1.71197700
C	2.63623400	2.79992100	-0.76020400
H	1.31499900	2.11190800	0.75774000
H	5.68880300	-1.25610400	0.00642000
H	4.55169100	-1.46519200	1.32824600
H	3.46781000	-4.06913400	-1.14383600
H	5.13831200	-3.64407800	-0.75904200
H	3.95163400	-3.82181900	0.53632400
C	-1.79260700	-0.23539800	-0.13289600
O	-0.24117000	-0.51981300	-1.95223900
H	4.14024200	2.92780900	1.57106800
H	2.66786800	3.86910900	1.77306000
H	2.88008300	2.36604500	2.67351700
H	2.22916600	3.81225200	-0.72292500
H	3.70222900	2.88537900	-0.98056900
H	2.16029300	2.28887400	-1.59890000
C	-1.99974200	0.69454000	1.03920700
C	-2.90648300	0.12703900	-1.09586700
C	-2.59908000	0.04880600	2.05314300
C	-1.71153600	2.10748800	0.91940900
C	-4.15843200	-0.36028600	-1.01426600
C	-2.62030400	1.25539100	-1.98072600
C	-2.89302700	-1.38680200	1.71663100
H	-2.93866700	0.51875600	2.96654200
C	-1.65756700	2.79920900	-0.23243400

H	-1.53040600	2.64266300	1.84477900
C	-4.79708200	-1.12404500	0.04627600
H	-4.85160400	0.00494600	-1.76526600
C	-2.02475400	2.38999700	-1.58208000
H	-2.96483200	1.18250000	-3.00618100
C	-4.32459400	-1.51463600	1.23960300
H	-2.68195700	-2.10539500	2.50668000
H	-1.38456600	3.84527200	-0.14928000
H	-5.85150400	-1.30806800	-0.12605200
H	-1.89089800	3.15788700	-2.33524600
H	-5.01853000	-1.96854900	1.93661000
C	-2.01219800	-1.60781600	0.51566400
O	-1.54651500	-2.64543300	0.13520500

Coordinates of homoannulene methyl ester 4 for calculations on bond rotations

O	-1.76374	-0.66328	-1.62563
C	-1.32046	-0.66931	-0.50051
O	-1.93104	-1.22487	0.54115
C	-3.18879	-1.85450	0.26968
C	0.07327	-0.14925	-0.12616
C	1.02000	-1.34652	-0.26921
O	0.64569	-2.45424	-0.58641
C	0.55148	1.00401	-0.98716
C	-0.28558	2.06080	-1.21582
C	-1.46169	2.35558	-0.46705
C	-1.66119	2.03630	0.85883
C	-0.72132	1.36143	1.69094
C	0.21403	0.46373	1.25564
C	1.42669	0.17194	2.00457
C	2.48197	-0.45762	1.45707
C	2.44417	-0.97925	0.03102
C	2.84158	0.11981	-0.93918
C	1.94077	0.99679	-1.41779
H	-3.52970	-2.24677	1.22571
H	-3.05699	-2.66219	-0.45273
H	-3.89942	-1.12328	-0.12074
H	0.00559	2.77918	-1.97931
H	-2.17572	3.03716	-0.92155
H	-2.51545	2.49334	1.35132
H	1.49807	0.57599	3.01062
H	3.40614	-0.57996	2.01205
H	3.07917	-1.85869	-0.08561
H	3.88960	0.19576	-1.20918
H	2.26148	1.80101	-2.07409
H	-0.69970	1.64392	2.74153

Coordinates of homoannulene methyl ester (L)-6 for calculations on bond rotation

O	-1.42999	2.10736	-1.05436
C	-2.18565	1.35177	-0.48375
C	-1.84435	-0.11193	-0.18459
C	-0.44805	-0.35113	-0.77589
O	0.49755	-0.00533	0.08585
C	1.87807	-0.09819	-0.33516
H	1.91050	0.05503	-1.41818
C	2.39256	-1.49256	0.00135
C	3.88171	-1.63861	-0.32864
H	4.00208	-1.50298	-1.41392
C	4.39412	-3.03008	0.03848
C	4.67746	-0.53234	0.37211
C	4.13960	0.85973	0.03277
C	2.65086	1.00537	0.38623
H	2.54540	0.80871	1.46466
C	2.06683	2.41330	0.12863
C	2.17589	2.87385	-1.32854
C	2.68378	3.45437	1.06825
O	-0.25643	-0.71560	-1.91405
C	-2.91744	-1.02690	-0.74194
C	-2.67468	-2.37011	-0.82437
C	-1.63031	-3.06011	-0.14255
C	-1.08514	-2.68273	1.06549
C	-1.48086	-1.54279	1.82385
C	-1.99503	-0.38876	1.30048
C	-2.80663	0.53120	2.08205
C	-3.56081	1.49066	1.51588
C	-3.55156	1.72602	0.01525
C	-4.54975	0.80565	-0.66579
C	-4.21815	-0.44504	-1.03410
H	1.80263	-2.23722	-0.54446
H	2.23495	-1.67237	1.07415
H	5.45220	-3.14073	-0.22089
H	3.83305	-3.81036	-0.48640
H	4.29137	-3.20737	1.11590
H	5.73568	-0.60277	0.09420
H	4.62358	-0.69061	1.45931
H	4.28303	1.04470	-1.04008
H	4.72082	1.62081	0.56276
H	0.99911	2.34955	0.36840
H	3.22036	3.00542	-1.63289
H	1.67177	3.83731	-1.45374
H	1.70261	2.17037	-2.02003
H	2.15575	4.40941	0.97946
H	3.73758	3.64042	0.83182
H	2.62391	3.13267	2.11394
H	-3.37711	-2.97623	-1.39287
H	-1.37042	-4.04844	-0.51236
H	-0.43714	-3.40223	1.55922
H	-1.43808	-1.63079	2.90765
H	-2.86778	0.35325	3.15218
H	-4.22552	2.10275	2.11641

H	-3.75213	2.77182	-0.22225
H	-5.55644	1.18115	-0.81645
H	-4.96565	-1.09659	-1.47803

Coordinates of *iso*-homoannulene *(*L,*S)*-7 for calculations on bond rotations

O	-0.21323	-0.65988	-1.93139
C	-0.40334	-0.31992	-0.78540
O	0.54552	-0.01326	0.09229
C	1.92876	-0.12051	-0.31894
H	1.96723	0.01147	-1.40437
C	2.43468	-1.51018	0.04462
C	3.92195	-1.66743	-0.29038
H	4.03601	-1.55581	-1.37903
C	4.43300	-3.05186	0.10418
C	4.72515	-0.54869	0.38173
C	4.19033	0.84020	0.02287
C	2.70371	0.99377	0.38239
H	2.60627	0.81527	1.46476
C	2.11519	2.39698	0.10799
C	2.11158	2.79347	-1.37216
C	2.81120	3.47064	0.95073
C	-1.78086	-0.08149	-0.17789
C	-2.06752	1.42720	-0.26553
O	-1.64613	2.17881	-1.10965
C	-2.87233	-0.92549	-0.81540
C	-4.16130	-0.52576	-0.94090
C	-4.84153	0.62567	-0.36045
C	-4.38400	1.57917	0.48134
C	-2.94271	1.77462	0.91782
H	-2.77222	2.79780	1.25767
C	-2.57296	0.69409	1.90213
C	-1.93688	-0.33950	1.30569
C	-1.54738	-1.61634	1.87495
C	-1.43006	-2.77747	1.18763
C	-1.82791	-3.09319	-0.18244
C	-2.51211	-2.32541	-1.06017
H	1.84037	-2.26247	-0.48631
H	2.28045	-1.66931	1.12097
H	3.86681	-3.84194	-0.40006
H	4.33616	-3.20524	1.18574
H	5.48917	-3.17083	-0.15907
H	5.78092	-0.62754	0.09719
H	4.67961	-0.68593	1.47215
H	4.33039	1.01153	-1.05276
H	4.77643	1.60476	0.54180
H	1.06929	2.35659	0.43582
H	1.53526	2.09530	-1.98650
H	3.12836	2.84836	-1.77696
H	1.65511	3.78097	-1.49280
H	3.83337	3.65788	0.60349
H	2.86080	3.18007	2.00610
H	2.26618	4.41796	0.88715

H	-4.83260	-1.24636	-1.40350
H	-5.90901	0.65004	-0.56390
H	-5.10474	2.27971	0.89343
H	-2.88595	0.71050	2.94050
H	-1.32569	-1.62482	2.93899
H	-1.07099	-3.63408	1.75266
H	-1.63423	-4.11924	-0.48320
H	-2.85987	-2.77548	-1.98641

Coordinates of *iso*-homoannulene *(*L,*R)*-7 for calculations on bond rotations

O	0.19931	-0.58914	1.94279
C	0.39782	-0.35979	0.77084
O	-0.54784	-0.21763	-0.15201
C	-1.92999	-0.31762	0.26949
H	-1.98095	0.00734	1.31326
C	-2.38231	-1.76764	0.17170
C	-3.85548	-1.90859	0.57256
H	-3.94749	-1.60278	1.62554
C	-4.32498	-3.35734	0.45385
C	-4.71558	-0.95895	-0.26839
C	-4.22504	0.48990	-0.19453
C	-2.75166	0.61845	-0.61433
H	-2.67058	0.24157	-1.64590
C	-2.20375	2.06411	-0.62717
C	-2.07730	2.69605	0.76276
C	-3.01788	2.96721	-1.55894
C	1.78245	-0.29008	0.13664
C	2.06967	-1.66611	-0.48736
O	1.63417	-2.72184	-0.09881
C	1.96527	0.63164	-1.05043
C	2.61204	-0.00772	-2.05117
C	2.96501	-1.42619	-1.68217
C	4.39850	-1.46843	-1.18263
C	4.84109	-1.02131	0.01369
C	4.15026	-0.26899	1.05413
C	2.86296	0.15052	1.11144
C	2.49539	1.27463	1.97885
C	1.83169	2.37186	1.55084
C	1.46917	2.74005	0.18465
C	1.59661	2.03244	-0.96258
H	-1.74902	-2.39022	0.81263
H	-2.24664	-2.11325	-0.86249
H	-3.71835	-4.02251	1.07697
H	-4.24815	-3.70452	-0.58351
H	-5.36967	-3.45867	0.76607
H	-4.35174	0.86296	0.83085
H	-4.84821	1.11889	-0.83724
H	-1.19115	1.99781	-1.04120
H	-3.04406	2.74362	1.27673
H	-1.70054	3.72057	0.67456
H	-1.37683	2.14955	1.40231
H	-3.17098	2.49741	-2.53707

H	-2.49722	3.91698	-1.71956
H	-4.00259	3.20113	-1.13928
H	4.81324	0.14805	1.80946
H	2.81751	1.23074	3.01600
H	1.62667	3.13734	2.29429
H	1.12356	3.76539	0.07889
H	1.39989	2.54012	-1.90346
H	2.94528	0.46313	-2.96969
H	-5.76034	-1.01653	0.05883
H	-4.69289	-1.29580	-1.31527
H	2.80116	-2.16998	-2.46399
H	5.12639	-1.90188	-1.86258
H	5.90488	-1.14617	0.19872
